# Identification of expressed resistance gene-like sequences by data mining in 454-derived transcriptomic sequences of common bean (*Phaseolus vulgaris *L.)

**DOI:** 10.1186/1471-2229-12-42

**Published:** 2012-03-23

**Authors:** Zhanji Liu, Mollee Crampton, Antonette Todd, Venu Kalavacharla

**Affiliations:** 1 College of Agriculture & Related Sciences, Delaware State University, Dover, DE 19901, USA; 2 Hi-Tech Research Center, Shandong Academy of Agricultural Sciences, Jinan 250100, China; 3 Department of Biological Sciences, Delaware State University, Dover, DE 19901, USA; 4 Center of Integrated Biological and Environmental Research (CIBER), Delaware State University, Dover, DE 19901, USA; 5 Department of Plant Pathology, University of Georgia, Tifton, GA 31793, USA; 6 Department of Biological Sciences, University of Delaware, Newark, DE 19711, USA

**Keywords:** Common bean (*Phaseolus vulgaris *L.), 454 pyrosequencing, ESTs, Resistance gene-like sequences, Molecular markers

## Abstract

**Background:**

Common bean (*Phaseolus vulgaris *L.) is one of the most important legumes in the world. Several diseases severely reduce bean production and quality; therefore, it is very important to better understand disease resistance in common bean in order to prevent these losses. More than 70 resistance (*R*) genes which confer resistance against various pathogens have been cloned from diverse plant species. Most *R *genes share highly conserved domains which facilitates the identification of new candidate *R *genes from the same species or other species. The goals of this study were to isolate expressed *R *gene-like sequences (RGLs) from 454-derived transcriptomic sequences and expressed sequence tags (ESTs) of common bean, and to develop RGL-tagged molecular markers.

**Results:**

A data-mining approach was used to identify tentative *P. vulgaris R *gene-like sequences from approximately 1.69 million 454-derived sequences and 116,716 ESTs deposited in GenBank. A total of 365 non-redundant sequences were identified and named as common bean (*P. vulgaris *= Pv) resistance gene-like sequences (PvRGLs). Among the identified PvRGLs, about 60% (218 PvRGLs) were from 454-derived sequences. Reverse transcriptase-polymerase chain reaction (RT-PCR) analysis confirmed that PvRGLs were actually expressed in the leaves of common bean. Upon comparison to *P. vulgaris *genomic sequences, 105 (28.77%) of the 365 tentative PvRGLs could be integrated into the existing common bean physical map. Based on the syntenic blocks between common bean and soybean, 237 (64.93%) PvRGLs were anchored on the *P. vulgaris *genetic map and will need to be mapped to determine order. In addition, 11 sequence-tagged-site (STS) and 19 cleaved amplified polymorphic sequence (CAPS) molecular markers were developed for 25 unique PvRGLs.

**Conclusions:**

In total, 365 PvRGLs were successfully identified from 454-derived transcriptomic sequences and ESTs available in GenBank and about 65% of PvRGLs were integrated into the common bean genetic map. A total of 30 RGL-tagged markers were developed for 25 unique PvRGLs, including 11 STS and 19 CAPS markers. The expressed PvRGLs identified in this study provide a large sequence resource for development of RGL-tagged markers that could be used further for genetic mapping of disease resistant candidate genes and quantitative trait locus/loci (QTLs). This work also represents an additional method for identifying expressed RGLs from next generation sequencing data.

## Background

Plant resistance genes (*R *genes) play an important role in direct or indirect recognition of proteins encoded by specific avirulence (*Avr*) genes of pathogens [1]. In recent years, a large number of *R *genes which confer resistance to diverse pathogens have been cloned from different plant species by using either map-based cloning or transposon tagging approaches [2-5]. Based on the amino acid sequence comparison among cloned *R *genes, several highly conserved domains, such as NBS, LRR, protein kinase (PK), transmembrane domain (TM), and TIR were identified as the majority of known *R *genes [6-9]. These distinct conserved domains provide scientists a convenient way to identify and then clone additional *R *genes and RGLs or resistance gene analogs (RGAs).

The largest class consists of *R *genes which contain NBS and LRR domains (NBS-LRR). This class can be further divided into two sub-classes, TIR-NBS-LRR and non-TIR-NBS-LRR, based on the presence or absence of a TIR domain at the N-terminus [7,10]. The second class of *R *genes is comprised of those with LRR and PK domains (LRR-PK), such as *Fls2 *in *Arabidopsis *and *Xa21 *in rice [11,12]. The third class contains those with a large extracellular LRR domain, for example, *Cf-4 *in tomato and *Vf1 *in apple [13,14]. The fourth class contains those only with the PK domain, such as *Pto *in tomato and *Pbs1 *in *Arabidopsis *[15,16]. And finally, the fifth class includes all other *R *genes that confer resistance through different mechanisms, for example, *Hm1 *in maize and *mlo *in barley [3,17].

Based on conserved domains of known *R *genes, PCR amplification has been successfully used to isolate RGLs or RGAs from *Arabidopsis *[18,19], soybean [20-22], rice [23], maize [24,25], wheat [26], and many other plant species [27-30]. For example, Bertioli *et al. *isolated 78 RGAs from cultivated and wild peanuts by using degenerate primers designed on NBS conserved domains [31]. Leal-Bertioli *et al. *identified candidate genome regions for disease resistances by using a combination of NBS profiling, sequence characterized amplified region (SCAR) markers, and Southern blot by probes with NBS encoding regions [32]. In common bean, eight classes of disease resistance related sequences and 15 RGAs have been isolated based on the conserved NBS and TIR domains [33,34]. An RGA, SB1, was found to cosegregate with the *Crg *gene which is known to be responsible for resistance to the rust pathogen *Uromyces appendiculatus *[35].

Recently, data-mining approaches have been employed to identify RGLs and RGAs from plant EST databases. In maize, 199 RGLs have been identified using data-mining, modified amplified fragment length polymorphism (AFLP), and rapid amplification of cDNA ends (RACE) methods [25]. In sugarcane, Rossi *et al. *identified 88 *R*-gene-like ESTs via the data-mining method [36]. In wheat, Dilbirligi and Gill found 220 expressed *R*-gene candidates using modified RNA fingerprinting and data-mining approaches [37]. In *Medicago*, Zhu *et al. *have revealed 179 unique NBS-LRR RGLs by using combination methods of database queries, hybridization with RGLs from related species, and PCR [38]. With the availability of several complete and nearly complete plant genome sequences, *R *gene analyses at the whole genome level have been carried out in *Arabidopsis *[10], rice [39], *Medicago *[40], and *Lotus *[41]. As a result, 149 NBS-LRR-encoding genes and an additional 58 related genes have been identified in the *Arabidopsis *genome [10]. Also 489 predicted NBS-LRR genes were identified in the rice genome, all of which belong to the non-TIR-NBS-LRR class [39]. From the *Medicago truncatula *draft genome (Mt1.0), Ameline-Torregrosa *et al. *have identified 333 non-redundant NBS-LRRs and predicted 400 to 500 NBS-LRRs in the full genome [40]. In the *Lotus *genome, 158 NBS-encoding sequences have been isolated and nearly 40% of them are pseudogenes encoding incomplete protein sequences [41]. With advances in sequencing technologies over the past few years, next-generation sequencing technologies have become widely available and have dramatically accelerated biological research [42], making identification of *R *gene homologs and members of other related gene families by data-mining methods much more efficient.

Common bean (*Phaseolus vulgaris *L.) is one of the most important legume crops in the world. Due to its high protein and low fat content, it has become a principal source of dietary protein and fiber in many developing countries, especially those in Eastern and Southern Africa and Latin America [43]. Diseases such as anthracnose, angular leaf spot (ALS), rust, common bacterial blight (CBB), and Bean golden yellow mosaic virus (BGYMV) highly reduce bean production and quality. For example, CBB is a serious seed-borne disease that can cause over 40% yield loss in both temperate and tropical bean production regions [44]. Rust can cause 18% to 100% yield loss of susceptible bean genotypes under favorable environmental conditions [45]. Therefore, it is very important to better understand disease resistance in common bean.

The objective of this study was to identify expressed RGLs from common bean and to develop RGL-tagged molecular markers for genetic mapping of candidate genes and QTLs for disease resistance.

## Results

### Identification of RGLs from 454 sequences and ESTs in GenBank

More than 1.69 million sequences with 349,977,079 base pairs (SRA028837) were generated by 454 sequencing from an adapter-ligated normalized cDNA collection from four different plant organs (leaves, flowers, roots, and pods) of common bean. After clustering and assembling using the Newbler software from Roche (Branford, CT), these sequences were assembled into 39,572 contigs and 19,723 singletons with sequence size more than 100 base pairs [46]. Moreover, 116,716 common bean ESTs are available in GenBank (verified on July 7, 2011). Therefore, a total of 176,011 sequences were used for identification of RGLs.

Full length protein sequences of the 50 known *R *genes (Table 1) were used to perform tBLASTn searches against 454-derived sequences and common bean ESTs in GenBank. A total of 259 contigs and nine singletons from 454-derived sequences and 629 common bean ESTs were identified with scores more than or equal to 100 and *E *values less than or equal to 1e-10. After clustering, 365 non-redundant sequences were developed and considered as tentative *P. vulgaris *RGLs, named as PvRGLs (See Additional file 1: sequences of 365 tentative PvRGLs). The average size of PvRGLs was about 1,110 base pairs, although some fragments were 2,500 base pairs or longer. Among these 365 tentative PvRGLs, 166 (45.48%) PvRGLs were from 454-derived sequences, 147 (40.27%) were from common bean ESTs, and 52 (14.25%) PvRGLs were present in both of the groups (See Additional file 2: Characterization of 365 tentative PvRGLs).

**Table 1:** Fifty known *R *genes from plants used in this study [2]

Plant	*R *genes	Protein ID	Structure	Plant	*R *genes	Protein ID	Structure
Apple	*Vf1*	CAC40825	LRR	Potato	*R1*	AAU95638	NBS-LRR
		
	*Fls2*	BAB11088	LRR-PK		*Rgc1*	AAF76163	NBS-LRR
			
	*Pbs1*	ABR46085	PK		*Rx*	CAB50786	NBS-LRR
	
Arabidopsis	*Rpm1*	CAA61131	NBS-LRR	Rice	*Pib*	BAA76282	NBS-LRR
			
	*Rpp1*	AAC72977	NBS-LRR		*Pi-ta*	BAF91352	NBS-LRR
			
	*Rpp4*	AAM18462	NBS-LRR		*Rpr1*	BAA75812	NBS-LRR
			
	*Rpp5*	AAF08790	NBS-LRR		*Xa1*	BAA25068	NBS-LRR
			
	*Rpp8*	AAC78631	NBS-LRR		*Xa21*	AAC80225	LRR-PK
			
	*Rpp13*	AAF42832	NBS-LRR		*Xa26*	ABD36512	LRR-PK
			
	*Rps2*	AAM90883	NBS-LRR	Sugarbeet	*Hs1*	AAW03319	LRR-TM
	
	*Rps4*	CAB50708	NBS-LRR	Tobacco	*N*	AAA50763	NBS-LRR
	
	*Rps5*	AAC26126	NBS-LRR		*Cf-2*	AAC15779	LRR-TM
			
	*Rpw8*	ACJ05900	TM		*Cf-4*	CAA05268	LRR-TM
			
	*Ssi4*	AAN86124	NBS-LRR		*Cf-5*	AAC78591	LRR-TM
			
	*Rcy1*	AAM13905	NBS-LRR		*Cf-9*	CAA05274	LRR-TM
		
Barley	*Rpg1*	ABK51312	PK	Tomato	*Hero*	CAD29728	NBS-LRR
			
	*Mla1*	AAG37354	NBS-LRR		*I2*	AAD27815	NBS-LRR
			
	*Mla6*	CAC29242	NBS-LRR		*Mi-1*	AAC67238	NBS-LRR
			
	*Mlo*	CAB06083	TM		*Prf*	AAC49408	NBS-LRR
		
Flax	*L6*	AAA91022	NBS-LRR		*Pto*	AAB47421	PK
			
	*M*	AAB47618	NBS-LRR		*Sw-5*	ACQ42910	NBS-LRR
	
	*P2*	AAK28806	NBS-LRR		*Cre3*	CAD12795	NBS-LRR
		
Maize	*Hm1*	AAC04333	Toxin reductase	Wheat	*VRGL 1*	AAF19148	NBS-LRR
			
	*Rp1-d*	AAD47197	NBS-LRR		*Yr10*	AAG42168	NBS-LRR

Lettuce	*Dm3*	AAD03156	NBS-LRR	Pepper	*Bs2*	AAF09256	NBS-LRR

In addition, all tentative PvRGLs were used to identify their putative functions by BLASTX searches against the GenBank databases. Among the 365 tentative PvRGLs, 29 belonged to the NBS-LRR class, 96 belonged to the LRR, LRR-TM, and LRR-PK classes, 229 belonged to the PK class, six were similar to *R *gene reated sequences with putative TM domains, and five showed high similarity to *R *genes with Toxin reductase domains (See Additional file 2: Characterization of 365 tentative PvRGLs).

### Validation of expression of selected RGLs

**Figure 1:**
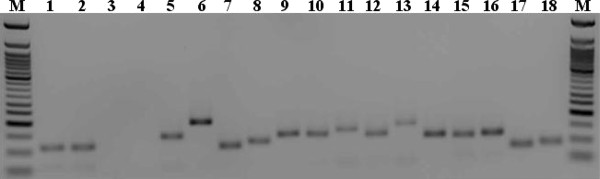
**Validation of fifteen selected tentative PvRGLs by RT-PCR**. Confirmation of absence of DNA contamination is shown in lanes 1-4 where RT-PCR amplification was carried out with primers designed from contig11286 in lanes with genomic DNA, leaf cDNA, leaf cDNA control (no reverse transcriptase added to reverse transcription reaction), and water as template to check DNA contamination. In lanes 5-18, RT-PCR products derived by amplification from an additional 14 common bean RGLs using leaf cDNA as a template are shown. M: 50 bp ladder.

In order to determine whether the identified PvRGLs from common bean were expressed, cDNA was prepared from two-week old leaves of the genotype Sierra grown in the greenhouse. RT-PCR was then used to amplify with primers specific to each of the 27 selected PvRGLs. Twenty four of the 27 primer pairs produced a single product and we showed amplification of 15 of these primer pairs (Figure 1). Furthermore, to ensure that the amplification products actually amplified from expressed mRNA and not from contaminating DNA, we used genomic DNA, cDNA, cDNA control (no reverse transcriptase added to reverse transcription reaction), and water as templates to perform RT-PCR and the results showed that only DNA and cDNA have specific products, while cDNA control and water have no products (Figure 1). Based on these results, we concluded that there was no DNA contamination in the cDNA and our PvRGLs were indeed expressed.

### Mapping identified PvRGLs to the *P. vulgaris *genome

Mapping of the identified PvRGLs is important for isolation of candidates of a particular resistance gene or quantitative trait locus (QTL). Recently, a Finger_Printed Contigs (FPC) physical map has been established for common bean http://phaseolus.genomics.purdue.edu/ with about 9X coverage of the genome [47]. The common bean FPCs makes it possible to integrate some of the PvRGLs into the physical map by comparison to BAC-end sequences [45]. BLASTn search was used to determine those PvRGLs matching to a BAC-end sequence with the criteria of scores more than or equal to 100 and *E *values less than or equal to 1e-50. As a result, 105 (28.77%) of the 365 tentative PvRGLs matched to BAC-end sequences with minimum criteria and could be integrated into the physical map (see Additional file 3: list of 105 tentative PvRGLs having matches to a BAC-end sequences).

Interestingly, there were five cases of two PvRGLs and three cases of three PvRGLs located to one physical contig: for example, PvRGL093 and PvRGL233 were located in the *P. vulgaris *physical map FPC_Contig13 while PvRGL266, PvRGL275 and PvRGL365 were located in the *P. vulgaris *physical map FPC_Contig137. Surprisingly, PvRGL083 and PvRGL236 have a match to different regions of the same BAC-end sequence PV_GBa0056B11.r (EI454956). Another seven PvRGLs matched to a Genome Survey Sequence (GSS) with *E *value less than 1e-50 from other common bean genotypes in GenBank.

### Mapping PvRGLs to *G. max *pseudomolecules and *P. vulgaris *genetic map

In order to take advantage of the synteny blocks between common bean and soybean as per McClean *et al. *[48], we used the BLASTn algorithm to search against soybean genome with the same criteria as McClean *et al. *[48]. Given that nearly every common bean locus can match to two soybean loci, the best two soybean hits were selected. Out of 365 PvRGLs, 360 (98.63%) PvRGLs could be mapped to the *G. max *genome pseudomolecules. Among the 360 PvRGLs, 343 (95.28%) have soybean hits with *E *value less than 1e-50. The 360 PvRGLs are broadly distributed among the 20 *G. max *pseudomolecules. Among the 713 soybean loci matched to 360 PvRGLs (7 PvRGLs with only one hit from soybean), 689 (96.63%) have transcripts. The annotation of these transcripts indicates most of them show high similarity to sequences containing NBS, LRR, PK, TM and toxin reductase domains (See Additional file 4: Mapping PvRGLs to *G. max *pseudomolecules and *P. vulgaris *genetic map). Based on the comparison of PvRGLs to the soybean genome sequences and the conserved blocks between common bean and soybean [48], a total of 237 (64.93%) PvRGLs can be located in the *P. vulgaris *genetic map. The PvRGLs are broadly distributed among the 11 *P. vulgaris *linkage groups, but the distribution was not even. *P. vulgaris *chromosome 8 (Pv8) contained the largest number of PvRGLs, 41; while Pv4 contained the least number of PvRGLs, 9 (See Additional file 4: Mapping PvRGLs to *G. max *pseudomolecules and *P. vulgaris *genetic map).

### RGL-tagged molecular markers

Ninety primer pairs were designed based on the 105 PvRGLs matching to BAC-end sequences. Eighty two of ninety primer pairs (91.11%) have specific products among the six genotypes. All of the PCR products were sequenced three times from both the 5' and 3' ends to ensure polymorphisms were not due to sequencing errors. The average fragment size was about 590 base pairs, while the range was from 309 to 1,549 base pairs. Approximately 291 kb of sequence data was collected from six genotypes for sequence analysis. Sequence alignments indicated single nucleotide polymorphisms (SNPs) and small insertions/deletions (InDels) occurred frequently. Twenty three SNPs were related to restriction enzyme sites, and 11 small InDels were identified.

Based on small InDels, 11 RGL-tagged STS markers gave rise to distinct and polymorphic PCR bands among six genotypes (Table 2). Likewise, 19 RGL-tagged CAPS markers have been obtained from those SNPs related to restriction sites (Table 3), and each CAPS marker showed unambiguous polymorphic bands following digestion with its corresponding restriction endonuclease.

**Table 2:** Primer sequences and sizes of InDels for the RGL-tagged STS markers

PvRGL	Forward primer	Reverse primer	InDel Sizes (bp)*
PvRGL011	GAGGTCATTCACATTATCGGTTT	TTCAAGTCACCACTGGCAAAG	14/G

PvRGL016	TGCACCATGACTGTTCTCCACC	AATACCACCTCCAGCCCAAGA	6/G

PvRGL041	GCCTCCGTTTGAAAGTTGCTC	CACGAAATATCTTACGTTTGTTTGC	7/G

PvRGL049	CACATTAGTAGCCTTTATGTCCC	CTTCGGTCCTGTCTACAAGGT	10/S, O, B, P, A

PvRGL084	TGTAATCATTGACAAGCGTGGAG	TTGCTTGAGCATTGATGTATTG	6/G

PvRGL153	AGTGATTTCCATTTATACGAGG	AAAAGCCAAGTAACATTCTTCT	8/S, O, B, P, A

PvRGL155	GAACCAGCAATTTGTTACCGAG	TAGTTGTTGAGCAACCTTTAGC	6/S, O, G, B, P

PvRGL189	AAGGGCAGATAACACCTCATTC	GATAGTGGTTAAATTTCACCAAGG	13/S, G, B, P, A

PvRGL193	CAGCTATAATAACTTTGGTGGGAG	CATTTAACAATGATGCAGGGAC	4/S, O, G, P

PvRGL272	CATCAGTCACGTTGAAGGAAGG	TATGTCCCAACAATTCTTCCCG	3/G

PvRGL297	TTGGAGCAATGAGAAAATAGGGG	TCCAGCAGAAGCCCTGTGATG	3/P

*The genotypes shown in this column have insertions with the corresponding InDel size

**Table 3:** Primer sequences and restriction enzymes for the RGL-tagged CAPS markers

PvRGL	Forward primer	Reverse primer	Restriction enzymes*
PvRGL016	TGCACCATGACTGTTCTCCACC	AATACCACCTCCAGCCCAAGA	HindIII/G

PvRGL039	CTACTCCCTCAAAGGAATCACC	TGAGAAAGTTTTTGCTCAATGG	DraI/B, A

PvRGL040	TCTTAAAGGACGCACCTACACG	ATTGGCCTTCAAGACGGTTAT	SnaBI/G

PvRGL041	GCCTCCGTTTGAAAGTTGCTC	CACGAAATATCTTACGTTTGTTTGC	AgeI/G

PvRGL049	GAACAATGTTAGTAGTACTTCGCC	GGCTTCCTCTTGAGAGTACTTTG	PflMI/S, O, G, B, P

PvRGL050	CACATTAGTAGCCTTTATGTCCC	CTTCGGTCCTGTCTACAAGGT	HindIII/G

PvRGL065	CGCTTAGGAGTTGGTAGAGTAACAC	TCAAGGAAACACCGACAGAATG	NsiI/S, O, B, P, A

PvRGL073	CCAAAATCAGAAACTTTGGCATG	AGAGCCCTGGGCAGGAAAGAC	AflII/G

PvRGL093	TGGGTTACTGTTGTGACGATGT	TATGTGCCCATGATCCTGGTAG	DraI/O, P

PvRGL099	CTTCGCCATCTCCTTGTCTATC	GCCCTATGCTCAGCCTTTCTAG	BglII/B, G

PvRGL102	TGAAGCCCTTATGCACAGGTCC	TCATCATCGCCAAACCCCAAC	XmnI/S, O, P

PvRGL153	AGTGATTTCCATTTATACGAGG	AAAAGCCAAGTAACATTCTTCT	SphI/O

PvRGL155	GAACCAGCAATTTGTTACCGAG	TAGTTGTTGAGCAACCTTTAGC	NdeI/O, B, P

PvRGL173	GGATCATTGCCTGTATTCGAGG	TGGCACTGTCCCTGATAAACTG	EcoRI/G, B, A

PvRGL246	CAGCCACCTTGGGTTGGTAATC	GACGCAGTTGCTAAATGGACACC	HindIII/S, O, P

PvRGL264	GTAACTCGTGTCCTCCTCATCT	GATACTTGAGAAGGTAAAGGCTG	SpeI/B

PvRGL297	TTGGAGCAATGAGAAAATAGGGG	TCCAGCAGAAGCCCTGTGATG	SpeI/G

PvRGL302	CTCTTAAAGGACGCACCTACAC	TATTGGCCTTCAAGACGGTTAT	SnaBI/G

PvRGL308	TAAGATGTGTCAATAAATATGCTG	CAGGAAGTTGCTGTAAAGAAGT	BspMI/G

			

*The genotypes shown in this column can be digested by the corresponding restriction enzyme

## Discussion

As a component of the gene-for-gene resistance mechanism in plants, resistance genes play an important role in recognizing products encoded by specific avirulence genes of a pathogen [49]. In this study, 365 tentative RGLs from common bean were successfully identified by data-mining based on the availability of 454-derived sequences in our lab and common bean ESTs in GenBank. About 60% (218) of identified PvRGLs were from 454-derived sequences; moreover, 166 (76.15%) of the 218 PvRGLs were new transcripts.

ESTs are highly valuable for genome annotation and gene structure prediction [50]. The 454 sequencing is a faster and more cost-effective method of producing sequence data than the Sanger method and is capable of producing a 400 to 600 million base pairs per run with 400 to 500 base pair read length [51]. It has been successfully used to maximize gene discovery, improve gene predictions, and detect SNPs and mutations [52,53]. In the past few years, data-mining approaches have been successfully used to isolate RGLs or RGAs from sugarcane [36], wheat [37] and maize [25]. As reported previously, Dilbirligi and Gill [37] adopted four different data-mining methods including domain search, individual and multiple motif searches, consensus sequence search, and individual full-length search to mine *R*-gene-like wheat sequences, and showed that the individual full-length search was the most successful method. There were 243 NBS-LRRs in addition to 101 other types of expressed *R*-gene candidates which were then isolated via an individual full-length search using a low *E *value of e-1 [37]. Xiao *et al. *[25] used three methods including modified AFLP, RACE and data-mining to isolate RGAs and *R*-gene-like ESTs from maize and found that the data-mining method is the most efficient way. A total of 186 expressed RGAs were recovered from 550,000 maize ESTs using a moderate *E *value of e-10 or better [25]. Rossi *et al. *revealed 88 RGLs from sugarcane ESTs by using a very stringent *E *value of e-50 or better and represented three major classes of *R *genes, namely NBS-LRR, LRR-TM and PK [36]. The above three research reports showed that different *E *values have a great effect on the number of resulting RGA or RGLs. In the present study, the moderate *E *value of e-10, similar to that in maize, was used to mine common bean RGLs and a total of 365 tentative PvRGLs were identified. Of the 365 tentative PvRGLs, 29 belonged to NBS-LRR type, 96 belonged to LRR, LRR-TM, and LRR-PK type, 229 belonged to PK type, six and five contained sequences with similar to putative TM domains and Toxin reductase domains, respectively. The number of RGLs identified in the present study was about two times more than those in maize due to three reasons. Firstly, 1.77 million 454-derived sequences and common bean ESTs were screened to identify RGLs in common bean, about three times more EST sequences than those in maize (550,000 ESTs). Secondly, 454 sequencing can generate more sequence data than the Sanger sequencing. Therefore, transcripts at extremely low levels can be detected [51]. Finally, some identified PvRGLs match the same *R *genes or RGLs. For example, PvRGL083 and PvRGL236 match different regions of the same BAC-end sequence.

RT-PCR was used to examine the expression of the PvRGLs in this study. Results indicated that all of the selected PvRGLs were actually expressed in the leaves of genotype Sierra (Figure 1). In contrast, many RGLs or RGAs amplified from genomic DNA using degenerate primers or mined from whole genome are not expressed [33]. Previously, eight classes of disease-resistance related sequences were amplified from common bean DNA using degenerate primers based on the conserved NBS domain [33]. Expression analysis indicated that three RGAs (SB1, SB3 and SB8) were not expressed [33]. In *Lotus*, 62 NBS-encoding sequences were considered as pseudogenes due to encoding of incomplete protein sequences [41]. In *Arabidopsis*, at least 12 NBS-LRR-encoding genes were predicted to be pseudogenes due to frame shift and nonsense mutations [10].

The PvRGLs discovered in this study correspond to most of the 25 previous common bean *R *genes or RGAs in the PRGdb database [2]. PvRGL266 and PvRGL275 have strong hits to the *P. vulgaris *TL5601 disease resistance protein gene with amino acid identity of 90% and 85%, respectively. TL5601, located in the *I *locus of common bean, controls resistance to Bean Common Mosaic Virus [54]. PvRGL262 matched to coiled-coil NBS-LRR (CNL)-B11 with amino acid identity of 94%. CNL-B11 was mapped in the B4 *R *gene cluster which contained at least three *R *genes (*Co-9*, *Co-y*, and *Co-z*) and QTL effective against anthracnose, and Bean golden yellow mosaic virus [55,56]. PvRGL309 was a part of polygalacturonase-inhibiting protein (PGIP) gene. PGIP can inhibit fungal endopolygalacturonases and is considered to be an important factor for plant resistance to phytopathogenic fungi [57]. PvRGL294 was the same as, but much longer in the 5' end than the previous RGL SB3 and OB9 [33].

So far, most of the known *R *genes have been cloned by map-based cloning and transposon tagging approaches [58]. Therefore, mapping RGLs and RGAs to genomes and/or genetic maps is very important and will facilitate *R *gene cloning. In this study, 105 PvRGLs could be integrated into the common bean FPC physical map by comparison of PvRGLs to *P. vulgaris *BAC-end sequences. Additionally, we were able to anchor 237 PvRGLs to the common bean genetic map by using conserved syntenic blocks between common bean and soybean. The PvRGLs are broadly, but unevenly distributed among the 11 linkage groups of common bean with a strong tendency of clustering. For example, 41 PvRGLs, the largest number, were mapped to Pv8, while 17 of them clustered at the bottom of Pv8. David *et al. *(2008) also found that many specific *R *genes against various pathogens cluster together: for example, in the B4 resistance gene cluster, 73 BAC clones (FI159954 - FI160067) were identified by using a NBS probe PRLJ1 to screen a common bean BAC library [56]. In the present study, PvRGL173 was anchored at the top of Pv4 by *in silico *mapping. Meanwhile, PvRGL173 shows high similarity (92.56% and 96.79%) with BAC-end sequence FI160023 (5e-153) and FI159996 (1e-101), respectively. PvRGL173 should be located in the B4 resistance gene cluster. The B4 resistance gene cluster contains various *R *genes resistant to different pathogens, such as *Colletotrichum lindemuthianum *(anthracnose), *Uromyces appendiculatus *(rust), and *Pseudomonas syringae *pv. *phaseolicola *(halo blight), in addition to QTLs for resistance to BGYMV and anthracnose; therefore further work will be needed to determine the role of PvRGL173. In *Medicago*, two superclusters of disease resistance genes were identified. One is located at the top of Mt3 containing 73 CNL and 9 TNL encoding sequences. The other is located at the bottom of Mt6 containing 57 TNL encoding sequences [38]. Clustering of *R *genes facilitates the genetic variation of *R *genes and benefits in the evolution of new *R *genes. Bertioli *et al. *found that retrotransposons were associated with the evolution of some resistance gene clusters via analysis of the synteny among *Arachis*, *Lotus*, and *Medicago *[59]. Several other hypotheses such as duplication, gene conversion, and unequal crossing-over have been proposed to elucidate *R *gene cluster and evolution [1,38]. Therefore, further studies will be needed to better understand common bean *R *gene evolution.

## Conclusions

In this study, 365 PvRGLs were identified from 454-derived sequences and common bean ESTs in GenBank using data-mining. As a result, 105 and 237 PvRGLs were mapped to the *P. vulgaris *FPC physical map and genetic map, respectively. RGL-tagged markers were developed for 25 unique PvRGLs, including 11 STS and 19 CAPS markers. These methods and results will help develop more RGL-tagged molecular markers that can be used further for genetic mapping and the isolation of resistance genes from common bean in the future.

## Methods

### Plant material

Plant genotypes used in this study included *P. vulgaris *cv. Sierra (S), Olathe (O), G19833 (G), Bat93 (B), Pinto114 (P), and Aurora (A). All of the genotypes were planted in a greenhouse. Leaves of each genotype were harvested from two-week old seedlings for DNA extraction. Additionally, two-week old leaves from Sierra were collected for RNA extraction.

### Data mining of common bean RGLs

The amino-acid sequences of 50 known *R *genes (Table 1), covering the five major *R *gene classes, were used to search for (tBLASTn) homologues in 454-derived sequences and common bean ESTs in GenBank http://www.ncbi.nlm.nih.gov. Those sequences with scores more than or equal to 100 and *E *values less than or equal to 1e-10 were clustered to develop unigenes, and all of the unigenes were considered as putative PvRGLs [25]. The resulting unigenes were in turn used to search the GenBank databases by BLASTX to confirm their putative *R *gene-like functions. In addition, all tentative PvRGLs were used to identify possible genomic sequences in the *Phaseolus *genome database http://phaseolus.genomics.purdue.edu/.

### Mapping of PvRGLs to *G. max *pseudomolecules and *P. vulgaris *linkage groups

The 20 pseudomolecules of the soybean genome were downloaded from ftp://ftp.jgi-psf.org/pub/JGI_data/phytozome/v7.0/Gmax/[60]. BLASTn algorithm was used to search soybean genome as per McClean *et al. *[48]. Firstly, we selected the hits with a cutoff *E *value less than 1e-10 and overlaps of at least 150 bp. Secondly, the best two soybean hits for PvRGLs were retrieved for further analysis (See Additional file 4 for all of PvRGLs that matched to soybean with these criteria). The PvRGLs were mapped to common bean linkage groups based on the syntenic blocks between common bean and soybean according to McClean *et al. *[48].

### RNA extraction and expression analysis

Total RNA was isolated from two-week old leaves with Trizol (Invitrogen, Carlsbad, CA) according to the manufacturer's instructions. Total RNA was treated with DNaseΙ two times to remove all DNA contamination. cDNA synthesis was carried out from 1 μg of RNA using the MMLV reverse transcriptase (New England Biolabs, Ipswich, MA). The RT-PCR primers were designed and synthesized at Integrated DNA Technologies (IDT, Coralville, IA). The RT-PCR was performed with the BIO-RAD PCR system (Hercules, CA) with the following cycling parameters: 94°C for 3 min; 35 cycles of 94°C for 30 s, 58°C for 30 s, 72°C for 60 s, and a final elongation at 72°C for 5 min.

### PCR amplification and sequence analysis

DNA was isolated from the above six genotypes according to the methods described in Doyle and Doyle [61]. PCR amplification was carried out by designing forward and reverse primers from PvRGLs having a hit to BAC-end sequences. The PCR products were purified using QIAquick^® ^Gel Extraction kit (Germantown, MD) and sequenced at the Macrogen USA Corp (Rockville, MD). The software DNAMAN version 4.0 was used for sequence assembly and multiple sequence alignment to confirm either identities or similarities.

### Development of RGL-tagged molecular markers

PvRGL sequences amplified from the above six genotypes were carefully analyzed by the DNAMAN program to identify InDels and SNPs, and those with InDels and SNPs were taken as candidate RGL markers. For STS markers, two 200-bp sequences flanking the InDel site were extracted and used to design PCR primers. Polymorphic PCR bands from the six genotypes were observed on 6% polyacrylamide gels. For CAPS markers, polymorphic PCR bands were observed among the six genotypes following digestion with a particular restriction endonuclease.

## Authors' contributions

ZL participated in conceiving the research, sequence analysis, developing molecular markers, PvRGL mapping, and writing the manuscript. MC participated in material preparation, developing molecular markers, and contributed to writing of the manuscript. AT participated in PvRGL sequencing and contributed to the writing of the manuscript. VK participated in conceiving the research, providing the 454 sequences, and contributed to writing of the manuscript. All authors read and approved the final manuscript.

## Supplementary Material

Additional file 1 **Sequences of 365 tentative PvRGLs**. Click here for file

Additional file 2 **Characterization of 365 tentative PvRGLs**. Click here for file

Additional file 3 **105 PvRGLs having a hit with a BAC-end sequence**. Click here for file

Additional file 4 **Mapping PvRGLs to *G. max *pseudomolecules and *P. vulgaris *genetic map**. Click here for file
