# Supplementary material for: Identification of expressed resistance gene-like sequences by data mining in 454-derived transcriptomic sequences of common bean (Phaseolus vulgaris L.)
Source: BMC Plant Biol. 2012 Mar 23;12:42. doi: 10.1186/1471-2229-12-42 (PMC3353201; doi:10.1186/1471-2229-12-42)
Supplement: Additional file 1 — Sequences of 365 tentative PvRGLs. [file 1471-2229-12-42-S1.DOC]

>PvRGL001

GTCAGCTCGATTGGAAAGGGTGGTTTTGGAAACGTCTACAAGGGAGTCCTCAAGAATGGCATGACAGTGGCTGTGAAAAGAAGCCAGCCAGGGTCAGGTCAAGGCCTTCCAGAATTTCAGACAGAGATTATGGTTTTGTCCAAGATTCGCCACAGGCACCTGGTTTCCTTAATTGGGTATTGTGATGAGAGGTTCGAGATGATTCTGGTCTATGAATACATGGAAAAAGGAACCCTAAGGGACCATTTGTATAACACAAAGTTTCCAACCTTGTCCTGGAAGGTGAGGCTTCAAATTTGCATTGATTCTGCCAGGGGTCTTCATTACCTTCATAAAGGAGCAGCTGGGGGAATCATTCACCGTGATGTAAAGTCCACAAACATATTGCTTGATGAGAACCATGTTGCTAAAGTTGCTGATTTTGGTCTTTCAAGGTCAGGTCCTCTTGATACTCAACCGTATGTCAGCACTGGTGTTAAAGGCACTTTTGGGTATCTTGATCCTGAGTATTTCAGGTCTCAACAGCTGACAGAAAAATCTGATGTGTATTCATTTGGAGTAGTTCTTTTGGAAGTGTTGTGTGCAAGAGCAGTGATTGACCCATCTCTTCCAAGAGATCAGATAAACTTGGCGGAATGGGGATTGCTTTGCAAAAACAAGGGGACACTCCAAGAGATCATCGATCCTTCCATCAAGGATCAAATTGATCAAAACTCACTCAGAAAATTTAGTGAGACGATAGAAAAATGCTTGCAAGAAGATGGTTCTGATAGGCCAACCATGGGTGATGTGTTGTGGGACTTGGAGTATGCGGTGCAGCTTCAGAGAGGTGCAAATGCAATACAGAGAGAGCCGTATGAGGATAGTTCTAGCAATGTTTCTGCATCATTTCAACTGCCCAATGTTCGGCGCCTTCCTTCACTCTCCACACTGAGTGAAGCCGATGATACCATTGTGAGGCATGATGAATCCGATAGTGCTGTGGATTACGTTTTCTCCCAGTTGAAGATTGATGATGCCAGATAGATTATTGAGCACTTCAAGGTAGTCATCTATTCGCAAATGTCTTTAGTAGAGTAGCTTGCTTTTAAGATAGATTTTTCAACCTTTCGTCTTTTTGTTTCTGTAGTTTTTGTTGT

>PvRGL002

GANAAAGANAGNANTCCCTTCTGCTCGTGGACTGGCTTATTTGCATGAAGATTCAAGTCCTCATGTCATACATAGAGACTTCAAGTCTAGTAATATCTTGCTGGAAGAAGATTTTACACCAAAAGTATCCGATTTTGGATTGGCCCGAACAGCAGCTGATGAGGAGAACAGACACGTATCAACACGTGTAATGGGAACTTTCGGTTATGTGGCTCCAGAGTATGCAATGACCGGGCATCTTCTTGTGAAGAGTGATGTTTACAGCTATGGTGTTGTCCTCCTTGAGCTCTTGACTGGAAGAAAACCAGTAGACATGTCACGATCTCCCGGTCAAGAGAATCTCGTTGCGTGGGCTCGTCCATTGCTCTCAAGCGAAGAAGGAGTGAAAGCAATAATAGATCCATCTCTGGGACCTGATGTGCCTTCTGATAGTGTGGCTAAAGTTGCAGCCATTGCTTCAATGTGTGTACAACCAGAGGTTTCTGACCGTCCCTTTATGGGTGAGGTTGTTCAGGCTTTAAAACTAGTGTGCAATGAATGTGATGAGGCAAGAGAAGCAGGCTCAAGTTCTACCTCTGTTGATTTGAGTCATTCTAGACAAGTATCAGATAATTTCCAAGGCCAATCCTCAGCCACCAATTACGATTCTGGAGTTGATATTGAAAATGGACTGTTGGCATCAGAGTTATTCAGCTCATCATCCGCTAGATATGGAAAGCGAGTGTCAGGGTCATTTAGAAGACATTCTTATTCAGGTCCTCTGAATACTGGAAGAAGCAAACGATTATGGCAGATAATTAGAAAGTTTTCTGGTGGTAGTGTCAGTGAACATGGAACTATGTTCAAGTTATGACCAAATTGCAATTTTTGAACTTGCTAGGTGCATACTTCTCTCTCTCACTTTGTTAATGTCCACTTCAGAACTCTGCTAAAGGCATTGCTCTTCTCTGCCTGAGCCCTTTGAAGTTTGACAACAGAATACAAGCAACTTGTAAATAGGTGTTGACATGTTTCTCTGCTAACCTGCAAAACCATGTGTATGTAACTATACTAACTCTTTGAAAAGAGGAAGCAATGCAAGTCTAGCTTGAAAATAGAAGATGACTGAAGATGGCCTTTACTAAGACCCATTGGCAATACAACATACAAGGTATAGTCTTTGCAGTAGCAGCATCAGTAATGTAATGTTGAAATTGTCAAAAACACACATGTATTTAGAAGAGGAGATGAATGGAATCTGGTTAGTTAGTTCTGTAGAACATGATATATCACAAATACATTACGGAATATCAGAATATGATGATAGTTTCTCTTTTAAAATAGATTTTAAGTCTAATTTAACATCGAAACATAGTTACATATGCAAGGACCTTTGTTTTCTGCATTTCAATAATTACCTATTGCACCATTCAAATTTCAATATATTGATTCTCTTTTTGTTTA

>PvRGL003

CAGAGATGGAAATTTTGGGGAAAATAAGGCATAGAAATATACTTAAACTCTATGCTTCTTTACTTAAAGGAGGATCCAACCTTTTAGTGTTTGAGTACATGCCAAATGGTAATCTTTTTCAAGCTCTCCATGGACAGATAAAAGATGGGAAGCCACACTTGGATTGGAAACAGAGATATAAAATTGCTCTGGGATCTGCCAAAGGAATTGCCTATTTACACCATGACTGTAATCCACCCGTTATTCATAGGGACATAAAATCCAGCAACATTTTGCTTGACGAGGATTATGAGCCAAAAATTGCTGATTTTGGCATTGCAAGGTTTGCAGAAAAATCTGATAAGCAGTTGGGTTATAGCTGTCTAGCTGGCACACTTGGTTATATTGCTCCAGAACTTGCTTATGCCACTGACATCACTGAAAAGAGTGATGTTTATAGCTTTGGAGTGGTGCTGTTAGAATTAGTGTCAGGCAGAGAACCCATTGAAGAGGATTATGGAGAGGCGAAGGATATTGTTTATTGGGTTTTGACTCATCTGAATGACCGTGAAAGTATTCTCAACATTCTTGATGAAAGGGTGGCATCTGAGTGTGTTGAAGATATGATAAAAATGTTGAAGATTGGCATCAAGTGCACCACTAAGCTTCCATCTGTGCGTCCCACCATGAGAGAGGTTGTTAAGATGCTAATTGATGCAGAACCTTGTGCACTGAAATCACCAAAATTCCGCCATGACAAAGACACAAAAGCCCTTCTCTGATGGCTTTCTTTTCAAATCTAATGTAATTGTAATGTGAATCCACCCAACTTCCACTCAATTATATTTCCCAACTTTGTACCAATTTCGTAACCTAGTCGACATACATTTATATGATAATTATGCCTTCCTAAAGTGATCTTA

>PvRGL004

ATTTCACTTTTCAATATCGAAAAGCCTATCTCTCTCGTTCTGTCTCTAAGTTCCCAACGTTTTTCCCGTCCTACAGCTCGATCCTCAGGATAGAGAAAGAAGGATGATCTACACAGCAATTGACACGTTCTACCTCACCGACGAGCAGGTAGCAAACTCACCTTCTAGAAAAGATGGCATAGATGAAGCCACTGAGACCACCCTTAGAATCTATGGCTGTGATCTTGTCCAAGAAAGTGGCATTTTGCTCAGATTGCCTCAAGCAGTCATGGCTACTGGGCAGGTTCTATTTCAACGTTTCTATTGCAAGAAGTCGTTTGCACGGTTCAATGTCAAGAAAGTGGCTGCAAGCTGTGTATGGTTGGCTTCAAAACTGGAGGAAAACCCTAGAAAAGCCAGACAAGTAATTATTGTTTTTCACAGGATGGAGTGCAGGAGGGAGAACTTTCCCATGGAGCATTTAGACTTGTATTCCAAGAAATATGTTGATTTGAAAATGGAATTGAGCAGAACAGAAAGACATATTTTGAAAGAAATGGGGTTCATTTGTCATGTTGAACATCCTCATAAGTTTATCTCAAATTACCTTGCTACCCTTGAAACACCTCCAGAATTGAGGCAAGAAGCTTGGAATCTGGCTAATGATAGTTTGAGAACTACATTGTGTGTTCGATTTAAGAGTGAGGTGGTGGCTTGTGGAGTTGTATATGCTGCTGCTCGCAGGTTCCAAGTACCCCTTCCTGAGAACCCACCATGGTGGAAGGCATTTGATGGGGAGAAGTCTGGGATTGATGAAGT

>PvRGL005

CAGACAAGGGACTNTCTTGTTCAAACCCTTTCCTAACCCACCTCACCAAATCAGGGACTTCCATGGAAGTTGATGCTGCCAGAGAAGAATCTGGGGACTTTCCAGTAAGCAATTCAAGTAATACAACTCCAAATGAATATACATCCCATTTCTGGGTGGGTCTGCAACCGAGAACTCGAGCCTCTGGAGCTTTGTAGTTGTTAGTTCGCTCTGTTTGTGATGGCTTCAAGTAAGGAAGAGCTCCTCCCATAAAGCCACCAGAGGATGGGTTGTTGCCTGTGATGCTGATGAGTCTGTTAAGACCAAAATCAGAAATGTGGGGTTGAAAGTCCGTGTCAAGGAGGATGTTGGAGGGTTTGATGTCACCATGAACAAATTTTCTAGGACTGCATTCATGGAGATAGGCCAAGCCCCTGGCAGTTCCTTTGATGATCCTTAGCCTGGTTGACCATGAAAAATTCGGTGGTGGTTGACCATTTCTTCCTCTAAGGGCAGTGGCCAAATTGCCATTGGAGATGAAATCACTGATCAAAAGCTTCTCATCAGGAGCCCAATAATAAGCTCTCAACTTCACAATGTTGGGATGCTTCACTTTGCCAATGGCTTGAACCTCAGCTGCAAACTCCTTAAACCTCTGTTCCCCACCTTCCCCCAATCTCCTCACAGCCACAGGCACACCATTCCCCAGGACCACCTTATACACAATCCCTAACCCACTCTGTTCCCAACACTTCCCGACAGCAACGGCCCTCAACAACTCATCAAGCTCAAAGTTAAAAC

>PvRGL006

GGCAAAGGTGGTTGTAGTTATGTTTACAGAGGATGTCTTCCAGATGGCAAGGAATTGGCGGTGAAAATTTTGAAGCCATCTGAAAACGTGATAAAGGAGTTTGTTCAGGAAATTGAGATCATTACAACTTTGCGCCACAAGAACATAATATCTATATCTGGCTTTTGCTTAGAGGGCGACCATTTGCTACTAGTTTACGATTTTTTGTCAAGGGGAAGCCTAGAAGAAAACCTTCACGGCAACAAGGCGGACTGTAGTGCATTTGGTTGGCAAGAAAGGTACAAGGTGGCTGTGGGTGTAGCTGAGGCACTGGATTATCTGCATAATGGCTGTGCTAAGGCTGTTATCCACAGGGATGTGAAATCTTCTAATATCCTTCTTTCAAGNTGATTTTGAGCCTAAGCTTTCAGATTTTGGACTGGCTAGTTGGGGTTCATCTTCTTCCCATGTTACTTGTACCGATGTAGCTGGAACTTTTGGATACCTCGCACCTGAGTATTTTATGCACGGTAGAGTGACTGATAAAATTGATGTATATGCTTTTGGTGTTGTACTCCTTGAGCTTCTATCAAATAGGAAGCCCATTAACAATGAATGTCCAAAGGGACAGGAGAGCCTTGTTATGTGGGCAACACCAATCCTTAAAGGTGGGAAGGTTACCCAGTTGCTTGATCCAAACCTAGGCATTGAATACGATGATTGCCAGATGAAGAGAATGGTTTTGGCCGCTAGTCTCTGCATTAGACGGGTACCTAGACTGCGGCCTCAAATCAGCCTTATATTGAAACTTCTGCGTGGTGATGAAGAGGTGACAAGATGGGCAGAACAAGAAGCTAATGCACCACAGGAACTTGATGGTTTTGACGAGGAACCAGTTCCAACTAACATACAGTCCCACCTGAACCTTGCTTTGATGGACCTGGAGGATGATGCGGTTTCTATCAGTAGCACTGAGCAGAGTGTATCTTTGGAGGACTATTTGCAGGGAAGATGGAGTCGCTGTTCAAGTTTTGACTGACAAGTTCATTTCGATACCATATTGGAATCAAATGTTTGTTTCATATATTATGTTCGTCTATCTTAGGTTTAGATTTTAAGTTCTGCGGTATCACAGATTGGGTCTTGGAAGAGAGGTCCAACAGCAAAAAGTTCTGTCCCCCCTCCTTGACTTTGTAGCTACGTACGTCTCAATCTGTATTCCGAAAAGCTTTTAGCAGTAGAACACATCTTGGCGAGCTTTTGGGAGCAAATTCTAACGGGCTAATTCTGTAAACTTCTACAGAAAAGATGTAAATTACATATTTCACAATGAGAGATCGTTGTCTTGCTTCATAGTGGCCTGGTGCCACTGCTTGTTTCTTTTATCTTTTTTTTTTAACTGTAATAACTATTTCCATGTTTTCTTCTTGTAGTGTGAAATTGTAAATAAACTGAGCATTAAAATTTGAGAGTATAAGGCCACTTTCAC

>PvRGL007

CAACGATGCATAATAATCTTTTATTGGGTGAATAGAACAAAATGGCTTGAATTTACATGAGCAAAACCTCTAGCTACAATCATCCCTTGGAATGGGAGAAATCTGGATGAGTGTGTAATGCGAGTATAAAAGCTGAGGTATAGTAAGGATCAAACAATGTAGAGCAATGTATCAAGTTATGCTCCATGGATGCACAAATTGTTTGGCCTTCGTCAATGACAGGTTGTAAAGGGAGACACAAGTTTTAGGGTCATTAAAGAGACAGCTATGCAAATTTATTGCGTTTCTATTTGAGGGTTCGTTCTTGTCTCATTGACACACATCCCGTTTTCAACTTTGCCATCATCAACTGATACAGCTGCCTCCTCTTTCAACCCATCCCCTACTCTATCATTTGGTGAATGTCCTGCTTCCCTTCTAGCTCTGCGATCCTCTTTGTAAGGGGAATCTTCAGCTTCAAGCATGTGTATCACATGCCCCATTTTAGGTCGCTTCTGTGCATTAGGATCAGTGCAACGTAAGGCTACAAGAAGGGCTCGTTTTAGTGCCCTTGAAGTTGGTTTCTCAGGAAGCTTGGGATCCAGGACCCCCTCTGGATTTCTGTTACTAACCATCTTCTTGAGCCAGTCAACTAAATTCACCTCTTCTGGAGGCCTACTATAATCAACAGGGTTCCTTCCTGTAAGAACTTCCATAATAAGAATTCCAAAACTATAAACATCACTTCGTTCATTCAACATGCCAGTACTTGCATATTCAGGAGCAACATATCTGCGAATAGAAGAATTAGTAAAATTGCTGGTCATGAACAATACATAGAGGACGAAATGGAGCAACGGAAAAGGAAGAGTATACAAACCCAAAAGTACCCATGACACGGGTGGTAATATAACTGTTGTCAGAGCCAAGAAGTTTGGCGAGGCCAAAGTCTGAGACCTTTGCATTCCACTGCTTACTGAGCAAAATGTTGCTTGATTTAATATCACGGTGAACAACTTTTGGTTCCAACCCCTCGTGTAGATAAGTTAGCCCTTTAGCCGTTCCCAGAATAATGTTCATTCGAATTTCCCATGTTAGAGGACTGTAAGGCCCAACATCCCCATGTAGCCACTGCTCCAAGTTACCATTGTCGACAAACTCATAAACAAGCATTCTATGAGCTCCTTCAGCACAATAACCAAGCAACCTCACTAAATTCTTGTGGCGAACTCGTCCTATAGCTTCCACTTCCACCTTAAACTCCTTCTCAGCTTGCCCCCTGTTATTGAGCAAATTCTTAATGGCAACATGAGTATTGTCGTTCAAGATACCGTGATAAACAATTCCATAACCTCCTTCGCCAATAACATTCTCAGGGGCAAAGGCGTTGGTGGAATCCTCAAGCTCCCGGAGAGTGTACCAGTGGCCCCAGCCCAAGTGAGAAACTTCGGGAATGACAGTGGGAACCTGATCGAGGAGGCGAGCCTCAGCACTGGAAGGGTCGTTGGAAGATGAGCGAAGCAAGGCACGTTCGGGGTAGGAAATTCGGTGGTTCTTCCCAATCTCGAACTGGATTCTGTGGTAGCTGAGGGGGTTGGTTTCTTCCTCGGTAGGCGGAACTGGGTCAGGTTCTGGAAAAGGGTCGGGTTGGTGGGTCGGGTTGGGGATGGGGGCGTGGTCGACCCTGATTTCTTGGATCTCCTTGGAGACATCGGGGATGGAAACGGGGTTGGCTTTGGAGCGCTTGAAGGCCACCCAGACGGATATGAGGAAGAGGATGAGGACGATGGCGGCGCCGACACACACGCCAAGCACCACCCAGAGACGGAGGCCAAAGATGGAGGTGGGGTTGGAGAGCTGGTCGTTCATCGCGCCGCTGCTCGACTGATCGGGCATTGCTGTTCATTCATTCATTCATTCCAGC

>PvRGL008

CCAAGGATTCCTCAAATTCTTGAGCAATTTTAAAAAATACAAACAGTTTTTGTTGAGCTGATAGCCTAAGTTGAGGGAACTGAATGAATAGAAAAGCAAATCAAAGACACAACCATGATCTTCTTATCCATTAGGTTTTGGTGACTTGATATAATGATGATATGGAGAACCATGAGGACCTTTTGGACTTGACAAGGACCTTGCAGGTTGACCCTTTTTTGATATTGTTATCTGTGCTTGCATACCATTTTTAGAAGTTGGCATGGACATGGTTCGATCTACTCGGGCAACCTGGAAATGATAAGTTGAGATAGCCATATCCTTAAGGTTTTGCAAATGCTTCAGAGCTTGAACAACATCACTCATCATAGGTCTGGATTTTGGATCACGGCAAAGGCATTGAGCAGCCAACTGGGCAGCCTTCTGTGACCCTTTAACAGAGAAGTGACCTTCAAGCCGAGGATCAATAATCCGTAGTATCATCCTCCGGTCCCCAAGTACAGGTCTTGCCCACTCCACAAGATTGTGTTCCCCATTTGGTCTATTCTTATCGATGGATCGCCGGCCAGTCAGCATTTCTAGTAGCACCACTCCAAAACTATAGACATCACTTTTTGATGTCAAATGCCCAGTCATCACATACTCGGGAGCTGCATAACCATATGTTCCCATGACTCTCGTTGATATGTGTGTCTTTTCCCCTTCAGGACCATCTTTGGCTAGTCCAAAATCAGAGAGCTTTGCATTGTATTCCGCATCTAACAGAATATTAGATGTCTTAAAATCACGATAGATTACAGGCCGCTGAGCTTCTTCATGGAGAAAAGAAAGACCTTTTGCAGCACCAAGTGCAATTTTCATTCTGATAGACCAAGGAAGAGGCAACGACCCTTTTCTGAAGAGGTGGTTCTCCAAACTTCCACGGGGCATACATTCATACACCAACAATCTTTGGTCGTCTTCAATACAGAAACCAACCAATTTGACCAGATTCGGATGGACGAGGTCACCAAGAATGTCTAACTCTGCAAGCCACTCTTTGTGACCCTGAAGCCCATCATGGTTGAGAGTTTTCACTGCTACAGTAAGCCCAGTACCAGGTTTCACAGGTGCAGTACCATTTTCTTCAATCCAACCCTTGAAAACACATCCAAATCCTCCCTCACCAAGAAGACTCTCTGGTCTGAAATTCCTAGTGGCCAACTTAAGCTCATTGAATGTAAATTTTCTCAAACGAGAAGAAACCTTCAACTCCTCACTGAATTTTGGGGTGGAAGGGATACTTTCTGCATTACTGGTTGTTGTAGAGGACCCTGGAAGAGCATTGGTTTCCTTCTTGTTTTTCTCACAAGCAGAAGTTTTTTCCACACTGTTAGCACTAGTGCCACTGATGGAAGTATCAACCTTTGATCTTGAAGGTATACAGCTCCCAATGAAACAGAATTTAACACAGCACCCAATTTCCTTTTCTGCACCAGCCTTTTGCATCTTTACCTTTGACTTACCCACATCCAAACTCCCTGCCTGAATTGCATTATTCCCAAGCCCCATCTTCCACAAACTCTTACAAAGAGCTTTTCATGCTCAAAGTCCCCACAAGTTTCAGCCTTTAGGAAAATAAAAGA

>PvRGL009

GAAGGAGTAAAGAATAATTTTATTAATTGAAAATTTCGTACAATCTCAACTGAAGAAGGTGTATTTAAGCCAATAGTTGAAGAAAGCAAGAAATGACTTGGTTTTGTTTGAAATGGGCAAACGAACCAGAACCAAAAATACCCAAAATTGACTTTCAATATTGAGGAGAACATGTGGATGGTGGTAAATAAGAAGAGATGGTTAAAATAGTGAGTTTCCCACAAGTTCTTGATCCTCCAAAAAAACAAAACTCACTATTTACTTTTTTCTGACCAATTTAGACCGGGATGAGGAGGATTCAGCAATGTCCTGAGAAGAGTTTGAAGTTTCTGAATGTTGCAATGCTTGCTGCAAGGCTTCCACTATCTGACTCATGGATGGTCTATCTTCTGGATTCTTCTTCAAGCAACTATCCGCTAACTTGGCTATTTTGCGAGCTCCTTGGGGAGAATACTGGTTCCTGAGACGTGGATCCATTATCACCCCGAACCTGCTGGTGTCAGCAGGGTACTGTTTGACCCAATCTAGAAGCTTTTGTTCTGCTGAGGGACGGTTTCTTTCCAGTGATCTTCTTCCTGTGAGGATCTCATACAGCACTACACCAAAACTCCACATGTCGCTCTGAACTTTCAGATGACCCGTTTCAATGTACTCTGGTGCAGCATACCCTTGTGTTCCAACCACCGCAGTGGATACATGAGTCTGATCACCTTGTGGTCCTTCTCTAGCAAGACCAAAATCTGAGAGCTTTGGATGGAAATCCGCATCCAATAGAACATTTGAAGATTTGAAATCTCGGTAGATCACCTGAATTTCCAGTCCTTGATGGAGATAAGCTAATCCTTGAGCAGCACCAAGCATAATCTCCAATCTTGTTTTCCAAGGCATGCATGGTAAACTTTTATTGAATAGGTGATCCTCCAAGCTCCCGTTGGGCATGAACTCGTACACCAACAGTCGTTGGATTCCTCTTTCTCCATCCACGGAGCAGTACCCCAAAAGCTTAACCAAATTTGGGTGATTAACAATGCCAAGAAATTGAACTTCAGCAAGCCATTCTTTATGACCCTGGAAGCCACGCGTGTTGAGCCTTTTGATTGCTACTGGAAATGGATCACCCTGTCCATCGGAGGGTCTGATGGATCCTTTATACACACTCCCGAACCCCCCTTCTCCAATTTTGAGCGTCCTATTGAAACCTTGGGTAGCATCTCTGAGCTCTTGCAAAGTGAAAACTCTGAAGCTGTTCTCTTTCTCTCTGTACAAGTCCTTCACGCTCTTGGGCGAAGATAGGGAGCCGGTGGAGTTGGCGGCGCGGTTCACGGCGGGGTTTTTCTTCTTATGCAATTCGGGGGCGGATTTACACTTGTCTTTGAAGAAGAAACACTTCATCTTCTCACCATCAAACCAAGGAGAAGGGAAATGATCAGAAGAAGAAGAGGAAGAGATAATTAAGTTGATGACCCAGATGAGAAAATGGGACCTTGATTATATATGTGAATGGTGAATTATGATAAGGAACAAGAGATATGAAAAGGGAAAAAAGTTAGCTTCCGTGAAGTGAAAGAAATGTATTGAATGGGAACGATGAAGAAGAAGAAGGTATGAAAGTAATAAGAAAAACACATCCGCGTGGGGGAGGAGAGAGACGATGACAGGAGTAAAGCGGGTTAACGACGTTGTGTGTGTGTGGAAGAAAGACCA

>PvRGL010

CCGTTGCTGTCGGGGGTTGGTTCCACCGTGGGTGCTGCTGGTGATTGGGCTTTGAGGCTAAGAGGGTATAAGCTTGTGCAAGTTAGTCTCTTTCAGAAGCCTATTGTGAAGGTGAAGTTGGGGGATTTGATGGCTGCTACAAACAATTTCAGTGGGGAGAATGTTCTTTTTGCAACAAGAACCGGGACAACGTACAAGGCTGATCTCCCAGATGGTTCCACGCTTGCAGTGAAGAGGTTGAATGCTTGTAGGATTGGGGAGAAGCAGTTTGGGATGGAGATGAACCGGTTGGGGCAGGTTAGGCACCCTAATCTGGCTCCTCTGTTGGGGTACTGTGTGGTGGAGGAGGAGAAGTTGTTGGTTTACAAGCACATGTCCAATGGAACTTTGTATTCCTTGTTGCACAAGAATGGTGGGGTGCTGGATTGGATGATGAGGTTCAGGATTGGATTAGGCGCAGCAAGGGGGCTTGCATGGCTGCACCATGGCTGCCACCCTCCCATCATACAGCAGAACATTTGCTCCAATGTCATTCTTGTTGATGAGGAGTTTGATGCTCGGTTGATGGACTTTGGGCTAGCAAGGCTCATGGCTTCAGACTCAAATGGTAGTTTTGTGAATGGAGATTTGGGGGAGATTGGTTACATCGCCCCGGAGTATCCAAGCACTTTGGTTGCTTCATTGAAAGGGGATGTGTATGGTTTTGGTGTTTTGCTTTTGGAGTTGGTGACAGGCCAAAAACCACTTTATGTGAGCAATGGGGAGGAAGATTTCAAGGGTAGCTTGGTGGATTGGGTGAACATGCATTCTAGTTTGGGAAGGATGAAGGATTGCATTGATAAAGCCATGAGTGGAAGAGGGCATGATGAGGAGATTCTTCAGTTTCTGAAAATTGCTTTGAATTGTGTGGTTTCTCGCCCCAAGGATAGGTGGTCTATGTACCAGGTTTATCATTCCTTAAAGGGGTTAT

>PvRGL011

ACGAGATACTATCAGCTGCTAAATATCTACGTTTAAATGCAGCAGTGAGGGATACGAGCCACTTTTATCCGTGAATGGTTTGCACCAGTTACACATCCTCATATTCTTCTTAGCAGTCTTACATGTGCTTTACAGTGCTATCACAATGCTTCTTGGAAGACTAAAGATACGTGGATGGAAGGCATGGGAGGCAGAGACTTCAACTCATAATTATGAGTTCGCCACTGCTGCTTCAAGATTTAGGCTTACTCATGAAACATCGTTCGTGAGAGCCCATGCCGATTTATGGACAAGGATTCCCATTTTCTTCTATATTGGCTGCTTCTTTAGGCAGTTTTATAGGTCTGTAAATAAGACGGATTACCTGACTTTGCGGAACGGGTTTATCACTGTACACCTGGCTCCTGGAAGTAAATTTAATTTCCAAAAGTATATCAAAAGATCATTTGAAGATGACTTCAAGGTGGTTGTAGGAGTCAGTCCTATCCTCTGGGCATCCGTTGCTGTTTCCGTTGCTCCTAAATGTTACATGGATGGCGTGCTATGTTCTGGGCATCCTTAATTCCTGTTGTTATAATTTTGGCTGTTGGAACAAAACTTCAAGCCACATTGGCAAAGATGGCTATTGAAATAACCGAAAGACATGCAGTTGTCCAAGGGATTCCTCTTGTTCAAGGCTCAGACAAATATTTTTGGTTTGGTCGGCCTCAGTTAGTTCTTCATCTTATCCATTTTGCTTTGTTTCAGAACGCATTCCAAATAACATATTTCTTGTGGATATGGTATTCTTTTGGGCTGAGAAATTGTTTCCATGCTGACTACAAGCTTGCAATTGTGAAAGTAGCATTAGGGCTTGCGGCTCTATGCCTCTGCAGCTATATCACCCTTCCATTATATGCTCTTGTTACTCAGATGGGCTCAAGAATGAAAAAGTCAATATTTGATGAACAAACATCAAAGGCAATAAAGAAATGGCGAATGGCAGTGAAAAATAAGCAGGGAGTGAAACTTGGAAACTCCAAGGTGCGAGCCATGGATGGAAGCACAACTGATTCAACAGTACACTCTTCTGGCCCCACACTTCACCGTTTCAAAACTACTGGTCACTCAACTCGCACCATGTCAGCCTATGATGATGACCAAGATTACCATTCTGACATTGAGCTGTCTCCCATTTCACCAACAGCAAACTTGATAGTAAGAGTGGACCATGATGAGCACCAAGCAGAAGAAGCTCAACACCACCCAACACCCAACAATCAAGACCTATCACGTTTGTCAACCCTGGAAACAAGCATGAAATAGCTTCAGAAATTCAACCATTTTTGTTCTTTTACAGATAGTCAGTTTTGTACATTCTTGTTCTAATACACTTAAAAAGCCTTTGAAACTTTAACCTTGTTCAAAAATCATGCTTCAGCTTTGTAATTGTGTTTGAGCAAGGGCTTATAAGTAGAGGACAAAGGCTCGTTAGAGGACAAAATTGTATTACATATTGAAGGGTATCTACACATCCCATTAAAATGCATGCTTTTTGGCTAAGTACACCAAATATAAATATATATATACACACACATTC

>PvRGL012

GAACCAAAATTTGGTACATCCAATTTTGGCTTTGTAACAGAAAACACTTGAAAAAACAAAACAGTATTCTATAAGCTAAATCTAGAAACAAATTATCCAGACACTATAATCTCATTCTGTACACAAAATCCTGGAGCTGGAGCTTCTTTCTTCCCTAGAATTAGTGAACTGGCTATCACTTTCTATAGTATATGCTTGATAACCCGTTGGACTAGAGAGCTGACCAGAGCCACTACTGCTCACAGATCTCTCAATATCAAGCCTCCCAGCAACAAGAGGGATTGGTCGTTCAGGTATTGAGGGCACTGATTCATCAGACTCCATCATTTTAACAACCTGATCCATTGTTGGCCTAGCATACAACTGCGGGTGAGAGCAAAGTACAGCAATTAACACATACTTCTCAAGAACTTGTGTTGAACCAGGTTGTGGCATGCCATCTTCAATTACATCCAAAGCCTTTCCTGTTCTGACTAATGACCAAGCCCAATCAGTCAAAGCAGAAGGTTGACCATCATTGTTCATCTGAAGCGCCTTCCTCCCACTCAAAAGCTCAAGAAGCACAACCCCATAACTGAACACATCACTTCTCTCTGTCAACTGTCCATACAAAGCATATTCCGGAGCAACATACCCCATGGTTCCAGCCACCCTAGTGCTCATATGCGTCATCCCCTCGGGGTTAAACTTCGCCAGTCCAAAATCTGCAACCTTGGCTTCAAACTTATCATCCAAAAGTATATTACTTGCTTTAATATCCCTATGAATGATTGCAGGCTGAGCCC

>PvRGL013

CTCACACTCTTTTCCTTGGATAAAGGTTATAATTGGCATCTTGGCTGCTTTGACATTTACCTTGTTGTGCCTTTTGTTTTTTGTAACACTCCATAAGAGATATATGGAATTTGAAACTCTTGAGGACTGGGAGATGGATTGTCCTCATAGGTTCAGATATAAAGATCTTCACCTAGCAACAAAGGGGTTCATAGAGAGCCAAAAAATTGGAGTTGGAGGCTTTGGTTCTGTGTACAAAGGGGTGTTACCCAGCACAAGAACTGAGGTTGCTGTTAAAAGGATCCTGAGAAGTCCAGCCCAAGGAATGAGGGAATTTGCAGCTGAGATTGAAAGCTTAGGAAGATTGAGGCACAAGAACTTGGTGAACCTTCAAGGGTGGTGCAAGCACAAGAATGATCTCCTCCTAGTTTATGATTACATTCAGAATGGGAGCCTTGACTCTCTCCTATTCAACAAAAACTTTGTTTTGGATTGGGACCAGAGATTCAAAATTCTCAAAGGTGTAGTTGCTGGGCTGTTGTATCTTCATGAAGAATGGGAGCAAGTGGTGATCCATAGGGATGTGAAATCTAGCAATATTCTTATTGACGTAGACTTCAATGCTCGTTTGGGTGACTTTGGACTAGCAAGACTTTGTAGCCATGATCAAGTGTCACACACAACAAGTGTGGTTGGAACCATTGGATATATTGCACCAGAGTTAACTAGGACAGGAAAAGCATCTACAAGCTCTGATGTGTACGCATTTGGGGTCCTACTTCTTGAAGTGGTTGCTGGAACAAGGCCTGTTGGTTCATCAGGTCAGTTTTTCTTGGTGGATTGGGTTCTTGAAAATTGTCAACTGGGTCAGATTCTTGAAGTGGTTGATCCAAAGCTAGATTCTGTTTATGATGAAGAGGAAGTAGAGTTGGTTCTGAAATTGGGTCTACTATGCACTCAAAACAAAGCTGAGTATAGACCCTCTATCGAACAAGTTACTAGGTACCTAAACTTTGATGACCCTTTTCCTGATATCTCTGATTGGAGATACTATGATTCTCAAAGTAGCACAACAAGCTTAGGTTTTACAGAAGCCATGTCTTCATCCTCGTACAGTTTAGCCTCCATTGAAAGGAATCCAATGTCCAGTGGCAGATAGCAAAAGAATATTTGTTATTTAATTTCCTTTTACCATATTCCCTTTTAATTGTTTCTACCTACTACCCAATTTATGAGACTTAACTGCTACAAATTGGTGGA

>PvRGL014

GTTTTGGGAAGGTTTATAAGGCAACCCTTCCCAATGGACAAGTGATAGCAATAAAAAGAGCACAAAAAGAATCTATGCAAGGAAAGCTTGAGTTTAAAGCGGAGATTGAACTTCTATCAAGGGTTCACCACAAGAATCTTGTCAGCCTTTTGGGCTTCTGCTTTGACCAAGGAGAGCAAATGCTGGTTTATGAGTATCTTCAAAATGGAAGTTTAAAGGACGCTCTCACAGGGAAGTCGGGAATTAGGTTAGATTGGATAAGAAGGCTAAAAATTGCCCTTGGTACTGCCCGGGGTCTGGCTTATCTTCACGAACTTGTTAATCCTCCCATCATACATAGGGACATCAAATCAAACAATATTTTGCTGGATGACCGCCTAAATGCCAAAGTTTCTGATTTTGGTCTCTCCAAGTCTATGATGGATTCAGAAAAAGATCATGTTACCACTCAAGTTAAAGGAACACTGGGTTACTTGGATCCAGAGTATTATATGTCTCAACAATTGACTGAGAAGAGTGACGTGTATAGCTTTGGTGTGCTAATGATGGAGCTGATATCTGCAAGAAAGCCATTGGAACGAGGGAAGTATATTGTGAAAGAGATTAGGAATGCACTGGATAAGACAAAAGGCTTATACGGTCTTCATGAATTTATTGATCCAGCCATTGGATTGTCGTCCACAACACTGATCGGTTTTGACAAGTTAGTGAATTTGGTCTTGAGATGTGTTGAAGAGTCAGGTGAAGAAAGGCCTAAAATGAGTGAAGTGGTAAGAGAAATTGAGAACATATTGAAGTCAGCTGGTACAAACCCTACTGAAGAATCACCCTCCATCTCTTCTAGCTATGAAGAGGTAAGCCGAGGGAGTTCCAGCCATCCTTATAACAGCAACGACACCTTTGATTTGAGTGCAGGGTCTCCATATCCAAAAGTTGACCCTGTGTAAGTAGTAGTCAATAATTAAGTACCTCAAATCCAAGCCAAAATTGTTTATATATCAAGTATATTAGTTTGCTAAATGTTGATCCAGTGTGTGCATAGCTGCATGTTAGCTTGAGAATGGACTAGTTAAGTCGGCTGCGAGTCAGTTGACGAGATGCCTGCTTCTTTTGTGAGGACTCTTCATTTTTAGCAGCATTATTTTTCTTCTCCAAAGACTTAGAAGAGAAAGGAAAAAGTGGCAGAAGCGCAAAGAGGTGAACCAATGTTTATGCAATTTTCTGATAATGATAAAGAGGTGAGCCAAGGAAGAAATATAATTCTGTGTTTTTAGTTATCCTATAGAATTTCGCTTATTGTGAAGCACTGGTGTGTACGTTTTGCACAAAATGATCAAAGTAAAAATAAGAACTATAGTGAATTATATTTTATTAGTGTGGTTTAATGTTTTTGTTGTTCTTTTATAACACTTG

>PvRGL015

GCAATTGTCATCACAATCACGGCAGGGAAATCGTGAATTTATAAATGAAATAGGCTTGATATCTTGTGTGCAACACCCCAACCTCGTAAAGCTTCATGGATATTGTGCTGAAGGGGAACAACTTTTGTTGGTATATGAGTACATGGAAAATAACAGCCTTGCCCGAGCTTTATTTGGTAGTGAGAACCGTCAGCTTAAATTGGATTGGCCCACAAGGTTTAGGATTTGCATTGGCATAGCAAAGGGTCTAGCATTTCTCCATGATGAGTCAAGATTCAAGATAGTACACAGAGATATCAAGGCTTCTAATGTGTTACTCGATGGCGATTTGAATCCAAAAATATCTGACTTTGGCTTGGCTAGGCTTGATGAAGCAGAGAAGACCCATATCAGCACCCGAGTTGCTGGAACCATAGGGTATATGGCACCAGAATATGCATTATGGGGCCATCTTACATACAAAGCCGATGTTTACAGTTTTGGAGTTCTAGCATTAGAAATTGTTAGTGGGAAAAACAATAACAATTATTTACCAGACGATGGAAGTACTTGTCTTTTGGATTGGGCATGTCAATTAAATCAAGCTAAGAAATTAGTGGGGCTTGTTGATGAAAGGTTGGGACCAGACCTTAATAAAACAGAAGTAGAAAAGGTGGTCAGAATAGCTCTTCTGTGCACAAATGTTTCTCTATCACTTAGGCCTACAATGTCTGAGGTGGTGAATATGCTTGAAGGACATTTAGATATTCCTGATGCAATCCCAGAACCAAGTACCTACAGTGAAGATTTAAGGTTCAAAGCCTTAAGGGATCTCAATGAACATCGATCGAAGCAGAGTTTGAGTGTAAACCAGAGCCAGAATTCATCAACTCATACATTCAGCTCAGCATCTGCTAGTAATACTCACACTAGTAGTAATATTGAGGATTATTGTTCAGTTGACAATAGTTAGCTACCCCTGTCAACAACTCGGATACACACTTACCCGTTAGCACTGGTTGCTCCATATCTTTAGGCCTTGAAATTTTTTATCATCAACCAGCCAGAGTTATCTTTACATATGCATATAACCTTCCATTTCCACTTAAGCAGTTTTCTGCTTTCTGCAGAGGTCTTTCCAAAGGAGTACGAGAATATTTTTCCTTTGTGTCACCTTTATAATACTTTATTTTTGTTTCTATTTAGTAGTGTACAAACATATTGCTACAAATAAAGCCATCACTGAATCATAAAATTTGGTTGCATCTTTATTCCTTACACGATGAGTTAAAAGCTTTCTATGA

>PvRGL016

CTCAACCTGTCCTCTAATCATTTTACAGGGAGAGTTCCAAATGAGTTTGAAAATTCTGTGTATTCTGAGAGCTTTTTGAATAATTCTGGCCTCTGTGCTGATACCCCAGTACTGAACCTAAGGCTGTGCAACGTTGGCTTTGAAAAGACAACCAAAGGCTCTTCTTGGTCTCTTGCTTTGATTCTGTGTTTGGTGGCAGTAGCCCTTTTGTTGGCTTTGTTGATATCACTCTTGATTATCAAACTTTTCAGAAGAAGAAAACATGGTTTGGATAACTCATGGAAGCTCATTTCCTTTCAAAGGCTGAGTTTCACTGAATCAAACATAGTGTCATCAATGACAGAACACAATGTTATCGGAAGTGGTGGATTTGGCACAGTATACCGTATCCCGGTTGATGGTTTAGACCATATTGCTGTCAAGAAGATCTGCAGTAACAGAAAGTTAGACCGAAAGCTAGAGAGCTCATTTCGTGCAGAAGTGAAAATATTGAGCAACATTCGTCATAAAAACATTGTGAAGTTGTTGTGTTGCATCTCCAATGAGGACTCTATGCTCCTTGTGTATGAGTATTTGGAAAACCGTAGCCTAGATAGGTGGCTGCACAATAAGGGCAAGTCATCAACTGTTTCAGGTTCAATGGATCATGCTGTTCTTGATTGGCCAAAGAGATTGAAAATAGCAATTGGGGCTGGCACAAGGTTTGAGCTACATGCACCATGATTGCTTCTCCACCTATTGTTCATCGAGATGTGAAAACAAGCAACATTCTTTTGGATGATCAATTCAATGCAAAAGTTGCTGATTTTGGTCTGGCCAGGATTTTGATGAAACCAGGGGAACTTGCCACCATGTCAGCTGTGATTGGATCATTTGGCTACATGGCTCCAGAGTATATTCAAACAACTAGAGTCAGTGAAAAGATTGATGTCTTCAGCTTTGGGGTGATCCTATTGGAACTTACAACTGGTAAAGAAGCTAATTATGGTGATGAGCACTCATCTCTTGCAGAGTGGGCGTGGAGGCACCACCAATTAGGAAGCAACATAGAAGAGCTGCTAGACAGAGAAGTCATGGAATCCAGTAACTCAGGTGGAATGTGTAAGGTTTTCAAACTAGGTGTCATGTGCACTGCAACACTTCCTTCTACTAGACCATCCATGAAAGAGGTTCTACAAGTATTGCTTTCTTGTGAAGAATTTTCTAAGGGAGAGATTAATATTGGCCACTGTGATGATGATGTTCCCTTTCTTAGAAATTCAAAAAGAGAGCACAAGTTGGATATTGATAATGATACCTAGCTACATGTTAAGAATCCAAGTATGAGTTTAGGTAAACATCAGATAGAAGAAAACATCATAGAAAACTCATATAACTGTTGTGTTAATGTCTTTGGGCTGGAGGTGGTATTTCTTGATGGGTTGTCTTTTTGGTCTTTATGGTCATAAAGACCCAAAAAGAGAAACATTGAAATGCATCAAAAGTGTCATAAAGTAGAAGTACTTATTTTT

>PvRGL017

GGAAGGAAGAAGGGTAGTTCACCTCTTCCGTGGTCTGTGAGGTACAAAGTTGCAATCGGGATTGCTGAAGCTGTGGCTTATCTTCACAGTGGAACTGAAAGATGTGTTGTGCACAGAGACATTAAGCCCTCAAACATTCTGCTTTCTTCAAGGAAGACTCCCAAGCTATGTGATTTCGGATTAGCTACATGGACTTCTGCACCTTCAGTTCCTTTCCTTTGCAAAACCGTCAAAGGAACATTTGGATATTTGGCTCCTGAGTATTTTCAACATGGGAAAGTATCGGATAAGACTGATGTATATGCTTTTGGAGTGGTTTTACTGGAACTCATTACTGGCCGGAAGCCAATTGAGGCAAGAAGACCTTCAGGAGAAGAAAACTTGGTCTTATGGGCCAAACCTTTTCTACAGAAAGGGAAAGGTGCCATTGAAGAGTTGCTTGATCCTCAACTCAAGTGCAGTCTGAGATTCTCAAATCAAATGGGTAGAATGATTGAAGCAGCAGGTGCCTGTGTCACCAATGAAGAATCTCGGAGACCTGGCATACGTGAGATTATTGCAATACTGAAGGGTGAAGAAGAGCCTCTTTTGTCTAAAAGAAAGAAATCCAGTTTTCTTGGAAATGGTTGCGTGATTGATTGTTATTCTCAGTTAGAACAAACAAATAATGAGATGAAAAGTCACTTGGCTTTAGCAATGCTAGGAGTTGCAGAGTTTGAGGATGATGACTATCTCTATGGTCGATAAAACATGTTGACAACCCTTCAATAACCTTTTACTTTTTGCTATGATTGTGATTAAGAGTGCTAAAGTTTAGATGTGAGCGAGTTGAANATGTAAATAGAAATAGGAGCTTAGATGATGATTGTGTGTAGATACTTTCCTTTTCCTTTTACCTTTCCC

>PvRGL018

AATAAATTATAAGTTTCCTTAAAATTAAGCTGTACATAACATTATTAATTGGTACAAAATTGGGTATATACCAGCTGTTAACGGTGTGCCTGACAATGTTGATTTCACTGACTGAAAACTATGTGATCAACAAACATTGCGGATTTGCGATTTGCAGGTGACTGACTGTGAGGTCACTTAGTCACCAACTTCTTACTAGTCCAGGCGCCCAACTTTTCCAATCCAACCTTCCAATAGACTTGTTTCTTAAGCTGACCATGTCTTTGAATTCAAAAACCTTCTTCAGGTAATTGTTCCCTTGTTTAGCTGGTTCAGGTTCAGTTTCCTCTACAGATATCTTCTCTTCTTTTTCTTCTCGGGCGGCTACAGCAGCTTGAGGGAAGCATTCATCCCGAGTATCTGTCTCAATAATAATGCTTCCAAGAGAGTCAACCACCTCACTCATTTTTGGGCGAGACTTTGGCTGTTTCATGAGGCACTTGTTTGCTAGGATTGCAATTTTATGAGCTGATTTGATGCAGTATTGTCCCTTAAGTCGAGGGTCTACAATATGATGGAACTTCCTAGGATCAGAAACATAAGGTCTAACCCACTCTAAAAGCTTTTGTTCAGTTTTAGGTAGGTTTCTCTCTACAGCTCTCCTCCCAGTGATAAGCTCGTAAAGAACTACACCAAAGCTCCACACATCACTCTTAGCAGTTAGCTTACCAGTCTGAACATACTCTGGTGCAGCATAACCTATGGTGCCAACAACAGCTGTTGAAACATAGCCAGACCCTTCTGAAGGTCCTTGCCTAGCCAGTCCAAAATCAGAAAGCTTTGCATTGAAGTTCTCATCCAGCAGGATGTTGGACGCCTTAAAATCTCCGAAATATTAGCTGAAAATCCATTTCTTCGGAGGCGTGAAGATAAGCCAAACCCCGAGCCGCATCTTGCGCAATTCTCAACCTGGTCCCCCAAGGCAGCACCGTGGAAGGGACTCGCGCCAAGAGATGCTCCTCCAGGCTCTTATTCGGCATAAACTCGTAAACCAGAAGCCTCTGAATCCCTCTTTCATCGTCCTCAGCACAGTACCCTACCAACTCGCACCAGATTAGGATGCTTCACCACTCCCAACAAATTCACCTCGTTGATCCACTCCTTGTGGCCCTGCTGCCCGTTGCGGTTCAGCTGCTTGATCGCCACCTCCTTTTCCTCCACCACGCCGCGGTACACGGACCCGAACCCCCCCTCGCCTACGAGGAGGGCGCGGCTGAACCCGCGCGTGGCACACTTCAGCGCTGAGAAGGGGAAGAGGCGGAGGTCGTTGGCACGGCGCTGGACCAGGAAGTGATGGAAGTCCACCGTGTCGGAGAATTCCGTGGAGTCGGAGTCGAAGTTGGAGCGGCTGCGCGTGTCCATGAGACTGAGCGAACGCGCCCACGACACGCGCGAAGTGGAACAGCGCGAACTCGGTGCCGCTGCCACGCAATCGTCGTCGTCAACGTTGGAGAAGTAGAAGCACTTCATTTGCAGAAACCAATGAACAAACCCAAAATCATTCCAATTCGCTCTCTGCTTCCCGTTACAGCAACGGTGCCAGGCATATCCTATGAGACTTGAATCGTCTGTGTTGCGANAGCTAGTGTTCCTGCGCCCACTCATAATCTCTAGTAGCAATACACCAAAACTATAGACATCAGATTTAACTGAAAATAGACCTTCCATTGCATATTCTGGCGCCATATAGCCATATGTACCAACCACTCTGTTTGTGTTGGCTTCATTTTGGTTTCCTCCAAATATTCTGGCTAAGCCAAAGTCTGAAATTTTTGGATTCATGTTTTCATCTAATAAAATGTTGCTTGCTTTTAGATCCCGATGAATGATTCGTAGTCTTGAATCCTGATGCAGGTAAAGCAGCCCTCTTGCAATGCCTTCAATTATTTCAAAGCGCTTTGTCCAGTCTAGTTGTGTTTGCTTCACCGGATCAAATAAAAAACAGTCCAAGCTTTTGTTTGGCAAGTACTCATATACCAGAATTTTTTCTTCTCCCTGAATGGAACATCCCAATAGTCTAACCAGATTTCTATGTTGCAGTTTGGCTATTAGCATCATTTCATTCTTGAACTCCTCTAAGCCTTGACTAGACTTTCTTGAAAGCCTCTTAACAGCTACTTCCTCTCCTCCTGGAAGGTTCCCCC

>PvRGL019

AAGCATTGGCCCTTCTGCTGTGTATAGAGATTATCCTAATGCTATTTTGAATGGGCTGGAGATAATGAAAATGAACAATTCTGTGAGAAGTTTCAGCTCATCGACAGCGCCTCAGACTAGTTCTTCTGGTTCAGGTTCTAAAAAGGTTGGCTTGATAGTAGGTGTGAGTCTTGGGGCATTTTTTGCAGTGGTCATGGTTCTTGTTTTCTTTCTACTATGCCGGAAAAGAAGACGTTTGGAAAAAGAAAAACAAGCGCATTCAAAGACGTGGATTCCTTTATCAATCAATGATGGAACTTCTCACACCATGGGAAGTAAATATTCTAATGCCACAACAGGAAGTGCTGCTTCAAACTTCGGGTACCGCTTCCCTTTTGTGACAGTTCAGGAGGCTACAAATAATTTTGATGAGAGTTGGGTTATTGGCATTGGTGGTTTTGGTAAAGTATACAAGGGTGAGTTAAATGATGGTACTAAAGTTGCAGTTAAGAGGGGGAATCCTCGGTCGCAGCAGGGGCTTGCGGAATTCCGAACTGAAATTGAGATGCTGTCACAATTCCGCCACCGCCATCTAGTATCATTGATTGGATATTGTGATGAAAAGAACGAAATGATATTGATATATGAATATATGGAAAAGGGAACTCTCAAAAGTCATCTGTATGGTTCAGGTCTTCCAAGCTTAAGTTGGAAGGAGAGGTTTG

>PvRGL020

CCCAAATCCATCATCCTAGGATTTGTTTTACGCCATGAAGATTCCTTGTTCATTTTGCACTTGTTTCTCAGCATCGGTCAAAGAACATCACACTGAGCACGAAGAGCCAGGTGAAGATAATGATGGAAACTTTCGCATATTCACTTATAGAGAATTGAATTCCGCCACGCGGGGTTTTCATCCCTCGGAAAAGATTGGAGAAGGAGGCTTTGGCTCCGTCTACAAGGGGCAGCTTCGGGATGGAAGTTTGGTGGCTGTTAAAGTGCTTTTGATTGAGCTAGATTCCTTAAGTGGAGAGAAGGGGTTTGTGGCAGAATTGAATACACTGGCATATATCAAGCACCAAAATCTAGTTATTCTTCGAGGGTGCTGTGTTGAAGGAGCCCACAGATACATAGTCTACGATTATATGGAAAACAACAGCCTCCGTCAAACTTTCTTAGCTTCTGAGCAAAAAATAATGGCATTCAGCTGGGAGGCTCGGATGGGTGTATCAATAGGTGTAGCAAGAGCGCTTGCCTTTCTCCATGAGGAACTTCAACCCCATATTGTGCACAGAGATATCAAATCCAGCAATGTTCTTCTTGATGGAAATTTCACACCAAAAGTCTCAGATTTTGGCCTGGCCAAGTTACTAAGAGAAGAAAAATCCTACATCAGTACCCGAGTTGCAGGCACACTAGGTTATATTGCTCCGGACTATGCCAGTTCTGGCCACCTGACACGAAAATCAGATGTTTATAGTTTTGGAGTACTACTTCTAGAGATTGTCAGTGGCCAAAGAGCAGTAGATGAACATCAAGATGTGGATCGTTTCTTAGTTGAGAAGTCTTGGGCAGCGTACGAGGGTAATGATCTTTTGAGAATGGTGGATCCAATGCTGAAGATGAACTATCCAAAGGAAGAAGCTAAACGGTTCCTAATGGTGGGACTTTGTTGCGTGCAAGAAACGACCAGGACCAGGCCACGAATGTCAGAGGTTGTAAACATGTTGAGCAACAACACTGAGATGGGAGAGTTTCATATTTCACGACCAGGATTTGTTAATGATATGAGGAACCTCAAAATGAAGAGGCAGCTAAATTCGTTAG

>PvRGL021

GTAAACCCCAGGTTCCAGTCACCGCGTGTTAACCGGCCCGTGAATGTTATCACTCCCTTAGGGGAATGGGTACCGGGCCCCCTTTTTTTTATAAAAGATGAATTTCATAACCAAGCAGCTTGCAATCTATAATTAACCATCCAATCTTTAAAGTTATCCTCTAAGAGAATGACAAAATGAAGGTGGTAACTGTAACTTGTGGGGGAAGCTGACAACGTGATGCTAATTCTGCGTGCATAAATTCTGGTGTTGTTTTTTTTTTAACAGCAAGAAGTATTTGGTGTGTTGTGTGCTCACGGCTTAGATCAGGTGATGTGGAGGAGGGTCATCCTTCAGCAACACAACATCATCAGAAGTGCTGAGATATCCATTACCTCAGGTTGACTTCTGTTTTTTGCCATCACTCTGAATATCCTGAGAGTCTCCACCACTTCTTCCATAGAAGGTCTCATGTCTTTGGGACTCTGCAAGCACTGAAATGCTAACTCAGCCCACCACCACTTATCATTTTCCTTATCTTGAAATCTGATTCAAAACCAAGGTTTCGGTCCACAAGCTCTTGCAGTGTCTGATTGTGAATCTTGTTAATGGCCATGTTGGCCAAATTGATTTCATGTCTATGCCTTGTGATGTCAACAGCAGGCAGTGATGATATCAGCTCAACCAGCACCACTCCAAAGCTATACACATCACTTTGTTGGTAAGCTGGTAACACTCGTGGTACTCTGGATCCACATAACCTGGAGTCCCTTGTGGAGCTGTTGAAACATGGGTGGCTTGGTGTGGGAAAAGACGCGAGAGTCCAAAATCTGCCACTTTCACACAGAAGTTACTTTCAAGTAGAATGTTGTTGGTTTTAACATCTCTGTGGATGATACCTTTGAGATGAAGAAACTTCAGTGCACTTGCAGTCTCCACAGCTATATTCATTCTAATATGCCAAGGGAGTGTTCCAGGTTTAGATAGATGACCATGAAGATGATCTGCAACAGTTCCATTAGAAATGTACTCATACACAAGCAGAAGTTCACGACTGTGGCGAGAGGTGCATCCGAATAACYTCACAAGATTTGGATGATCTATGCGAGTGAGGATTTTAATTTCATTCATGAACTGTGCAACTATCCTGTAGTTGTTCTCATACAACCTCTTCACTGCAACAGAACGCCCATCTCGCAGTTTGCCTAGAGAACAAACATCAGCCTCGTGCCACCTTCTCCTAGTTCCTTAGTTGAGTCAAAGTAATTCGTGGCCACTTCTAGTTCTTCATAGGAGAAGAGGTGAACTCCAGGTATAATGCTTTGAGTGTAGCTTTGACTTCCCTTCTCAGTGTCCTTGAAGATGGATCCCAAGAGAGGCTCCCGGACGCGTGGGTCGACGATACCCCGGGAATTCCGGACCGGGTACCCAGCCCTGGTTTTTTGGTACAAAACTTTG

>PvRGL022

ACCGTCACAGCTCCTACAAATGTAAAATCTTATTCTATAGCTGACCTACAGATTGCTACTGGAAGCTTCAGTGTGGATCAACTTCTTGGTGAGGGGTCTTTTGGACGTGTGTAGGGGGGTCAATTTGATGATGGAAAGGTTCTTGCTGTCAAGAAGATAGATTCATCTGTCCTTCCCAATGATTTGTCTGACGATTTTCTGGAACTGGTTTCAAGCATATCTAATTTGCACCATCCCAATGTGACAGAGCTTGCAGGTTATTGTTCAGAGCATGGACAACACCTACTAGTCTATGAGTTTCATCAAAATGGATCACTGCATGACTTCCTTCACCTACCTGATGAGTATAGTAAACCATTGATATGGAATTCACGTGTGAAGATTGCTTTGGGGATTGCACGGGCTTTAGAGTACCTCCATGAAGTTTGTTCACCATCTGTTGTTCATAAGAATATAAAGTCAGCCAACATATTGCTTGATACAGATCTTAATCCTCATCTTTCCGACAGTGGATTGGCAAGCTATATTCCAAATGCAAATCAGGTATTGAACAATAATTCTGGATCTGGATATGAAGCTCCCGAAGTTGGCATGTCTGGCCAATATACTCTTAAGAGTGATGTCTTTAGCTTTGGAGTTATCATGTTGGAACTTCTTAGTGGACGGAAACCATTTGATAGCTCAAGGCCAAGGTCTGAGCAGGGTTTGGTTCGATGGGCAACACCTCAACTCCATGATATCGATGCTCTGGCTAAAATGGTTGATCCTGCATTAGAAGGACTATACCCTGTGAAGTCTCTTTCTCGATTTGCAGATGTTATTGCTCTTTGTGTTCAGCCGGAGCCGGAGTTCCGACCACCTATGTCAGAAGTGGTTCAAGCGCTGGTTCGATTAGTGCAACGAACTAACATGAGTAAGAGGACAACGTTTGGAACTGATCAAGGAGGATCCAACCGAGGGGGAGATAACCAAGAATCACAAGACATGTAAATGGGAAGAAAAGCCTTGTGTTGATAGAATGGCAGAAAAGAAAAATAATATTCTTCAGAGTTTCACAATTCCTTTTTAACACCACCTTCAGCCAGTTTTTCTTATAAGTTAGATGGATTGCACTTTTGATTATTGAATCTAGATACCTGTTGGTGTATGTTTCTGAGCTATTTATTTAATCGGTCTTATAAATTTC

>PvRGL023

CATTGTGATAGTAACATTGTTTGTCATTTCTGTTATGCTGTTTGTGGCACATAGATGTTTCAGGGAAAAAGAAGATTTGCCTGAGTCTCCTCAAGAGAATTCAGAAGATGACAATTTCTTGGAGGGTTTAACCGGCATGCCAATCCGTTACAGCTACAATGATCTAAAAACTGCAACAAGTAACTTCTCAGTGAAGCTAGGAGAAGGGGGTTTTGGATCAGTTTATAAAGGAGCTGTACCAGATGGTACTCAACTAGCTGTGAAGAAGTTGGAAGGAATTGGCCGGGTGAGAAGCAGTTTCGGATGGAAGTTGCCACTATAAGTAGCACTCATCATCTGAATTTGGTGAGGCTCATTGGGTTTTGCTCAGAAGGGCGCCACCGGCTTCTGGTTTATGAGTTCATGAAAAATGGATCTCTTGATAATTTTCTATTTCTGACAGAACAGCATTCAGGAAAATTGTTGAATTGGGATTACCGATACAACATTGCGCTGGGCACAGCAAGAGGCATCACATACCTTCACGAGGAGTGCCGTGACTGCATAGTCCATTGTGACATAAAACCTGAAAACATTCTCTTGGATGAGAATTATGTTTCCAAAGTCTCTGATTTTGGTCTGGCAAAGCTTATTAATCCTAAGGACCATAGGCATCGGACCTTAACAAGTGTGAGAGGAACCAGAGGATACTTAGCTCCCGAGTGGCTTGCAAATCTTCCAATAACATCCAAATCTGATGTTTACAGCTATGGTATGGTTTTGTTGGAGATTGTGAGTGGAAGGAGGAATTTTGATGTTTCAGAGGAAACAAACAGGAAGAAGTTCTCGATTTGGGCCTATGAAGAGTTTGAGAAAGGTAACATCAGTGAAATTTTGGACAAAAGACTAGCTCGGCAAGAAGTTGATATGGAGCAAGTAAGAAGGGCAATTCAGGCAAGCTTTTGGTGCATCCAGGAGCAGCCATCTCAGAGACCAACAATGAGCAGAGTGCTGCAAATGCTAGAAGGGGTAACTCAGTTTGAAAAGCCACCTGCCCCAAAATCAGTAGTGATGGAAGGAACTGTTAGTGGAACAAGCACATACCTGAGTAGCAATGCCAGTGCATTCTCCACGGTTGGAGTTTCACCCCCTGGACCCTCCTCTACATCATCATTTCAGATTAGTAATAATGTTTCAACCTTCAACTCAGAAAGGAACCCTGAGAAGCCAACCTCGACCCTTTTACAATCAGACACATGACAGAAAATTGCTTCCATTTTCATTTTTCACATTTCCCAATTGGTTCAAACTCTCATTCTCTTGGTTTGGGGGGACTTTATCCTGGAACAGCAGAAGTACAAGCTTTGCTGCTCAAACCGTGTATAATATGATATTAATTGTATGCTATATTTCATTTTTTTTCTTTTCTTTATCCTGATCATTGTATTGTATACTCCTTCAAAGCAGGATATTGCAAAGTTGCAGTAATTATTTGCATTCAATTCCTGAAAATGTGCTTGTGTCAGCATATTTCTGGGTCAACAGTGCAACCCAATATCTTTGGTGTGCCAATAAAAGTATTAAAATTTTATGTTCTCAGCA

>PvRGL024

AGGAATATTTAGACACACACTGAAATTCACCATTGCATGGCACAATAATTATATGGTGCACTGAGCCAACATTTTGCAGCTTTGAGAGTAACTAATTGTTTCTACAAAGATCATCTAGGACCAGACAATTCATCTGGTGCAATATTTGAAGTTGAGTCATAATTTTTTTATGTAGAGATTGAAGGGGTTGTAATCTTTTTGGATTATATCTTCTTTCTTCCTCCATTTATCCCACTTTTCTTTCAAACCTTCACCCTCTAGCATTCTCACCACCTCAGACATCTTTGGTCTATCCAAAGGGGAGCTTTGTGTGCATAGCAATGCTATTTGGATTAACTCCTCTAGTTCCTCCTCGTCATAATCTCCATGTAAATCTGCATCCACCAGTGTCTTCAACTTCCTCTCTTTCAGAAGTGATTTCACCCATTCTAGCAACATGACCTCATCACTAATGGCATGTCGTGCTAAATTAAAAGCCCTCTGTCCAGTTATTATCTCAAGAAGCATCACACCATATCCAAAAACATCAGTCTTCTCTGAAGACTTTCCAGTTGAGAGATACTCTGGTGCTATGTGGCCAAGTGTCCCACGTACAGCAGTAGTAACATGAGTATTTTTGTAATCCATAATCTTTGCTAAACCAAAATCTCCTACAACTGCTTCAAAGTCCTTATCCAATAATACATTAGCAGCTTTGATATCACGATGAATAATCTTTGGGTTACAATGATCATGCAAATAAGCAAGCCCCTTAGCTGCTCCTAGAGCAATACGCTTCCGAACTGGCCATTGAACTGGTGCTTGTGATTCTGCTCGCTCTCGTAAACATGACTCTACACTTCCATTAACCATGAAAGGAAACACAAGCAATCTTTCAGTAGTTGTCATACAAAAACCAATCAGCCGAAGCAAATTTCGATGCACAGCCATGCTGATAATTTCCACTTCTATTTGAAATTGTTTCTCCTCACAGTGGATGCGTTCTTGGTTAAGTCTTTTTACTGCTACAAGATCACCATTTGTTAAGCGTCCTTTATAAACCTTGCCAAATCCACCTCTGCCAATAATGTTTTTGTTATTAAAGGCATCTGTTGCAACTAGCAGTTCATGCAGCGAAAACCTTTTAAGTTGACCAAAGTTAACTTCAGGATCCTCGTCAGCTGCAACATCAAAGAAATAATCTGGTGGCTTCCTTCTTTTCCAGTAAACAAATGCAATTACTGGAGCTGCAAACAACAGTGCAGCACCCACGGCAACTCCTCCAGCAATGGCTGCAATAGATATGTAACTATTACCTGAAGGATTTTGTTTCTGTATAACGCGTGGTGATGTTGGAGTGGTTTGCTTCAA

>PvRGL025

AGTTGTATTGTTGGGTACAAATCACGTAAGATCTTGACAGCATTTGAGTGATGTTCCACTCTCTCAACCAGTAAATATCTTCCATTAGCTGAAGCATTCTCATATGCTAGAATATGGGCCATATGCAACATCTCTCACGTCGATCCATCCCAAAGTTATATTTTTAAATGTTGGTGAACCATTAACAAAATTTAGAACTGCAGCAGCACTGGTGTTAAGAACTGGTTGCAAGAGAGGTCCAACTAACCAATGCTGGGTTTATTGTAACCAAATCAATATTGTGCTCTTTTGCAAATTTCCATGCAGCCTCTTCGGCCAAAGTCTTTGAAAGGTTATACCATACCCCATTTTTCCTTACAGATATTCAGGGTCGGAATACCAAGTCTCGTCAACCACAACATCAGGGGTTTTAGGCCTGTAGTTGAACGAAACTGCAGCAATGGAAGAAGTTAAAACGACGCGTTTCAGCGTGGGCGAGTTCACACACGATTTCAGAACATTAGAGTCCCCTTCAAAGCTGGATCCAACAACTCAGTCTGCGGATCCTTCGGCACTCGTGAAAAAAGGGAGAAGCAGTGTGAAACACAGCGTGACAACCTTCAACGGCAGAGTCAAAGGAACCTTCTTCTAGAAGATTCGCCTTCACCAGATGCAACCTCTCCTTGGCACCATCAAGGTTAAGCAAGTGATCAACCTTTTTGGGATCACTTAGGTCGCGAACAGTGGCCTTAACAGTGTAACCGCGTTGGAGGAGAAACTTGACGAGCCAGGAAGCGATGAAACCGGAGGCGCCGGTAACACACACTACTTGTCCGGCGCCGGTGCTCATTTCTGGATTATCTTCTTCGAGCTGATCTCTGCAGTTGAAGCGCTTGATAATGCCTCTCACGGGATATCGACCTTGAAGTAAAGGGTCAACCATCAAGGGAAATTTTTTCCTATCTCTGAACAAGGGTCTTGCCCACGCAACTAGATTTTGTTCTTTGGCAGGTTTCATATGGTCAATAGCCTTCCGGCCAGTAATAAGCTCCAAAAGAACAACCCCAAAGCTGTAAATATCTGACTTAAATGTCAATTGACCCGTCATTGCATAATCTGGAGCACAATACCCATATGTGCCCTAACCCTGTTGAAACATGGGTCTTATCACCACTTGGGCCTACTTTTGCCAGGCCAAAATCTGACAACTTAGGATGAAATCCCTCTCCTAACAAAATGTTAGAGCATTTCAAGTCACGATATATGACAGGAGGCTTCATTTTATCATGCAGATACTCCAAACCCCTTGCTGCCCCAGCTGCTATTTTCATTCTTGTGTTCCAATCTAGTGGTTTGCTACCAGGCCGAAGAAATCAGCAGTGATTCTCCAAAGACCCCAATGGCATGAATTCATAAACTAATAGCTTCTGCTCTCCCTCAGCACAAAATCCAATCAACTTGACAAGATTAGGGTGGTCTGCCAAACTCAATGTCAACACTTCAACTGCGAATTCTCGTATCCCTTGAAGTCCATTAGGGTCAAGTTGCTTTATAGCTACAACCTGGTTTATTCTCTCCAAGTGTCCCTTGTAAACCTTGCCAAAGCCTCCTTCACCCAAAAAGCAATCTGCCGTAAAATTTCCCGTCGCAGCTTCAAGTTCATTAAAACTGAATGACTGCGCCCGATTGCCATTATCTTTTCCATCATGAGAACCCTCCTCTTTCAAATTTAAATCCTTCACGTCCAGAGCAAGCTGATCACCTTTAGAACCATCTTCTTTTTTCCCATTCACTTTGAAATCAACTTTGACGCTTTCTGCGGGTGTGAAATTGGAGGGTTTGTGGTCGAAGTTGTTACCATAGTCTTCGGTATCCGAATTGGTGCATGATTTCCCCGCGCAACAAAAGCAACCCATGTTCTTTCTCTGCCCAAGCTCCACCAAGAAAACAGAGCCCAGAACAGAGACC

>PvRGL026

GGGATTACTAAAAGATGGGAAACTAGCTGCTATAAAAGTTCTTTCAGAAGAATCAACTCAAGGGGTGAAAGAATTTATGACAGAGATTAATGTCATCTCAGAAATAGAGCATGAAAATTTGGTTAAGCTATATGGTTGTTGTGTGGAAGGGAAAAACCGAATATTAGTCTACAATTTTCTTGAGAACAATAGCCTTGCGCAAACCATTTTAGGTAAATGCTTTGTAATTTATGACAGAGATTAATGTCATCTCAGAAAATCTGAGAACATGAAATTCTATTGTGTCGTTAAGACTATCATGGTTGTTGTGTGGAAGGGAAAAACCGAATATTAGTCTTTACCAATGCTTTCTTGAGAACAAGTTATTTGCTCTTGCGCAAACCATTTTAGGTTCAGGTCACAGTAACAATATTTTTGATTGGAAAACAAGATCTAGGATTTGCATTGGGATTGCACGCGGGCTCTCCTATCTACATGAAGAAGTAAAACCACATATTGTTCATAGGGATATAAAAGCAAGCAATATTCTCCTTGACAAAGACCTTACACCTAAGATTTCAGATTTTGGTCTTGCAAAGCTTATTCCATCATACATGACTCATGTCAGCACTCGTGTGGCAGGAACAATAGGTTATTTGGCACCAGAGTATGCATTAAGGGGGCACCTGACACGTAAAGCAGATATTTACAGCTTTGGTGTACTCCTTGTGGAGATAGTGAGTGGAAGATGTCACACTAATACACGATTACCGATAGGAGAGCAGTTTCTCTTAGAAACGACATGGAAACTTTATCAGCAAAGGGAACTGGTAGGGCTTGTAGATGCATCCCTAGACGGGTATTTCGATGCCGAGGAGGCCTGTACATTTCTGAAAGTTGGACTTCTCTGCACTCAGGACACATCCAAACTCCGGCCACCAATGTCTTCTGTGGTGAAGATGCTCACTGGAGAAATGGACATTGATGAAAGTAAGATAACAAAACCAGGCTTGATTTCAGATTTCATGGACCTGAAAATCAGAGGAGACAAAAAAGATGTTGATAATGATACCAAGGGTTCATCTTCCTACAATGCATCCTCTGCTTCAGATAGCCAGGGTAACACCATGTCATATGCTGCAAGTGCAAGTACAACTGCAACCTTCTCTGTTAAATATGATCCAAGCTTGTAAACATTATTTGTCCATGGTTGCCACTTTGATTCCAAATTATTTCATTTTTCTGAGGTCTCCCTTTCCCTTGTAAATTTGCTGCGGGTGCCATAAATTTGTGTGTTAATATTTTCTTCTGTTCGTGACTAGGAAGTTTTGCCCAATGTACACTATTTTGTAAATATGAAGAACTTTGTTGTTCAGAAACCATGCAGGTATATAAGGGTTCAGAAACCATGCAGGTATATAAGGGTTCAGAAAATTACAAAATACTTGATTTCATGGTTTCATCCTCAAAAATATTTTGTTTCAATATAATCCTCAATATTAATATTCTGTACNTA

>PvRGL027

GAAATGCAAAACATTCTCTCTCCGATTCACTCATTCGGATCTTATTTCTTCTTAATTTCTATTATTATTTATTTACTTTAAACCAAATTCAGTTCTCTGTAGCTATGAGTTGTTTTTCCTGCTTTGTTTCTCGCCGGAAAGATGTCAGGAGGGTTGAAATTGACAATGGCGCTCGATCTGCTGCTACTGCTGCCTCCTCAGAGGGTGAAAGGAAGAGAGAATCCTCAGAGGCAAAGGGGAAGAGTGTTAGCAGTAGTAAAGGAAGTACTGCAGCGGCTAGTTTTGGTTTTCGCGAACTTGCTGCGGCAACGAGGGGTTTCAAGGAAGGAATTTAATCGGGGAAGGTGGTTTTGGAAGGGTTTACAAGGGTCGACTTTCAACGGGCGAGCTTGTTGCCGTAAAACAATTGAGTCATGATGGTCGGCAAGGTTTTCAGGAATTTGTTACGGAGGTTCTTATGTTGAGCCTGCTGCACCATTCTAACCTTGTGAAGTTAATTGGCTACTGCACTGATGGAGATCAAAGGCTTTTGGTTTATGAGTACATGCCCATGGGTTCACTGGAGGATCATCTTTTTGATCCTACCCAAGACAAAGAACCCCTAAGTTGGAGTTCTTTGTCTTGGGTAGGATGAAGATAATGCTGTTGGAGCTGCTCGTGGCCTCGAGTATCTCCATTGTAAAGCAGATCCACCTGTTATCTACAGAGACTTGAAATCTGCTAACATCTTGTTAGATAATGAATTCAACCCAAAACTGTCAGATTTTGGGCTTGCTAAACTTGGACCTGTTGGAGACAATACCCATGTTTCAACCAGAGTAATGGGAACATATGGCTACTGTGCGCCCGAGTATGCCATGAGTGGCAAATTAACTCTTAAATCTGATATATACAGCTTTGGTGTGGTTTTGTTGGAGTTGATCACTGGACGTAGAGCAATAGATGCCAGTAGAAAACCAGGAGAGCAAAACTTGGTTTCATGGTCTCGCCCATATTTTAGTGATCGGAGAAAGTTTGTACATATGGTAGACCCTCTTTTACAAGGCAACTTCCCCTTGCGCTGTTTGCATCAGGCAATTGCAATAACTGCTATGTGTCTCCAGGAGCAACCAAAATTCCGTCCACTTATTGGTGATATTGTTGTTGCACTGGAATACCTAGCTTCTCAGAGTATTGCTGAAGTGCATAGACATGGTGGGCGCAGCTCACCGCAACAACCATCTTCTGAAATCGACAGAAATTAATATTCACAGGCTTAAACTTCCTCGAGTCACCTCTGACACGCATCTTGTCTTTTCAACCAAATGAGAAAGGTGGGGTGAAGAGTGTTTTTACTTGTTGGTTTTGGTGTTTGGTTTTAGCGAGATTGGCTGGAGCTGGAGGGTTATTGCGTGACGGTATAAAATGAAGGGTATTTAATTATTTTTATATATTTTAGTTGAGAGAGAGGGGATAAATGCTTCCAAATACGTTCTTCTTTATTCTTTGGATGCAGCTTGTACATATTCTTAGTGCTATGTTGGTCTTTTTCCCCCCTCTGTGAAATAGTGCTGTATTAGTCTTTAGCGTATTCTTGGACTATGATCCTTCAATCTCTCTGTTCTGAAAGAATTGCTCTGTGAAAGACAGAGTTTCC

>PvRGL028

GTTACGGTCCAGAAGTAATGGAGGTTCCAAAAGGGAATTGGCTCCTCCCAACACCAAGGAAGGACATGGAGCCGCCGGCCAAATCGCTGCTCAAACTTTTACTTTCCGTGAACTTGCAACTGCAACCAAAAACTTTAGGCCGGACTCCTTTGTAGGGGAGGGTGGTTTTGGAAGGGTCTACAAGGGCAGGCTTGAAACCACTGGTCAGATTGTTGCAGTCAAACAGTTAGACAAAAATGGTCTACAGGGTAATCGGGAATTCCTTGTAGAGGTTCTCATGCTCAGTCTTCTGCATCACCCTAACCTTGTGAATCTCATTGGATACTGTGCGGATGGGGAACAACGCCTCCTTGTTTATGAATTTATGGCTTTGGGATCATTGGAAGATCACCTTCATGATCTTCCCCCTGATAAGGAACCACTAGATTGGAACACTAGAATGAAAATAGCTGCCGGTGCAGCAAAAGGATTGGAATACTTGCATGACAAGGCAAATCCTCCTGTCATTTATAGAGACTTCAAGTCGTCTAACATATTACTTGACGAAGGTTACCAACCTAAGCTTTCGGACTTTGGTCTTGCAAAGCTTGGTCCTGTTGGTGACAAATCACACGTTTCCACCCGAGTCATGGGAACTTATGGTTACTGTGCCCCAGAATATGCTATGACTGGACAGCTGACTGTGAAGTCTGATGTATATAGTTTTGGGGTAGTCTTCTTGGAGCTGATTACTGGGCGTAAAGCCATTGACAGCACCCGACCCCATGGAGAACAAAACCTTGTGACATGGGCACGCCCACTGTTCAATGACCGGAGGAAGTTTCCAAAGTTAGCTGATCCAGAGCTGCATGGACGATATCCCATGCGGGGTCTTTACCAAGCTCTAGCTGTGGCATCAATGTGCATTCAAGAACAGGCTGCAGCGCGTCCTCTGATTGGGGATGTGGTGACAGCCCTTTCTTTTCTAGCGAACCAGGCATATGAGCATAAGGGAGGTGGTGATGATAAAAGGAACAGAGATGATCAAGGTGGAAGAATATTGAAAAATGAGCAAGGTGGAGGATCTGGAAGGAGATGGGATTTGGAAGGGAGTGAGAAAGATGAGTCCCCACGTGAAACTGCAAGGATTTTAAACAACAGGGATCTTGATAGAGAACGTGCAGTGGCTGAAGCCAAGATGTGGGGAGAGAATTGGAGGGAGAAAAGACGACAAAGTGCCCAGGGCAGTTTTGATGCTTCTAATGCCTAGTCTCTCTCCATTGTACAATTCCCATCACCCTCTTCTGTGTCTTCCATGCACTAAGTTTTATTTTATGTATCTTTCATTTACCTCCTGCACGCGAACATATTGGAGTTGGACTTGGAGAGTGTACATTATTTGCCACAAGTAGGAGGTTAAGTTTGATAATGCTGTTACTGTTATTGCTTAAGTAAACACAAAGTTAGAATTTCAATGGAATCAAATGTGTAAAACACACAAAAATATTCTCATGC

>PvRGL029

ATGAGTAGAAAGGTTATGGTCTGATCCTAAACATTTCTCTACCAACTTTTCAATTTCCTTCTTTCTTCAGCAGCACACAACTTTCATCTCTTCCATGTCTTTCTCTGATGGTGATGATGAGATGGAGATGGACTTTCTTCGTGATCGGTACGAGGACAGCAGAAACTATGAAGTGTTCTTGAGTTTCAGAGGGGAAGACACGCGTGCTTCTTTCACTTCCCATCTCTATGCTGCTCTTCAGAATGCCGGAATCTTTGTTTTCAAGGATGACGAATCACTTCCCCGCGGAAGGCAAATTTCACCCTCGCTGAAGCTGGCGATCGAACAGTCTCGAATTTCTGTTGTCGTTTTCTCCAAAAATTATGCAGAGTCGTGGTGGTGTTTGAAAGAGTTGGAGAAAATAATGGAGTGTCACAGAACCATAGGGCATGTGGTGCTACCAGTGTTCTATCACGTCAATCCCATTTATTGTAGAAATGAAGAGTGGTTGTCTTCACGGGGGAAATTTGAGCAGGACCCCAGCAGCTGCATTTCGAGGAGTCCAAGTGTTCACATATAGAGAATTAGAAAATGCTACAGGTGGATTCAGTGAAAGAAATGTGATTGGTAGTATTGGAGGGCTTGGTTTGATGTTTAGAGGAGTTCTAAGTGATGGCACTTTGGCAGCAATTAAGTCGCTTCGCAGTGAGGGTAAGCAAGGGGAGCGTGCCTTCAGAATAGAGGTCGATCTCCTTAGTCGCTTGCACTCTCCATATTTGGTGGAGTTACTTGGCTATTGCGCTGACCAACACCACAGGTTATTGATTTTCGAATACATGCCCAATGGTACACTTCACCACCATCTTCACTCCCCCAACGATCAAACTCAACCGTTGGATTGGTGGGCCCGGATGAGGATAGCCCTTGATTGTGCCAGGGCACTGGAATTCCTTCATGAACATGCAGTCTCTCCTGTTATCCACAGAGACTTCAAGAGTAACAACGTTCTACTGGATCAAAATTTCCGTGCCAAGGTGTCTGATTTTGGATTGGCTAAGATGGGATCAGAGAAGAGGAACGGTCAGGTTTCCACCCGTGTGTTGGGGACCACTGGATATTTGGCACCAGAGTACGCCACGGGGTAAGCTTACCACAAAGTCAGATGTTTACAGCTATGGTGTGGTTCTTCTCGAACTCCTCACGGGACGTGTACCTGTTGATATCAAGCGGGCCCCG

>PvRGL030

GAAATGAATAGAAATATTAGGAAAATGAATAACGGGTAACATTCANATTTGGTCANAGCCGATTGCACTACAGAATTTGAGTGGTTGACTCTGTATGTAGGTAGTGAGAGTGTTGTTACAACACGACCTTAACGCCCTTTTGGATTCATGATCTGGGAAAACACAGCACTTGGGGTTAACCCATCAGAGTCTTCACTAGCCAAGCTTCTACCTCCAATGCTCATTGAAATGCCACTGCTTCTGGAGTCAGTCACATTACCATCATAACCCCCCATCGCATCAGAGTCTTTCTTTCCTTTAGAATCTGTATAGGTCGGCTCCACATCATTGCCAATGCCGCCAAAGCCGTTGCCACTTTCCTCTGCACTTTCTTGCAGCTGCAAAGCAAATTCAAGGTTCCACAAGACATCACCCATGGATGGCCTTTCAATACCCTGGGTCAGCCACACACTTCATCGCAGTCTCGGCAAACTTCTTGAAGCATTCTGGAGCTATCTTGCCTTTTAGATAGGGATCAATGATTTGGTCAAGAGTGCCTTTGAGGTAGCAATGAGCAGCCCACTCAGCCAGACTCACTTGCTCCTTAGCAAGGGCCGGGTTCAAAGCTGGTCGAGCACACAATATCTCAAAGAGAACCACCCCAAATGAGTAAACATCAGATTTGTCAGTTAGTTGCTGCCTCCTGAAGTATTCTGGATCCAAGTACCCAAAGCTACCCTTTACTACAGTACTTACATGGGTATTATCCAATGTTGGACCTGTTTTTGACAATCCAAAATCAGAAACCTTGGCCACCCACTTCTCGTCCACTAAAATGTTTGTTGTCTTCACATCACGGTGGATGATTGTGTATTTAGCACCAGTGTGTAGATAGTGTAAACCCCGAGCAGCTCCAATGCATATCTCAAGCCTTTGCTTCCATGGAAGTGGAGGTTTCTGGGTCTTGTACAAATGCTCCCTTAGCGTTCCATATGCCATATAATCATAAACGAGGATCATTTCAGTGTTTTCTTCACAGTATCCAATCAGAGAAACAAGGTGGCGGTGACGAACGTTTAGAGAGCATTTCAATCTCAGTTTGGAACTCATGCACCCCTTGGTCAGATAGTGGATTCCCACGTTTAATTGCTACTTTGGTTGTTCCACCATCAATTTCTCCCTTGTAAACCTTACCGAATCCTCCCACACCAAGAAGCAAAGCCTCATCAAAGTTGTTTGTGGCGGACTTGATTTCAGCAAATGAGAAATGACGGCAAAGGTTTGATGGNAGA

>PvRGL031

GCGCCAGAGGAGTGGAACCTCATCAACAAAGCACCCAGACAGGGCTGAGGAGTTCACCCTGGCTGAGCTTGTGGCAGCCACCAACAATTTCTTACTTGAAAACAAGATTGGAGCTGGAAGCTTTGGTGTTGTGTACAAAGGGAAACTCGCGGATGGACGCGAGGTGGCCATCAAGAGGGGTGAAACTGGTCCCAAGATGAAGAAGTTTCAAGAGAAGGAAAGTGCATTTGAGTCTGAATTGGCCTTCTTGTCTCGCCTACACCACAAGCACTTGGTTGGACTTGTTGGGTTCTGCGAAGAAAAAGATGAGAGGCTCTTGGTGTATGAGTACATGAAGAATGGGGCGCTGTATGATCATTTGCATGACAAGAACAATGTGGAGAAGGGTAGCAGTGTGTTGAATTATTGGAAAATGAGGATCAAAATTGCTTTGGATGCTGCTCGAGGAATAGAATATCTTCACAATTATGCAGTTCCATCAATTATTCACAGAGACATCAAGTCTTCTAACATTCTCATTGATGCTACTTGGACGGCAAGAGTATCAGATTTTGGATTGTCTTTGATGAGTCCAGAACCTGACCGTGATTACCGACCAATGAAGGCAGTAGGAACCGTTGGATACATTGATCCTGAGTACTACGGTTTAAATGTATTGACTGCAAAGAGTGATGTGTATGGGCTTGGAGTTGTACTACTCGAACTTTTAACAGGAAAGAGAGCTATATTCAAGTATGGTGAAGATGGAGGCACCCCACTAAGTCTGGTGGACTTTGCAGTGCCTCGTATTTTGGCTGGAGAAATGGTGAAAATTTTGGATCCAAGGGTTGGACCACCCGATGAGAAAGAGGCAGAGGCTGTGGAATTAGTGGCCTATACAGCTATCTATTGTGTGAATTTGGAAGGGAAAGATAGACCAACCATGGCTGACATTGTGGTCAATTTGGAGAGGGCTTTGGGTATTTGTGAGAGTAGCCATGATAGCATTTCCAGTGGCTCTATCTCTGTTGTTTCAGAATGATAAAGTTCAGCCCCGGGAAAGGGGACAAAATATTGAAAATTTTGTGAATTCTTATTCATTCTTTCTCAATTTCATATCTTTATTGATTCTTTCCAATATGGGTATTCCGTGCCTGTAAAGATTTAGAGTATATTATGCTCTAATTTGTAGATTGTAGAATCTCACAAACTTGCCTATTGCCTACCAGAAAAATTGACCAGTTAGAGTCTATCTGACAGTTTAACTTAGTACGTAACTGTTTAAGGTAGTCTGGCGGGAAGTGTCTGATGCACTTTGTATGTGAAAACCTTTTGCTGTTAAATAGGAGCGTCTATAGTCTGATTGACCATCTACACTAGAACTAATTTAGTGTGATAAACCTGTATCATGCGTCATTATTCTGTTGAAATATAAATTGTGATGTGATCTGTGGAGTT

>PvRGL032

TAGGGGGGGTCAGGTAGTTAAACATTGACGTTTTTTTTTTTTTTTTTTAAATCAAAAGATAAATATGTAGTGCATCAACCACATGGGTAGTGTTGCAGATTTTTACAGGGTTCAAAATTAGTGAACACCATAATCTTAAGGATCCACATTAGACTCTTGCATGTCTAACAATCAGGACCACAACAACAACAAAAATTCTATATATAGCACAAAATATAACAGAAATAATGCAAACTATACATTTCTTACATAAATATGGTTCATGTCACACCTAAACCTCTGAATCTTGTGTTTTCTGCTTCCTATTTGTAGAATCACAAAATACTATCAACCGCCAAGGAGAGAAGCTGTAGGTCTGGGATAAGTAGAAGGCATTCTAGGCACATCTGCACCACCATGGATGCCGTTGGAACGGCCTTGGCCGGAATTGCGTAGATGATGATGTTTATGATCAGCACCTTTTCTCTGCATGTCCTTTGATTCCTGAACCTGCTCCAATGCTTTTACCACCTCCTCCATATTTGGCCTGCACTTGGACTCTACAGAAAGACATTGCATAGCAAGTGCAGCTGCTGCTTGAGCTCGACTATGTGAATATTGACCTTCCAAACGTGGATCCATTACACGGAAAACTCTTCGTTTATTAGACAGGTAAGGCTTGGCCCAGTCAACAAGGTTATGCTCCCCAGATGGCTGGTTCTTGTCTATAGCTCGTCTTCCCGATATCATTTCCAGAAGAACTACTCCAAAACTATATACGTCACTCTTGGCAGTGAGATGACCTGTTGCTAAATACTCTGGAGCCGCATATCCTCGGGTTCCCATGACCCTAGTAGAGACATGGCTTTTATCACCAGTTGGACCATCTCTGGCCAACCCAAAATCAGAAAGTTTGGCGTTATAATGTGTATCAAGTAGGATATTTGAAGTTTTAAAGTCACGGTATATGACTTTAGGCTCTGTACTGTGAAGAAAAGCAAGGCCCTTCGCAGCCCCAAGAGCTATTTTCATTCGCAAACTCCACGAGAAAGGCTGAAAGTAAGAACCTCTTCTAAAAAGATGATTTTCCATGCTACCCTTAGGCATGAATTCATAAACCAAAAGCCGATGCTCATCCTCAAAGCAGTATCCTATTAACTTGACGAGGTTAGGATGCTGCAGTTGCCCAAGATAGTTGATTTCAGCCAGCCATTCTCTGTGACCCTGGAAACCCTCTTGGTTAAGCCTCTTCACAGCAACAATCATGCCTATTCCCGGTTTGGTAGCAGTAAGTGAATGTTCATCAATCCAGCCCTTAAAAACTGAACCAAAACCACCCTCCCCCAAGACACTATCTGGGCGGAAATTTCTTGTGGCCACCCTTAGCTCACTATAGCTGAAGCTTTTCCAAATTGGACGATTGCAAGATTTCACCTTCACTCCGAGAAGTGACGGATACAGAGGCTGATGAGTTCCTGCTAGTTGAGCTGATGTCATGGCCACTCCTGCTGACACTTCTCGAGGTTATCCCTGTATTGGAAGGACTCACAGCCTTAATCCTATTACTCCAGCAAGCACCCATCAACAAACCACCCCAGGATGAAGGCAGATCTCAGTGATTACGC

>PvRGL033

ATTCGCTTGGCAGAAAGAAACCCTAACACATAACATACGAAATCTCTCATCTCATATCAAAGCATCTTTAAGGCACATGCTTCACCTGATTACTTCGTCCGCCAGGGGAATACAACTTATCAAGACGATCTATTTTTGAGGAAGAGGACAAGGATGCGTCGGTGGCTTTGCTGCACTTGTCAGGTGGAAGAGTCTTACCCATCCAACGAAAATGAACACCTGAAAAGCCCAAGGAATTATGGAGACAGCAACCAAAAAGGCTCAAAGGTGTTACCTCCTGTGAAGCCTGAAACACAAAAGGCACCACCACCTATTGAAGCTCCCGCCCTATCTTTAGATGAGCTGAAGGAAAAGACTGACAACTTTGGTTCTAAGGCATTAATTGGCGAGGGATCGTACGGAAGGGTGTATTATGCAACCTTAAACAATGGAAAAGCAGTGGCTGTGAAAAAGCTTGATGTTTCATCTGAACCCGAATCAAATAACGAGTTTTTGACCCAGGTTTCCATGGTCTCAAGATTGAAGAATGATAATTTTGTTGAGTTGCATGGTTACTGTGTTGAAGGAAATCTTCGTGTACTTGCATATGAGTTTGCTACTATGGGCTCTCTTCATGACATATTGCACGGTAGAAAGGGAGTTCAAGGTGCACAACCAGGGCCAACTCTTGATTGGATACAGCGAGTTCGAATCGCAGTTGACGCGGCAAGGGGATTGGAGTATTTACATGAGAAAGTTCAGCCACCGATTATACACAGGGATATCAGATCAAGTAATGTGCTGATCTTTGAAGAGTACAAGGCTAAGATAGCTGACTTTAACCTTTCTAATCAGGCCCCTGACATGGCTGCACGCCTCCATTCAACTCGTGTATTGGGAACTTTTGGGTATCATGCTCCAGAATATGCAATGACTGGGCAGTTGACTCAAAAAAGTGATGTCTATAGTTTTGGTGTTGTTCTTCTTGAGCTTCTTACAGGAAGAAAACCTGTTGACCACACCATGCCTCGAGGACAGCAGAGTCTTGTCACATGGGCTACTCCACGATTGAGTGAAGATAAAGTGAAACAGTGCGTAGACCCAAAACTGAAAGGAGAATACCCCCCTAAAGGAGTTGCTAAGCTTGCAGCTGTTGCAGCACTCTGCGTGCAGTATGAAGCTGAGTTTAGGCCAAATATGAGCATTGTTGTTAAAGCACTCCAACCACTTCTGAAGACTCCTGCTCCTGCTCCTGCTCCAGAAAGTTGAAGCAAATGAAATGGTCCACTTTGGTTTCTGCATCTTCTGCACAGCTCCATTCAATTATCTCCAGATTTCATCAAATACTGCAGATATATCTATATATTTTTATTTTTGTTTGAGATTGGATGTGTAGGATTGGATTGGCTCGTTTCATGCCATTGTTAAATTCCATGTATTTATGGCATCGCACTTGTAGTGCCGAGTCTGCTGACATATATTGAGCCATGATGCCACATAGGTTTGGGTTGCGTGGTGCCTATCTGTGGGTAATGTTGTTTGTCAGTTAACGTTAATTCACCCATATTCTTTATTTGTTTGTTGTTATCAAGCTGAGCTGATACTTGTCTTGAAAGATTATCATTTGTTCCCTCTTTTTATTTGCCCTT

>PvRGL034

GATCCTGAATCTCTACCTCGTAGCTTGATAGGCTGTTGTGTATTCAAATGCCTTAAATGTTTGAGTTTCCACATTTCGACAGGCAAAGAAATTGGAGTCATAAAGCTCAAATGACCTAGGTCTATGGTTTGTAGATTCCAAAGTTTAAGTATTGAAGCTGGAACAAATATAAGATGAGGTGTTGCTTCAATTCTCAAGTACCTTAAGTGGATAAAGTTCCCTAAATTAGAAGGGATCTCAAACCATGTTGGTCCAAGCTCCAACACTCGAAGCAATTTGAAGCCTTTTGATACTCATTTCCAGTGTATCCCGTGAACATAGTAATCTAGGCCAGAAGAAGAACCAGGGAACGAATACATGAATGGTAACCTTTTTGTATTGACATAAATGCTCCAATGACCCATCAAAATATTTATTGTATCCAAGTTCAAATGAAAAATCTACGAGGTTATTGTTGGGATTAGTATCTTTTCTGTTTGATGCAAACTTCGCACCTAAACTATAGGCCTCTTTGCACTCTGACATATTTTGCCAAGATCATCGATAGAAGGTCATGCATGATGAAATGACTTCAAGCCTTCGTTAGTCTCCACTTTTGATGCTTGGACCAAGACTACGACCTTGATAGTAAATCATTGAAGTAGTCTGTCTGGCAAACTTCTTCTGAAGTCTTACTATGTGGACGACACTGGGCAAAATTTTCAGTCATCCACAACCCTGAATCAAATCCTCCTTTTTAAACTCATAATCTTTGGGGATCACAGGAATTGCACAGTCTTCAGGCAAAGTATACCTTCAAGATAGGGAAGGACATGGTGTTTAATCAGCTTGGCAAGTTGTCGTAAAGCTGAGTTTGAGAACTAATATCCTACAACTTGGGTCTCATTTTGAGTGAAATTCCCATATTATCGTTTTGGACACACCACGTTTGACCATTCTCTGAAAAAGATGACTTTTCGTTGTATAACTAGACTATCCCATCCTAAGATTTTTAAGGCGAGAGGTAGTCCTTTACATGTTTTAACAATCTTCATGCCAATCTCCCTGCACTCTTGATTTGGTTGAGTATCATCATCTCGGAATGCACTGTTCTAGCAAACAACTTCCAACAATCATCTTCTTGTAATTGTTGTAGGGAGTATTCTTCTGACCACATGGTAGAAGCAACTTCCTTACTACGCGTGGTGATCAAGAATCTTACTCCCTTGGGCCCCTAAAAGAAGGGGTTTCCGCACTTCCTCCCATTTACGTTGGTTTTCATTCCAAACATCATCCAAAATAAGAAGAAACTTTTTCTCCTAACAGTTTTTCTTTCAATCTTCTGTGAACCAGGGTCTCTGGATCTCTAGTGTCACAAGGGTTTTGTGGATGACAAACTTAGGCCAGGTTTGAAAGCTCAGACTGTTGCTGTGAAAAGGTTGGACTTGGATGGCTTGCAAGGTCACAGGGAGTGGCTGGCAGAGATTATATTTCTTGGACAACTAAGGCATCCACATCTTGTAAAGTTAATAGGGTACTGTTATGAAGATGAAAACAGGCTTTTGATGTATGAATACATGCCAAGAGGCAGCTTGGAGAATCAACTCTTCAGAAGATACTCTGCTGCTATGCCATGGTCAACCAGGATGAAAATTGCATTAGGAGCTGCCAAAGGTCTGGCCTTCCTTCATGAAGCAGATAAGCCTGTAATATACCGCGATTTCAAAAGCTTCAAACATTTTACTGGACTCAGATTTCACAGCTAAACTCTCAGACTTTGGTCTAGCCAAGGATGGCCCTGAAGGGGAAGATACATATGTAACAACACGCATAATGGGAACACAAGGATATGCTGCTCCTGAGTACATAATGACAGGTCATCTTACAACCAAGAGTGATGTGTATAGCTATGGAGTGGTGCTGTTGGAATTGCTTACGGGAAGACGAGTGGTGGATAAGTCTCGGTCGAATGGTGGCAAGAGCTTAGTGGAATGGGCAAGGCCTTTACTGAGAGATCCGAAAATAGTTTATAGCATCATAGATTGTAGACTAGAAGGGCAATTTCCCATGAAAGGAGCCATGAAAGTTGCTATGTTGGCTTTTAAATGCTTGAGTCCTCACCCAAATGCAAGGCCAACTATGAGTGATGTGGTGAAGGTGTTGGAGCCACTTCAGGACTTCGATGATGTTTTCATTGGACCATTTGTGTATGTTGCCGTAAGTGAAAATGGTAACAAACATAAAATATAGCAGCAGATAACGCTCCTCAGCAAAGTAGATAAAATATACATATCATATAGAAAAGGGTAAAAAGAAAAAAAAGGTGTGTTATTTTATTTTACAAGACGGCATGGCTTGGTCATCATGAAAAGGGTGGCAGGTAGTTCTATCACATCTGACTGTGGCCAGAAATACAGAGTTGCAGCAAAATGATGGTTAGGGTGAAATAAGGGCAATCACATTGAACACAAGCTATTCGAAGTGATAATTGAGTTTTCTATGGTTTGATGATATACACGACAATGGACAAGTATAAGCACTTGATACTAGTTCAGGATACACACTAAAATTTATTCACATGTAATGTATGTGACAAATATGGTTGGGATTGTGTTTGGACATGGTTTTGGATGTTAAGTTATAGTTTAGAGAAAATGAACAAAAAATGGATTTCA

>PvRGL035

TCCAAATCTTGTCACTCTGATTGGTTATCATGCTTGTGAGACAGAGATGTTTCTCATATACAATTATTTGCCAGGTGGTAATCTCGAAAAGTTTATCCACGAGAGGTCAACGAGGGCAGTAGATTGGAGAATTCTTCACAAGATTGCATTGGACATAGCCCGTGCACTGGCCTATCTGCATGATCAGTGTGTTCCCCGTGTTCTTCACCGCGATGTCAAGCCCAGCAACATCTTGTTGGATGATGATTTCAATGCTTATCTATCGGATTTTGGATTGGCTAGACTTCTGGGAACTTCAGAGACACATGCAACCACTGGTGTAGCAGGAACATTTGGATATGTTGCTCCAGAATATGCAATGACTTGCCGTGTTTCTGATAAGGCTGATGTGTATAGCTATGGTGTGGTGCTTCTGGAGTTGCTCTCAGACAAGAAGGCATTGGACCCTTCATTTTCTAATTTTGGAAATGGGTTCAACATAGTGGCATGGGCATGCATGCTACTGAAGCAAGGAAGGGCAAATGAGTTTTTCACTGCTGGGTTATGGGAAGCAGGACCTGGAGATGATTTGGTAGAGGTGCTTCACTTGGCAATTGTGTGTACTGTTGACACTCTCTCTACCAGACCTACAATGAAACAAGTTGTCAGAAGGCTTAAGCAACTTCAACCTCCGTCATGCTAGCCACGTTTCTGTGGCTTCATCATCTTTAAACATTATTAGTCTTAGAAATATTGTAATTTGTAATTTAGCATTTTTGTGGATTTAGGTTGAGTTTCTCAATTTGTACTTATTCTCCCCCTCTGTACATTTTTATATAGTTGTAGCTCCAAATTTGCAGTACTCTTTTCTTGATTAGGTTAGGTGGCCATGCGTTGATTCTGCAGAAGAATGCAGATATTGGATTTCCCCTCCATTACATGTCTATTTGTTCAATAAAATTGAAGAATAGATTATGTTTTTT

>PvRGL036

GGCAAAAAAGGTAGAAATATGTTACACAAAACATCAAACATAATTCATTTATAACCATGCTAACTACATGAAATAATGATTGTAGAAATATGTTTGTTTATTGAAATAAACTGAAGTGGAACTGCATGTTGTTCTGTCATGAGCGCTGATCATCTTCCATTACCAAAGTCAATTAATTCAAGTGCTCTCAAGCTATCATATCCAGTATTTTGTTGGGCACTACTACTATCACCATGTTCATGGGAGCTTTGTGCCTCATGAAGCAAGTATTGACCCTCCTCAAAGTTTTTGCCTGTATTGATCCAAGGGTGTTTCTCTATAAGCTTTATTCCCTCTAATTCCATTGCCACTTCCTTCATGCTAGGCCTTTCCTCCCCTGTAAGTCGTCAGGCACCTAGCAGCAAGAATAGCAACCTCCATAAGCTCCTGCTTGTTTTCTTCATCCAAAATACCCACCTGAAGAACATCAAATAGACGATCCTCTTTCAAGCTACATAGAAAGTGAACCGTAAGACTTCGTTTCTCTTCTGCCCTGTCAAAACAGAACGGTTTCTCCCCTGTTAGTAGTTCTACAAGCACTACCCCAAAGCTATAGACATCACTCTTTTCTGTCAACTGACTTGATTGCATGTACTCTGGGTCTAAATACCCAAAGGTTCCTTGCACAATTGTGGCTATTTCAGTTTGGTCAAGAGGAACCAATCTAGAAGCTCCAAAGTCAGACACTTTGGCAGTGTAAGTTTCATCCAAGAGTATATTTGCAGTCTTCACATCTCTGTGGATGATGGGTATGGAAGCTGCCGAGTGTAGATATGACAAAGCTGCAGCTGTCTCTGTAGCTATCCTTAGACGCGTTTTCCAAGACACATTAACCACCTCTCCTTGGTTATGTAGATAATCAAAAAGGGTACCATTGTTAACAAATTCATAAACCAGTAAGGGAACTTCTATCTCTAGACAACATCCCAAGAGTTTGACCACATTTCTATGATTAATTTGTGAGAGTATAATAACCTCATTAATGAATTGCTCCACCTGACTCTGATCCACTATTTTGGACTTCTTGATAGCAACAACTTTGTTATTTGAGAGAAGTCCTTTGAAAACTGTACCATAACCTCCTTTACCTATGATTAAGCTCTCATCAAAATTGTTGGTGGCTTTCTTAAGTTGCTCTGCACTGAAAATTGTAGCGGACTGAGATGAGTCTTCCCTAGTGGAGAGTTGTTGYCTCAGAATGATGCCTCCATTCTGCTGAAAGAACTTCTCCTTTAATTTGAGGACTTTCCTTTTCTGGTACATCAAGTATAGCCAAGAAACTCCTATAAACAGAGCAATTAATCCTACACCTGCACCAATGACGATCTTGATAAATGGATCAGCCTTCTGCTTTGGGTGGCATCCTCCGCCTTCTTTTGTCCCATTTCCGATTAGTCCGTCAGGACAGAAACATTCAAAAGAGCCAGAAGTTTCTCTACAATGATCTTCACTATCGCAATTGTGTTTATTCCTTGTACACTCCGGAAAGTCTTGGCAAAGGCCATGGAGAAGGTATGGGTTTCCCTCAAAGCCTGGATTACATTTGCATCGGTAACCATATTCATTGGGCGAGTTCTCACAGGAGCTATTAGCACTCTTGCATGCATAATCTGCTGTATGCATAGAAGCGTCGCACGTGTCG

>PvRGL037

GTCCGAAGAAAACTACTTCACTTTCATTTCAATCTTGAAAAGCAAAATGCTTCCTTTTACTTACAACATAGATGTGTTGGATATGTATAGAAGCTATAATACAATGAAATGTTTCTAATATTCATCTGCTCTACCAGGGTTGAGTCCCCTCCCAAATGAAAATTCAGATAAAGCAATGTTGATTTCATGTTGAGTTCGAACAGGATTGAGTCCAGCAGGAGGCAATTCTGCTGCTGCTGGAGGCTGCACCTGCATGGTCTCCTCATGATCAATATCTCTTGCATGGTGGCTTGGGGAATGTGACCCTTCAGCATCCCATTGCCCATTTTCATAATCGGATGCTCTTGGAGAAACTAGTGGACTATCACTGTGTCTAGGGTGCTTGTGAAGCAGGTGCACTGGAGACATGCCACGAGTTGGGGTTCCTGGTCTGCTTGAGAATGGTGTGTTAGAAGTTGAATCCCTGTTGTGTTTCAGATGCTTTTTAGCAGAGTGATGCCAGCTCTTTAGTGCTGATGCCACGTTATCTTTGAAAATGGTGGGTTTCATGCTAGAACCCATCTGTATGACTAGAGCATAAAGAGGCAAAGTCACATAGCTGCATAGAACTTGGGTGAGAACCCCCATTGTAAGTCTAATGACAATATCTGCAGTAGTTCGGTGAAAGCAGGAGTTTATTTTGAACCCATTGTCTAATGTGCTCCAAGTAAAAAACGCCAATTGAAATGCATTCAGGAACAGAACAAGATGAATCAGAAAGAGAACGAGGCGAGGATTATTGAACCAGAACAATTCATCTCCTGGCTGAACCACTGGTTCACCCTTGACCACTTCCCCCCTATCTTGAATCTTTAGTGCCATAGTCTGTTATGATCATTTGTAGCTTTGTTCCCACCAACAAGATTATAATTAGTGGGATAAATGGAACCCAAAAATAAGAATACCACCCGTGAGTATTTGTGAGCAGAATTACCACTGCAAAGAACCATATGGTTGGACTTATGCCCACCACTACTGCAAAATCCTCATCTAGTGTTCTCGTGATATAGTTCTGGAAGTCAAATTTTGGCTCACTTGCTGGAGTCAAATGTGCCACGACGAATCCATGCCGTAATGCCATATAGTCAGCTTTATTAAGTGATCCATAGAATTGTCTGCAGAAGCTAACTATCCATAATAAAGTTGGTGATTTACTCCATGAGTTCAAGTGCCTTCGTCCAAACGTTGTATCCCTAGCAAACCTGAACCTCTCAGGATCATGGGAGAACTGATGTTCAAGACCCTTTGTTTCGTCTTCCCACTTCCTCCATATTGATATCTTAGTTCTACCCAAAGTCAGTGTCATGATGCACTGTAGAATGTGAAAAATAGCAAGCACAAAGACGAATATATGGAGCTGGTGAAGCCCATATTTAGACATAAAAGCAACTGTGCCTTCCTTTTTTTTTGCGCATTTCTCAACATATCCTTCTGCACCTTTACTCACATAACTGGGATCACAAGGATTCCAAGTGGATGCAACACTTTCTGGGATGCAGATAGAAACAATTCGATCTTCAAACACCACTAAGAGAAAGGATAGGAATCCCAGCAGCATAAGCTCTCCTTTGATCTTTTCCAGCGCTTCATGAAGAGCCTTTTTGTGTTTCTTTTTTAACCACTTTCCAAGTTCTTCAATAACATGTTCAATGATGATTGAAATAGCAAGCATCACAAAGCACACAGCTGCAACTACCCATGTAGGAGTTGCCTCCAACTTCCTCTGTAGAACTTCTTCTTTGCCAATGTATTAGCCTCTGCACTGATATATATATAAGTATCTCTGCAATTTTTCCTGTGTTAAAGAGAAGAGTGTGGTTGAAAACCCAAATTTT

>PvRGL038

CCGTTGCTGTCGACGAGTTGCTACCGAGGACTTTTCTGATTCTAATAAACTTGGACAAGGTGGATTTGGAGCTGTTTACTGGGGTAGGCTTTCCAATGGACAGATAATTGCAGTCAAAAGGTTGTCAAGAAATTCCGGGCAAGGAGATACGGAATTTAAGAATGAAGTGGTTTTAGTTGTCAAGCTTCAGCACCGAAATTTAGTTAGGCTACTTGGTTTCTGCTTGGAAGGAAGAGAAAGACTACTTATCTATGAATTTGTTCCCAATAAAAGCCTTGATTACTTCATATTTGATCCATCCATGAAAGCCCAATTGGATTGGGGAAAGCGCTTCAAAATCATTACAGGTATTGCTCGAGGTCTTCTCTACCTTCACGAAGATTCTCAGCTACGAATTATCCATCGAGATCTCAAAGCAGGCAACATTCTCTTAGACGAAGAGATGAATCCTAAGATAGCAGATTTTGGCACGGCAAGACTGGTTTTAATGGATCAAACTCAAGTAAATACAAACAGAATTGTTGGAACCTATGGATATATGGCACCGGAGTATGCGATTGCACGGACAGTTTTCAGTGAAATCAGATGTCTTTAGTTTTGGTGTACTGATCCTTGAGATTGTAAGCGGGCAGAAAAATGGTGGAATTAGTAATAGAGAGAATATGGATGATCTACTGAGCTTCGCTTGGAGAAACTGGAAGGAGGGGAAGGCTATAAATATTGTAGATCCATCACTGAACAGCAATTCACGGAATGAAATGCTGAAATGCATCCATATTGGTTTAATTTGTGTTCAAGAAAATTTAGTTGATAGACCAACCATGGCTAACATTATGCTGATGCTTAACAGCTATTCTTTGAGTCTCCCAATTCCTGGAGAACCAGCATTTTATATAAACAGTAGAACTAGAAGCCTTCCAGAGACGCAGTCATGGGAGTATAATTCAAGGGAAACAGGATCACGTGAAGCGAAACTTATATCAGCTCAAGAATCAGAAAACGAAGCTTCAATAACTGAGCTATACCCTCGCTAGATGTTGGATTCATTCATTATCCATCTTTCTTCTCTTACAACAAAATCTAATGTCTTTATGGCAAAATCTTGTTTAAACACATTTTGTTGTAGTCTAATTAATTTATATCTGTAGTT

>PvRGL039

ACCGAACATAACTTAACAAAACATTTCTTCTTCTTCGTCTTCAAACCTCTTCTTCTTCATCTTCAAACCTCTTCATCATCCATTTTCTGTTCCAAACAATACAAAAGCTGTAACCACACACACCGTCTTCGCTTTGAATTCCGAAAGAATTCAGGATCATGGGTTGCTTTGGCTTCTGCAAAGGAGATGACAGTGTGGCAATTTCTGACAGAGGACCTTTCATGCAAAACACTCCCACTGGGAACGCAAGTTATCATGGCAGACATAATGCAGTGACTGCTCCTCGGCCAATAAATTTTCAACCTATTGCTGTCCCTTCTATTACAGTAGACGAATTGAAGTCTTTAACAGATAGCTTTGGCTCAAAATCTTTCATTGGTGAGGGTGCATATGGGAAAGTATATCAAGCTACATTGAAAACTGGGCGTGCAGTGGTAATCAAAAAGCTAGATTCCAGTAATCAGCCAGAACAAGAATTTCTTTCTCAGGTCTCCATAGTATCAAGGCTAAGACATGAAAATGTTGTTGAGCTTGTTAACTATTCTGTTGATGGTTCTTTTCGTGCCCTTTCCTATGAGTATGCTCCTAAAGGATCCCTTCATGATATTCTACACGGACGAAAAGGTGTCAAGGGTGCACAACCTGGTGCATTACTCTCGTGGGCTCAGAGAGTTAAAATTGCTGTTGGAGCAGCCAGGGGACTTGAATATCTTAATGAAAAGGCAGAGATTCATATAATCCATCGTTATATTAAGTCTAGTAACATACTTCTTTTTGACGATGATGTTGCAAAGATTGCTGATTTTGATTTGTCAAATCAAGCCCCTGATGCAGCAGCACGTCTTCATTCTACCCGTGTTCTTGGGACTTTTGGTTATCATGCTCCTGAATATGCAATGACGGGACAACTCACTTCAAAAAGTGATGTTTATAGCTTTGGAGTTATACTGCTGGAACTCTTAACCGGGCGCAAACCGGTTGATCATACACTACCCCGAGGACAGCAAAGCCTTGTCACCTGGGCAACACCAAAGCTTAGTGAAGATAAGGTGAAGCAGTGTGTTGATGTTAGACTAAAGGGAGAGTACCCTTCAAAGTCCGTTGCAAAGATGGCTGCTGTTGCTGCATTGTGTGTACAATATGAAGCTGAGTTTCGGCCAAACATGAGCATTATAGTGAAAGCTTTACAGCCTCTACTGAATACTCGTTCTACTCCCTCAAAGGAATCACCAAACATGTAAATTCCAAAATCATTCATTCCTTCTTTCAGATTGCAGATTGCAGATTTCATTCACCAATTGTCCTTGTGACAGTTGTCAGTAATTTACTCGTTTTGTGGCATACAGCATTAAAGTACCATTATTATATTTGTGGCTCAACTAACCGATGCTATATAAATAGTGATGTAGGTGACAGGAGCCTATCATAGATACTTAAGTTTATGGCTTTTGAATACTTTTTTTTTAATTTTTGTTTGGATTTCCTTTTCGACAAGTCATGTAAAAGTGAATGTATATTACCATATACCTTTCATGGACAACACATGATGTCTGGTTGAATTGCCATCTTTAACTTACATTTCTTCATTTTTTTTCCTTTTTGATGTTATTAAGTTTTATATTGAATAAGGTGTTTGACAGATTTAATACGC

>PvRGL040

CCCTGATTCAGCTTTACTTCAAACAGGCAACAAGGCTGAAGGAAGTATTCCTTCTATCATGGGAGTTTGTTTGAGCACCAAACCTAAAACTGGGTTAAACTCAAAGCATGTGAGTGCCGAGGCCAAAGATCTCAGCAGTCCAAGTAGTAAAATTACGAAAGATCTCAGTAGCACAATTAGTAAGGTCTCTGAGGTTTCGGTGCCTCATACCCTTCAGAGTGGAGGTGAGATCTTGTATTCATCCAATCTGAAGAGCTTTAGTTTGACAGAACTTACAGCTGCCACAAGAAATTTCCGTAAGGACAGTGTGTTAGGAGAGAATGGTTTTGGATCAGTTTTTAAGGGCTGGATTGACGATCACTCACTTTCTGCAACCAAACCTGGCACGGGGGTTGTTGTTGCTGTGAAAAGACTTAGCCAGGATAGCTTCCAAGGTCACGGGGAGTGGTTGGCAAGGCATGGTATGACTCATGAAGCAGGTCTCGAGGATGAAGTAAACTATCTAGGACAGCTTTCTCATCCTCATCTAGTGAAATTAATTGGATATTGCTTTGAAGATAGAGACCGTCTTCTGGTCTATGAATTTATGCCTCGTGGTAGCTTGGAGAATCACTTGTTCATGAGAGGCTCATATTTCCAACCTCTTTCTTGGGGCCTGCGTTTGAAAGTCGCTCTTGGTGCTGCCAAAGGGCTTGCATTTCTTCACAGTGCTGAAACAAAAGTGATATATCGAGATTTCAAGACCTCAAATGTCTTGCTGGATTCAAATTATAATGCAAAGCTTGCTGATTTGGGGCTCTTAAAGGACGCACCTACACGTGACAAAAGTCATGTCTCCACCAGAGTAATGGGAACCTACGGATATGCAGCTCCAGAATATCTAGCCACAGGTCATGTGAGTGGTAAGAGTGATGTCTTCAGTTTTGGAGTTGTGCTGCTAGAACTGTTATCTGGCAGGAGGGCTGTGGACAAGAACAGACCATCAGGACAGAACAATTTGGTGGAATGGGCAAAGCCATACATGGCAAACAAGAGAAAGCTTTTGAGTGTGTTAGATAACCGTCTTGAAGGCCAATATGATATAGATGAGGCTTTTAAGGTAGCTACCCTCTCTATGCGATGCCTTGCAACAGAATCTAAGTTGAGACCAAACATGCAAGAAGTTGTCACAGATTTGGAGCAGCTGCAGGTTCCCCATGTACATCATATTCGTTCTGTGAATACTTCTCGTAGTCGTAGAAAAAGTGCTGATGATTTAATCCATGGTAGAAGCGTTACAACTTCAGTTTCCCCTCTCTCACATGACATTGCAAATACACGTCCTTGAAAATTGCATGTTACCACATAAACTTGAACACGTTCATATCACACATGTTTATGTAATAGGCTTTACATATCTTAGTATTATTGCATGTACATATTTTAGGTTCTGTTTGTAACATTAGTCAAAATATCTGGAACTGCAGATACTTTCTTGTGTAGCTCTGGTTAGCTCAATTTTGTTGTTTTTCAATATTGGTAAGCAGTGTTCCTTCTCTATGTTGTTAAACTTAATTGCAATGTGCATCAAAAAAAAAAAAAAAAAA

>PvRGL041

AGCACAANGCTTTTCAATGTATTTCGTATGTAGAAGTCCTATAAGGCAATGAGGTGTTACAAAATAGCCTAGGAAATCTATAGACAACAGAAATGAACAATCATACAAATCTACCTGCCTTCCCACCTAGTTTAATAGTAGTTACACACACTTTCACTTTGACAGATCTCAGAATACGAGTAATACTAATGGTGGTGAGAGGAGTTTGGTACTGAATAATATATTGCGTGAAATATGATATGTACAGTGTAATAGCTACACAGGTGGCTGAGGTCATGGAGAAGATGAGAAGAAGCATCATGTTAAGTTAGAAGGCTGTGTTTGAAATGTCAAGTATAGAGAGGGGATGCAGAGGGCCTGGGATAAGTTGAGGGAGTCTCCATGCGGCTAAAGTCATCAGCACTTCTTCTACGAGCTCGAGATCCATTAACAGAAGGATTTTGATAGCCTCTACTTACATCAGCACTTCTTCTTCGAACGCGACAAAGATTTTGATTGCCTTCATTTGCATTAGGAACCTGCAGCTGCTCCAATGTTGTAACCACTTCATCCATGTTTGGCCTGAACTTGGAATCTATTGACAGGCACCGCAAGGCAAGGTTGGCTACCTTATAGGCATCATCCGTTGAATATTGTCCTTCAAGCCGGGTGTCTAACACACGAAATATCTTACGTTTGTTTGCTAGAAATGGTTTACCCCATTCCACCAAACTGTGTTGTCCAGATGGTCTGTTCTTGTCAACTGCTCTCTTTCCAGATAACATTTCCAGCAAGACAACTCCAAAACTATAGACATCACTCTTGGACGTGAGATGACCTGTGGCTAGATATTCAGGAGCTGCATATCCGTAGGTTCCCATTACCCTGGTGGAGACATGACTTTTGTCACCCGTTGGTCCATCCTTTGCCAGCCCAAAATCAGAAAGTTTTGCATTGTAATTTGAATCCAGCAAAATATTTGAAGTCTTAAAATCTCTATATATCACTTTTGTTTCAGCACTGTGTAGAAAGGCAAGCCCTCTGGCAGCATCAAGAGCAACTTTAAAACGGAGGCTCCAAGAAAGTGGCTGAAAATATGAGCCTCTCCTGAACAAATGGTTTTCCAAGCTTCCGCGAGGCATGAATTCATAGACGAGAAGGCGGTGTTCATCTTCAAGGCAAAACCCAATCAATCTCACCAGATGAGGATGAGAAAGTTGTCCAAGGTAATTGACTTCAGCCAACCACTCCCTGTGACCCTGGATGCCATCTTGATTAAGTCTTTTCACAGCAATAACAATGCCAGTGCCTGGTTTTGTAGCAGTCAATGAATTCTCATCAATCCACCCTTTAAAAACTGATCCAAAACCACCTTCTCCTAACACACTGTCTGGACGGAAATTCCTAGTGGCCGTCTTAAGTTCTGATAAGGTGAAGCTCTTCAAATTGGATGACTGCAAGATCTCACCCTCACTCCGAGGAGTCTGAGGGACTGAGTTCCCTGAAACCTTATCG

>PvRGL042

GGCAGGTAACAATAAAAATAAATAAATAAAAGAACCAAGTAAATCTCAGTACCTATGGATTATGAACAAAATGAATGTTGCGTAAACAATAGGATATATAAACATGTTCAAGCAATCTTCCATCCGCTTCTGAAGCAGTCACTTTCTTACATCTCGCGTGCTCACTTTGCACATACTAATTGATACAGATAGACAGAAATACAGTCAAAGATTAGTTTGAAGTTTCACCAGCAGGTCCATGTCTTGCATTCAACAAAGGTTGGAGAGCTTTGACTACAATGCTCATGTTTGGTCTGAAATCAGCTTCATATTGTACACACAGGGCAGCAACAGCAGCCATCTTAGCAACAGCTTTGGGAGGGTAATCTCCTCCTAGTCTTGCATCTACACACTGTCTAACTTTATCCTCACTGAGTCTTGGAGTAGCCCAAGTAACCAGGCTCTGTTGTCCACGTGGTAGTGTATGATCAACAGGCTTCCTTCCCGTTAAAAGTTCCAGAAGAACGACACCGAAGCTGTATACATCGCTCTTAGCATTCAATTGTCCAGTCATTGCATATTCTGGTGCATGATAACCAAAGGTTCCAAGGACACGGGTGGAATGGAGTCGTGCTGCCATGTCAGGAGCCTGATTTGACAAATCAAAATCTGCAATTTTAGCAACATCATCGTCAAAGATGAGTACATTGCTTGACTTGATGTCCCGATGGATAATGTGAGGATCAGCCCTCTCATGCAAATACTCGAGTCCTTTGGCTGCCCCTACGGCAATTTTAACTCTTTGAGTCCATGTTAGAACAGGACCAGGCTGTGCTCCTTTAACACCTTTTCTGCCGTGCAAAATATCATGAAGAGACCCATTAGATGCAAACTCATAAGCAAGAATTCGGGAGTTTCCATCAATGCAATAACCAAGCAATTGAACAAAATTGTCATGTTTCAGCCGTGAAACCATGGAAACCTGGGCTAAAAACTCGTCATCGGGCTGTTTACTGGCATCTAATTTCTTGATTGCTGCAGCCTGCTCACTTTTCAGAACACCGTAATACACTCTTCCATAGGATCCTTCTCCAATCAAAGAACTTTCTCCAAAACCATCAGTAATTTCTTTGAGTTCATCTACTTGTAATTCAGGAACTTCAATGGGCTGAATTTTAACAGCTTGAATGCCCTGCTTTGCAGTTTCAGATGCACGACCATTTCCATCATTTCCTGTTGAGTTTTTAACAAGATGTTGTCCTCCACTTTCAGCATGCTTCTGGAAGTCATCTTCTTCGCAACAGCTAAAACAACTCATGATTCTTTCTGCTGAAATCCAACAGGATATTGTGTCGTTGAGAAAAAGTGGATAGCTGCCGCTGGAGGAGGATGATCTCGGCGGAAAGCTGCTAAATATGCGCAGGATAATCAGGGC

>PvRGL043

CAGAATAATTGAACCTTTAATAAATAAGTATAACCGAAGTCGAACTAAATGTGAGTTATGCTGATAGTCTGATCTATACATACATCCCCACCTATTGGATTGGACGCACAACATCACGCTTTTAACGGAATACATTCTGAGTGCACAGATGTTAAAAAATGTTTTACATCTACTCTGGCTAAATATTCTAATAAACAGCGTCCTGATCACTCGAAATTGGAATAAAGACAACGGAGGATGCTTAAGCCACAATTTCATCTCATCTGAACATATACCAGTCAGATCCACCTGCTCAAAATTTTGTGGAAATTTTAATTGCCATCCACGTCGGTGAAATTTCAACTTCTGGAAGAGAAGACGACTTTTTCGCGAGCACAAACTCTAGTGAAGAAACTACATGGATTAGAGTCATACAAGGCTGGCTTCAGCCTTTTCTTTTCGTCATGCTTCACACGTACAAACCTCATCACATTTAGTTTCGTTTTGTCTTAAGTTGGAGTGTCATTGCTGGCCTTGGGAACCTCATAAAGGGTTACACCACTGCTACCAGCAGTTTGAAGCATTTGATCTTCCTCATTTTCCCCACTTGACTGAAAGTTTTCGAGAATTTCAACAACTTGGCTCATTAGCGGTCTTCCTTTCGGGTTTTGGCTAAGACATTGGTATGCCAAATGGGCTACCTTCAATGCAGTTTTACTTGAATATTGTCCCTCTAATCTTGGGTCTAAAATTTTCAAAAGTTTCTTATTATGATTCAGAAGCGGACGGGCCCACTCGACCAAGTTGTGTTCTCGGCTCGGCCTGCTCTTGTCTAGTGCCCTTCTACCAATAAGCATTTCAAGTAGCACCACCCCAAAACCATAAACATCACTTCGAGCTGTCAAATGCCCAGTCATGACATACTCGGGAGCAGCATATCCATATGTGCCCATTACTCGTGTTGAAACATGGGTTTGGTCCCCCATCGGGCCATCCTTTGCAAGACCAAAGTCTGAAAGCTTCGCATTGAAATCCGCATCTAGCAAGATATTTGAAGTCTTGAAGTCACGATATATAATAGGTCTTTCTGCACCATGAAGAAAAGCCATGACCCCTCGCAGCATGTAAAGCAATCTTCATTCTTTTTGACCAAGTCAATGTTGAGCCCACTCTGCGAAAAAGATGTTTCTCCAAGCTCCCACTTGCCATGTATTCATAGACCAATAACCGGTGTTCGTCCTCACAGCAATATCCAATGAGCTTCACCAGATTAGGGTGACTGAATTGACCTAAATAGTTGACTTCGGTGAGCCATTCCCTGTCTCCTTGGAATCCTTCGCGATTAAGTTCCTTAATGGCGACCACAGTGGCCTTGTAACCCGACCTCACACTGTGGTCTATGACGCCTTTATATACAACTCCAAAGCCTCCTTCTCCCAAGATGAAATCGGTGCGAAAATGCTTCGTTGCGAGCCTCAGTTCCTCGTACGTGAAAATATCGACATTGCTATATCCAGCACCTTCGCGGAGATCTTTGATGTTTATGGAAACAATCGAAGCGACAGATTCTTGACCTGGAGGCTTAGGTTTGGCGTTGGAATCGGAAACGGAGAGGTGGCTCTTGTCTTCAATGCTGAAGCAAATCCCCATGAATCTGGATCTGGATCGAAATCGCAGAAAACCGCATTCAGTTTCCCCTTCGCTCTGGAGTACACTTACATTATTCGACGGATTTGAGATGGTTCGAGGGAAAACGGAACCAACTCC

>PvRGL044

CTCTCTCTCTCTTACCAGCCCGCCATTTTTTTCTCTCTCTCCAAAGCTTAAATGACTTCTCCATATACACCTCGCCGGCGATCCAGCTCCGACCCTAGCAGAACGCCGGGAAGAGTGCCCCCTTCTCCGGGATACGCCTCCTCCGAGCTCAGCACCTCCACAGTCTCAGCGCGCCAGAACCCCGTCTCCGCCGCCGCAAGGTTCTTCACCGGAATGTTCGCCGCGTGCATATCGCCGCCGGAATCCGACAATTCCAAGAGCATCGGGGATTCCGAGGAGTTTAAGTCTTCTTCCACTGCATCGAATGGTTCAAGAGCTGGTAGTAGGAGAGGGCGTGCCTCAAATGGAAGTATGAGCATCAGTTCATACAACATAGTACCAAAGAAAGAACCTGGCATTGTGAAGTTCACCATGGAGGAAATCTACCAAGTCACAAGAAATTTCTCCCCTTCTTTCAAGATTGGGCAAGGTGGTTTTGGTGCTGTGTACAAGGCAAAACTCCTGGATGGGTCTGTTGTTGCAGTAAAGCGTGCTAAGAAGAGTCTATATGAGAAACATTTGGGGGTGGAGTTCCAGAGCGAGATCCAAACACTATCCCGGGTGGAACATTTGAACTTGGTCAAGTTTTATGGATATTTGGAGCAAGGGGATGAAAGGCTCATTGTCGTTGAGTATGTTCCAAATGGAACCCTCAGAGAACATTTAGATTGCATTCATGGAAGCGTTCTGGACCTTTCTGCACGTCTGGAAGTAGCAATTGATGTTTCTCATGCTATCACCTATCTTCATATGTATATAGATCATCCTATCATTCATAGAGACATAAAATCTTCGAACATTCTTCTCACTGAACATTTTCGAGCTAAAGTAGCAGACTTTGGTTTTGCGAGACAAGCCCCAGACAGTGACTCTGGCATGACCCATGTTTCCACCCAAGTCAAAGGAACAGCTGGCTACTTGGATCCTGAATACCTGAAAACTTATCAACTAACTGAAAAGAGTGATGTGTATTCATTTGGAGTTTTGCTTGTTGAACTTGTCACCGGCAGACGCCCCATTGAACCAAAATTTGAACTCAAGGAGCGAATAACGGCAAAGTGGGCTATGAAGAGGTTCATGGATGAAGATGCTATCTCGGTCTTGGACCCAAGACTGGGTCAGACTTCTGCAAACACTTTGGCACTGCAAAAGATTCTGGAGCTAGCTTTACAGTGCTTGGCTCCACGTAGACAAAACAGGCCTACTATGAAAAGATGTGCCGAGATCCTTTGGGCCATCCGCAAGGATTTCAGAGAGGAGTTATCAGCTTCAAACTTTCACTCATTTTCCACTACTTCCCAAAGGAGCACCTCACTGAGAGAGTGACATGGCTAGTTGCAACATTGCATAAAAAAAAAATGCTGGAAACACTCTTTCGGATGTTCTCCTTTGAACACATTCTTTTTTATTGGTTGAAATTAATGAATCTTTCTCCTGGTTATTAGTTTTCAATAGATTTCAATCAATAGAAAAGAGTGTGAGTATGATAGAGGATAGGTTTCCAACATTCCTGGTGAGGACAAACAATGGCATTGGGAGAAAAACCACTCTTCTATACTATGATAGGAAACTTTGGCACTTGGGATGTTTGGAATTAGACTAGGGTGGCATGTTGAAGTTCATATTTTGTTGAAATTGTACATGAGAGTCTTGGCTTGTGTATAGGAATGAGAGAACATACTTTCACTAAAGATGAATGCATTACTTTCCTTAGTTGTGCATTTTTTTTCTTGATCTTCCCAAGGAGGAAAACTTGTATTCTATTGATTGAAAACTTGATTGGTGTATTCTATTTGGGTATAATATTCCATTTTGCAGTTGAGTA

>PvRGL045

CCGTTGCTGTCGAATGAGACTCCTGATTGGCCTCCTTATGTAGCATTTCTAAACTGCAGTGATCCAGTGAGTGATGATCCTCGATACGTGGAAGTGAATGGCAGACTTTGTGATTCTGGAGGCCATGTCTATGCTGTTCTTTCTTCTTCTTCTAAATTCACAATGATGGACATCAAGTTATTTGTACAGACCAATGTGGAAAGGGTGCAAGATTTCTCATTTCATTCAGGCCACTCATTTCAATATTTCATGTGCTGCCAGATTCCTATTTGGAGTCGCAAGTTTTGGGCTTAGGACTATTTAACCATACGTGGCGACTGTAGGAATTCAACTCCAGATGTATTCAATAAGCAGTTGGAACATTCTAAACTATACTAGCACAACAATCTAGAATTCTTCATATTAGGTACAGAATACAGAGAAATTAAGAAAATGAGCAGAGGTTACAAAGTTAAATTGGGGGAAGGAGGTTACGGATGCTGTATACAAAGGAAAACTTCGAAGTGGGTCAGAAGTAGCAATAAAGATGTTGAAAAAATCGAAAGCTGATGGACAAGATTTTATGAATGAAGTGGCTAGCATTGGAAGAATACATCATGTGAATGTGGTACGTCTCATTGGATATTGTGCTGAAGGACAAAAGCGTGCTCTAGTTTATGAGTACATGCCAAATGGTTCATTGGATAAATACATCTTCTCCAAAGAGGAAAGTATCCCTTTAAGTTATGAGAAAACATATGAAATATCTCTTGGCATAGCTCGTGGAATTGCTTATCTTCATCAAGGCTGTGATGTGCAAATTCTACATTTTGATATTAAGCCTCATAATATTCTTCTAGATGATGATTTCATTCCAAAGGTTTCAGATTTTGGACTTGCAAAGCTATATCCTGTCAAAGATAAGTCAGTTGCTTTAACTATGGCTAGAGGAACTTTAGGTTACATGGCTCCAGAATTATTCTACAATAATATTGGTGGAGTATCCAATAAGGCCGATGTGTATAGTTTTGGAATGCTTTTGATGGAAATGGGAAGTAGGAGAAGGAACTCAAATCCTCATGCAGAACATTCAAGCCAACAGTACTTCCCCTTTTGGATATATGATCAAATTAAAGATGAAAAAGAGACTCACATAGAAGATGCCTCAGAAGAGGATAAGATTTTGGTGAAAAAAATGTATGTAGTTGCCCTTTGGTGTATACAATTGAAACCAAATAACCGTCCTTCAATGAAGAGAGTTGTGGAGATGCTTGAAGAAAAAGTTGAAAGTGTTGAAATGCCTCCTAAGCCTGTTTATTATCCCCCTGAAACAGTTGGACATGATTGTGGAGACAACTCAAACCAATCATCATGGAGTGGTTCAACTAGTTCTAGCAAAAATCTTGGCAAAACCAACACTAAATCATCCATGGGAGAGCAACACTTTGACGAACAAGAAAATTGCTTAGAGCTAAATGTTGGTCGTCCTTTAAAAGATTGATTTGGAAGGAGTTAGCTGTTAACAGGGAAAAGGTTTTTAATTTTAAAGTGGTTGCTATTATATATGTTGTTGCCTAGTATATAGAAAAGTTTCATTTAGTAATATAATTGAATAAATTGATTCTCGAAATTAAATGTACTAGAAACTTTTGGCAAAGGTTTGGGTTTCAAAGAACTATCAGTTGCACTTATGGATGAGTTAAGACCCTCAGATAAAGTAGTTTAATAGGACATGAGATAAAATCATGTCTATTGTAGCCAAATTTATATATGCATATACAAGTCTTGGGTTTTGGCATTTTGCA

>PvRGL046

GGTATCAAGAAACTCATAAATTTTAATGCAGTCACTTTTCCGATACTGGACCTCAACATCTGTTTAAGCAGAACACAAACCATAATTTACAACTTACAGTACTAACATATGTTTGTTTTACACGTACAAATTTCTGAGTTGAAGAATTACATTATAAATGAACAAGTGGCAAACTCTTGAATGGAAACAAAACAACACAACCAAAGGGGAAACAAACAATGATTAGTTACTTCAGCAGCTTGGTGAAGTAACTACTGTCTTTAAGCGGAGAACGATGGTGCACAGCTTGGTGGGCTTGACGAGTATTAGTAGATCGTGATTTAGGCTCCACAGGTTTCTCATTGGCAGCTTGAATTCGCTCCATACTCTCCAATACCTCCTTCATTGATGGTCGCTGTTTGGGCTCAGTTGCAAGACATTTCAAAGCAAGCTGAGCTATACGAAATGCAGCTTTTGATGGAAACTTTCCTTCCAATCGAGAATCCATAACGCCCTTCAACTTTCTTCTGTCATGCAGGTATGGTTTCACCCAGTCTGTTAGATTATGTTGCCCACTTGGACGGTTGCGATCAAGTGCTCGTAGCCCTGTTAGTATTTCCACCAAAACAACTCCAAACCCATATACATCACTCTTCACATATAGATGCCCTGTGGCCACATATTCAGGAGCTGCATAACCGAATGTGCCCATCACCCTTGTTGTGACATGAGATTGACTAGCTGAAGGACCCAACTTTGCCAAGCCAAAATCTGATATCTTTGCGTTATAGGACCCATCAAGCAATATATTTGAGGCCTTGAAGTCTCTGTAAATTACTTTCTGGGATGTATGAAGGAAAGCAAGCCCACGAGCTGCGCCAATTGCTATCTTAAGCCTGATATCCCAGGGAAGTGGTTGAACGGCAGAACCCCCAAGAAAGTGGTATTTATTTAAGCACTCACTTCCAAATAGATGGTTTTCCAAACTTCCTTTTTGCATAAATTCATAGACAAGAAGAAGCTCTGTTTCCTCCAAACAGTAGCCCAATAGCTTAACAAGATTAGGATGGGAAAGGCGTCCTAGAAAGTTTATTTCGGACCGCCACTCCTCCATTCCTTGTGAGCTTTCAGAGTTGAGTTTTTTGACGGCAATGACTGTTCCACTCTTGGAATTGCCCTTCTCTTCAAGCCAACCCTTGAAGACTTTTCCAAAACCTCCCTCTCCAAGAACTATATCAGCCCTGAAATTCCTAGTTGCAGCCTTCAAGTCAGCAAAGGAGAAAATCCTCAAATTGGAGGCGGGTAAGATCTGCCCAGTTGGATAAGGTTGGTCACCACTTGCCACCGAGAACTTGCTATTCATGGAGCTGTTCACACTTCCAGAAGTAGTAGTTGTGGTTGTGCTAGTGGTAAAGCTGGTGGTGCTGCTGCTGATGGTCTGAGATGTATCCGCCGTGGCTGTGCTAAGGTTACCGGTGGTTGTTGGGGTTACGCCGCCGTGAGGCTGAGAAGGCCAGCAGTTTCCCATTAGAACGGCGCAGAAAGAAGAATTCAGAGGCTCCTTCTCCGATCGATGATGGTGTTGTTGTTACTTCTTTTGGGTCCAATAATTGAAAATAGCTTTGGCAGAGCCTGACAGCAACGGGTGCGATGAAAAAAGCAAAAGTAATGAATTGTTAACTGTTATGGTATTGACAAGTTAAGTCACATAGACTTAGTCTCTCTCTCTTTCTCTCTCTCTATTTCTATCTAACTCTGCTCCATCCTTAGTCTTCAAGTCAACTATCGTGACAGGGAAAGTTTTTATTATATTGAGTCAAATCAACAAACACCCTCTGTTCAAACAAATCGCACACGCACACGCCTCTAT

>PvRGL047

AAGATGAAGAATCTCAACATTCTTGATCTCTCCAATAATAGACTGGAAGGACAAATCCCGCAGAGTCTTACTGGACTCTCCTTGCTCACTGAGATTGACTTGTCCAACAACATGCTTTCCGGGACTATTCCTGAGACTGGTCAATTTGATACTTTCCCTGCGGCGAGGTTTCAGAACAATTCTGGTCTCTGTGGTGTTCCTCTGTCTCCATGTGGGTCGGACCCCGCAAACAGTGCTAGTGCGCAGCATATGAAGTCTCACCGGGGTCAGGCTTCTCTGGCCGGGAGTGTGGCCATGGGATTGCTGTTCTCCCTCTTCTGCATCTTTGGTTTGATCATAATTGCCATTGAGACCAGAAAGAGGAGGAAGAAGAAAGAGGCTGCTCTTGAGGCCTACATGGATGGTAATTCGCATTCGGGTCCAGCCAATGTGAGCTGGAAGCACACCAGTACTCGGGAAGCGCTTAGCATAAACCTTGCAACTTTTGAGAAGCCTCTCCGAAAGCTTACTTTCGCGGACCTTCTTGATGCCACCAATGGCTTTCACAATGACAGTCTCATCGGCTCTGGCGGATTTGGTGATGTGTACAAGGCTCAGTTGAAGGACGGAAGTGTTGTTGCTATCAAGAAGCTGATTCATGTCAGCGGACAAGGGGACAGAGAATTCACTGCTGAAATGGAGACCATTGGAAAAATCAAGCACAGGAACCTTGTTCCCTTGCTGGGATACTGCAAGGTTGGGGAGGAGAGACTCTTGGTTTATGAGTATATGAAATACGGCAGCTTAGAGGATGTTYTGCATGATCAGAAGAAAGCGGGGATAAAGCTGAACTGGTCGATTAGGAGGAAAATTGCCATTGGAGCTGCTAGAGGATTGGCTTTTCTTCACCACAATTGTATCCCTCACATCATTCATAGAGATATGAAGTCAAGCAATGTGTTGCTTGATGAAAACCTAGAAGCGAGGGTGTCTGATTTTGGAATGGCTAGGCTTATGAGTGCTATGGATACACATTTGAGTGTGAGCACACTGGCGGGCACCCCAGGGTATGTTCCTCCTGAGTACTACCAGAGCTTCAGATGCTCCACTAAAGGTGATGTCTACAGTTACGGTGTGGTTTTGCTGGAGCTGCTGACTGGGAAAAGGCCTACCGACTCAGCTGATTTTGGTGACAATAATCTTGTTGGATGGGTTAAACAACATGCCAAGCTGAAAATAAGTGATATTTTTGATCAGGAGCTCATGAAGGAAGACCCCAATCTGGAGATGGAACTTTTGCAGCACCTGAAGATTGCGTGTTCCTGTTTGGATGATCGGCCATGGAGGCGTCCCACCATGATTCAAGTAATGGCAATGTTCAAGCAGATTCAGGTGGGATCTGGGATTGATTCTCAGTCAACCATAGCCACCGAAGAGGAAGGTTTCAATGCCGTTGAAATGGTGGAGATGAGCATTAAAGAAGCTCCAGAATTGAGCAAGCACTAGGCATTAAATTCCGGGAAGTGTCATGTGAATTCTTTGGGAAACAGACAAGAAGAGAGAAGAGGAGATGACGACAGATTCAACTCCCCTCAAAGTTTTTCCTTCTTCTTTGCCGCTTCAAATTATTTCAGACATAAGGGAAACGGTTAAAAGGGGGAATGAATGCTTTTGATGTATGTAGTCTTGTTATTTTATACATAAGAAAAAAGTTGTTTAACTTGTATATAAACAGCTTCAGTTGTTACCATCTGTGTTTTCCTAGAATTCTCAAAGTACTTTTTTTCATTCAGATATAA

>PvRGL048

CATACTTAGAAGTTCATTACACACTGGTGATTCAAATAGAAGTAGCAGAAAATACAATTTTATTGGAAAGAGAATTTCACTACTACTACACATATGCTACAAACCTACAACATACAACTTAATCCTCCTTGGGTTTATCACTCACACATATAACAGGAGATAATTCCATTCTAACTGCAAGACATTTTTCAGTTTATAACAGAGGTCACTGCCTCACAAAAATTTCACCTAATTGATCCCAAGACCAAGGCATATATAAGAGTATTTATCACCCCTTCTCAGCCTCAATTTTTGTGCTGCTTAGAAATGAGGAAAACTCAAAAAATGTCTATTCTTTGGAGCACCTTATTTAAGCATCAATGGTCACAGTCATTTGGTTTGGGGTGCATGGATCTGCCTCTTCCAGCATTTGAACCAACATTCTCATTGAAGGTCTTGAAGCTGGAATTTTGGAAGTGCATAGTAATGCAATTCTCAACACCTTCATGGCATCCTCTTTGAAATGCTTTGCAATAGTAGGATCCACCAATTCAAGAGCCTCTTCTTTGCTCCTAATAGTGCTGCATACCCAATGAACAATGTCATGATTCTCTCCAAACTCTGCCTCCATTGGCCTCTTCCCAGTTACTAACTCCATCAGAACTACCCCAAAGCTGTAAACATCACTCTTCTCTGTCACCTTACAAGTGTAGGCATACTCAGGAGGTATGTATCCAAGTGTTCCAGCAATGACATTGGTCCAATTGCCTGCTCCTCCATTGAGAATCTTGGCAAGGCCAAAATCAGCAATCCTTGGCTTCCATTCCTCATCCAGCAATATGTTACTGGATTTGACATCCCTATGTATAACTGGTCTATCACAGCCACGGTGCAGATACTCCAATCCTGTAGCAGCACCCAATGCAATGTCATACCTAACCTCCCACCCCATCTCAGACTTCTTATTACATGTGTGCAACCTGTCCCACAAGCTCCCTTTTGGTAAAAACTCATAAACAAGCAAACTGCTATCTTCACTTGTGATGCTACAATAGAGCTTCACAACATTTACATGCCTTATAGAGCTAAGAGTGGCCACCTCTGCATCAAATTCTGGTGACCTTGAACTCCTCCTAAGCATAGCTGAGGTGCTCCTGCAACTTCCCCTGTCACTTAGATTTGAGGTCCATATGTGTTTCACTGCAAATTCTGCCCCACCTTTAAGCACAACCCGATAAACACTCCCGGATCCTCCCTTGCCAATCAAATTCTCAGCCTTGATCCCGTGAACGATTTCGCTCTCATTGAAGTTTAGCACATGGTACTGCTTCACATCCCAGGAAGTAGTCTTCATTGGCTTCTCAAACTTGTTCTGCCTTAGTTTTGTGTAGAGGAAAAATGCTCCAAGCAGAACCATTACAGCAGCAACCAAACAGACTAAAAGGTTTCGGACTCTCCTGGAGCGTCTAGATTGCATTGAACATGGTCTGATACCTTTCAAAGTCTGGCTGCATAAGCCAGGGTTTCCCATGAACCCGTCTTTGAAGGCTGAAATACAGAGTAATTCTGGTATGG

>PvRGL049

CTTCTTTGTAGGAAAGATACAACTGATAGAGAACTTCTAGATATGAAAACGGGCTATTACAGCTTAAGACAAATTAAAGGAGCTACTAATAACTTTGACCCTGCAAATAAGATAGGTGAAGGAGGATTTGGGCCTGTATACAAGGGTGTGCTGTCAGATGGTGATGTGATTGCAGTTAAGCAGCTCTCCTCCAAATCAAAGCAGGGGAATCGAGAATTTGTCAATGAAATTGGAATGATATCTGCTTTGCAGCATCCAAATCTGGTGAAGCTATATGGTTGTTGCATTGAAGGAAACCAGTTGCTACTGATATATGAATACATGGAGAACAACAGTCTTGCTCGTGCACTTTTTGGTGAAGAAGAACAGAAGCTGCACTTAGACTGGCCTACAAGAATGAAGATCTGTGTGGGGATAGCAAGGGGACTGGCTTATCTTCACGAGGAATCAAGGTTGAAAATAGTACACAGAGACATTAAGGCAACCAATGTCTTACTTGACAGGGATCTGAATGCCAAGATCTCTGACTTTGGTTTAGCTAAGCTTGATGAAGAAGAAAATACTCATATCAGTACACGTATAGCTGGAACAATTGGTTACATGGCACCGGAATATGCTATGAGTGGTTACTTGACTGATAAAGCAGATGTATATAGCTTTGGAGTTGTAGCTTTAGAGATTGTTAGTGGAAAAAGCAACACAAAATACAGGCCAAAGGAAGAGTTTGTATATCTTCTAGACTGGGCTTATGTTCTCCAAGAGCAAGGAAACCTTCTGGAATTGGTGGATCCTAGTCTTGGTTCAAAGTACTCTCAAGAGGAAGCCATGAGAATGCTGAGCTTGGCACTCTTGTGCACCAATCCATCTCCCACTCTTAGACCAAACATGTCTTCTGTTGTGAGCATGCTTGAAGGAAAAATTCCAATCCAAGCACCTATAATCAAGCGCAGTGAGAGCAACCAAGATGTAAGATTTAAGGCTTTTGAGTTGCTATCACAAGACAGTCAAACTCATGTTTCATCTGCATATTCTCAAGAAAGTATGAAGCAAGGACACAGATCAGAAGATGGACCATGGATTGACTCCTCTGTGTATCTTCAAAGTGCAGATGATTCTTCAAACAGTAAACTAATTTAAGATTCAAACGGTGCTTGAATTTGTTAAAAGCTCACAGAGGCCTACTCTAAATAATTTCCAGTTCATTTTTGAAATAGATAAACCGAAAGTACACACTGCATGTACAATCTGTCATATATGAATATATAATGCTTCATATTATTAAATAGAGAAAGAATTTTAAGTAT

>PvRGL050

AGGAAAAAATGGTACCAAACACTTCAATGCCATCAAAACAATATTACAATTACAAATTAAAAATGTTACAATCAAGAGGGAAGAGTTACCCGAACGCAATAACAAGGAAGAAATAAAATGTTTATATTACAACAAAATTATTTATACAAATTAATCCAATTTGATTCCGAAGTCTCTAGCCTCTCTTCTGCAAGTAAGAAGAATCCAGGGTTAAAGGATAAAGATCTGCTGCAGAAGTAGATGAGGAAGTCCATGGCTCATCCTTTGAGATACTCAACTCTTGGTAGTATTGTCTCATTTTCTCTAACTTCTTCTCTTCCACATCACTTGCCTCTGAAAACACTTCCTCAACAAATGTTCTTCCTTCTAGAATGCTTACCACTGATGACATGCTTGGCCTAACTGTTGTAGTAACATTAGTGCATAAGAGAGCCACTTTTATCAACACTAACACCTCCTCTTTGTTGAAGTTTGAACCCAATCTTCTATCAACTAGGTCCATTAGATCTCCATTCTCTTTCAGCAGATGTGCCCATTCAAGAATGGAGAATGATTGTTCTTTAGGACGGTGAATGGTATTGTTCCTTCCATTTATGATTTCTAAGACAACAATTCCAAAACTATAGACATCTGCTTTATCAGTCAAATAACCATGCATGGCATATTCTGGAGCCATATATCCATATGTTCCAGCTATGCGAGTGCTAATATGTGTATTTTCTTCCTCATCCAGTTTGGCCAAACCAAAGTCAGATATCTTTGGATTGAGATCTTTATCAAGCAACACATTAGTGGTCTTAATGTCTCTATGAACAACCTTTAATCTTGATTCTTCATGGAGGTATGCCAAACCTCTAGCAATACCAATACAAATCTTGTATCTTGTTGGCCAATCCAATCTTATTTGGTGTTCTTCAGGTCCAAATAAAGCACGAGCAAGGCTATTGTTTTCCATATATTCATATACCAGTAATAGTTGATCTCCCTCCACACAACATCCATAAAGTTTAACAAGATAAGGATGTTGCAAAGCAGAAATCATGCCTATCTCATTTAAAAACTCACGATTTCCTTGTTTTGATTTAGAAGAAAGTTGCTTCACTGCTATCAGTGTTCCATCTGATAGACTTCCCTTGTACACAGGACCAAATCCTCCTTCTCCAATCTTATTTGCAACATCAAAGTTGTTTGTCGCTACTTTGATTTCTCTTAAAGGAAACACACCAGTTCGCAGGTCTAAACCCTTTAGCTCTCTTTCTAAAGAACTTTTCTTTCCTAGACATCCTTT

>PvRGL051

GCCAAATCCAGGACATGACAACAATGGAATGATGATGAAACCTTTAGGAGTAGATGTCCCAAAGAGGAAAAAAGAAGGGAGTAATGGAAGAATGATTGTTATAATTGTGCTGTCATCAGTTACTGCTTTTGTTGTATTCATTGGTCTTGCTTGGATTTGTGCACTGAAATGCTGTGCCTATGTTCATGTACACAAACCACTTCCCGATGGTTTAATTTCATCCTCTTCAAAACAATCAAGGGCTGCAAGTTCATTGGCTTACGGGGTCAGGTCAGGCTCTGGATTACAGTCCTTCAATTCTGGAACAATAACATATACAGGGTCTGCTAAGATTTTTACTTTGAATGACATAGAAAAAGCAACAAATAACTTTGATTCTTCGAGAATATTAGGAGAAGGTGGTTTTGGACTTGTTTATGAAGGTGTTCCGTAAATGCTGTGGAGGAATATAGCCGTGAAGGTTCTCAAAAGAGATGATCAGCGTGGTGGTCGTGAATTCTTGGCAGAAGTTGAGATGCTTAGCCGTCTACACCATCGAAATTTAGTTACATTGTTTGGTATATGCATAGAGAAACAGACTCGCTGCTTAGTTTATGAGCTTGTCCCTAATGGAAGTGTGGAATCCCACTTACATGGACCTGACAAGGAAACTGATCCACTGGATTGGACTGCGAGGATGAAGATTGCTCTTGGTGCAGCTCGGGGTTTGGCCTATCTGCATGAAGATTCAAATCCATGTGTCATACACCGGGATTTCAAGGCTAGCAACATCTTGTTGGAGTATGATTTTACACCCAAGGTCTCAGATTTTGGATTGGCTAGAACAGCACTGGATGAGAGAAACAAGCACATCTCCACACATGTTATGGGAACATTTGGATACTTAGCTCCTGAATATGCAATGACTGGCCATCTTCTGGTCAAGAGTGATGTTTACAGTTATGGAGTTGTACTCCTTGAGCTCCTAACTGGAAGAAAGCCCTTGGATTTGTCACAACCACCAGGTCAAGAAAATCTTGTGACGTGGGTTCGTCCACTTCTCACTAGTAAGGAGGGTTTGCAGATGATCATAGACCCAGTTGTAAAGCCTAGCATTTCTGTTGATATTGTAGTTAAAGTTGCAGCGATTGCATCCATGTGTGTGCAACCAGAAGTCTCACAACGTCCTTTCATGGGAGAAGTTGTTCAGGCCTTGAAGCTGGTGTGCAGTGAGGTTGAGGAAACAGACTATTTAAGATCAATGAGTTATCAAGAGGGTATTCTAACTGATGTGGAAGGAAAGTTTTCTGAAGCTTCAGGTGAAAGAGTGGAATTTTCGGAGTATCAAAAAACTCTTTCTGGCTATCAATCTGGTGAAGAAAAGGTAGAATTATCAGCATCAGAGTTTCTCAGTAATTCAGGCCAGGAATTTGAGTCATTCAGGAGGTATTCTCGATCAGGGCCTCTAACTATTGGGAAGAAAAGGCAGTTCTGGCAGAAGTTGCGAAGTTTGTCAAGTGGTAGTACCAGTGAACACGGATTTTCAACTAAACTATGGTCTGGATCCCATTAAGCTTTTCAACAATCTTGCATTTTGCTTACTGAATTCATGTTCCATGAATCACTTCTTAGATTCATTCTAAAGAAAATGTGGTGATAATATGTAAAACGTCACGAACTACAACTACTTATCAATGTATAAACTATGTACCCCTGTCGGTTTCTAGGTAGCTATGTCTAAAGTCTGAACAATCGGTAGTAGTACAGAGAATAGTAACAAGTTGTTCATGTCCATTATATTAGTTATCTGACAAAAAATGGACTAAATTTTTTAAAGGATTTAAGTGC

>PvRGL052

CGTGCTCAATTTGATGATGGAGAGGTTCTTGCAGTGAAGAAGATAGATTCATCTGTCCTTTCAATGATTTGTCAGATGATTTTATAGAAATAATTTCAAACATCTCCATTTTGCATCATCCAAATGTGACAGAGCTTGTAGGTTATTGCTCAGAGTATGGACAACACCTCTTGGTCTACGAATTTCATAAAAATGGATCACTGCATGACTTCCTTCACCTATCAGACGAATATAGTAAACCATTGATATGGAATTCCCGTGTCAAGATTGCTCTCGGTACTGCACGTGCTTTAGAGTACCTACATGAAGTTAGTTCTCCATCAGTTGTTCATAAGAATATTAAGTCAGCCAACATATTACTTGATGCAGAGCTTAATCCTCATCTTTCAGATAGTGGATTGGCAAGCTATATTCCAAACGCGGACCAGATATTGAGTCATAATGTTGGATCTGGATATGATGCACCTGAAGTTGCGTTGTCTGGTCAGTATACTTTGCAAAGTGATGTCTACAGCTTTGGGGTTGTCATGTTGGAACTTCTCAGTGGACGGAAGCCATTTGATAGCTCAAGGCCAAGATCTGAGCAGTCTTTGGTTCGATGGGCAACACCTCAACTCCATGATATTGATGCATTGAGTAAAATGGTTGATCCTGCATTGAAGGGGCTATATCCTGTTAAGTCCCTTTCTCGATTTGCCGATGTTATCGCCCTTTGCGTGCAGCCGGAGCCTGAATTCCGACCACCAATGTCAGAAGTGGTTCAAGCACTGGTGCGATTAGTGCAGCGAGCTAACATGAGCAAGCGAACATTTAGCAGTAGTGATCTTGGAGGATCCCAACGAGGAAGTGATGAAGCAGCTCTACGGGAGATCTAAATTCCCTTGTTTTACTACAATGGCTCCCAAGCAGATGTAGTTATATTATTCTCCCTTTTCTTTAAGTTAGACACCGGGTGATCTATGTCCTTTATTTCTCATATAATTTCTACAGTTACAAATTTTAAGCTCAGGTTAGATTGGTGCCTTCAATATTCTGTTAATAACAACTGCAAATAATGAAAGGTTT

>PvRGL053

GCCGCACGCACTGCAAGACCCTCAAGCGTGACGTTAAGGCTTTGAACGAGATCAAGGCTTCTCTTGGGTGGAGAGTGGTCTATGCGTGGGTCGGTGATGACCCTTGTGGTGATGGGGATCTTCCTCCTTGGTCTGGTGTCACGTGTTCCACTGTCGGTGATTATCGCGTCGTTACCGAGCTTGAGGTGTATGCGGTGTCGATTGTGGGACCTTTTCCTACTGCTGTTACCAGCTTGCTGGATCTTACACGGCTTGATCTTCACAATAACAAGTTGACAGGGCCTATTCCTCCTCAAATTGGACGGTTGAAGCGTCTTAAAATACTAAATTTGAGGTGGAACAAACTACAGGATGCAATTCCTCCAGAAATCGGTGAGCTTAAAAGTTTAACACATCTTTACCTAAGCTTCAACAATTTCAAGGGAGAAATTCCTAAGGAGCTAGCAAATCTGCCAGACCTTCGCTATCTTCATCTCCATGAAAACCGTTTAACTGGAAGAATACCACCAGAATTGGGCACTCTACAAAACCTTCGGCACTTGGATGCTGGTAACAATCATTTGGTGGGTACCATAAGGGAACTCATTCGTATTGAAGGTTGCTTTCCAGCACTACGCAATCTATATCTAAACAATAATTATTTTACCGGAGGAGTTCCTGCACAACTTGCTAACTTAACCAGTCTTGAAATATTGTACCTTTCCTTCAACAAAATGTCAGGAGTTATACCATCTAGCATTGCTCATATTCCTAAGTTGACATACTTGTACTTGGATCACAATCAGTTTTCAGGGAGAGTTCCCGATCCCTTTTACAAGCATCCGTTTTTGAAAGAAATGTACATCGAAGGAAATGTATTTCGGCCTGGTGTCAACCCCATTGGTTATCATAAAGTGCTTGAAGTTTCTGATTCAGACTTCCTTGTTTAATAAAAGTTCTAAATATTTATATTTTTTCAGGAACTGTACTTGTATAATTATTGGAAAAAAAATTGTTCCTCTAATTACCTCAATGAAATACCCACATAGGTAATTCAATTTTGTCTAATCTGTCTTGTCGTAATAAACAGTCGTTTTTAGAGTACTACTTGAGAGCTTTCCACGTTTCCAACAAGTTGTAATATATAGTCATGAACTCTTGTTATCAGCTTCCAAATTAAAGAATGATATTACATATTTTTTT

>PvRGL054

TAAAAATGAAGACTGTGAAGCAGACTTAGAGCTTCCTTTCTTTGATTTTGCTACAATAGTTCGTGCCACCAGCGATTTCTCAAGTGAAAATATGCTTGGCCAAGGTGGTTTTGGACCTGTGTATAGGGGAACACTCGCAAATGGACAAGAGATCGCTGCCAAGAGGCTTTGTAGTAATACTGGACAAGGAATGAAAGAATTCAAAAATGAAGTTATATTGTGTGCCAAACTTCAGCATCGGAATCTTGTAAAGCTTCTTGGTTGTTCCATCCAAGGAGAGGAAAAAGTTTTGGTCTATGAATACATGCCTAATAAAAGCTTAGACTACTTTCTCTTTGATTCTTCCAAAAGAGGACTCCTAAACTGGTCAAAGAGGTTGAACATTATACATGGCATTGCTCGGGGACTACTATATCTTCATCAAGATTCTAGATTAAGAATCATTCATAGAGATATGAAAGCAAGTAATATCCTGTTGGACAATGAGTTGAATCCAAAAATTTCAGATTTTGGTATAGCACGAATGTTTGGTGGAAATCAAACAGAAGGAAGTACAAAACAAGTTGTTGGAACATATGGATACATGGCACCAGAATATGCCATTAATGGACTGTTCTCTACCAAATCTGATGTATACAGCTTTGGAGTACTACTACTAGAGATAGTAAGTGGGAAGAAAAATGGAGGAATTCTGTTTCCAGATCATGGACTTAATTTATTAGGACATGCTTGGAGATTGTGGAAAGAGGAGACTCCTATGCAATTAGTTGATACTTCTTTGGGTGACACCTTTACCATATCAGAAGCATTACGGTGCATTCATGTTGGTCTTCTATGTGTGCAATTACATCCAGATAATAGGCCAAACATGGCATCAGTGATTTTTTTGTTGAGCAGTGAAAATATTTTGCCACAACCAAAGGAACCAGGTTTTCTTATTGGAAGGATGAAAACTGGTGGCGAATGTTCACCTAGCAATGCAATTAAGATTTCCACTACTGAATTAACTATAACCCAGCTGCAGGCTAGGTAGAGAAATTCTTTAGTATGGTTTGGTGTCATGTAAGAGCAGTTTTTTATTTACTCAATTGTCTCCATAAGTTAAAGTTGATTTTTTTATAGCAGTGTAGCGCATTATGTTTCCTACAAAACTTCATATATATACTTTGTTGAAATAATGGTTAAAGTAATGTACTACATACTTCCTTGACAAATTAAGGCTTCTTCCCTTACCATC

>PvRGL055

ACCAACTAACCAAGCATGTTTCAACTCAATCTTTCTTTACAAATCTATGAAATAACTAGTCTTCCATGTGCAAGAAGAGAACTAGTCATCAGAAGAAAAACAACAAAGATCATAACATAAGAGCACTGTGATGAGACATAATTTATAAGATAGGATAACTAGAGCCATCTATCTACCCTCAGACTTCACCAATTGTGAGAAGACCCTACTCATAGAAACACCAGACAGATCATCCAGACTCGTGCCTTCGAATTGTGCATTAGAAGCACTTGCATCCTGGCTGAAATTGTTGATCTGTGGAGAGAGTTCACCAATCATATTAGTGCTGTTTTCTTCAGGATCACCTTGAACCACAGCCTCTTGAAGTTGGAGAGCATACTCCAAATTCCACAAGACATCTCCCATAGAAGGTCTGTCAACACCATAGTCAGCCAAGCATTTCTCAGCAGTTTCTCCAAACTTCCTAAGAGAATCTGGCCTGATTTTGCCTGCAAGTGTTTGATCTATGATTTGCTCCAACTGTCCTTTCTTCTGCCATTTCATTGCCCATTCTGCCAAGTTTACCATTTCCCTAGGAAGTGTTGGATATGACAGGCCTTGCACAAAGAACTTCAAACAGGACTACCCCAAATGAATACACATCTGACTTTTCTGTTAGTTGTTGCCTCCTGAAATACTCTGGATCGAGGTACCCAAAACTACCTTTGACAGCTGTGCTCACATGTGTCTGGTCAATTTCAGGCCCTGCCTTTGATAGTCCAAAATCAGCGACTTTAGCCATTAGTTTCTCATCAAGTAGAATATTTGCAGACTTCACATCACGGTGAATAACAGCTTTAGCATAGCCAGTGTGAAGATAATGAAGTCCTCTGGCTGATCCAATGCATATCTCAAGCCTCTCCTTCAAGCTTAAGCTAGGCAGACCTGAGCCATATAAATGACTCTTGAGAGTTCCTTTCTCCATATATTCATATATCAAGATCATTTCATTCCTTTCATCACAATAACCAATCAAAGACACCAGATGGCGATGGCGGAACTGAGACAGCATTTCAATTTCAGTTCTGAATTCTGCAAGCCCCTGCTGGGACCGTGGATTCCCTCTCTTCACTGCTACTTTTGTGCCATCACTTAACTCTCCTTTGTACACTTTGCCAAAACCACCTATCCCAATAACCCAACTCTCATCAAAGTTGTTTGTAGCCTCCTGAACTGCAAGAAAAGGCACGCGGTACTCATAGTTTGAAGCAGCACTTAGTGTTGTGCCATTAGAATATTTACTTCCCATGGTATGAGAAGTAGTTCCATCATTGATGGATAAAGGAACCCATGTCTTTGACTTCCTTTGCCGAGCCAACTTTCGTCTTCTCATGCATAATACAAAGAAAACTCCAGCCAAGACAACCGCAGCAACTACCCCAACAATCACACCCACAATCATGCCAATGCTCTTAGAACTTGAACCTGAACTAATAGCCACAGGTCCTGCTCCTGTGATAAGACTGCCCATGGAATTGTTCATTTTCATGATCTCAAGCCCATTCAAAATAGCATTAGGGAAATCCTTACTTAAATCAGAAGGACCAATACTTACACGAAGTTTATTGCTGACAGCCAACGGTGTGACCAAATTCTTATGATATGGAACACCCAAAACATTATTACCATGAAGACTGAGATCA

>PvRGL056

AAGCTTTCTCTAACTTCACTTTCCCACCCAAAACCAAAACCAATGCCTCCAATTTTGTAAGCACACCCATTTCTTTGCAACCATGTCAGTGTACGACGCAGCTTTCGTCGACACGGAGCTTTCCAAACGGACTTCCATCTTCGGCCTCCGTCTCTGGGTCGTCATCGGCATCCTCGTCGGATCCCTCATCGTCATAGTCCTTTTTCTCCTGTCCCTCTGCCTCACCTCCCGCCGCCGCCACCACCACCACCACCACAAAACTCCCCTCCGCCGCAGCGCCGCCGCCGTCTCCACGCCGCCCATCTCCAAGGAAATTCTGGAGATCGTTCACGTTCCCATGCCGCCAGAAGCCGTCAACCTCCCGCCTCCGAAGGCGGAGCCCCGCGCCGCCGCCGTACTGTACTCCAGTGGGGAGAGTAGGGGAACTGGGAGCTTGTGCGAAACGGCGTCGTCACTGGGGAGCGGGAGTGTGGGCCCCGAAGTGTCGCATCTGGGGTGGGGGAGGTGGTACACGCTGAGGGAACTCGAAGCTGCGACTAATGGGTTGTGTGAAGAGAACGTGATTGGTGAAGGTGGCTATGGAATCGTGTATCGTGGGTTGTTACCTGATGGAAACAAGGTTGCTGTTAAGAACCTTTTGAATAATAAGGGCCAAGCTGAGAGAGAATTTAAAGTAGAGGTAGAAGTAATTGGTCGTGTGCGGCATAAGAATCTTGTAAGGTTGCTTGGATACTGTGTTGAGGGGGCGTACAGGATGCTCGTATATGAATATGTGACAAATGGGAATCTAGAACAGTGGCTGCACGGGGATGTTGGAACTGTAAGCCCTATAACTTGGGATATCCGAATGAACATTATACTGGGCACGGCAAAAGGACTGGCCTATCTTCATGAGGGTCTTGAACCCAAAGTTGTCCACCGAGACGTTAAATCGAGCAACATACTGCTTGATCGCCAATGGAACCCAAAGGTTTCTGATTTTGGGCTTGCGAAGCTTTTGTCTGCTGATCATAGTTATGTCACTACTCGAGTGATGGGCACCTTTGGTTATGTTGCACCTGAGTATGCCTGCACTGGAATGCTGACTGAGAAGAGTGATGTGTATAGTTTTGGGATACTTATCATGGAAATAATTACGGGAAGAAGTCCTGTTGATTATAGTAGACCACAAGGAGAGGTTAATTTAATAGAGTGGCTGAAAAGTATGGTTGGGAATCGAAAATCTGAGGAAGTAGTTGATCCTAAGCTTGCTGAAAAGCCATCTTCAAGGGCTCTTAAACGGGTTCTTTTGGTTGCTCTTCGATGTGTCGACCCTGATGCTGCAAAGAGGCCTAAAATAGGACATGTTATCCACATGCTCGAGGCTGAGGACCTCTTATTCCGTGATGAACGAAGGACTGAGGGAGAATCTTCCCGTTCTCATCGTGATTATCAACTGGAGCATAAAGGGTCTGGATTAGATAAAAGGCAGACAGGTGGAGAGATCAACAATCAGATTGAAGATGACAGTACTAGTAGTAGTAGATATCACCATCAGCCTTCTAGATGGAGATAATCGTAGCATGATAACTGAAAAGCAATGTGCCATATTATTTTTCTTCCTAGTAGTTTGCTAATATTTATTTTTACTTTAATGATGAAAGAAGCAATTAGTCCCCTATTTGCATAATGTCCCCAGTTACCTACCTCAATCTGGGGTGTTCTTTTGTATACTCTGATTCATTGCTATAAAATTCAAAATTATTTTGTGTTTCAAAAA

>PvRGL057

ACACCATGATTGATGATCTTAAATTGATTTTTCAATATCAATAGCACACAACTGAATCTAGATCCTCAGAACTACATACACCACTAACATTTATTTTAGGACAACAAAGTAAGAGATATATAATTACATGATAAAATATAATACCAATTACAAATACTATACTATACCCTATATATCTCACATACCTTTCAATAACCTCATTCTCGAGCTACAATAAGTTCCTCCAAGTTATCAGCATCGCCATTATCAGTAGGTACCAAGATCTCATCATCAGCTGTGAAATTGTATCTAGCCCCAATAGCTCTTAAAAACTGATACACTTCAAACATGGTTGGCCTCTCCTTTGGAGTTGGCAGAACACAGTTGCATGCAACCTTCAGAAATTGGAAAAGCTCACTGTCAACATCCTTGTTGACTAATGATTCATCAATGGCATCATGGAGTTTTGCATTGTTTGTTAGCTCCGTAATCCATTCTACCAGATTTCCTTTAAAAGTTTCTTGAGCTTTAGCCACATTGGTAGGTCTTTCACCAGTCACCAACTCAAGGAGAACAGTCCCAAAGCTATAAACATCCCCCTTGGGTGTGGCAATCAAAGTTCTTGCGTACTCAGGAGCAACATAACCCAAATCACCAAACTCCCCATTTACAAAAGTACTCAAATGGGTATCAATTGGGTTCATGAGTCTGGCAAGGCCGAAATCAGAAATTTTTGGCTCAAAATCTGCGTCCAACAAGATGCACTTTGAGCTTATGTTTCGGTGGATAATACGGGGATTGCAGCTATGATGAAGCCATGCGAACCCTTTGGCTGCTCCAACTGCAATTTTGAGTCTTGTAGTCCAGTCAAGGGTGCTCACACCATCGGCGGGATGGAGTTGGTCATGGAGATTGCCATTTGGCATGTTTTTATAGACCAAAAGCCTCTCCTTTTTGGCCATGCAAAAGCCTAAAAGAGGAACAAGGTTGCGATGCTTGACAGTCCCTAGTGTACCCATCTCAGACATGAATTCCTTTTCAGGATACTGAGATTCCTGTAATCTCTTAACCATAAGTGTTGTGCCATCTCCAATGACAGCTTTGTAAACAGTTCCTGTTCTTCCTGACCCAATGATATTGGTATTACTGAAGTTGTTAGTAGCCTTCATGAGATCAACCAACTTCATTTTTGAGATTGATTTCTCAAACATAGAAACCTTAATTTGTTTAGTTCCTTTTAGACTTCTTGCCCATTTGTTTCCTTCGGGGTCCTCTTCCTTCTTGAAAGAAACACGTCGAACAAAAAAGAACATTCCAATAGCCAATCCTAAAGCCGCAAGAGTCACCCCACCGGCGGCAGCTCCAGCAATAACAGCCATGTTACTCTTAGAATTCTTGGTTTGGCAAGTCCCCAATCCATTACCTCCACATAGGCCTTGATTATTTGCATAATTTACCGAAACTGAATGGCCAAAGCTTGG

>PvRGL058

AAGAAAAACATTAAAAGCAGTGGTGGCATACTGAAAGGAAAGGCTTCATGCGCTGGAAAGACAGAGGTTTCAAAGAGTTTCGGGAGTGGGGTGCAAGGGGCTGAGAAAAACAAACTATTTTTCTTTGAAGGTTCCTCTTCTAGCTTTGACCTTGAGGATTTGCTGAAGGCTTCAGCTGAAGTTCTTGGAAAAGGAAGTTATGGAACAGCATACAAGGCTGTTTTGGAGGAGGGAACAACAGTGGTAGTCAAAAGGTTGAAAGAAGTTGTGGTTGGAAAGAAGGAGTTTGAGCAGCATTTGGAGATTGTGGGGAGATTTGGAAGCCACCCAAATGTGATGCCCCTAAGAGCTTATTACTATTCCAAAGATGAAAAACTTATGGTTTACAATTACATGCCAGGTGGAAGCTTGTTTTTCTTGTTGCACGGAAACAAGGGTGCAGGAAGAACCCCACTAGACTGGGATTCCAGAGTGAAGATTGTGCATGGAGCTGCGAAGGGAATTGCTTTCATCCACTCCGAGGGAGGTCCAAAATTCACACATGGCAACATCAAGTCAAACAATGTGCTCATAAGCCAAGAACTAGAAGGCTGCATCTCTGATGTTGGATTGCCACCTCTAATGAACACCCCAGCAACCATGTCTAGAAGCAATGGCTATAGAGCTCCAGAAGTCACTGACTCAAAGAAGATCACTCAGAAGTCTGATGTATACAGCTTTGGTGTGGTGCTGCTTGAAATGCTGACAGGGAAGACCCCTCTGAGGTATCCAGGGTATGAGGATGTGGTTGATCTTCCAAGGTGGGTGAGATCTGTTGTTAGGGAGGAATGGACTGCAGAAGTGTTTGATGAGGAGCTTCTGAGAGGGCAATATGTTGAAGAAGAAATGGTGCAGATGCTTCAGATTGCATTAGCATGTGTGGCTAAGGGGTCAGAAAATAGGCCTAGAATGGATGAAGTTGCTAGAATGATTGAGGAAATTAAGCATCCTGAATTGAAGAACAGGCCATCCTCTGAGTCTGAGTCAAATGTGCACACACCATGATGAGTCTTTTCATCTTTTCCTGCCAACCGTGTTTGAGAGGGAGAGTAATAAAATCTCAAGAAAATGGGGTATTCTCATCTTTTGTTTTGTCCTCAATTAATAAGCATGTTTCATTGTAAAATACTACTCATGTTTTTATTCATGTTTCACATTATTTGTTATGAAAGATTTGGATATTTATTTAGTAAAGGGTGTTAAACATATAATTTTCATTATTTATGCATTTAGTTTGGTA

>PvRGL059

CACAACAACAACAACACAAAACTCCCAAAATGGTTGATTTACATTGGAAATCAAAGATGCCCGCTTCCGACATGTCTTCCAAATCTCCAAAACTCTCGCTCTCCGCCAACTCCTTACCCTCTTTGCACCTTCCCTTCCGCACCACAGACATATCTCCCGCTGCACCTTCACTCTGTGCTGCATACGATTACTATCTCTCTCTTCCACAACTCAGAGCCCTTTGGAATTCAATAGACTTTCCCAATTGGACCAACGAACCAATTTTGAAACCTGCCTTGCATGCTTTGGAAGTCACTTTCCGTTTTCTTTCCATTGCTTTCTCCGATACCAGGCCTCATTCCAACCGAAGAGAATGGAAACGTACCATAGAGTCCCTCGCTGTTCATCAGATAGAGATCATAGCCATGCTGTGCGAAGACGAGGAGTACAATTCTCAGACACGTGGCGCCGTCCCAACCGCTGATCTCACTCTAGATACCACCAACAGAAGCTACAGCGAGGCCAGTCTTCTTCCGCGGCTTGCCACGTGGTACAAATCCAAGGACGTGGCGCAGAGGATTCTTCTCACCGTGGAATGCCAAATGAGGAGGTGTAGCTACACCCTAGGGTTGGGAGAGTCGAACCAGGCGGGGAAACCGAGCCTGCTCTACGATCGCGTCTGCAAACCAAATGAAATCCACGCGCTCAAGACCACGCCTTACGACGAGCGCGTGGAGAACCACGAGAACCACGCGGTGCATGCCACGCTCCAGATCGCGGAGTCCTGGATCCACGCGTCGCGGAAGCTTCTTGAGAGGATCGGCGATGCTATCGTCTCCAGGAGGTTGGAGCAGGCGGCGCAGGACTGCCACGCGGTGGAGCGCATCTGGAAGCTTCTCGCGGAGGTGGAGGATCTCCACCTGATGATGGATCCGGACGAATTCTTGCGTTTGAAGAACCAGCTCTCGGTGCGCTCGTCGAGCGGCGAAACGGCGTCTTTCTGCTTCAGATCGAAGGAATTGGTGGAACTGACGAAAATGTGCAGAGATCTGAGGCACAGGGTGCCGGAGATATTGGAGGTGGAGGTGGATCCGAAGGGAGGACCGAGGATTCAAGAAGCGGCGATGAAACTGTACGTCTCGAAGATCGCGTTCGAGAAGGTTCACGTGTTGCAGGCGATGCAGGCGATTGAAGCGGCGATGAAGAGGTTCTTCTACGCGTATAAGCAAGTTTTGACGGTGGTGATGGGGAGTTCGGAGGCTAACGGCAACCGAGTTGGACTGAGTTGCGAGTCGGGTGACTCGTTGACTCAGTTATTCCTTGAACCGACGTACTTTCCAAGCTTGGATGCCGCCAAGACTTTCCTTGGTTACTTCTGGGATAATAGCGATAACAAGTGTTAGATCTCATCACCAACACACTTTCATCTTCGTGTAATTCACAAAAACTAAATTTGTAATTTAGAAATTCTTTTTTTCTTTTTAAAGAAAATTATATTTTGTAACGGGCTTCGTCGGTCTAAATTATATACCAAATTATTTTTCGACAATGAAACTCTTTGAAGCTCCCTTCTGTTGAAGCTTTAA

>PvRGL060

GTAGGGTTGAGAATTGTGGCAGGGCCTAATGGGTAACTGCCTAGATTCTTCTGCCAAAGTTGATGCAGCTCAGAGTTCCAGATCCACTTCTGGAATCTCTAAAGCTACACCTTCCAGTTTATCTATTCCATCATACAGTGAAAAAAGCAACGCCTTAAGTCTTCCCACACCAAGGTCTGAGGGTGAAATCTTGTCTTCTCCCAATCTCAAGTCCTTCACATTCAATGAGCTGAAGAATGCCACCAGAAATTTTCGGCCTGATAGTCTTCTCGGTGAAGGGGGATTTGGGTGCGTCTACAAAGGATGGATTGATGAACACACATTTACAGCTTCAAAGCCTGGATCAGGAATGGTTGTTGCTGTAAAGAAGCTCAAACCTGAAGGTTTTCAAGGTCATAAAGAGTGGTTGACTGAGGTTAACTACCTTGGACAACTGTACCATCCTAATTTGGTGAAATTAATTGGTTACTGCTTGGAGGGAGAGAATCGGCTATTGGTCTATGAGTTTATGCTAAAAGGGAGCTTGGAGAACCATCTGTTTAGAAGAGGACCCCAACCACTCTCTTGGTCAGTGAGGATGAAAGTGGCTATTGGTGCTGCTAGAGGACTCTCTTTTCTTCATAACGCCAATTCACAAGTCATATACCGTGATTTTAAAGCCTCTAATATCCTACTTGATGCGGAGTTCAATGCTAAACTTTCTGACTTTGGTTTAGCTAAGGCGGGTCCTACTGGTGATAAGAACTCACGTCTCTACTCATAGTCATGGGTACTCAAGGATATGGACTAGCATCCTGAATATGATTGCAACTGGTAGGTTGACAGCCAAAAGTGATGTCTACAGCTTTGGAGTTGTGTTGCTTGAACTTTTATCTGGAAGACGTGCAGTTGATAAAACAATATCTGGTATGGAGCAGAATCTGGTGGACTGGGCAAAGCCATATTTGAGCGACAAACGAAGACTGTTCCGGATCATGGATACCAAGTTGGAGGGGCAGTACCCGCAAAAGGGTGCCTTCATGGCTGCCACACTTTCTCTACAATGCCTCAACACTGAGGCCAAGACAAGGCCTCCAATGACAGAGGTTTTAGCCACTCTGGAACAGATAGAAGCCCCCAAAACTACAACCAGAAACTCTCATTCTTCAGAACACCATAGAGTTCACACTCCTGTGAGAAAATCTCCTGCCCGAAATCGGTCACCTCTAAATCTAACTCCTACCGCATCCCCTCTTCATGCTCACCGGCAATCGCCTCGTGTGCACTGAGATTTGAACATAAAACATATCTGTATGTAAATTCCTTGTTTCTATCAGGTTGTGTTTTGGACTGCATGCGTTGAGGCATGTGTACTTACATGCAATTTTGTCATGCTCAGTAGAATGAATAGTTAATACCATTTATCCAAATGCTATGCTGCAAGTTATTGCAGCTGTGTTCTGT

>PvRGL061

GCGGACTACTATAGCATGTGGAAATCATGCGCTAATATTTAATTGAAATTTCAATTGAAGTACATGGCACAATATACCCTGTCAAAAGCAACCTGATAAAAATAAGGAAGGTATGGCACCATATATCCTGTCAAAACCAACTTGATAAAATAAGGAATGTATATACAGATGTTCAAAATTGTTGCACACAAGCAGATAATGGCTGGGATGGCAATGAATGAAGTAGGAATATGGTTCAAAGGGCACCCATTTTCACTTTAGACTTTCTTACAGGAGCATGGACTCTCTTTTGTTCTGTTTGGGAGTTTCTCCCAGCAGTTTTTGAGGCTGCAATCTGTTCAAGAGTTTCTAAAACCTCTGTCATTGGAGGCCTTGCCTTGGCTTCGCGATTTAGGCATTGTAGTGCAAGAGTGGCAGCCATGTAGGCTCCTTTTTGTGGGTATTGGCCTCCCAACTTGGTATCCATAATCCGAAATAGTTTTCTTTTGTCACCCAAATATGGTTTTGTCCACTCTACTAGATTCTGCTCCACACCAGCTTTTGTTCTGTCAACGGCACGTCTACCAGACAATAGCTCAAGCAACACAACACCAAAGCTGTATACATCACTTTTAGCTGTCAGCCGACCTGTTGCAACATATTCAGGTGCTGCATATCCTTGGGTTCCCATAACTTGAGTAGAGACATGAGTTCTATCACCCGTAGGGCCAGCCTTTGCTAAGCCAAAATCAGAGAGCTTAGGATTGAAATCCGCGTCAAGTAGGATATTAGATGCTTTAAAATCACGGTATATGACTTGAGACTGGGCATTATGAAGAAAAGAGAGCCCTCTGGCAGCACCAATAGCCACTTTCATTCTCACTGACCAAGATAGAGGCTGTGGTCCTCTTCTAAATAGATGATTCTCTAAGCTCCCTTTGGACATAAACTCATAGACCAACAACCGGTTCTCTCCCTCCACGCAGTATCCAATCAATTTAACCAAATTCTGATGGTGAAGTTGCCCAAGGTAGTCCACTTCAGTCAACCACTCTTTATGACCTTGTAACCCTTCAGGCTTGAGTTTCTTAACAGCAACTACCATTCCTGATCCAGGTTTTGAAGCTGTAAATGTGTGTTCATCAATCCACCCCTTGTAAACATAACCAAAGCCTCCTTCCCCAAGAAGACTGTCGGGACGAAAGTTTCTGGTGGCATTCCTTAGCTCATTAAAGGCAAAGGCCTTAAGATTAGGGGAAGACAAAATTTCACCCTCGGACCTTGGAGTGGGAAGATTTGAGACATCACTTGCTTCACAGTATGACTGAATAGATAAGTTCGAAGGAACAGATGAAGGACTAGTCTTTGAAATTCCAGAAGGGGTTCTGGAACTGTGAGCTGCTTCTACTTTAGCCGAAGAACCTATGCAGTTACCCATTTTCC

>PvRGL062

GGCAACACAATGTTCCCCCTCCGCAGGATCATGTGGTTTCAATGATGCCTCCAAAGTCGTCGCTTCCACCGGCTCCGGCTTATGCTGCTCAAACTGCTCCTCCCCCGCCTCCTTTGTTCACCAGCAGTGGTGGGTCTGGATCAAACTATTCAGGTGGTGAACCACTTCCTCCTCCTTCTCCAGGGCTTTCATTGGGGTTCTCTAAGAGCACTTTTACCTACGAGGAGTTGGCACGCGCCACAGATGGATTCTCTGATGCGAACCTCCTTGGACAAGGTGGATTTGGATATGTTCACAGAGGAATTCTTCCCAATGGCAAGGAGGTGGCAGTGAAGCAATTGAAGGCTGGAAGTGGGCAAGGGGAGCGTGAGTTCCAGGCTGAAGTTGAGATAATTAGCCGTGTTCATCACAAGCATCTTGTATCTTTGGTTGGATACTGCATCACTGGGTCCCAGAGGTTGCTTGTTTATGAATTTGTTCCAAACAACACATTGGAGTTCCATTTGCATGGAAGAGGACGACCTACCATGGATTGGCCCACAAGACTAAGAATTGCCTTAGGATCTGCTAAGGGACTGGCATATCTTCATGAAGATTGTCATCCTAAGATCATCCATCGTGATATCAAGTCTGCCAACATTCTTCTGGATTTTAAGTTTGAAGCCAAGGTTGCAGATTTCGGTCTTGCTAAGTTTTCTTCTGATCTTAATACTCAGGTTTCTACACAATTGATGGGGACTTTTGGGTATCTGGCTCCAGAATATGCTTCTAGTGGAAAACTCACAGACAAATCAGATGTTTTCTCCTATGGAGTCATGCTCCTTGAGTTAATAACTGGACGGAGACCAGTTGATAAAAATCAAACATTCATGGAGGATAGTTTGGTAGAATGGGCTAGACCTTTGCTCACACGAGCTTTGGAAGAGGATGATTTTGACTCTATTATTGACCCAAGGCTTCAGAATGATTATGACCCTAATGAGATGGCACGAATGGTGGCATGTGCTGCAGCTTCCACACGTCATTCGGCAAAGCGCAGACCAAGAATGAGCCAGGTTGTGCGTGCTCTGGAAGGAGATGTGTCTCTATCAGATCTTAACGAAGGAGTCAAACCTGGACACAGCACTATGTACAGTTCTCATGAAAGCTCAGATTATGATACTGTACAGTACAGGGAAGACATGAAAAGTTCAGAAAAATGGCATTGGGAACTCAGGAATATGGTGCAAGCAGCGAATACAGTGCAGCTACAAGTGAGTATGGTTTAAACCCATCTGGCTCAAGCAGTGAAGCTCAGAGCCGCCAAACCACAAGGGAAATGGAAATGAGAAAGATGATGAAGAACAGTCAAGGTTTCAGTGGAAGCTCTTGATCCTTAGTAACTGTTTATGCCTTTATCTTTTGTATAAATCTATATTCATTTCTTGCCAATGTCACAGTTGATTGCTTTACTGACATCTTTGGAGAGACTTACCAATTACTATAATTTTTTTTCTTTACCAAATTGATCATCTTTTGAATTGTATTTATCAGGGTATGTATACAAAAAAAAAAATAACACAGGTCCATTCTTTATTTTCTTTTTCATTTTTTCGGCTATTCAGCTATAGTGTTGGAAATGGGTTGCTGCATCAAACCTTGTTTTACA

>PvRGL063

CTACTATTTCAATTCAGGTTCTTCGGCAAGTTACGGATAATTTCAGTCAGAATAACATTTTAGGTAGGGGAGGATTCGGGGTTGTTTATAAAGGGGAATTGCATGACGGAAGTAAAATTGCTGTGAAGAGAATGGAATCTGTTGCAACGGGGAGTAAGGGATTGAATGAGTTCCAAGCAGAGATTGCAGTTCTCAGTAAAGTCAGACACAGACATTTGGTTGCTCTTTTAGGATTTTGCATCAACGGCAACGAAAGGCTTTTGGTGTACGAGTATATGCCTCAAGGGACGTTAACACAACACCTGTTTGATTGGCAAGAAAATGGATGTGCTCCATTGACTTGGAAACAAAGGGTAGCAATAGCTTTGGATGTAGCTCGGGGAGTGGAATACCTGCACAGCTTAGCTCAGCAAAGCTTCATTCATAGGGACTTGAAACCCTCGAACATATTATTGGGTGATGACATGAGAGCCAAGGTTGCAGATTTTGGTTTGGTAAAAAATGCACCAGATGGAAAATATTCTGTCGAGACAAGGTTGGCTGGAACATTTGGATATCTGGCACCTGAATATGCAGCTACTGGAAGAGTGACCACCAAAGTAGATGTGTACGCATTTGGAGTAGTTTTGATGGAACTGATCACCGGTAGAAGGGCATTGGACGATACTGTGCCTGATGAAAGGTCTCACTTGGTTTCCTGGTTCCGTAGGGTACTAATTAACAAGGAGAACATCCCAAAGGCAATTGATCAAACCCTGGATCCGGATGAGGAAACCATGGAGAGCATATATAAAGTGGCTGAACTGGCAGGTCATTGCACTGCTCGTGAACCCTACCAAAGGCCAGATATGGGTCATGCAGTGAATGTCTTGGTTCCTCTGGTAGAGCAATGGAAGCCTACCAGCCATGAAGAAGAAGAAGGTTATGGCATTGACGTTCACATGAGCCTTCCTCAAGCTCTGCAAAGATGGCAAGCTAATGAAGGCACTTCCACAATGTTTGACATGTCCACCTCACAAACTCAATCAAGCATTCCTGCAAAACCTTCAGGATTTGCAGACTCTTTTGATTCCATGGATTGCCGATGACAAAAATGACAAACAGATCGCTGATGAGAACGATGTTAAGCTATTTTGATCTCGCAACATCACCGAATTGTATCACAATTTATTTATTTATTTTTTAAATGTTAATTTTGTTAATGTTCCTGCTGCATTCTCCTTCTGCCCCAGTTTGATGTTTTTTATTGATTTTTTTCAAAGCTAACTGATGGATTAGTCGATGGTTGGTTTCTTAGATGAGAATTATTATAGGAATGTTAATTGTATGATATTTTTCATTATTTTTTGAATCAGAAGAATATTTATTGCA

>PvRGL064

GAAATCATTATCAATATCTTTTACATTATGTGACAGAGAAGCTTGCATACAAAACAAGAATAATTTTGAAATTAGCTTCAGCTTTTCACCAAAACATGAATTCCTTCCTTCAAAACCCTTGTTCTGTTGTGCACGTGATCTCAAACTGCATCAACATCACTTCTTTTTCTTTTTGTCCCTTTCATCTTTCTCACTACAACATTATATTACACCACTTAAAAAACAAACACCGAAACATTTTACTATTACACCAAAATTCACACTTGTATCCATAATCGAATCGGGTACTTAATCGGGTCACCCGCAATAACTAGCAAGTCTTCAACGGGTTCCCACACAAGCAATCGTTGAACCCAAACGACGACGCTTCAAGGTGATCAAAGGGTTCTCCCACAGGAATGGATCCGCACAGATGGTTGTGACTGAGATCCAAATGCCCGATATATTTGGCGGATGCCAAGGATTCGGGTACCCGACCCTTTAAATTGTTGTAAGACAAATCCAAAGCCATGAAATAGGAGTGTGACCCGAAAACATCGGGTATGGATCCTTCGAACCCGTTTCGGCTAAGGTTCAAAATACCCATTCCGCCGTTGCTCAACAAACTCTCGGGGATTAAACCCTTGAGAGAGTTGCTATCCAGATTAAGCACGGAAAGAACCGGCATTTTGCCAAGCTCAAACGGAACCGAACCGGTCAAGCGGTTCACGGACAGGTCCAAGTCCGCGAGCCGGTAAATCTTGGAAATCGAAACCGGTATTGAACCGGTTAACTGGTTCCGGTTCAGCATTGCTCGGCTCAGCATTGCAAGGTTCCCGAAATTCTTGGGAATCTCGCCGGCGAGAAGGTTGCTGCTGAGGTCGAGGTGTTTGAGTGAGGTGAGTTGGGTGATTGAAGGGGGAATTTTCCCGGTGAGGGCGTTGTCGGCGAGGTTGAGGACGGTGAGGCGGTTCAGTCTGCCGATGTCGCCGGGGATCTCGCCGGAGAGTTTGTTTCCGATGAGGTCCAGGATGCGGAGGGAGGAGAGGCCGGTGATGCAGGCGGGGATATCGCCGGCGATGTCCTTCCAGTCAGCGACGACGAAGGTGGTGAGGGTGTCGATGCCGCAAATTGCCGGCGAGAGTTTTCCGGTCATGTAGCCGGAGCGGCCGGATTTCTCGAAGATAGGGTCCTCCGACTCGCCGCGGAGGTTGACATCGGTGACGCGGCCGGTGGCGGCGTCGCAGCTGACACCGTACCAGTTGACGCAGCAGTTGGAGCCAGTCCACGAGTTGAAGAGGCCGAGGTAGGGCTCGTTCAACGCCTGCCGGAAGCCAAGAAGTGCCTCCCTGTCCGACGGCGAGCAACCTGTGGCGACGGCGACTGCGGCGGCGATAATGGCCGCTGCTAACAGGAGGAACGAGTGGCGTGCCATTGAGTG

>PvRGL065

AGAAGGCTTCTGCAAAGGAAGGAAAAAATGTAGATGACATCACCACTTTGGAATCCTTGCAATTCGGTTTCGGCACGATTGAAGCCGCCACAAACAATTTCTCAGCGGACAACAAGCTGGGGGAGGGTGGATTTGGTGAGGTTTTCAAGGGTACTCTTCCGAGTGGACAAGTAATAGCTGTGAAGAGACTCTCGAAAAGCTCTGGGCAAGGTGGAGAAGAATTTAAGAATGAAGTGGTGGTGGTTGCCAAGCTCCAACATAGAAATTTAGTGAGGCTTCTCGGGTTTTGCTTGCAAGGAGAAGAGAAGATACTTGTCTATGAATATGTTCCCAACAAAAGTCTCGACTACACTCTCTTTGACCCTGAAAAGCAAAGTGAGTTGGATTGGAGGAGACGTTACAAGATTATTCAAGGGACTGCTCGAGGCATTCAATATCTTCACGAAGATTCTAGACTTAGAATTATACATCGAGATCTTAAAGCAAGTAATATATTGTTAGATGGGGATATGAGTCCAAAAATCTCAGATTTTGGCATGGCAAGAATATTTGGAGTTGATCAAACTCAAGGAAACACCGACAGAATTGTAGGGACTTATGGTTATATGGCTCCAGAATATGCAATGCATGGAGAGTTCTCTGTTAAGTCTGACGTGTACAGTTTTGGAGTCCTCCTTATGGAGATCATAAGCGGCAAGAAGAATAGTTCTTTCTATCAAACAGATGGTGCTGAGGATCTACTGAGCTATGCGTGGCTACTTTGGAAGGATGGAACACCTTTGGAACTGATGGACCCCATATTAAGAGAATCCTACNATCAGAATGAAATCATCAGAAGCATCCATATTGGTCTGCTCTGTGTCCAAGAAGATCCCGAAGACAGACCTA

>PvRGL066

CCGTTGCTGTCGGGTGGTTTCATTCTTGTGCTGCTACTGGCCAAGGTTTTGTTTCATGTGTATGAGCATTATAAGATGAAAGGAGAAGACCAAGCTCGGATAGAGAAATTCTTGGAGGATTACAGGGCAATGAAGCCTACCAGATTCACTTACGCTGACATTAAGAGAATCACAAATGGGTTTAGTGAGAGTTTAGGAGAAGGAGCTCATGGAGCAGTGTTCAAGGGAATACTCTCCCGTGATATTCTCGTTGCTGTGAAGATACTCAACGACGCAGTTGGAGATGGAAAGGATTTCACAAACGAAGTGGGAACCATGGGCAAAATTCATCATGTTAATGTTGTTCGCTTGCTTGGATTCTGTGCAGATGGATTCCACCGTGCTCTTGTCTATGATTTCTTCCCAAATGGGTCACTGCAGAGATTTTTGGTTCCACCCGACAACAAGGATGTTTTCCTTGGTTGGGAGAAGTTGCAACAAATTGCTCTTGGTGTTGCCAAAGGGATTGAGTATCTCCATCTGGGCTGTGATCATAGAATACTTCACTTTGACATCAATCCTCACAATGTTTTAATAGATGACCACTTCAATCCAAAGATCACTGATTTTGGACTTGCTAAATTGTGTCCTAAAAATCAAAGTACTGTGTCTGTGACTGCAGCCAGGGGAACCTTAGGCTACATTGCCCCTGAAGTTTTCTCAAGAAACTTTGGCAATGTATCTTATAAGTCTGACATTTACAGTTATGGAATGTTGCTCCTGGAGATGGTGGGAGGAAGAAAGAATACAAATATGTCAGTAGAGGAAAGTTTCCAAGTTCTGTACCCTGAGTGGATCCATAATTTGGTGGAAGCCCGAGATGTAGAAGTTACAATTGAGAAGGAAGAAGATGTTAAAGTTGCAAGGAAACTTGCCATTATAGGACTTTGGTGTATTCAGTGGAACCCAGTGGATCGTCCATCCATGAAAACTGTGGTACAGATGCTTGAAGGAGATGGAGAGAAGTTAATTGCACCCCCTACTCCTTTTGATAAGACCGGTTCTTCGAAAACAAATGCAGTTGTTCCAACAAGACACCAGAATTTCGAGTTGGAAATTATTCATGAAATGGAAGAGTAAATAAAGTCTCACTTTTAAGTTTCCATCAAATGTAATTTGTACTTTAAAGTTGTAACACAATAAGCTTCTTCCCTTTTTAAGCTTACTTAATTTGCTTGT

>PvRGL067

AGAATTCGCATTCCCAGATCTGTTCTTTTCACTGCTGCAGGTTCTGTTTTTCTGGGGCTGGCGGTTATTGCCGTCTTTAAGATAGACCGCCATTTTAGAAAGAAAGAAGAAGACCAAGCTAGAGTGGATAAGTTTCTAGAGGATTACAGGGCAGATAAGCCTGCAAGATTTACCTATGCTGATATTAAAAGAATCACAGGTGGTTTTAAAGAGAAGTTGGGGGAAGGAGCTCATGGAGCTGTATTTAGAGGAAAGCTTTCGAATGAGATTCTGGTGGCTGTGAAGATCCTCAATAATACAGAGGGAGATGGGAAAGATTTCATCAATGAAGTTGGAATTATGGGAAAAATCCACCACATCAACGTGGTTCGTTTGCTTGGCTTCTGTGCAGAAGGACTCCATCGTGCTCTTGTCTACAATTTATTTCCAAACGGTTCGCTACAAAGCTTCATATTTCCACCGGACGACAAGGACCATTTTCTTGGCTGGGAGAAGCTTCAACACATTGCTCTTGGCATAGCTAAAGGGATTGAGTATCTTCATCAAGGTTGTGATCGTCCCATTATTCACTTTGACATCAACCCTCACAATGTGTTACTCGATGACAACTTCACTCCGAAAATTTCTGACTTTGGCTTAGCCAAATTGTGTTCAAGGAATCCTAGTTTGGTGTCCATGACAGCTGCAAGGGGAACCTTGGGATACATTGCACCAGAAGTTTTTTCTAGAAACTTTGGAAATGTGTCTTATAAATCTGATATCTACAGTTATGGAATGTTGTTGTTGGAAATGGTTGGAGGAAAGAAGAATGTAGACATATCTTCAGCACAAGATTTCCATGTTCTCTACCCAGATTGGATCCATAACCTGGTTGATGGAGATATCCATGTCCATGTTGAGGATGAAGATGAAGTTAAAATTGCAAAGAAACTAGCTATTGTTGGACTTTGGTGTATTCAGTGGCAGCCAGTGAACAGACCATCCATAAAATCTGTCTTACAAATGTTAGAAACTGCAGAGGAAAACCAGTTAACTGTTCCTCCTAACCCATTTCACTCAACGACTTCCACTGCTACTGGCAGACTCACTTCAACAAGACCTCCTATGGCGTTGGAAGCGATTCAAGAATAAGCGAATAACAACTTCTACCCTCGCAATTTTATTAATGTATTCACAGTGGAAGAAAATAGTATATTAATTTGTAGAAAAAAAGAACATGTATTTTATGTAATATGATTACATTC

>PvRGL068

CCTTCTTCTGGTCCTTCCTCAAGAGGTACATCAAGTATAGGGACATGGCCACCACACTAGGCCTGGAGCTTCTCCCTGCCGGGGGTCCCAAGAGGTTCACCTACTCAGAAATCAAGGCTGCCACCAATGACTTCTCCAATCTCATAGGCAAAGGAGGCTTTGGAGATGTCTACAAAGGTGAGTTGCCAGACCACAGGGTGGTGGCAGTGAAGTGCCTCAAGAATGTCACAGGTGGAGATGCTGAGTTTTGGGCAGAAGTCACAATCATTGCCAGAATGCACCACCTCAACTTGGTGAGGCTTTGGGGTTTCTGTGCTGAGAAGGGCCAAAGGATCCTAGTCTATGAACACATCCCATGTGGCTCAATGGACAAGTACCTCTTCAGGGTCAACAAGTCTCACAGTAACAATGACAAACACCTCAAAGACCAATCAAGTCCAAACACCCCACAACAAAAACCAACCTTGGATTGGAACATGAGGTACAGAATAGCCCTTGGTGTGGCAAGGGCTATTGCCTATTTACATGAAGAGTGTTTGGAGTGGGTTTTGCACTGTGACATCAATTTCAAATCATCTAATATACTGTTAGATGACAACTTTAATCCTAAGCTCTCTGATTTTGGCCTTGCTAAGATTGGTCCTTTAGAAGGCCAGGAGCAGGTGACAACCAGGGTGATGGGAACTCCAGGTTATTGTGCACCAGAGTATTCTGCTACGGGTAAACTTACCACAAAGTCGGATGTTTATAGTTTTGGGGTGGTGTTTTTGGAAATAATCTCGGGTAGGAGAGTATTTGACATACAAGAGCTACAGAAGAGAACGAAGCCTTGTGGAATGGGCAAGGCCTCTTTTGAGGGACCAAAGAAAACTGCACCATATAATAGACTCTAGATTGGAAGGGCAGTTTCCTACGAAAGGGGCTTTGAAGGTTGCTGCATTGACCTATAAATGTTTGAGCCACCACCCCTATCCAAGGCCAACTATGGGTGATGTGGTTAAGATATTAGAGTCTGTTCAGGATTTTGACGATGTGATCAGAGGACCATTTGTGTATGTAGCGGTGAGTGAAAGTAGCCAATGAGATGCTACTGTCAAAGGGCATGAAGGAGTCCTTTTTCTGTGGTAACAACAACTCATCAGCAACGTAGATAAAATATAGCAGATAGCAAAACAAATGAAGGAACAGTTAATGACAATCTTGTTTTTGCTCATTTTTACTTGTTAAGGCATGGCCTACAAAGAAAATAAGTGATTCAAGTAATTAAGTAGTAGCGAATAATATTTTACAGAAGCATACGTGTGTACATCAACCTATTTTTTTTTTGTATCTTTTTTCCTTTGTGTGGGTAGGTTTAACTATGAAGTGAATTGAATTGACATTATGTAACCAATTTAACTACCATGGAACTACCAAATTTTGGTTTTGGTTATTGTTGTTATTTTCCCAGTAATAACCAAGAAATGTCTTGGCAGCATCCAAGCTAGGGAAGTATGTGGGTTGAAGGAATATCTGAGTTAATCCATCGGCCTCGGAGCTTCCCATCAGAAGCGTCGCAACCTGCTTGTACGCGAAGAAGAACCTCTTCATCGCCACCTCAACCGCTTGCATACCCTGCAAAACAACAACCTTCTCCGACCCCTTCGTTTTCTCCGCGTACACCTTCATGGCCGCCTCCATCATCCCGGGCCCACCCTTCGGATCAACCTCCACTTCCAATATCTCCGGCACCTTCTGCCTCAGATCCCTACATACCCTCGCCAACTCCACAACCTCCTTCGACCTAAAGCAAAACGCCACCGTTTCGCCTTGCCTTCTCATCCCCAACTCCTTCTTCAGCCGAAGAAAATCCTCCGGATCCATCATCACATGCACGTCCTCAACCTCCGCCAGAAGCTTCCAGCATCCGCTCCACCGCGTGGCACTCACTCGCCGCCTTCTCCAACGCCCTCCTCTCCACAGACTCCACCAATCTCTCCACCAACTTCCTCCCCACACGCGTCCAGCACTCCACGATCTGCTGCGCCGCGTGCAGGGTCCTGTTCTCGTAGTTCTCCACGTGATCAAACGGCGTCGGATCCAGAGACGGGATCTCGGTCGGCCTGCAAACGGCGTCGTAACGGAGAACCTTCTTTCCCGCGAGGTTGGCCTCTCCCAGGCCCAGCGTGTAGGTGCATCTCGTCATCTCGCATTCCACGGTGGCCAGGATCCTCCTCGCCACATCCCGGGACCTGTGCCACGTGGCGAGACGCGGGAGAAAGCTCTCCTCGCTGTAGCTTCTTCTTTGAGCGGTGACGAAGTCGTTGACGTCACAGACAGGCGCCTTGCCACGTGTCTCTGGGTTTTGCTCCTCGTCTTCGCAGAGCATGGCAATGATCTGGACCTGCGCGGAGGCGAGAGACTCGAGGCGTCGGGCCCAGTCGCGCTTGTTAACGTAGGGCCTCGGGTCCGATAAAACGGTTGCGAGAAATCGGAAGGTGATTTCGAGGGCGTGTAAGGCGGGCTTTATGATTGGGTAGTTGGGCCAATCGGGGAAGTGGTTAGAGGCCCAAAGATTTCTGAGGTCGGGGAGGCGAAGGTAGTTGTCGTAGGCAGCACAGAGCGGGGAGGAGGCAGCGGTGATGTCGTTTTGGAGAAGAGGGATTTGCAAAGTGGGGATTGGGATGTTGTTTGGGAAGAGGGAGAGTTTGGTGGAGTTGGGAGACATGTTTGAACACACCATCTTTGTTTGCCAATCTAGATCAACCATGTTTGGGAAGATGGATAGGAAGAAGTAATAGTTTTGTGAATTAGAGAAG

>PvRGL069

CACTCCTCAACGGGTCTACGTATGCATTGGCGCTGGCATGGTTTGCGTAAATGGATGGTTATAGTTGGGTGCTCCGTGTTGGGGGTTCTGATAATTTTGCTTTACGGGTGTCTTTTATCGGTCAGAAAGAGGACAAAGAAACAATCCAAGTCTTGCATGGGGAAACCGGAGCAAGAGGATGATAATGTCAATGTTGCTCTTCAATCAACACCTTCTGTTAATTCTTGTCCCGGGGCGCCTCAGGTTTTCAGACTTTCTGAACTGAAGGATGCTACGAATGGGTTTAAGGAGTTTAATGAACTTGGGAGAGGAAGTTATGGGTTTGTATACAAAGCTTTGTTGGCAGATGGGAGGGTGGTTGCTGTTAAAAGAGCAAATGCGGCTACCATAATCCACACCAATAATCGTAATTTTGAAATGGAACTAGAAATTCTCTGCAAAATCCGCCATTGTAATATTGTTAATTTGCTGGGGTACTGCGCAGAGATGGGGGAGAGGTTGCTTGTTTACGAGTATATGCCTCATGGAACGCTTTATGATCACCTCCACGGTGGTCTTTCTCCCCTTAATTGGAGCCTCAGGTTGAAGATAGCGATGCAAGCCGCAAAGGGGCTTGAGTATCTTCACAAGGAACTTGTTCCTCCAATTGTGCATAAAGATCTAAAAAGTTCAAACATTCTTTTGGATTCGGAGTGGGGGGCAAGAATTTCGGACTTTGGACTTCTTGCTTCGAGTGACAAAGATCTCAATGGAGACATAGATAGCGATGTTTACGATTTTGGGATTGTGATGCTAGAGATTCTGAGTGGAAGAAAGGCTTATGATAGGGATTACACTCCACCCAGCATGGTTGAGTGGGCAGTGCCTTTGATCAAACAAGGGAAGGCTGCTGCTACTATTGATAGGTACGTAGCTCTTCCAAGAAATGTTGAACCTTTGCTTAAGCTTGCTGATATAGCAGAACTTGCTGTGAGAGAGAATCCAAGTGAACGACCACCTATGTCCGATATAGCATCTTGGCTGGAACAAATTGTGAAGGAAGGATTGATCTTATAGCATTAGTGAATGCCCTGTGAGAAACTTGTTTTTGAATTTTGATCCATCCACCCTATCTTTCATAAAATTATGTGAGTAATGCCCTTCTGCGAATGTAAATTGTTTTTGAGTTTGTTGTAGATTGTCTATCTTTTTTGCAGTTCTTTTGAGTTCACTTTTTCCAATCTTCACAAAGTGAAGGACATGGTATTATTTAGACATACACAATTGTTTTTATCTGTACATGCTGATGTCAAAGGTTTTTTTACTTCTCATATAAGCCTAAGAGCATCTCAAGCATACAGATAGAGTCAATTTACATGTACAACAGTGAGAAAATGGTAGTCTCAATTGGAATTATTTTGGTGAGGCATTAGTGATTCAATGAATGTGCAAAGCAACCTTGTTCTTTCTCTA

>PvRGL070

GTAATTCATCCATTTCCTTCAAAAGAAGAAGCAGCAGTGATGCAGTGGCAAAAATGCAAAAACAAGAAGATAAGAAAATTAAAAGAGGAAATATAATTAACGAGGGAGGAGTTTATTTTGATTGGCCTGAAGCATCTCTGCAACTTGGCTCATGGATGGTCTTTCATTCATGCCATCTTCCACGCACTGCAAAGCCACTTTGACCAAAACCTCTACTTCTGAAACGTCATAGTTTCCTTCCAAATTGGGATCGATTATCTCCTCCATCCAAAACGCACAAGTTGGTGAGTGCTTTATCTTCTCCCTTATCCAAGTCACCAACCGCTGCTGTTCAATGTCCCCGCTGTTCTCAAGACTGTGGATTGCCATAGGGTTTCTTCCTCTCACCATTTCCAACACCACAATCCCATAGCTATAAACATCCACCTTAGAAGTGATACGGCGATTGTAAACCCACTCTGGAGCCATATACCCTCGAGTCCCTCTTATTCGTGAAAAACTAGAATTTCCACGCTCGTCTCTGTTTAAAAGTTTTGACAACCCAAAATCTGCTACCTTGGGTAGGAAATTTGAGTCAAGGAGAATGTTTTGAGGCTTCACGTCGCAATGAAGAATCCACTCCAAACACTCTTCGTGTAAGTAAGCCAAACCTTTCGCTGTGCCCACTGCCACGTTGAACCTTTTCTTCCAATCAAGTGCGTTGCCAAAGAGATTGTCTGCTAAAGATCCATGTTCCATGTACTCATACACCAAAAGCCTGTGCTTCCCATCAACGCAGTACCCCCACATGTCAATCAAATTCATGTGGTTCAGCATGCCAATTGTGCTTATTTCAGCCAAAAATTCAGCTTCCCCTTGAGTAGCTTCGTTTAAGCGTTTAATAGCGGCAACACGGTTATCATATAACGTCCCTTTGTAAACAACCCCTCCTGCTCCTCGCCCAACCTCTTCTTTGAACCCTTTTGTTGCCGATTTTAGCTCTGCGTAGGTGAACCTCTGAAACCCTGTGGCAGAAAAAAGTTGTTGTTGTTGATCCTTCGTGTCAGGGTGCTTGCTTGTTCTTAACAAGAAGAACCACACAAGGAAAATGGTGGAAAACTCAAACACCCCGACCCCGAAAGCGAACCAAACCAAGAAACTCAACGTCGAGCTTTTTTCTGGCTGTTGATAAATCCTGTTCAACTTTTGAGACAAACCAACGGAGCAATTCATCGGGGAATGCTTTAGAATCTTCGTGGACGAGCGA

>PvRGL071

GGACTTTCGGGACGGCGTATAAGGCGGTGCTGGAGGCAGGGCCCGTGGTGGCGGTGAAGAGGTTGAAGGATGTGACGATTTCTGAGAAGGAGTTTAAGGAGAAGATTGAAGCGGTGGGAGCTATGGATCATGAGAGTTTGGTTCCTCTCAGAGCTTTCTATTTCAGCAGAGATGAGAAGCTCCTTGTCTATGATTACATGCCCATGGGAAGCTTGTCTGCACTTTTACATGGAAACAAAGGGGCGGGTAGGACGCCACTGAATTGGGAAGTGAGATCAGGTATTGCACTTGGAGCAGCCCGTGGCATTGAGTACCTGCATTCACGAGGGCCTAATGTTTCTCATGGAAATATAAAGTCATCCAACATCCTCTTAACCAAATCATATGATGCCAGAGTGTCTGACTTTGGCCTTGCACATCTCGTTGGCCCCTCCTCCACCCCCAACAGGGTCGCCGGCTACCGTGCACCTGAGGTGACTGATCCTCGCAGGGTGTCTCAGAAGGCAGACGTGTACAGTTTTGGTGTGCTACTCTTGGAACTTCTGACTGGGAAGGCACCTACCCATGCTCTCCTGAATGAGGAAGGAGTAGACCTTCCCAGATGGGTTCAATCTGTGGTTAGAGAAGAGTGGACTTCTGAGGTCTTTGATCTTGAGCTCCTCAGGTATCAGAATGTGGAAGAGGAGATGGTTCAGTTGTTGCAACTTGCAGTTGATTGTGCAGCACAATACCCTGACAAGCGCCCTTCAATGTCTGAAGTGGTAAGGAGCATAGAAGAGTTGCGAAGGTCTAGCCCTTAAGAGGAGCAGGAGCAGGACCAAATCCAACATGATCCCGTCAATGATATAGAATTGTAGAAGATGTATCTTCTGCCCGAGTTTCTTCCTCTGGACTTTGCTTTGTCAGAGTCGGAGCCCATATTTTACCTGCTAAAGCTAAATCCTGTGCGTATGTTGGTTGTATTCTGTAGCTTGGGCCTCAAATTTCTTTGGCACCAAACTTTCCAATTTCTTGGTTTTTTCTCTCTCCTCAAATCTCCATTGTGCCGTCTCCTCCTTTTTTCTTCTCAATTAATACATTGGGGGAGTGGATATCTGTTCCATTAGCTATTGTTGTTGTTGGATTCTGGTTGATTGTAATTATTACTTTTGCTTTAAACATGCCTTTTACTTTTGTTGAATTCTTGTGTCTGTTGAAAACTTGTAATAGAGATGTTATAGTAGCTGTCGTAGGATGGTTTGTTGTGATGTCTCGTCGGTGGCTTTTATTATTGCCAAAAAAGTTGGTTGTGAACGTGATCGCCACCTTGGTATGGTAAGGATGGGCTTTGATTAATTCATCCTTCTTTTTGCCACTTGGTTTAAGATTTATTTATACGCACACACCACAAACAAGGTAATATTGACATTAAAGAATTCAGTGTA

>PvRGL072

GTCAATTTGGTTGGGTATTGTGCGGAAAAGGGGCAACATATGCTTATATATGTGTACATGAGTAAAGGCTGCTTGGCTTCCCACTTGTACAGTGAAGAGAATGGAACTCTGGGTTGGGAATTGAGGATTCATATAGCTTTAGACGTAGCAAGAGGAATAGAGTATCTTCATGATGGGGCAGTACCTCCTTTAATCCATCGAGATATCAAATCTTCCAATATTCTCTTGGACCAGTCCATGCGAGCCAGGGTTGCTGATTTTGGACTCTCAAGAGTAGAGATGGTGGATAAACATGCAGCAATTCGGGGTACCTTTGGGTATCTTGATCCTGAGTATATATCTTCAGGAACATTCACCAAAAAAGTGATGTATATAGTTTTGGGGTTTTGCTCTTTGAACTTATAGCGGGCAGAAATCCTCAGCAGGGTCTAATGGAATATGTTGAGCTTGCAGCAATGAACAGTGAGGGGAAAGTTGGTTGGGAGGAGATAGTTGATTCAAGGCTTGAGGGTAAATGTGAGTTTGAAGAGGTGAATGAAGTGGCAGCCCTTGAACCTACACATGCCAAAAGATTACAAAAATTCAAAGTCTTCCGGGACTTCCAAGTGGGGTCCACTCTCCTTCGCAACGACCAAGTCAACCAACACAAACTCCTCTCTACCGTCCGATCTTTGCCGCCACTTCTCCCTCGCGGAGACCAAAGCCGCCACCAACAACTTCGACGAAGTCTTCATCATCGGAGTCGGGGGATTCGGCCACGTGTACAAGGGCTACACCGACGGCGGTTCCACTCCGGTGGCCATCAAGCGTCTGAAACCGGGTTCGCAGCAGGGTGCGCACGAGTTCATGAACGAGATCGAGATGCTCTCGCAGCTCCGCCATCTTCATCTCGTGTCCCTAATCGGATTCTGCAACGAGATGAACGAGATGATCCTGGTGTACGACTTCATGGCGCGTGGCACCCTACGCGACCATCTCTACAACACGGACAACCCCGCCATCCCGTGGAAGCAGCGGTTGCAGATCTGCATCGGCGCCGCGCGTGGACTCCACTACCTCCACACGTGGCGCGAAGCACGTGATCATTCATCGGGACGTGAAAACGACGAACATCTTGTTGGATGAGAAGTGGGTGGCGAAGGTTTCGGACTTCGGGCTATCCAAAATTGGGCCGAATGAGATGTCGAAGGCCCATGTGAGCACCGCCGTGAAAGGCAGCTTTGGATATTTGGATCCGGAGTATTTTATTCGGCAGCGGCTGACGGAGAAGTCTGACGTGTATTCTTTGGGGGTGGTGTTGTTTGAGGTACTCTGTGCTCGTTCTCCTCTGATCCATACGGAAGAAATCGAACAGGTTTCACTAGCTAAATTGGGCCAGATATTGCTACCAAAACGGGACAGTGGCGGAGATTGTGGACCCCATATTAAAGGGGAAGATCGCTCCAAAGTGTTTCGCCATGTTTTGTGAGATTGGAGTGAGTTGTTTATTACAGGAAGGGACGCAGAGACCGTCCATGAACGATGTCGTTTTGATTCTGGAGTCTGCATTGAAACTGCAAGAGAGTGCCGACGAAAGCGAAGAGGAAGTTCGTGAAGATGTGTTTGATATAGAAGAGCACCACATTAGTTCGAAGGACAATGATAAGTTGATGCTTGAGTTGTTTTCCGAGATTGTCGACCCAAAGCCACGTTGAAGTAACCCAATAATGTTCACCAAATGCTTGTTCAACAATTACATTTCAAGAACTAATTAATACAATCTAGATGCTCCAGATTTATGTGTTTAGTTTCGTGATTGTAATGTGAAAGTTTCTCTTCGCAAAGACGACACGTGCAAGGTTTCTTGCT

>PvRGL073

GTGAGCCAAGTCAGAGTGTGTCTTCCAACTTCGAAGCTCCGACACANGCTTTTTAACCATTCTCGCTTTTTCTCGCATTGCCACTATGCTTATTTCTAGGGTTAGGGTTTTTGCTCCTCAGAAAAATCGAAATGGACGAAGATTACGGCTACAGAAGGAAGGCCAAGATTGCACTCATCGCCATTATTGTCCTCGCTTCCCTCGCCGTCTTTGCCTTGCTCGTTGCCTTTGCTTACTACTGCTACATCCTCAACAAGGTCTCCAACCGCCGCAAAAGCCTCAAAAAAGTTGAAGACGCTAACCTTAATGAGAATAGTGACTTTGCAAATCTGCAAGTTATAGCCGAGAAAGGACTTCAGGTGTTCACTTTCAAGCAGCTGCATTCTGCTACAGGGGGCTTTAGCAAGTCGAATGTCATTGGTCATGGTGGATTTGGACTTGTTTACCGAGGAGTGCTCAATAATGGCAGGAAAGTTGCAATTAAATTCATGGATCAGGCAGGAAAGCAAGGGGAAGAAGAATTTAAAGTAGAGGTGGAATTGCTAAGGCGGTTGCATTCACCATATCTGCTGGCATTGCTTGGGTACTGCTCTGATAGTAATCATAAATTGCTGGTGTATGAATTTATGGCAAATGGTGGACTGCAGGAACATCTTTATCCTGTTAGCAATTCTATAATAACACCCGTAAAGTTGGATTGGGAAACTCGGTTAAGAATAGCACTTGAAGCTGCAAAGGGATTGGAATATCTTCATGAGCATGTCAGTCCCCCAGTTATTCACAGAGATTTTAAGAGCAGTAACATCCTCTTGGATAAGAAATTTCATGCCAAAGTTTCTGATTTTGGATTGGCAAAGCTTGGACCTGATAGAGCTGGTGGACATGTTTCAATTCGAGTTTTGGGCACCCAAGGATATGTTGCCCCTGAATATGCACTAACAGGGCATTTGACCACAAAATCAGATGTGTACAGTTATGGTGTTGTACTTTTAGAGCTGCTCACTGGCCGAGTGCCAGTAGATATGAAGAGACCTCCAGGAGAAGGTGTTCTTGTTTCTTGGGCTCTGCCTCTTTTGACTGATAGAGAAAAGGTTGTCAAGATTATGGATCTTTCACTAGAAGGACAGTACTCTATGAAAGAGGTTGTCCAGGTGGCTGCAATTGCCGCAATGTGTGTGCAACCAGAGGCAGATTATCGTCCTCTCATGGCAGATGTTGTGCAGTCATTGGTTCCTCTGGTCAAGACTCAAAGGTCCCCTTCAAAGGTGGGAAGCTGCTCTAGTTTCCACTCCCCCAAGTTGTCCCCCGGCCCAACATCCAGTTAGTGCTTGTAACATATATTATCAACATTAAATGAAGGATAGGTACATGCTCACCTAACTAATATTTATAGCTTGGTAGTAGTTGCCTTTTCTTTGTAATCTTAAACCATGGGATACTTTTTGAAGAGGAAAAAAATGGTGGGTTCAATGCTAATGTTCAATTTGATGAAAGATTAGATTTGGTTATCTTAGTTTGTGATGGAAGGTTCTGCTTAAGAGACA

>PvRGL074

ATCGTGGTCTGTGCGGAGTGAGAATAAATTCTACATGTAAAGATGATGGTTCACCGGGGAACAATTCTCAATCTACTTCCTCAGATCAAAATCAAATGGGAAAGAAGAAATATTCTGGTCGGCTGCTCATCAGTGCATCAGCAACTGTGGGAGCTCTACTTTTGGTAGCACTTATGTGCTTCTGGGGTTGTTTTCTTTACAAGAAGTTTGGTAAAAATGACAGAATAAGCCTGGCAATGGATGTTGGTGCAGGTGCATCAATTGTGATGTTTCATGGAGACCTTCCGTATTCTTCCAAAGACATCATTAAGAAATTAGAGACTTTGAATGAAGAACACATAATAGGAATTGGGGGATTTGGAACTGTTTACAAGCTTGCAATGGATGATGGGAATGTCTTCGCTTTAAAAAGGATCGTAAAATTGAATGAGGGGTTTGATCGTTTTTTTGAGAGGGAGCTTGAAATTCTTGGTAGCATCAAGCATCGTTATCTAGTGAACTTACGAGGATATTGTAATTCACCAACATCGAAGTTGTTAATATATGATTACCTACCAGGTGGTAGTCTTGATGAAGCTCTTCATGAAAGGGCTGAGCAACTAGACTGGGATTCACGCTTGAATATAATCATGGGAGCTGCAAAAGGGCTTGCTTACTTGCACCATGATTGTTCCCCTAGGATTATACACCGAGACATAAAATCTAGTAACATTTTGCTTGATGGAAACTTGGATGCTCGAGTATCCGTTAATTTTCCACTTGTTGCAAACTGCTGGAGGAATGTACCCAGAAGGTCCCACATCACCACTATTGTTGCCGGATACATTTGGGTATCTTGCATCGTCAGAGTATATGCAACGTTGCAGAGTCAACTGAGAAGTCTGCAACCTGTGTCCTCATAAGCATTATCCAAAAGAATGTTTGCCGATTTAATATCTCGGTGAATAATCTTTGGGTTACATCCATCATGAAGATATGACAGGCCCCTTGCAGAACCAATTGCTATT

>PvRGL075

CAACCATGGGTAACCTCTTCTCGCTCAGCCTGTGATTAATATGAAAGTTGTGAGCATGGAGACAGAGAAGAAGTGTGGTTGCTGGTCTGTCCTTAAACGCGGTGTTAGAGGTGCGTGCAAACCTACAGCTTCCAGAGACTCTGCTAACACTATCCCTCGTTCTAGTCTCGTTTATGATGCAGCAACCGAGACAAGATACCTGAATGCTAGTAATCGAGAACTTTGTCCTCCCCTTGAAGCTCGATTATCTTCTGATAACCCTGATCCCCCACCCCAGGAGACCAAGGCTGCATGCCAGCTTCTTCAATTTTCATTTCAAGAACTTAAAGCTGCAACTGGAAATTTCAGACCTGATAGCATTCTTGGGGAAGGTGGCTTTGGTTATGTTTTCAAAGGATGGATTGAGGAAGATGGAACCTCGCCAGCTAAACCTGGGTCAGGGATTACAGTGGCTGTCAAAAGCTTGAAGCCAGATGGCCTTCAGGGCCATAGAGAATGGGTGGCTGAAGTTGACTTTCTTGGACAGCTTCACCATCATAACCTTGTGAAACTTATCGGTTACTGCATTGAAGATGATCAGCGGCTTCTTGTYTATGAGTTTATGACCCGTGGAAGTCTAGAAAATCATCTATTCAGAAGAACCGTACCCCTCCCGTGGTCCAACAGAGTTAAGATAGCCCTTGGTGCAGCAAAAGGATTGGCCTTCCTCCATAATGGTCCAGAACCAGTCATTTATAGAGATTTTAAGACATCTAACATTTTGCTTGATACGGAGTATAATGCAAAGCTTTCAGATTTTGGTCTAGCAAAAGCAGGACCTCAAGGAGACAAAACACATGTTTCTACCAGAGTAGTTGGAACTTATGGTTATGCTGCTCCAGAATATGTCATGACAGGGCACTTGACTGCTAAAAGTGATGTTTATAGCTTTGGAGTTGTGTTACTTGAGATACTAACAGGAAGAAGATCAATGGACAAGAAGCGCCCTAGTGGGGAACAAAATCTTGTTTCTTGGGCTAGGCCATACTTAGCTGACAAGCGAAAGCTCTACCAGCTTGTGGATCCTCGCCTCGAGCTAAATTATTCTCTAAAAGGAGTGCAGAAGATTTCCCAGTTAGCTTATAGTTGCCTCAGTAGAGACCCTAAATCTCGCCCCAACATGGATGAAGTTGTGAAAGCGCTTACTCCATTGCAGGAGCTTAATGACCTTGCAATTTTGTCTTATCACACTCGCTTGTCTCAACAAGGGAGACGGAAGAAGAAAGATGGAACTCCACACATTACATACACACAATCCAAGAGCATGAGGGCCTCTCCCTTGAATACCAGCAAGCAGCATCGATGATAGGAAGGAAGTGATCCTCTTCTTTATTGCTGGCTCAATCAAACCAAGGTTGGAATGCCTCCTTNCGTGTTGTTGTTGTGTGGAGATGGATGTTTGAAGCTTACATATTTTTTGGGTGTTTAAAATATGTCAGGGCCAGAAGAATGAGGAAAGAGTGATGAATGATGATACATTGATTTTATTTGAGCTCTTTTATTAGAGAGCTAACCATCAATATGGAAAGAAACTTGTCATTTGCTTATGCTGTAACTGAGAATTCCCCTATATGTATATGAAATGCCTCACTTCT

>PvRGL076

TGTTGAACAAACTGATTATTATATTATAGTGAAGATGCTACGAAGTACAAAAAGTGGAGGAAGCTTAGAAAAAGCTTAAAAAGGTAGAAAATGACCCTCCTCCATCTTTATTTTTTAAAATAGCAATTAGTGGATGATATAGAAATGTTAACTATGATTACCTCTTCTTACCCTCAGTTTCCCTAATAATCTCCAATAAAATACATCATTAGGTACTTGCGTGTTTCGCTCGTCTTTCTCTTCCACTGCTGATGATGTGAGGAGGAGCCAAGCCAGTTACATTAGTTCTGATGCCACCACGATTACAACTACACATATTTCCACACAATAGTTCCCACCCAATGAACACAAGCCCAAACATGGATACAAAAATTATGCACCGGGCTTCTTGTCACATTATCATGTACAAAGCAACAAAACACAAGGATGCACCCATTTTAACAACTACTATTTCTTGAGATAAGTGGAGATTCGAGAAGCACACCAAGAAGACAGGGTTTTAAAGCTGCATGAGATTATCATCTCACCCGTAGTGCTGCTGGGGCACCTGGTGAACAATTTGGGTTTGGAGATCGACAGGTAGCACCAGGACCAACATTGTTTGAAAACCTTTGGCGCATACGGTAGTCAGAGATTTTGTTCATCGCAAAAGGCCCAGCACTCACACTATTTGATCCAGATAATGAGACTTCACCTGCACCAACACTGGAACTTTGTAAGGGTTCCAATGTCTCAACTACATCGCTCATTAGGGGCCTCGCTTTGGGGTTTTGGCTTAAACAGTAATAAGCCAAGCTACATGCTTTTTGTGCAGCCCTCACTGAGTATTGATTCTCCAACCTAGGATCAATTATTTGTAGCAGCTTTCTCTTGTCATTTAGCTTTGGCCTTGCCCAATCTACCAAGCTCTGTTCCTTCCCAGGTCTGGTCTTGTCAACTGATTTCCTTCCCGTTAAGAGCTCCAAAAGAACAACACCAAAGCTATAGACGTCACTCCTAGCCGTCAGGTGGCCAGTCATTACATATTCAGGGGCAGCATAACCATATGTACCCATGACTCGAGTGGATACATGGGTTTCGTCACCTTGTGGTCCTGCTTTAGCTAGTCCAAAATCAGAAAGCTTTGCTGTATAATCCGAGTCCAACAATATATTAGACGTTTTGAAGTCACGGTAGATAACCGGCCTTTCAGCATTGTGGAGAAATGCAAGTCCTTTGGCAGCTCCAAAAGCAATCATCATTCTTGTAGCCCATGATAAAGGGACAGTTGCCTTTCGGAATAAGTGGTTTTCCAAACTCCCACGGAACATGAACTCGTACACAAGCAGCCTATGATCATCTTCACAACAGTACCCAATCAACTTCACCAGATTAGGATGCCTTAGCTGCCCTAGAAAATTCACCTCCGTAAGCCACTCTCGATGTCCCTGTAATCCTTCCTTGTTCAGAACCTTAACCGCTACAGGGAGCGATTTGAGTCCCACTCTAACGTTCTCATCAATGTAGCCTTTGTAAACGGTGCCGAACCCTCCCTCGCCGAGGATGTAGTCTCCGCGGAAGCTCTTCGTTATGGTCTCCAACTCGTAGAGCGTGAACGCTATCACGTGCGTGCTGTTGTTATTGTTACCGCGCGGTGTCGAACACGCTTCGCTCAGATCCGAAGCGCACTCA

>PvRGL077

CGAAATGATTTATTTTAAATTGTTGAATAACTTATCTGAAATAATCTGAACCCCATGTTTTTATTGGAAAGTTTGTTCTACAAGACTAACTTTTAAGCAAGAACTGGTCTAAACTAAAAAAGAATTGTCCTGAAAATTTTAGACAAGGTGAATCTTTGGTGACTCTACAAATGAATCCATTGATTAGCTTTAAAATGCATTTACTGACAAAGCAAGAGATTAAAATGCAGGTCTTTACCACATGATTTTCGTATCGCCTAGTTTATGCATGGAATCAATAACCAATTCAGGAAAAATCAGAATGCATAGTTTCAACTATGAAGCTATCAAAAGTTACCAATCATATGCTCTGGTGTTCTTCCCTTGGAAAGCTTTGAACAGTATCTGACCATCTACCAGGGGAGTGATCACTACTGTTAGATGAGACATGTGCCTCCCCTCTTGCATCTTTTATCATCTTTAAAACCTCTCTCATTGTAGGCCTGTTCTCTGGCACCAGTGAAACACATGCCATGGCAATGTTCAAAAGAGCCTGAAGCTTCTCTTCTGATGCTTCATTGCCTGAGGCAGGATCATCCCCTGACTCTGTCTCTTCTTCACGCACTGACCTGACCCACCTAGGTATGTCTGAACCATACGTCTGAACAAGGTCCTGGAAGGGTGTTTTGCCAGTTAGAAGCTCTAGAACAAGGACCCCAAAGCTGTATACATCAGCTGGTTGAGTTAGTGACCTTTGAAAGTTGCGGCATTCAGGTGCTCTATAGAACAAAGAAGTGGCACTAGGCTCATCCATTGATTCAGGATTTAGGAACGCACTGAGACCATAATCAGTAAGACATGACTCAAAATCAGAACCAAGCAACACATTCGAAGATTTAAGGTTTCCATGTGTCATGCCGGGGTTCTGGTGGATATAGAGCAGGCCTGTTGCTAGATCTTCCGCAATCTTAAGGCAGGATGTCCAGTGAAGAGGCTTTCCCCCGCCAGATGTTTTTGATCCGTGAATAAGGGAGAAGAGGCTGCCATTGGGAAAGTAATCATAGACCAGAAGACGTTCTTCCTTGGCCTGAAAATAAGCTCTGAGTGGGACCAAGTTGGGGTGGGTGAGCCTCCCAAGAACATGAATGTGAGCTCTGAACTCTTCCAAACCAGGGTACCTAGCATCCTTCAACCTCTTAACAGTGACGATGAACCCAGATTCCATAACTGCCTTATAAGTACTTCCAATAATACCCCTCCCCAGAGTCTCCGCCGAAGCCTTCAGCAAATCCTCCAAACTATAACTCATCTCGCGATCACCTCCGCCGCAGAACACCAGCTTCCCCAGACCCTCACCTTCCCACGCAAAACCCCCTTCTTTATTACTATCC

>PvRGL078

TGTCCTGAAGAAAGAACTTATAACCTTCATATATGGTAAGGTGTAAGAACAACTTGAGGAGCAGAACTCCTAATTGGTTTATATTTAGTACAACAGAATTTTTATGGGGTACCTTGCATGCAATAACTGACCAGCTTAATGGCACCCCTCACTATCATTCAAGTATACTACAAAGTAAATTGATTTCGGGTGGGAAAAAATCGATTACAAAAGCAGTAACTAAATAAGAAACAACCTTCATTCACCAATCAAGCCAGAATAGCCACTTACTTTGAAGGCCTTTGGCCAGAATTGTGGATACACAAGAGCTTATCTTCCGCGAGGATTTACCAATTGGGAGAACACTGCACTTGTAGCAGCATCTTCTGCATCATCATCTGTGCAAGAGTTGCCCCCATCAACCATACTCACACTGTTATCAAAATGGTCTAGGGGTGTCAACTGAATTCCGGTTATGTGGTTTGTGCTGTTATCTTCAGGCTCCATGAGGGCTGATGATGTCTCTTGAAGCTGCAATGCATATTCAAGATTCCACAAAACATCTCCCATAGACGGCCGGTCCACACCATGCTCGGCCAAGCACTTCTCGGCTGTCTCCCCAAACTTCTTAAGGGATGCAGGATTCACCTTGCCAACAAGATTTTGATCCATGATTTGATCAAGCATACCCTTTTTCTGCCAGGCCATTGCCCATTCAGCTATGTTGACCTGCTCCCTTGGAAGGACAGGGTTCAAGGCTGGCCTTGTGCATAACACTTCCATCAACACTACCCCAAATGAATACACATCTGATTTTTCAGTAAGCTGTTGCCTTCTGAAGTATTCAGGATCAAGATAACCAAAACTACCCTTAACAGCAGTGCTCACATGGGTCTGATCAAGAGCTGGACCAGTCTTGGAGAGGCCAAAATCAGCAACTTTGGCAACAAAGTTCTCATCCAAGAGGATATTTGTTGTCTTTACATCTCGGTGAATTATGCTTTGAGCTGCTCCGGTGTGGAGATAATGAAGGCCTCTTGCTGCTCCAATGCAGATCTCAAGCCGCTGCTTCCATGAGAGAGGTGGCAGGTCTGTTCCATACAAATGACTCCGGAGGGGTCCGTTAGCCATGTATTCATAGACAAGAATCATTTCTGACCTCTCATC

>PvRGL079

GGGGCGATGGTGGTTATGTTTCATTCATGAAGGTCTCCATTAGTAGTGATGATGGAGTACATAGTAACAAAAATGGAAGGAATGACACTGTGCTAGTTGTAGTCATAGTGATTCTCACAGTTCTGGTTATTGTTGGTCTTGTCACAGGATTCAGGTACTACTACAAGAAGAAGAATGTTGCAAGGTACCCTCAAGACACCTTTGAGGAAGATGATGATTTCTTGGAGAGTATCTCAGGGATGCCTGCACGTTTTACTTTTGCTGCTCTTTGTAGAGCAACCAAGGACTTTTCCACAAAGATTGGAGAAGGAGGTTTTGGCTCAGTGTATTTAGGTGAGCTTGAAGATGGCACCCAATTGGCTGTGAAAAAGTTGGAAGGTGTTGGACAAGGAGCAAAAGAGTTCAAAGCAGAAGTGTCCATAATTGGAAGTATTCACCATGTTCATTTGGTGAAGCTCAAAGGGTTCTGTGCTGAGGGTCCTCATCGCCTTCTTGTGTATGAATACATGGCAAGGGGTTCTTTGGACAAATGGATCTTCAAGAACAGTGAGAACAGTTTCTTGCTGAACTGGGAGACAAGGCACAACATTGCAATAGGCACGGCTAAGGGATTGGCCTATCTCCACGAGGAGTGTGAAGTGAGGATCATCCACTGTGACATCAAGCCACAGAATGTTCTTCTTGATGATAACTTCACAGCAAAGGTTTCAGATTTTGGATTGGCCAAGCTAATGAGCCGTGAGCAGAGCCATGTGTTCACAACTATGAGAGGCACAAGAGGGTATTTGGCACCAGAATGGATTACTAACTATGCAATTTCGGAGAAGAGTGATGTGTTCAGCTATGGCATGCTCTTGCTTGAGATAATAGGAGGGAGGAAGAACTATGATCAGTGGGAAGGGGCTGAGAAGGCACATTTTCCTTCCTATGTGTTCAGAATGATGGATGAAGGGAAACTGAGAGAAGTTGTTGATCCAAAGATAGATGTTGATGAGAAGGATGAAAGGGTTGAAGCTGCTCTAAAAGTTGGTCTTTGGTGCATACAAGATGATGTGAGTTTGAGGCCTTCAATGGCAAAAGTAGCTCAAATGCTTGAAGGGTTGTGCCATGTACCTGATCCACCATCATTATCTCAAGCTGGTACTTATTCAGCTTTCATGAAATTGAGCAGTGGAGAAGCTACTTCATCACAGGCTAGTTTTTTTAGCAATGTACCAATGTCATGTGTTCAATTATCAGGACCAAGATGAGATCAAGTGCAATGCAAGTACCTAAATCACTCATGCATACGTGCTTTTTTAATGTTTAGTTTCAATTCTATGAACATCAATTTTGCTTGTCATTGCGTTTAGATTCAGAAATCCTATGGAATTTTGCACATACCAAATGCAGAATTGAACTTGTCTTTCTTCTGCC

>PvRGL080

TGCCTGAATAGTGAATTCTGCATATTGATTATTGATGATTTATGAATATAATATTTGGAAGTTGTTAGTAAAACAGAATCCTAATGACAAAATCATACACAAAAATAGCTATAGCACCAACTGCGACAGAGAAAAGGAAGTGTTATGCCTTTAGTATTCACATTGCTGGAGGCTATCCACCCCAAAGTAAAGTATATTTCAGGGGATGACATTTACCAACAAGCTGTTTGACGAAGGTGAGCTGTGTCCACTCGACCAATGAATAGGATCATGCACTGATACAGGAGAACTGTCCTTCAGCTCCACAGAATGAAAAAATCTCTCAATCTTCTGCGGAACTATCAATCTCGCACTCCCTTGGCCCGCCTTTTTTATACCACTCAGTGACTTCTCACTTCCATTGCGAGGTGATGAACAAGTGGACCCATTCGATGAAGCCATGGCAGTTTCCTCCATAGATGCCCATCCACTGCGTTTAATGTGTTCTAGCTCCTCTGCCACTTCCATCATAGTAGGCCTCATGTCACTATGAAAAGCAAGGCATCTAAATGCAAGCTCAGCTACCTTATGAATAGAATATAGTGTCCAAGCATCTCTATGTGGTTCAAGGAAGGGGTCTATGATCTCATCTACAGCACCCCTCCTAATCCTATCTACGGCAAGTGCAGCTAAGTTAACCTCAGTCCGAGGTCGAGAAAAATCAACCGCTTTCATGGCAGTTATTATCTCTACCAAGACAACTCCAAAACTGTACACATCACTTTTATCAGAAAGATGGAAGTTCTGGTGATATTGGGGATCGACATAACCCGGAGTCCCTTGTGGAGCGGTTGAGATATGAGATGTTTCTGTCAAGCCAAGCCTAGAAAGCCCAAAATCAGCTATCTTTGATTTGAATTGATAATCCAAAAGTATATTACTAGATTTTATGTCTCTGTGGTAAATTGGGGGATGGATTTCGGAATGAAGATATGCTATAGCGTTAGCAGTTTCAGTAGCAATGGTAAGTCTTACTGTCCACGGTAGTCCTTCACTCCTCTCTCTCTGCAAGTGCTGTGAAAGTGTTCCATTTTGCATAAATTCATAAACAAGGATCTGTTCTCCCTTCTCTACGCAGCAACCTAAGAGGCGAACTAAATTTGGGTGACTCACTGAAGTAAGGAGCTTGATCTCGTTCATGACTTGGTCAGCACTGTTGGTGTCTCTTTGCCTTAACTTCTTTATAGCAACCCATTCATCATTGTGCAGTTTCCCGGCATAAACTGTACCAAATGCCCCAGTTCCCAGCCTGTGTTTCTCAGAGAAAGAATTTGTGGCTTTTTCTACTTCTTTGTAGGGATAGAAAGGTATGCTTGAGTTGCCTGCAGCTTCACGTAATTGGCGCTTTACTCTGGTATGTTTTCCCAACCAAATAGAACGGCGCCTAGCACAGTAGCATAGAAGAAACAGACCAGCCATCACCAAAGCTCCAGCTATGATTGC

>PvRGL081

GGCAAGGGTGGTTTTGGAAATGTCTACAAGGGAATTCTCCCAGATGGCACTCTTGTGGCAGTCAAGAGGCTTAAAGATGGCAGTGCCACTGGAGGAGATATTCAATTTCAGACTGAAGTTGAAATGATCAGCTTGGCGGTGCACCGAAACCTCCTCAAACTGTATGGATTTTGCATGACACCAACAGAAAGGCTTTTGGTTTATCCATACATGTCCAATGGCAGTGTTTCTTCCCGTCTCAAGGGTAAACCAGTGCTGGACTGGGGCACTAGGAAGCAAATTGCCATGGGAGCAGCAAGGGGACTGCTGTACCTTCATGAGCAATGTGATCCAAAGATAATCCACAGGGATGTGAAGGCTGCCAATATATTGCTTGATGACTATTGTGAGGCTGTAGGGGATTTTGGGTTGGCAAAGCTTTTGGATCACCAAGATTCACATGTTACAACTGCAGTTAGGGGCACAGTGGGGCATATTGCCCCAGAGTACCTTCCACAGGGCAATCTTCTGAGAAGACTGACGTTTTTGGATTTGGCATTCTCCTTCTTGAATTGATCACAGGCCAGAGGGCTCTGGAATTTGGAAAAGCAGCCAACCAAAAAGGAGCCATGCTTGATTGGGTAAGGAAAATCCATCAAGAGAAGAAGCTTGAGTTGCTTGTGGACAAGGATCTCATGAACAACTATGACAGGATTGAGCTTGAGGAAATTGTTCAAGTGGCACTCTTGTGCACACAGTATCTTCCTGGCCATAGGCCCCGAATNTCTGAAGTTGTACGAATGCTTGAAGGTGATGGCCTTGCAGAGAAATGGGAAGCTTCTCAAAGTGCTGACACCACCAAATGCAACAAACCACATGAACTCTCTTCATCAGATAGGTATTCTGACCTCACTGATGACTCTTCTTTGTTGGTTCAAGCAATGGAACTCTCAGGCCCCAGATGAATTTTTCATAGTCATTTTTAGCTCTTTTGCTTTCACTTTGCTTTTCTTTTTCATTTGGGTTTCTAGCTTCCTAAAGAAAGTATATGAAATTGAAGAACATGTGATCATTGAGTCCATTTTTATATGTTGTATGATAGAAGAAGCCTTCAAAGCTGAAAAGGGCTTTTTTTTTTGTTTTGTTTTGCTTTTCAAATGTATATATACTTATTTGTTGAAGATGATTTTGTAGTGCAAAGTTTTGTAAAGTTCAGAGAAATTAAATGACAACCTTTGCCAGGTTTTCAGTGA

>PvRGL082

GACCTTACCAGATCGTTCACCGTGTAAAAGTCCCTGGTCTTCTTCCTCTGAGGAAAATCCATTTGAAAGGAGCATCAGCGATATTGATGATGAGAGCTCCAAATTTTCTTCAGCTCCATCTGTTGAGCTACCAAATGGCCTAGAGGAGATGCTACGCGTAAATTCAATCGACTGCAAATGGTTCAGCCTTGAAGTTCTGCAGTCTTGCACTAGGCAATTTTCTTCAGAAAACTTGGTTGGGAAAGGAGGGAGCAATCGTGTGTACAAAGGAGTCCTGCCTGATGGAAAGCCTGTGGCAGTTAAAGTATTGCATTCATCAAAAGAAGCCTGGAAAGATTTTGCCCTTGAGGTGGAAATAATATCCCCATTGAGGCACAAAAACATTACCCCTCTACTTGGGATTTGCACAGAGAACAATACTTTAATCTCCGTTTATGATTATTTGCCCCAGGGCAGCTTAGAGGAAAATCTGCATGGAAAGAGCAAATTGTCATGGGAAATGAGATTTAATGTGGCAGTTAGGATCGCTGAAGCCCTTGATTACATACACAGGGAAGCTTTGAAGCCTGTTATACATAGAGATGTCAAGTCTTCTAACATTCTTCTTACCCAAGGGTTTGAACCTCTGTTATCTGATTTCGGACTTGCAACATGGGGACCAACAACTTCATCCTTTTTGACTCAAGATGTAGTAGGAACATTTGGTTATCTTGCTCCTGAATACTTCATGTATGGAAAGGTCAGTGACAAGATAGATGTTTATGCCTTTGGTATAGTTCTACTTGAACTAATATCAGGAAGGGAACCAATCAGCTCAGCAGCTTGCAAAGGACAGGAGAGCTTGATCGTGTGGGCAAAACCAATAATAGATAGTGGGGACATTAAAGGCTTATTGGACCCAGAATTAGAAGGGAAATATGATGAGGTACAATTGCAGAGAATGGTTATGGCAGCATCTTTATGCATCACACGGGCTGCTCGACTTCGTCCTAAACTGAATCAGATACTGAAGATCCTAAAAGGGGATGAGAAAGTTGAATATTTGCTGAACTCGCATGGGAACAATGAGGAGGATTCAGAAAATGGAGAAAACATTGATGATGAAGTGTATCCAAGTTCAAGTGCAGAGTTACACCTGAGTCTTGCTTTACTTGGTGTGGACGAAGACAATACGTCACAAACTAGCACTGAGCTCAGCTATAGTGAGCACTTGAAAGAACAATGGAGCAGGTCATCAAGTTTCAATTAGTTTTGTTGAATTCTAAGTTCCTAACTCTTGTACTTGTCATTAGTTTATGGATGTACAGTTACGTTAAAAAAAGCACAGAACACTAAGGATCAGCACCCTTGATGAGGTGGTTTGTAGATGTAGAATGGCGATTTTTG

>PvRGL083

TCGACGAAAGTAGGAGATAACACATGATATTATGTTATACTTCCTTGGTTGAAAAAGCCAAATTTTACAGAGCTTCATTACTATACAAACGACCAGGAGAAAGAAGAATTGACAACTATGGAATGCTGGTACACCACTAGAACAATTGCCAAGACAGAAGGAACATGAATCATGAATCCAACAATTTTCTTTTAAAATAGAAACTAAGGGAAAAGGAAGTGAATATACCATCTTGGCTCCAAATGGAAAATAGGAGCAGTTATGTGCACCAGAAGTAGAACAAAATTTTACAACATTTTTAGCTACTATGCTCTGAAGGAAGAACACCACCATAAGACAACCAGTATTAACATCTACAAATCTATATCTTGCAGTGTGTTGTAAGATGTTTGAAATTTGCACCAGACTACCATAACTAACAAACATGGAGCTCCATATCACTATCCCCTCTTGGTTCTAGGAGGAGTACCAGGAGCATGGCGAGAGCTTTGTACAGTCTGTGTACTGGGATCATATTTTTGTGACGCAAGGTAACTCAAAGCAGTGACAACATCAGCTATAACTGGACGCATATTGGCCTGCTCCTGAACACACATAGCTGCAACAGCAAGAGCCTGGTATAGCCCTCTTGAAGGATATTGACCTTGGAGCATTGGATCAGCCATTTGTGAGAATTTTCTTCGATCTTTGAACAAGGGTCTGGCCCAAGCAACAAGATTCTGCTCTCCTGCAGATCTTGAATTGTCAATTGCTTTCCTTCCAGTAATTATTTCCAGAAGAACAACACCAAAGCTATAAACATCTGATTTCAGAGTCAGTTGACCTGTCATTGCATACTCTGGAGCACAATATCCATAGGTTCCCATAACCCTTGTAGATACATGGGTGTTTTCCCCCACGGGACCAAGTTTAGCCAGACCAAAATCAGATAACTTAGGATGATATCCTTCACCAAGCAAAATATTGGAGCATTTTAAATCTCGGTATATGACAGGAGGATTAGCTTTGTCGTGTAAATATTCCAATCCCCTTGCAGCTCCAGCAGCTATTTTCATCCGTATGTTCCAATCAAGTTGTTTCTTCCCAGGAGAAATATCATGTAAGTGGTCTTCCAATGATCCTAATGACATAAATTCATAAACTAGAAGCCTTTGATCCCCATCAGCACAATAACCAATCAGGTTGACAAGGTTAGGGTGGTGAAGAAGACTTAACATCAAAACTTCAACAAGGAACTCTCTATTCCCTTGTAGTCCATTTCGGTCAAGTTGTTTAATTGCAACAATTTGATTAATACTTTCCAAACGCCCCTTGTAGACTCTTCCAAAGCCTCCCTCGCCTAAAAGACATTCAGCTCTGAAGTTTCTAGTAGCAGTTGCCAACTCACGGAATGAGAATGTCTGTGCAGCAATGTGATCAGGGTTTCCATTTCTAGAGGAATCTTTGGAATTCATGGATGAATTCCTCTTCAATTTCCCTGGGGTGGCTTTGATCGGATCAACAAGACTGCTCTGCACTTCCATCTTCTCCAACTTCTTCTTTAGCTTCTTCTTAGTGTTTGAATTTCCGGAACAGGGAATCCAACCCATGTTTCAATTTCTTCTTCCCTCTTCCTTTTTCAGGCAGATACGCAGATGGTTGCTGAATAATCAATGGATCAGTCAAAATTCACATGAAAAGGAGTCACCTTTGCATTCTCTCTCTTACACTTGGTGCTGCTTTAATGTCATCATAATCATCGGTGTCTCTAGTGATTGTGAGGTTGCCTACGATTGGTTTT

>PvRGL084

GATTGGTGATGATATTGCAAGGGCGGTACGGGCAATGAATGAAACACTAAAGCAAAATCGTCTTTTGAGTGAGCAAGGGGTAGTTGACAGTTCATTGTTCCATTTGCCAAATGATAGAGCCGGCAGTATGGATCTTCNNNAAAAAAAGTCAAGTTTCCATCTTGATGGGCATCAAGAGAGGTCCCCTTTGTATTCGCCCCACTGTGACCCTGTAACGTCTCAAAAGGCAATGTCATTACCCTCTTCCCCGCACGATTATAGAGGACAAGCGTCTGAGAGAAGTGAAGTGTCAGGATATCCAATGAATGATGAACTGGAGTCTACTTGGAATAAAGTCCTTGAATCACCAATGTTCAATGATAGACCTCTGTTGCCATATGAAGAATGGAATATCGATTTCACAGAATTGACTGTTGGAACTCGCGTTGGGATTGGGTTTCTTTGGAGAAGTTTTCCGTGGCATATGGAATGGCACAGATGTTGCAATCAAGGTTTTTCTAGAGCAAGATTTAACTGCTGAAAATATGGAAGATTTTTGTAATGAGATATCTATTCTGAGCCGACTCCGACATCCTAATGTTATCTTGTTTCTCGGTGCATGCACAAAGCCTCCACGCTTGTCAATGATTACAGAATATATGGAATTGGGATCGTTGTATTACTTGATACATTTAAGTGGTCAAAAGAAAAAGCTTAATTGGCGGAGAAGACTAAGGATGTTACGTGACATATGCAAGGGTTTGATGTGCATACATCGAATGAAGATTGTTCACCGTGATCTGAAAAGCGCAAATTGTCTGGTGAATAAGCATTGGACTGTGAAAATATGTGATTTTGGGCTTTCAAGATTAATGACAGAGTCTCCCATGAGAGATTCTTCCTCGGCTGGAACTCCAGAATGGATGGCTCCTGAGCTCATTCGAAACGAACCATTCACAGAAAAATGTGATATCTTTAGCCTTGGGGTGATAATGTGGGAACTCTGCACCTTGAGCAGACCATGGGAAGGAGTACCACCTGAGAGGGTGGTTTATTCCGTTGCAAATGAGGCTTCCAGATTGGAAATACCAGAAGGCCCTCTAGGAAGGCTGATTTCAGAATGTTGGGCAGAATCTCTTGAGAGGCCAAGTTGTGAGGAGATTCTTTCTCGTTTGATGGACATCGAGTACTCCTTGTGCTGAAGCAATATCTTTTGTACATAGAAAACTTTGGGTAGAGTTTTTACGTTGTATTATCGGCTGATGTTGTGGACTATTTGTGAATTTTGTTCGTTTTCGCAGAACATGCGAAATATGAATCCATATTTTAATGTGCTGCAACTCCATCTCCTTCTGGGCCAATGAGAAAGACGAGATCAATCTATCTCGTATTTTATGTGCAATGAAATTAAAAACAGTGTCTCATGGAGAATAGTTATTCCCGATCC

>PvRGL085

CGGGAACCTTATCAGGGTTCGTGTTTTCTCTGTTGTTCAAGCTGGCTTTGGCGTTGATCAAAGGAAGAGGAAAGAGAGCGGGTCCTGATATTTTTAGTCCCTTGATTAAGAAAGCAGAGGATTTGGCCTTCTTGGAGAAAGAAGAAGGGCTCGCTTCGATGGAAATCATTGGACGCGGTGGCTGTGGAGAGGTTTACAAGGCTGAGTTACCAGGAAGCGGTGGCAAAATGATTGCTATAAAGAAAGTGATTCAACCTCCCAAAGATGCTGCTGAATTGGCAGAGGAGATAGTAAGATTATGCATAAGAAAATGAGGCAAATTCGGTCTGAGATTACAACTGTGGGACAGATTAGACACCGAAACCTTCTGCCTCTGTTAGCGCATGTTTCTAGGCCTGATTGCCACTACCTAGTGTATGAATTCATGAAAAATGGTAGCTTGTTGGACTTGCTGGACAAGGTTCAAAAGGGTGAGGGAGAGTTGGATTGGTTGGCGCGGCATAGAATCATGCTTGGTGTTGCTGCTGGACTTGAATACCTTCACATGAGTCACAACCCTCGCATCATTCATAGGGATCTCAAGCCTGGAAATATTCTTCTCGATGATGACATGGAAGCGCGCATAGCTGATTTTGGACTGGCTAAGGCAATGCCGGATTATAAGACTCACATAACCACCTCCAATGTTGCTGGCACTGTGGGGTACATTGCTCCTGAGTATCACCAGATACTTAAGTTCACTGATAAATGTGATATCTACAGCTTTGGGGTTATCCTTGGGGTGTTGGTGATTGGGAAGCTTCCTTCCGATGAGTTCTTTCAGCATACTGAAGAGATTAGTTTGGTCAAGTGGTTGAGGAAGGTTTTGAGTTCTGAGAATCCCAAGGAGGCTATTGATACAAAGCTGCTCGGAAATGGGTATGAGGAGCAGATGCTTCTGGTTTTGAAGATTGCATGCTTTTGCACCATGGATGATCCCAAGGAGAGGCCTAACAGCAAGGAAGTTAGGTGCATGCTGTCTCAGATCAAACACTGATCAATCTGTTGGATAAGTACACGTACGTACCATATTTGTGTTAACGTCTTCGTGTTTAAGAATATAGTTCATAGCTGAGTGATCAATGTTGCTTCATAAGGTAAATAATGAAGCCGTGTAATATTGTTTCCTTCATCACAGATTTGCTGAATTTCCAGAAATGGAAAGGATATAGAAAGAAAAATTCAGCATTGCA

>PvRGL086

TGGACAACTTTCCACTGCCACATAACAAAAGGGAACTGTAATTTGTGGTATGCAATAAGAAAACATAGGAGGGTAATCACATTCAGCTTGATGGTTTTGGAAGATGACAATCAAACTACAATTTAACTGAAACTAGAGTTACACGATTTCTATTTCCTTTATGGGGGTTGGGAGAAATAATTAACACAGGATGAACTAAGTCATATAATATGGAACTGCAGCAATAGAAAGAAAAAGGAAGTGAAGGTGCCAGTGAAACTGAGCTGGCCCTACTTATGGACTTCATTACAACTTCTCCAACATGGGCACACATCTACAGTAGTAATCCATACCTGGATATGGAGCCAAAATGTACAAGCACTCGCAGCTCCTCTTTCCAAGGAAATTATGGAGAAACTCATGATTTGCATGTTCAAATATGATCCTCTTGCTTTGCTGTTTGTAGCAACTGCAGTAACCACATCTTCATTCGCAAATTCGTCAAAGACCAAATTACGCCAAATACCTGAACAGATTTGCGTTATCTTTGTCTCTTTCCAAGTAGTCTCGAGAAATCAAATCTTCAATCCGCTTCTTAATTGCCTTCACATCGGGCTTAAACATGCGACCTAACTGCTCAACACACTCCATTACTAACTGTTGGTAATTTAAAACTTTCCGACTCTTCATGATACGCACGATTGAGGCATCAATAGCATATCTTCTGTCCTTGTCAACATCCTCAATTACTTTTTTCTTCTCATCAACGGGAGGAAGAGGAATCTTGATCCTCCTCATTTTGTCCGTAAATTTCGAATTAAATTCAAAATAATCTGTAGAAGATATTGTTTTTGTGTTTGGTTCCTTGTTAAGAATCTTGTATTTGGCACATGACAAGGAGTGGAGCAGTCTAATAACATCATCATCAGATAAATTTAACTGAGTCATTATCTCTGAATAACTCAATCTGTCCGAGGAATTAAATAGAAGCAATGCTGAAGCCTGGTAAGTTGTTACAATCAGCTCCACAGTTTTTGGATCAAACTTCCCATTTATGTTGCAGGTTCCCAAGGAATAAATCCATGTAAGTTTTCTGTGCTTAGTTTTTGTTTGATAAAATTCCTTGAAAACTTCAACGCATCTAACCATTTCTGCTGGAAGATTGAGGTCAAAAGATTTGTAACTAGGCCAGAAGCCAGTAGTCAGAACTGTAACTGTCAAGTCTATTCCAGGGTCGGCATTAGGATTATTGCTTAAATACTCCTCAAAGCTTGTTTGGTTCTCCTTTGCAAGTGTCAAATCTGTAACCATTCCTTCCATCTTCGATGTAAATTGACCACCACATTGTTGCTTTAGCTTTGTTAAAATGCTTCTCTCATGGTCATCATTGGCACTCTTGTCAAACAGGAGCCTTCGAGCTAGTTTCTTCCTATAAAATTCAGCAAACAAATCTTTGTCGCTGATATAGGCAAGCAACTTGACTACCTTTTCAAGAGTTTCTTCAATTGCTTCATCACTCAATTTTTCCTTCCTCTTTCTAAAGTCTGCCTCTTTCTTTCTTACTTTCTCTCACTCAACAAAAAAATTGCTCACAAAGGAGGAATAATGGCACCTTTCATTTCCCTCTCTTTGTTGTTTCTCTTTCTCCATTACCCCCTGCTATCCCTCTCAACCAACCCTGAAGGGAATGCTCTGCATGCTTTGAGAAGCAGGCTTTCTGATCCCAACAATGTGTTGCAGAGTTGGGACCCAACACTGGTTAATCCCTGTACTTGGTTCCATGTTACCTGTGATTCCAACAATCATGTGATTCGCTTAGACTTGGGCAACTCTAACGTTTCTGGGATTCTGGGCCCAGAACTTGGCCAGCTACAGCATCTTCAGTACTTGGAGCTCTATAGAAATGACTTAACAGGAAAGATTCCTGAGGAACTTGGTAACTTGAAGTCACTAATTAGCATGGATTTGTATGATAACAAGTTGGAGGGGAAGATTCCAAAGTCCTTCGGAAAGTTGAAATCACTTAAGTTCCTGCGGCTAAACAGCAACAAGTTGACAGGATCCATCCCAAGAGAACTCACTCATCTCACTGATCTCAAAATCCTGGATGTTTCTGATAACGACCTCTGTGGAACAATACCAGTAGACGGCAACTTCGAATCTTTCCCAATGGAAAGTTTTGAAAACAACAGTTTTAGTGGTCCTGAACTGAAAGGACTTGTTCCATATGATTTTGGATGCTGAAGAAATATACAAGTCGCAATGAAGAGGCTTAGTTTATGTAGGGAGGGAGTATCAGAAAGGGAAAGGGTGGTGTTATTTGCCCATCAAAATTTCCTCACCATTTTTACACTTTTTAGTTGGGAAAAAGTTGAAATCTATGCTTTTCTTATTTGATTATAAAAAGAAGTGATGTAAGAACATTTGTGTGTAAATAACATACATTATTAATGTTGTATGCTTGACCCGAATATCAATCTAAAAATGTCAGATTTCTTGTGACAAAAAAAAAA

>PvRGL087

AGTTTTGAATATCAAGAGAGGTCAAGGGATGGTTGATCAAGGCGATTTAGTTGTCATATGTGTCTCTATTGGAGTGGCCCTTGGGGTTTTGATGGCTTGCTTAGTGTATTTTGGAATAAGGTGGTACAATAAGCGAACTCATCTTAGCCGATCTGCGAATGAGCCTAGTTTAACAGCTATCCCTATACGAACAAATGGATTAGGAACAAGCACTGACTTCAGTGCTTCCCTCACCAGTTCGGTAGCCACTTATAGATCACCAAATCCTTCAAAAAACTCTCCAGCTGGGTGGAATCATCAGAATAAAGATGCGTTTGCTTCTGTATCAGGCATTCTGAGGTACTCATACAAGGAAATTCAAAAGGCCACACAAAATTTCACAAATACTTTGGGAGAAGGGTCATTTGGCACAGTTTATAAAGCCACAATGCCTACAGGAGAGGTGGTAGCTGTGAAGATGCTTGCACCCAATTCCAAACAAGGGGAGAAAGAATTTCTAACGGAGGTGCTTCTGCTAGGAAGACTGCATCATCGGAATCTTGTGAATTTGTTAGGGTACTGCGTAGATAAAGGACAGCTTATGCTTGTTTACGAGTTCATGAGTAATGGAAGTTTAGAAAACCTTTTATATGGTGATGAAAAAGAATTGAGTTGGGATGAAAGGCTGCAAATTGCTGTTGATATTTCACACGGAGTAGAATACCTTCATGAAGGGGCAGTACCACCCATTGTACATCGTGATTTGAAATCTGCTAATATTCTGCTAGATCACTCAATGAGAGCTAAGGTTTCTGATTTTGGGCTCTCAAAGGAAGAGATCTTTGATGGCCGTAATTCTGGCCTTAAAGGTACATATGGCTACATGGATCCAGCATACATTTCCTCGAGCAAGTTCACAGTGAAGAGTGACATTTACAGTTTTGGTATAATAATTTTTGAGCTCATCACTGCCATCCACCCACATCAAAATTTGATGGAATATATCAACCTTGCTGCAATGGATTACGATGGTGTAGATGGGATTCTTGACAAGCAACTTGTGGGAAAATGCAATGTTGAAGAAGTGAGACAGCTTGCTAAAATTGCACATAAATGTTTGCACAAATCACCTAGGAAACGCCCCTCTATAGGTGAGGTTTCGCAGGCTATATTGGGGATTAGGCAAAGGCGCCTCGTGAAAGAAGACACCATGTCATTTGCAAGCACCAACTTCTCCCGAAGTGTGAGTCAAATAGAGGAACAACAGATTGAATTAAGTAGAATAATCACCATGAACCACAGGGAAATGGGGTGAACACATGTTCATATATTTTGTTGTGAAAATTCTTTTTGAGTAACTCAAGAAAATTTGTACAAAAAATCATTGAATGTCACTAGCTGCATATGTGAGTCAAGATGTACAGATAACAAGGGTGGATCTCATGCTACCCTGAAACATGATTTTGGTTTGTGAATGATCAATTGTCTTGTGAGTTCTTTTGTAGCTAAATAACAAATAAGTTTTACATTGGACCA

>PvRGL088

GGTCTCTATGTTTGTGAAACCCGAACGCGCTCACTGTTTTCAATTGCTCTGCTCTGCATGAAACAGAGTACCCTGTTGGATTGTTCATTGCAATGAGGTATATTCGGAGCAACAGCTTCAAGAGGCTCTTCTCCTTTGGAAGACGAGGTTCAGGGAACAAGTTCTGAGTCCTAAAGGTGGAGAAAGTCATGAGACTCTTCCGTATAAAGAAGAACCTTATGGAAGACCCTCTTGGAAGTGCTTCTCTTATGAAGACTTGTTTGATGCCACAAATGGCTTCAGCTCAGAAAATTTGGTTGGAAAAGGAGGGTATGCGGAGGTTTACAAGGGAACAGTGAATGGGGGTGAGGAAATTGCTGTGAAGAGACTCACAAGAACTTCGAGGGATGAGAGAAAGGAGAAAGAGTTTTTGACAGAGATTGGTACAATTGGTCATGTGAACCATTCCAATGTGTTGCCTCTTCTGGGGTGTTGTATTGACAATGGCCTTTACCTTGTTTTTGAGCTATCTTCTAGAGGTTCTGCTGCATCACTTCTTCATGATGAAAAGTTGCCCCCTTTAGATTGGAAAACGAGGCACAAGATAGCTATTGGAACTGCACGCGGCCTCCACTACTTGCATAAGGGCTGCAAGAGGAGGATAATCCACCGGGACATTAAGGCGTCAAACATTTTGTTAACGAAGGATTTTGAGCCACAGATATCTGATTTTGGACTAGCAAAATGGCTTCCATCTCAATGGACTCATCACTCCATTGCCCCAATAGAAGGGACATTTGGACATTTGGCCCCAGAGTACTACTTGCATGGAGTTGTGGATGAGAAGACAGATGTGTTTGCCTTTGGTGTGTTCTTGCTAGAAGTAATCTCAGGCAGAAAACCAGTAGATGGGTCACACCAAAGCTTACACAGCTGGGCAAAACCAATTTTGAACAAGGGTGGGATTGAAGAGTTGGTAGATCCAAGGCTTGAAGGGGCTTATGATGTGACAGAGTTAAAAAGATTTGCCTTTGCTGCATCTCTTTGCATTCGGGCTTCTTCAACTTGGCGACCTACCATGACTGAGGTACTGGAGATAATGGAAAAGGGAGAAAAAGATACAGAAAAGTGGAAAATGCCAGAGGAAGAAGAAGAAGAGGAGGAGGAGTTCTGGGGTTTTGAGGATCTTGAGTATGAATATGATAGCTCCTTTTCAATGTCATTACTTGATTCCATTGGAAGCACTACTTAGGAATCACTTGCCTCTTTCAATTGCTTGTATATATACAAATTTCATGGAGAGGAGTTCATGTGGCTATGCCACTCCTTCCTATCTAATCAATCTCTTGTAACATTTTTCTTTCAATCTATTTTTGTTTTTGTGGATGTGAAATATTGATTGTAATTGAATCCAATTATGTCGGCAACTCTTAGAGGAAAATGACACACCAAGGTTGTTCATGGACTAACCAATTTCAATCATATTATAGTTTTGTGAAACAAGGCATGGTGATTGGGTCCA

>PvRGL089

GGAGGGTTTGGATCTGTTCACAGAGGGGTTCTACCTGAAGGACAAGTCATTGCTGTGAAGCAACATAAATTAGCTAGCTCTCAAGGTGATCTTGAATTTTGTTCAGAGGTGGAAGTCCTGAGCTGTGCTCAACACCGGAATGTTGTTATGCTCATTGGCTTCTGTATAGAGGATAAGAGAAGGCTACTGGTTTATGAGTACATATGCAATGGATCATTGGATTCGCATTTATATGGGCGACAAAAGGATCCATTAGAGTGGTCAGCACGGCAAAAAATTGCTGTGGGAGCAGCCCGTGGATTACGATATCTACATGAAGAATGCAGAGTGGGTTGTATTATCCACCGTGACATGAGGCCAAACAACATTCTCATAACTCACGATTTTGAACCTCTGGTTGGTGATTTTGGACTGGCAAGGTGGCAGCCTGATGGAGACACAGGTGTGGAAACTAGAGTGATTGGAACATTTGGGTATTTGGCTCCTGAATATGCTCAAAGTGGTCAAATCACTGAAAAAGCTGATGTTTATTCCTTTGGGGTGGTATTAGTTGAGCTTGTTACAGGGAGAAAAGCTGTGGATCTCACTAGGCCAAAGGGACAGCAGTGCCTTACGGAGTGGGCACGACCTCTCTTAGAAGAATATGCAACTGAGGAACTGATTGATCCAAGACTTGATAACCACTATTCTGAAAATGAGGTCTATTGCATGCTGCATGCTGCCTCATTATGCATACAGCGAGATCCTCAATGTAGACCACGCATGTCACAGGTCCTCCGAATACTAGAGGGTGACATGGTGATGGACACAAATTACATCTCAACTCCAGGCTATGATGCAGGAAACAGAAGTGGTAGATTGTGGTCAGAGCCATTACAAAGGCAGCACCATTATAGTGGTCCCCTGTTAGAAGAGTCTGTAGAGTCCTTCAGTGGGAAGCTATCCCTTGACAAGTACAGGCCTTCCTATTGGGGTGATAGGGACAAGGCTAGGAGGGCCTCATGTGAAGATGACATTTGAATTGATTGATCATAAAGTTTGGGTTCTTGTTATGTTAAATACAAAGTATGCTTGTTCCACACCTATTTTGTCTGTTTTTTTAATCCTTTTTTTGTGTGCAATGGATTTAATATTGCATTTGTATAGCAGAGTGAAAGTAGAATAGGTAAATATAGAAGCTTTTGAAGAATTTTCAGGAAATCCTGTTAGTGTCTCAATGACCTTGATAGAATCAGTGCTGCTGTGCTGTCTACTATCTT

>PvRGL090

GTTTTGGCATTGTTTCCTTGGTTTCAGTGTTGATAGTGTGGGTAATATCCAAGAGGAGGATCACTCCTGGTGGAGACGCTGACAAGATTGAACTAGAATCGATTTCTATCAACTCTTACAGTGGGGTTCATCCTGAGGTTGCAAAGAGGCTAGCCAAGTGGTACTGTTCCCTAATAAAACAAGTGAAATCAAGGACCTTACCATCGTTGAAATATTAAAAGCTACTGAAAACTTCAGTCAGGCAAACATAATAGGATGTGGGGTTTTGGTTTGGTTTATAAAGCAACATTACCCAATGGCACTGCTCTGGCCATCAAGAAACTTTCAGGAGATTTGGGTATTATGGAAAGGGAATTTAAAGCAGAAGTAGAGGCTTTGTCTACAGCACAGCATGAAAATCTGGTTGCACTGCAAGGCTACTGTGTGCATGAGGGTGTTCGGCTTCTCATATACTCCTACATGGAAAATGGAAGTCTGGATTACTGGTTGCATGAGAAGGCTGATGGTCCATCTCAGATTGATTGGCCTACTCGACTGAAGATTGCACAAGGTGCAAGTTTTGGATTGGCTTACATGCATCAAATATGTGACCCACATATAGTGCATCGTGATATAAAGTCGAGCAATATACTTCTTGATGAAAAGTTTGAGGCACATGTTGCTGATTTTGGGCTGGCTCGACTCATTCTTCCTTATCATACTCATGTTACAACTGAACTTGTAGGTACATTAGGTTACATACCCCCAGAGTATGGACAAGCATGGGTGGCAACTCTGAGAGGAGATGTGTACAGCTTTGGGGTTGTCATGCTTGAGCTGCTAACGGGGAGGAGGCCTGTGGATGTAAGCAAGCCAAAAATGTCAAGGGAGTTAGTTTCCTGGGTTCAACATATGAGGAGTGAAGGAAAACAAGATCAAGTCTTTGATCCATTATGAGAGGCAAGGGCTTTGAAGAAGAGATGCTGCAGGTTCTTGATGTAGCCTGCATGTGTGTCAACCACAATCCTTTCAAGAGACCGAGCATCAGAGAGGTTGTTGAATGGCTGAGGAATGTAGCATTATCCAACCAGCCATTGAATAAAGACTAGAAATAAATGTCTGTCCTTGACAAATATTGACAATAGACATGTATACTTGTGCAAAAATGTTCCAACTCAATATATACAGAATCATTTTTACCTCTGT

>PvRGL091

CAATAATTTTTAGACAAGTTTATTTATTTATTTATTTGAGTGAACCCATTATTTAAAACCAAAATAAAACACCTCAAAACTTAAAACAGAAACTTACTTAATGTAATACATTATTAACTAGGCTTTTTGCAAGGGTTCAGAGGAGAACCGCAGAGACAATCGTTGCCGACAAAAGCACTTGCCGGAAACGTGTTTTTGGGAATCTGACCGCAGAGATGGTTATAACTCACGTTCAACTTCTGAAGCCCAACGACGCTGTTCGGAACCTTGCCAAACACGGCGTTATGCGACAAATCCAAAACCCTAAACCTTTCCCCAAACTTCAATTTCCCCAAATCAAACTTCAAGTGTTCCTCGCCGCCCAAAACCCCACCAAATACTGCGTGCTGTTCACCAAACTTATCGCGTTCCCCGATATCTCGTTTCCCGAGAGATCGATGTAATCGTAGAAGAACGTCTCCGAAGGCTTGAAATCCTCGATTTTCATCTTGATCCCGCAGTTCGCGAGCTTCAGGGAGAAGATTATCGGCGACGAGAACACCCACTTCGGAATCTTCCCCAGGTGGAAGCTGTTGTTCGACAAGTCCAGCGATTCGATCCCCTTCACGTTCATCTCGGGAAACGGGTCCACGAGTAAATTGTTCGAGAGGTCAAGGTTGAAGATCTTGGTCAAATTCGCAAAACTCGCCGGCACGGTTCCGGTGAACCTGTTCCACGACAAGTCCAGCGTGTCCAGCGCCTTGAATTTTCCCAAAAAATCGGGGATTCTGCCGGAAAACGAGTTGTGCCCTAGCTCCAGGTAAGCCAAATTGGACGCCAATTTCGAAATCGACGCCGGAATGCCGCCGGAAAAATTGTTCCGGGAGAGTCTGAGACTCCGGAGGTTGGAGGAAAACGCAGGTATGGTTCCTTGGAAGTGGTTCCCTTCGAGGGAGAGGTAGTTCAGGTTAACGAGTTTGTTGAGGGCTTTGGGGAGTGTTCCGGAGAGCGAGTTGTTGTCGAGTTTGAGCTGAGTCAGGTGAGTCAACTCGGTGATCGAACTCGGTAGGGTTCCGGTGAAGCGGTTACCCACCAAACTGAGCGCGTATAGTTTGGTGAGGTTGCCAATGTTCTCGGGGATCCGACCCGAAAGGTTGTTGTTTTCCAAATAGACGTATTCGATGTTGGGAAGTTGGAAGAGGAAAGTGGGAAAAGGACCCGAAATGTTTTTGAGGTCGAAAAGGTAGAGTCCATCAAGAAGTCGGAGCTTCGATAAGGTGGGTGAGATCGTACCCGATAAGATGGATTTGGGGTTTTCATCGGGTTGACCCGTTAGGGAGATTCTCTGGACCCGGGTACTGTTGAAGTGACACTCTACTCCGTTCCACTTGCAGCAGTCCGTACCCGGTATCCAGTTTTTGAGGATGCCGGACGGGTCGGATCGGATGGCGGATTTGAAGCCCAAGAGGCCCGCTTCCTCGTCGGGGAGACACGTGGCGGCGGTGGCCTTGTGGGGTGTGAAGAAGATTGTGAGGAAGATGAAGATGAAGATGGTGGTGATGGCTAAGGGAGAGTTGAAGAGATTCATTTTGGGGTATGAGAGTTTTGAAGTGAAAAGGGTTTTGAAAGTGTCTTTAATG

>PvRGL092

TGGATCATGTTAATTTTTTATTTTATTTTTGACATTTCAAGGTATATTTGATTGCTGATGTTAGGTTATTTTTNNNANCNANNNTNNNTNAANNNTTNNTNGTCGTAAATGATATTTTTTAACTAGTATCATTTTCAAAATGTGACTAATTCAGTAAAATTAAGAAAAAGATACATAGAGCTGACAAACCTCTATCTGGTGCACATCATAACGTAAGTGTCTACTAATTTACGTTTCATATACCTCGACAATATGCTAGCAATGCAGAACATAGGATAAAAAATTGGCTATTGGAAATTGTTCATAGCTGCAAACTTAGAGCAATGTTTGTTTCTTTTCCTCCTTCTAACCTTGATAGCTTGGATGCTTTCCAATGTGTGAAGAACATCTTTCATAGAAGGACGTTTATTACGGTCATTTTGAAGGCATTCCAGAGTAAGTTGTGCTGCTTTCAGTGCTGCTTTGGTTGAATACTGACCCTCAATCTTCTCATCCATAATATCACTTTTCAACTTGGTTTTATCAGATAGATAAGGCTTCATCCACTCAACCAGATTTTCCTGCCCTGAAGGGCGATTTACATCAAGTGCCCGCATCCCTTTCAGCAGTTCAAGCAGCACCACACCAAATCCATAAACATCACTTTTCACATAAAGGTGACCTGTTGCAATGTATTCGGGAGCAGCATACCCATATGTTCCAATGATCCTGGTAGTCACGTGTGAATCTCCCACAGAAGGGCCTAATTTCGCCAAACCAAAATCTGATATTTTCGCATTGAACTCCTCATCGAGTAGTATATTGGAGGCTTTGAAATCTCTATATATGATTTGCTTCTCTGAGGTGTGCAAGTAAGCCAAGCCCCGAGCTGCACCAATAGCTATTTTGAGTCGAGTGTCCCAAGACAGTGGTACTGTGTTACCACTCCAAAATAGATGAGTCTCCAAGCTTCCCTTTGGCATGAATTCGTACACAAGGAGAAACTCCACATCGTCACAGCAGTAACCCAAAAGCTTCACCAGGTTTGGGTGAGAAACTCTTCCTAAAAAATTTATTTCTGACTGCCACTCTTGAAGGCCCTGCATACTTTCGGGATTCAACTTTTTGATAGCTACCATGATTCCAGAACCAGCTTTGGTTGGTGTCAGAGTTTTCTCATGCAACCATCCTTTGTAAACTTTCCCAAAACCTCCTTCTCCAAGCAACGCATCCGACTTGAAACTCTTCGTGGCTGCTTTCAGCTCCGCGAAACTAAACTCCTTCAACCTCCTCATTTCCTCACTCGGAAAAACAATGCCACTGCCATTACTACTGCTTCCGACAACTCCAGACTCCTCCGAAACTTCACTCTTTCCTTCTACGCTCTCCAAGGATACTGTCAAGTCTCTACTCC

>PvRGL093

TAGAAATGGACATGTATTGTATTGAATCGAATTTATAAAATACATACATTCAAATAAATTGATCTCACAAACTTAAAGTGCTATGCATTTTAATTTTATCAACCCCTAAAAAAGTACACATTCTTCAATCTAAAACTTCAAGAGTTCAGTTATTCTACCTAAATTAAACTTTGAGCTAACATTGAATTCCTAACTGTTAATTACACAAGAATTGGTAGCTAACTACATATTGATGCAACAGATATCTATGTTTCAGACCTTGACAATATGTTGCAAAATAAAGTGGTAACCAGAAGGATGATAAATTAGCTAATGAAAATTGAGCATAGTTCCAAGCATAGTACTATGTTTCTTGGATTATTATTGTCTTCTATTCTTGATAGCTTCGATGCGTTCCAATGTTTCCAGAACATCTTTCATGGGAGGACGTTTTCTATGGTCACGTTGAAGGCATTTTAGAGTTAAATGTGCTGCTCGCATTGCTGCCTTGGTTGAATACTGACCCTCTATTCTCTCATCCATTATGCTTTTCAACTTCTTTTTATCAGAAAGAGAAGGCTTAGCCCATTCAATCAGATTCTGCTGCTCTAAGGGGCGATTTCTATCAAGTGCCCTCAACCCTGTCAGAATTTCAAGCAGCACTACTCCAAAACCATAAACATCACTCTTCACATACAAGTGGCCTGTTGCAACGTATTCTGGAGCAGCATACCCATATGTGCCCATGATCCTGGTAGTCACGTGTGAATATCCCCCAGAAGGGCCTAATTTGGCCAACCCAAAATCTGAAATTTTTGCATTGTAATCCTCATCAAGTAATATATTGGAGGCTTTGAAATCTCTGTATATGATTTGCTTTTCTGAGGTGTGTAAGAAAGCCAAGCCTCTAGCTGCACCAATAGCTATTTTGATTCGGGTGTTCCAAGACAGTGTTTCTGTGCTAGTATTTCTTCTAAATAGATGATTCTCCAAGCTTCCCTTTGGCATGAGCTCATACACAAGTAGAAATTCCACATCGTCACAACAGTAACCCAAAAGCTTGACCAGGTTTGGGTGAGAAATTCTTCCTAAAAAATTGATTTCTGACTGCCACTCTCGAAATCCCTGCACACTCTCGGAATTCAATTTCTTAACAGCAACTACCATTCCAGATCCAGATTTGGTTGGTGTCAGAGTTTTCTCATCCAACCATCCTTTGTAAACTTTCCCAAAACCCCCTTCTCCAAGCAATACATCAGACCTGAAACTCTTGGTTGCCGCTTTCAGATCCGCAAAATTGAACTGCTTCAAGTTCCTCGTTTCAACTCTCGGAAACACAGCGTTATTGCCACTGATACTACTACTGCTTCCTCC

>PvRGL094

TGAATCAGTTGCTGTTAAAATCATTATGAAACCCGAAGACGATGAAAGTGGAGATTTGGCTTGTCGATTAGAGAAACAGTTCGTTAGAGAAGTTACCCTTTTATCTCGCCTTCACCATCAAAATGTTATAAAGTTCTCAGCAGCATGCAGAAAGCCACCTGTTTATTGTATCATCACAGAGTATCTTTCGGAAGGTTCCTTGAGGGCATATTTGCATAAGTTGGAGCGCCAAACTATTTCTCTAGAAAAACTATTAACTTTTGCCCTGGATATTGCTCGCGGTATGGAATATATACATTCTCAAGGTGTCATTCATCGAGACCTTAAGCCAGAGAATATCCTCATCAACGAAGACTATCGCCTTAAAATTGCTGATTTTGGCATTGCATGCGAGGAAGCCTCTTGTGACTTATTGGCTGATGACCCTGGCACCTACCGTTGGATGGCACCCGAGATGATCAAACAGAAATCCTATGGGAAAAAGGTTGATGTCTACAGTTTTGGGCTTATCTTATGGGAGATGTTGACTGGAACTATTCCATATGAGGATATGAATCCAATACAGGCTGCTTTTGCCGTTGTAAACAAGAATTCAAGGCCAGTTATTCCATCAGACTGTCCACCTGCTATGCGAGCTTTAGTTGAGCAATGTTGGAGCCTGCAACCAGATAAGAGGCCTGAGTTCTGGCAGATTGTGAAGATATTAGAGCAATTTGAATCTGCACTTGCTCGTGACGGAACCCTGAGTCTGGTGCCAAACCCTTGTTGGGATACTAAAAAAGGGGTTCTTCATTGGATTCAAAAGCTTGGCCCTGTGCAACAAAATAGTGGCCCTGTGCCTAAACCCAAGTTCACATGAATCTTTCAAGTGTCCTGGCTTTTATTGTACACCAGGTCCATTGTAATTAAAAATGAAAGGTTGGTTTTTGATGTTCTCTGGTTGTAGTCACGTTATGACATTCATGTGATAGTAAATTGATTTTTAGGCTTGCACATAAAGGTGAAAAGCTTCTTTTTTTCTTTTTACCTGAGTGCATACACAACTTATGAAACTCTTGCATCGTGTGTACCAAGAAATCATCACCCCTTTACTTCTTATGAATGTTTATAATTCTTGTGTGGA

>PvRGL095

TGACAAGGCCAACAAATTTCTCAAATAAATCTCGTAAAGAGCTATCAGATTCTGTACATACCCCAGATACTTTGGGTTCTTATAAATAACAGCCACCTTCATACACAAACAATATATATCCATCTAGAACAAATGCCTATTCTTCTACAACAAAAGAAAGGAAAGGAAAACACGAAAATTAATATTATCCCCTGCTCAGCCTCTGCCATGCCCATCTCGGGTAGGCAAATCCAAGAATCTAACCAGACAAAAATAAAATAAATCACTCACTTTATACACACCACTACTGATCCTAAAAAAGAATCTCATTCTCTTAGGCCATTCCTGGAGTTACCTTCCACTTTCTGATCTAGAACCCTGAATGTCACTCCCATCCGTGTCAGAACTATCCTTATGGGATTCAATTTCTGAGTTAACCCCGTGATTTCTTCGATGCCTACGGTCCTCTCTAGCTAAAGGGTACTCCTCTGACTCAAGCATACGCACAACTTGACCCATCTTGGGTCTTTTCTCTGAATCTGGATCTACACATCTCAAAGCAGTTAAGAGCGCCCGTTTCAAAGTCCTGGTAGATGGCTTCACCTCAATGTTTGCGTCCACCGCTTCTTCCGATCGCCTGTTTCCAACCATCATCTTCAGCCAATCAACCATATTAACTTCCTGTGCTGGGCGACCATAGTCAACTGGATCTCTTCCGGTTATTGCTTCCAATAGCACAACACCAAAGCTATAAACATCACTTTTTTCATTTAGAAGACCAGTATTTGCATATTCAGGAGCCACATATCCAAAGGTTCCCATAACTCGTGTAGCAACATGACTCTTCCCAGAACCCAGTAACTTGGCCAGGCCAAAATCAGAAACCTTGGCATTAAAGTCCTCATCAATTAATATGTTGCTTGACTTGATATCTCGGTGCACCACCTTTGGCTCAATTGCTTCATGCAAATATGCAAGCCCCTTAGCTGTGCCAAGGAGAATCTTGATACGTGCTTCCCAGGTAAGATATCCATGATGTCGCATAGCTCCGTGAAGCCATTGCTCTAAGTTTCCGTTACTGACATATTCATAGACTAACATCCTGTGAGCCCCTCCATGCAGTACCCCAAAAGTCGAACCAAATTTTTATGTCGGACATGGCCAATAGCTTCAACTTCCACTCTAAACTCTTTCTCAGCTTGACCAATGTTATTGAGTATCTTTTTAACCGCCACTGGAGTACCATTGATCAACTGTCCCC

>PvRGL096

TCAAATGAAAAGGAACGCAGAAATTTGCATAAACGAGTAAATAAAACTTAAATGTAACACATTGTTCTGGAAAAAGATCATCTACAGATATTCAATGTAGCCATAGATTAATGGATAACAGGCCAAAATATTTGTTTGCCATCAAACAAGACCGAGTCAGAAAAACTCGGGAAAGTCAGGGCACTAGATTATTACCAAGAACATTTTGCATTGTAAATATTCTCCAAAGCTCAGTGTGCTGCTTCTTTTACCAACTCTTCTTTCTTGCCCTTGCCTCCTGTGAAGAGCTTGTATCCCCCATAGAACAGTAGTCCCCAGCCACTTAAAGAGATAATCACAAAGTGTTCTTCCTTCCATTTAGACGGACTCATTGGGTCTGACCAGAAGTTAACTTTGGGAGGTCCATGGTGATCAGCTGCTCCGGCGAGACCACGGCGGTGGATGAGTTGAATGGGCGTCTTGGAAGAGGAAAGACCAACGCGGGTTAGGGATGCGGCTCCACGAGCAAAAGAGGCCATGGTTTTTGTACTACTCCAACTGAACGTCTCTTAGTTTATCCCTTCATGCAAAACTTAAGTGTTGCCTATCGTCTACGAGAACTCAGACCTGGGGACCCCGTTTTGGATTGGCCTACTAGAAAACGAGTAGCTCTAGGAACAGCCCGTGGCCTTGAATATCTTCACGAGCAGTGTAATCCTAGGATTATTCATAGGGATGTGAAGGCAGCTAATATATTACTAGATGGAGATTTCGAAGCAGTTGTTGGTGACTTTGGTTTGGCAAAATTAGTTGATATTCGAAATACTACTGTGACAACTCAAGTTCGTGGGACAATGGGTCATATAGCTCCAGAATACTTGTCCACAGGAAAATCTTCAGAAAGGATGATATTTTTGGTTATGGGATTATGCTTTTGGAACTTGTTACAGGTCAACGTGCAATTGACTTTTCA

>PvRGL097

ATTGCTGTGCTGACATTGGTCCTCGTGCTGGTTCTAAAACAACGTCACAATCGTAGAAGCTTGGAAGTTCAAAGGGGGACCAAAGAAGAGGGACCTGTTCAGGAAGGGGAAAGCCCTATACCTCCTAAGGATGCTGATTTTGTGACTGGCGTGGGAGAAGCATTTAGTTCTGAGAAAATTATCCATTTAACTGGCAATTTTGCTGAAGCGAATATCATAAAGCATGGCCATTCTGGGGTCCTCTTCTTGGGAGTCATGGAAGGTGGAGCAACTGTGGTTGTCAAAAGGATAGATCTGAGTTTATTTAAGAGAGAATCATACATCGTGGAATTGGGACTATTAAGTAAGGTTTCACATGCAAGATTTGTCCCAATCCTGGGCCACTGCTTGGACAATGAGAATGAGAAATGTATAGTTTACAAGTACATGCCAAATAGAGATTTGGCTACTTCTTTGCACAGAGTCAACGAGTCAGATGGTAAATTGCAGTCCCTGGATTGGATCACGAGATTGAAAATTGCAATAGGAGCTGCTGAAGGCCTAGCTTACCTACATGAGTGCAGCCCTCCCCTTGTCCACAGGGATGTCCAATCTAGCAGTATACTTCTTGATGATAAATTTGAAGTGCGACTTGGAAGTTTGAGCCAGGTTACCGCCCAAGGAGATCTTCAACAGGGAGTCATCTCAAGGGTTTTCAGCAAGCCACCACCTTCTAGTGAAGGCGATCCTGGTAAATCATCAGTGACATGCACCTACGACGTGTACTGTTTCGGGAAGATTTTACTGGAACTCATCACCGGTAACATTGAAGTGAGCAAGTCAGATGATGGCACTTCAAAAGAGTGGTTAGAGCAAACCTTGCCTTACATCACTCTGTATGATAAAGAACGAGTGACTAAGATTGTTGATCCACAATTGATTGTGGATGAGGACCTATTGGAAGAAGTATGGGCCATGGCAATTGTGGCTCATGCATGCTTGAATCCCAAGCCTTCCAAACGTCCTCCCATGAGACATGTCCTGAAAGCATTGGAAAATCCATTGAAGATTGTGAGAGAAGAAAATGTTAGCTCAGCAAGGTTGAGAACAAACTCTTCTAGGAAATCTTGGAGCACTGCATTCTTTGGTAGCTGGAGACACAGCTCATCAGACAGTGTTGTGGCCATAACACATACAAACAAAGAGAGTACCAGTGACACTAAAAAGTCAAGAGTAGGTTCTCAAAGCAGTGGAAATGATCACTCATCTTCCAATAAAAGGTCATCCAATGAAATTTTCCCTGAACCATTGGACATGCAAGATGTGGAGAGTGGAGGGGCAAGATAAGCACTTGTGAAAGATTTGGTTATTCTTTAGTCTGTGAGGTTTTTTTACCACTTTTTCAGATAATTGGTGGTGCCATAGCAGTTGCTTGTGAAAGTCACTACCCAGCATCCATTATGGTCCATTTTGAATCAATGGAAAACCTTGATTCTGCCAGTGATAGCTTTTGCTTTCTGAAGCTCAGATTAAGAGGTCTTGTTGAAATGTCTGGTATATGAGAGCTCCAAAAAACTGAATATTTTTTACTCATTCAAACAGCCCTCCTTTTAGGCAAAGAATTGTGTGAAAAAATGTAAGATAATGAGAGGGTTTTCATTCCTGCGAGGAATTGGAAGTGGAAAAAAGGTTGTGTTACTCGTGCATTCCATTCGCAGTTTGTATTTAGCATTGTAAATCTTATTGAGTTCTGTGGCATAAGCCCTACTCTTGAAGTGTTGAAAAGTGTGACATGATTGGTAGATAATTTTGTTCTTTACGTATGTGGTTTAGAGGTTTAAAA

>PvRGL098

AATTTCAATTGCATATATCTCTCATTCATCGAAAACAATTTTATACATTAAACTACAAAATAAAAATACAAAGCTGAAATGTTTCACACTCTTGTATTAAAACTGGACGATCCTGCGGTTCTTTTTGCAGTGCAGGTGATCCTAATCCAATGACTGAGAGTGATCTTAGAAATGACAATGGCCTTCACTAGCATCTACTCAGGGAACTTCAAACTTATGCTAAAACGCTTACACCAACTCAAACCGATGTTCCGAAATGCTAAACTAAGCAACAAAGAAAATAATATAAAGAAAAGTGCAATTTGAGTGCAACTTTACAGAATTAGATAGTAATTGTTCCATTAGATATGCACACTCAGCTGGTTTGGTCAGCTTTGGGGGCAGAAGAACTGTTTCCTGGACCAGATGCCTTCTTCGGGAAAATCGACCCAAGCTGGATTCCTGTATTACCACTTTCCTGAGAACTTCCTTGGCCAGGATTGCCATCAGCATTTTGTTCTTTCCTCCTCTTCGCCTTTTGTGCCCAACCAACAATGTTTTGTTGCAAATGATCATTAAATATTGCCCTCTTATAGTGAGTTCCCATCTGCGTAACTAATGCATAGAGTGGCAGAGTGCTGTAGCTACATAATACCTGAATAAATACCCCAATAACGAGCCTTGGAATAATGTATCGAACTTGCCCCATTATACACGAGTCAAATCCATATGTAACCCATATCCAGAAAAAAAATGCTATCTCAAAAGCATTTTGGAAAAGGATAAAGTGAATTAAGAAGAGGACAACACGGGGCCGATGAAACCAAAAGTGTTCATCTGATGGTTGCACAACTAAGTCACCTTCTATGGCTGCATGCTTTTCAGCCACGTCGTGAGCTAGTTGGGTTATTACATGCTCCAGCTTAGTGCCCACAGAAAGCAATAGCTGAAAGAATGCAAACCAGAATCATGAAATTAATGAAGGGGGAGATACAGTGCGGGACAATAAAAAAATAAGGATATTCCAAACATAAAACCTACTACTATACGTCTACTTGCTCGAACAAATAGATCATGCTAGTGCATACAGGTTTAACAAGAAAGCACCGGTAGAAAGCAAAACACTAGCACAAATGTGGCTCTCCTCCAATAATCAGTAAAAATAATAATTGGAAAATTGCAAAAAAAAGAGTCCCAAAGTAACCACTAACTAATATAATTCTTAAGGGCATTTGTTTTTCAGAAGACATAAAACAATACTAGTTGTTTCCAGGATCAAAATTTCTTCGAATAATACTCATACGAAAGGTACTCCTGGAAATCTGTCCTGCCTACAATTGGTTGGTACTCTTAAGGCTTAAAACTGATTGACAAGAATATTTTATCAGAAATTACAAGACAAAAGCATAACTAAAATAAACAAAATTTTCTTCTTCCCTCCCACAACCCATTTGGTAAGACATGTCACATGGATAACTTCTGTTCCGGACTAAGTAGCAAGGGCCCAATTCACCAAGTATATGTACACTAATAAGACCCAAAAAGGTTAGTGACAGCATCTCTTAACATTTCACTTGCAAGGGGTTCTAGAATATTCAGCCAATAACGGAGTCCACATTCAACGAGGATTACCCG

>PvRGL099

AGCTCCATTTCAGAAATAACCTAGCTAGGCTATGAAGAGACTGTATCCAACATGGTAACTCTTGTAGACGCCCTGACAAATACAAGCGTTGAAGAAATGGAGGGGGTGAACAAAGAAATGTCAGATCAATGACTTTACTCTCACCCTCAGAAGTAACAGAGAGGGCATGAAGATTTGTTAATCTCTCAATGGACAAACAGAAAGCCATCCCATCTTCCTCTCTAAGTTTCAAGATTCCTAACCTTCTTAGTTGAGTTAGCTTCGCTAACTGCCTTATTATCATTCCACGGTCTTGGTTTGCCTCCACAAAACAAAGCTTTTGTAGTGACTTCAAATTTCCTATTTCAGTAGGGGCCTTAACACCATGTTTGGAGTGAAACTGTGCATAACCTTTGAATTTAAACTGATAGACAAGGAGATGGCGAAGCTTTTGAAGCTTGAGAATATCTACAGGCAATTTCTGACAGAAGTCTTCTTAAGATCGAGTGTTTCTAGATTGTGCAACTTCCCTATTAGGTAACCTGGAACCATTTTCACCTTTGTGTTCCTTAAGCTTAGATATCTTAAATGATATAGATCAATAACTGCTAGTGGAAACTTCTTCAAAGGTGCATCTTGATAATCTAAAACACTAAGCAGTTTAAAACCACTTGGGAACAGTTTGCCAAGGGGTACATGTTCTCCAACGCCAAACATTAAAAAGGAACGGAGTTGAGAGACAGACCTATGTTGCTGCCGATATGGTAATGTGCCGTGCACTGAAAGCCGCCGAATCTTTTCAGGCCATGCCGCACTTTGTTCTTTGACAATGGATGCAAAGTTTTGGTCCTTAGACTTCAAAATGATGATTTCCCGTAGAAGATCATGGATTAGTACAAAGACATGGCTGTTGACCTTTCCATCAGATGTCTGTCACCATGCATACTTGTATCAAGTTTAAATACCTCAGGAGCTCCTTCAGGTAATCTTCTCCCCAACCCCTATTTTTGTTCCTTTGTTTTTCCTTCTTTTGCTTCAACTAAATCCTGTTTGCCTATCACATGATAATGACGAATCAAACTACCAGAATGACGCTGGGTCAGAACAGTCCACAGTTGAAAGATGCTTAAGTACAACCAACACAGTCTATCTTCAAATGGTAAGCTCAACCATACATGATGAACAGCAAGATGGTTGGGGTTCAGGTGTCTAAGACTCAGTCAAGTTTGTCATTGCCATCAATTACTGCTACCAAGACTATGGCATTTGCTTGTCTTTTTCTCTCTCACATTCTCGCCCCAGACTTTTGCTTCGGCCACTGCACGCTCTCGGTCCAGGTCGCGGTTCCATGGAGTCTCTCTGGCTCTTGCAGAATTTTACAGGGCTGTCTCTCTGAGAGTCCTGCCTTTCATAATCATCCAATCCCCATTTCCTCCCTGAGCCACCACCCGTATCCATCCTGCCTAACTCAGTCCCGCTGCTTGGATCCCTGCTGTCTCTCTTCCTGAAGTCAGGGGAGTTTCTGTGTGTGGAAGGGGAGCCAAGGCGCCGATCAGGGCTGTCCACACCGTCTGCCAGGCTCCTTCTGTCATCCCTCATCCTAGGGGTTGAGGGGCCAACCCTGTTGGATTGATTGGCTGCATTTGGATCATATGTCTGCGAAGCCAGATATGTGAGAGCAGTCACCACATCCCCAATGAGAGGCCTTGTAGCAGCTTGCTCCTGCAGACACATTGCAGCAACTGCAAGTGCTTGATACAGTCCTCGCATCGGGTAACGGCCTTGAAGTAGAGGGTCGGCCATTTTGGGAAACTTCCTACGGTCCTTGAAGAGAGGCCGCGCCCATGCAACAAGATTGTGCTCTCCATGTGCACGCGTATTGTCAATAGCCTTGCGCCCAGTAATAAGTTCAAGGAAGACAACTCCAAAACTGTAGACATCAGATTTCAGAGTTAATTGACCAGTCATAGCATATTCTGGGGCACAATAGCCGTATGTTCCCATTACTCGTGTGGATACATGGGTCTTGTCGCCAACTGGACCCAGTTTTGCCAGAGCCGCAACGGAATTCTGATATCAAGCTTGGGATGGATAACCCTCGACCTCGAGGGGGGTGCCCGGATGATTTTAAGTCGCCCTGTATATAACTGGTGGATTTGCCTTGTCATGCAAGTATTCCAATCCCTTTGCAGCTCCAGCTGCTATTTTCATCCTAGTGTTCCAATCCAGAGGCTCCTTGTCAGGAGGAAGATCGTGTAAATGATCCTCCAAAGACCCCAATGGCATAAATTCATAAACAAGCAGCCGCTGGTCACCATCTGCACAATAACCAATTAAGTTCACAAGATTCGGATGATGCAAGAGACTGAGCATGAGAACTTCCACCAAAAATTCTCGATTTCCTTGAAGACCATTTCGATCTAGCTGTTTTACAGCAACTGCTTGTCCTGTGCTCTCGAGGCGACCTCTGTAAACGCGTCCAAAACCTCCTTCACCTAATAAACATTCCGGCCTAAAGTTCTTTGTAG

>PvRGL100

AAAGAACCAAACCGTTATTCCAATCTTTTACAACGTCGATCCCTCCGATGTAAGGAACCTGAAAGGTAGTTTTGGCGCTGCCATGGTTGCCCATGAAGATGGGTTCGGAAAGGACATGAGAGATTACAAAAGTGGAGATCAGCATTGACTCAAGTGGCCAACTTGTCAGGATGCTGTTTGGGTACAGGAAGCAGGTTCGGATATGAATATGAGTACATTGAAAGGATTGTGAGAAGCGTGACCTTGGTGATACCCCGCTATAATATTTTTGTGAGTTTCAGTGGAAAAGATACACGCTCCTTCTCGGGTTTTCTCTACAATGCTCTGAGCAGAAGGGGATACCATACCATCCTCAATGATGGGGACCAGAGTTCACAATCTACTACTGTTGGGGTTATTGAAAAATCAAAACTTTCAATCATTGTGTTTTCTGAAAACTATGCACGTTCTCCCTCATGTCTTGATGAGCTTTTGAGGATCCTTGAGTGCAAGGAGATGAAAAACCAACTGGTTTGCCCCATCTTTTACAAAGTGTTACCGTCTGATTTAAGGCATCAAAGAAATAGTTATGGTGAAGCCATGAGTGAACATGAAAATATGATGGGTAAGGACTCTGAGAAGGTGAAGATATGGAGGTCAGCTTTGTTTGAAGTCGCCAACTTGAAAGGATGGTACATGAAAACAGGGTACGAATACGAATTTATTGAAAAAATCGTGGAATTGGCCAGTAAAATTTCTCGGGTGTCAAGTTGAAGGAAGATTTGGGGAAATTTTCCATTGACTAAATTGCCGTGGAAGCCAGAACTGAACAGATTGTTCTTTGAGCAGTCATTAGCTTACAGATTCATAGTGGTGGTGTTACAATCAACACTGAAGAAAATTGTCCTTCATGAATATCATACTCACATTCCATATCTCTCTCTCAATATGGCAAATAGAGGTGTCTATGGACAACCTACTCCCATTAGTTCAATTGAATGTACTATAATTCATAGACTAAAACCCTATTTTCTCTTACTTTTTTAATGTATTAAAGATTCTCATCACTTTTGTTCTAGGAAGTTTGCAAAGGCACTCCGACGATCAAGTTAATTTGAGTACAAAGTGCAGGTGAGTTGAGAGTAAAAAATTATCTTTGACGATGGGTCTCATTTGGTATTTATACCTTTTGGATGAGCTCCCGTCTGTCATGGGTCTGCTCATGAGTCCAAGAGAAGACCTAATTACGCCT

>PvRGL101

CCACCGCCAACTTTACACAGACAAATACTTCAGTGGCAAGACAAGGAAGTTCCTCGTCGTACATTTTTTCTTTTGTCGAAGTTTGTTTATTCAGGTATTGCTTTCTGAGCCAGTTGCATTTGATTGTCACTTCCTAAGTAATGTTCACACTTCACAATAATGACTCAGGCAGTTTACTGTGGAATAAGTCACCAAGAAAGATTCAAATGACATCACTTCATTTCACAATGGTTGAAACACCTTTAGGTAGGATAGATAAAGAAATAAAATTCTTAAAAGGAAAAAGAGATAAAGACCAACTTCACTATATCATCATCAAATACGCTACTTATGCTCTCCCTGCACTCTTGAGGTAATCTGCCCTTATTGCCCACAACTGCTCGCTCACTGATTTCATGTCAGGTCTCTCTGTTCTAATGGGTGCTGCACATTGAAATGCCAAATCAAACATCTTCATGAGAACATCTGTGTTTACTGCTTCTTCCATTAAAGGATCCACCAGTTCCACCACAATTCCTTCGTTGTACTTCCTGATGGCCCATCTAAGTGTAACACGCTCTTCAACAGTTTTCTTCATCTCAACGGGACGGCGGCCAGACACAATTTCTATAAGCAAGATTCCAAATGAATAAACGTCACTTTTGGGAGTGAGTTGGTATGTTTTCATATACTCGGGGTCCAAATAACCAACTGTTCCCTTCACTTTGGTAGAAATGTGTGTCTGATCAGCGTTTACAGGCCCTAGCTTTGCAAATCCAAAATCAGCAACTTTAGCTCGCATGCGTTCTGTCAGAAGAATGTTGGATGATTTCACGTCTCGATGGATAATTTGTTTTTCTGCATACTGATGCAGATAGGTCAAGCCATGAGCAACATCAATTGCAATTTCAAGTCGCTGATTGAAGTCCAGTGTTTTTCCACGCATACCATCCAAATGTTCTCTAAGGTTACCATTTGGCACAAACTCTGTAATAAGAAGGCGTTCATTTCCTTTATCAATATAACCTAGTAGCTTCACCAGGTTTCGATGATCAGTTTTGGCCAGAAGTTGAATTTCATTGCTGAATTCTGTTCTCAAACTATCAAAATGTTCCTTTTTTGCACGTTTTACAGCCACAACCAGGCCATCTTCCAACTTGGCCTTGTATACAGTTCCAAAACCCCCTTCTCCTATTTGCAATGTTTCTGAGAAGTTATCAGTAGCTCTTGCAACTTGGTTCAGATTGAGATGCAGTGATTGAAGCCTACTGAGTTTTGGAGACATTGAGAATCTGGACGGACTAGCTGGCACCCGTAGTGGACTGGCCGGAATCTTGTCAGTAACGGAGGCTTCGAAGGAGGAAACCGAATCCATTGAATTGGGTTCCTTATTCAGAACAGCATGAGAAGTTGTTTTCCTTCTCTTAAAGAAACAAGGACAAAGAACAAGACAACATACAAACAAAGCTCCCCCTGCAGAAATACCAACCACGTGGGCTGACAGAGGCTTAGAATCTCCTTGCGATTTGGATTCACTATTTGAATCCGTTTTCAGAAGTTTCCTCCCTGCCTTTAAAGGAAAATCCACAAAAGAAACATCCGTCACACATTTGACACTTTCAAAGTAGTTCTTCACATCACACCCCTTTTCAGTATACAATTGGAGAGCCTCACAGAAAGCAACCTTGTTCACCACATTGCCGTTTATGTAGAAAAGCTCCTGACCATGACTAGAATATGAATGTGCCACCCAATCAGTGCCACAATCCTTTGACTGTAAGACAACCTCAGAACAAAAAATTGTTGACAATTGCAGCAACAGAACCAAGAATAACGCAGAAATAGCCATTATGAACTAAACACCGCAATCCAATAGGAAAGCATCTCCTTGTACTAACACAGAAGCAACCAGGTGCATAAAACTATCAAACGAAACCAGCACAGTCCTGCTCAAGCATCATATTTAGCCTAAAACACAAATCATCTCCACAGTTCCAAAATATTGACCAGCAGAACCCAGCAAACAATCAAAGGTGTTGGCCCAATTCACCAAATTGGGCCACCATGAATTGAATTGGATTTCTGAATTAAAAAAAAGGCAAGATCAATTCCTGACAGTGAGAACAAAAGATGACCGTGAAAAAGGAGTTGCTAGGATGAAGTTACAGTTGTGCAGTGCTTCCTGAATTTCTCCAAAACCTCGATCACCCAATCCGACAAAAAAACTCAAGGTGGCAGCTGGCAAGTGATCCAAGACAAGACCTTGGAACTCACCAATTGTTGTTAGAATTCAGACTACCCAATTCACCCAAAAANAAGAAAAGACAAAAGAGCAAAACTTTCCACCGAGAACCC

>PvRGL102

GAAGAAAAAACTCAATCCTGTTTTCCAATGCAAGGACCATTACAATGTCACTCTTTTCTACTTTTCCTTTGCATCCTTTTCTGTCTTTTATTTGCTTCAAGGCTCGTTGCACTTAGCCTGCTACTAGTACTAGTACTAGTATGTATTACCTCTAATAAGTATTTACAGTATGATCTATGTGTGTCTACTAACCCATGCTTCCTTGTGTTTCTGATGCAATGGAGGTGTCAGGTGAGTTTGACCCACTTCTTCTACCATTTCGGTGTTCATTGCTCTGTGACTCCGATGATGAAATTGGTGTCTTGGGCGATTTAAGTGGATGAGGCCTATGCCCATTGTGGCGACGATTTCGGTGGTTGTGGTGGTGACCATTTGCATCTTTATTGGCCTCATCGTGATCATGATGTTTATGACTATTGTGATGATGATTCCTGTGATGATGATGACCATTTTCCCTCTTTCTCTCCTTGGGTGTTTCAACCTCTTTTGCCATTTCATTGTGATCAGTGGGAACAGTGTAAACAAAGGGTCCAATTGGAACATCATCAAAGTCCTTAAGAGGCTCAAGAACACTAACCACAGTGCTAATCAAGGGTCTATTTCTTGGCCTGTGGCTGAGACATTGGTAAGCCAATGCAGCTGCTTTTCTTGCCCCTTCCTCAGAATACTGTCCCTCGAGTCTTGGATCCATTATTCGGCTAAGTTTCCGGGGATCATTCAAAACAGGCCTGGCCCATTCCACCAGATTTTGTTCTCTTGGCGGGCGCCCTTTGTCAACTGATCTTCTTCCTGTTAGAATCTCCAAGAGAACAACTCCAAAACTGTATACATCACTCATTGCTGTCAAGTGTCCTGTCATGATGTATTCTGGGGCCGCGTACCCTTGTGTGCCCATCACTCTAGTTGAAACGTGTGTGTCATCTCCTTCGGGACCATCTTTTGCCAAGCCAAAATCAGAAAGCTTTGCATCATAATCAGAGTCTAACAATATGTTTGAAGCTTTGAAATCTCTATAGATGACTGGTTTTTTTGCTTCATGTAGAAAGGCTAGACCCTTTGCTGCTCCAGCAGCAATTTTCATTCTTGTTGACCATGTTAACGACGCCGTAAATCTTCTAAATAGTTGATTCTCTAAGCTGCCTCGTGGTAGATATTCATACACCAGAAGCCTGTGTTCTTCTTCACAGCAATATCCAATCAACTTCACCAGATGTTGATGCCTTAGTTGACCCAGAAACACAACTTCAGTCAACCACTCTCGGTGCCCCTGAGAACCGTCCAAGTCCAAGAGCTTGACGGCCACCGGTTGAGCCTCGAGGCCAGGCCTAAGCTTGTCATCAATGAAGCCCTTATGCACAGGTCCAAAACCTCCTTCTCCCAGAAAGTTACTCGAAGAGAAACTCTGCGTGATGATCTTGAGCTCGGCGAGTGAAAAAACATGGAGGTTTGACCCCACTAAAGAAACGGAGAGATCCTCCGACAGCGTTGAGCCGGGAAAACTCAAGTCCATTATGGAGACCCTGTTGGAAGAAGAACCCTTTGTAGCCACCTCTTTGGTGGGCTTGGGAGAAAGAGAGACAGAATCACTCTTGAAACAAATGGAGAAGATGGATCCCCATGCGATTGTCATGTCAGTTACCCTTGTGTTTTTATCTGGGGTGTGTGTGAATTACCTTCAATCTGATGAGTCTTATGCTGCAAAAGTTATGTTTCTCTGGGAAGAGAGATATGTGTTTGAAGAAGATAGCATAGGGATGGTGTGGCAGAAATGCAAAAAGGTTGAAGATT

>PvRGL103

CTTGATAAATAATCTATTATTGAGAAGTTGAATTTGAATGATAAGAAGATTTGACTCTGATTTTCTCTTATAACTCTTTTTCATCGTTACATAATAAATTAATTAACATCAGCCAAAAGTTGATGAAAAAATTGTTGAGGATCTACACTCGTACTAGCCCAAGGCAGTGAGCACGCCCGATGAGACCAGCTTAGTCAGAAAAGAATTCTGTGGGGAAGCATTCAACAAAATAAAAACTAAAAGATAAAACAAATCAAGCTATGCAAATGCATCCAAAGAAGAAAGAAAAACTATGAACACTGGATCAATCCTTGTGATGCTCTTCAGAGTGACCAATTAGTTCTGATCCTACCCTTACGCATGCTCAATAATCTAACTAACGAGCAAGATTGAATGAACTCCAAATGGAGCTTGTGAAGGTTGCTCCAGCTGAACTTCTTTCGTGGGAAGAGTTTTGTTTTTTGATTCTGATCTTCCCGAGATCAGCAACGAACCCTGGTTTCGAAATGTGAACATCTTTCATATCAACGTTGTTGGTTAATTTCTCCACAACCTCTGACATTCGTGGCCGAAGTTTCACGGTTTCTTGCACACATAGTAGCCCCACCTTGAGGAACTTTATGGCTTCTTCATCCGGAAAATTTTTGTTCAACATGGGATCCACCAGTTTTAATAAATCGTTAGATTGATACGCTATCCACACCTTCTCCACTATGAAACGTTCAAAGTCTTGATAGGCATCCACCACTGCTAGGCCACTCACAATTTGCAGAAGCAGCACTCCAAAGCTGTAAACATCTGATTTTCGTGAAACCTGTCCAGAATTGGCATATTCTGGAGCTAGATAACCCAGTGTCCCTGCAACCCGAGTGCTGATGTAAGAAGCTTCGTCTCTTAGCAATTTTGCCAAACCGAAATCTGAAACCTTTGGAGTGAAATTCCGATCAAGAAGAATATTTTTGGCTTTGATGTCTCTGTGTACGATATGAGGCTTAAGATCTTCGTGGAGAAAGTCAAGTCCCC

>PvRGL104

TCAAATGAAAATGGAATAGGTATTAAGGAATTGTCGTAGTGAGTTCATTTTGACAACATGGAACGTCCTTTCTGATAGACATGGAAATGGAAAGACGCCATGAGATACAATTCAAGGCATTTACAACACAGTTTAAATTACATTTCCTCCTTGTATAATGAATCAATATTTCAAAATTTGTCAGTCTCTGTATCTCTCCAACCATAACATGAAAACTGCAGAAATTTGAGATGCCTAAGCTAAATCAGTCACTCAATCATTAGTTCTGATCCCTTTAGAAACTGTACATTTAAAAAACACTGGAAAATCCCCCAAACCTCTTAAACCCAGTAACCATCATTCCAATTACGCTTGGAAGAAGGTCCTCCATGCTCACTCCGACTTCCTGTAAATCTATGTAAGGACAGCTTGCTTCGGACTGTTCTTAAGGGGCCTGATCTATTCCCATGCCTAAATGCTAAAGATATTTCATCTCCAATCAAGCTTGATGTTGAAAACGGTCTGTTTTCCATTTCTTCAAGTGGACCAGAACTGTATTCCATAGTGATGAAGGAAGATGCTTGCCCATAAGTTAATCGAGGGGTTAACCCTCCCGCATTCCACCAACTGCTATCAGAAGGGGCAAGGTCACCTCTAAAATCGGACTCCTGAGCAGAGGAATCCTTCTGACTACAACAATCTCCACATGTCTCATCTGTGTCGTTGTATATTAACTTAAGAGCTTGCACAACTTCACCCATAAAAGGTCTCTGTGTAACCTCCGTGTGAACACACATTGAAGCGATTGCAGCAACCTTTGCCATGTCATCAAAGTTGTAACTTCCAGCCAAAGATGGATCCACCAGCTGTTCTAGACCTTCTCTACTGGTCAACATTGGCCGTGCCCAAGTTACAAGATTCTCCTGTCCCTGAGGTTGAGACATATCCACTGGCTTTCTGCCTGTGAGAAGTTCAAGCAGCACAACACCATAACTATAAACATCACTTTTGACGAGTAAATGCCCTGTCATTGCATATTCTGGGGCAACATACCCAAAAGTTCCCATCACTCGTGTTGAAATATGATTACTTCCTTCAGTTGCTTCTCTTGCCAATCCAAAATCAGAAACTTTGGGAGTAAAGTCATCTTCTAGTAACACGTTACTAGCTTTGAAGTCTCGATGAATTACACGGGGATTGGAATCCTCGTGAAGATAGGCCAATCCTCTTGCAGCTCCTAGGGCAATTTTCATCCGTGCTTCCCAATCTAGCATCCCCTTCATCTTGTCATCACCATGCAGATGGGACTCGACACTGCCATTGCGAACAAGCTCATACACCAAGCAACGCCTGCGACCCTCAATGCATATACCAATCAGTTTCACTAGATTGCGGTGATGCAAACGGCTTAGCATCTCAACCTCTGCAATAAATTCCCGGTCTCCATTCTGATTATCTCTTGTTAGCAGCTTAACTGCAACTTCAGCCCCATCTTCCAATGTTCCACTATAAACACGTCCAAATCCTCCTTCACCCAGTACTCTCTTTGAATTAAACTTATCTGTTGCTTTCTCAAGCTCAGAAAGTGAAAATGTTTTAACAGAGAGAATGGAAGTAGCCATTGTGGACATAAGTGACACTGATGTTGAGCTTGTAATACTGCTTGACAACATAGACCCCAAGCCTGATCTCTTGTTTGGAGATGATGTGAATGCAGGGCCAACAGCACTCGATGGTCTCCTA

>PvRGL105

TTGTTGGCTTAGGCTGAGGTTTATTGGCAGCTGCATTTCCTCAAGATCCAAGGTCGATACCTCTGTCAGTGGCAGTGGCACCAGTACTCATTATGCTGAAAGTAAATCAACTAATGATACTAGTAGAGACCAACCAACGGCTCCAGCAGTCTCTTCTACAACCACTAGTATTGCAGAAAGCAATTCATCCACTTCCAAACTTGAAGAGGAGCTTAAAATTGCTTCCAGGCTGCGAAAGTTCCCTTTCAATGATCTTAAGTTAGCTACCAGAAATTTTCGGCCTGAAAGTTTTCTTGGTGAAGGTGGGTTTGGTTGTGTTTTCAAGGGTTGGATTGAAGAAAATGGAACTGCTCCAGTGAAACCTGGCACAGGGCTTACGGTTGCTGTAAAAACCCTCAACCATGATGGGCTCCAGGGTCATAAAGAGTGGCTGGCTGAAGTAAATTTTCTTGGCGACCTAGTTCATCCAAACCTTGTTAAACTTGTGGGTTACTGCATTGAAGGTGATCAAAGGTTGCTAGTGTACGAGTTCATGCCTCGGGGTAGCCTAGAAAATCACTTGTTTAGGAGATCCCTGCCTCTTCCATGGTCCATTAGGATGAAAATTGCCCTAGGAGCTGCAAAGGGTCTTGCTTTTCTTCACGAAGAAGCCGATCGACCAGTAATATATAGGGATTTTAAAACTTCAAATATACTATTAGATGCAGAGTATAATGCCAAGCTCTCAGACTTTGGTCTAGCCAAAGATGGCCCAGAAGGTGATAAGACACATGTGTCTACTCGAGTGATGGGAACCTATGGCTATGCAGCACCAGAATATGTCATGACAGGTCATCTTACATCAAAAAGTGATGTGTACAGTTTTGGAGTGGTACTACTTGAGATGTTGTCTGGCAGAAGATCCATGGACAAACACAGACCCAATGGTGAGCATAACCTTGTGGAATGGGCTCGGCCACATCTAGGAGAGAGGAGAAGGTTCTACAAGTTGATAGACCCTCGTTTGGAAGGCCATTTTTCGGTGAAAGGTGCTCAGAAAGCTGCCCAACTGGCTGCTCACTGCCTTTGTAGGGATCCAAAAGCCAGGCCTCTGATGAGTGAAGTTGTAGAAGCTTTAAAGCCTTTGCCAAACCTCAAGGACATGGCAAGCTCTTCATATTATTACCAGACAATGCAAGCTGACCGCATTGGAGCGAGCCCGAACACACGAAACGGGCGAACACAAGGAGCATTGCTCACCCGCAATGGACACCAGCAAAGGAGTCTTTCTATACCAAATGGTACCTATGCATCTCCTTATCATCATCAGTTTCCTCAACCATCACCAAAACCCAATGGCAAAGCGTAGTAGAGCTGGTGAAAATTCTCTTTTTTCCCTTTCACCTTTTATGTCATTTCTTAGTTTTTCCTTGTAGATGTACCTTCTGTCAAAGCTTCTGTTCATGGAGATGTTGAAAGCAAAAGAAGCATGTTGCTTTATCATATTATGTTATGGCTTTTGCTTGAAAGAATATCTGGGTGGGAGCAAATGAAGCTTATTGAGCTACTATCTCTTCTGTCAAATTGTTATTCAATATTTTCATCAGGTTGTTTGAAAATATTGAACATTATGAAATCCTCATCCTTATTTTATTTTGTTTTTCACATTTGTGATCGGAAAATGTTATCAAATCCTCATCCTTATATAGGATTTGCTTGTGATATC

>PvRGL106

CAAAAACACTAAATATAATCTCATTTAAAATAATTTTTCTTTAGTTATTATCTTGCCAATCCATCCTATAACTGAAAGATATTCTGACTTTAATTGTAGCCATGGTTCTGCATGTGGAAACTGTGTATCAATTTCTTAGGGCTCATTAGAATTAAATTGACATCTAAGATTCAACACTATTGACGACACACATTTAACAATCTCTTTAACAAGCATAGCATCATCCCGAAAAGTAGATGAATCAAATCCTGCTACATTTGCAGATTCCTTCAAAGCAAATCTCCATGTTTGCACTGTGCTCAGATTGTATTCTCTTTCATGAGCAACAAAGGCTTCTACATAACTTCCCCTCTGATGTCGTACATCGGAAGGATCTACTTTGTAGAAAATAGGTAGCAAAATGTGTCCATATTTTTTACTACACTCAACAATTTTCACAAGTTCTAATAAACACCAGCTTGAAGAAGCATAGTTTTCTGAAAATATAATCAAATAAATAGATGATACTTGAATTGCTCTTAGAAGTACATCAGATATTTGATGTCCTTTGGGAATGTTGTAATCTACAAAGGAAGCAATCTGGTTTAGAGAAAGTGCCTCAATCAAATGGCTAAGGAAACCATCACGAACATCTGAACCTCTGAAGCTAACAAAGACATCGTACTTTGATTCAAGGGTGTCATGAAAAGTTGATGAATGAAATCTTGATAAATTAGCAGATTCAGGCAAAGCAGATCGCCAGCTTTGCACAGTAGTGAAGGAATAGTTTCTTTCATGTTTAACAAAGGCATCTCCATAACTCCTCCTTTGATGCCGTACGTCTGAAGGATCTAATTCGTAGAAAATAGGCTTTACAATTTGTCCATTCTTTCTCCTGCACTCATAAATTTTCTCAAGTTCTAACAAACACCATCTTGAAGAAGCATAGTTTTCTGAAAATATAACCAAAGAAATTAATGATTCTTCTATGGCTCCAAATAGTGCTTCAGATAGTTCTTCCCCTGTCTGAATGTTATCATCTGCGAATACAGCCATTTGCTTCTGAGAAAATTCCTCAATCAAATGGCTAAGGAAACCTCGATGAACATCTACACCTCTTAATCTAAGGAAACCTCGACGAACATTTACACCTCTCAAACTAACAATTTCGTCGCACATTATTATTTCAGCGTTGTAACAACAACAAAATGTTGCGTTGTAACAACGAAAAAATGTTACCAACAGATAAATGACTACAACATATTTGATTCTATGAGAAACTCGGAAACACATGGGATTGACTACAATATGCATCTTACTTTCTTCTTCTTTCTACACTAATATAATTTTGTTTATGTCTAATATGATTAGCTCTGTTCTTCATAGACAAACTTGGGATTTCCGGATCTCAAGTAAGATCGGTTCAGGATCATGGGATCCTTTTCTGATAGGAAGAGGTGTTTC

>PvRGL107

GGGAAAGAAAGAGTTCAGGGCTGAAGTTAGCATCATTGGCAGCATTCATCACCTCCATTTGGTTAGGCTCAAGGGATTCTGTGCTGATGGAACTCACAGGCTTCTTGCCTATGAGTACATGCCGAATGGCTCCTTGGATAAATGGATATTCAAGAAAAAAAAAGGTGAGTTTCTGTTGGATTGGGATACCAGGTTCAATATAGCTCTGGGAACAGCAAAAGGGCTTGCTTATCTGCACGAAGATTGTGACTCCAAGATTGTTCATTGTGACATCAAACCAGAAAATGTTCTGCTGGATGAACACTTCATGGCCAAGGTTTCGGATTTTGGGCTGGCTAAGCTCATGAACCGAGAACAAAGCCATGTTTTCACAACACTGAGGGGCACCAGGGGCTATCTTGCGCCGGAGTGGATAACAAACTACGCTATATCAGAGAAAAGCGATGTTTACAGCTATGGGATGGTGCTGTTGGAGATCATCGGGGGAAGGAAAAACTACGATGCAAATGAGTCTTCAGAGAAATCCCATTTCCCAACTTTTGCTTTTAAAATGATGGA

>PvRGL108

TGTAGAGGGAGTTCATAGGCTGCTTGTGTATGAATATGTGAACAATGGTAACTTAGAACAGTGGTTACATGGAGACATGCACCAGCATGGGACACTTACTTGGGAGGCCCGCTTGAAAGTTATACTTGGCACTGCCAAAGCGCTTGCTTATTTACATGAAGCAATAGAACCAAAAGTTATTCATCGGGATATAAAGTCTAGCAACATATTGATTGATGATGAATTCAATGCAAAGGTTTCTGATTTTGGCTTGGCCAAACTTTTGGATTCAGGAGAAAGTCACATAACGACAAGAGTAATGGGAACGTTTGGTTATGTGGCACCAGAATATGCTAACAGTGGTTTGTTAAATGAGAAGAGTGACATTTACAGCTTTGGTGTCCTCTTGCTGGAAGCAGTTACTGGAAGGGACCCTGTAGACTATGCACGTCCTGCCAATGAGGTTAATCTAGTTGAATGGCTCAAAATGATGGTAGGGACAAGGAGGGCTGAAGAAGTAGTGGACTCAAACCTTCAAGTTAAACCACCGCTGCGTGCTTTGAAGCGCACTCTTTTGGTTGCAC

>PvRGL109

ATGAATCCATTGAGCTTTTTAGTTGGCATGCATTCAAGCAAGCAAGTCCCAAAGAAGATTTTATTGAACTTTCAAGAAATGTAGTTGCTTATTCTGGGGGATTGCCACTAGCTCTTGAAGTCCTTGGGTCTTATTTGTTTGATATGGAGGTAACAGAGTGGAAGATTGTATTGGAAAAGCTCAGGAAAATTCCTAATGATGAAGTACAGGAGAAGTTAAAAATAAGCTATGATGGTTTAAGTGATGATACAGAGAAAGGAATATTCCTTGATATTGCATGTTTCTTTATAGGGAAGGATCGAAATGATGTTATACATATATTGAATGGTTGTGGACTTTTCCCAGAAAATGGAATTCGTGTCTTGGTAGAGCGAAGCCTTGTAACTGTAGATGATAAGAACCAACTTGGAATGCATGATTTGCTACGGGACATGGGAAGGGAAATTATTCGTTCAAAATCACCAATGTGGCTTGAGGAGCGTAGCAGGTTATGGTTTCACGAAGATGTGCTTGATGTGTTATCAAAAGAAACTGGAACAAAGTTTATTGAGGGATTGACTTGGAAGTTACCAATAAGTAATACAAAATCTCTAAGCACTAAAGCTTTTATGAACATGA

>PvRGL110

ATCATATGGGGAAGTTTACCGTGGTGAATGGCACGGAACTGAAGTTGCAGTAAAAAAGTTCTTATATCAGGATATCTCTGGTGAATTACTTGAAGAGTTCAAAAGTGAGGTCCAAATAATGAAAAGACTGAGGCATCCAAATGTTGTTCTCTTCATGGGAGCAGTAACTCGGCCTCCAAATCTTTCAATTGTTTCTGAATTTCTTCCTAGAGGGAGTTTGTATAGATTAATTCACCGACCTAACAATCAATTAGATGAGCGGAGGCGGTTGCGGATGGCCCTTGATGCTGCCCGAGGAATGAACTATCTGCACAGCAGTACTCCAGTGATAGTGCATCGTGATTTGAAGTCCCCAAATCTTCTTGTTGACAAAAACTGGGTTGTAAAGGTATGTGATTTTGGCTTATCACGAATGAAGCATAGCACCTTCCTTTCTTCCAGATCAACTGCAGGAACAGCTGAATGGATGGCTCCAGAAGTGTTGAGAAATGAACTTTCAGATGAAAAGTGCGACGTTTTCAGCTATGGCGTCATATTGTGGGAGCTCTCTACTTTGCAGCAACCCTGGGGAGGAATGAATCCAATGCAAGTTGTCGGTGCAGTGGGTTTCCAACATCGCCGTCTTGACATT

>PvRGL111

AAAAACAAGCATTCTCATTGATGATTGATTGCGGCTCAATATCTACAACCATTAACACTTAAATGTCATAGTGGAAAATACAAAGCATACATACACATGACATCTCAAAGGGCACATGAAATCTCATATACTGAAACACTATCATAGCACTGGTACTAGAAGAATTAGCCAGGACATTTTAAGACAGACAGAAAGAAAGGAGCATGGTATCTACAACTATTTTGAAAAACAAAAGGAAAAAGGGTACAACAAACAATTTGTTTCAACGCCCATCAGCTGAAGTGAAAGAATCTGCAAATCCAGTAGGCCTTGCGGGGATACTGCTCTTACTGTCTTCTAGGTCCATATAACTCATGTCCTTTCCTTCTGCCTCTTGCCATCCCTTCACCATCTGGTTAAGGGGAAGGCTATAATCGATCCCGGAATACTCCTCAGCCTCATCATTAAAGGGTTTCCATTTTTCAACAAGCGGTGCCAGCACATTTACGGCATGACCCATCTCTGGCCGTTGGCCTGGTTCTCTGGCTGTGCAGTGCCCTGCTAACTCAGCAATGATTGAGACGCTCTCAAATGTTTCTTCCTTAACATCAAGGACTTTGTCAATAGCAGCCATTAGCTTCTTCTTATCTGATTTTATACTCCAGAACCATGCTGCTAAGTATTGGCTTTCCTCAGGTCTGTCCTCATCAAGTGCCATTAATCCAGTCAATAGCTCCATTAGCACAACCCCGAAACTGAAAACATCTGCTTTGGTAGTGATTTTTCCTGTCACTGCATATTCTGGCGCCAAATATCCGAAAGTCCCAGCAAGCCTGGTAACTACAGATTTTTCACCGTCAGGAGCAAGTTTTACCAAACCAAAATCTGAGACCTTTGCTCTAAAATCATCAGCAAGCAAGATATTTGACGGCTTAAGATCTCTGTGAATGAAGCTCTGGTGAGCCAGAGTATGAAGATACTCCATCCCTCTGGCAACATCCAATGCAATATTGAGCCTCCTCTTCCAAGAGAGTGGCTCCAGTCCATGGCTTTTCCAATGGAAAAGATGCTTACTTAGAGCCCCTTGTGGCATATATTCATACACCAGAATCCTTTCATTACCTTCTATAGAATAACCCAAAAGAGACACCAGATGCCGGTGCCGGACTTTTGATAGAACCGCAATCTC

>PvRGL112

ATCACTGCATAAGCTAAGAAGAGATGTAGAGGAAGAAAATGTGGTTGGCTTTGTCGATGAATCCAAGGATATCATCAAACGACTCTTGGAAGGTGGTTCAAATCGTAAAGTTGTCCCTATCATTGGCATGGGGGGATTGGGCAAGACCACCCTTGCCCGGAAGGTCTATAACAACAGCGAGGTGAAGCAACACTTCAAGTGTCGAGCATGGGTTTATGTGTCAAACGAGTGCAGAGTTAGGGAGTTTTTGCTTGGCCTTCTTAAGCATTTGATGCCAAATTTTGAACAACAATGCAGAGGCAAGAAGAAAGGAAAGAAAAGCACTCAAGACAATAGTAGCTTGAGTGAGGAGGAGCTGAAGAAACTGGTGCGAAACAGCTTGGAGAGTAGAACATATCTTGTGGTGGTAGATGACCTGTGGAAAAGGCAAGATTGGGACGAGGTGCAAGATGTTTTTCCGGACAACAAAAAAGGCAGCAGAGTATTGATCACTAGTCGTTTGAAAGAGGTGGCAGTGCATACTTCCCATGATCTTCCTTACTATCTGCAGTTCCTGAGTGAAGAAGAAAGTTGGGAGTTGTTTTGCAGGAAAGTGTTTAGGGGTGAAGACTACCCTTCTGATTTGGAAGCTCTTGGAAAGCAAATGGTTCAAAGTTGTCGTGGTTTGCCACTCTCCATCATTGTCTTAGCAGGGCTGCTAGTCTACAAGGAAAAGTCATACACAAAATGGTCAAAAGTGGTGGGTCATGTTAATTGGTATCTTACTCAAGATGAGACCCAAGTGAAGGATATAGTTCTCAAACTCAGCTACGACAACTTGCCAAGGAGATTAAAACCATGTTTTCTCTATCTTGGGATATTTCCTGAAGACTTTGAAATTTTTGTTACACCATTATTGCAAAAATGGGTTGCGGAGGGTTTTATACAAGATACAGGGAATAGAGACCCAGATGACGTTGCAGAAGACTACTTGTACGAGCTCATCGATCGTAGTCTGGTCCAAGTAGCAAAACTGGAGACTAATGGAGGTGTGAAAACATGTCAGGTTCATGATCTTCTTCGAGATCTCTGCATATCGGAGAGCAAAGAGGACAAAATTTTTGAAGTTTCCACTGATAATAAGATTCAAATCACGACAAAACCTCGTAGACTCTCCATTCACAGTAAGATGGATCACTACATTTCTTCAAGCAATAATGACCATTCATGTATCTCTTCCTTGTTCTTCTTTGGCCCAATGTACTATGTTCGCGGGAAGGACTGGAAATGGATTTTAAAAGGCTTCAAATTGGTTCGAGTGTTGGAGTTTGGACTAACGGCTTCGAAAAGATCCCTTCCAATTTGGGGAACTTTATCAATTTAAGGTACTTGAGAATAAAAATGAAGTATCGTATATTTTTTCCAGCTTCA

>PvRGL113

GATAACTTCAGTGCGAAGAACATATTGGGACAAGGAGGTTTTGGAACGGTTTATAGAGGTGAACTCCATGATGGTACAAGAATTGCAGTGAAAAGAATGGAGTGTGGGGCAATAACAGGGAAGGGTGCGGCAGAATTCAAGTCTGAAATTGCTGTTTTGACAAAGGTTCGCCACCGGCATCTTGTGGCTCTTCTAGGTTACTGCTTGGATGGGAATGAGAAGCTGCTTGTGTACGAGTACATGCCTCAAGGAACATTGAGTAGGCATCTTTTCAACTGGCCAGAGGAAGGACTGGAACCACTGGAGTGGAATAGAAGATTGACAATTGCCTTAGATGTGGCAAGGGGTGTCGAGTACCTCCATGGCTTGGCCCACCAAAGCTTCATACACAGGGACTTAAAACCTTCAAACATTCTCCTTGGAGATGATATGAGGGCAAAGGTTGCAGATTTTGGGCTTGTGCGTCTTGCTCCGGAAGGAAAAGCCTCTATAGAAACAAGAATTGCAGGAACATTTGGATATTTGGCACCTGAGTATGCAGTCACTGGCCGTGTGACAACTAAAGTTGACGTGTTCAGCTTTGGGGTAATATTGATGGAGGTGATAACAGGAAGAAAAGCACTTGATGAGACTCAGCCCGAGGATAGCATGCACCTTGTAACATGGTTCCGCAGAATGTCCATAAATAAGGACTCATTCCGCAAGGCCATTGACTCCGCAATTGATCTCAACGAGGAAACTCTTGCCAGTATTCACACTGTAGCAGAGTTAGCTGGCCACTGCTGTGCGAGGGAGCCATATCAAAGGCCTGACATGGGTCATGCTGTTAACGTGCTTTCTTCTCTTGTTGAACTTTGGAAACCATCTGATCAGAATTCAGAAGATATATATGGCATTGACCTTGACATGTCCTTGCCACAAGCACTTAAGAAGTGGCAGGCTTATGAAGGTAGAAGTCAAATGGAGTCCTCTTCTTCATCTTCTCTTCTACCAAGCTTGGATAACACACAGACAAGTATACCTACTCGCCCATATGGATTTGCGGATTCTTTCACATCAGCAGATGGTAGGTGATCATGTGTGGTTGTAAATATTTGTTTTAGTGTGTTTGTTTATTCTCCAGTCCTTGCTTCTTGTGCTGGCTTTTCTTTGTGAACTTGTTTATCTTTTTTCATGGTGTACTTCTCCATGCTAAATTTTACACCCTCTTTAGCCCTTGTTAAGTACTTTTGCTGACAAACTGGGAATAGTTTCTGAGCCGATAATGTATTTTTCATCATCTCACATTCATGTTTTCCCCCTTTTGAGATGTGTAAATCAAATATTACTGTTGAACTTGTTGATTAGTTAATGTTAATTATATTACACTTGTTGAACCAATTTGGTTGCGAGTAGTATTGAGTGCA

>PvRGL114

CCGGGAAACAACGACACACGCACATGGAATTATTTGTGGGGGCTGTCCACACCTTATAGAACCCAAACCCACCGTTCACAGATCTCAAGGATCTCACAATTTCAGCTTTGAACTTGGCTTTTTCTTTTTTCTTGTTTTTCACCAATAAAGTTCTGCTCTTTTTGGTGTTCTCAGTGCTTACTCCTCTTGGTTGGATTCAAATTCTCCATTTCCAAGTTGGTCTGTGGTGGCAGAACAGGAAAAAAAAAAAATTTTGGCATCTGGGTTCTGTTCGGTTTGATGTTCATTGAATTGGGTAGTGGAAGTTGCTGTTTTGGTGAGAGTGGAGTTGCTTTATGTGGAATCTGATTCCAGAGACTGGCTTTTTCTGTTTGGATAAGGTGATATAGACTGGACATTCTTTGTGGGAATGGGGAGAGAAGTTGTTAATTATGGTGGCTTTGAGCACTGGTCAGTGGTTGTGTGAGGGAAACACAGTTGAAGGACTGATTGTAGAGTATGGAACTTAGTAGTTTGGTTTTCTGGCTACTGGGGTTGCTGCTTTTGCTGTTGATGGAGATTTCTTCTGCTGCTCTTTCTCCATCTGGCATAAACTATGAAGTTGTAGCTCTGATGGCCATAAAGGGTGCCTTGACTGATCCTCACAACGTTCTGGAAAATTGGGATTCTAGTTCTGTTGATCCATGTAGCTGGAGGATGATTACGTGTTCCTCTGATGGCTCCGTTTCTGCATTGGGTTTGCCGAGTCAGAACTTGTCTGGTACACTATCTCCCGGGATTGGAAACCTTACTAACTTGCAATCTGTGTTGCTTCAGAATAATGGCATTTCTGGTAAGATTCCTGCTGCATTAGGAAACTTGGAAAAGCTTCATACACTTGATCTTTCCAATAATGCATTTAGTGGTGAGATACCCAGTTCTTTGGGAGGCCTCAAGAACCTGAATTATTTGAGGTTAAACAATAACAGCCTTACAGGATCCTGCCCTCAGTCTCTTAGCAACATTGAAGGTCTAACCCTTGTGGACCTCTCCTACAACAATCTCAGTGGTTCCTTGCCTAGAATATCGGCAAGAACATTAAAGATTGTGGGTAACCCTTTAATTTGTGGTCCAAAAGCAAACAACTGTTCTTCTGTTTTACCAGAGCCACTTTCCTTCCCTCCAGATGCACTAAGAGGCCAATCAGACTCGGGTAAAAAAAGCCACCATGTGGCACTTGCTTTTGGTGCAAGCTTTGGTGCTGCATTTGTCATTGTGATTATAGTTGGGTTTCTTGTTTGGTGGCGATATAGACGCAACCAACAGATATTCTTTGATGTTAATGAACATTATGATCCGGAGGTGCGTCTTGGTCATTTAAAAAGGTTTTCATTCAAAGAGCTTCGAACTGCAACAGACCATTTCAACTCGAAGCACATTCTTGGAAGAGGTGGTTTTGGAATAGTTTACAAGGCGTGCTTGAATGATGGGTCTGTTGTGGCTGTTAAGAGGTTAAAGGACTACAATGCAGCCGGTGGTGAGATCCAATTTCAAACAGAAGTTGAGACAATCAGTTTGGCTGTCCACCGGAATCTTCTCAGGCTTTCGGGATTTTGCAGCACTCAGCATGAAAGGCTCCTCGTTTATCCCTATATGTCTAATGGAAGTGTAGCCTCTAGATTAAAAGATCATATCCATGGCCATCCAGCGTTAGATTGGAGTAGGCGGAAGAGAATAGCTTTAGGTACAGCAAGAGGGTTGGTTTACTTGCATGAACAATGTGACCCTAAAATTATTCACCGTGATGTGAAAGCAGCTAACATATTGCTTGATGAAGACTTTGAAGCTGTTGTTGGTGATTTTGGTTTAGCTAAGCTTCTGGATCATAGAGACTCCCATGTGACCACTGCTGTGCGTGGCACTGTTGGTCACATTGCTCCAGAGTATCTATCCACTGGCCAATCATCAGAAAAAACTGATGTGTTTGGGTTTGGAATCTTGCTGCTTGAACTGATTACAGGTCACAAGGCTCTAGATTTTGGGCGAGCAGCGAACCAGAAAGGTGTAATGCTTGATTGGGTTAAGAAGCTCCACCAGGATGGAAGATTAAGTCAAATGGTAGACAAAGATCTAAAGGGAAACTTTGATCTGATTGAGTTAGAAGAAATGGTTCAGGTTGCACTCTTGTGCACACAATTCAATCCTTCACACCGCCCGAAGATGTCAGAAGTATTAAAGATGTTGGAAGGGGATGGTTTAGCTGAGAGATGGGAGGCCTCACAGAGGATTGAAACACCGAGGTTTCGGTCTTGTGAGCCTCAAAGATACTCAGATTTAATAGAGGAATCTTCACTCATAGTTGAAGCCATGGAGCTTTCTGGCCCTAGGTGACAAACATCTTGGTTTTCCACTATAGCTCCTTGTTTAAAAGGTGTAGTTTAAATGTTAAAAATGACATTGTCATTAGTGTCAATAGCTATGTAATGCACGTATTCATCTGTAAAAAGAAAAAAAGGAAAGTCATGTATAAGTATCTTTGTTGGAATGTTCATTTTAGTTTCTTCAGAAATTTTTTGAATCAATGTTAGAAAATTGTATGTAGTTGCACTTAACATGATCTTGATGTTAGTCTTGGTGGCTAAGCAATCTCATAGAGCCAGTGGAATAAACTTGTATTTAGCCATTCCTAAAGTCTAATAAATTTCATTGGTTAGTTACCCTA

>PvRGL115

TGCATTTGGAAATTAAAATCAGTTTATATGTGTTTCATGTCTGATTTTTACTTCAGCATAGGTCTCAAATTTCTGTTCTTCAATTGTAACATGAATAGATCACTTCATTTCAAGTACATGTTTTACTCCTAAAGTTTGTAACATCAACTTCTAATCCATGATAATAAGTAACAATAAACAAATTCCTTGCCCCTTTTGGGCATTGCTTATGATGACAATATTGCTACCTTGGTTCTCATAACTATGTTGGAACACTCTTGTTGCAAGATCAATTGCAGGGTCCTCTTCTAAAAGACTTTAGGGTCTTACAGAACTCTTGTTTCTTCAAACTCACTGCATACCAGTGTCAAGGCCTGAACAACTTCACCCATAAAAGGACGCTGTGTGACTTCTGGTTGCACGCACATGGATGCAATTGCTGCAACCTTTACAATGGTATCAACAGAAACACTAGGCTTCATAACTGGGTCTATGATCTTCTGCAGACCCTCCTTGCTTGTAAGAAGTGGACGAGCCCAAGCAACAAGGTTTTCTTGACCTGGTGGCTGCGACAAATCCACAGGCTTTCTTCCACTTAGAAGCTCAAGGAGAACAACTCCATAGCTGTAAACATCACTTTTGACAAGAAGATGTCCCGTCATTGCATATTCAGGAGCTACGTAGCCAAATGTTCCAATAACATGTGTTGAGATGGGCTTGTTTCCCTCCTTCAGTGTTGTTCTTGCCAAGCCAAAATCTGAAACTTTGGGTATAAAATCATATTCCAATAAGATGTTGCTGGATTTGAAGTCCCTGTGTATGACACATGGATTACAATCTTCATGAAGGTAGGCTAATCCTCTAGCTGCACCAAGTGCAATCTTCNCCGGG

>PvRGL116

GCTGCAAGTCCTTGTTTTACGTGCTAATAGGTTCAATGGTACCATCAATCGTTTCCAATCCAAGAACAATACCTTTTCCAAGCTGACGGTTTTTGACATCTCCAACAATAATTTTCGAGGCAACTTGCCAATAGCTTTCATAAAAAATTTCAAGGGAATGATGATCAATGTCAATAATGGCTTGCAGTATATAAATGCTTCTCAATTGTACAATGATTCGGTGAAGGTCACAATGAAAGGCAACGACATGGAGCTAGAGAGGATCTTAACTACTTTCACAATTGTTGATTTAGCAAATAACAGATTTGAAGGAGCAATTCCAGCAATCATTGGAGAGTTAAAGTCACTAGTAGGCCTTAACCTTTCTAACAACAGAATCACTGGTTTTATTCCACCAAACCTCGGTGGTTTGGAAAATTTGGAATGGTTAGACCTCTCCTCAAACATGCTCATGGGTGAGATTCCAACTGCATTGGCCAATCTTCACTATCTCTCTTTCTTAAACCTTTCACAAAACCAGCTGGTAGGAAAGATACCAACAGGTAAACAATTCGACACATTCCAAAATGATTCCTACAAGGAAAATCTTGGGCTATGTGGTTTTCCTTTGTCCAAATCATGCCCCAAGGACGGAAAGCAGGAGAAAGATTCAGAAACCTTTCACCATGATGAACAATTTGGGTTTGGTTGGAAACCCGTAGCTATAGGATATGCATTTGGAATGGTCTTTGGAACGTTGTCGGGATATATTGTCTTCTTTGTTTGGAAACCACAATGGTCAATCTATTTTGTTGATGGCATTCTTAATCAAAGATCGAGAAAGAAGAGTCACCGAACTAATGCAAATACAAGACCACACAATGAGGGTCGTTGAAGAATCTCAGAATCAGTATAACAGGTGTGAAGTTCTAGTTCCATAGCGAACAAACACCATTTCACTGAAGGCCATTCTTCTGTCCCATTCCTTTTTTTTTGTCTCTTTTGAAATATGTGTTTTTAGGCTGAAAATGTTGAAGCAATTTGTTGTAATAAAGTACTC

>PvRGL117

AGGAAGGAAAATTTCCAACATCATGGCCTTTTTATCACGAGGATTGTGATTCAACAACCGCGGGCAAGGCATCAGCCCGGAAGCATAGCCACACAAGTTTTTGTTGGATTTCAATGCTTCAAATGGAGCGTTAAGGAAGGCTCGACTATTAGGAATTGGACCCTCTAATTGGTTGTTAGATATGTCAACATTTGTCAAACTCATCATATCCTTGAAACTGGAAGAAATGTTTCCAGTGAGATTGTTGTGGGAGAGGTTCAAGTTTTCCAACATCTTCAATTTTCCAAGTGCTGCTGGAATTTTTCCATTTAAAAAATTACAACTAAGATCAAGATTTTGAAGATATTGCAATTGGCTAAATTCAGATGGAATGCTTTCCATTAATTTGTTATGGCTTAAGTTCAAGTGATATAACTTGTGCAACCTTCCAAACCGTTTTGGGATTGGACCACTTAAACCATTTGCTGCAAGATCAAGGTACTGAAGTTGCTCTAAGGAACCAATTTCAGTGGGAATGTTGCCCGAAAGTTGGTTCTTACTTATGAAGAGTTCAGACAAGTAGGTCAAGTTCCCAAGTTCTTTTGGAATCTTTCCTGTTAGACGATTTGAAGACAGTTCAAGCTTTTGTAATTTAGGTGC

>PvRGL118

CCCAGTCGAGGAGATTCTGCGCACTCAAGTTCGTATCAAAGGATGAATTTTTCTGGCCACTGACCAATTCCAACACCAAAACTCCAAAGCTAAATACGTCTGCCTTCACAGATAAATGGCCATGCATTAAATATTCTGGAGCCAAATACCCGTTGGTTCCAGCCACACGTGTGTTGACATGGGTTTGGTCTTCGGGGAAGAGTCGGGCGAGACCAAAATCTGCTATCTTAGGCACCCATTTTTCATCGAGTAAGATGTTACTGGCCTTTATGTCGCGATGGATTATGCAGTTGTGAGAGTCTTCGTGAAGATAAAGCAAACCCCTTCCCACCCCACTGATGATGTCAAATCTACGTTTCCAGTTTAGCTCTTCTTTCTTCCCCGATTTGAAGAGAAGCTTGTCAAGGCTCTTGCGAGGGACATACTCATAAACAAGAAGCTTCTCGGAGGCATGGGCGCAGTAACCGATCAGATTCACCACATTTCGGTGCTGCACACGACCCAACAACCT

>PvRGL119

TGGTAGGGAAGAAGGACTTTGAACAGCAGATGGACTTTATTGGAAGAGTTGGACAACACACAAATGTGGTGCCCCTTCGCGCTTATTATTATTCAAAAGATGAAAAGCTTTTGGTTTATGACTATATCCCTGCTGGCAACCTGCACACACTCTTGCACGGTGGAAGGACAGGTGGAAGAACTCCCTTAGACTGGGAGTCCCGAATAAAAATATCCCTTGGAAGTGCCAAAGGACTAGCCCACATTCATTCTGTTGGTGGTTCTAAATTCACACATGGAAACATTAAGTCCTCAAATGTCCTCCTTAACCAAGATAACGATGGCTGCATTTCTGATTTTGGCCTCGCCTCCCTTATGAACGTTCCTGCTACCCCTTCAAGAGCTGCTGGTTATCGTGCTCCAGAGGTGGTTGAAACCCGTAAACACTCTCATAAATCAGATGTGTACAGCTTCGGTGTCCTCCTTCTTGAGATGCTTACTGGGAAGGCTCCTCTCCAATCTCCTGGACGTGATGACATGGTTGATCTCCCTAGGTGGGTTCAGTCTGTTGTTAGGGAAGAATGGACTGCCGAAGTTTTTGATGTTGAGCTTATGAGGTACCAAAACATTGAAGAAGAAATGGTGCAGATGTTGCAGATAGCCATGGCCTGTGTGGCAAAGATGCCAGATATGAGACCTAGTATGGATGAAGTAGTAAGACTGATCGAAGAGATCCGGCAATCTGACTCAGAGAACCGACCATCTTCTGAAGAAAATAAATCTAAGGACTCAAATGTTCAGACTCCATAGATTAACTGTGCTTAGTGACGATATAAGAG

>PvRGL120

CCGGGGGGTTTTGAGAGATGGTTCTGTTGTGACTATAAAGTGCATTGCCAAGACAAGTTGCAAATCTGATGAAGGTGAGTTCTTGAAAGGTTTGAAGATTTTGACCTCGTTAAGGCATGAAAATGTAGTTCGGTTGAGAGGGTTTTGCTGTTCAAAAGGGCGCGGGGAATGTTTTCTGATCTATGACTCTTTCTCTAATGGGAATTTGTTACAGTATCTTGATGCCAAGAGAAACAGTGGTAGGGTACTTGATTGGTCGACCAGAGTATGGATCATTCGTGGTATCGCCAAAGGTATGGATTATCTGCATAGGAAGAAAGGAAGGCAACATGGTAGAGTTCAGGAAAATATATCAGCTGAAAATATAGTTCTGGATTCTGGGTACAAGGCTTTAATTGGAGATTCAGGATTGCACAATATCCTTGAAGATGATGTTGTTTTTTCCGCCCTGAAAGGTAGCGCTGGCATGGGATATCTGCCTCCAGAGTACAGGAGAAGTGGTGGTTTGAGTGAAAAGAGTGATGTGTATGCCTTTGGAGTGATTGTTTTCCAGGTTCTGAGTGGAAAACGTGATATGAGGCAGTTAAATTTGGAAAGTGGTAGTTTGAAAGATATAGTTGATGAGAATCTTG

>PvRGL121

GTTTGTGGTGATTGCAGGTTTCTTGATTGTGTGCTGCTATCAGAATACAGATGTGAATGTGCAACCAATGAAATCCCAGAAAAAACAAGCCAATCTGAAAACACAATCTTCTGGAAGTCAGGACAAAAACAACAAAATAGTCTTCTTTGAGGGTTGCGATCTTGCATTTGATCTAGAGGACCTGTTGAGAGCATCTGCTGAGATTCTTGGCAAGGGCACCTTTGGCATGACATATAAAGCTGCTCTAGAGGATGCAACCACTTTGGTGATCAAGAGGTTGAAGGACGTCACAGTTGGAAAACGAGATTTTGAACAGCAGATGGAGCTGGTGGGGAGAGTAAAGCACGACAATGTGGAAGCAGTGAGGGCATATTACTACTCAAAGGAGGAGAAACTCATAGTATATGATTACTATCAACGAGGCAGCGTTTCTGCAATGTTACATGGCAAAGGAGGGGAAGGTAGAAGTACTTTAGACTGGGATAGCCGTTTGAGAATTGCAATTGGTGCAGCGAGAGGCATTGCTCACATCCACGCACAACATGGAGGGAAACTCGTTCATGGAAACATAAAAGCCTCAAACATCTTCCTCAACGTACAAGGATACGGATGCATATCTGATATTGGCTTGGCTACATTGATGAGTCCAGTACCTGTCCCAGCCATGAGAACAACTGGATACCGTGCACCAGAAATAACTGACACGCGCAAAGCAACCCAGGCATCTGATGTCTACAGTTTTGGGGTGCTACTACTCGAGCTTTTGACTGGGAAATCGCCCATAAACAGCACAGAAGGTGAGCAGGTTGTCCACTTAGTTAGATGGGTGAATTCTGTTGTTAGAGAGGAGTGGACTGCAGAAGTGTTTGATGTAGAGCTGTTGAGGTATGCGAACATAGAGGAAGAAATGGTGGGGATGCTACAAATAGGGATGGCTTGTGCTGTAAGAATACCGGATCAGAGACCAAAGATGCCTGATGTGGTCAAAATGGTGGAAGAGATTCGTCGTGTAAATACGCCAAATCTACCATCAACGGAGTCTCGGTCAGAAGTTTCTACACCCACACCTCGTGCAGTGGACATACCTTCTACCTCTGTTCAACAATGATATTCTTTTGTTCTGGTTAATAGTCGTTAAGATTTAACCAATTCAATTATATTCATAACAGTAGAAATGATATCCATCAAAAGGACTTCTGGTCCTAGGAAGTTTGCAAAACATATTTCTTATGAGTAGTTAAGCTACCAGTAATATCATACCCGGTCCCTTCGCTCGTAATTATTGTGTTATATTATGCAGTCAAGTAATCTTTCGTGTCCA

>PvRGL122

TTAGAGTTATTGTGTGGTTTTTAGGGTTTATTCTCCAGTATAAATTATTGGGATGTGTGTGTTCAACAGTACAAAGATCTTAAAACAGACTGCATAATTATAGTAACATCCAAAGAGCTAGAACTAAAACTCTGAAGCAAACAGAAAACAAAAAAAAACATAGTCTAGTGGCTTTAAGAAAAGGTACAAAAATTACACCAACACTCCATTATTCCAACCAACTCAAACCCAGATAAGAAAGTTCATCATCTCACTTTAATCTTCAGTTTCAACCTTGGTTCAATGTTGAGACTGTTAATTATAGTGAGAACAGATTGTTGTCTACCTTCACCCTCTTCTTCACCTCCAATAATAGTAATTCCAAACCCTAATCCTTGTATGGGAGTTCAATACATCTAATAACTGGAAGATCTTCTGAATTTATTTGTAGCAATGGTTCTGCATGTGGAACTGTGTACCAATTTCAGAATTAACTTTATGTCTGAGATTGAACACCAATAACGACACATATGTAACAATCTCTTTAACAAGCTTAGCATCATTCCGAAAAGTAGATGAATCAAATCCTGCTACATTTGCAGATTCCTTCAAAGCAAATCTCCATGTTTGCACAGTGGTTAGATTGTATTGTTTTTCATGAGCAACAAAGGCCTCTCCATAACTTCCCCTCTGATGTCGCACATCTGAGGGATCTACTTTGTAGAAAATAGGTAGCAAGATGTGTCCATATTTTCTACTACCCTCAACAATTTTCGCAAGTTCTGACAAACACCAGCTTGAAGAGGCATAATTTTCTGAAAATATAATCAAATAAATAGACGATACCTCAATTGCTCTTAGAAGTACATCAGATATTTGATGTCCTTTGGGAATGTCGTAATCTGGAATGAGTATTTCTGAATTTTCAGCACTCGGATGAAGATTGTGTAGGTTCCTTTGAGGGAAGATGAGTTGAAAGAGCTTCTTCAT

>PvRGL123

CGCTGCTTCCGTCACCCCAGGCTCCTGCATCTACTCATGGGACTTCTCCTTTGATCCCTGCGACAACCTCTTCAGTGACAAGTTCACCTGCGGCTTCAGGTGCGACGCCGTCGTTTCGGGAACCTCTCGACTCACCGAACTCACACTCGACCCCGCCGGTTACACCGGCACTCTCCCCGCCGCCTGGAACCTCCCCTACCTCCAAACTCTCGACGTCTCAAACAACTTCTTTTCGGGCCAAATCCCCGAGTCATTCTCCAACCTCACGCGGTTAACTCGACTCGGTTTATCCAAAAACTCCCTCACCGGAACCATACCCTCTTCCTTATCCTCCCTCTCAAACCTCCAAGAGCTTTACCTCGACAACAACAACCTCCAAGGACCAATCCCCGTTACTTTTAACGCCCTCACCAACCTTCAACGCCTCGAAATGCAGTCCAACAAGCTAAACGGCGTCGTTCCCAGTTTGAGCTCTCTGAAGAGTCTTTACTACTTGGACCTCAGCTTCAATTTTTTCGGCGGAGGGTTCCCCTCCGCGTTGCCCGAATCTCTGATTCAGATTTCAATGCGGAACAACAGCTTAAGCGGCGCGTTGGAACCCGAATCTCTGAGGAGCTTGAACTCCCTGCAAGTGGTGGATTTGAGTGGCAACGGGTTCAGCGGTGCCGTTCCGTTTGCTCTCTTCGAGCTTCCTTCGTTGCAGCAGGTGACGCTCTC

>PvRGL124

TAGAGATTGCTGAATGATTTTGACAAGTGACAAGTAATAAGCAAGCAGAGGAGTCACAAATTCGAACCATGTTTCCAAAATGTTTGGGTTTCTGCGGTTGGAAGCACACTAGTTCATCTCAGAAACAGCATCCAACGCTAATACATGAGTTGTGCCAGCAATTTTCTCTCACCGATCTTAAGAAATCGACAAACAACTTTCATCATACCAGACTAATTGGATACTCAAGCTTTGGTAAACTATACAAAGGTTGCCTCCAACACGACGATGGTTCTGAATATTCAGTTACAATAAAGCCATTATATGGAGAAGATAACATCGAAGGAAACAACCAGTTTAACAAAGAAGTAGAGTTGCTCTGTCAGCTTCATCACCCTAATTGTGTCTCTCTTATAGGATTCTGCAATCACAAAAAAGAGAAAATTCTTGTGTACGAGTACATGTCCAATGGATCTCTAGATCAATACATACAAGGTAAGTTTAGAGAAGCACTGTCATGGAGGAAAAGGATAGAGATATGCATAGGAGTTGCTCGTGGACTGCACTACCTTCATGCTGGAGCCAAACGCACCATCTTTCACCGTGACGTTTGTCTCTCAAACATTCTTCTGGACGACCAAATGGAGCCAAAACTAGCAGGTTTCGGCCGTAGCATACAGGGAGCACGCTTTATGTCAAAGCCAAAAACAATTCCAATAGATGATGATACTTGGGGTAGTTATGGCTACCATCCTATGCAGCACTTCAGAGACACTATCGTCACAGATAAATGTGATGTTTTTACATTTGGTATGCTTCTTCTTGAAATAGTCTGGGGAAGAAGGTATTTTGCCATGAAGGAAATAGATGGATTTCTGGAGAAGCCTGTTGAGGAGAATATTGATATGGATATAAAAGGAAAGATTGCGCCAGAGTGTTGGAAAGTGTTTATAGATATCATCCAAAGATGTGTGAATTCTGCAGCAGATGAGCGACCAACAATGGGTGAGGTAGAGCTGCAACTTGAGTATGCTCTGTCGTTGCAGGAACAAGCAGATAGCACAAACATCCATGGTGATTATATCTTATTGTCTAAAACCATTGTTAACCTAAAGCCTTAGTTGGAAGATGAGAGTCATAAATTTTAGTTATTGTTTCCGATATATTAAAAACAAATTACGTAACGGAAGTGATTCCATATTCAAGTGTTTTTTTTGTCTTTGAATACAAAGAAGTGAAAGCGTGTTTCACCA

>PvRGL125

TGTCCAACTAAATTTAATGCATCACAATAGCATCTTCATACACCCATGAAAACATGAATTTAACTCTCTTGAAAGAGGAAAAGTAAAATTGTTACTATTGGTTTGAAGTTGAGTGCACCAAATAATCAATAGAGACCAGAAAAATAAGTACTTTGGGTGCACCAAGTTTGTTACTCTTCGGTGGGAAAAAGACCAGTGTTCCTTGTGACCCTCCCAGTATAAACAAAAAAACTGAGTAGCTACAAGTCTTAGACACCACCAGGCGAAGAAGAATCATAGAATGCATTATGACAAAGCTCACCAAAGTACAAAATACCCACATCCTCAACATAAGTCTCAATGTCAGGCTTTCCCAGTTAGAACAAATGTTAGCATCGAAGAATGAATCAAGCTTTACAAAGCTTCATGTGTATCAAATAATTCAGGAATTGTACTTAGGAAACAAGTTAGACCGTGAAAATCACCGGGGAAAAAAAAAGACACAGCTCTAAGTAAGTACTATTCATTGTGTGCAAGCCAATTAATATATTGGACCTACAAAATATACTGACATTATATGTACCTGTACCAGTCTCATGGGTGTGCACTAGCAAACTGATGTCTGAAGCGTAATTTTGAAAGACGTGGGATTCATCAATCAAGAGCTTGTCTCAGAGTCACGGTTGCTGCCCTGCACGTTCCCCCAATTCAATTTCCTGCGTATTTTGCTGTCCCGAACTGGTAGAGACAGATGGATGTCGACCGTAATCACTGCTGGGACCACTGTACTCACTAATACCTTGCTCTTGGCTTTCCAGTGCCAGCTTCCTGAAGTTCTTCATGTCTTCACTGTATTGGACACTATCATAGCTAGAACTCTCAAAGGAACCAAAAACTCTGCTGTGTCCAGGTGCGCTCCCCTCGTTTAGATCTTCTAGAGAAATATTTCCTTCCAGAGCTCGAACAACCTGGCTCATTCGAGGTCGTAGCCTGGCTGAATGGCGCACACAACTTGCAGCACAAGCAGTCATTCGAATCATCTCATCGTGATTGTAATTTCTCTGCAACTTTGGATCAACAAGTCCATCAAAGTTGCCATTCTCAAAAGCTTGAGACAGTAAAGGCCTGGCCCATTCAACCATGCTGTCATCTAAGAAGGGATGACTTTTATCAACGGGTTTCCGTCCAGTGATCAACTCCAAAAGCACAACACCAAAGGAGAAAACATCTGACTTTTCAGTTAGTTTTCCACTAGCAGCATATTCTGGAGCCATGTATCCAAAAGTTCCCATCACTCGAGTAGAAACATGGGTTT

>PvRGL126

GGAGGATTTGGTAAGGTGTACAAAGGACGGTTGGCAGATGGTTCACTGGTGGCTGTTAAACGGTTGAAAGAGGAACGCACACCTGGTGGGGAGCTTCAGTTTCAGACTGAAGTAGAGATGATTAGCATGGCTGTGCATAGAAACCTCCTCAGACTACGTGGGTTTTGTATGACACCAACAGAAAGGCTACTTGTTTATCCATACATGGCTAACGGAAGTGTTGCCTCATGCTTAAGAGAGCGTCCTCCACATCAAGAACCCCTGGATTGGCAAACTAGGAAAAGAATAGCTTTGGGATCTGCAAGGGGTCTGTCATATTTGCATGATCACTGTGATCCAAAGATTATTCATCGTGATGTGAAAGCTGCAAACATATTGCTGGATGAAGAGTTTGAGGCTGTTGTTGGGGACTTTGGATTGGCCAAACTCATGGATTACAAGGACACCCATGTGACAACTGCTGTAAGGGGCACAATTGGGCACATAGCTCCTGAATACCTATCCACTGGTAAATCTTCAGAGAAAACTGATGTTTTTGGTTATGGTATCATGCTTCTGGAGTTGATCACTGGACAGAGAGCTTTTGACCTTGCTCGGCTTGCAAATGACGACGATGTTATGCTGCTTGATTGGGTAAAAGGACTCCTGAAGGAGAAAAAGCTTGAAATGTTGGTTGATCCTGATCTACAAAAGAATTACATAGAAGCCGAGGTAGAACAATTAATCCAGGTTGCACTGCTCTGCACACAAGGTTCCCCTATGGACCGACCTAAGATGTCAGAAGTGGTGAGAATGCTGGAAGGTGATGGCTTGGCAGAAAGATGGGATGAGTGGCAAAAGGTGGAAGTTCTCCGGCAGGAGGTGGAGCTGGCCCCTCATCCCAATTCTGACTGGATTGTGGACTCGACCGAAAATCTGCATGCAGTTGAGTTATCTGGTCCAAGGTGACCTTGGCACAGTAGTAAATTAAGA

>PvRGL127

ATTACCTAAAGAGGCACAGTCTGTGGGCTCAGGGAATGCAGGAGAATGTCAATCAATCTGCTTGAGCATCTGCTCCTGCACAGCTTATGCATATGACAGTAACGGATGTTCAATTTGGGACGGCAACCTTCTGAACGTGCAACAGTTGTCTTCAGATGATAGTAGCGGAGAAACTTTGTATCTTAAACTTGCAGCATCTGAGCTTCATGCTGATAAAAGCAGTAAGGGGACGATTATTGGTATAGTTGTGGGAGTGGTTCTTGGCGTTGGGATTCTCTTGGCCATTCTTTGGTCAAGGAAGAGAATGTTGGTATCAGGAAAGGCTGCAGAAGGTTTGGTGGTATATGGGTACAGAGATTTGCAAGATGCAACGAGAAATTTCTCTGAGAAATTGGGAGGAGGAGGGTTTGGTTCTGTATTCAAAGGAACATTGGATGATTCCACTGTGGTAGCAGTGAAGAAGTTGGAAAGTTTTAGCCAAAGAGAGAAACAGTTTCAAACCGAAATTCGACAATAGGGACAATACAACATGTTAATCTTGTCAGACTCCATGGATTCTGCTCTGAAGGCACAAAAAAGCTGCTAGTTTATGAATACATGGCAAATGGCTCCTTGGATTTGCACTTGTTCCAGAACAAGAACTTCGTGGTATTGGACTGGAAAACGAGATACCAAATTGCTTTGGGAATAGCAAGGGGATTGGCTTACCTTCATGAGAAATGCAAAGACTGTATTATACACTGTGACGTAAAGCCAGAAAATATTCTCCTAGATGCTGATTTTTGTCCCAAGATTGCAGACTTTGGCCTGGCTAAGCTTGTAGGAAGGGATTTTAGCAGGTTCCTCGCAACAGTTAGAGGAACAAAAAACTATCTTTCTCCAGAATGGATTTCTGGGGTGCCTATCCAGCCAAAGCCGACGTGTACAGCTATGGAATGATGCTTTTTGAGTTTGTGGCAGGTAGGAGGAACTCTGGTGAGCTATCTGATTGTGATGAAGATGACCAATTTACGTTCTTTCCTACCAGGGCAGCAAATATAGTTGCCCAAGGTGGCAATGTTCTTAGCCTTTTAGATCCTTCTTTGGAACAAAATGCTGACGCCGAGGAGGTGACTCGAATGGCAACGGTTGCTTCCTGGTGTATCCAAGAGAATGATACTCACAGGCCAACCATGGGTCAGGTAGTTCATATCCTTGATGGGATCTTGGACGTGAATTTACCTCCAATTCCAACATTTCCTCAGAACTAGAGAATCTTGTTTAATTTCCTAAACCAACTCAAACTCAAGCTCAAACCAGGAGTTCATGTGTGAAGATCAACGACTCAACCACCTCTCTTCAAACAAGCCCCATGGCCTTGGCACGAGAAAATTAGGAGCAGACGTCTGTCTCATTACTTGTTACTAATACAAAATATGACATCTTTTTTATTGTCACCAGTGTAATTCTCTTCCT

>PvRGL128

TGGAAAGCCCCGAACCAAGACTCAGAATAACAAAAGGGACTGAGAAGAATAAGGTAGTAAGAGTGTAAGACAAAGGACACATACACAGGTGGTTTCTATAATCCCTCAATGAATAAACCAACAAACAAACAACACTTGTTGTGTAATAATATGCATGCCAAAAACAGGCCAAATATCAAACAATGAGGTCTTTCTTCAGTTCTATATTCAAGCTTGGGTGTGACAGTGATGAAATAAAAGGGCTCTTTCTTCAATTCTGTATTGAAGCTTGTGACACACCCGAGTCAACAGAGTAGTATTGTTGGGACAATCCATTATCAGCATCTTTAAAATCATTGTTCTTCTCTTCCTCTTTCTCTAAACTAGGATGACAAATTTCCTCAATCTTGCTTTCCACCACATCCATTGAAGGTCTTTTATCAGGATATTGGGCAGTGCATTCAAGGGCAAGCTGCAAGAGCTTCACCATTTCCTCCTCAACACTCTGGTACCTTAGAAGCTCCATGTCAAACACATCAGTATTCCACTCACCTTGAACCACAGATTGAACCCATCTTGGGAGGTCCACCCCTTCGTCATTCAGTGAAGAATGAGCAGGAGCCTTCCCTGTAAGCAGCTCCAAAAGCATGATACCAAAGCTATAGACATCAGCTTTTTGGGATACTTTGCGCGCATCAGTGATCTCTGGTGCTCTGTAACCAGAAACACGGTTTGGAGTAGAGGTTGGAAGAGCAAGGTAAGCAAGGCCAAAGTCAGAGACACGAGCTTCAAAAGATTTGGTGAGAAGAATATTCGATGACTTGATGTTTCCATGAGAAGATGTTGGGCCATGCGAATGAATGTAGGCAATCCCACGAGCAGCACCAAGGGCAATGGCAGACCTTGTTTCCCAATTCAGTGGAGTCCTCCCCACCCCACCATTTGCATGTAAGAGAGCAGATAAGCTTCCCATAGGCATGTAATCATAGACAACAAGCTTCTCATCCCTGCTAAAGAAATAACCCCTAAGTGGGACCAAGTTGTGATGAACCATCTTCCCCACTTGCTCAATCTTCTCCCTGAATTCCCTCTCTGTGGCAGTAACATCCTTCAACCTCTTCACCGCCACACTCACCCCCATTTCCAAAGTCGCCTTATAAGTCGTTCCAAACGTTCCTTTCCCCAACACTTCCGCAGAAGCCCTCAACAACTCGTCCAAACTAAACACTCTATTCACATTCCCAAAAAACACCAAACTCTTATCTCCACCACCACTACTTTGAACCTCACTCTTCTCCACCGAACCTGCCACTGCGGAACCTGAATTCCC

>PvRGL129

GAAAAATTAGGCACAAGAACATTGTCAAACTGTGGTGTTGCTGCACTACTAGGGACAGCAAGCTATTGGTTTATGAGTATATGCCGAATGGTAGTCTGGGTGATTTGCTGCATAGCAATAAAGGAGGGTTGTTGGATTGGCCAACTAGGTATAAGATAGCTGTTGATGCTGCAGAAGGGCTCTCTTATTTGCATCACGACTGTGTTCCTTCAATTGTTCATAGAGATGTGAAATCTAATAACATCTTGTTGGATGGGGACTTCGGTGCAAGGGTGGCGGATTTTGGAGTGGCTAAGGTAGTTGATGCCACCGGGAAAGGAACGAAATCCATGTCTGTCATAGCTGGCTCTTGTGGGTACATTGCACCAGAATATGCATATACACTTAGAGTGAACGAGAAGAGTGACATATATAGTTTTGGTGTTGTCATACTTGAGTTGGTTACTGGAAAGAGGCCCATTGACCCTGAATTTGGGGAAAAAGACTTGGTTATGTGGGCTTGCACCACCTTAGATCAGAAAGGCGTGGACAGTGTGATTGACTCGAGGCTTGATTCTTGTTTCAAAGATGAAATTTGCAAGGTCCTCAACATTGGCCTCATGTGCACCAGTCCTCTTCCAATCAACCGGCCTGCAATGAGAAGGGTGGTGAAGATGTTACAAGAGGTGGGCACAGAGAACCAAACAAAATCAGCCAAGAAAGATGGAAAGTTGTCCCCATATTACTATGACGATGGCTCTGATCATGGAAGTGTTGCTTAGTTCAAGTTCTTGCAAGGGTTGATAGATTCTTTGGAGCGGGAGCTGAAAGGTCCAATCTTTTCTTTAACTTAACAGAGCCAAATTTCCCGTGGTGTTTTGTCGGCCATGGTTGGCTTCACAAAGTTGGCTAAAAATAGGTGAAGTAAATCTTTTTAGGGGTTGAAAATAGAGTAAGTTCAGGTTTTTGCCCTCTGAAGTTGTTCAATATCAAGGCGCGGCCCTACACAATTTAAATTGGTGTCTGCATTAATGTCTCTTGTGTATTCAGAAACCAAGTCTCACTGTTAAAGATCAGACTAGATGTGACCCATCTCTGGTATGGGTCAGGTGAATGTTTGAATTGTAAAATCAGTAGTGACTTCTAAAATTGTAATAGAAAATCACCCCTATAGTAATTTCAATTAACTTCAATCACAGTGATAGAAAAATTAACACTGGATTGGGAGAATCTGTATCTAAAGCATTCTTAAATGGTTGTAATTCTCTGGAGATGCTCCTGCATAAATGGTTTGACTGTTGTCCTCATAAAGATTTACTTCTGGCAGTGGTCTCAAGTATGTG

>PvRGL130

GATTGCTACTGACTTAATAATCTTGCATGTTCTCAATTAGTTAATTATATTATTCATTTTTCTGTACTGAGTTTTGTGATTCAGTTATCAGATTTTGAATTTGCTATGGTTTCTGGAATGCAACATAAGAACATGAAGATGTCAGGAACTTTAGGATACGTGGCCCCTGAGTACATATCACATGGTAAACTAACTGACAAGAGCGATGTCTACGCTTTTGGGGTTGTCCTTCTAGAACTCCTAACCGGAAGAAAACCCATGGAGAACATGACCTCACATCAATATCAATCCCTTGTATCATGGGCCATGCCTCAGCTAACTGACAGATCGAAGCTTCCAAGTATTCTTGATCCTGTTATCAGAGACACAATGGATTTGAAGCATTTATATCAGGTTGCTGCAGTGGCTGTACTCTGCGTGCAAGCAGAACCAAGTTATAGGCCATTAATAACAGACGTGTTGCACTCTCTCATCCCTTTGGTACCTGTTGAGCTTGGAGGGTCACTAAGAGTTACGGAACCAATCAGCTCAGAGAACTCTCATTGATATTGGATAATTGTTGTGTTTTCATTTTTCAACTACTGTATTTCTGCAATTTTTAAACATTTTATTATTATAATCATGGTTATTATTGTTACAAAGAGGCAAAGAGGAATATCCAAGTGTAATTATTTATGATTGCA

>PvRGL131

GGGGCCACCTTAAACTGGATAATCTCTTCATGGGGTGCGAGGATATAAAAAAGTGCAGTAGTATGACACAGTCACCGGGAAGTGATGAATTTGCTAAGGACACACCTGAGAATGATGTTCATCGGCTGTTAATTACCGGAGACGAGACTAATGAAGTTTTTCAACAATCTCCTGTCATCTTACCTAACGCATCAATCAGAAATGCACCAGCACCAGCACCAGCACCAGCACCAGCAACAGTTCCATTGCCTGCTCCTTCATTATCGATATCGCCATTATCAATACCAACAGAAAGAACCACATTAGGAAAACACCTATTTTGGAAAAGCAAAGTCATTATAGGCGTTACAAGTGTTGGTGTGGTAATGGGAGTATTCACTATATGTATCATAATTTATTGTTTTAGATACAAGTTATTCACTCCATCGATGAAATTTTGTTTGAACACAAAGAATGACCAAGATATTGAAGCATTCTTAAAAATTCATGGAGCTGAGTCACAAAAAAGATACAAATTTGCAGAAGTCAAGAAAATGACCAATTTCCTCAAAGTTAAACTTGGTCAAGGTGGTTTTGGTGAAGTGTACAAAGGACAATTACCCTCAGGCATCCCTGTGGCTGTAAAATTATTGAATGCATCGAAAAGAAATGGAGAAGAATTTATCAATGAGGTTGCTAGCATTAGTAGAACCTCTCATGTTAATATTGTGTCACTTCTTGGATTTTGCTTTGAAGGTCACAAGAAAGCTCTCGTCTATGAGTTTATGGCTAATGGTTCTCTTGAAAAGTATATATATAAAAGAGAGGCAGAAAGCATTATACCATTGAGTTGGGAGAATTTATACCAAATCTCAATAGGAACAGCTCGAGGGTTGGAGTACTTACATAAGGGATGCAACACTCGAATTTTACATTTTGATATAAAACCACATAATATTCTTTTGGATGAGAACTTTTGTCCCAAGATATCAGATTTTGGGTTAGCAAAACTTTGTCCTAGAAAAGAGAGCATTATTTCCATGGCGGATACTAGAGGGACAATGGGGTATGTAGCTCCAGAAATGTGCAATAGACAATTTGGTGGAGTTTCACATAAGTCTGATGTTTACAGTTATGGGATGATGTTGCTAGAAATGGTAGGAGGAAGGAAGAACATTAATGCTGAAGCTAGTCATACAAGTGAGATATACTTCCCACATTTGATACACAAGAGGCTTGCGTTGAACAAGGATTTAAGACCTGATGAAGAGATGACCACAGACGAAAATGAGATTGCAAAGCGAATGACTATAGTAGGTTTATGGTGCATTCAAACGTTTCCAAATGATAGACCTACAATGAGTAAAGTAGTGGACATGCTAGAAGGCAACATGAATTTCTTTGAAATGCCACCAAAACCTATGTTTTCTTCACCTACAAGATCGACAACAGAATCTTCCTCTTAAATTTAATTACTACATCTGGATTCTAGGCAAATGTTTGAAGTTACTTTGTAAAGTCAACCTCTCAACATATTTATTTGGTACTTTTGAATATGTAAAGCTAATGGATCTTTTATGTGCATCATCAAACTAATGTTACATTTAATGCAAATAAACAAATATCACTACTAGAAA

>PvRGL132

CCTGATGCGCAGCCATTTTTGGAGGAGGCCAGGGCTGTTGGTCAACTGAGGAGCCAGCGATTGATAAATCTGCTAGGATGTTGTTGTGAAGGTGACGAGAGGTTACTTGTTGCAGAATATATGCCCAATGATACGTTGGCAAAACATTTATTCCATTGGGAAAATCAGCCCATGAGATGGGCAATGCGAATGAGAGTTGCATTATGCCTTGCCCAAGCTCTAGAGTACTGTACCAGCAATGGACGTGCACTATATCATGATCTAAATGCCTACAGAGTTCTTTATGATGACGACTACAATCCTAGACTTTCATGCTTCGGTTTGATGAAAAATAGCAGAGATGGAAAAAGTTATAGTACGAATTTGGCGTTTACTCCACCTGAATATCTAAGGACAGGCAGAGTTACTCCAGAGAGTGTAACTTACAGCTTTGGCACACTTTTGCTTGACCTTCTCAGTGGAAAGCATATTCCTCCAAGCCATGCCCTTGACTTAATTAGAGACAAGAACCTTCAGATGCTATCAGACTCTTGCTTAGAAGGGGAGCTTACAAATGATGATGGGACTGAATTAGTGCGTCTAGCCTCACTATGTTTGCAATCTGAACCACGAGAGCGTCCTAGTCCAAAGTCACTGGTTGCTGCTCTAATACCTCTACAGAAGGATTCTGAGGTTCCTTCTCGCGTACTGATGGGTATACCAGATGGTACATCTACTTTCCCCCTTACACCTCTAGGAGAAGCTTGTCTACGAATGGATCTGACTGCTCTGCATGAGGTGATGGAGAAAATGGGGTATAAAGACGATGAGGGCGCAGCAACTGAGCTTTCGTTCCAAATGTGGACGAACCAGA

>PvRGL133

TCGGTATAAAAAAACTAGTTATTTTTTTTATCAGTCTAAAATAAAATAAATTAAACTGAACATTTCAGGATGCTACAACCCTGAAACAGATAAAAAAAAACATATACCAAAATACAAATCATCTACTTTCTTAAGGCTCTTCCCAGCTTAAAACCAAGCTGCAATCACTCGTACAAAAAGAAACAAGAAAGAATCAAACTAAGAGTTATTACTTCACCAATTTCTGCTGCATTTTGTATTTCTTTCTGCAGCTTTTCATCCAGCTTTAGTGCAGGAGGTTAAGCTTGGTGAATCAATGGCTCCAAAAGAAAAGCCACGCTCACTGCTTAAAACTGCAGCTTGTCCGAGACCATCTATCAAAGCCTATACTAATGCGCATTGGCTTCTTCTGTTGTTGGCTTCTTCTAGTTTAAGATTTGAATCTTCTTCTCTTAAATTTCTTCCAGGATAAGGCTTTGCTCAAGCTTCTATCATTCGTAAGTTCCATATGTTGCTTCTCAGTACACCTTCCCACTCTGAAACAAATGACTTTGAATGTAAAAGACCTCCTACCATTTCAAGGGTAAGAGGCTGTCCTTGGCACTTTTCAACTATCTTCATGCCAATCTCCACGAACACAGAATTCGACTTAGAGTTATCATCTTGAAATGCATGTTTAGCTAAAACTTGCCAACTGTGATCTTTTTGTAATTGTAGTAGTTGGTGAATATTGTCTGACTCCACGCTATAAGCAACTCCTGTAAGGCGTGTTGTGATAAGAATTTTACTTCCCTTAGCACCATACTTAAGAGGAGTTTGCAATGATTTCCATAGGTCTTGGCGTCCATTCCAAACATCATCGAAAACAAGAAAAAACTTCATTCCATTCAATTTTTCTTTCAATCGTCCCTGAGCCATTTCTAGGTTTATGCTATTATCAGTTTCCTTACTGATTGACTCAATAATTTTTTGGGTTAACATGAAAACATCGAATTCATGAAATACATAAATCCAGGCTTTGATAGCAAATATATCCTCTATCATTGGGTGGTTGTATACATGTTTAGCAAGTGCACTCTTACCTATCCCGCCCATACCCACAATAGAAAGTATTGAAAGCTGGTTATGATTTCCATTGTCTGATGTCAACCAATTAAGGATATTTTTTTTTCCTCTTCTCTACCATAAATAACCTCTTGAGCCACCAAAGATGAGGATTCATTTCTTTTCC

>PvRGL134

CTAGACTTGCTCTAAGAAAGCTCAATGTTGCGCTCTCATTTCCACGATTTCTTTGCATAGGGAAGCCTAACTCAAATACAATCTCAACTTTATTCCACTCTTGCTCTGAAAACACCCCGTCCACTTTGCCTTCTACATCACACCAAAGTGTTGTTTTCCTGTTTCTTTCTATGAAATTGCTCAGAGCAATGAATTGCATACTCTCGTTAATGAATACTCTGAACTTGAAATTCATTGCCATCAAACCTTTCTGGAAAACCGCTGATGTTAAACAACATACCGCTATCTTAGGAAATTTGTTTCGAAACGAGAAATGAATCGATGATTCCCTGCGAACGTGCTTCTTTTTCTTTCTAAGCTGTTGGAATAACAAACGGAAATCAGAATGCATAATAGGATTAGGACTGCATACTTGGATGGCATTATTGGGACTTATGTATGAATAATACATATCAAGATATCGGTCATAACCGCCAATAAGCATGGCCCTTTCAGACACCTCTGAGCTCAACTCTTCAAAATTTTGATACGTTCCATGCCCATAACCCACTAACACCTTAACTTCCCGTATCATGCTTATACTATCTGGTAGCTGATAAAGTCTTCGGCAACCCTTCAAGGACAGCAGTTCAAGCCCAACAAAATTTCTAATTGAAAATGGCAATTTCTCTATATTTGTGCCGTCTAAATATATTTTCCTTATTTTCTCCATCTTTCCCAATACTTCTGGGAAACTCTCAAGACTTTCGCACCTCCGAAGATCCAAAGTTTCAAGAGATGTCAGCATGATGCAGGGTGCAAGAGTTTTCAGCTTGGTGCATTCTTGAGCACTCAATAAGCAAAGCTTATCAAGAAACCCAATTGATTCATCAATGTTAACCAAATTAGAGCATTTATCTAGACGCAGAGTTGTTAAGAGTGGTGCTTCTCGTAGGCTCGGTAGATCAGTTAAGAACTTGCAATCTTCAAGGTTTATTATAGACAATGATCCCAACATCTTTTCTGGTTTGAATATCTGAAGGCAACTTTCAGGCATAAGGAATATCTCAAAACGTTTGGGATTGAAATTAGAAGGTAAAGATGGTGAAGGATAGCAACTCCAGTCTAGCACTCTCAGGCTATTTGGTAGATGCTCAGGGCCAGTAGAAAAGGTTGAATCTTCAATGATCAAAATTCTCAAGTTCTTCATTTCCCTGAATGCCTTTCCATTCCATTGCACTTCTATGTTGTTGTGCCCTTCAAGCTTTATGAATTCTATTTTATCAGATCCCTTCCACGCCAAACAATAGTTTATCCACAAAAATGTGCTTTTAATTAGCTATGCCATCCATGGATAAATATGTAATATTAAATCTAGCATGTATTCNANNAAANNGNTNAANNANNAANGGNNNNAANG

>PvRGL135

TAGAATGCCTAATTGGCCAAAATTGTATGAGAAAGTGATAACATTTCATATAGCTAGAATGGAGGCCATTCCAAATACAAAAACCCACTGCAATATCTAAGTTGTCCCTTTAGTTCTTGAAGGGGACAACAAGTGTTTCAGATAATTGACAGATCTATTCACATTTGCTACGAAGTAATCTAGAATGCCACACAAAGGAAAAGGGACACACGAAATGGTGCAGTGGAAATAGCAATGGCATCCATAATTGGAGAGGATTACTTTTGTCATCAGTGATCTGAATGAGACTCATAAAACTCGCTTGGACAAGGAGACATGACCTCTTGTTCCAGCAACTGTAACACCTGGTTCATTGAAGGACGATCATCTGCATTTCCATCTGTGCATCTTTCTGCTAGCTCAAGAATTACTTCAAGGGTTCCTGCATCTGCATCGCTGCATCTTTTGTCCACCACATCTTCCAATCTGTTATCTCTCAGTAATGTGTTCACCCAACCAACAACATTTAAACCTCTTTTTACAAAGGAAGGATCTGTTGGCCTCTTTCCAGTTACAAGTTCGAGTAATAGAACTCCAAAGCTATACACATCTGACTTCTCAGTTGCTCTTCCACTTTGTAGATACTCTGGTGCCAAATACCCAAACGTGCCAGCTACCACTGTGGTAACATGGGCCTCTTCATCGACCAAAAGCTTTCCGAGGCCAAAATCAGATATATGTGGTTCCATGTTTTCATCAAGGAGTATGTTGCTAGATTTTATGTCACGGTGAACAATTTCTGGGCTGCATTCATGGTGCAAGTAAGCCAAACCCCGGGCAGAACCAAGAGCTATCTTTAGGCGATCACTCCAGTTCAGTAGTCGTCTTTGTTGAGTATTTTCATGCAAGAGATCATCTAAGCTGCCTAAAGCCAAATAGTCATAGATAAGGAGTCTTGAAGAAGGAAGCCTGCAGTAACCACGCATATTTACTAGATTTATGTGCTTGATGCTCCCCAAGATCTCTAGTTCCCTTTCAAACACTTGATCAGACCCTTCACAACTTCGATCAATCCGTTTAACAGCAAATGTACCACAATCATTCATCACCATTCGGTAAACAGTACCATACCCTCCTGATCCTATTATATGCTCTTCATCTAGAGACTCCAGCTTCTCTATGATCTCAGATGATGTGTATGGCAGGTCACCATGGAAAGTAATAAGTTTTGTGCTTGCTCCTGGATCAACTTGTTTCTTGACTTCCGTGTATCTCTTCGCAGCTCGTTCCTTCTTTGATAATAAACGAGTCCAGAGGAATGAAAGGATCATGACAAGTGCGAGACCCATTGTTGCCATTGCACCAATTAGCACCCCCTTCATGTAATGTGAAGACCTTTTGGCTGGGACTGCAGCTTCATCACTTTCAGCATGGGGCAGTACCACAGGAAAACCAAGTGATGTCCGACATGGCTTTTGGACTTGCCGCCCACAAAGATCTAAATTTCCATTAAACGAGCTCTTGTCAAAGGTGCTTAGTACTCCAATGTCAGGAATTTCACCAGAAAAGAAATTGGTAGACAAGTTCAGA

>PvRGL136

GGATACGCAGCACCAGAATATGTGATGACAGGTCATTTAACATCAATGAGTGATGTGTATAGTTTTGGGGTAGTGCTGTTGGAGCTGCTAACAGGGAGGAGGTCAGTGGATAAGAATCGTCCTCCAAGGGAACAGAACCTAGTGGAGTGGGCAAGGCCAATGTTGAATGATTCAAGGAAACTCAGTAGGATAATGGACCCTAGACTTGAAGGGCAATATTCTGAGATGGGGACCAAAAAA

>PvRGL137

GGGAAAGAGATCATCATACCAGTTTCAATTTGAACATAAACTCTCCACCCTTTCTAGTGAGTTCTGACCTTACAACTAAAAATGTACAAAATCATGAATGCTACATCCATTTTACACACATCATCTGCTTCACCAATTGTGTTGCCTAAAATTGGTCTAAAATGTATAATTGTAAATACAAAACAATTGATCTAGTATCATCCTATCTTCCTGACATTAGATTCACAAGATTTTGATTTTCATAGAAAGAACCAACATCCCACTCGTCTGTAGTTGAAGAAAGTGTCATCAACGCTACCACAATAGATCTCATACTTGGACGGAGTTGCGGATTGTCTTGTGTGCATGCTTTGGCTAGTTGTGCCATCTTGCGGACAGAATCAATTGGGTAGTTATCCCCAAGCCTTGGATCAACTAGTTTGCAGAGATCTTCAGTGGGATCCGGCTGACCAAAAACTCCATCAAACAGAGCTACTAGGCCCTTTGAGTCGACAGCAGAATCGTTTGTCTTGACAATTGCTTCCTTAGCCGAAATAAGTTCATAAAGAACAACTCCAAAAGCATACACGTCTACTTTGGGAGAAACATCCCCATATTGAGCATACTCTGGTGGCATGTATCCAAATGTTCCAACTAGGCGACCTGTGGGAAGTGATGAACTTCCAACTTCTGTAAGTTTGGTTAATCCAAAATCAGCAACCTTGCCCCGGAAGTTTTTGTCTATTAATATATTTGCTGACTTTATGTCACGATGAATATATAAAGGCACTGTATGCTCGTGAATATACTCAAGACCTCTTGCAGAATCCAAAGCAATCTGCACACGAGTAGCCCATGGCAGTGGTTCTCTACTACCTGAACCACGCAGATGTTGACTTAGGTTTCCATTTTCAATGTATTCATAGACAAGGAAAAGAGAGCCCTCCATGCTATACCCAATCAACCGCACCAAGTTTAAATGATGAACACGTGTCAAGACATTCAATTCAGCGAGAAATTCTTTTGTTGCTTGCATATCCATCTTCTTGATTGCAGCTTTCTCTCCCCTCAGCTCCGCATAATAGACTGACCCAAAACCTCCTTGACCAATTTTATTAGCCAAACTAAAGTTATCTGTGGCAGTTGCCAGTTCCTCATATGTGAATTCCACCGAGTTGTCCACTTTTATGCCTGTGATGGCGGCAGGACCACCAGGTCCTAAAGTTTCATTCGCACTACTACGAGAAGCTTCATCCTTCCCATGTTGAGCAAAGAGTGCACTAGAATCTCGTGGAAGTTCATCCTTCTGTATCTTCTTCCGGAATCTTCTAAAATATATAAAAACTCCTAATAGAAGAAGAACTGCCACAACTCCAACAACTATTCCAGCAATAGCCCCAACAGCAAGACCTCCTGAACTGGACGCCAAAAACACATAGCTACCATTTTGATCTTTTCCAATATCTCTGGGAAAACTTGGTTGGTCACTTTTGTAGAATCTCCTCCAACCACTCACAAAGTTCGAAACCGAAACCGAAACCTCGCACTCCGAACACATGTCGTTAATTTGCTCATGTTCAAGTGAAAAATAGAATAAACGAAAACCTACCAC

>PvRGL138

TCAGTTGAAAAATTCTGGGTCTAACAGTGAGTCAAACCATGCTGTGAAGCCTTCCCCCCCTGCAGTGTATTCAAGTTTCCAACTTGCTTCTATGTTTTCCAAGGTAGTTGGCTTGAGTGTCTTGTTTTCATCCTTGGTGGTGGTTGATGTGTGAGATAAATTTGAAGGACAAATGTTGTTGGAAGGTTTAATGGGAGCTTAGGAGACTGGAGCCGGGGCATAGTCATGTTTTGTAATTGGTTTGGTGCTTTGAATTGTTTTGGAAGGAGGGATGATCCTGCAAACCAGCCACATGAACAAGTGGTTGCAACTAAAAAGTTTAGCTTTAATTCATTGAGATCAGCAACAAAAGATTTTCATCCATCAAGCAAAATTGGTGGAGGAGGTTATGGAGTTGTCCACAAGGGAGTTTTAAGGGATGGTACTCAGGTTGCTATCAAGTCTCTTTCTGTTGAGTCTAAACAAGGAACTTATGAATTTATGACAGAAATTGATATAATATCAAATATACGACATCCAAATCTTGTGGAACTGATTGGCTGCTGTATTGAGGATAGCCATCGAATACTTGTTTATGAGTTTATGGAGAACAATAGCCTTGCAAGCTCTTTGCTTGGTTCAAAAAGTAAATATGTTTCTCTGGATTGGCCAAAGAGGGCTGCTATTTGTCGTGGCACAGCTTCTGGTCTTTGTTTTCTTCACGAAGAGACTCAACCAAACATTGTTCACAGGGACATCAAGGCCAGTAACATATTGTTGGATGGGAACTTTAATCCGAAAATTGGGGATTTTGGTCTTGCAAAACTTTTTCCTGACAATGTCACCCATGTTAGTACTCGAGTTGCAGGAACTGTGGGATATCTTGCACCGGAATATGCACTTCTGGGGCAACTTACAAAGAAGGCAGACGTTTATAGTTTTGGGATTCTAATGCTTGAAATAATAAGTGGGAAAAGTAGTAGCAAAGCTGCATTTGAAGAGGACTGTTTGGTTTTGGTGGAATGGGCGTGGAAGCTGAAGGAAGAAAATAGGCTTCTAGACCTTGTTGATTCAGAACTAAGTGAGTATGATGAGAGTGAGGTGTATCAGTTCCTTGTGGTGGCTCTATTTTGCACCCAATCAGCTGCTCAGCATAGACCAAGCATGAAACAAGTATTGAAAATGCTTTCCAAGGAAGTTCATCTTAATGAGAAGGCATTGACTGAGCCTGGAATATACAGATGGCACAGCACTGGAAAAAGGGGTGGTTCTTTGAATGAGACATCATCTCAGGTAATCAAGTACAGAAAAACTGAAAATCCACATGAAGCAGCTTCAACCCACTTTAGTGGTACAGATATTATGACAGATATGCTCCCAAGATGATGAAATGGTTGCATTGCATGTGCACCTGGTGTTACGTACATAATAGTGGAATGTGATAATATTTATCATTGTAGTTTTTAGGAATAAGATCAATGTGTTTCCAAAGGGAAATATAAGATCATGGAAGGAAAAGCCTTCTTTCATCAACATGTATATGTGTGTGTGCTTTATGCATATTTTCACTTTGCTATTTATTTCTTTCTGATGTTAATAGTGTGATAATAGTCNTNNTTNNN

>PvRGL139

GGTGTGCCAAAGCTGAAGAGATCAGAGCTTGAAGCAGCATGTGAAGATTTTAGTAATGTAATTGGCACTTCATCCCTTGGTACTGTGTACAAAGGGACTTTGTCCAGTGGTGTTGAAATAGCTGTGGCCTCTGTTGCAGTGACAGAATCAAAAGACTGGTCAAAGACTTTAGAAGCCCAATTTAGGAAGAAGATAGATACATTATCAAAAGTGAACCACAAGAATTTTGTAAATCTTCTTGGACATTGTGAAGAAGACGAGCCTTTCACCAGAATGGTGGTTTTTGAATACGCCCCAAATGGAACTCTCTTTGAGCATTTACACATAAAAGAAGCTGAGCACTTGGATTGGGAAACAAGACTTAGAGTTGCCATGGGCATGGCTTACTGCCTACAACATGTGCACCAGTTGGAGCCTCCTTTGGTTCTTGGCAACCTGAATTCTTCAGCTGTTCAACTCACTGATGATTGTGCTGCCAAAGTCTCTGATTTTAGTTTCTTAACTGAAATAGCTTCAGCTGTGATAAAATCTTCTGCTAGACAGCACCCTGACATGACCCCAGCAAGTAACATTTACAGTTTTGGTGTTATATTATTTGAAATGGTGACAGGCAGGCTTCCTTATTCTGTGGACAATGATGGCTCACTTGATGACTGGGCTTCACAATATTTACATGGGGATCAGCCCCTCAAAGAAATGGTGGATCCAACTCTAGCGTCCTTCCAAGAAGAACAGCTGCAACAAGTTGATACTTTAATCAAATCTTGTGTCCATCCTGATCAAAAGCAAAGACCAACTGTGAAAGAGGTTTGTGCGAGATTAAGAGAGATAACAAAAATAACACCCGAAGCTGCTGTTCCAAAACTTTCTCCACTTTGGTGGGCAGAGCTTGAGATTGCTTCAGTAAATGGAAACTGAAATCAGTTAATGAGGTTGTAAGTATGAAGTTCTTACCATCCTCATCCTTTTTCAATCGGTTAAGGCACAAAAATGTATTCACCTGTACATTAATACATCCTGTGTTTCATCGTACAAGAGTGGCAAATCACATGTACATAAGGGAGAGAAAAAAATGTGATCTTTTCTTTGGGTGCTTTGTTTGGTTATGGTTTATGGAATCAAACTAACAAAAAAAGCTTCAAATTTCAGTTAATTTCATTTCCGCAGTCTGTTTATTTGGTGA

>PvRGL140

AACAAGGAGAGAGAGAATTTCAAGCTGAGGTTGAGACAATTAGCCGAGTGCATCATAAGCATCTTGTTGAATTGGTTGGCTACTGTGTTATTGGGGCAGAGAGAATGCTTGTTTATGAATTTGTTCCAAATAACACATTGGAATTTCACTTGCATGGGGAGGGGAATATCTTCTTAGGATGGACAACGAGAATTAAAATTGCCCTCGGATCTGCAAAAGGGCTGGCATATCTTCATGAAGATTGTAATCCAGCAATTATTCACCGTGATATTAAAGCATCTAATATTCTTCTTGATTTTAAGTTTGAACCCAAGGTTTCTGACTTTGGCCTGGCAAAAATCTTACCAAACACCGATAGTCGCATTAGTCACCTCACCACCCGAGTGATGGGAACCTTTGGATATCTGGCTCCGGAGTATGCATCAAGTGGTAAATTGACAGATAAATCAGACGTATATTCCTACGGCATCATGCTTTTAGAACTTATAACCGGGCGTCCACCAATCACTGCAGCAGGATCAGGGAATGAGAGCTTGGTTGACTGGGCTAGGCCATTGCTTTCTCGAGCACTACAAGATGATAACTTCGACCTTGTTGATCCAAGATTGCAGAAAAATTACGATGCTGATGGGATGGTTAGAATGATTACATGTGCAGCTGCCTGTGTGCGCCAGTCATCCACCCTTCGACCTCGTATGAGCCAAATTGTTGGAGTCTTAGAAGGAGTGGTTTCTCTTTCTGATCTTGTTGGAGATGTTACACCGGGACATACCACAGTACACAATTGGTCAGATTATTTAGATTATGGTGCCAACCAAGACCAGAAGGACTTCAGAAGTTTCGATTTGGCATTATCATCTCAGAAATACAGCTCCAGTGGGTACATTGAAACAACTAGTGCTTGTGGCCTTTACTCATCAGGCTCAGGTAGTGAGGCTCACCAATCATTTCGAGACATAATATAAAAGAGGTTTAAGATGGTGAATTGTATTGTGAACTTGGAATTCAGAACTATGGTAATTATGTTCTACTCAAGATGGTAAAGCTCGTTCAAATTCT

>PvRGL141

CCAAACAAATCAAAATCACATGACATTAATTAATACACCTTAGAGGTGGTAAACTTTACATTTTGATAATTATACACTTAAAATCAAATATTATTACAATTTTTTCCCATCAGGATAACGTATAAAATGAACTTATGATATATTATGCAGACAAAGATCACGTTATTATTAACACAAATTTACTTGGAATCAATTTGTAAAATCACATTGATTCAAAAACGATTCCCCGTTCATATGTCCAAACATACACAAAAAAGTAGTTCTTTGAACATGGCTCAACGTTAGTGACTTGGAAGGTGAACATATTTTGTTGTTGTACACTCATATGTAGACATCATTGATCTGTCAACAACAAATTATGCCTTCAACACAGTCTCTTTCCTCATATGTTCCTTCAACAAGGTTATGCCTCATTCTCCAAACGGCTCTGCATTGATGCAAAAGAATGCATCTGATCTGAACTTTGATATGTACATAAGCTGTACATTTGAAGTGGTTGATAAAAAACCATTATCTTCATATCTCCTCACTAGCACCAGTGTGTCTCTTCTCAAAGGAAAACTCTGATGAACTAACTCTGATTTCAATCTGAGAGCTGGTAGGAAATTCTGGTGCTGAGCTTGAGCCTGGCTCCATGACTTGCATTTGAGTTTCTTCAGCTGCTGCATGGTTGCTTGTGGAAGGTGAGCCTTCAACATCCCATTGTTCATTTTCATAGTTTGATGTCCTTGGAGATGTTTGTGCACTGTCACTTCTACCAGCAAGGTGTTTTTGCAACAGATGCATTGGAGACATACCATATGTTGGGGTTGATGGCTTGCTTGAGAATGGTGTGTTTTGCTTGCTGTTTTTGACTTGCTTTTTGGCAGTGCTGTGCCAGTTCTTCAGTGCTGATGCCACTCTCTGATTGAAGATGGTAGGCTTCATGGTTGAACCCATCTGTGTGACTAGAGCATATAGAGGTAATGTCACATAGCTGCATAGAACTTGTATGACAACCCCCATTGTAAGTCTTATGGCAACATCTGCAGTTGTTTCATGGAAGCAAGAGTCTAGGGAGAATTCATAAGTACTCCAAGAGAAAAATGCCAGTTGAAAAGCATTCTGAAAGAGCACAAGATGAATGAGAAAGAGGAGGAGACGTGGGCGATTGAACCAGAACAGGTCATCTCCTGGCTCAA

>PvRGL142

GATTATAGTTGGTGCTGTAGTTGCTGTTGTGGTCTTTGTTGTACTAGCATCCCTTGCAGGGATGTATGCCATTCGCCAGAAAAGAAGAGCAGAAAGATCTGCGGGGCTGAACCCTTTTGCCAATTGGGAACAGAATAAGAATAGTGGCACTGCTCCTCAGTTGAAAGGAGGACGATGGTTTGCTTTTGATGAACTAAGGAAATACACCAACAACTTCTCAGAAGCCAATACTATTGGATCTGGAGGCTATGGACAGGTTTACCAAGGAACTCTTCCCTCTGGAGAGTTGGTTGCTATCAAACGGGCTGCAAAAGAATCTATGCAGGGTGCTGTTGAGTTTAAAACCGAGATAGAACTTCTATCAAGGGTCCATCATAAGAATCTCGTTAGTCTTCTGGGTTTCTGTTTTGAGAAGGGTGAACAAATGCTGGTGTATGAGTACATTCCAAATGGAACTTTAATGGATAGTCTCTCAGGAAAGTCTGGGATCTGGATGGATTGGATAAGGAGGCTAAAAGTAACATTAGGTGCAGCCAGGGGTCTGGCCTATCTTCATGAACTTGCTAACCCACCAATCATACACAGGGACATCAAATCTAGCAATATCTTACTTGATGATCACCTCATTGCAAAAGTTGCTGATTTTGGTCTCTCCAAACTTCTTGTTGATAGTGAAAGGGGCCATGTCACTACTCAAGTCAAAGGGACAATGGGATACTTGGATCCTGAATATTATATGACCCAGCAGTTAACTGAAAAGAGTGATGTATATAGCTTTGGAGTGCTAATGCTGGAACTAGCAACAGCAAGAAGGCCAATAGAGCAAGGAAAGTATATTGTAAGAGAAGTGATGAGGGTAATGGACACATCAAAAGATTTATACAACCTTCATTCCATTATAGATCCAACTATATTGAAAACAAGACCAAAAGGTTTAGAAAAGTTTGTGATGTTGGGAATGAGATGTGTCAAAGAATATGCAGCTGAGAGGCCTACAATGGCTGAGGTTGCAAAAGAGATTGAGAGCATAATCGAGCTTGTTGGATTGAACCCCAATGCTGAATCAGCTACAACATCAGAAACTTATGAGGAAGCAGGTAAAGAAAATGCCCAACATCCTTACAAGGAAGAAGATTTTAGCTATAGTGGAATATTTCCATCAACAAGGGTAGAACCCCAATAGGCTTCGGTACAATTTTCTTTTGTAATTTTTTCCTCTCTTCAGATCCAATGCCTGAGATCTGCTGTGTCAATTTTGGACTCGAATCCTTATACAGGCGGGTGCTTTACAAAAACTTGCTCTTTGTAGAAAATGAAGGTTCAAGTTGAATTTTTGAATTGATTATTGGAAGGTTGTATTGATATCTTTGTTCTTGTCATGCAAAATATTTGTGAATTGTGAATTGCAGTACTATTTGAATGAATGTAAGTATTGAAGCGTTGAACTTTCCA

>PvRGL143

TTCTATGCACATAAACTGCCAAGTTCACATCATCTGGTGCCCTGTTAAAATCTATTGCCTTCTGAGCTGTCAAAAGCTCAAGAAGCACCACTCCAAAACTGTAAACATCACTCTTATCAGTCAATTGGTAGTTCCTATAATACTCAGGATCAAGGTAACCAAGAGTCCCCTGAGCACAAGTGGAGATATGACTCATATCTGTCTGAGCCAACCTAGACAACCCAAAATCTGAAACCTTGGCATACATATTGATATCTAGGAGAATATTGCTGGACTTGACATCTCTGTGGTAGATAGGAGGCACAGCCATGAAGTGAAGGTAAGCAAGACCCTCAGCAGNGTGACGTGCAATTTGAAGGCGATGTGTCCAACTAAGCAAACAACGGCCCTTAGGAATTTGACCTTGCAAGTGATCAAGAAGGGTACCATTTTCTATGAACTCATAAACCAAAATAGGCTGCTCCAGTTCCACGCAGCAACCAAGTAGACCAACAAGGTTTCGGTGGTTAACTTGGCATAATATGCGAACCTCATTGGGCACTTGATCAATGCCTTTGGCGTTGCCAAGCTTAGCACACTTGACAGCCACAGGAGTACCATCTTGAAGAAAGCCCTTGTAAACTTCACCATAGCCTCCAACACCAAGAAGTCGGTCATTGGAGAAGTCGTTTGTGGCTTTCTTTATCTCTTTGCCACTGAAAAGTTTAGCCGCTCTTCCCC

>PvRGL144

GAGGACTTTGAGCCGCAGATTTGTGATTTCGGGCTAGCAAAATGGCTACCAGAGAACTGGAGCCATCACACCGTCTCAAAAATTGAAGGGACTTTTGGTTACCTTGCTCCAGAATACTTGCTTCATGGGATAGTGGATGAGAAAACAGATATTTTTGCCTTTGGTGTAGTGCTATTAGAATTAGTCACTGGGCGTAGAGCAATTGATTATTCACAACAAAGCCTTGTTTTGTGGGCAGAACCTTTTCTGAAAAAGAATAATATTAGGGAGCTGATTGATCCTTCACTTGCTGGCGATTTTGACTGTCGGCAGATTAAAATTATGCTCTTGACAGCTTCTTTATGCATTCAACAATCCTCCATTCGTCGCCCCTCTATGAACCAGGTTGTACATCTTCTGAATGGTAACTGCTCTAGGTTCACGAAGAAATCTCAACTTCCATTCTTTCGTAAAGACTTTCAGGAAGAGTTCATTGATTCTGATTAACTGAAAGGAACGTAAATCATGAGTGACAGCAACACTAAATTCTGATTAAATGATGACAGTGATGTATGTAATTTGGCTTTTCTCTT

>PvRGL145

GATTCTTATTATCTTGAATGGCTCGTCTTCCTGTCACAATTTCAAGTAGTAACATCCCAAAACTGTATATGTCACTTTTCTCGGTGAGCTCTTGAGTAACAACATATTCAGGATCCATATAACCCGGAGTTCCCCGAATTTCAGTGTTTACAGGTTCAAAGCATACTGATCCATCTTTTGAAGCTTGTGCAAGGCCAAAATCAGCTATCTTGGCAACAAAGTTCTCATCCAGTAAAGTGTTGCTAGATTTAATATCTCTGTGACACAAGGGAGGATCGCAATAGAAATGGAGGTACTCCAGTGCATTGGCCACATCAATCGCAACTTGGATTCTTG

>PvRGL146

GAGTGAAATACGGAATGTCTACTAATAAAATTTCATAACTGGAACAATTTATGGCACACCACCAATTTATTTCCAAATATTTCTAGAAAAGCTGCAACACATTACAAGCAGCATAAACAGAGGAAAGAAGAAGTGAAGTTATTATAGAAAAGAAAAGCATGTTCAATGATTTTGAAGTTGTCCAAGTACAATGGTGTTGAGTGTCTCCCTCATTAAAGGTGACTTAGGCATCACAAACTTGTTGTAGACATCTTCCATGGTTGGACGAGAAAATGGTCTTTCATTCAAACAAGCAAATGCCACTTTTGCAATCAAGACCACCTCCAGCACAACTGGCATCACTGGAAGAGGAAGTCGTTGGTCTATCACATCCTTCAATAGCAAATTAGATGTCTCTGGTGTTGAAGAGGGTGAATGCATCCAAGAAATGAGATCTCGTGGATGATTTCCCATTATTATCTCCATACAAAGCACCCCAAAACTGAACACATCGCATTTCTCATTCACTTCCATGGTATAAGCAAGCTCTGGTGCAGCATATCCATAAGTGCCAGCAAACGAAGTTAAATTTCGTGAATTAGGATCTAGAATCTTTGCTGTTCCAAAGTCAGATATTCGAGCTTCATATTCCAAACTTATAAGAACATTCTTGCTTGATATGTCACGATGAACAATAGGAGGAGAGCAACCATGATGCATGTAATACAAAGCATTTGCCAAACCTTTAATAACGTTAACCCTCTTATTCCAATCAAACATTTCTGCATGTGTGTCGTTGTTTAGTACTTTATCCAAGCTGCCACATTCTAGGAACTCATAAACCAATAAACAATGGCGTGGATGTTCACAAAACCCATGCAACTTCACAATGTTACGATGTTTGATTTTTGTCAAGGCTCGAACTTCAGTTGTAAAAGCCTTAAAATCAAGCTTTTCCCTGTCAACTTCAACTTGAAGTTTTTTCACAGCAACAATGTGGCCAGAGGGTAAGATGGCTTTGTAAACACATCCAAACCCTCCTCTTCCGATGAGATATTTGTCATCGAAATCATTTGTTGCTTCGATGATATTTTCATACATTATTTTGCCATCATAATGCAATACA

>PvRGL147

GGGTTATCCCTATGCCTCTCCCCCTCCACCGGCTTGCCACCCGCCTCCGCCACCTCCACCTCCTCCTCCGATGTGTGAGCCACGCTTGGAGAGAGGTCGCAAAACTCTGGTGGGCTTCACCGGTGCTGTGGACAAAAACAAATACATCGGTGACTGGAGGGGTAGCGACCCTTGCAAATTCAAGGGGATACGATGTGCAAAATATCCAGACTCAGAAAAGCAGTTAGCAATAGCGGGAATAGACTTGAACGGAGCTGGGTTGTCGGGGAAAAACCGCGCCCCTCTGCTCCTCTCCGGCATCTTGGACAGGATACCGGAGCTGACCTTCTTCCACGTGAACTCCAACAACTTCACCGGTGCCATCCCACCTCAGATAACACAGTACGAATACTTCTTCGAGTTGGACCTCAGTAACAACAGGCTCAAGGGCGAGTTTCCCAAGGAAGTTCTCCAGTCCTCGCAGCTCGTTTTCCTTGACCTCAGGTTCAACTCTCTCTACGGTTCCATTCCTTCCCAGCTATTTCACCTCAAATATCTCGACGTCATTTTCATCAACAACAATCAATTCACCGGCAATCTCCCCGATGACTTTGGCTCCACTCCGGCAAGGTACCTCACGTTTGCCAACAACCAACTCACCGGTCCAATCCCCGCCTCCGTCGGAGCTGCCAAAAACCTGGTCGAAGTCCTCTTCTTGGGCAACCACTTCCAGGGCTGCTTGCCCTACCAGATTGGCCTCCTAAACAAGGCCACCGTCTTTGACGTCAGCAAAAACTGGTTGACGGGTCCCATCCCCCGGTCCTTCGGGTGCCTGGAGAGCATCCGGTACCTGAACCTGGAGCAGAACCAGATGTACGGTGAAGTTCCGGAGACGGTGTGTGAGCTCCCGGGGCTCCGCAACAGAGGCAACTTGTCCCTATCGGACAACTACTTCACACAGGTTGGCCCTGCATGCAGGAGGCTGATCGAAGCAAATGTGCTGGACGTGAGGAACAATTGCATCCTTGGTCTTCCCAATCAAAGGACTCATGAGCAATGCTCCCAGTTCTTCTCCAACCTCAAGCCATGCCCGAATCCCAAGTCTCTGAATTACGTGCCATGCAAAAAGTATGATCCGAGCGGGCACATCCACAGCGCCGCCACTCCTCCTCCTCGTCCGGTGTCTTACAACTCTCTGGACCCCACTCTTCATCGCTAGCTTATCCTTAACCTCTCCGTCCCATCGCACGCACCTACTGCAACTGCATGCTTTAATTATTGTTATTGTTATTACCTACGTCTCTCTTCATTAACCAATAATAAGCAGTTCCTTTGTTTCTACTGC

>PvRGL148

GAGGATGTATACTTATGACAGATTCATGGGAAGCAATTTGAAACTGAAAGATTCAGAGAGTCCTAGCTCATCAATTGATTCCATCACAAACAGGGTGGATCAGATATTGGATGATGTAGACGTTGGTGAATGTGAGATTCCATGGGAGGATCTGTTTCTTGGTGAAAGAATTGGTATAGGTTCATACGGTGAGGTATACCAAGCCGACATGAATGGCACAGAGGTTGCTGTAAAGAAATTTTTGGATCAGGATTTTTCAGGCGCTGCATTGTCTGAATTCAAAAGAGAAGTACGGATAATGCGTAGGTTACGGCATCCAAATATTGTTCTGTTTATGGGTGCTGTTACTCGTCCTCCTAACCTCTCAATCATTTCAGAGTATCTACCGAGAGGAAGCTTGTACCGATTACTTCATCGCCCTTATTGTCAGATTGATGAGAAACGAAGAATAAAAATGGCTTTGGACGTGGCTAGGGGCATGAATTGCTTACACACCAGCACACCTATAATTGTTCACCGAGATTTAAAGTCTCCAAATCTTTTAGTTGATAAGAACTGGAATGTTAAGGTATGCGATTTTGGGTTGTCAAGGTTGAAGCACAACACTTTTTTGTCATCCAAGTCAACTGCTGGAACGCCGGAGTGGATGGCTCCTGAAGTTCTCCGCAATGAACCCTCAAATGAAAAATGCGATGTTTATAGTTTTGGAGTCATCCTATGGGAGCTTGCCACTTTAAGGTTGCCGTGGACTGGAATGAATCCTATGCAAGTTGTTGGTGCTGTTGGTTTCCAAAATCGTAGACTTGAAATTCCTAAGGAAGTCGATCCTTTAGTTGGAAGAATAATCTGGGAGTGTTGGCAACAGGATCCAAATTTGCGGCCCTCGTTTGCACAACTCACAGTGGCTCTTAAGCCTCTGCAGCGTCTGGTTATCCCATCTCATCAAGACCAGGTAGCTCCATATGTGCCGCAGGAGATTTCTGTAAATTCAACCCCATGAAAAGTCTCGCTTCAAGTGTGAATAAGTAGGCATTCTGCATAGTAATGTGCGTATAAAGGTAACAAGAATACAAAATAAAATGGAAAAAAAATATGGAAAGGCAAATGTTTGTGTTGAGGGAAATATCAACTGCAACTGCACTGCAGCCAAACAACTGCAGAGAGCCATTTTGTATTTTGTATTGTTATGATAGCTTGTCCGAGGAACTAAAACTCGGTAATCTGTAAATTACTGTACTTCACAAGCAGGAAATATCTATACTTTTNCAAAA

>PvRGL149

GTAAGTAGTTTCTGCTTCATTTATTTTGCTTAGATGGTTTATTTAAGTTACTTACACTTCCTCAATCCAAAACATAACAACTTGTTTACCAAACTAGTTAATACAAAAGGGTGAATTGATCTTAAAAACCCCAAACTTGTCACTGCAAATGAACATACATACCTCCAAATCCTACAGTTCACAAGGAATGTAGATAACACAGGTAACTGATTAGTTTAGGGAATATTGAATACTAACTTGTGAGGGACATGGTGGCACCTCCACTTCAACAACACCTTCAAGCATCTGTGTCACATTTCTCATGGTTGGTCTAAAACCAGGATCCTCTTGCACACACCAAAGGGCAATCATCACCAACTTCTCCACTGTCTTCATGTCATCCAATGCCTCCTTATCACCCTCAACCAAGGTATGAAGAGAACCTTCAGAGTAGCAGTCATAAGCCCATTCACTTAGGATTGCTCTGGATTCATCTTCTTCATCAAATTCTACACTTTTTCTGCATGAAACTATCTCAAGCAACACCATCCCATAACTATAGACATCCACTTTAGCCGTGATTGGCATGTTTTTGAACCATTCAAGTGCAACATACCCTTTTGTTCCCCTTATTGCAGTGTTGGTTCTGCTCTGATTCATGTTCAAAAGCTTGGCCAATCCAAAGTCTGAAATTCTTGCAACATAGTAATCATCAAGGAGTATGTTCTGAGGCTTTATGTCACAGTGGATGATCTGTGTGATGCACTCTTCATGCAAATATAGAAGTCCTCTAGCAACCCCAGATGCAATTTGTAGCCTCAGTTTCCAACTTGGTTTCTCGACATTAAAAAGAAGACTTGCTAAAGTGCCATTGCCCATGTACTCATAAACAAGTAACCTTTCTGAGTCAGTCTCACAAAATCCAAGTAAACGAACCAAGTTCTTGTGGTGTGTGAGTCCAATGATATTCAGTTCATTCTTGAATTCCTTCTGAACCTCTTCCAAGAGAAAAGTGTTCAATCTTTTCACCGCCACACGAGTAACAGAACCTATGTTGACGACGCCTTCATATACAACNCCAAAAGCTCCCTTCCCTAGCACTTTGCCAAAGCCATCAGTGGCCTCTTCAAGTTCCTCATAAGTGAAGCAACGCAAGTTGGTTCCCACAAGTAGTGCCACTTTTGCCATGTCTTCTAAGCCTCTTCTTGTACTTGAAAACATAGCAAGTGCTCATGCAGATTGCACAAACCAATATGAGATTGAGAAAAGCAGAAGTACCAAAAAGCACTGACAGAACCAAAATCAAAGTATTCCTATTG

>PvRGL150

GCAAGATTCGGCTTTGAAATATAAAAGATTGAAAGAAACTGGGAACTTGGGCCTGAATGAAGAGGTGACTTTGAGAAGGTTTTCATACAGCGAACTCAAAAGAGCCACAAACAATTTCAAGATAGAACTGGGAAAAGGGTCTTTCGGAGCAGTTTACAAGGGGGCGTTGGAGAAAGGTAAGAGGTTGATTGCTGTGAAGAGACTGGAGAAGTTAGTGGAAGAAGGAGAAAGCGAGTTCCAAGCAGAAATGAGAGCCATTGGGAAAACCCGTCACAGGAATCTAGTTCGTTTGTTGGGTTTCTGTGCGGAGGGTTCTAAAAGGCTTCTGGTGTATGAGTACATGTCCAATGGTTCCCTTGGAAACCTTATCTTCGTGGGTAAAAGTCAGAGACGTCCAGAGTGGGATGAGAGAGTAAGAATAGCGGTGGAGATTGCGAGAGGGATAATGTATCTCCATGAAGAGTGTGAGGCACCAATCATTCATTGTGACATAAAGCCTGAGAACATCTTGGTGGATGAGTTCTGGACTGCCAAAATATCCGATTTTGGGCTGGCCAAACTTCTCATGCCCGATCAAACCAGAACCATGACAAGGGCTAGAGGGACAAGAGGGTACGTGGCCCCTGAATGGAACAAGAATGTCCCCATATCTGTCAAGACTGATGTTTATAGCTACGGAATAATGCTGTTGGAAATTCTATGTTGCAGAAGAAACTTCGAAGTTCATGTCTCTGAACCTGAGGCGGTTATGCTCTCCACCTGGGCTTATAACTGTTTTGTTGCAGGAAAACTCAATAAGCTTTTTCCTTGGGAAGTCGTGGATGACAAAACTGCCGTGGAAAATATGCTTAAAGTGGCACTTTGGTGTATCCAAGATGAACCTTTTCTTCGCCCAACAATGAAGAGTGTCGTATTGATGTTAGAAGGGGTCACGGACATAGCAATTCCTCCTTGTCCCGCTTCCAATTCTGCTTGAATTTTCTCTGTCAAGGAAATGTTGCAGTGTTCATGAATCATGTGCTTTTTACTTTATTTGTTTGTCTTCTGTTTCAGTTGTTATATTTTCTCGTGCTGTTAAGGTAGAAAGACAGAAGCTGAACTAGTAATTTTCTATGTTAGTTTATGTTTAAGCAGCAGATATATAAGTTTGCTTCATGCTTCTGTCCAATAAAGAAATGTTTGACGA

>PvRGL151

GAGTACATGGAGAATGGTTCTTTAGCTCAGAACCTTTCATCGAGTTCAACTGTTCTTGATTGGACTAAGAGGTATAACATTGCTCTCGGAACTGCAAGGGGTCTTGCTTACTTGCATGAAGAATGCTTGGAGTGGATTTTGCATTGTGATATTAAGCCCCAAAATATACTTCTCGACTCTGATTACCAACCTAAGGTAGCAGATTTTGGCTTGTCTAAGCTACTTAACCGGAACAACCTCGACAATTCAACATTCTCGAGGATAAGAGGAACAAGAGGCTACATGGCACCAGAGTGGCTTTTCAACTTGCCAATCACTTCCAAGGTGGATGTCTACAGCTATGGCATTGTCGTCTTGGAGATGATAACGGGAAGGAGCCCAACAGCAGGTATCCAAATCACTGCATTAGATGCAGAATCACATAATCATGGGAGGTTAGTGACATGGGTGAGGGAGCAAAGGAGGAAAGGATCATCTTGGGTGGATCAAATTGTTGACCCTGCTTTGGGTTCAAACTATGACAGGAATGAAATGGAGATATTAGCAACGGTGGCTTTGGAATGTGTAGAGGAAGAGAAAGATGTGAGACCCAGCATGAGTCATGTTGTTGAGAGAATCCAAAGTCACGAGCACAATTCTTGATGATGGTTTTGAACAAAGAATAGCCAGACATCCCATGGTTTCACCTACCTTGTTTGTCATCCACAGTTCCAGTTCCTCTCGTTTTTTGGTAGATTCAGTCTATCAAAAGTTGTCTGTTCGTGCCTTGTGGTAAATCTTCTTTTGTTGTGAAGTTTAGTGTTGCTGTTGTATATCAGATTCTAGTGCTTGTAAACCGTATGTTGTTACAGTTGCAGAATATTTCCTTTAATTGCAACATGCACTTTTGTTCCTTTAACATTATCTGTTGTATGATTCCAACTCTATCATAAAATTAGTTACTTCGTACAT

>PvRGL152

TGCCTAATGGTTCTCTTGATAAATATATCCTTTTCCAAACAAGATAGTGTCTCTTTAACTCAGAGACAAATATATGAGATATCTATTGGAGTGGCTCGTGGAATTGCTTATTTGCATGAAGGTTGTGACATGCAAATTTTGCATTTTGATATCAAGCCACATAACATCCTTCTGGACGAAAACTTTATCCCAAAGGTTTCAGACTTTGGCTTGGCAAGGCTTTATCCCATAGACAAAAGTATTATCACTTTGACTGATGCAAGGGGAACACTTGGATACATGGCCCCTGAGTTATTTTATCATAATATGGGGAGAGTATCACACAAATCAGATGTTTATAGTTTTGGAATGCTTTTGATGGAAATGGCAAATAAAAAAAGAAATTTTAATCCACATGCAGATGATTCAATTGAACTTTTTTTTCCTTTTTGGATATACAATGAATTGAGTGGAGAAAATGAGATAGAAGGGGAAATTGTTAGCAAGCAGGAATACAATAATGAGGTAAAGAAGATGTTTTTAGTAGCATTATGTGTATACAATCGAAACCAAGTGATCGTCCTCCAATGAACAAAGTAGTAGAGATGTTGGAAGGAGAACTTGAGAACATTGAAATGCCTCCAAAACCTTTCCTATACCAGGTAAATGATCATAATATTAAATCTTACCAAACCCTTTCAAATGATTCTAGTGAATCAACAAGTTATCCTACTGGGTAAAATACTGTTCTTATTGTTTCTGAACATTCTACTTGATATTTGTAATGATTAATAAATACATTTATATCATGCATGATATTTCTTTC

>PvRGL153

GAATAACGGATTTATATGATAGTGATTTCCATTTATACGAGGAAAATCTGCTTGTACTTAAAAGAACAATCCACATAAACCAAATGGCTAAGGAAATTGAAAAGGATACACAGCATAAAAAGGAAAAAAAAAAATCTCAATAGTTAACATATTTTTCTATATGACAAGCTAGTTCTCAGAGAAGCATATCTCATCAAAACACTTCCATCCTTTGAGGAACTGGGGTTTTAATGACCTGCAGTATTTGCACCACTTCAGACATAGTTGGCCTGCTTGAAGGAATTTGGGAGGTGCATACCATTGCTAGCTTTAGAACAGGCAATACCTCATCTTCTGGGTACTCATTCATACTGTGATCCACACACTCCAACACATTACCCTGCTCAAGCAGCACCCTCACATGGTCATTCAGTATAAGCACATTGTCTTCACCATACTCCACTGGCCTCCTACCTGTCACCAGCTCAAGGATCATCACCCCAAAACCATAAACATCACATTTCTCATTCACCCTTAGGCTCTGACATGCCAATTCTGGTGCCACATATCCTAATGCACTCTGGAACCTATTGCTCATCACATGCCTGTCCAGCTTTGTCAGAAGCCGAGCTAACCCGAAATCCGAGATCTTGGGGTTGCAATTTTCGTCAAGAAGAATGTTACTTGGCTTAATGTTGTAGTGGATGATTGGTGGACGGAAAGAATGGTGCAAATGAGCAAGCCCCTTTGCTGTCCCAAGCAAAATTTTGAACCTTATAGGCCAAGAAAGAGGTGGACTTGAGGGAAGCCTCTCATGTAGTTTGGCCTGCAAGCTACCATTTGGTGCAAACTCAGTTACTAAAAGCTGTAATTGAGGAGTCCAATAGTACCCTTTCAATGCTATTAGATTTGGGTGCCTTGCTTTCCCTAGGATCCTAACCTCCTTATCAAAGTCTTCTGGATATTGGATTATGTTGGTGGATATAAGCTTCTTGATTGCTACCATTCTACCCTGTGATCCCAATGGAACCTTGTAGAGGGTTCCAAACACCCCTTCTCCAATCTCTGACGCCTTGTTGAGCAAGGACTCAGGACTGCTGATCCAATCAGGTGAGGACTGGGAATCAAACAGAATCAGCTTTCCTGTGGCTGGACTCCCTGATCTTGAAGAGCTTGAGCACATGCTTTCCAAAGCATTATCCACAAATGATAGCCTTCTCCTCACAGAAACATTAAGTAGGCTAACAGCAATCACTCCTAATACAATCACAAAGGATGCAGATATTGCTACAATAGCAGA

>PvRGL154

GCATGGAGCTGAGAAACCAGTCATTTATAGAGACTTCAAGACTTCCAATGTTTTGCTTGATTCAGAGTTCACAGCTAAATTGTCAGATTTTGGACTTGCAAAAATGGGGCCAGAGGGATCAAACACACATGTTTCAACCAGAGTCATGGGCACTTATGGATATGCAGCCCCAGAATACATCTCAACAGGGCATTTAACCACCAAAAGCGATGTCTACAGCTTTGGGGTAGTCCTGCTAGAACTGCTGACGGGAAGGAGAGCAACGGACAAAACAAGACCAAAGACTGAGCAAAACCTTGTGGATTGGTCAAAACCTTACTTGAGTAGCAGTAGGAGATTGAGGTATATTATGGATCCAAGGCTTGCAGGCCAATATTCTGTGAAGGGTG

>PvRGL155

GGATCACATTCATGATAGTTCCAAGAAGAAGAACTTAGACTGGTTAGCTCGTCTTCGTATTGCAGAAGATGCAGCTAAAGGTCTTGAATACTTACACACAGGATGCAATCCTAGCATCATTCACCGTGATATAAAGACANGGAACATTCTCCTGGACATAAATATGAAAGCAAAAGTATCAGACTTTGGACTCTCTCGATTAGCTGAAGAAGATTTAACCCATATATCAAGCATTGCACGAGGAACTGTAGGCTACTTGGATCCTGAGTACTATGCAAGTCAGCAATTGACAGAAAAGAGTGACGTATACAGTTTTGGAGTTGTTCTGCTGGAACTGATATCTGGAAAAAAACCTGTGTCAGCTGAAGATTACGGTGCTGAAATGAACATTGTTCATTGGGCTAGATCTTTAACTCGTAAAGGAGATGCAATGAGCATCATTGACCCTTCTCTGAATGGGAATGCTAAAACTGAGTCCATTTGGAGGGTGGTAGAAATTGCCATGCAATGTGTGGAACACCATGGTGCCTCAAGGCCAAAGATGCAAGAAATCATTTTGGCCATACAAGATGCTCTAAAGATAGAAAAAGGAACAGAAAATAAGCTAAAATCACCATCTTCATCATCATTTTCAGGTAGCAGCAGTTCAAAGCCACACTCTTCACGCAAAACTTTACTCACAAGCTTTCTTGAAATTGAGAGCCCTGACGTGTCAAATGGTTGCCTCCCATCAGCAAGATAAATTATATCTACCTTACTTACATTCCATTTTGTTTGTATATTTACTTCTTTTAACCAACCCTACTATACACCAGGAACAGTGTGACACTAACTAGTCACAGCATTCCCATTTTTGCTTTGCAACCAATGAAATTGTAATCTTCTTAATGATGACCATCACAATATGATTTACTGTGAATGCTATTTTCCAGAACATAAACAAAACATGGAAAACTAACTTTTTCCACATTTCTTGA

>PvRGL156

AACCCAAAAGACAAGGAAAAACCAGAATAACAGACTATTAGAGAAAATAAATGGCTGAAATGAATAGAAATAGAAATAAAAATCTTTTACCTAGCTACAACCAAAGTCCAACGCATTCACATTCATTCACATAAAAGCTAGTCCTAAGTTAAGGACACAGAAACATGGCATGCCTTAAAGGGGTCACCATCACCATGACCATGAGCCAGTGGCCAAATACTAGGAAAACTGATGTTGTGTTGATGGTTAAAGTAATTCAGGTAAGATTCAAATCAGAGACAGCTACTACAATTCCCCAGTTCTTGAACAATGGCTTCCATGCTTGGCCTCTCAAATTGAGACTCATGGGAGCAAAGTAACGCCATTCTTGCTAGTTTAGCTGCCTCATATTCAAAGAACTTGCCACGCAAGTTCGGGTCAATAAATTCTTGAAACGTGAAGGACTCTGCTGCAAGGCGCATTGCACTTGTGATCTTCTGTTTCCCTGTGATAATCTGGAAAAGAAGCACTCCAAATGCGTAAACATCACTTTTCTCTGTGAACCGGCCGGTTGTGGTGTATTCTGGAGCCAAGTATCCCTTTGCTGCACTACCCTTTAGTGCAGAGAAGACAATGTCATTGGTGAGAAGCTTGTACAGGCCTGAATCTGAAAGCATTGGGTTGTACCGCTGATCGATTAGCACTTTCTCAGCTGAGATGTTTTGGTGAACAAGGGCTGGTTTGTTTGCTTTGTGTGCATGTAAATATGCTATACCTTTAGCAATCCCTTTCACAATAGAAATTCTAGTTGACCATTCAAGAACTTCTCCATCACCTTCCTTCACATCAAGGTAGCGTGTCAGGTTTCCATTAGGAACAAAATCATAAACCAAGAAGCATTCCCCTCGTCCCCTTGAGCAACAAAATCCTCTCAACCTCACTAAATGTTCATTTCTCAGAGAGGTCAAAATGTTCAATCCCTTCAAAAACTCAGCTTCATCTGATTTGCAACTAGTTTTACTGATGCTCTTGACAGCAACAGCAGACCCATCTCTTAAAACCCCTTTATATGTTGCACTAAAGTTGCTCTTCCCCAGCAGATTCAACTCTGAGAAATACTGAGTAGCTGACTCCACTTCTTCCAAGTTGAACCTGAAACTCTGGAACAAATCTTGCTTATCTCCGTTGAAATTCCTGCTATCAGCCAAAGGGTCCCACCCATTAGAATACTCAAGGCTAACCAAAGGAGATCCATTTTTCCTGTATATACTCTTTGCTTGCTCTGTACTCAGTCGGCCTTCAGAGATATCAAAAGCACTTCCAAGCTTTTGTTTCCGGCGACGATACATAGTGAAGGTCAAAATACCAATTGCTGATATTGCAATTGTTGCTAGAACTACGCCAACTGTAATAGATGTGGCTTGTTTGGATTTTGATGGATTTTGGCAATGAGATGTATTGCAAGGCGACTTGACATTAGCAGTTTCTGGGATATCTCTAGTGATACTACTG

>PvRGL157

ATCTTGCCTCAACCAATGTCTCAGACATTTCGTTGAGGGAATACGCATCACAATTTCTTGAGGAAGAATCTGTCGTGGTCGAATTATTTCTTCCTGTGTAAAATCCAGGTTGAGTTGGCTCAGGCAATAAATTTTCACTGTTCAACATGAAAACTACTGAAGACATGTTTGGCCTATCCTCTGGTCTTTGTTGTACGCATAACAAACCAATATGAATATGTCTTAATATTTCAGATGGAACAACTGCAGTGTCCACTGAGTCATCCATTAGTTCCATTGGCCTTTTTTCAATCCATAGTCTCCATGCATGTCCAAGAAGATTTAGCTGATTGTTTGGGTCACGAAATCCTCTATTCTTCCTCCCACTGATTATCTCCAATACAATCACCCCAAAGCTGAATACGTCTGATTTGACTGAAAATGAACCATGCACAGCATATTCAGGAGGCATATAGCCATATGTTCCCATCACTTTATTTGTGTTTGCTTCATCTTGATCCAGGCCAAAAGTTCTAGCCATACCAAAATCTGATATTTTTGGTTTCATATTACTGTCAAGAAGAATGTTACTGGTCTTTAGATCCCTATGAATGATTTTTAGTCTAGAATCTTGATGAAGATAAAGAAGTCCTTTAGCAATTCCACCAATAATTTCAAATCTCGTAGCCCAACCAATTAATGTACTTCTTGTTGAAT

>PvRGL158

GTCTTGTTTCCACGGACTAAGGCTAAGGATGTCTAAGAGTAAACCTTTACTTTCATCTTCCTCCAAGTCACGGTTGGAGAATGACACGGAGAACATGGAAAGGAAGAGATTTAATAGCACGGAATCGTGGTCCATGATATTGGACTCAATGGATACATGGGAGGCTTCAAAGGAAGACCAGGAGGGGGAGCAAGAGGAATGGACTGCAGACCTATCACAGCTTTTTATTGGTAACAAGTTTGCTTCTGGAGCTCACAGTCGAATTTACCGTGGAGTCTACAAGCAGAGAGCTGTTGCTGTGAAAATGGTGAGGATGCCAAGCCAGGATGAGGAGAAAAAGGACTTATTAGAGGAGCAATTTAACTCTGAAGTGGCTTTACTTTCACGTCTCTTCCATTTTAACATAGTGCAGTTTATTGCAGCATGCAAAAAACCGCCCGTGTACTGTATCATAACAGAATACATGTCACAAGGGAATCTGAGGATGTATCTGAACAAGAAAGAGCCATACTCACTCTCAATGGAAACAATACTAAGGTTGGCTCTAGATATATCAAGGGGCATGGAGTACCTTCATTCACAAGGTGTGATTCACAGAGACTTGAAGTCAAGTAATTTGCTCCTGGATGATGATATGAGGGTTAAGGTGGCGGATTTTGGCACATCTTGTCTTGAAACAAGATGCCGGAAAAGCAAAGGGAACTCGGGAACATATCGTTGGATGGCACCAGAGATGATTAAGGGAAAACCTTACACCCGAAAAGTTGATGTGTACAGTTTTGGTATTGTGCTTTGGGAGCTCACTACTTCTCTACTTCCTTTCCAAGGAATGACCACAGTGCAAGCTGCTTTTGCTGTGGCTGAGAAGAATGAAAGGCCTCCTCTGCCAGCTAGTTGTGATCCAGCAGTAGCACATCTGATAAAGCATTGTTGGTCAGCAAACCCCTCGAAACGGCCAGATTTCAGTTATATTGTGTCTACTTTGGAGAAATACGATGAGTGTCT

>PvRGL159

ACCCATGGTTGGCCTCTTCAAAGGATCTGGTTCAAGACACATCATTGCAATGTTGAACACCTTATTTACCTCTTGCATTGAACAAGATCCTAAGCTACTATCAAGGACTAAATCCTCCTTGTTCTCTCTAACAACAGCCTTCACCCATGTGACAAGCATGGTTCCCTCTTCCATAAATGCTTCATCACTGGGTTTTTTCCCAGTTAAAAGCTCCAGGAAAACAACTCCAAAGCTGTAAACATCCCCTTTGAGTGTCGCTCTTCCTGTATCAAAATATTCAGGTGCCAAGTATCCAAAAGTTCCAGCCACTAATGTTGAGACATGAGTTTTATTTGGTTGCATCAAAGTGGCTAATCCAAAATCAGAAACTCGGGCATCCATATTTTCATCCAACAATATGTTACTTGACTTGATGTCTCTGTGGATAATGCGAGGGATGCAATCATGAT

>PvRGL160

AGAAGGAGACAAACTAGAAATAGGAGTAGAATTGGTGGTAGAGCTATGACCATTTATTTGTGTGTGATTGGAATCCTGAAGATGTTCCAATGATCTCACCACATCTTCTATGTTTGGTCTAGCTTTTTGTTCTGTAGATAGGCATTGAATTGCAAGGTAAGCTATTCTCTTGGCTTCTCGCTTAGAGTATTGGCCTTCTATACGTGCATCCATGACTTGAGAAATCTTGTGTTTGTTTATCAGAAGTGGTTTGGCCCATTCAACTAAATTATGCTCCCCACTTGGCCTGTTGTCGTCCAGTGCACGTTTTCCTGACATAAGTTCGAGAAGAACAACTCCAAAACTGTATATGTCACTCCTTTTGGTTAGGTGCCCTGTGGCAATATACTCAGGAGCAGCGTAGCCAAAGGTGCCCATCACCCTTGTAGAAACATGACTCTTGTCACCTTGTGGTCCATTTTTTGCCAACCCAAAATCAGAGAGTTTTGCATTATAGTTAGAATCAAGTAAGATATTAGAAGTTTTGAAGTCCCTAAATATTACATCCACTTCATCACTGTGAAGAAATGCAAGGCCCTTAGCAGCATCAAGAGCAATCTTCATACGGATGTTCCAAGAGAGTGGTTCAATGTAAGAAGCCCTCCTAAACAAGTGATTA

>PvRGL161

CTCTTCTCTCTGATTTCTCTCATAACCCAAGGGACAATGTGGGAACCTTCTGGAATCCTTCTATCTATTGGCTCCATCCCTGTTAAGACCTCAATGATAACTACACCAAAGCTATACACATCACTCTTTCTGTGATCCTTAAACTGTATCCATATTCAGGAGCTATGTATCCATAAGAACCTGCAACTATAGCAGAAGCTCCGGAATTATCAGAGGAACCAACAAGTTTTGCAAGGCCAAAATCTGCAAGAAAAGCTTCAAATTGTGGACCTATCAAGATGTTGTTAGCCTTAATGTCTCTATGAATAATTGGAGGGATACAATCATGATGAAGATATTCTAATCCATGAGCAGCTCCTAGAATGATCTTATATCTTGCATCCCAGTCCAAGAACAAACTACTTTCATGGAGCAATCCAGACAAACTCCCATTGCATATGTAATCAAATAAGAGCAATCTAGTTCTTCCATTGTTGTAGCATCCTAAAAGTCTCACTATATTTTTGTGCCTTATTGATCCAAGGGTGTGAACTTCAGCAGTAAAGAGGTCTCTCTCCGGTGGCTCATCAT

>PvRGL162

GGTGAAGGTGGGTTTGGTTGTGTTTTTAAGGGGTGGATTGATGAGCAGTCACTTGCACCAGTTAGACCAGGGACTGGATTGGTCATTGCTGTGAAGAGGCTAAACCAAGAAGGTCTGCAGGGACACAGTGAATGGTTGACAGAAATCAACTACCTGGGGCAGCTGCATCATCCTAATCTTGTGAAACTGATTGGTTACTGCTTAGAGGATGACCAAAGGCTTTTGGTGTATGAGTTTTTGACCAAGGGAAGTTTGGATAACCATTTATTTAGGAGAGCTTCTTATTTTCAACCACTTTCTTGGAACATCCGAATGAAGGTTGCTCTTGATGCTGCTAAGGGTCTTGCATATCTTCATAGCGATGAAGCAAAAGTGATATATCGAGACTTCAAAGCTTCAAATATCCTGCTTGATTCAAATTACAACGCAAAACTTTCTGATTTTGGCTTGGCAAAGGATGGACCAGCAGGTGATAAAAGCCATGTCTCTACAAGGGTAATGGGCACATATGGCTATGCTGCTCCTGAATATATTGCCACAGGTCACTTGACAAAGAAGAGTGATGTATACAGTTTTGGGGTTGTTCTGCTTGAAATTATGTCTGGCAAACGTGCTCTTGACAGCAACAGGCCAAGTGGGGAGCACAATCTAATTGAATGGGCCAAACCATACCTCAGCAACAAACGTAGGATCTTCCAAGTCATGGATGCTCGCATTGAAGGCCAATACTCTATGCGCACTGCTATGAAAGTATCCAACCTTGCCATTCAATGTCTCTCTGTGGAACCAAGATTTAGACCAAAAATGGATGAAGTGGTGAGAACATTGGAGGAACTTCAGGATTCTGGTGATAAAGCTGTTGCAGGGGTGGGAAGCTATCATTATCAAACTGTGAGAAGGAGTGGTCATAGCTCAAGCAGCAGTGGCTACAGACTACATAGAGGTAAACAACATGAAACAACAAAGAAGTGAAAGTGAATGTTGTTTTCATGTTATCTGATTTTGCTCGAGTTGGTAATTGTATGTATGTATATCATGATGAACTTAAACTTGTCTTAAGATTTCTGATTATTGTACAGTTGTTGATTCAGTAATAATATTAAAATTTTCACATTGGTGTTTTTTTCA

>PvRGL163

GACATGCGGCCAAACAACATTCTTATCACTCATGATTTTGAACCACTGGTTGGTGATTTTGGACTGGCGAGGTGGCAGCCTGATGGTGACACTGGAGTGGAAACAAGAGTAATCGGAACATTTGGGTATTTGGCTCCTGAATATGCTCAAAGCGGCCAAATTACTGAAAAGGCTGATGTTTATTCATTTGGTGTGGTATTAGTGGAACTTGTTACAGGGCGAAAGGCTGTGGATCTCAATAGGCCAAAGGGACAGCAGTGTCTTACTGAGTGGGCACGACCACTGTTAGAAGAATATGCCATTGACGAACTGATTGATCCAAGGTTGGGAAGTCACTATTCGGAGCATGAGGTCTATTGCATGCTGCATGCTGCCTCATTGTGCATAAGGAAAGATCCTTATTCTAGACCACGCATGTCTCAGGTTCTGCGAATACTGGATGGTGACACGGTTATGGACCCAAATTATGTTTCAACTCCCAGTTATGATGTAGGAAACCGGAGTGGCTGATTTTGCTTTTTAGCCAGAAAGGTAAAGCAAAGACAGCGCCATTACAACGGCGGTCCCCTGTTGTAAGAGTCACTGGAAGCTTTCTGTGGGAAGCTGAGTACAAACCTTGGCTATTGGGAAAGCAACAAGTCTGTCTAAGTGGACAGGCAACATGTGAAGAGACTACAGTTAAAACAATTCACGACGAAGATTGCTGTCTTGTTATGCTAAGCCTTAGTTAGTATTTTGCAATTTGTAGGTAGTTATGTTTCTATTTCCTGCAATTTAATCTCATGCTGCATATGTATATGGTAGTAGTGTTAAAGTAGGGTAAGTAATATAGAAGCTTTTGAAATTCTTCAATGAAAGTTTAGCCCTGTGGCTTAATGACCTTTGATCATCAGACTATGCTCATTCAATGGATCAAAAAGCTTTTTTTGCA

>PvRGL164

TTAATATTGTTCCTTCCTCCTACCATCTCTAGTAGCATCATTCCATAACTATACACATCAGACTTTTGCGAAACTCCTCCAAAATTTCTGCTCCACATCTCTGGAGCAAGATATCCCATTGTTCCTCTAGCATCTGACATGGAAATAATGCTTTCATTCCTGGGACACAACTTTGCTAGTCCAAAATCTGATATTTTAGGACACAAATTATCATCTAAAAGAATATTATGTGGCTTGATGTCGAAATGTAAAATTTTAGTATTGCAACCTCTATGCAAGTACTCCAACCCTCGAGCTATTCCTATTGAAATTTGATATAAATTCTCCCAGTTCAAAGATGCAATTGTTGATGGCCCTCTTTTGTAGATAAATTTATCCAAAGAACCATTGGCC

>PvRGL165

TGGAGTCTCTCAACCTTCTTGATCTCTCCAACAATCTTTTAACTGGGAGCATCCCTGAAAGCCTCTCTGTGCTGCTACCAAACTCTATTAACTTCTCGCACAATATGCTTTCGGGTCCAATTCCTCCCAAACTGATCAAAGGAGGCTTGGTGGAAAGTTTTGCAGGGAATCCTGGGTTGTGTGTGCTACCGGTGTATGCTAATTCATCGGATCAAAACTTCCCCATATGTGCAAGTGCCTACAAGAGCAAGGGGATCAATACCATCTGGATAGCTGGGGTGTCAGGGGTTTTGATCTTTATTGGGTCTGCCCTGTTTCTCAAACGTAGGTGTAGCAAAGACACTGCTGCAGTGGAACATGAGGAGACACTCTCTTCTTCGTTCTTCTCTTATGACGTTAAGAGCTTCCACAAGATCTCTTTCGACCAAAAGGAGATCGTTGAGTCCTTGGTGGACAAGAACATAATAGGGCATGGAGGGTCTGGGACAGTGTACAAGATTGAGTTGAAAAGTGGTGATATTGTCGCAGTGAAGAGGCTGTGGAGTCGCAAGTCCAAGGATTCAACTCCAGAGGATCGGTTGTTTGTAGATAAGGCATTGAAGGCAGAGGTGGAGACCTTAGGAAGCATAAGACACAAGAACATAGTGAAACTATACTGCTGCTTCTCCAGCTTCGACTGCAGCTTGTTGGTTTATGAGTACATGCCAAATGGTAATCTTTGGGATTCCCTGCACAAGGGTTGGATCATTTTGGATTGGCCTACTCGTTATAGAATTGCACTGGGGATTGCACAGGGTTTAGCATACCTTCACCATGATTTGCTTCTTCCTATTATCCATCGAGACATCAAGTCAACTAATATCCTGCTGGATGTTGATTACCAGCCCAGGGTTGCAGACTTTGGGATTGCCAAGGTTTTGCAAGCAAGAGGGGTGAAGGATTCCACAACAACTGTCATTGCAGGAACGTATGGTTACTTAGCCCCAGAATTTGCATATTCATCAAGGGCCACCACTAAGTGCGACGTCTACAGTTTTGGGGTGATTTTGATGGAACTGTTAACTGGGAAGAAGCCCGTGGAGGCAGAGTTTGGAGAGAACAGAAATATTGTTTTCTGGGTTTCCAACAAAGTAGAAGGCAAGGAGGGGGCAAGACCAAGTGAGGTGTTTGACCCGAGGTTATCATGTTCATTCAAGGATGACATGATCAAAGTGTTGCGGGTTGCCATTCGCTGCACCTACAAAGCCCCAACAAGCAGACCAACCATGAAAGAGGTGGTTCAGCTTCTGATTGAGGCAGAGCCACGCGGTTCCGATTCTTGCAAGTTGTCAACAAAAGATGTATCAAATGTCACCGTAGTAAAGAAACCATATGAATTATAATTTGAGTAGCTTAAAGGGTAAGAGGGAGCATGTGACAACTACAGATAAGCAATCTCTCTATGGATGTAATGAAACTGAGGGATTGCAGAAGAGAGACCAAATTAGTATAATCTTTTTTTCTTCTAGACAAGTAGTTTGTAGATTC

>PvRGL166

CAAAATCATGAAAACCTCAGAAATCTGTGGCCTTGAATTGAGGTCACGAGCAACACAGCGTTTGGCAAGTTCAGCCATGGAATAAGCCAGTTCCAAAGGATATTCATCTCTGAGATTTGGATCCATGAAACCTCGAAGTTTCTCTTTAACGTTCTCTCCTTCAAGCACCTGATTCACAGTCACCGACAGCATCTGGTCCCCAGATCCATTCTTATCCCCACCAACAGCCTCTCTCCCCGAAAGAAGCTCCAAAAGCACAACCCCAAACGCAAAAACATCCATCTTTGGAGTAATCAAACCATTCTCAATATACTCAGGTGACATGTACCCTTGAGTCCCCACCACATGCCTTGTCAATTGAAATCCCCCATCATCCCCTTCACCCTCCACCGTTCTCGCCAACCCAAAATTCGAAACCTTTGCCCTGAAATTCCCATCTAAAAGCACGTTCCCACTCTTCAAATTCTTATGCACATGAGGTGGACTAGTATAGTTATGAAGGTAATTAAGCGCGTCAGCAACATCATGAGCAATATGAACCCTCTGCACCCAACTCAGACACACCGAAGTCTCGTACTTCTTACAACCACTATGAAGACAATCCTCGAGAGAATCATTCTCAGCAAACTCGTACACAAGATAGGTGTCACCTTTGTGAACACAAAAACCCGACAATCTTATCACATTGAAGTGGTTAATTTTCTTTGAGAATGTTGATCTCACCCGACACATCNNCNTTNNNNANNNTNANCNNNN

>PvRGL167

GGTGGATTTGGAGTTGTTTATCGCACTTTCCTTCGTGATGGACACGCTGTTGCAATCAAGAAACTTACAGTCTCCAGTTTGATCAAGTCCCAAGAAGACTTTGAGAAAGAAATCAAAAAGCTTGGGAAGATCAGGCACTCGAATCTTGTGGCACTTGAAGGTTATTATTGGACTTCATCCTTGCAACTCCTGATTTATGAGTACCTGTCCACTGGGAGTTTGCATAAACTTCTACATGATGATAACAGCAAAAATGTCTTCTCATGGCCACAAAGGTTCAAGATTATTTATGGCATGGCAAAAGGGTTGGCCCATTTGCACCAAATGAACATAATCCACTACAATCTTAAATCAACCAATGTTCTCATTGATTGTTCAGGTGAGCCAAAGGTTGGAGATTTTGGCTTGGTGAAACTACTGCCAATGCTAGACCATTGTGTTTTGAGCAGCAAAATTCAAAGTGCACTCGGATACATGGCGCCAGAGTTTGCTTGCCGCACGGTTAAGATAACCGAGAAGTGTGACATATATGGTTTTGGGATCCTGGTGCTGGAGGTGGTGACAGGAAAAAGACCTGTGGAATACATGGAGGACGATGTGGTGGTTCTGTGTGACATGGTGAGGGGTGCCTTGGAAGAAGGCAAGGTGGAGCAATGTGTTGATGGAAGGCTCCTTGGTAACTTTGCTGCAGAGGAGGCAATTCCTGTGATAAAATTGGGGTTGATTTGTGCATCACAAGTGCCATCAAACCGTCCAGATATGGCTGAGGTAGTCAACATACTAGAATTAATCCAATGCCCTTCAGAAGGACTAGAGGAATTAGAATGAGTTTTGTTGCAGTCTTTGAGAACAACAGCATATATTGCAATACCTGGTGAAGTACAATCCGGTTAAAAAGCTCTATACATGCAAAGAATATGATTCAGTCACCAACTTTTCTTTGTTTAAGTTGTTGAAATTTTCTTCTTTCTTAGGCCTCTTAAATTTTGTTCATCTCTTAATGTATTACTGGATTTTTCTTCACCAGTTAGACGGCTGATACAAGGATAATATATCATATATCTCTTAATCATAGATTTCCCTCCA

>PvRGL168

TGTAGTCTGGTTTGTTGAATTTGATATGGCAAAAGAGTGAATAAAGACAATAAGATTCTCTCTGAACAATTCAATTATCTAATCCTATATACACAAATATTTTCACAAAAGCGAAACCAATTTACTCGCATTTATCATAACAATCCAATGCTCTCATACACTATTTAGGCCTACTCTTTTCCTCAATCTATCTGTAGTCACACTCCCGGGCTGTGTTTATTACAAAACCAGTGTTGTTATGGATCTTTCTGTAATATCAAGCCTTGATGAAGCCTCACTAGTTGGGTAAGACAAAAGATGTCATTGATGGATTATGAGTGAGTGGTAATTTCTCAAACTGATCATGGACTAGAATCCTCATGTGGGTTAGCAGGTAAGACTTGCTGTGGCAGTTGTGTGACTTTGAGTGTTCCTCCAAGCTCTACGGGGACAAGAGGGATAAGTGAGTGGAGAACATCTGCAATCAGGGGACGGTAACTTGGCTCTGGTTGCACGCACAACACTGCCACTGCCGCAACCTGGTATAAGTGTTTGGGGTCCATTGTATTCTTAATTACAGGATCCACGATGTCTGGAAGCTTTTGTCTGTCTGTGAGCTGTGGCATNGCCCATGTGACAATAGATTGGCATTGAGCTGGTGTTAGTTTTTCCACAGGCTTTCTTCCCAAGAGAAGCTCCAACAGCACAACTCCAAAAGCATATACATCACTTTTATCTGTCAATTTACCATCTAAAAGATACTCTGGGGCTACGTAACCCAACGTGCCTGAGAGCTTGATATTATTCTTGTTTTGGGTCCCATTGGTTATGGCAAGACCAAAATCAGAAAGCTTGGCATTGAACTTTGTATCCAAAAGAATATTAGAAGATTTCAGATCTCTATGGATCACTGGAGGGTAACAGTGTTCATGCAGATATTTTAATCCCCTTGCTGTGTCAAGAGCAATTTTCATCCTCAAATGCCAAGTCAATGCTGAGCCATGAGAAGGTCCATGTAATTGTGTTTCCAATGATCCATTATGCATCAATTCATAGACAATAATCCTTGTATCTTCATTACTGCTACATCCCAGTAGAGAAATTACATTTGGATGTTGAATTTTACTTAACAATTCCACCTCGTTCTCATATTCTT

>PvRGL169

GCTCTCAGTAAACATTTATTTGACTGGAAAGAAGAAGGGATACAACCACTGGATTGGAAGAGAAGGCTCTCCATTGCCTTGGATGTTGCTAGAGGTGTTGAATATCTTCACGGTTTGGCCCAACAAATTTTTATCCATAGGGATCTAAAACCATCAAACATCTTGCTTGGGGATGATATGCGGGCCAAAGTCTCAGACTTTGGATTGGTTCGGCTTGCCCCAGAAGGACAGACCTCATTTGAAACTAGGCTTGCTGGTACTTTTGGATATTTGGCTCCAGAGTATGCAGTGACTGGACGAGTTACAACAAAGGTTGATGTTTACAGCTATGGAGTGATCTGATGGAGATGCTAACAGGAAGGAGAGCAATTGACAACAGCCAACCAGAAGAGAATGTCCACCTTGTTACATGGTTCAGGAGGATGCTACTGAACAAGGACTCTTTTGGCAAGATTGTTGACCCC

>PvRGL170

TGGGGTTTAGGCATCATCTATTTGGATTATGGGAACTCGTCCATTACAAGAAAATTGGTGGAAACTTGCATTCACAACACAACAATGAGTGTTGCAAAGAAGTTGAGTGAGTAAATGTTTCTACACAAAATAACATGGCAGGTGAACCTATTTTATGGATTTGATGGTGCACTACTTGTAGTTGATGGTGACTATGATTGTGATGCTAACCCAAAGTCCCTCAAATGGTTAAGCTTTGGCACTATCTCAGTTGCAAGATCAGGCCTGTCTCTTTTGCTGAGCTCTGCACAACTCAATGAGAGTTTGCCAAATTCAAGAGTCTCTTCAAGGGGCCAATTAGTGATCACAGGATCAAGCATTTCTGAAAATGTTCCCTCTTGAATGGCCCTCATAACATGGTGAGCAAGCCCCATTGGAGGCTTGGCTGTGATGATTTGTAGAAGCATTATCCCCAATGAATATACGTCTGATTTTGTTGTTAGCATCCCAGATTGTTGGTACTCAGGGTCAATGTAACAAAAGGTTCCTGCAGCAGCAGTCAAGTGATATTGTGTGACAGAATCGGCCACAGAAGCTGGCACTAGTCTTGCAAGACCAACATCACTAATTTTGCTCACAAAATTCTTGTCCAAGAGAATGTTGGCAGGTTTGAGGTCCCTGTGCACAATTGGTTCTGGCTTTGTTTGGTGAAGGAAAAGAAGCGCAGTTGCAATCTCAGCAGCTATTTCAAAACGTTTCCACCATGGAATTGGAGGACTATCATTTTTCATCAATAATCTATCTTCTAAGCTACCATTCTCTAAGTACTCATACACTAAACATCCATATTCAGGACATGCACCAATGAGGAGAACCATGTTTGGATGCCTTATGCTGCATAATACCTCAACCTCTTGTTGGAACTGCCTCCTGCCATGGGTAGCATCAGGGTTCAAAATTTTGATTGCTACTTGG

>PvRGL171

ATATGGAGGAGGTTGGCATTTTCCGTGAGAACCCAGTTGAAAGGTGTTGCTGCCTCCACAAGAAGCCCTCATTGGTTCCAGTATTCTCCAAACATCTGGATAAAAATATGGTCTGTTTCTTCTATTCATCTCACAACACCTCAAAGCCAAGCGAACCAACTCTTCAGCTAGCATAAATGGCCAATCTCCAGCCAATGGATCCAAGATGGATTTCAACTTTCTACTATCTAATGCATATTGCACTTCCTTTGCTATTCCCAAAGCAGGTTTCCCAGTGATCAATCTCAATAGAATGATTCCAAATGAATAAACATCTGACTTTGGTGTGAGTTCACCAGAAGTAAGGAATTCAGGATCCACATACACAAAAGTTCCCTTTGGAACAGTTCTCCAAAACTGTGTAGTGCTGCTACTTGAACCCTCTTGACAAGATAATATACGACAGATTCCAAAGTCTCCGAGCTTGCTTACAAAGTTTGCATCAAGGAGAATGTTGCCTGGTTTTAAATCACCATGTGCTATGCTGTGAGGTTTACTTGAGTGAAGAAAGATGACAGCCGAGCAGAGTTCTGCAGCAACGCGAATTCTAGTTTGCCATGATAATGGAGGTGTGTTATCCTTGCAGTTTAGACGATCTTCAAGGCTTCCATTGGGTAAATACTCATAGACAAGAGCCCATGCATCTGGGCAGGCTCCAATGAGTGTGATAAGATTGGGATGCCTTAGCTTGCTCAACACATCAACCTCCTGTTGAAACTCCAAGGGTCCTTGCATGCTGTCAGAGTGCAACATTTTTANAGCCACCTCAGTGTAACTCAAGACTAA

>PvRGL172

GTTGTTGCGGTGAAACAATTAAAGTTTGGGGGTTCTCAAGCAGATATTGATTTCTGCAGGGAAGTTCGGGTTTTGAGTTGTGCACAACACAGAAATGTTGTGCCGTTGATAGGATTTTGTACAGAGAGCAATTTGAGGATATTAGTTTATGAGTACATATGCAACGGATCCTTAGACCTCTACTTATATGGAGATGAGATTATGCCTTTCGATTGGAACTCACGGTTAAAGATAGCAATTGGAACTGCACGAGGCTTGCGCTATCTTCATGAAGATTGTAGAGTTGGTTGTATAGTGCACAGAGACCTTCGACCCAAAAATATACTTTTAACCCATGACTTTGAACCTTTGGTGGCGGATTTTGGGCTTGCTAGATGGCACTCAGAATGCAATATAAATACAGAAGATCGGGTTATAGGAACTTCAGGGTAATGAATTGGCTTATATGCATGTATATTTTCTTATCATCAAGATAAGGGACATCACGGTTTTGATTCTAATGGAGGTTACCTTATATAAACTAGGTATCTTGCACCGGAGTATCTTGAGACTGGAAACCTTACATATAAAGTAGATGTATATGCATTTGGAATTGTATTATTAGAGTTAATAACAGGTAGAAGAATTGGCGAGTTGGAACAGTTCAATGGACTCTCGTATCTTTCTGAATGGTTTCACCCTCTACGCATGTCAGAGCCCAGTCATATATTACAAAACGTTCGATCTCTCAAACCATGCTTCAACTCTGATGAGTTATTGGCATTTAATCTTCAATTTCAAGCCATGGCACGAGCTGCTTCGTTGTGTCTCCGTGTGGATCCTAATGCCAGACCCCCAATGTCAAAGATCCTGAGAGTGCTGGAAGGGGCTGATCCAGTTCGTCCT

>PvRGL173

AAATAATTTTGACTCTTCAATTCTGTCATGGGTGTTGAATGTTAGCAAAGTCCTCACAGTTCTTGATCTCTCACATAATGAACTTGATGGTGGCATAATAAAGTCCTTTAACACTTTGTGTCAACTGAAGAAGTTGTACCTGGGTTCCAACAAATTGTCTGATCTACTCAGTGATTACTTACCAGAATTTTGTTCTGCAAAAGATTTAGAGGTGTTGAATCTCGACCACAACGCATTTGTTAATGGGTCACTTCCTGATTTTTCAATGTTTTCATCCTTGGAAAGACTATCACTTCAAAATACCAGTATTGTTGGGCCATTATCATTTGGTCACTTGCCTCGTCTTAAAGCTTTGGATCTTAGTTTAAACAATTTGAGTGGATCATTGCCTGTATTCGAGGTTACTAAATATTCCTCATTGCATTTTTTGGATCTTTCACACAACAAATTGAGTGGGACACTTCCATATACTATTGGACAACTTTCTAATCTCTGGTTTTTGTCCATCTCCTCAAATGAGTTGAATGGGAACATTAGTGAAGAGCATTTATCGAAACTATCTGGATTGAAGATATTTGATGTGAGTCAAAATTCTCTTTCATTTAACTTGAATTCAGATTGGATTCCACCTTTCCAATTAGTGGCATTGTATGCATCATCCTGCATTTTGGGGCCTCAATTTCCAATGTNGCTTAAACAGCAAAGGAAACTTCAGGTGCTGCAAATCTCTAATACCCACATTATGGATGTATTTCCTGAATGGTTTGGGGACATATCATCAAGTTTGTTATACATAAATGTTTCCCAAAATAAACTTAGTGGAGTCTTACCAAAATCATTATCAGGTATTGAAACAGGGATCA

>PvRGL174

TGTTATGAAGGAAAAACTTAAGCTGAAATATGTAATCAATTTAATTTGATATAACATATAAGAATACTATCTAGGGAAGAATATGGTAGTAGACATCTTATTTACAGAGGAGGAGAAAGAATGATTGGTGGAGTAACCTGATGATGGTTGATGTTCAGTTGAATGTCTTCTTACTCTGCCATGCATGAAAAACGCTGGCTCTAAAGGGATTGGAACTTGAACTGAAGTGTTGTTAAGATATGAAACGATGGTTCCTATTATAGGTCTCTCATCAGGATTTTCTTGCACACACAATAAACCAATCTGTATACACTTTTCAACTTCGTTTGGACAATAAGATTCTTCAAGAGCTGGATCCAACAACACAAATGGTGATTCATCCCTCCAATTGTTCCATGCATAACTCAAAAGATCATCAACACGATGTGATTCAAAAGAACAAGAATTCTTCTTTCCACTAATAATCTCAAGAGTCATAACTCCAAAACTGAACACATCAGACTTGTCAGAAAATTTTCCATGCATGGCATATTCAGGAGACATGTACCCGTATGTACCCACAACTCTTTTCGTACATCCTTGAATATGATTTGTATCTACCATCCTTGCCATGCCAAAATCGGAAATTTTTGGATTGATGTCATTGTCCAATAGAACATTGCTTGGTTTGATGTCACGGTGTATAATCTTAAGACGAGAGTCTTCATGAAGATAAAGAATTCCTCTTGCAATTCCTTTTATGATTTTAAACCTCTCTGACCAAGTCAATACTCTACGTTTTTGGGAATCAAATAAAAAGTGATCAAGGCTTTTGTTAGGCACGTATTCGTAGACAAGTATTTTCTCTCTGTCTTCTTGGCAAAATCCTATTAACCTCACCAAATTTTTGTGTTGAAGTTTAGCAATTAACAACACTTCATTCTTGAATTCCTCCTCTCCCTGCTTAGAATTTGTTGACAATCTCTTTACAGCCACTTCTTCTCCGTTAGGAAGAATTCCCTTGTAGACTTCTCCGTAACCCCCTTTGCCAATCCTGCGCTCTTCAGAAAACTTGTTTGTTGCTGCTTCAATTGTAGCAAAATCAAATTCCAGGGACTCCAACACACTGATTTCTTCCCCAACTGCATTACAATTCACAGAGCAGTTACTAGAAATACTCAAAGCAAGTACATGTAATTTATAAAATAAAAAAACTAGAGGCTTTTCTACTAAGCTCAAATGTTGAAGATGATAAGGTTTATTGAGATTACAGTTTTCTCTGTCCCAGGCCGTAA

>PvRGL175

GAGAAAAAAAAAATCAAAATGCATAAAACTGTAGTAGTTGTTGTAGTTGTATCATTCTCTTTTTCTTTTCACTACCAATTGTATAGCATTCGGTTACAGTTACAAGTATAAAATTTACAAGGTCCCAAATGGGAAGTGCAGATAATAGCTATGTCCCTAGTAACTTCCTACTTTTACCATCTATCAAAATCATGGTACAAAAAATATTATAATTACATTTTTCAGAATATGTGAAGGAGAATGGCACAGTACAGCATCAGCGAGGAACAATGGTGGGAATGACATCACTGACAAGATCGCTTCCTGACACATATGAGGACATGTTTTGCTCCTCCCTTATTACATGAGAACCTGTTGTTGATGCTGATGATGAGGGTGGTGCTATGTTGCCAGAACTGTCTAAGCTAACATCTGAGAAAATAGTTTCAGCTTCGGGCAACATTGCAATGATATCTTCCAGTGTCCTCACCACATCTACCATTGATGGTCTTTCCTCTGGATTGTCTTGGCAACAACTGAGGGCCAGAGTTAAGAATTTGTCCAAGCATTCAGATGTGTACAACCCAATTGTGCTGTCTATGATGGAATATACTGTGCCAGATTGAAGAGCCATGTTAACCTCGCGGACAATGTTTTTCCCATGAGATATTGGTTGCATGCCTGTTAAAAGCTCTAGATACACAATTCCCAGGCTATAGACATCACATTTGTCTGTCAGCTTATGAGTCAACAAATATTCTGGGTCCAGGTAACCTGGAGTTCCTCTCACAACTGTTGACACATATTTAGGAGCAGTTCCTTCTTCATCTAAGTCTGGTACAAGCCGTGACAGTCCAAAATCGGCCACTTTAGCTGTGAACTTGGAGTCCAGAAGAATGTTACTGGCTTTAATATCTCTGTGGAATATAGGAGGGTTAGCCTCTGTATGCAGATACAGTATGCCTTTGGCTGCTCCCATTGCAATTCTCAACCTCATACTAAAATTTAGGCTGCCCCTGGTTTTTCTTCTTCTACCAGAAATCCAGTCCCTTAGAGTGCCATTGGGCATGAAATCATAAACCAGTATCTGCTCCCCTTCTTCGTTACAGTATCCAATCAGCGAGACAAGATTTCGATGGTGTAACCTTGACAAAAGCTCAATCTCAGTCAAGAACTCCCTTTCTCCTTGTAAGGAACTTGCTTCTGCCCGC

>PvRGL176

TTGTAGTGAGAATTTCATATACCATTTTTAGCTGGAATTAAAGACCATGATAGGATTGAAATTGGAGATTTTGTTACAATTGTTGTTTCAAGAACTATTGCCTAAGCAAAAACTAACTTTTAACTAACAATTTACCAAAGCTGAAGATTATTTTGATTTTGAAGATGAAGTCAAACTAAGCTTCCCTGAAGATCATTATCAGAGGCCTCTTGAAAATAAGGAGAGAGTTTTCCACTTAAGGATTTGGGAAGTGCAGGTGCAGTTACTTCCTTCAGCATTTTCACCACTCTACGCATTGAAGGACGGGTTATAGGAAGAGAATTAGTGCAAAGAAGTCCCACACTGAGTACCTTCCTCATTTCTTCCCTATATTCTGTATCCAAACTTGGATCAATCAACTCTTCCACTCCTTTCTCATCCAATGTAGAGTAAACCCATTTCACTAGATCCTTTTCTCCATACTCTTGATGAAGAGGGGGCTTCCCAGTTACCAACTCCAAAATTACCACACCAAAACTATATATGTCACTCTTTTCATTCACCCGAAGAGTATAAGCATATTCTGGTGCAATGTAACCGTAAGATCCAGCAATCACAGACATGGATTCTGCACCTTGGTTGGCTCCCTTCACAATTTTGGCAACTCCAAAATCGGCTACTTTTGCTCCAAATTCATCATCTAGCAAGATATTGCTAGATTTGACATCCCTATGAACAATAGGGGGGACACAATCATGATGCAAATAGGAGAGCCCCTCAGCAGCATCGATAGCAATTTTGTATCTGGTGGGCCAATCCAGCAAGCCTTTCTTGCTACTATGCAACAAATCAGCTAGGCTCCCATTTGGCATATACTCATAAACCAAGAGCTTGCTATCTTTATTGTTGCAGCAACACCATAATCTCACAATATTCTTGTGCCGAATCTTACCCAGTGTTTCAACCTCAACTTCAAATCCATCCTTCTCAGAATCAACAGATCCATTTCCCATGTTAGGTGTTCCGAACAATTTCTTCACAGCCACCACCTCACCATTGCTCAGCGCAACTTTGTAAACCTTCCCAGATGCTCCACTTCCTATAACATTGTCCTCACTTAGCAATTTAACAAC

>PvRGL177

TCTTCAAACATTCTTTTAGATTTCAATTATGAAGCTAAGGTCTCAGATTTTGGGCTAGCCAAATTAGCTCTTGATGCAAACACACATATTACCACCCGTGTCATGGGAACTTTTGGGTATGTTGCCCCTGAATATGCTTCAAGTGGCAAATTGACTGAGAAGTCTGATGTATATTCTTTCGGAGTTGTGCTTTTGGAGCTAATTACTGGACGGAAGCCTGTAGATGTATCCCAACCTTTGGGAGATGAAAGCCTGGTTGAATGGGCTCGGCCTTTA

>PvRGL178

CATTCTGAAAATCCAATCTGCATATGCTGAGAAAATATCCCACCTTTTTTACAATAAAATACAAAATTAATGAAAGCACATTTTAGTGGAAGACCCTCTTTTCCAAGCCACTTCCAATCTTCACTAATGTGCTTCATTTCTACATAGTGCCTAATTCATCAACTTCACATCAATCATACTTGACAATTTACCTGGCATTTGTAGCACAAAATTGAGCAACAATACTTCAAGAATTGGGGATGAAAAATCCCTTCTTTTAGAACATTGTTTCCTTCAGTTTCCTTAATTGGACATCACATAAGCTCTTTCTTAAGACATTCATCGAGCTGAGAGGATAGAAATGGAAGCAGTAGCATTAGATGTAGAGGAAGCAGTTGAGGTAGAGGTGTCAGCTGCACGAGTCCTGAAGTTAGATTCAACAAATACAGGCATAGAAGGCCTAGTTTGCCCCACTGAATTCTTGCTTTTCAGTGAGGCTACTACTTCAGACATTGTTGGCCTTGCAGCAGCTGATGCTTGAATGCAAAGCAAAGCAATCTCTATCACTTTCTTCACTTCTTCTACCTCATACTCTTCAGGATCCAATGTCTTGTCAACCAACCTCAAATGCATGTCTTCCTCATAAAGTTTCCATGCTCTCTGAAGGAGGAATTCACCATTAGCATCTTCCCTCAATCCAGTATTCTTTTGGCCACTTACTATTTCTAGGACTACAACACCAAAGCTGTAGGCATCAGCCTTCTCTGATAACTGACCATGGATTGCATACTCAGGTGCTGTGTATCCCAATGTTCCTGCAAATCTTGTGCTAAGATGAGATTGGTCATCTGGTAGAAGTCTTGCCAACCCAAAATCAGCAATTCTTGGTTGCATCTCATCATCCAAGAGGATATTGCTAGCCTTTATATCCCTATGTATGATGCAAACATGGAAATCCTCATGTAGATAAGCCAAACCCTTTGCTGTNACCTAAAATTATATCATATCTTTGTTTCCAGCTGAGGGAGTCCTTTCCTTCACCAAATAAGAATCTATCC

>PvRGL179

AAAAAACTCAGGAGCATTATGCCATATGGAGTTATGATGGAAAATTAGTGTATGAAAATATCATTGAAGCCACAGAGGAGTTTGATGACAAATATCTCATTGGAGAAGGAGGGACTTCATCTGTTTATAAGGCTAAATTACCCACAGGACAGATTGTTGCTGTGAAGAAGCTTAAAGATGCACCCAATGAAGAAACACAGGATTTAAGAGCTTTTATGACTGAGGTTAAAGCCTTGGGAGAAATCAAGCATCGTAACATTGTGAAGTCACTAGGATATTGTTTACATCCACGTTACTCCTTTTTGGTTTATGAGTTCCTAGAGGGGGGTAGTTTGGATAAAGTACTCATCGATGATACACGTGCAACAATGTTTGATTGGAACACGAGGGTAAAGGTTGTTAAAGGTGTGGCAAATGCTTTGTACCATATGCATCATGGTTGCTTTCCTCCTATTATTCATCGAGACATATCAAGCAAGAATATTCTTATAGATTCAGAAT

>PvRGL180

CGAGTTATGGGCACATTTGGGTATGTAGCTCCTGAGTATGCTATGACTGGACATCTACTTGTTAAAAGCGACGTTTATAGTTATGGGGTTGTCTTGCTGGAACTGCTGACCGGAAGGAAACCAGTGGATATGTCACAGCCAACTGGACAAGAGAACCTTGTTACTTGGGCAAGACCTATTCTTAGAGATAAGGATCGGTTGGAAGAACTTGCTGATCCAAGGCTTGGGGGAAGGTACCCAAAAGAAGATTTTGTACGTGTTTGCACTATCGCAGCAGCCTGTGTTGCTCCTGAAGCAAGCCAGCGACCTACAATGGGTGAAGTGGTACAGTCACTTAAAATGGTGCAGCGCATCACTGAAAGTCATGATCCTGTGTTAGCCTCATCCAATGCACCGCCCAACATGAGACAGTCCTCTACAACTTATGAGTCTGATGGGACTTCTTCAATGTTTTCTTCGGGTCCTTACTCTGGTCTAAGTGCCTTTGACCATGACAACATTTCGAGGACGGCTGTTTTCTCTGAAGATCTTCATGAAGGACGATGAAAGTGCTTGGAAGAGCAAGCTAACCCCGAATATGTACCCAAAGGTTTTTCACACCATGATTTTGATAAGTTTTCTTTCCCATAGCTGAAGGTGGAAAACATAACTGGTGGTGTTTTCTTTCAAGTGATTGGGGATTTCTTGGTGTTGTTGCTGACTAGAGAGGATTCTTCTGAGGTCGCTTATGCCATCCTCCTCGGTAAATCACCATTCTTTCCTCCTCTACTAGACAGTGTTTTCACGTGTGGATGAATTTTGTATATATGTGTAATCTAGTTTGTTTTTACAGATTGGCATCAAAAGCCTTTTTAATTATAAATATATATACCCCCGCA

>PvRGL181

TGGAAACACCAGATAAATCTGCTACACTTCCCATGCTGTATTCCATGGAAGATAATCCATTATCTAAGCTATAAGTTTGGGAAGCCACATCATCAACATGATTGGATCCGTCATCAACATCTTCTAGTCGCAAAGCATATTCCAAGTGCCACAACACATCTCCCATGTTAGGCCGATTGATTCCGTGTTCCGACAAGCATTTTATGGCAATCTCTATGAATATCTGGAGTGACTTTGAGTTCATCTGAGCTGCAACCGCAATGGGAGGATCAATAATTTCATATATTGAGCTTCTCCCCTGCCATTTCATTATCCAATCCACTAGATTCACCTTGTCCCTTGTGAGTGATGGATCAATTGCTGGTCTTCCACATAGTACTTCAAACATCACCACCCCAAAAGAGTAAACATCTGATTTTTCGGTCAACTGTTGCATTATTAAGTATTCTGGATCAAGATACCCAAAGCTGCCTTTCACTGCTGTACTGACATGTGTCTTATCACTCTCTGGACCAGTCTTTGAAAGACCAAAATCGGCAACTTTGGCCGTGAAATTTTCATCAAGCAGGATATTTGCCGTTTTGACATCACGGTGAATTATCGCCTTGGCAGAGCCAGTGTGAAGATAATGTAGTCCTCTGGCTGCTCCTATGCAAATCTCAAGCCTCTGTCTCCAACTTAAGCAAGGTCTCTCTGCATCTGACCCGAACATATACTCCTTCAGGCTCCCTTTTTCCATGTACTCGTAAACAATGATCCTCTCATTTTGCTCGTTACAATACCCAATCAATGATACCAAGT

>PvRGL182

AATGCACACTTCTAGGCGCTGCTTCCAAGGTAAGGGAGGGAGATCACTCCCAAATAGGTGGCTTCTGAGAGTTCCATTGGCCATGTACTCATAAACCAAGATCATTTCATTCTTCTCTTCGCAGAAACCAATTAAGGACACCAGATGTCTGTGTCTGAGTTTGGACAGCATCTCAATTTCTGTCTCAAATTCAGCCAGGCCTTGATCAGACTGGGGGTTGGCGCGCTTAA

>PvRGL183

ACCATATGTTCCAACAACTCTCAATGTGTTTCCTTCTGTCTGATTCGCGCCAAAAAGTCTAGCCATCCCAAAGTCTGATATCTTTGGGTTCATTTCACTGTCAAGTAAAATGTTGCTTGCCTTGAGATCCCTATGGATAATCCTGAGTCTGGAATCATGGTGCAGATAGAGTATCCCTCTAGCTATGCCACATATAATGCTGAAGCGTCTATTCCAGTCCAACATGTGTTTTTTGGCTTTGTCAAACAAAATGGAATAAAGGCTTCTATTTTCCATGTGCTCGTACACTAGCAACTTCTCATCCATCTCAACGCAGCATCCAAAGAGCCGAACAAGATTTCGATGTTGAAGTCTGACAAT

>PvRGL184

TGGATACTGCTGCGAGGATCAACATAGGGTACTAATATATGAGTACATGTCCCGGGGAAGTGTGGAGCACAACTTGTTCTCAAAAATCTTGCTTCCCCTGCCATGGTCCATAAGAATGAAGATTGCATTTGGTGCTGCTAAGGGACTTGCATTTCTTCATGAAGCAGAGAAACCTGTCATCTATCGTGATTTCAAGACATCAAATATTTTATTGGATCAGGACTACAATGCAAAACTTTCTGATTTTGGCCTTGCTAAGGATGGACCAGTTGGAGACAAA

>PvRGL185

GCAAATGTTAAACATGCAAACCTTGTTGAATTGATTGGATTCTGCATCCAAGGACCTAGTCGCACATTAGTCTACGAGTATGTGGAAAATGGAAGCCTTAATAGTGCACTACTTGGCACAAGGAATGAAAAGATTAAACTAGACTGGAGAAAAAGATCTGGTATCTGTGTAGGTACTGCTAAGGGTCTTGCTTTTCTGCATGAAGAACTTTCACCACCAATTGTGCATAGAGACATCAAAGCTAGTAATGTACTACTTGACAAAGATTTCAATCCCAAAATAGGTGATTTTGGGTTGGCTAAACTATTTCCAGATGGTATCACTCACGTTAGCACAAGAATTGCTGGAACAGCTGGTTACTTAGCACCGGAATATGCAATAGGTGGCCAGTTAACGAAGAAAGCTGATATATACAGCTTTGGGGTTCTTGTGCTTGAAATAGTTAGTGGCAGAAGCAGTGCCAGGAGAACAAACGGAGGAGGATCACACAAATTCCTCTTGGAATGGGCCTGGCAACTTTATGAAGAAGGAAAATTGTTGGAGTTTGTGGATCCAGAGATGGAAGAGTTTCCTGAGGAAGAGGTAATGAGATACATGAAGGTTGCACTTTTCTGTACACAGTCAGCAGCAAGTAGAAGGCCTTTGATGATACAAGTTGTTAGTATGCTATCAAAGACAATCCAACTCAATGAGAAAGAACTTACAGCACCAGGATTATTCACAGATGAAGGAGAATCTTCTCGAAAACACCCTCTTCCTTTTATTACCTCTACTCATGTTGCTATTACTCAGGTCACCCCAAGGTGATGTCTATTCAAGAGTACTTTTTGTTTTTCAAATATTGTTTTATTTTCTTATGTTATTTCTTTTCAATTTAGCTATAAAAAAATATAACTCGGCATGAAAGCAATCTCGTGCTCCTGCATANT

>PvRGL186

GAGGTTGCTGTGAAAATTCTGATGGAACAAGACTTTGCAGGTGAACGCTTCCAGGAATTCTTGAGGGAGGTTGCAATAATGAAAAACTTACGGCATCCAAACATTGTTTTGCTTATGGGTGCAGTCACTAAGCCTCCCAATTTATCAATTGTCACAGAATACTTGTCCAGGGGTAGCTTGTATAGGCTGTTGCATAAACCTGGTGCCACAGAAATGTTGGATGAGAGACGCAGGATTAGTATGGCTTATGATGTGGCTAAGGGGATGAATTATCTTCATAAACGAAATCCTCCCATTGTTCATAGAGATCTGAAATCTCCAAACCTTCTTGTTGACAAGAAATATACAGTGAAGGTTAGTGATTTTGGGCTTTCCCGACTAAAGGCAAACACATTTCTCTCATCCAAGTCAGCTGCTGGGACACCTGAGTGGATGGCTCCAGAAGTTCTTCGTGACGAGCCATCCAATGAGAAGTCAGATGTTTACAGCTTTGGTGTCATCTTGTGGGAGCTCGCAACATTGCAACAGCCTTGGAGTAATTTGAATCCACCACAGGTTGTGGCAGCTGTTGGCTTTAAGGCAAAAAGACTTGAAATACCACGAGATTTAAATCCTCAATTAGCATCAATAATTGAGGCTTGCTGGGCCAATGAACCCTGGAAACGCCCTTCTTTTTCAAGTATTATGGATTCTTTGAAAGGATTGATCAAACCCCCTATACCTCAACCTGGTCGTCCAAACATGTCATTACCCATTTGAATGGTTGGAACGATAGTTGTTATTCATGCACATACTACAGGATTTTGTTAGTTTGATGTCTATCAGGAATACACTGTTTCAATAGTCCCAGAGGCACATTTGTACATTTGTGTTCAAATTCGGATTCAGAATACCTATTTTCGACACAAAGCCATACCCCATCTTTGATGCAGCAAGTATTGTTGGGTTAGGTAATCTTGTAATTTTATGAAGAAAGTGCATACTTTTTTTCATCCATAATTTTTTTAAAAAAAATATTCTCATTGTAACTATAATCACTTAATGATAAGCGACTTTATCGTACCA

>PvRGL187

GTATGGACTCACCGGCTGATGGTCAAAATGCAAATGGATTATCCCTTGAATATTTGCACCGTTTTAGATTTAATATTGATGAGATAGAATCAGCAACGCAGCACCTTTCTGAGGCCAATCTATTGGGTAAGAGCAAACTCTCAGCAGTCTATAAAGGTGTTCATAGAGACGGTTCTCTTGTGGCTATTAGAAGCATTAGCGTGACTTGCTGCAAAACCGAGGAAGATGAATTTTTGAAGGGGTTGATCCTATTAACCACTCTGAGACATGAAAATATTGTTAAGATGAAAGGTTTCTGTTACTCAAGAAGCAGAGGGGAATGGTATTTTGTCTACGACTTTGCCACCAGGGGTAATCTGTCTCAATATCTTGACAGGGAAGATGGAAGTGACCGTGTACTTGAATGGTCCAAGAGGGTTTCTATCATCAGGGGTATTGCAAAAGGTATTGGTTATTTGCACAGCAACGAAGCAAGCAAGCCCACAATAGTACACCAAAACGTTTCAGTTGAAAAGGTTATTCTAGACAATGAATTTAACCCCTTGATCATGGATGCTGGGCTCCCCAAGCTTCTTGCAGATGATGTAGTTTTCTCAGCTCTTAAAGCAAGTGCTGCCATGGGATACCTGGCACCAGAATATATTACTACTGGATGCTTTACTGAGAAGAGTGATATATACGCATTTGGGGTTATTGTCCTCAACTCTCTCTGAAGGNNNTNNNNNGGGGNTNNN

>PvRGL188

ACCAATTTCATGGAAGAAACGGTTCGAAATATCTGCTGAGATTGCAACTGCACTCCTTTTCCTTCACCAAAACAAACCAGAACCTATCGTGCACCGAGACCTCAAACCATCCAACATTCTCGTGGACAGAAATTACGTGAGTAAAATCGGTGATGTTGGTCTTGCACGTTTAGTCCCTGCTTCTGTAGCTGATACAATGACTCAGTATCACATGACTTCGGCTGCTGGAACCTTTTGTTACATTGATCCTGAGTATCAACAAACGGGAATGCTAACACCAAAGTCAGATATATATTCTCTGGGGATAATGCTACTTCAGATAATCACAGCCAAGCCTCCCATGGGCCTTGCTCACCTAGTTAGGAAGGCAGTTGAAAAGGGCAGGCTTGAAGAAGTGCTCGACCCTGTGATCACAGATTGGCCAATGGAAGAGGCTCTATCATATGCCAAATTAGCATTGAAGTGTGTCCAACTCAGCAAGAAGGACAGACCAAATCTTGCAACGGAGTTGTTGCCTGAGCTTAACAGGTTAAGCAATCTTGAATTGCCTCCACAGAATGATCGATTTTCTTACAGTAGTGCTGATACGATCTATAGCTGTAATTCACATCCTCCCACACCTCCGCGAAGTCAATACTCTGATTCAGAGACATCCTACAGGATCTAGGTTCGAGCATTGACTTATATGTCATGTACAAAACAATCAAAATGTAATCCATACAATTCAAATTATTGAAATATTTGAAACTGAAAGGAGCAAAACACAAGCCATCAATACTTAATTCCTAGAATATTTTCCAGTTGTAATTTTTTTTGTATTTCTTAACAGTTGTAATTATTGATATGGTTTCCACCAACTAGGAAGTAATATATAGGATGGTGTTGGGGAATTTAATGCTACCCCAAGAAA

>PvRGL189

GAGTATATGGAAAATGGAAATTTGGATAGCATCATACATGGTAACGGGGTAGATCATCCTGTGATCTCTAGGTGGACATTATCTGAAAGAGTCCAAGTCTTCATATCTATTGCTAAAGCATTGGATTATTTGCACTCGGGTTATCATATTCCCATTGTGCACTGTGATTTAAAGCCTTCTAACGTTCTTTTAGATAAAGAGTGGGAAGCTCATGTCAGTGACTTTGGGACAGCTCGAATACTTGGTTTTCATCTACAAGAAGGTAGTACCCTTTCGTCATCAGCAGCTGTGCAAGGCACAGTTGGCTACATGGCTCCAGAATTTGCCTACACGAGGAAAGTGACCACTAAAGCAGATGTGTTCAGCTTTGGTATCATAGTAATGGAGTTCCTAACAAAAAGAAGGCCGACAGGACTCTCGGAAGAGAATGGTTTACCAATCACTTTGCGTGAATTAGTGGCAAAAGCCCTTGCAAATGGAATAGAACAACTTGTCAATATTGTGGATCCTTTGTTGACCAGGAATGTTACAAAAGACAATGATGAAGTGTTGGCAGAGCTCTTTAAGCTATCTCTGTGCTGCACCATACCTGATCCTGAACAGAGACCCAATACGAATGAGGTGTTATCTGCCCTTGTGAAGCTTCAAACAACACTGTCTTGTTAAATGATAAACACCACCAACCAACCAATTCTTG

>PvRGL190

GGGAAGGCACACTAGGGCCTGTTTATCGGGCTGAACTCCCTGATGGGAAGCTATTGGCAGTGAGGAAACTGGATGCTACTGCTTCCATGGGGCAGAGTCATGAGCAATTTCTTCAATTGGTGTCCAGCATCTCAAATATTCAGCATGCAAATATTGTAAGGCTTATAGGCTACTGTGTTGAACATAGCCAACGACTACTTGTATATGAGTATTGCAGTAATGGGACCCTACATGATGTACTGCATGGGGACGATGATCAACGAATCAAACTTCCGTGGAATGCACGCATTCGAGTAGCACTTGGAGCTGCCAAAGCTTTAGAGTATCTGCATGAGAACTTTCGGCCACCTATTGTGCATCGAAATTTTAGGTCTACTAATGTTCTTCTGAATGACAATTTGGAAGTGTGCATCTCTGATTGTGGATTAGGGCCATTGTTATCTTCTGGCTCTACGGGTCAGTTATCAGGACGTCTCCTTACTGCTTATGGTTACAGTGCTCCAGAATTTGAGTCTGGAAGTTATACGCAGCAAAGTGATGTCTTCAGTTTTGGTGTTGTGATGTTAGAACTCCTTACTGGACGAAAATCTCTTGACAAGTCCCGGCCTCGTGCAGAGCAATTTTTGGTCAGATGGGCTGTTCCTCAACTCCATGACATTGATACCTTGTCAAAAATGGTTGATCCCTGCTTAAGCGGAGCCTATTCTATGAAGTCTTTGTCACGTGTTGCAGATATTGTTTCTTCATGTCTACAGCGTGAACCTGAATTCCGACCTGCAATGTCGGAAATTGTTCAAGATCTCCTGCGAATGATGTGAAAAAAATGCAGCAATCCGTGGGAAGATGAATTCCCTTAGCCAGGAAATGGTGCTGATGATAAGCTTTGGTTCCAAGATCTTGTCTATTTTTGTTTATTAATTTTGTGAGCCAAAGATTTTTACCTTATGTGTGTTGAAAATCTGGTTTCTAATGTAATTATGAGTTAGCACGAATGTTGTGCAATGTTTTTGTT

>PvRGL191

CACTGAAGCAGTCGCTCACAGACCCACAAGGGTCCATGAGTAACTGGAATTTCTCTGATGAAAACCCTTGTTCATGGAATGGGATCACTTGCAAGGACCAAAATGTTGTGGCCATCAGCATCCCGAAGAGGAAACTCTACGGCTCTCTCCCTTCTTCTCTGGGATCTCTCTCCCAGCTCCGCCATGTCAACTTCAGGAACAACAAGCTTTTTGGAAACTTGCCTGCACAGCTCTTTCAAGCTCAGGGACTGCAAAGCTTAGTGCTTTATGGCAATTCATTTTCTGGGTCTGTTTCTAGTGAGATTCAGAATCTCAGGTACCTTCAAACTCTAGATTTATCACAAAATTTCTTTAATGGGTCATTGCCTGCTGCAATTGTCCAGTGCAAGAGACTCAAGGCCCTTGTTCTCAGTCAGAACAATTTCACTGGCCCCTTACCAGATGGGCTTGGTACTGGTTTGTTTTCTCTGGAAAGACTAGACCTTTCTTTCAATCACTTCAATGGGTCAATTCCCAGTGATTTGGGAAATCTGTCAAGCCTGCAAGGAACTGTTGACTTGTCTCATAATCATTTTACTGGTTCAATTCCAGCTAGCCTTGGTAATCTGCCTGAGAAGGTTTACATTGATCTTACTTTCAACAATTTAAATGGTCCAATTCCTCAAAATGGTGCTTTGATGAACAGAGGACCAACTGCCTTTATTGGCAATC

>PvRGL192

CCTAACAACAGGGTCAGTACCCACACACAACAAACCCAAATGAAGCATCCTTTCAGCTTCCTCAGCATTATAACCACTTTGACCCTTCAAACGCTCATCAATAGCACAACTCAACTCCCCTTTCTCTATAAGGGAAAACACCCAATCAACCAATGGTGGCTGATTTGCTATGATGGGTCTTCGCCCACACACCACTTCTAGAACCAATACTCCAAAGCTAAACACATCACACGCAGCTGTTGGTCGCCCAATTCGAACTAGTTCCGGTGCCATGTACCCCAGAGTTCCTATCACTTTTGTTGTGTCAGCCAAATGTTCCTGGTGATGCAGCCTTGCAAGCCCGAAATCCCCCAATCTAGCATTCATGTTCTTGTCAAGTAGTACATTACAAGCTTTGATGTCCCTATGCAAGACCTCAACATCCCAACCCTCATGTAGGTATAAAATCCCATCAGCTACATTTTGCAAAACTCTAATTCTTTCTTCCCAGCTCAGCAGCATTGCATCTTCACACTCAAAGATCCTTTTGTCCAAGCTTTCATTCACCATGTAATCATAAACCAGTATAAGTTTTCCTCCTTTTCTT

>PvRGL193

AAGTAATTCCCATATAGCACCAGATAAGTCAAGCTTGATAAAAGGCCTATCTCGTGTGGTATGCTTCCAATGAAGTTGTTTAACCCAAAGCTAATTCGAGACAAAGAAGAAAGGTTTCCAATCCAAGTTGGGATTGTTCCTGTAAGATTGTTAGCTCCTGCAGCTAGTACTTTCAGTTTTATGCAGTGACTGAGATTACTTGGAATACTCCCACCAAAGTTATTATAGCTGAAGTTTAGATGTTGTAGGTAGAGTAAACGACCCACCTCATGAGGAAATTCACCATGGAAGCTATTGTTTAACAAGTTGAGTCTGTTGAGGAATGTGAGGTTTCCTATGAATGGTGTAAGAGTGCCTCCTAGTCTCAGTTGCTCAAGGCTAAGGTCCATGATCCTTCCATTGGAGATGTTGCATGTGATTCCTATCCAACTGCAGTGATGGATGGAGTCATTCCAAGAGCTCATAATTTTGAATGGATCTTCTACTATCCTACTCTTGAAGTCAAGTAAGGCTTGCAAATCAGTTTCATTTCCTGCAGGAGTTG

>PvRGL194

TCAGAAAAGAGAGAGAAGCTTGATTGGAAACGTAGGTTGGGCATAATCACAGGAGTTGCAAAAGGGTTGCTTTATCTGCACGAAGACTCACACAATTGCATCATCCATCGTGACATCAAGGCCAGTAACATCTTGCTTGATGACAAATGGACGCCCAAGATTGCAGATTTTGGCATGGCTCGTCTCTTCCCCGAAGATCAGTCCCAAGTCAACACACGTGTGGCTGGAACCAATGGATACATGGCTCCGGAATATGTTATGCATGGAAATCTATCGGTGAAGGCAGATGTATTTAGTTATGGAGTTTTGGTATTGGAGTTGATCACCGGTCAAAGAAACTCCTCTTTTAATTTGGATGTGGACGCGCAGAACATGCTTGATTGGGCATACAAGATGTACAAGAAAGGAAAGAGCTTAGAAATTGTGGATTCGACGATAGCAGGTACAATGGTAGATGAAGAGGTAGCCATGTGCATTCAGATGGGTTTGTTATGCACTCAAGGAGATCCACAGCTACGTCCCACGATGAGGCGTGTGGTGGTGATGCTGTCGAGGAAGCCAGGGCAGATGCAAGAACCAAGCAGACCAGGAGTGCCTGGTTCTAGATACAGAAGACCT

>PvRGL195

GCTATAAACATCTGATTTTCTAGTCACTTGATTTCGGATTGCATATTCAGGTGCTAGATAACCAGCTGTTCCAGCAACACGTGTACTGATATGGGTCAGGTTTGGTGGAATAAGCTTTGCAAGCCCAAAATCTGAAATTTTTGGCTGGAGATCTTTGTCGAGTAGTACATTGCTTGCTTTTATGTCTCTGTGGATAATTTGTGGCCGAACCTCTTCATGAAGGAATGCAAGGCCCC

>PvRGL196

TGCATCTTAAATATGAAAGTTGAAAATAAAATACAAAACATACACATGAAAACTCTTAAATCCACCACTACACCAAACACTTGCCATCGACAGCAAGAACTGGAACAAAAGAGGATGCTGAGGTATCTGCACTGTACCAACAAACTGTCTCTAAAGAAAATGAACAACTAGGAGCAAGGTTTTGACCACAAAACCACAATTCTAGCTGCATCACCAACTCTAACATGGTCTCCAAAACCACTACAATGCAAACACATTTGTCTACAGTTTTCTGCAATGTTAAGGTTTCGAAGACTCAAAACTGCAACAGCAATTGCAATTTAAAACCTTGAGGAGACTAGAAATGAGACCAATGATTTGAATATGTACTTCACCAACTTAAAACTAGAAAATGGAAAGCAAGGTGCATTCAAGAAATCAACAAACAAATGGTTTCAGCGGCCATCAACAGAAGTGAAAGACTCTGCAAACCCAGTAGGCCTTGCAGGGATACTACTCTTACTGTCCTGCGGGTCCACATAGCTCAACTCCTTTCCTTCTGTCTCTTGCCAATCCTTCACCATTTGGTTAAGGGGAAGAGTGTAGTCTATGCCAGAATACTCCTCGGTGTCATCATCTAATGGTTTCCATTTCTCAACAAGCGGTCCAAGAACATTCACAGCATGACTCATATCTGGTCTTTGGTATGGTTCTCTGGCAGTGCAGTGCCCAGCTAACTCAGCAATGATGGAGACAATGTCGAACATTTCTTCCTTTATGTCCAGGGAAGGGTCAACAGCAGCCATTAGTTTCTCCTTGTCTGATTTTACATGCCAGAACCAAGATGCCAGGTACTGGCTTTCCTCAGGTCTATCCTCATCAAGTGCCATTAATCCAGTTAGCAGCTCCATTAGCACAACACCGAAACTGAAAACATCTGCTTTGGTGGTGACTTTGCCTGTTACTGCATATTCTGGTGCCAAGTATCCAAATGTACCAGCAAGCCGGGTCACTACAGATTTTTTGCCATCAGGAGCAAGTTTTACCAATCCAAAATCCGAGACTTTTGCTCTAAAATCATCCCCAAGCAAAATATTTGATGATTTAAGATCTCTGTGGATGAAGATCTGGTGTGCCAGACTG

>PvRGL197

GCTGCTTCCAATCCAGCACCNTTTGCCTCAATGTCATTCGAAATCTTGCTTGAAAGGCGTCGAAATGCTTCCCCGAAGTCCCCTCTTTGATGACGTACTTCAGAGGGATCAACGTGATAGAACACTGGTAGCACCACATGCCCTATGGTTCTGTGACACTCCATTATTTTCTCCAACTCTTTCAAACACCACCACGACTCTGCATAATTTTTGGAGAAAACAACAACAGAAATTCGAGACTGTTCGATCGCCAGCTTCAGCGAGGGTGAAATTTGCCTTCCGCGGGGAAGTGA

>PvRGL198

CATATGCCAGCAGTGTCGGGGCAAGTTCAGTTTGGTAAAAACCATTGCTTCTTTTTCCGCTGATGATTTCAAGCAAGACAACGCCAAAGCTGAACACATCGGATTTCACACTGAACAATCCTTCCATTGCATACTCTGGAGCCATATATCCATATGTTCCCACCACTCTTTTGGTGTTTGCAGCATTCTGGTTCTGAGAAAATATTCTGGCCATTCCAAAATCTGATATTTTGGCCAATAGTTCGTGATCTAACAAGACATTGTTGGGCTTTAAGTCTCTGTGAATGATCTTAAGGCGTGACTCCTCGTGGAGATAAAGTAGTCCTCTGGCTATGCCATTGATAATACCA

>PvRGL199

AATACTCTGGGTCCAAGTAGCCCATTGTTCCTTTGACTTGTGTGGTGATATAACCTTTTGCACCATCACCTAAAGGTTTGGAGAGACCAAAATCAGATACTTTAGCATTCAAGCGTTCATCCAGTAATATGTTAGTGGATTTGATGTCCCTGTGTATGATTGGAGGATTGGCAAGTTCATGCAGATAGTCCAGACCCCTGGCAGCACCAAGGGCTATTTTCAACCTTCTAATCCAATCTAA

>PvRGL200

ATTTCAAATCTTCAAATGTATTACTGGATAAAGACTTTAAGCCAAAGCTTTCAGATTTTGGACTTGCTAGGGAGGGGCCAGTAGCTGGTGATACTCATGTTTCAACAGCTGTGATGGGGACATATGGTTATGCTGCTCCAGATTACATTGAGACAGGTCATCTCACAGCCAAGAGTGATGTGTGGAGCTTTGGGGTGGTGCTATATGAAATGCTTACTGGAAGGCG

>PvRGL201

GAATTCCCTCAAATCTTCTTTTGTCAGTTTCTTTAAAGAAAATGAAATTGAAACAAGGATAGAACAAGTCATTGAAGACTTAGAAGATCTTGCAACCCACATCCATGTTCTAGGTTTGAAAAAGGCTGATACTGTTGGGGTCAGATCCGGTAGTAAATTATCATCAACATATTTGCCAAACGAAAGTGNGATCTATGGCAGAGAAATGACAAACAATTTGTCTTTAACTGGCTCACATCCGACACTCACAACAACCTATCAATACTTTCTATTGTGGGAATGGGAGGAGTGGGTAAGACCACTCTTGCTCAACATGTATTCAATGACCCAAGGATGGATGAAACTAAATTTGATGTCAAAGCTTGGGTTTGTGTTTCAGATGAATTTGATGTTTTCAAGGTATCAAAAGCAATTCTTGAGCATGTTACTAAACCCACTGATGATGACACTAGAGATCTGGAGATGGTTCACAGAAGATTGAAAAAAAGAATTGAAAGGGAAGAAATTTTTTCTTGTTTTGGATGACGTTTGGAACGAAAATCAACCTAAATGGGAGGAAGTGCGAAAGCCCCTTGTTTTCGGAGCCCAAGGGAGTAGGATTCTAGTGACTACACGTAGCAAGGAAGTTGCTTCTACCATGCGGTCTGAGGAACACTCCCTAGAGAAACTACAAGAAAATGATTGTTGGAAGTTGTTTGCTAAACACGCATTTCAAGATGATGATACTCAACCCAATCAAGAGTGCAGGGAGATTGGCATGAAGATTGTTAAAAAATGTAAAGGACTACCTCTTGCCTTAAAAACAATGGGAAGCATGTTATATAACAAATCGTCTGTTTCAGAGTGGCAAACTGTGTTCCAAAGCGAAATATGGGAATTTTCAAAAGAGCGTTGTGATATTATTCCCGCTTTAGCA

>PvRGL202

GTTTACTTTGCAAAATTGAGTGATGGTACTGAGGCTGCAATTAAGAAGCTGGATAACAGTTCTTCCCCAGAACCTGACTCCGATTTCGGGACTCAGTTATCAGTTGTTTCAAGACTGAAGCATGACAACTTTGTTGAGTTGATTGGATATTGTCTAGAGGCAGAAAACAGAATTTTGGTTTACCAACATGCAGGCCTTGGATCTTTGCATGACGTGTTACATGGAAGGAAGGGGGTACAAGGGGCTGAACCTGGTCCAGTTTTAAGCTGGAACCAGAGAGCCAAAATTGCATTCGGTGCTGCAAAAGGACTTGAGTTTCTTCACGAAAAGGTTCAGCCTTCTATTGTTCATCGCGATGTTAGATCCAGCAATGTCTTACTGTTCAATGACTATGAGGCCAAGATAGCCGATTTTAGCTTGACAAATCAGTCTTCAGACACAGCCGCTCGGCTACATTCAACAAGAGTCTTGGGAACATTTGGCTATCATGCTCCAGAGTATGCCATGACAGGGCAGATAACCCAGAAAAGTGATGTTTACAGTTTTGGTGTTGTGCTATTAGAACTCTTGACTGGAAGAAAGCCAGTAGATCACACAATGCCGAAAGGACAACAAAGTCTTGTAACTTGGGCAACTCCAAGGCTGAGCGAGGACAAAGTGAAACAGTGTGTTGATCCTAAGCTAAACAATGAGTATCCACCAAAAGCTATTGCAAAGTTGGCAGCTGTGGCAGCACTTTGTGTTCAGTATGAAGCAGACTTCAGACCAAACATGACGATTGTTGTCAAAGCTCTCCAGCCACTTCTCAATGCAAAACCCCCTGGTCCTGAAACTCATGCTTGACATTGTGATCTCATTTAACTATGAGGGTTATTAGATTCTGTGCACAATTTGTAACACAATGGTGAAGTTGATTTGCTTTCATTGTCATATATGATTGTATTATGCTATGCCAAAGTTGATTTGGTTTGCTTGCTCTTCCATTTATTTGGAAACATATGTCTGAACACATAATGAATGATGGAGACCAAAATTTGGCATATCA

>PvRGL203

TGAATGTGTCCACAAGCAGACAAACAAAATTGATAGATTGTGAAGTTCGTATTTACAAGCCATGCTCTTCTCACTTCACAAGATAATGCAATACAACAAAATGCATGGTGTTTAAACCGTATAAGATATGCACAGCGACTCATAGAACCCGTTCATAACGTCATGAAATCTGAATACAAATAATTCGATCACCAAAGTTGCTTCACCAACAATACAAACAATCATCTCCATCTTGATTTTTCTATGATTTCTGCATGCTTTGGTGGTGGATAAGGAACTTTAATGGAAACATCTGCATGAGAAGGCAAAGGATCTTTGTCTCTCACTGTGCGTAGCTCAGAACGAAAGGGGAAATCATCTGTTTCGAGCATATGAACAATTTGACCCATCTTTGGTCGCTTAACCACATCCAAGTCTATACACCGCAGACAAATAAGTAAAACTCGTTTCAAAGATCTTGGAGAGGGTGGAATCTCAATCAAAGGATCAACTAGCTCCTCACTGCGACGACTTGCTACCATTGTCTTGAACCAATCTACTAAATTCATCTCTCCAGGTGGTTTTGAATAATCAATGGGGCTTCTTCCTGTGATTATCTCCATGAGTAGAACTCCAAAGCTATACACATCACTGCGCTCATTAAGCATACCTGAGCTTGCATACTCAGGTGCAACATATCCGAATGTTCCCATCACACGCGTAGTCACATGGGTTTTCTCAGATCCTAAGAGTTTGGCAAGTCCAAAATCTGAGACTTTGGCATTCCAATTTTTATCCAAGAGAATGTTACTGGATTTTACATCCCGGTGCACAACTTTGGGTTCTAAGCCTTCATGCAAATAGGCTAGCCCTTTTGCTGTCCCAGTAGCAATTCTCATTCGAATCTCCCATGTCAAGGGGCTAACTGGTCCTACATCACCATGCAGCCACTGTTCCAAGTTTCCGTTGTCAACATACTCATAAACTAGCATCCTTCGAGCACCTTCTGCGCAATATCCTATCAAACGAACCAAATTCTTATGCCTTACTTTTCCAATAGCTTCAACCTCTACCTTAAACTCCTTCTCTGCTTGACCCTTGTTGTTGAGAAGATTCTTAACAGCAACGACGGAAGCATCGTGCATAATTCCTCTGTACACAACGCCGTACCCACCTTCCCCAATAACATTCCCTTCTGCGAAACCGCGCGTGGCGAGTTCCACTTCCTTCATGCTGTACCAC

>PvRGL204

CGGGTGTATGAGATATGAAAATAAAATTGCATCTGGACCTTTCAGTGATTTGTATAAAGGTACATTCTGCAATCAAGATGTAGCTATCAAGGTTCTAAAGCACGATAATCTGAATGAAAATATACAAAGGGAGTTTGCTCAGGAAGTGTATATCCTAAGTAAAATTCAGCACAGAAATGTTGTTAAATTTGTTGGCGCTTGTACAAAACCCCCAAACCTGTACCTGGTCACAGAATACATGCCTGGTGGAAGTATGTTCGACTTTTTGCATAAACAGAAGACAGCCCTTACTCTTCCATCTTTACTCAAAGTGGCCATTGATGTTTCCGAAGGAATGAAATATCTGCATCAGAATAATATCATACATCGGGACCTTAAAGCGTCTAATCTTTTGATAGATGAGAATGGGGTAGTTAAGGTTGCTGATTTTGGTGTGGCTAGAGTGCACAATCAGTCTGGCATAATGACTGCAGAAACAGGAACTTATCGGTGGATGGCTCCAGAGG

>PvRGL205

AAAAGTCATAGGCTCTGATTTAGCTGCTTTTCATGAAAGTTTAGAAGATTGGGAAATGGCAATGATAGAATATGAGAAGACTCCAAATAAAAAGATTCAAGATGTGCTTAAAATAAGCTACGATAGATTGGATGATAATGCCCAACAAATTTTCTTGGACATAGCTTGCTTCTTTAAAGGGGAGAGAATGGAATATGTGAAAAAGATACTTCAAGAATTTTGTCCAACCACCAATATGAAAGTACTTCTTAATAAATCTCTCATAACTGTCGATAATGGCTGCCTGAAGATGCATGATCTAATACAAGATATGGGTAGAGAGATTGTTAAGCAGGAATCACCAAATCCTGCTAACCGTAGCAGAATATGGTATTATGAAGATGCTATTGAAATACTAACCAAAGATTATGGAAGTGATATGATTCAAGGGTTTATGCTTGATCCCCCTCAACAGAAAAAGTAGAATGGAGTGGTACTGCGTTGGTGAAGATGGAATGTCTCAGAATTCTCATTGTTCGAAACACATCATTTTCATCTGAACTTGAACAATTACCAAATTATTTAAGACTTCTTGACTGGGAAGAGTGTCCTTCAAAGTCTTTCCCACCAAAATTTAATCAAAAGCACATCATTGTTTTAAATTTGCCTAGAAGTCGTCTAACATTGGAAGAGCCATTTAAGGGTGTCGCAAATTCAGAATTGAAGCAGAACGCATCTTGATCTTTGATCTACGAGTTTTGTTGAGTGACAAGGAGTGGCTTGGCCATGACCGACTTCTGGAGGAGGAGTGAAATCTGGTGCAGATTGCGTACAAAACTTTTGTGTATGAATCTTATTTTTAAGTTATTTTGGAATAGATTTGAAATGTTAAAGTAATAAGTGTCTAAGATTTCA

>PvRGL206

GGAAGGGAAGTTTCTCTTGTGTGTTCAAAGGGGTTCTCAAAGATGGGAGTGTTGTTGCTGTTAAAAGGGCCATAATGTATCCCAACGTGCAGAAGAATTCCAAGGAGTTTCACACTGAGCTTGACCTGCTTTCTAGGTTGAACCATGCACACTTACTCAATCTACTAGGCTACTGTGAAGAGGGTGGAGAGAGACTCCTGGTTTATGAGTACATGGCTCATGGCTCATTGCATCAACACCTTCATGGGAACAAAGTGATGAAAGAGCAAATGGATTGGGTGAGAAGGGTAACAATTGCAGTCCAAGCAGCTCGAGGAATCGAATATTTGCACGGCTATGCTTGTCCACCAGTGATTCATAGAGACATAAAGTCTTCAAACATCCTCATTGATGAAGAACACAATGCCAGAGTGGCTGATTTTGGTTTGTCACTACTTGGTCCTGCAGATAGTAGCTCCCCACTGGCCGAACTACCAGCTGGGACTCTTGGCTATCTTGATCCTGAATACTACAGGCTTCACTACCTTACCACAAAGTCTGATGTATACAGCTTTGGAGTTCTACTTTTGGAGATATTGAGCGGCAGAAAAGCTATTGACATGCAATATGAAGAAGGTAACATTGTTCAATGGGCAGTGCCTCTGATCAAGTCAGGAGATATAGCTTCAATCTTAGATCCAGTTTTGAAGCCTCCTAATGATGTTGATGCATTGAGAAGAATAGCTAATGTGGCTTGTAAAAGTGTGAGAATGAGAGGGAAGGATAGGCCTTCAATGGACAAAGTGACAACAGTTTTGGAGAGAGCTCTTGCACAGTTGATGGGGAGTCCTTGCATTGAGCAGCCAATTTTGCCAACTGAGGTGGTTTTAGGAAGCAACAGACTGCACAAAAAATCATCTCAAAGGTCTTCAAACAGGTCAGCCTCAGAAAGCACTGATGTGGAGGATCAGAGGTTTGAGTTTAGAGCACCCTCATGGATCACTTTCCCCAGTGTGACTTCTTCTCAAAGAAGATCAGGCTCTGAGGCTGATGTTGAAGGAAAAAATATAGAAGTGAGGAATTTGGGCAATGTTGGAGGTGGTGGTGATGCTCTAAAAAGTCTTGAGGAAGAGATTGGTCTTGCCTCTCCTCGAGAGAAACTCTTCTTGCAACACAACTTCTAACCTTTTTGATTTGTTTTGTTTTGTAATTGAGTTTCATCATGAATTGTTCAGTTCCCATTTTCATGGAGTTGTCATTTGTATTCAATTCTGTGATAATATTGGTTCCTTTTTCATTAGATACAGAATCTGTCAAACCATAATTTCA

>PvRGL207

GGAGCAGAAAAGGATATCGATGCAAATGGTTCTGATTTGACAATATTCAGCTATCTCACTATTAAAGTGGCAACAAATAACTTCTCAAAAGATAATAGGCTTGGAGAGGGTGGATTTGGCACAGTGTACAAGGGCAAATTACGGAAGGGTCAGGAAATAGCTGTGAAAAGACTCTCAGAAACCTCAAATCAAGGCCCTGAAGAGTTCAAGAATGAAATCGCACTTACAGCAAGACTACAACATGTGAATTTAGTTAGACTTCTGGGTTACTGCACAAAAAGGGATGAGAAGATTCTGATCTATGAATACCTGCAAAACAAAAGCTTAGATAATTTCCTCTTTGATCCAACGAAATCAATTCTTTTAGACTGGAACAAGCGTGTGAATATCATTGAAGGCGTTACTCAAGGCCTTCTTTATCTCCAAGAGTACTCAAATTTTACTATAATTCACCGAGATCTTAAAGCTAGTAATGTTTTGCTAGATGAAAATATGAATCCAAAGATATCCGATTTTGGTATGGCTAGAATTTTCAGAAAGTATGACCTTGAAGCAAACACAGAAAGGATAGTTGGAACATATGGGTATGTACCTCCTGAGTACGTGAGAAAAGGCATATACTCAACCAAGTACGATGTTTATAGTTTTGGAGTTCTTCTCTTGCAAATCATAAGCGGAAAGAGGACTTCATGTTATTATGGCATGCATGAAAATATGACCCTTTTGGAATATGCATATGGGTTGTGGAGGGAAGGAAGAGGTGTGGAGTTTGTTGACCCTTCATTGGATGATACAGCATCTCCTTGTAAAATCATGAGATGCATGCAGGTAGCTCTGCTGTGCGTTCAAGAGAACTCTGCAGATAGACCTTCCATGTTGGAAGTTGATTCACTGCTGAAGAATGATGGTGCAGTCATTGGTACTCCCAAAATGCCTGCCTTTTCAGTTCAGAAACGTGGAGATGAGGAAGAAACATCTCACAGCGGGATCAAATTTTATTCAATCAATGATGTTACAATTTCTCAACTGGCACCAAGATGATGGAGTAGGACATTCTAGTACTTCTCAAGCGTGTAACTTTTGTTCGACTCTGTTAAGCCTACCAGGTTTCATCACTAATTCTAAACTCACAAAGTTATAGTGACGATTAATTTTTGTTTTTAAAATTACATGCATATGATTTTGTAAGTAATTTTAGTGATGTTCTTACACGTTCTGGTAAGGTATCGAAGGTCTTCTATCGTCAATTCTTGATGGGTCTAGTCTTTGCCTTTGGTCCTCAGTCTTAGTCAAAGATGATGGATACCAGGAAAAGACACTACGCTTCAAGAATCAATATACGGTAATTGAATG

>PvRGL208

CCAATATTTAACTATTAATCATTAAGATCATAAAAAATGCCTAATATCTGTCCCAGTTGCAGAAACATGATGATGATTTCTGAAGCAATAAATTAATTAGGTCCTCCTCAAATTCATTTCTAAGCAAAAAAACATTAACAAGTACTTCACTAAATAAGGTTGATTAACCCTTCTGTTTTGTAATCAACAGATTCAAGTTGATTAACCACAATCATTTCAATGTAGGTCTTTCCAAAGAACCAAAACCATTGCAATGTTAAACTACTAAGGTTGCACATTTGTCATTTACTACAACAAATTTACAGAGCCACGGAATGGGGCCAGGAACAGAACGCAAAGGTTGTTAGCATAGCTTCTTGAAGTTGGTTGAGTCACTTATCAGTGATCTGAGTGTGACTCATAAAAGTCACTTGGGCAAGGAGACATAACCTCTTGTTCTAGTATCTGTAACACTTGGTTCATTGTTGGACGTTCATCAGCATTTGCATCAGTGCAACTTGCTGCCATTTCAAGAATTACTTCCACAGATTCTGAATCTGCATCACTGCATCTTTTGTCTACTACCTCCTCCAATCTATTTTCTTTCAGGAACGCGTTCATCCAACCAACAACATTTACACCTCTGCTTGCAAATGTAGGATCTGTAGGTCTCTTTCCAGTTACAAGTTCTAGCAATAGAACTCCAAAACTATACACATCTGACTTCTCAGTGGCTCTTCCACTTTGTAGATACTCTGGTGCCAGATAACCAAAGGTGCCAGCAACCACTGTTGTAACATGAGCATCTTCATCAACCAAAAGCTTTGCAAGACCAAAATCAGAGACACGAGGGTCCATGTTCTCATCAAGAAGGATGTTGCTGGATTTTATGTCGCGGTGCACAATTTTCGGGGAGCAGTCGTGATGCAAGTAAGCCAAGCCCCGGGCAGAACCAAGCGCTATCTTAAGGCGAGTACTCCAATTCAGTGATTGTTCAGTGTTTTCATGCAAGAGATCATCTAAGCTTCCCATGGCCAAATAATCATAGATTAGAAGCTTAGTAGAAGGAAGTCTGCAGTAACCACGCAGGTTAACTAGATTTATGTGCTTGATGCTGCCCAATATCTCTAGTTCCCTCTCAAAGCCCTGATCAGAACCTTCCCGGCTTCT

>PvRGL209

AGACAAAGTGGAAACTATTACCATCTCTTTAAACGAAAGATGAAACATAAAACAGCGGATTGCATGAAGTGACCTACTTACTCAATTAGTTTTTACACCTGAAACGCTTTACACATAGGCAACAAATTGCATATGCTTTCACTTGAATGAAACGAGAGCTTAAATGAGATTAAAAACAGAGTAGAAACCAAAGGCAGGCAATTAAAATCACCTGGTCTGCTCATTAATCCAAACAGTTGAAAAAAGTATTGACTTTGCAGGATTTCCAGTTGGCCCAATCACTCCATAAATGCCGCAGCAAACCAGATGGTAGTGGTAAATAAATCATACGAATTTCTGAACAAATTAATGCTATTAGACATTTATGATGCAAACAAATGCCAGTGATTATCTGAAGGTAAATTCTCCTTGATCTTTTCAAGACGTTTAAGGATCTCTATGAAGGGTGGTCTCCGATTCACGTCATGAGCCCAACACTGCTCCGTTAATTCTTGCAATTCATTGGTATAACCCTTTGACCGAAATGAAGGCCTATGTCCCTCAGCAGCGCGTTTTGCTCCATCATAAGGCTCGAGATTTGCAAAAGGTGGTTCACCTTCAAGCATCTCATACAAAATCATTGCAAAGGAGTACACATCAACCTTCTTATCATATCGCCGATGTTTGAAAACTTCAGGAGCCATGTAGCGGTAGCTCCCAGTTTCACCGGTCATCTTGTATACATCATGAGAACTCTGGACAGTGATAAGCTTACTCAATCCAAAATCTCCAACTTTTAAATGGTCGGCACTAGAATTGACTAAAAGAACATTCCTTGGCTTTAGGTCCCGATGAATTATAACATTTGGTTCATTGTGGAGATAGGCCATTCCTCTCACAATATCCATGGAAAAGTTGATAGCTGTTGCTGGACTAAGTGCACCTTTTTCTTTGAGGTACTGATGAAGATCACCCCCTCTTAGATACTCAGTAATTAACATAAGGGGCTTCCTGTCTGTAACAGCTCCAAGAAACTGAACTATATTGGGGTGTCGAAGCTTCACTAACAAATTGACCTCATGCCTGAAGTCCTGAATCACCAATCTATCTTCTGAAAGAGATGGAAGAATACGTTTAACAGCGACTGGTGTTCCA

>PvRGL210

CGATAGAATGTCAGCGGTGTATTCCAGAATCTCGTGTATTACACATGACTATTGTCCAATGATAATTTCATGCAGAAACTAAAAACAGAAATAATCGGAGTAATTGCCTTGGGGCTAAAGAACTGGAGGGGGGAGTAAACAAAATGCACAGAGAGGGGTATAATTGAGCTTTGATTCATACAATTTAACCTTTATTCATAAAATTAGTATTGATTATGCTGTCCCTTTTTACCATTATCAACCATCTTTCACTTGCCATGTAGCAAGAGGATTCTAAATAGAGGCACAAATCCTCCTACCTCCTGTGAATCTTCTAAAGAAATGCCAAAGCATGCTGAAAATGAATACCCCGGGCTAACTACAACATAATATTTATGTAGCATGTAACAAACACACAACTTTAAGCATTTGCAGCATTTGGAAAATCAATCCATTGTTGCAGGCTTAAGAATGGTCCCTTCAACATTTATTCATTCATCTCCCACATAAGCATTCACCAACATTATGGTTTCGCCTAAAAAGTACAAGAATATGTGTTGCAAAGTCCTGTGTTGGCGCTATTATAGGGGAACAAGGAAACAGGACGAGTTCTTGGCAGTTCAGGAATGGGCTTATCCATGTTCAGAAGAAACTCCACCACTTGCTCCATTGAGGGTCTTCCTTTGTTCTCATTTAGGGTGCAAAGCAACCCAATTTCTAGAACACGAATTGCCTGGTCCAAGTTGATCAATGATCCCATCCTTCTGTCCACCAGCTTAACTTTCTCATCAATCTGATGCAAGTTCCAAGCATAATCCAATAGGTTCCTCTCTTCTGGATTTCCCACCTCTTTCTCATCCCTTACCCTTCCAGCTATCACTTCCAACACAAGGACACCAAACTCAAACACATCTGCGCCATGGCACACTGCTGACTCAAACTGTTTTGATTCAGCCCCCAACAGCACAAACCCAAAATCACCCAAAACAGCTCTGAAGTTGACATCTAGAAACACACTGCTGCATTTCAAGTTCTTGTGGGCTAGTAGCTTAGTGTGGAGGAAGCTCAAGCCATCAGCTACATCCTTAATAACTTTGAACCTCCTAGTCCATGGAAGAACACCAGCCCCAAATAACCATTTGTCCAGGCTGCCATTGGGAACGAAGTCATAGACCACCATGACTTCATGGTTGTCTTGACACCATCCTCTAACAGGAAGCAAATTCGGGTGGCGAACATGGCCAATGATTTTGATTTCCTTCAGCAAACGCTTCTTGTCTGATCCATGTGTGTTGAGAAACTGAGTTGAGAACCTTTTCACAGCCACCTGGCTTCCATTAGAAAGCTTTCCTCTGTAGTATTCTCCTCTAGAATCA

>PvRGL211

GCAATTCCCTCGGCAGACCCGATTGCGATGATCATCCGCCGGTTCCAATCAAGAAGGGATTCTGCTGAGTGCTGTCCATGAAGATGAGAGAGCAAGCTCAAATTTGGCATATAATTATATACAATTAACCGTTCCTGACCTTCAGCACAATAGCCACGTAGGCTGAGAAGATTCTTGTGACGGACTCTAGCCAATATCTCAACTTCAACAGCAAATTCCATGTCTGCTTTGTTGCTCCAAACTTTCAATCTTTTCACTGCAATCTGCGATCCATCCCAAAGCTGGCCCCAGTACACACTGCCAAATCCCCCTTCCCCTAGCTTGTTATCATAGTTGAAATTATTAGTAGCTGAGTGCAATTCCTTCAAAGAAAACACCCGCCAAGGAGGTTGTTTCTTCCCTCTTCCTTTTCGATCCGAACCATTTCCGCAGCAAAAGATTGGGCAAAAAGCCATAACCTTCGCAGAAAGCACCCCCACTGTCTCTGGAACTGAACTTTCTCAATCGACAAGAGAAAACTCAGCAAAATCGAAACTTGAAGTTGACCCACCAAAGCAAATCAAGCGCTCCACTCAGAAAGCCGTTACTTTATCGTGTTACTGGTCACAAACACGTTGTAGAGAAACTGAAGACAAGTTTTTTTTTCTGGGTTGTTTCAGGTTGTGCGAAACTCAAAGCCGAAAAGAACA

>PvRGL212

ACACCCCATTCCCAACATATCCCTTCAAATGCTAAGAAGATCAGGTGGTGAAGACTGAGGAAGATGTTGATTATCTTTAGGTTCCTTAGATGCCGTTGGAGACTCTAAATTGTTTGATGATGACAGAGAGGACTCCGTGATTGTTAAGTCTCCCTGTTTTGAGCTTGCTGACTTTGGAAGCTCAGGTCAGGATTTGAACAACTTCTCGCATTGTTGGTCGCTCCACTGCCTGTTCTTCAACACATAGCATGGCTACATAGAAAACATGCATCACCTCATGGAGTGGAACTGAGGGAAGTCTAGGATCAAGAACCTTTAGAACTCCTTCCTTGTTTGAGTCCGTCATTTTCCTCACCCATTGCACAATGTCCACACCATCACCAAACTCCCCAACTGGTTTCCTGCCTGTTACAAGCTCCAAAAGAACAACTCCAAAGCTGTACACGTCACTTTTCTCGTCAACTTTTAACGTGTAGGCATACTCTGGAGCTATGTATCCATACGAACCGGCAATTGCAGACATGCATTCGGATGTTCCAGAATCTTGCAGGAACTTAGCGAGCCCAAAATCCGCAACATGAGCTTCAAAGTTGGAATCAAGAAGGATATTGTTGGATTTAACATCTCGATGGACAATGAGTGGCGAACAGTCGTGGTGAAGATAGCAAAGGCCCTTCGCGGCCTCCACAGCAATTTTATACCTTGTGTCCCAATGCAAATGACCACCTTTCTTGCCATGAAGAACCTCACCTAAACTTCCATTAGGCATATACTCGTACACCAAGAGATTTGTCTCGTGGTTTGAACAGAAACCCAACAGTCTAACAATGTGTCTGTGTCGAATCCTCCCCAAAGTCTGAATCTCAGCATTGAAACCATGGTCATGCGAAGATCCTCTGCTCATGGCAGGTAACCTTTTCACGGCAACCTGATCCCCATTCGGCATTGCACCTTTGTACACAATGCCTGCACCTCCCTTTCCTATTATGTTGTCCTCCTTCAAGGAATCCAAAACATCATCAGCAGTGAAGTCCAAAC

>PvRGL213

TGCATCAAGGACTTTAAAATTATANTATTATATATATTACATCATGACAAGTTAACTAAATATACAGTTTATTTTCCAGAGTATTTCTGAATGTTGTGTGAGAAAATCCAACACGTAATTAATTAATATCTTAAAATTGTTTGATACATTTTCACACTTTCTACTCCTTCCTTGCTTCCTGTGTTTTATGGTAGTAAATTTTAGCCGGGGCAACTGCACAAAATTGCAAAAGAGTAGAGGCACTAACACTTAAAATGACATGTCTATGGCCTCATGTTCTGGGGTCTTGTGACCAGTGCCAGAGTCGTCACTGGGTCGTCTTTTAACCACACTTGCTCTTTGCACTAGCCGGACCAATGCTTGCACCACCTCAGACATTGGAGGTCGAAATTCAGGTTCCGGCTGAACACAGAGGGCAATGATATCAGCAAAGCGTGACAGTGACTTTGCAGGATACATGCCATTCAAAGTAGGATCCACCATTTTCGCCAAGGCATCTATATCATGGAGTTGGGGTGTAGCCCACCTCACAAGTGACTGCTCTGATCGAATTCGCGAACTGTCTAGTGGCTTCCGACCAGTCAACAATTCCAGCATAACCACCCCAAAGCTGTAAACATCACTTTTTACAGTGTATACTCCTGATAATGCAAACTCAGGAGCACTGTAACCAAATGAACCGACCATCTGAGTTGATACCTGCCTCTCTGTGTTTGGAGTCAAAGCAGCTAAACCACAGTCAGACAGGTGAGGATTTAGCTCCTCATCAAGTAATATATTTGCTGATTTGAAATTTCTATGTACAACAGAAGGCAAGCACACTTCATGCAAATACTCTAAAGC

>PvRGL214

TAACGAGTGAAGATTCGAGCTTGTTGGTGTACGAGTATTTGCCCAATGGAAGCTTGTGGGATCGGCTTCACACAAGTAGGAAGATGGAGCTCGATTGGGAAACGAGGTATGAAATCGCTGTTGGGGCAGCCAAAGGATTGGAGTATTTGCATCACGGGTGCGAACGGCCCGTGATTCATAGAGATGTCAAGTCCAGTAACATCTTGCTGGATGAGTTTTTGAAACCCAGGATTGCGGATTTCGGACTTGCTAAGGTTGTTCAAGCCAACGTTGGAAAGGATTCTTACACTGGTGTCATTGCCGGAACCCACGGTTACATTGCTCCTGAATATGGCTATACATACA

>PvRGL215

TGAAGGATTTTTCCAGCATTGGATCCATCAATTCCAAACATTTTCCTTCATACCACATTCTCCAAGCATATGAAATAAGAGTTTGACCATCTGACAGGTAGAATCCACCATTCTTTCTCCCACAAATGATTTCTAGAACAAGAACTCCAAAGCTGAAAACATCAGATTTCACTGAAAATAGTCCTTCCATAGCATACTCAGGAGCCATGTATCCAAGGGTTCCCATTACTCGTTTTGTATTTGCCTGGTTTTGTCCTACTTCAAATGCCCTTGCCAATCCAAAATCGGATATTTTGGGATTCATGTCATGGTCTAATAGAACGTTGCTTGCTTTGAGATCTCTATGAATTACTCTGAGTTGAGAATCCTCATGAAGGTATAAAATACCTCTTGCTATTCCATTGATAATGCTTAATCTTAGTTTCCAATCGAATTGTTTTCTTCTGTCGACATCAAATAGGTGAAAGTCGAGACTTTTATTCGGCAAATACTCGTATACAAGTATCTTTTCATTTTCCTCTAAGCAACATGCCAAAAGTCTTACAAGGTTGCGATGCCTCAATTTAGCTATAAACATTACTTCATTATTGAACTCCTCTGAGCCTTGACCAGAAAATTTTGATAGTCTTTTTACTGCAATTTGCTTTCCGTCACGTAGAGTTCCCTTGTAAACGGGGCCAAATCCACCTTCCCCTAGTTTAGATGCTTCTGAGAAGTTATCAGTACTCTGTAGAATTGTGATTAATGGTATTCTAGGCAGGTCTGTGTTCCACATTTCCTCTGATTGAATCTTACGAAATGAAGGAAGAGGAAGCCCTTTTATCAGCCTTTTCCTGTGCCAGAAGCAGTATAGGCTCAAA

>PvRGL216

TTGTTCCTGCATTGATAGAGCATGCTCAAGTTGCACCTCCACTTCACCAATTGTTGGTCTCTCATCTGGTTCATACTTCAAGCAAGTTACCATCATATCTACAAACAATTGCCAACAATCTGGTGCAATCTTTCCTTTGATAATCGGATCAATTCTCTCCTCAACAGGATTCTCCAGACATTCTTTTTCAGTCAGGCTTATTAAATAAAACATCCCCCTGGACACAACTTTTAGTAGAAGGAAACCAAATGTGAAAACATCCCATTTAGCTGTGATGGTACCATCCATGATATAGTCCGTAGCCGGGTAGCCCCAAGTACCCAAAATATGATCTACATTGATTTGCTTCTGCTTTGACATAAAACGTGATCCCTGCNCGCTAACACCGAAACCAGCGAGTTTTGGCTCCANGTTGGTCATCCAAAAGGATGGTACTAGGACCGAGGATACAGTGAAAGATGGAACGCTTGGCACCAGTGTGAAGGTAGTGTAGTCCACGTGCTACTCCTATGCAGATCTCTAGCCTCTTCTTCCATGATAGTTCACCATCTTGAAGGTGTTTATCTAGAGATCCATTGGACATGTACTCGTATACAAGAATCTTCTCATTTTGGTGGTTGCAGAATCCTATAAGAGAAACACATCTAGGGTGACGAAGCTGGCATAGCAATTCATTTCCTTGTTGAACGCTTCCCATCCTTGATAATGAAATCGCTTTATTGCGACCGGGTAATCAGAAGCACCATCATGCTGCAGACACCCTTTGTATACTTCACTAAATATTCCACTTCCTATTACTCTCTTCTGATCAAAGTTATTGG

>PvRGL217

CCAAATGGACTGTTGGAAAATGCTACCAGCAATGTGTCCAAATGGAGAAAAGGAAAGCTTCTAGGACGAGGAACATTTGGGCATGTGTATCTGGGATTCAATAGTGAAAATGGGCAGATGTGTGCAATAAAAGAAGTCAAGGTTGTCTCTGATGATCAAACATCAAGAGAGTGCCTCAAACAACTTAACCAGGAGATAAATTTGCTAAATCAACTTTCACACCCTAATATTGTTCAATACTACGGGAGCGAACTGGTAGAAGAATCACTTTCCGTATATTTGGAATTTGTCTCTGGTGGTTCTATCCAAAAGTTACTTCAGGAGTATGGTCCATTTAAGGAACCTGTTATTCAAAATTATACCAGGAAGATTGTCTCTGGACTTGCCTATCTGCATGGAAGAAATACGGTACACAGGGATATTAAAGGGGCAAACATTCTAGTTGATCCTAATGGTGAAATCAAATTGGCTGACTTTGGAATGGCTAAACATATAACTTCTTCTGCCTCAATGCTTTCATTCAAAGGAAGTCCATACTGGATGGCACCTGAGGTTGTGATGAATACAAATGGCTATAGTCTTCCGGTTGATATATGGAGCTTGGGATGCACAATTATTGAAATGGCAACATCTAAACCTCCATGGAGTCAGTATGAAGGGGTAGCTGCCATATTTAAAATTGGCAACAGCAAAGATATGCCAGAAATCCCTGAGCATCTTTCCAGTGATGCAAAAAATTTCATCAAGTTATGCTTACAAAGAGACCCATCAGCTCGCCCGACGGCCCAAAAGTTACTAGACTACCATCCCTTCATTCAAGATCAATCAGCAACAAAAGCTGCAAATGTCAGCATAACTAGAGATGCTTTCCCTTACGTGTCTGATGGAAGCCGGACACCGCCTGTGTTAGAGTCCCATTCGAACCGAACAAGTATAACTTCCCTCGATGGAGATTATGCATCAAAGCCAGCTCTTGCAGCCCCACGTGCGTTAAGAAGTCCAAGAGATAGCACAAGAATGATCACATCTTTACCTGTATCTCCCTCTTCAAGTCCATTGCGACGGTATGGGCCAACGCACCAGAGCTGTTTCTTTTCTCCTCCTCATCCAGCTTATACAATAATGGGACAAAGTAGTTACACTTTGAATGACGCATGCTCGTTTCCCGTAAGGTCAAATGCCACGTTGACTCTTGATCCTCGGCATGAAACATCTCGGTACAAAGCCAATACACCACCTGGTGGATCTCCAAGAATGAGACTCATTTGAGTTGAATTTGTAAATAGCTACAGAACTAAAATATCCGTCTCCCCAGTCGTTCGTGGATGGACTTCGATATGGACTCTGAAGCACAAACTATCCAAAAGATTCTGGTCCATTCTTTATACATGAAAACCAGATGACAGAAAAAATGATTCTGGCCGTAAGCTCTTGGTGCATTGGATTGGGGTGGGGAGACTTTGCTGCTTGTAACAATTTCTTGATTATTGTAAATGCGATGTCTTACTATGTTTGTAGTAACTTTATGTTGTATAATGCTAGTTCATGGCAGTATAAAAAAATAAAAAAA

>PvRGL218

GGGATACGACCCCAACAATGCTGGGCATGGTTATAGGGGGTCTGGCGATGATAAAAGGAACAGAGATGATAAAGGTGGAAGAATACTGAAAAATGATGAAGCTGGGGGATCTGGACGCAGATGGGACTTGGAAGGATCCGAGAAAGATGACTCCCCTCGAGAAACTGCAAGAATGTTAAACAGAGATCTAGATAGAGAACGTGCTGTGGCTGAAGCCAAGCTATGGGGAGAGAACTTGAGACAAAAAAGACAACAAAGTTTGCAGGAGGGCACTTATGGTTCTAAGTAAGGCATAGTTTCCCAGCTCAGTCTTTTACAACTTAGGTTGATTTTCTGCTTTCAGGCTATGGAATGTGGAAAAGAATGGGGAGCTTACCTGGGGTAACTCGACCACCCAATTTAGGCTAAAAGATTAGGTTCTAATTTGTTCTGAATCTGCCTAGTAGCCAATACTTACAATAGCTACAACATGTATGAACCTCTTTACAAAATCTTTTCTAACATAGCCTTTTGGCTCTGTGTTTTCCCTAATTTATTTGCTGTACTTTCTGTACAGTTT

>PvRGL219

TGATTTGAAAGTTACTAGAGGCTTGATTGATTTTAAGTGGGCATACGAAATGGGTTAAGCATGATCAACCGATCCATATACAATTGCAACAAACAATCAAAGGCCTTCATGGCATCATCAATAGGTTAATAAATTTCATCAACAAAAAATCCCTATATATGTTATTTCCCACCCATTTTAATCGCATCCGCTGGTGACTTGAGTAGTTTCTTTAGAGATTCAACTATGCTTGAAAATGATGGGCGATCACCAGGTTTATCAGCCCAACAAGATTCCATCAGAGAGGCCAACGCTGGGGAAATGTTTGGAGGGATAGCAAGCCTCCTATTCTGGAAGGCCACAGCTCCCACTACCTGGGCATGACTAAGTCCATTCCACGGTTGTTGCAGGGTCACAAGTTCCCACAGGATAACCCCAAAACTGTAAACATCAGACTTCTCATTTGAAGGTTCGCCACGAAGAAATTCTGGAGCCATCCACTCAGGAGTTCCAGCGACAGATTTTGATGATAAGAAAGTGTTCGCCTTAAATCTGGACAATCCAAAATCACACACCTTGACAGTCCAATTTCTGTCCACTAACAAGTTCGGGGTTTTGAGATCCCAGTGCACAATTGGAGGCTTCAGACAGTGGAGATAATTGATCCCTTTAGCCACGTCTAAAGCCATCCGTAACCTTCTCCTTGGATCTAGAATTTCACCAGATGCTGGCTTATGTATTAGGCGGAATAAACTGCCCCTAGGCAAATATTCTGTTACTATAGATAGATGTGGACGTTTAGTAACTGCACCCATGAAAAGCACCACATTTGGATGACGTACTCGTTTCATTATGGCAACCTCTCTGAGAAATTCTTTCAACTGATCATCCTGGAAATCCTGAACTGTTAAAACCTTGACAGCAACATCCGATCCATGCCATTCAGCACGGTACACTGTCCCAAATGATCCAGCACCAACACGCTCTTTGATTCGTAAATCGTCCCACGGTATCTCAAGCCAGTCCATTG

>PvRGL220

CAACATAATCTTCATATATAATGGGCGAATAACATATTCCAAATGAGAAACTGAAAATACATTTCCACAGATAATAAATGCAATGACTCAAGTTGTACTGATTTCTACAGCCCCACAATAAACTACTGAAATAATATACTTATCTGAAACTGATAATAGAATTTCATTTATTTCCATAAAGATCTATTCTAATTTCTCATACATACAATGACAAAAGTTGCTTGTAACATACACTTCATTCTGCAACTTGATGACCCAATAGAAATTACAACCTTCTTCCTTTAATAATCACAACATCATCATCAATGGGCATGTAATCTGAACACAGAAGATAATTTCAAAAGAACAAGAGAAGAGGAAGGTAGAAGGGTGAAAATCATATGCACAAATCAGAAGTATATCCCTTTCCAACAGAAAGCCTAGTGAGGCAGAATTGTGTATTCACTTGTCCATAGAATGTTAAAGGGATCATCTAGGACCTGACAGTTCATCTGCTTGGATGTGGGAAGTTGAATCTACTATCCAATTAGCATTGGGGTGATGAATGTTGTTGTTGAAGTCTTGTCGGAACGTCTCGTCTTTCTGCCATTGCTCCCATTTCTCGGCCAAACCGTCACCTTCTAGCATTCTGACAACCTCTGACATCTTTGGTCTTTCCATAGGAGAACCTTGTGTGCATAGTAAGGCCACTTGGATTAACTGCTCTACCTCCTCGTCAATGTAATTGCCCACTAAATCTGCATCTACCAGTGTTTCCAATTTCCTGTCTTTGAGAAGTCCTTTAACCCAATCAAGCAACATGACATCATCATCATTGGCAAGTCGAGCTAGATCAAAAGCCCTTTGTCCAGTTATGAGTTCAAGAAGCATCACACCATATCCAAAAACATCAGTCTTCTCTGAAGACTTTCCAGTTGAAAGGTACTCAGGAGCTATATGTCCAATTGTTCCACGTACAGCAGTAGTAACGTGAGTATCTTTATAATCCATAAGTTTTGCCAAACCAAAATCTCCCACAACTGCTTCAAATTCCTCATCCAACAAAATATTTGCTGCTTTGACATCACGATGAATAATCTTAGGGTCACAATGATCATGCAAATAAGCAAGTCCCCTTGCAGATCCCAAAGCAATACGCTTCCGTTCTGGCCAG

>PvRGL221

GATAAGAGGAAGTCTTCATCTTTGGACTGGGCAAAACGATTCAGAATAGTTACTGGGGTGGCAAGAGGACTTCTCTACCTGCATGAAGAGGCCCCAGAAAGAATTATACATAGGGACATTAAAGCTAGTAACATATTATTGGATGACAAGTTAGTTCCGAAAATTTCAGACTTTGGTTTGGCAAGGCTGTTTCCTGGGGAAGACACTCACGTGCAGACATTTAAGATTTCCGGTACACATGGTTACATGGCTCCTGAATATGCATTGCGAGGATATCTGTCAGTGAAGACGGATGTTTTCAGTTATGGAGTCCTGGTCTTGGAAATAGTTAGTGGGAGAAAAAACCAAGATATGCGACTTGGGCCAGAAAAAGCAGATCTCTTGAGCTATGCATGGTCGCTTTATCAAGGAGGGAGAATAATGGAATTGATTGATCCAACTCTCGGTAAATACAATGGTGATGAGGCAGCAATGTGCATTCAGCTAGGTTTGTTGTGCTGCCAATCAAGCATAATTGAAAGACCTGACATGAATGCTGTTCATCTCATGCTCTCAAGTGATTCCTTCACTTTGCCAAGACCAGGTAAACCAGGAATTCAAGGTCGTGCAGGACATTGGACTACAACTACATCTACTGCTTTGACCAATACCAATCCTAGTAATACTACCAGGGCCTCAGGTGGTAGTGGTAGTTTTATAGAGGATTATTCTAGAAATTCTATATCTACCTCTTCTTTTGATGAAGGCAGATGACCTCCATCCATATTGTATAGGGTGCAATAGAAGCTCTATTTATTGAAGGGTATTCTTTTTGTATTACTGTAGTTACCTTAACAGAGAAATGGTTTGATGACAAACAAAATTCAGCATAGTTGATAGATATAAATGGGTTTGATTCTTGACCATATTTTTTTTGAAAGGATAAAGTTCACTTCAATAATCTTTACA

>PvRGL222

TACAGAACAAAATTGAGTAACTTCTTCAACATGAATAATGAGTTTCTTTACACGAGAAGAATGAACTGTGTACATGTGTTTTCTATGTAAACTGTATTGGAACTTACAGTATGCACAATTTTTTTTATGTAAAGTCACAAAAGACTATACATGGTATCCATAATTTGAGTCCCCAGTGGGATCATATATCTTAATAAGCATATCAAGAACCTCCTTCATGTTAGGCCTAGCCTGTGGTGCATCATGTGTGCATTTCACTCCAACTTGGAGCAAGTCACCCATCTCCTTAGCACCCTCAACTACCCCACTTCCCTTCAACAAAACTGGCACAGATTGAGCTAACCCTTGTCTTCCCGAACCCATCATAACACGCCTAGTCCATTCCACCAAACACTCTTCCCCTCCATCCACTGCTCTTCTCCCAGTTGCTAGCTCCATAACCAACACCCCAAAACTGTACACATCCCCTTTTGTGGTGGCTTTCCATGTTTGTCCATATTCTGGTGCCACATAGCCCACTGTGCCAGCCACTATAGTGCTCACATGACTGTCTCCAACATCAACAATTCTAGCAAGTCCAAAGTCTGTGACCTTGGCATTTCCATTCTTGTCTAGCAACACGTTACTAGCCTTCACATCTCTATGCACAATAGAAGGGTAGCACTCATGGTGTAAATAGACCAGTGCCCTGGCCACATCAATTGCCACTTCTAGGCGCCTTCTCCATCTCAATTCTTTTTGTATCAGTCACAAGTTCCTCCAAGCTTCCACCTCCTATGTACTCATAAACGAGTATCTTCTGTGAACCATAGAGGCACCAACCATACAGTGTGACAAGGTTTGGATGAGGCCAGCTAAATCCATGACCACTTAGAACCTTCATTTCAGCTCTGAATTCTTTTTCACCCTCAGTTCCTTCCCTTTGGAGCTTCTTCACCGCCACCTCTCTCCCATCTGGAAATGACCCTCTATATACTGTTCCATAGCCACC

>PvRGL223

CGGCGGGTGCTTGTCGTGGAACTTGAGGTACACAGTTGCTATTGATGTTGCCAAGGGACTTGCTTATCTTCACCATGATTGCAGGTCGAGGATCTTGCACCTTGACGTAAAGCCAGAAAATATATTGTTGGATGAGAATTACAGAGCACTTGTTTCAGATTTTGGTTTGGCAAAGCTTGTTGGCAAGGATGAAAGCCTAAAATTTGTGTCTGCTATTAGAGGAACAAGAGGGTACTTGGCACCCGAGTGGCTCTTAGAGCAAGGTATTTCCGATAAGACTGACATATATAGTTATGGGATGGTTCTCTTGGAGATTGTTGGAGGCAGGAAAAACGTCTCCTACATGGATGATGAAAAGGCCAAGTCAAAACGAAAATGGCAGTACTTTCCAAAGATTGTGAATGAGAAAGTGAGGGAAGGTAAACTCATGGAAATTGTTGATCATAGGTTGTTGGAAGGTGGGGGTGTTGATGAGAGGCAGGTAAAGACACTGGTTTATGTTGCTTTGTGGTGTGTGCAAGAGAAGCCAAGATCAAGGCCTAGCATGGCCCAAGTTGTTGATATGCTTGAAGGGCGTGTGAGGGTAGAACTGCCACCTGATACCAGAATGGTTCTTGTTGATTTTCTGGGTGTGGATGATAGTGCAACAGATAGCAGTAGCAAGCCAAGGGTGGGCTTTGTGTCGAATCAAAGAGCACAAAGCGATGTAGAGTGTTCATCAACCTACTCTCTTGCTACCACTGTTATGTCCGGAAGATAAGATGCCTTTGCTACTCTATTTGGAAAAAACGATGATGTTTTTATAATCTGAGTAATGTTTCTGCAAAAAACATTCATGACATTTCACTGTAATATTGTTTTTTTGTCTTTGTTTCTCGATTACATCATGTATTTTAATTTATATTTCTCTGTTATTGTAAAAAGTTTTGTAAGAATTTTGAGGAAGAATAGCTTTACTGTTCATAGCTTATTTGATTTCTTTTCTTGGTTGTATGATTAATTACATGTTTCGGTAGAATGTGAATTTGAAATATAGTTCCTAAA

>PvRGL224

TAGAACAAATGTTGCTTTCACACTTCAGTTTCTATTTTTTGTTGTCTTGCCAAGAATGACAAAATGGTACAATTCCATAAATATTACAATTACACCTGCATGTATATTATAACTTGACAAAATGGTACAATTCCATCAATAGTACAATTACACCTGCGTGTATATAACTTCAAATATTGTTTCGCACAATTTGCCTCTTCTAAGCCTAGTTCCCATTTGATTTAACAAAAAGCTAATTTGGCCCTAAAGAAGGGCAGGTTAAAGTACATAATATATGTTAAATGTATAAGAAAATATCCCCTCCAGTTTGAATTATAGATACAGAAATTTGAGGTGAAGAGTACTACCACTAGATTAGCATTACTTTGCAGGTGGTTGGGCAATCTGTTGCAGCTGGTGAACAACATGAGACATTCTAGGGCGCATAGAAGGAACATGCTGCGTGCAGGAATAAACAAGGTCAACCACCTTTTGGATGGTACTAGCCTCTGGGATGATGCTAGAAGAGGATGAAGTTATATGAGGATCCAAGAGTTCATGGTAGCGATGAACTTGCACAAGAGGTGTTGCCCATTCAAAGATACTCTGCCAACCAACAGAATCAACTGCCTGTGTAGGTCTACGTCCACTTACTATTTCAAGTAGTAGGACGCCAAAACTATATACGTCACTCTTAGTTGTAAACTCGTTTCTATACACAAACTCTGGAGCAAGATATCCAGAAGTTCCACCTGCCATCACAGTCCTCTCATGCATTACTTCGTACGGCACAATCTTGGACAGACCAACACCCATCAGCCGTGCTCCAAACTCCTCATCCAGCAGTACATTGCTTGCACGTATATCTCGATGCACCACTTGTGGCTTAACCTTGTCATGCAGAAACGCAATTCCTTGAGCAAGCGTTGTCGCAATTTTCATCCTCATAGGCCAATCTAAGCTGCGACCTCCCCTAGGTATGTGATGTAGCCATTTATCCAAGGGACCATTAGCTACAAACTCATAAACAATGTAGCGGTCACCATGATCATAGCAGCATCCCATCACAGCAACCAGATTGGGATATCTAAGCCTAGCAACCCTGCTAATTTCAGAATAAAACTCCTTTTTCCTCTGAAAGCTTGTCCTCTTTAATCTCTTAACTGCAACCCTTGAACCATCTGATAAAACCCCACTGTATGTACCACCAGTCTTGGTATCGCCAACAAGACGGTTACCTTCACTGAAATTCTTTGTAATTGACCTTAATTCCTCTTTGGTGAAGACTTTCCATGATGGAAGAGAAGCTCCTGGAATAGATATTTTTCTAGACCGCCTTCTTTTTTTTTCCCGCTTATAAACAAGAAGCCAAACTACCACTGCTAATGTTGTGGAGAGTATCAAGGCACTCACCACTATGAGGATGATAAGGTAATAATCCCTATGGCAGTGCATACGGTTACATGCAGCATCTATATCAAGTATACATAGAGAAGAGTGAGGATTGTCGCATCTCTCACTCATGAGAGAGTTTGAATTATTAGTCATTACCGTGCACAAATTGTCTGTACTGTTTTTATGACAGGATAGTTCAGTTCCATTACAATGGGGAAAGACATCTTCCAATGGAAATATGGAGTTATTCCCTGGTGAATTGTCAGACCACATCCACTGATAACCAGCTGTAGTGTTGAATCTTTGTCCACCAATCCAGCAACTATTCACAGATTCACCACATAGGCTCTGAGCAAATTGTAGCTCTGAAGTGATGTCAATGACGCTAAAT

>PvRGL225

GGAAGAATTGGTCGGCATCCCAATGTTATGCCCCTTCGAGCTTTTTACTTTTCCAAAGATGAGAAACTTCTAGTATATGACTACATGCCTGGAGGCAGCTTGTTTTCCTTGTTGCATGGAAACAGAGGCATGGGAAGAGCTCCATTAGACTGGGATTCAAGAATGAAGATAGCACTTGGAGCTGCAAAGGGAATTGCTTCCATTCACACCGACCACATGGATTCAAAACTTACTCATGGCAACATCAAGTCCTCCAATGTGCTCATAACCCAAGAACACGATGGCTGCATCACTGATGTAGGACTCACTCCTGTGATGACCACCCAATCAACCATGTCAAAAACCAACGGCTACCGTGCTCCCGAAGTAACCGAGTACCGGAGGATCACTCAGAAGTCTGATGTTTACAGCTTTGGAGTGCTCCTTCTTGAAATGCTCACTGGCAAGGCTCCAATGGGATATTCTGGCTATGAGGACATGGTTGATCTACCAAGATGGGTGAGGTCCGTGGTTCGAGAGGAATGGACTGCTGAAGTTTTTGATGAGGAACTGCTTAGAGGGCAGTATTTTGAAGAGGAAATGGTGCAGATGCTTCAGATTGCACTGGCATGTGTGGCAAAAGTGGCAGATAATAGGCCAACAATGGATGAAACTGTTAGGAACATAGAGGAAATTAGGCTTCCTGATTTGAATAACCGTAACACATCATCTGAGTCTGAATCTAATGTACAAACTCCATGATTATTTTGTGCCATGGACTTCTTATTTTCTGTATCACACAAGGAATAACCTTGTTGTGTCTCTAGAGTATTCAAGTTAAGATAACTGTATTGTATCATGTGGTATGTTCAGATGACGAAGTAGTTTCTTATTCCACAAATGTACTAAGAAACGTAGTGTCAAGTGAGTGAGTGACATTGAAGAAGTAAAAATTTCCAAATCTTGCAGTTCAGTATCA

>PvRGL226

TGAGGAAAGAAATTCCATAACTCCTCTCCCTTTACCAAAAGAAAAAGAAAGAGAAGGGAAGAAAAAGCAATTATGAAGAAGCAGCTTATGTTTTTCTGATCTTCCAGAAAGCAAGTGCAGCTTTTCCTCCATGACAATGAAACAAATGAAAGGACAAAAGAAAAGAAGAGTAATGAAACTGTACACACCACCATTAGTGCAAGGGAGCTGTTCAATCCCACATCCATACACAATAATCTACAACCTAACATCTACAACTAACTATAACAACAAATAGGTATATAAACCATTCCAAAGTAGCTGCTTCCTGCTCCATGTTTTTCATGCATAAAGTGGCAGCCATCAGAACTCTTACAAACCTCAAAAGAGTGCAAAATTTCCCACTTAACTTGTTTTCTTCATTCCATAAATTCTTTGAGATCTACTACACACATTGGTTCCTTCTGCCATTGGACTTGAAATCTGCATTCTCACTCTTATCTGTATCAGAATGAGTCTCCACCTCCATGTTTCCTGCCTGACTCTTCCGCCGTCTTCGATCCTCTCGAGGTATAGGATATTCCTCTGATTCAAGCATGCGGACAACTTGGCTCATCCTTGGTCTTTTTTCAGCATCTGGATCAACACACCTCAAAGCAGTCAAGAGGGCTCGTTTAAGGGCACTTGTCGATGGCCTAGTCTCGATGTTGGGATCCAACACCTCTTCTGAGCGTCGAAAACCTACCATCATCTTCAGCCAGTCCACCAAATTTACCTCGGCTGCTGGTCGGCTATAATCCACGGGATCTCTTCCTGTAATTGCTTCAAGAAGCAATACCCCAAAGCTGTAAACATCACTCTTCTCATTTAGCAAGCCAGAATTGGCATATTCTGGAGCTACATATCCAAAAGTACCCATTACTCTGGTTGTAATATGACTTTTTCCAGCACCAAGTAACTTAGCCAGCCCAAAGTCAGATATTTTGGCATTAAAGTCCTCATCAATTAGAATATTGCTCGACTTAATATCTCTATGCACAACTTTTGGCTCAATTGCCTCATGCAAATAAGCCAGC

>PvRGL227

TTTAGCTAACATGACACAACTTGCTTTTCTGGATATCTCTTATAACAACTTGAGTGAATCTGTACCAAGAATAAATGCAAAAACATTCAATATCGTAGGCAATCCTCAGATATGTGCCACTGGAGTCGAGCAGAACTGCTTCAGAACAACGCCAATTCCTTCAGCTCTAAATAATTCTCAAGATTCACGATCCACCAAAGGACCAAAGAGTCACAAAGTAGCCTTGGCGTTTGCTTCAAGCATAAGCTGCATAGGCTTACTAATTCTGGGACTTGGCTTCCTCATCTGGTGGAGACAAAGACATAACAAGCAAATATTCTTTGATGTTAATGAACAACACCGTGAAGAGGTTTTCCTTGGAAACCTCAAGAAGTTCCACTTCAGAGAACTTCAACTTGCTACAAAAAACTTCAGCAGCAAGAACTTGATTGGCAAAGGTGGTTTTGGAAATGTCTACAAAGGGTATCTCCAAGATGGTACAGTTATAGCAGTGAAAAGGCTCAAAGATGGTAACGCCATTGGAGGTGAGATCCAATTCCAGACTGAAGTTGAGATGATCAGCTTAGCCGTCCATCGGAATCTTCTTCGACTATATGGATTCTGCATGACAACTACAGAGAGGCTCTTGGTTTATCCGTACATGTCAAATGGAAGTGTAGCTTCCCGTCTGAAAGCCAAGCCAGCTTTGGACTGGCCTACGAGGAAGAGAATAGCTTTAGGAGCTGGAAGAGGCTTGCTGTATTTGCATGAACAGTGTGATCCAAAGATTATTCACAGGGATGTGAAAGCAGCAAATATCTTGCTTGATGATTATTGTGAGGCTGTGGTGGGAGATTTTGGGCTGGCAAAGCTGTTAGATCACAGGGATTCACATGTGACAACAGCAGTGAGAGGCACTGTTGGACACATAGCCCCTGAGTATCTGTCAACAGGTCAATCTTCTGAGAAAACAGATGTGTTTGGGTTTGGAATTCTTCTTCTTGAACTAATATCTGGCCAAAGGGCTCTTGAGTTTGGGAAAGCAGCAAACCAAAAAGGAGCCATGCTAGACTGGGTGAAGAAAATCCACCAAGAGAAGAAAATTGACTTGTTGGTTGACAAGGACCTGAAAAACCACTATGATAGGATTGAGCTTGATGAAATTGTGCAAGTGGCTCTTTTATGTACTCAGTACCTTCCAAGTCACAGACCCAAGATGTCTGAAGTGGTTAGGATGCTTGAAGGAGATGGTCTTGCAGAGAAATGGGAAGCCTCACAGAGAGCTGAATCAACAAGAAGTAGAGGAAATGAACTCTCCTCTTCAGAGCGCTACTCTGATCTCACTGATGATTCTTCATTACTTACACAAGCAATGGAACTTTCTGGGCCAAGATGATATATACTAAGCCACTTTGTGTGATATTTAAGTTGGTGAAGAGTGAAAGAAAGTTATATTTTCTTCAAATGCAAGTTCCTTAGTTGCAGTGAACTTGGTGTAACATTAGCATCATAGTAGCATCTCCAAGAGTGATGCTAGTTTCTGACTGTACAGAATCTGATTGAAGACATTGACTTATAGATTGAGTGTTATGTATTTTTCTTAAGTGATGGACCGAAGATTTTGTGGGAAATGTCCTTCTGGCTCCTTCAACTTGTTAGAAGAATAACGTTCATATAAATATATTGTACTTAAACTGTCAATAATAGTATTTTGGGATTTGAGAGTTTTCA

>PvRGL228

TGTAAGTGTGAACTAAAAGAAAGTTGAATATATTTTTCCAGTGATTTCTAATCCAAACTTTAAATCTTCTATGTATTTGTAGTAAGAAATTATATAATAGATCCTTACAAATTCTTTTTCCTCATCAAATTTTGGGGGCAAAGGAGTTACAATATAAGAACTTATACTATTCTGATGCTGCTGTATCTGCAACCATGAACCTTATAGGCTTATAGTGAAATGTACAAAAAAAAAGTGGAAACCTTTGGATTGAAACTAATGAAAGGGAAGTCCTCCAGATTCTGAGCCTGGTGACCCTTCACTACTTTGTGAATCCCCTCTCCATGCATCCTGCACTTGAGATGCAAACTGCAAGTTCCACAAAACATCTTCTATGGAGGGCCTATCTTCTGCCTCTTTAACCAGACACCTCACACATATCTCCATCATTGTCTTCAATGATTGATCCAAGCATGCCTTACGAATTGCTGTATCAACCACATTCCTCCTACCCTCTTCATCAGCTCCTAAGCTTGCTTGCAACAGATCCCTAAAAGCGTCTGCATCATTCGCTGTCCTTATTTGCCTTCCTAGAATGAGTTCGAGTAGTATCACTCCAAGGTCGTATATATCAGACTTATCTCCATGCTTGACACTTTTATTGTTGCTAGAATGTTTTAATCCACTTGAAGAATTTCCATGTCGAACCTTCCCCATGTTAGATAACAAAGGCAGGTGATAACTGCTGATTTTCGCAACAAGATTCTGATCCAATAAAACATCTTCTATCTTGAGATCGTTGGAATATACACCAGGGACAATCCCTGTGTGCAAAAACTGGATTCCCTTTGCCACTCCAATTGCAGCTCCAATGCGTTGGGTCCAATTCAAGCATTTTCTTGCATTCTCATCACAGATCCAATTCCTGAGCGTGCCGTTTGGTACGTATTCAAAGACAAGAAACACTTTGTTGACACTCGAATCATCCAAAGAACATTCAAAGCAGTGTCCAATAGCACTGACTAAGTGACGATGCCTGAGTTTTGATATCAGCTCTATGTGGTTCACAAAGTTTTGAGTGCTGTATTTCTTTTTCATTTCTACACATCGAATAGCAACGACCGAACCATTCTTCAGCTGACCTCTGTACATCTTTCCATAGGAATCTTCACCCATTAAAGAAACTGTGTCAAAGTAGTTTGTAGCTGCCTCAATCTCTTCCAATGAGAAACTTCTATAAGTTGGTAGGCCAACTGCTCCCAACTTCTTTGTTTGAGATATATACCTTGCATCAGAAAGCAACTTAGAAGTGTAACCAGAAGCAGCATTTTCTGATATTAATCTTGTTGGAGGATTCTTCATTTTGGTTCTAGCATTTCCTCTTCTAACAATAAAGAAAATTAGCAAAACAAGTGCTACTCCTCCAAGAGTCCCACCTACTATGCCTAGGGAAAGAACTACCTTAGATACTTTTCTGTGCTTCTTTCTCTCAGGTAATATTCCCACGGCTAAGGCCTCAGTGTGGCAAAAAGGTTGCGGCTGCTGATTTTGATTCGCAGTGTCCAGACAATTTCTAGCATAATGGAAAGTCCTATCACTGGAATTTGACACTAAACATTTAGGTAAGCTCCCAGTCAAAAGATTTGAGGATAAATCAACCACATCAAGCTCTGA

>PvRGL229

TGGGAATCAAATCATAGGAGCATTGGGATTAGGACATTGCAACTTGGTTGTAGAATCATGATCCACATGCAACTCTGAAACCCACATGCAATACAAGAATTTTTCAGAACTCTCTTTGACCTATGCTCTCTTGCATGCACATGCAACCAAGAACACCCTCATAATAACTTTTGTCCATTTCAACACTTGCCCTAATTTTATATTACATCACAAGAAAATATATCGCATTAAATAAATTCAAATCACACTTTGCCTCCTTCACTACAACCAAATCATCATCATATCTTGGAACATATATATTTATATTCACCGACAACCCAACACTCATTCGGGTCGGATCGGGTCGACCATCAACAAGCCTTCAACGGGTTTCCACACAAACAATCATTGTTGCTGAACGACGCCGCATCGATATGTTCAAAGGAAGACCCGATTGGGATGGTTCCACACAAGTGGTTGTTGCTCAAATCCAAATGCCCCATAAACTTAGACGCGGATAACGAAGCGGGTATCCGACCCGAGAAATTGTTGAAGGACAAGTCAAGAACCATGAAATAAGAACCCGCCCCAAAAACATCGGGTATGGTACCCGAAAACCCGTTTCGGCTAAGGTTCAAAATACCCATACCCGTATTTCTCAACAAAGTGGAAGGAACCGGACCCGTCATGGAGTTACCATCCAATTTCAAAGTAGAGAGAACCTTCATTTTTCCCAGCTCAGCCGGTATCGACCCGGTTAACCGGTTCAACGACACATCCAAATCCGCAAGCCGTTTCATTCTGGATATGGATTTGGGTATTGACCCGGTTAATTGGTTCCCACTCAGCAATGCCCGACTCAACATTCGGAGCTTCCCGAAATCATACGGTATC

>PvRGL230

TCCAAAGAAAAATCAATAACATTGACATCTACATTTAAATAAGAGAGTAGTAACATAACATCCTTTAATCTTAAATCATTAATCCTTTAAGATTACACAACGGAGGAAACTTATTTGAGCTAATTGAAATGTGAGCCATTGTATGTTTTTCATTATTAACATAATAGTATTATCATTTAAATGACCTTTGAATGAAGGTAGTAGTTAGTGCAGTGTAGATAAATGTTAGGCATTAAACCAAAAGATTACTAGAGTGATTTTTTCCTAGTTTATCCAGAAGGACCATATTGCATACAAAATATTTGACATATTTCCTCTGTGGCTTAAATATACATTGATTCTGAAAGTGGTCACACGCTGTTTTGAGATATTACTTCTCCAAATTTGAGGAAGAGTTGGGCATCAGAGGTGCTGATGGAGGGACAGTTGACCAAGTGTGGAGCTTTGAGATTGGCATATTCGTCCTTGTAGGATTGCGTTTTAGCAGATGGCCGTGAAGGAGGTATGAGTACTTGCATTGTTGGGACTGATTCTTTTGGTGGCACTAAACTTCCAAGCACACGCGTCACCTCGTGCATTGTGGGTCTATCCACTGGTTGCTTCTTGGTGCACAAAAGAGCAAGCTGAAAAACTTTTTTTACTGCTATCATTTCCTTGCATGTGGCACTAATATCTGGATCAACGGTTTCCATTACAGCATCACTAGCTGTCTTGGACAAAATCAGATGATGAAGGTTTGATTCGTTTTCCACAGCTTTCCTCCCAGTTAGCAGCTCAAGTAATACAATACCGTAGCTATACACATCAGATTTCTCAGTGAGTCGAGAAGTTCTAGAATACTCAGGGTCTATGTAGCCAATTGTGCCCATTATGTAAGTTGAAGTGTGGGATTTAGAGGGGCATAGACTCTTTGCAATGCCAAAATCGGTGAGATGGGGCTCAAAGTCTTTATCTAGTAATATATTAGATGATTTCACATCCCTGTGAATGATACGTGGACAGCAATCATGGTGTAGATAAGCTAGCCCTTGGGCTGATCCAAGAGCTATTTTAAGACGAACATCCCAATCAAGCCTTTTCCTCTTGGAAGGTCCATGTAGAAGATCCCACAGACTGCCATTTTCCATGTAATCATAAATGAGGAGGTTTCCAAATGGAGACAAAGAGTAGCCTTGGAGACTGACCAGA

>PvRGL231

GCTTCTTTAGGACTGCTTTCTTGACTTTGTCGTCTCTGCCTCCATGTCTCCCCCCACAGCTTGGCTTCTGCAACAGCTCGCTCTCTTACTGATTCTTTAGGCAAAATCGCAGTCGTTTCATTTGAGGACTCTCCACTCTCCATCTCAGCTGTGTTGTTGACTGTGTTCAACACTTTTGGGGAATATTGCTTGGATGACAAGAATTCCAGAGCTTCCACTATATCACCTGCATTAGGCCGTTGATGTGGCTCTTCACGAAGACACATGGCTGTCAATTCAATGACCATCGGTAGACCAGATCCTGGGTACTTGCCTTCAAGCCGTGCATCTACCAGTCTAGGGAAACTCCTTTTCTCTCTGAACATTGGACGTGCCCAGTCTACCAGATGCTTTTCAGTGCCACGGTTATCATCGTATGCTTTGCGTCCAGTAATTAGTTCCAATAACACAACCCCAAAACTGTAAATGTCAGATCTCTTCGTTAATTTTCCACTAGTGGCATATTCAGGGGCACAGTAACCTTGTGTTCCCATGACCCTAGTGGCAACATATGATTCATCTCCGGTTGGACCAAACTTTGCAAGCCCGAAATCAGAAAGTTTTGGATGGAAATCTTCATCCAATAGTATGTTGGAAGTTTTAAGATCCCTGTATATAACAGAGGGCTTTGCTTCATCGTGCAGATAATGGAGTCCTTTTGCTGACCCACAGGCTATCATCATTCTGGTATTCCAATCAAGTGGTTCTTCATCAGGAGAAACATCATGAAGATGAGATTCTAGGGATCCCAAAGCCATGTACTCATACACAAGAAGACGTTGATCCCCTTCCGCACAGTAACCAATCATATTAACAAGGTTGGAATGGCGTAGAAGGGAAAGCATGAGAACTTCCACCAGAAATTCTTTCTCCCCTTGGACACCTGTAGTATCAAGTCTTTTAACAGCTACAACCTGATTTTCTCCAATCATTCCCTTGTAAACAATGCCAAATCCACCTTGCCCAATAAAGGTTTCATCTCTAAAATTTTTTGTTGCTGTTGCAAGCTCTCGGAAGGTAAATATCCGGGCCTCATGAGGACTTTCGCCCTCCTCTTTTGGGTTTTCTTCTGGTCCATTTCCTGAAGAACTTGCAGAGACATCATTGATTCTCTCTACAGATTTACGATTCTTGTTTTCCTCGTCCC

>PvRGL232

GAGAGCAACACCGCAGTGGACATCATGGCAGCACAAAACGGGAGCCTTCCGAAACAAGGCACTCTTGTAACCGTTGACGGGGAAACAAATCTGGAGCTCGAGACTCTGCTCAAGGCTTCAGCGTATATTCTGGGAAACAGCCTCGTCAGCATTGTCTACAAGGCAGTGCTAGAGGACGGGAGAGCCTTTGCTGTTAGAAGGATCGGTGAGTGTGGGATTGAGAGAAGGAAGGACTTTGAGAACCAAGTGCGTGCCATTGCAAAGCTTCGCCATCCCAATTTGGTTAAGGTTCGCGCCTTCTGCTGGGGTCGAGATGACAAACTCCTAATATGTGACTATCTCCCCAATGGCAGCCTCGCCACCACTGATCACAGAAAAGCAGGTTCGTCTCCTTTGAACTTGTCCTTGGAGATTCGGTTTAAGATAGCGAAAGGGGTGGCACGAGGTTTGGTCTTCCTTCACGAGAAGAAACACGTGCATGGCAATGTTAAACCGAGCAACATCCTGCTGAACTCAGAAATGGAAGGCATCATAAGCGATTTTGGGCTGGACCGGCTCCTCCTGAACGATGTGACCCACAGAGGAAACGGTTCGGGTAGACAATTAATGGCGAGCCAGAGAAGCCAGCAAGAGGTGGCATTTGGGTCAAGTCCCTTCGCAGCAATGGGATCATCGTCGAGTGGTGCGGGCCACATAATGCCGTATCAAGCCCCAGAAGCAATTGAGAACATAAAACCCAGTCAGAAATGGGACGTGTACTCTTTTGGAATGGTGTTGCTGGAGCTTCTAACAGGGAGGGTTTTCCAGGAGCGGGAGTTGGAGCAGTGGAACGAACCGGGTTCGGTGGAGGAGGAAAAAAACCGGGTGTTGAGAATGGTTGACGTAGCAGTAAAATCTGAAATAGAAGGGAGGGAGAATGTGGTAGTGGCATGGTTGAAGTTGGGGCTTAGCTGTGTTTCTCATGCCCCACAAAAGAGACCCTCCATGAAAGAGGCACTACAAATCTTAGACAAGATCCCTGCACCTGCTCCACTCAATTAATCCCACACCTAGATCAAGGTGTTTCTTTTTCCTTTTCCATCTTTTCTTCACTTCAATTCGCTTCAACAGAGGAAAATATTAGAAACAAGTGTTTGTTTATGCAAGGTGACAATCATTGTAACTTTCACTAATCACCACTCAAACCAATTCTTGTCTCATGCTGCATTTTTGCTCACGGGCATGGC

>PvRGL233

GCATAGATAGGATGAAATTACCATCACAAAAACTAGTATTTTCTCCTTTACCATATGAAATGTATTATGACTAAAGCAGTTATGAAAAACGAATCAAAAGAATTCCATTTTCAACCATTTGCTATCTCGGCTGTGGATGGGACAAGGCTTGTGTGGTGATGGTTGATTCTGCTGTTGATGAAGTTGAGGGCAGCACCCTTTTCTCTATGACAATAGAGTAGCGGTTGGATCCTCCAAGGCTGTCAATGGACTTCATGTATTCTGATGCATTGTTTTCTATGATGAGAGCATCCTCTAACTCACGGACAATGTCCACCATGTTTGGTCTATATGCTGAGAATGGTTCAAGACACTGCAGTGCTACTTCCACCACTCTCCACATTGCCTCTGCATGGTATCCCCCCTTGATGCCAGGATCCACAATTTCTTCCATCTTTGATGCTCTTATATATGGCTTAGCCCATTCAACCAAGCTCCATTCATTCCGTGGTCTCTTTATGTCGAGAGGTTCCCTACCAGTCACAATTTCAAGTAAAACCACGCCATAGCTGAAGACATCACTCTTCTCAGATAATTGTTGGGTTTTGTAGTATTCAGGATCCAGGTACCCTGCAGTTCCTCTGACTTCAAGAGAAACGTTACTGTCTCCTTCTTGAGGAGCATATTTTGAGAAACCAAAATCTGCAACCTTAGCGCACATGCTATGATCTAGAAGTATATTGCTTGATTTTACGTCCCTATGTATTACTGAACGTCCTGGAAATGTATGAAGATATGCCAAACCTCGAGCTGCACCAAGAGCAATAGAGAGTCTAGTTGGCCAGTCTAATATTTTTCTCTTTGCAGGTTCCCCATAGAGTCTATCTTGCAAAGAACCATTGGACATGAAAGGATACACGAGAATTTGTTGATCATTTTCATTACAGTAACCAAGAAGAGGCACTAGGTTTTCATGCTGTATTGCAGAAAGTAGGTTTAGCTCATTATCAAATTCTCGAGTTCCCTGCGTTGATGTGGCTGAACGGACTTTCACT

>PvRGL234

TAACAAGGGAAGCAAAGGGAGGAAAGAGTGTTTCTGCTTCAGAAAGGATGAATCCGAGGCCCTGTCGGATAATAATGTTGAGCAATATGATCTTGTCCCATTAGATTCCCATGTGACTTTTGATTTGGATGAGCTTCTGAAGGCTTCAGCTTTTGTTCTGGGTAAGAGTGGAATAGGGATTATGTACAAAGTGGTGCTTGAAGATGGGCTTGCTTTGGCTGTGAGGAGGCTGGGGGAGGGAGGTTCTCAAAGGTTTAAAGAGTTTCAAACAGAGGTAGAAGCAATAGGGAAGTTAAGACATCCAAATATTGCAACTCTGAGAGCCTATTACTGGTCTGTTGATGAGAAGCTTCTCATATATGATTATATATCCAACGGAAGCCTTGATACAGCAATTCATGGGAAGGCTGGACTTCTCACATTTGCACCACTTTCTTGGTCCTATCGATTGAAGATCATGAAAGGAACAGCAAGAGGGTTGGTGTATCTGCATGAATTTAGCCCCAAAAAATATGTCCATGGAGACCTCAAGCCAAGTAACATACTTCTTGGACACGACATGGAACCCCACATTTCTGACTTTGGAGTTGGGCGCCTTGCAAACATTGCTGGTGGGTCTCCAACCTTGCAATCCAACCGAGTGGCTGCGGAGAAACAACATGGAAGACAAAAGAGCATCTCCACTGAAGTGACCACCAATGTGTTGGGGAACGGTTACATGGCTCCAGAAGCACTGAAAGTGGTGAAGCCATCACAGAAGTGGGATGTTTACTCATATGGTGTGATACTGTTGGAAATGATCACAGGAAAATCATCCATTGTCCAAGTGGGGAACTCAGAAATGGACCTTGTTCAGTGGATTCAGTTCTGCATTGAGGAAAAGAAGCCTCTCTTGGAGGTGTTGGATCCTTACTTGGCTGAAGATGCAGATAAGGAGGAGGAGATTATTGGAGTTTTGAAGATTGCAATGGCTTGTGTCCATAGCAGCCCTGAAAAGAGACCTACGATGAGGCACGTGCTTGATGCTTTGGATAGATTGACTATTTCCTCTGATTGAGTCATTTCAGGGAGCTGTGCTCTGTGAATGCAGCTCATTTGTGAATCTTTTACCTTTACTCTCTTGTCTTGCTCTTGTTTAACAGTGTCTTCAAGCTGATGAATCCTTGTAATTGGAGTATTAGAAGATAAATCGAATTTGTTTACA

>PvRGL235

TCGTTCTCCTTAATCTCATGGTATTCTTATTAGAATAGTTTAAATGGCTCATTACTACTAAATGAATTTAATTAAGATATCTTACAATTACAGACAAATCAATAAGCTATTCGGATCCAAAAAGATGGAACTTAAACATAACACTACAAAAATCAACATGGAAAAACAAAACTCGTGATTATACCATACATTACAATTACATTACTAAATGCCAAAAAGTGGCATTGTGTATATTAACAAGGGAAACCAATTTGTAGTCTAATTTTCTCCTCCAGTGGCCTTGGAGCTTGCTGAAGAGATGTTACTTCTGGCTTGAGAGGAGGAGGTAATTGAAACGTTGCTCTTCTCCTGTGAACTGTGTGTGGAGTTTGAGTCGGTGTAAAAGACTAAATTCTCCTGGTTGTCAACAAACACTTGAAGGGACCTTGGAATTGGAGGCATGTTCACCTCTAAGATCCCCTCAAGGATTTGAACCACCTGAGCCATGCTTGGCCGGTGAGCCTCATTGTCTTGGACACACCATGAAGCAACTTTTATTATTCGAGTCACCTCCTCAATCTCAGCATTGCCCTCCAAACCATGGTCCAAAAGGCTAACAACAGGACCACCTTGGACAACAACATTTGCCGCAAAGGTTGGAAAGAAGGTAAATTTGCCATCTTCTGATGGCTCAGAGTTCCTCCTTCCGGATACAAACTCAAAAAGCATCATTCCATAGCTGTACACATCTGCTTTGGCTGTGATAGCCACCCCAGAAATCCACTCTGGAGCAAGATACCCTCTTGTTCCTCTCATGGTTGTGAGAACCCTGCTGAAATCCCTTCCAACAAGCTTAGCCAGGCCAAAGTCTGCAACCTTGGGACAAAATTCAGAATCTAGGAGAATGTTTTCTGGCTTCACATCACAGTGTATGATACAATCTCTACAATTCTCGTGAAGGTAAGTTAATCCCCTTGCTGTTCCCAACGCAATTTGGTATCTCATTTTCCAATCCAACAACACCTTACGGCTCTTGTTGGGGAACAAATGGAAATCCAAGGAGCCATTTGGCATGTAATCATAAACTAGCAGCTTTTTTGCACCTTCAGAGCAGAATCCACGGAGCCTAACAAGATTAACATGTTGTACTGTCCCTATTGTGCTAACTTCTGTTCGGAACTGTTTTTCTCCTTGGCTAATACTCTCCAACCTTTTCACTGCTACCACACTTGAATCACCCAACTTTCCTTTGAAAACAGAACCAAACCCTCCTCCCCCCAATTTCTCTGAGAAATTCCTCGTTGCATTTTGCAAATCTCGGTACCCAAATGCGACCAACGTACCCTCCACAGGCTTTCCTGCTCCAACCATTCTCTTCCTTCGCCTAATCACGAAAAACAAAAGAATGGCCAAGAGAACCCCAATGCCAACAACCAGTCCCACAGCCACACCAATAACAGTCCCCTTACTGCTTTTATCATCATGAAACTCAGATGCTGCAAGTTTGAGATACAAAGTTTCTCCGCTACTATCATCTAAAGATAGCTTCTGCAGATTCAAAAGGTTGCCAATCCAAATAAAACACCCGTTACTGTCATATGCATAAGCCGTGCAGGAACAGTTGTTTAAGCAAGTCGATTCACATTCCCCTGCCTTCCCTGATCCCACAGATTGTGCATGTTTAGGTAACACCATGTTGGGAATCGCAAGAAACCTATCCTTATCCCCTTTAGAGGGATTCAAATTCTCACACTTCAACTTGGTTTTCCTTTTACACCCACCTGAGTGATCCTCCAGATTCCAATCAGACAGTGACTTTGGCTCAAAACCCGGCAAGCAATTACAATACGGCATGGAATTCTCAGTACAGCTCCCAAACACACCACAAAACGCGTATACCTCACACTGTTGTC

>PvRGL236

TATGAATCCAGCAATTTGCTCTTAAATAAAATTTTGGATAAAAAGGAAAAAAGTGGAGCAACTATCTTAGCTCCAGACATAAATGGAAGCGGTTTTGAGTACATTTTTAGGAACTAGACCAGAATTACAGAATTTTGTTCTTTTTTTAGAACTGTACTATGAACTGAATGAACACCACCAAAGGACAACTGATATTAACATCTATAAACCTATATCTTAGAGTTGAGAAGCTCATTAAATAATATATTTGATTTTTTCTCCAATTTTTGTTGTATCCACCAAGTATTGAGCTGCATATCATAGCCCCCTCCTGGTTCTAGGAGGTGTACCCGGAGCAAGGCGAGAGTTTTGAACTGTGTGTGTGTTGGGATCATATTTTTGTTTAGCAAGGTAAGACAAAGCTGTGACAACATCAGCTATAACTGGACGCATGTTAGCCTGCTCCTGAACACACATTGCTGCAACAGCAAGAGCCTGGTATAGTCCTCTTGGAGGATATTGACCTTGGAGTGTTGGATCAGCCATTTGTGAAAACTTTCTTCGATCCCTAAACAAGGGTCTAGCCCATGCAACAAGATTCTGCTCTCCTGCAGATTTTGAATTGTCAATTGCTTTCCTTCCGGTAATTATTTCCAGAAGAACTACACCAAAGCTATAAACATCTGATTTCAGAGTCAGTTGACCAGTCATGGCATACTCTGGAGCACAATATCCATAAGTGCCCATAACTCTAGTTGATACATGTGTGTTTTCCCCAACTGGGCCAAGCTTAGCCAAACCAAAATCAGATAACTTTGGTTGATACCCTTCACCAAGCAAAATGTTAGAGCACTTTAAATCACGGTAGATGACAGGAGGATTAGCTTTGTCGTGTAGATATTCCAATCCCTTTGCTGCACCAGCAGCTATTCTCATTCGCGTGTTCCACTCTAGTTGTTTCTTGCCAAGAGGAATGTCATGCAAGTGGTCTTCCAAGGATCCTAACGGCATATATTCATAAACCAGAAGCCTTTGATCTCCATCAGCACAATAACCAATAAGGTTGACAAGGTTAGGATGATGAAGTAGACTTAACATCAACACTTCAACAAGGAATTCCCTATTTCCTTGGAGTCCATTTCGGTCAAGTTGCTTAATGGCGACAACCTGATTAATACTTTCCAGACGCCCTTTGTACACTCT

>PvRGL237

TGAAGAACTTGAGGTGGCACAAGCAAATTTCCAACAGTGGCAAGTTGGGGAGAAGGCTTTCGCTTGGGGAGTACAACCGCGCAGTGTCATGGTCTAAGTATCTGGTGTCTCCGGGGGCTGAGATAAAAGGAGAAGGTGAAGAAGAATGGAGTGCAGATATGTCTCAGTTGCTCATTGGATCCAAATTTGCTTCGGGCAGGCACAGCAGAATCTACAGAGGGGTTTACAAGCAAAAAGATGTGGCAATTAAGCTCGTTAGTCAGCCGGAGGAAGATGAGGACTTGGCTGCTTTTCTTGAGAAGCAATTCACTTCTGAGGTCTCTTTGCTACTTCGATTAGGCCACCCAAATATCATTACTTTTATTGCTGCTTGCAAGAAACCTCCTGTGTTTTGCATAATTACTGAATATTTGGCTGGAGGTTCACTGGGAAAATTCCTGCACCACCAACAACCAGAAATACTTCCACACAAACTGGTTCTGAAGTTAGCCCTTGACATTGCACGGGGGATGCAATATCTTCATTCTCAAGGGATTCTTCATAGGGATCTCAAATCAGAGAATCTCCTCTTGGGGGAAGATATGTGTGTAAAGGTAGCAGATTTTGGTATCTCATGCTTAGAATCTCAGTGTGGCAGTGCAAAAGGTTTCACCGGAACATACCGTTGGATGGCGCCCGAAATGATCAAAGAAAAGCATCATACCAAGAAAGTAGATGTGTATAGTTTTGGCATAGTTCTATGGGAGCTTTTGACAGGAAAGACTCCATTTGACAACATGACACCAGAGCAGGCAGCATTTGCAGTTTCCCACAAGAATGCAAGACCACCATTGCCATGTGAGTGTCCTTGGGCATTTAGTGATCTGATCAATAGATGCTGGTCAAGCAACCCAGATAAGAGGCCTCATTTTGATGAGATAGTTTCCATTTTGGAGTTCTACACAGAATCACTTGAGCAGGATTCAGAGTTTTTGTCAACTTACAAACCGAGTCCTAGTAGTAGTAGTAACACCATTCTGGGGTGCCTCCCCAAATGCAATGCACGCCATAAATTTGGTGCTTGCAAAGCTTAGCATTACTAATACATGCTACTTCTCAGTTGTGTGACTATCAAGAATTCTATTACAATCAAACCTTTTATGGGAAGAAAAAAGTTTGTTCAATTAACAAATACAGTTCTCTTCCA

>PvRGL238

GAGGGAAGCTTGAGAACAATCGGTTAGTTGCTGTGAAACGGTTCTCAAAACTGTCTTGGCCTGATGCTCAACAGTTCATGGCAGAGGCAGCTGGAGTTGGGAAAGTGAGGCACAAGAGACTGGTGAATCTAATTGGTTGTTGTGCTGAAGGAGATGAAAGGCTCTTGGTAGCTGAGTACATGCCAAATGATACTTTGTCCAAACATCTCTTTCATTGGGACAAGCAACCATTACCATGGGAAATGCGTGTAAGAGTTGCGTACCATGTTGCGCAGGCACTAGATCATTGCAGCATGGAAAACCGGAAAATATATCATGATCTGAATGCATATAGGATTCTTTTTGATGAGGATGGTGATCCCCGCTTGTCGAGTTTTGGGCTTATGAAAAATAGTCGAGATGGGAAAAGCTACAGTACTAATTTAGCTTACACTCCACCTGAATTTTTGCGAACAGGCAGGATAATCCCGGAGAGTGTGATCTACAGTTATGGAACTGTTCTTCTGGATCTTTTAAGTGGCAAGCATATTCCCCCTAG

>PvRGL239

CGGAGAAGCAAATCTCAAGAGCAAACAGATCCCTGGGTTTACAAACCTGCACAGCTTTGGCAGCTTGAAGATCAGACACCACGACCAACTAAAAGGCTACATGGATCATCCGTTTTCACACTCAAGGAGATGGAAGAGGCAACATNTCATTCAGTGATGAGAATCTGCTTGGAAAAGGAGGATTCGGCAAAGTCTACAGAGGCACATTGCGGTCAGGAGAGGTAACACTCTAGACAGAAGTTTTTTGCCCTTAGGAATTTATCATAGTTTACAAGAGTTTGTAACGTCCGTTATAGCTTCCATACAGAAACCATGTTTCTTATAAAATGCTCAAAATTAGTGAATTTGATACAGATGAATACATACTAATATAACAAGTCTCCTGAGGTTGTAGCAATCAAGAAAATGGAGTTGCCAGCAATTAAAGCAGCAGAGGGGGAACGTGAGTTCCGAGTCGAAGTTGACATCTTAAGCGGACTTGACCACCCAAATCTTGTTTCTTTGATAGGCTACTGTGCTGATGGAAAGCATAGATTCCTAGTATATGAATATATGCGTAATGGAAACCTGCAAGATCATTTGAATGGAATTGGGGAAAGAAACATGGATTGGCCTCGAAGACTCCAAGTTGCACTGGGAGCTGCAAAAGGGCTTGCTTATCTTCATTCAAGTTCTGATGTTGGAATTCCTATTGTTCATAGGGATTTTAAATCGACCAATATTCTCTTAGATGACAGCTTTGAAGCAAAGATATCTGATTTTGGTCTCGCCAAGTTAATGCCAGAAGGACAAGAGACACATGTGACTGCCAGAGTACTTGGTACCTTTGGCTATTTTGATCCTGAGTATACATCGACTGGAAAACTCACGCTACAAAGTGATGTTTATGCTTTTGGTGTTGTTCTTTTGGAGCTTTTGACTGGACGTCGAGCTGTAGATCTAAATCAAGGTCCCAACGATCAAAACCTTGTACTACAGGTGAGGAACATCCTGAATGACCGCAAGAAGCTTCGTAAGGTGATAGATCCAGAGATGGCTCGAAATTCCTACACCATTCAGTCTATAGTCATGTTTGCCAATCTGGCATCAAGATGTGTTCGACCAGAGAGTAATGAGAGACCATCAGTGGCAGAATGTGTAAAAGAACTCCTAACGATTATCTATACAAATTCAAAAGGCTTGGGTATGGTTATGCATACTTTGAGAATGATCTAGTGAAATCACAGTTTAGACACCGGAATTATGATGATTTCTTGATGATAATAAAAAGTGAACTACAGCAGTTCTTC

>PvRGL240

GGCAAGAAAGGTTTGAGAAAATGGAAGAAAGCAAAGGAAGAGTGTGTGTGACAGGAGGTACTGGTTTTATTGGTTCATGGATTATCAAGACCCTCCTTCAAGATGGTTACTCTGTTAACACCACTGTGAGAAACAATCCAGAACATAAGAAGGATCTTAGCTTTCTCACCAGCTTACCAGGAGCATCCCAAAGGCTACAAATTCTGAGTGCTGATCTCAGGAATCCAGAAAGCTTCAATGCAGTCATTGAAGGATGTGTTGGAGTGTTCCATGTTGCTACCCCAGTTGACTTTGAACTAAGAGAACCAGAAGAAGTAGTGACCAAAAGATCCATTGATGGTGCACTTGGCATTTTGAAGGCATGCCTGAATTCCAAGACTGTGAAACGAGTTGTTTACACCTCTAGTGCCTCTGCTGTGGTTCATGGTGGCACAGAAGAACAACAAGTGATGGATGAAAGCTCTTGGACTGATGTGGATCTTCTTAGAACTTCAAAGGCATTTGGTTGGAGTTATGCAGTTTCAAAGACATTGACAGAGAAGGCAGTGCTTGAATTTGGAGAACAAAATGGATTGGAAGTTGTGACTCTGATTCCAACTTTTGTTTTTGGACCCTTCATTTGTCCAAAGCTTCCTGGCTCAGTTCAAGCTTCATTGAAATTCTCATTTGGAGAAAAAAGTGGATTTGATTCCTTGCTTGAGACACCAATGGTGCATGTGGATGATGTGGCTAGAGCACATATATTTCTGCTGGAGAATCCTAATTCAAAAGGGAGGTATAATTGTTCAAAATGTTTGGTTACTTATGAAAGGATCTCTGAAATTGTTTCTGCCAAATACCAAGAATTTAAGCCAGAGACAGTAGAATGTTTCAACAAAATAAAAGGTGTGAAGATACCAGATTTATCATCAAAGAAGCTCATAGATGCTGGATTTGTGTTCAAGTATGGAATTGAGGAGATGCTTGATGATGCAATCCAATGCTGCAAGGAAAAAGGGTTACCTCTTAAGTAATTTGTACCCTTATTTATGTAATATATGAGGACGGTTATTCATAAAGTGGGACAAAAATTATCTCATTTGAAAAAGTTTCTTGTACATTATGCTTTTGTTTTTGGAATTATAATACTATGAAGTTCAATTACGTTT

>PvRGL241

CTTTTTTCATTTTCCTCCATTGCACATAGTTTAACTCCCACTTGGCATAGAGGAGCATCTTATAATTACTTCAGCTTTAGAACTGCTATGCACTGAAGATATTGAGGCAGAGGGTCTTAGGAGGGGTGTAACTACCCTGCTTGAAGTTTCATTAGCAATGGGAGAAGGGGTGGATGTAACAGCCGTGACTGGTTGTAATTCTACTTCAGGGGCATCAATTAATTGGTCATCTTCTTCAATGGACAACACAGTGCTTGTGTCTGTATGAATGGTGACGGAGTCATCAGGGAACATACCATGTCTTCTCTTCTTCCTAGCTGCCTTACCCCATCCATGAATTGTTTCTCTTATCCTCTGTGGGATTAATGCGGCCTTATAGTTTGTTCCCATCTGTGTAACAAGTGCATATAGTGGCAAGGTGCTGTAGCTACAAAGAAACTGTCCTGCAAACCCCAATATTAGCCTTAAATACAGGAGAAGGTGATTCCTAATAAAGCAAGAATCATATCCGAACTGCCACCAGAACCAAAAGAATGAAGCCAGCTCAAATGCATTCTGGAAGAGAATGAAATGGATCAAGGACAACAATAGTTCAGGCTTGTTAAACCAAAAAAGTTCATCACGAGGCCTCAACTTTGTCTCTGGAAAGAACCCTGCAATTCCAGCATTCTCCAACGCCAAGGTTGCAATAACATGTTGTAGCTTTGTTCCCACCAATAGCACTAGAGATACAGGAATAATAGCTATCCAGAAATATAGGTTAGATCCTTTAATGTTGAAAAGCATGAAGCCAACAACAAATCCCCAGAGCGGGCCACTCACACCAACTATCCTTT

>PvRGL242

GGAGAGAAAGAGAAAGAGCAAGAAGAAGAAGAGCAAGATCATCAACAACAGCACCATCAAGCAACACACCAACACCAACTGAAACGTCTTAGACCCATAATTCTCTCCCTCTTTTCTTCTTTTTGTTAATTGTGTGGGAGGCACAGAAAAAGAAGAAGATGAAGGAAAGCAGTGATGGGTTTGTGAGAGCCGATCAGATTGATCTGAAGAGCATAGATGAACAGTTGGAGAGGCACCTCAACAAGGTGCTCACCATAGACAAAAAAAAGCGCTCTGATGAGGACGAAGATGCTGATCAGGTTCATGTTCACACCAGTGCCACTGCCTCTCCCAAATTCAGACCTGCCTCTACTAGGCTAAGCTTGAAGAAGCAGAAGCAGGAGTGGGAGATTGATCCTTCTAAACTCATCATCAAGAGTGTCATAGCTCGTGGCACTTTTGGGACTGTCCATCGTGGTATCTATGATACCCAAGATGTTGCTGTGAAATTGTTGGACTGGGGTGAAGAAGGACAAAGGACAGAAGCAGAAATTGCTTCCCTGAGGGCAGCGTTCACACAGGAAGTTGTTGTTTGGCATAAACTTGATCATCCCAATGTTACAAAGTTTATAGGAGCAACAATGGGGTCTTCAGAATTGCAGATACAAACTGATAATGGTCTAATTGGCATGCCAAGCAATGTCTGTTGTGTTGTTGTGGAGTATCTCGCTGGAGGAAATCTGAAATCATTCCTAATAAAGAACAGGAGGAGGAAGCTAGCTTTTAAGATTGTCATTCAGCTGGCACTTGATCTTGCAAGAGGGCTGAGTTATCTTCACTCTCAGAAGATTGTCCATAGAGATGTTAAGACAGAGAACATGCTGTTGGATAAGACACGCACAGTTAAAATTGCTGATTTTGGGGTTGCACGTGTTGAGGCATCAAATCCTAACGATATGACTGGGGAGACTGGTACTCTTGGTTATATGGCTCCAGAGGTTTTAAACGGCAACCCCTATAACAGGAAATGTGATGTGTACAGTTTCGGCATCTGTTTATGGGAAATATATTGTTGTGACATGCCATATCCTGACCTAAGTTTCTCAGAAATTACATCAGCTGTTGTTCGCCAGAATTTAAGACCAGAAATGCCAAGATGTTGCCCAAGTTCTCTGGCAAATGTAATGAAGAAATGCTGGGATGCCACTCCAGATAAGCGGCCAGAGATGGATGAGGTAGTTTCCATGCTGGAGGCCATTGACACAACCAAAGGTGGAGGTATGATCCCCCATGATCAGCAGCAGGGTTGTTTTTGCTTCCGCAAGCATAGAGGGCCTTGAAGGTTTATTCGTTAGTCTTGCAAACAATTTGGTTATAGTGATTTTTCACTATTTGAAGAATCAATTTGTATCTATAGTGAGAAAGCAGTATTTGGATACTAGATGAGACAAATACACAGTAAGGGGTACAGGTAGGACGAGCTGGTGACATGTCAATATGTTGGGTGTGTAAAACTGTACCTCTGATTTTCTTTTACCTTTTTCCCCACATTGGTTTGGGTTCTGTGGGTATGAGTATTACTTTCTGCTTGGCAATGTTGCCGGATTTGACTTGTAACTTGCAGGATCTTCATTATACTATTACATACTCGCAATCATAATTGATGATATTAAGTTGGATCCA

>PvRGL243

CTTAAAACTGGCACTCCACCTGGTGGCGGCCGCTCTANACTAGTGGATCCCCCGGGCTGCAGGATATCTTCACCTCCAGGCAACACCACACATCATCCACAGAGATATCAAGGCAAGCAATGTGTTGTTGGATTCAGATTTTCAGGCACGAGTTGCTGATTTTGGTTTTGCAAAGTTGATCCCTGATGGGGCAACACATGTGACTACTAGAGTTAAGGGCACCCTAGGCTACCTTGCACCTGAATATGCTATGCTAGGTAAAGCAAATGAGAGTTGTGATGTCTACAGTTTCGGAATTCTCCTTCTAGAACTTGCTAGTGGGAAAAAGCCACTTGAAAAACTTAGTTCTACGGTAAAGAGATCAATAAATGACTGGGCACTTCCATTGGCTTGTGAGAAGAAGTTTAGTGAGCTAGCAGATCCAAAACTGGAGGGTAACTATGTGGAGGAAGAGCTTAAAAGGGTCGTTTTAATTGCTCTATTATGTGCTCAGAGTCAGGCAGAGAAGAGGCCCACCATACTTGAGGTTGTAGAACTACTGAAGGGAGAATCCAAAGATAAGTTAGTTCAGCTGGAAAATAATGAAATCTTTAAGAACCCTCCTGCAGCACATACTGATGATGAGAACTTTGCAGCTGAAGGCAGTTCAGATTTCATCTCAGAAGAGAAAGAATCAAAACCTGAGGTGGAAGACAAAACTGGGGCATGAACAACTATTTGAATGAGATAGAACTTTGAATTTGATGTGTTAAATACAGATTGTGAATCTGCGTGTTTTTTTTGTATTACTATATTTATTACTTGTGCAGGGAAAGACTGGGCTGGGCGCCTATTTATTAGAGAGAGGGGGCTGCTTGTCTCTCCTTCCCACACTATGAATGTGGCTAGCTTTGAATCATGCAGAGTTGCTGAAGTCTCAAACTAGGAATTTGTTAAATACACATTGTGAAGTTCTCAAAATTTATAACTTTTTGTTTGGAAACACTTTCTTTTCGCAGGGGTATAATGTATAGAGCCGTTGTCTATAGGTTGTTTTGTAATACTCATTACTCATGCTCATAAGTAAAAGTCATGCCATAAGCA

>PvRGL244

GGAGACAATAATATTATACTATTCTTGAAATGGAGAAGGAGAAGATAGTGTGCGTGACCGGTGCTTCTGGTTACATTGCCTCATGGATCGTCAAATTTCTTCTTCAACGTGGTTACACAGTCAGAGCAACGGTTCGCGACCCAGGTAATCCGAAAAAGGTGTCGCACTTGGTTAACCTTGATGGAGCAAAAGAGAGGCTGCATCTGTTTAAAGCGGATTTGTTGGAAGAAGGTTCGTTCGACTCCGTCGTGGAAGGCTGCGACGGTGTTTTTCACACCGCTTCACCTGTCCGTTTCGTAGTCAACAATCCACAGGCTGAATTGATTGATCCAGCAGTGAAGGGAACTCTTAATGTTCTCAAATCATGTGCAAAATCGCCATCTGTGAAGCGGGTTGTCTTAACTTCTTCTGTGTCTGCAGTTGCATTGAATGGAAGACCAAAAACTCCTGAAACAGTGCTTGATGAGACATGGTTTTCAG

ATCCAGATGTCTGTAGGGAATTAGAGTTGTGGTACTCGCTTTCAAAGACTTTGGCCGAGGATGCTGCCTGGAAATTTGTAAATGAAAACAACATTGACATGGTTGTTATTAACCCGACAATGGTAGCAGGAACTGTCTTACAAGCAGAGATTAATGAAAGCGTTGAGCCAATTTTAAACCTGATAAATGGCAAACCATTTCAAAATAAATCTTTTGGGTGGGTCGATGTGAAAGATGTTGCCAACGCTCATATTCTGGCTTATGAGATCGCTTCAGCTAGTGGAAGATACTGTTTGTCTGAGAGAGTCATACACTACTCTGAGCTTGCTACGATTTTACGTGATTTGTACCCTACATTACAAATTCCAGATAAGTGTGAGGTAGATGAGCCGTATATATCAACATACCAGATTTCAACAGATAAGGCAAAGAAGGAGTTGGGAATAGAGCTTACTCCTTTGGAAGTGAGTATCAGGGAAACTGTGGAAAGCTTCAGAGAAAAGAAGCTTGTCAACTTTTAATCCGTTATCTATTATTCTCCAAGATGACTGTGAAATAAAAGGTTAATATTGTTTGACAAAGGATAAACTGCATGTTATAAACTGTACAATGAAAACATTTGTGACGTAAAAAAAANAAAAAA

>PvRGL245

CATTGAGAGGGGGGTACTAGAAGTGAGAAAATTCCTTTCTGAGAGACTTACATTTTGATGAGGCACCTGCTATCTTCCCATGAAACAAAATTTGGCAACTACAACGGACCACTGCATATGAACAGACAAAAAAATCTACTTCCTAGTTTAAGATCTGTCTGTCACATGCTCCGTGCTTAAAATAAGTCTACGTTCTTCATCATCATCCCTTTGCATTACCTGAGAAGGTGGACTTCTTCTAGAGCTTTGTGCTGGGTGAAGTTGAGGATCATACTTCTGAGAAGCTAGGTAATTTAGAGCTGTAACAACATCAACTATAACAGGCCGCATATTAGGTTGCTCTTGCACACACATTGCAGCAATGGCAAGAGCTTGGTATAAGCCTCTCACGGGATATCGACCTTGAAGTAAAGGGTCAACCATCAAGGGAAATTTTTTCCTATCTCTGAACAAGGGTCTTGCCCACGCAATTGGAGTCGAGAGGAGCTTGCTCGAGTATACCCAAGCCCACCAAGAAATGAGTTGAACATATTGATGGCCGCAAACTCATGAACTTGTCCATGTCC

>PvRGL246

TAATAATCTTTCGTCAAATTCACTTGATTGGAGCAAGAGGTATAACATTGCCATGGGAATGGCAAAGGGTTTGGCCTATTTACATGAAGAGTGCTTGGAGTGGATTTTACACTGTGATATCAAGCCTCAAAACATACTTCTTGACTCTGATTACCAACCCAAGGTGGCTGATTTTGGCTTGTCCAAGCCATTGAATAGAAACAACCTCAACAACTCAAGCTTCTCTAGAATAAGAGGCACTAGGGGTTACATGGCACCAGAGTGGGTTTTCAACTTGCAAATCACTTCCAAGGTGGATGTTTATAGCTATGGAATTGTTGTGTTGGAGATGATAACTGGGAGGAGTCCAATGATAGGTGTCCAAGACACAGAACCAGGGGCAGAGTCGCATGAGAGGTTGGCAACATGGGTGAGGGAGAAAAGGAGGAAAGCACCAGAAGGGGCATCTTGGGTTGAACAAATTGTTGACCCCACTTTAGGATCAGATTACAATGTGAAGCAGTTGGAGATATTGGCAAAAGTGGCTTTGGATTGTGTAGAGGAAGAGAAAGATGTGAGACCTAGCATGAGTCAAGTCGTTGAGAGGCTCCAAAGTCATGAACATGGTTCTTAATTAATCTCTTTTGCACATTTCACTTTCCTATGAGTTTAGAAAAAAAGTGTTTATTTCTGTGGTTGAACACTAGAGTTAGATTAATAAGTGTTTGCTTAGTCTCAGTTCTACTTAATGTAAAACTATTCATCTTGATCTTGAAGTTACACTCTGATGTAAATATGATGTGTGAATTTGCAACCAATATGATTAGGGGTGATTATATTTTTACACCCGCTCCA

>PvRGL247

GATGAAGACAATGACTTTATAAGAAAATACCCCCACCAATTCTTCCTACTGTACAAAAACCTTTCAGAAAAATCCAAACGCTAACACCTTCAGTTAAAAATAAAAACCAACCCCAGAAGACAGACATAAAAAGAAAGTAAACGAATAAGCTATCATAAACCAAGTGATCTAAAATGAAACACCCAATAGAGCATTTTAAATGCTCAGAAGATCGGGTGGTGGAGATTGAGAAGGATTTTGACGATCTTTAGAGGCTGTGGATGGAGATTCTAAAGCATTGGATGATGACAAAGAGGATTCTGTTAAGTCTTCCTGTTTAGAACCAGGTGGCCTTGGAAGCTCTGTCAGTATTTGGACAACTTCACGCATAGTTGGTCTCTCTACAGCCTGTTCTTCAACACATAGCATGGCTACATAGAAAACATGCATCACATCGTGGAGGGGAACTGACGAAAGCCTAGGATCAAGAACTTTAAGAACTTCTTCCTTGTTAGAATCCGTCATTTTCCTTGCCCATTGCACAATGTCCACACCGTCACCAAATTCCCCAACTGGTTTCCTGCCTGTGATAAGTTCTAAAAGAACCACGCCAAAACTGTACACATCGCTTTTCTCATCAACTTTCAATGTGTAGGCATACTCTGGAGCTATGTATCCATATGAACCAGCAATAGCAGACATGCATTCAGATGTCCCAGAATCTTGCAGGAACTTGGCAAGCCCAAAATCAGCAACATGAGCTTCATGATTAGAATCAAGAAGGATGTTGTTTGACTTCACATCACGGTGAACAATGAGCGGAGAACAATCATGGTGGAGATAGCAAAGCCCTTTGGCAGCCTCCACGGCAATTTTATACCTTATGTCCCAATGCAAATGACCCCCCTTCTTTCCATGAAGAACCTCACCCAAGCTTCCATTGGGCATGTACTCATAGACCAAAAGGTTAGTCTCATGATTTGAACAGAAACCCAACAATCTAACAATGTGCCTGTGTCGGATTCGCCCAAGTGTCTGAATCTCTGCGTTGAACCCATGGTCGTGGGAAGAGCCTCTACTCATAGCCGGAAGCCTTTTCACAGCAACATGATCCCCGTTACGCATAGCCC

>PvRGL248

GGATTAGAAAAGAGTGTGTCTAATTTGTACAGCTACATGGCAAATGGAAGTCTCCATGACGTTTTGCATGAAAGGACACCAGTACCAACCTTAGAGTGGAATGTCAGGTATAAGATAGCTGTTGGAATTGCTCATGGATTGGCTTATCTCCACTATGACTGTGACCCTCCCATAGTGCACAGAGACATCAAGCCCAGCAATATACTTCTGGACTCTGATATGGAGCCTCACATTGCTGACTTTGGAATTGCCAAACTTCTGGAACAGTCTTCTGCTTCAAATACTTCCATTTTTGTGCCGGGTACAATTGGTTATATTGCTCCAGAGAATGCTTATACAACAACAAATAGTAGGGAGTCTGATGTGTACAGTTACGGGGTTGTTCTGCTTGAGCTGATAACCAGAAAGAAGGCAGTAGCAGATCCTTCGTACTTGGAGGGTGGTGTTGTGGTGGATTGGGTTAGGTCTGTGTGGAGGGAAACAGGAGAAATTCATCAAATTGTTGATTCATTCCTTTCTGACCAATGTGTAGATACCCATATAATGGAAAACGTTACCAAAGTGCTTATGATTGCTTTGAGATGTACTGAGAATGATCCACACAAGAGACCCAGAATCAGAGATGTTATTAATCAATTATCAGATGCTAATCCACAGACAAGAAGCACAAAGGGTATCTAACTATCTGTCATCATGCTACAGCTTCTACAGCTTTTGCAGCATGCATTTTACTCCTTCTCTCACCAATCCAATGCAAAATTAGCTAATGTTTAATAGTAACAACAATGGTAGCAGTGTTGTACTTTTTTTATATTTGAATCTTTGCTGCTAAATAGGTTATGGTGTGTGTGGTATCGTTGCTTACCTTGGTAACTTAATTTTGGTTTATTTGGAATGTTGGATGAATCAAAGTAATGTTCTTTAAGGCATCCACA

>PvRGL249

GAACCAAGTTTAACTTTTTATCCTTCTACTTTTTGCTTTATGATTCTTCTATTCCTAACCAAAATCATTTCAACTTGAAAACTTTCTGAGATCTCAGCTACTCACTGAGCAATCACTAGCATCAACCAAAAGGCCTTTTGTGTTTTTCTTTCTTCTGTTTTGGGTGTTTGCTTTTGCTTTTGCTTTTGCATTTGGAGTTTTTTGTTTGCATGCTTGTTTTTGTTGGGTTTGAAAGATATAATCCAAACCTTTTTGTATTGGGGTGATTTGAGTTGATGGGTTCTTGTTGGAGCAGTAGGACGAAGGCTGTGAGCCCTTCAAGTGCAGGGTTCACTTCTAGAAGTGTGAGCAGAGAGGGCTATGACATCCATTCAAGTAGCAGGAACTCATCATCCTCCATGCCCATGACCCCTCGGAGCGAGGGTGAGATCTTACAATCTTCAAATTTGAAAAGCTACAGCTACAATGAGCTAAGAATAGCAACAAAACATTTCAGCCCAGACAATGTCTTAGGGGAAGGTGGATTTGGTTCAGTTTTTAAGGGTTGGATTGATGAGCATTCGCTTTCTGTTACCAAGCAGGGAATAGGCATGGTTGTTGCTGTGAAGAGACTTAACCAAGAGGGATTCCAGGGTCACAAGGAATGGTTGGCTGAAATAAACTATCTTGGGCAACTGCAGCATCCTAATCTTGTCAAGTTGATAGGCTACTGCTTAGAGGATCAGCACCGGCTTCTAGTTTATGAGTATATGCCAAAGGGCAGTGTGGAGAATCATCTATTCAGGAGGGGGTCTCATTTTCAGCAGCTTTCTTGGACATTAAGATTGAAAATATCCCTTGGAGCTGCTAGGGGTCTTGCTTTTCTCCATAACACAGAAACTAAAGTCATATACCGAGACTTCAAGACTTCTAATATCCTGCTTGATACGAACTACAATGCCAAACTTTCTGATTTTGGGCTGGCCAGAGATGGACCAACTGGTGATAAGAGCCATGTTTCTACAAGGGTCATGGGAAACATGGATATGCAGCACCAGAGTATCTAGCAACAGGTCACCTTACT

>PvRGL250

TACACAGAAAAAGTTTTCGCCCATGATCTCATTGCCTTCTGAACTCAAGCTATACATAACTGTAAAACATGCATGTATTACATACACCGTCTCCTCTTTACTAATTCAGAACAGATATTCAGTAATAAGAAACCGAATAAGCAAAAGGTCTCCAGATAACATTTTCATTTTTTTTCTTCAGAAGAAATAAATAAAACACACAAATACACAAAGCTATTATATAGTCACCCTTCACTACCAGAGTTTCATCTTGCCCCCAAATTTTCCAGTCTATGTTTATGTGACTTTTGCATTTGCATTCGCAGGATCAGTCAGCAGTCATTGGTTGAGTCATGCAACACCTGAACCGGGCCTTCCGAACTGTTGTCAAGATCTCAGTCTCCGCATTCTCGAGCATTCCAACAACTTCAGCAAATGGTGGCCTCACATCAGGGTTGGGATCCCAGCATCTTGTCATGATGTCACGGAGAACAGGTAGGCAATCATTGGGTATGATGGGGCGAACATTTTTGTTGACAACTGCAAATGCTGCCTGCACTGCTGTCATGTTCTGAAATGGAAGCATACCGGTGATAAGTTCCCATAGAACAATTCCGAAGCTATATACATCCACCTTTTGTGTGTAAGGCCTGTGCTGGATCATCTCCGGAGCCATCCAACGGTATGTTCCAGTCTCAGGTGTCATTCCTTCAGTTTGCACCTCAATTCGAGCAACTCCAAAGTCAGCAATTTTAATTGACTTGTCACCAAAAATCAAAAGATTATCAGATTTCAAGTCCCTGTGGATCAATCCGAGGCCATGAACATAAGCCATCCCCCTTGCAACATCCAAAGCTTGCTTGACAGCCAATTTGAGAGGGACTGATCGCTTTTGACGCTTCATTAAAAATTGTCGAACTGAACCACCTTTGGCATACTCGGTTACAATGCACCACACCATTGGCTTGCGGCATGCGCCAATGAAACGAACTATGTTAGGATGCTTTAGTGTAGCCAACATCATGACCTCCTGCTGGAATTGTTGTTCCATCAATTGAGCCTTTGCTGGATCATTTTCGGGCCTCTCCAATATTTTGATAGCAACATCTTCACCATTGTAAGTACCTCTGTAGAGTTTCCCAAAAGCTCCCTGCGCAAAAGGCTCACCCATATTGAGTTTCCTCAAATCAATTGTCCACTCATCAAAATTGTCAAGCCCTTCAGTAGGAGAACTGTTGTCCATTAGAGCTTGAGCTAAAGCATCATCACTCAAAGCATGGGTAACTCTTCCTCGACGATTGGCACTATGTGCAACAGAGTAGTTGTCATTGGCACGCCTCCGCAACCCTTGGTGGTCCAACATGCGAGTATGGGAATCATTGGACCCAACACTGCTATTATCAATAGACATTGCAACAGATCCTCCG

>PvRGL251

TATATAAAAAACAAAAAGATCTTCAAACTTATCATTGAACACAAGCCCTCATATTTACAGAACAGCACATAGCAACTCCCTAGTCATGTAGGATTACATCCAACACAAAAAAATCACTCAAAAAAGTATGAAAATAAGTTGCAAAATTTACATGAGATACATATCTAAGCAATCAAAATAATTTTTTGATTAAAGCACAAATCACCATCTGTTGATATTCACAAGCTTTCATCATATATTGCAGCGAAATTTGTAGTTGTGGCAGATGACATGGTAGTGTCATGGTTGTCTGAAGAAGAGGTTGTATTGTACATAGATGAATTCTTCACATCACTACTACTTTCTTCGTGCGTTCTTACTTTGAGGTCCATAAAATCAGAAATTAAGGCAGGTTTTGTTATCTTACTGTCATCAACATTCATTTTTCCAGTGAGCATCTTGACCACAGAAGACATGGATGGCCGGAGCTTTGGCGATTCCTGAGTGCAAAGAAGACCAATCTTCATAAACTCACAAGCCTGCTCGGCGTCGAATTCGTCCAGTGATGTATCTACCAGCCCAACCAATTCCTTTCTCTCATAAAGTTCCCAAGTCCTCTCTAGAAGAAATTGTTCTTCTATTGGTAATCGCGAGTTTGTGTTAGATCTTCCACTGACTATCTCCACAAGGAGGACACCAAAGCTGTAAACATCTGCTTTCCTTGTTAGCTTCCCTCCTATTGCATACTCGGGTGCCAAATAACCTATTGTTCCTGCTACTCGAGTGCTGACATGAGTCATATTTGCTGGAATAAGCTTTGCAAGACCAAAATCTGAAATTTTGGGTGTAAGATCTTTGTCAAGGAGGATGTTGCTTGCTTTTATATCCCTGTGAACAATATGAGGCCTTACTTCTTCATGAAGGTAAGCCAGTCCACGAGCAATCCCAATACTTATTTTACATCTTGTTCGCCAATCAAAGAAGATCCTACTATGACTTCCACCTAGAAGAGTTTGCGACAGGCTGTTGTTCTCAAGGTAATTGTAGACTAATATTCTTTTATTTTTATCCACACTACAACCATATAACTTGACCAAATTTTCATGCTCTATCTCTGAGATCACATTAATCTCTGTCAAGAACTCCTTCACTCCTTGTCTTGAATCAGCTGCAAGAACTTTTATTGCAGCAACTTTTCCATCTTTAAG

>PvRGL252

ATCATCAAATTGTCTAGGACTCAACAAATTTTGTATATAATAAATCCGAATTCCAAAACTTTTCAAACACCAATTTATGTTAACAAAACATGATGCCATGTGACACTTTTAATATAGTGTTAGATTGCACAGCAGAAAGAAGATGCAGAAAAAACACATCAGACCTCAAGAATTAAAGAGATTTAAAACAAATACAAAAAAGCATTGTCACTGCAACATAAGAACAATTCAAAGATTGTTACTGATGCCACTGTCAGTTGCTTACACAAAGCTCCATGACATGTATATTTTGACAGTCTTTCATTCATTCCTCTCAGCAGCTGTCGTTGGAACAAGGAAAGGACTTGCTATATGCTTCCCATCTTCAATATTAATGAGCTGTTCCACAATCCTTTCGATAGATTCATGTTGGAACTCATTTTCAATGAGATGCTCTCCATCCAAGTTGGTGATCTGAGACAAAGCAGACTTCCATTTCTGCACCTTCTCAGACTCCTTTCCAAACATTTTTCCATGTGCAGTCATGGCCTCACCATAACTTTTTGTTTGATTGCACACATCCATCTTCTCCACATTGTAAAATATTGGCCAAACCTTCTGATTCTTGGTGTTCATACACTCAGTGATCTTGGCAAGTTCATCAAGACACCAAGTGGAATATCCATAGTTTTCAGAGAACACAACAATTGAAATCCTTGAACTTTCAATTGCTCCCATGAGCGTTTCAGAAATTTGGTTCCCACCCTCCAATTCTTCATCGTCCATGAAGATTTTGAATCCCTCTCGGCTAATGGCATTATAGAGAAAACCTGCAAGAGTGTAGCGGGTATCCTCTCCACAAAAGCTCAGAAAAACATCATATCTTGGCAGGGATTGCACAGCTGCTTTCACGATCTTTCGGATGAATTTATATTCGTACTTGAACCTCCTTTGATAAACCCATCCACTTAATTGGCCAACTTGAACCAAAGCTTCTCTCCATTTGTTTATCCTCTCTGTGGAATATTTTTCTTCAACTCTGCACATGGCCTGTCCGAATCTACCTTCGTGAGTTACTAGGTCACTCGGATCCACTCCATAATAGATTGGCCAGACAAGTTGGTCGCTCTTCATCCTACAGTCAAGGATGGCCACAAGTTCATCGAGACATCTTGAGGAGGATGCATATTGTTGGGAGAAAACAACCATTGAAAT

>PvRGL253

TGGCTCAAGGTGTTGGCTTCTTTCTTGAAGATCTTGTCAGTATTGGCAGAGAATTGTTGCAAATTCAACCTTTTTATGGCAACAACCTGGCCATCTTCCATTTGGCCCTTGTAAACTGTGCTCAAACTGCTAGCACCAATGATGCTGTCCGCACTGAAAAATCTAGTGGCATTCTCCAATTCCTTTGGACTGAACCTTTTAAGTGCCAATGCTGAGTTGTAGTCTGGTCCATGATTTA

>PvRGL254

GGGTATGCAGGTAATGCAATCCTCGTGCAACACCTATGCATATTTCGACCCTTTGCTTCCACGGCAGGGACGGGTTATCGGTGTAGTAGAGATGGTCACGGAGGGTTCCACGATCCATGAAATGGTAAACGAGTATCATCTCGTTGCTCTCGTAGCAGTAACCGATGAGAGAAACAAGATGGAGATGACGAAGTTGAGAGAG

>PvRGL255

AACATTGCCAACAGGAGAGGAAGTTGCTATAAAAAGACTTGCAAGAAGTTCTACACAAGGAATGGTTGAGTTTAAAAATGAACTAACACTTATATGCGAACTTCAGCACATGAATCTTGTTCAACTACTTGGTTGTTGCATTCATGAAGAAGAGAAGATTCTAATTTACGAGTATATGTCCAACAAAAGCTTAGATTTCTATCTTTTCGATTGTACAAGAAGCAAATTACTCGATTGGAATCAGCGCTTTAGCATAATAAAAGGGATTGCTCAAGGATTATTGTATCTTCACAAGTACTCAAGACTAAAAGTCATTCATAGAGACTTGAAAGCTAGTAACATACTTCTTGATGAAAATATGAATCCAAAAATATCAGATTTTGGAATGGCTAGAATGTTTACTCAACAAGACTCGATATCAAATACGAACAGGGTTGTTGGGACATATGGTTATATGTCTCCAGAATATGCTATGGAAGGAATATTTTCTACGAAGTCTGATGTGTACAGTTTTGGAGTATTGTTGCTTGAAATAGTTAGTGGAAGAAAAAACACAAGTTTTTATGACGATGATCACCCTTTGAATCTAGTTGGACATGTATGGGAGTTGTGGAAAGATGACAAATATTTTGAATTAGTGGATCCATCACTAAAT

>PvRGL256

CCCACGCGTCCGCGGACGCCTGTGTTAGACTTCTCAGAATACCAAACTGGAACCATAAAGAGACCCTTTGGCGTTGGCTTACTTACTCCTTCACCAAAAAATAATGAAGTTCTAATGGAGGACAGAAAAGCCAACATAATCTCTTTGATATCAGTGATTGCACTGATCATATTCATCATGGTTGCACGCGTCTCCTTGAAGCTTTCACATGCCTTCTTCCTCGTATGTGGCGCTTCTATTGCTGTCATCATCGCAGTCTTTTCATGTTCACTCATAAGGCACCGTTACAACTGCAGGAGGAGGTTGTTGGAGTCACAGTTGAAGACGGAAGGGCGAGAGCTTCGAATAGAGTACAGTTTCTTAAGAAAGGTTGCTGGGGTTCCCACGAAGTTTCGTTGCGAGGAGCTTGAGGAAGCAACGGATGGGTTTCAATCACTGTTGGGAAGAGGGTCTTCAGCTGTGGTCTTCAAAGGCATTCTCAGCGATGGAACTTCGGTTGCAGTGAAACGGATCGATGGAGAGGAGCGTGGGGAGAAGGAGTTCAGATCAGAAGTTGCAGCCATTGCTAGCGTGCACCATGTCAACCTCGTGCGCATGTTTGGTTACTGTAACGCCCCAACAGCGCCTAGGTACCTTGTTTATGAGTACATCCCGAACGGATCTTTGGATTGTTGGATCTTTCCCCCAAGGGAAAACCGTGCACGTAAAAGTGGGTGTTTGCCATGGAACACGAGGTATAAGGTTGCGATTGATGTTGCCAGGGCGTTGTCTTATCTTCACCATGATTGCAGGAAGAGGGTTTTGCACCTAGACGTAAAGCCCGAGAATATACTCTTGGATGAGGATTATAAGGCTCTTGTTTCTGATTTTGGTCTCTCAACGCTTATTGGCAACGATGTGAGTCAGGTCATGACGACGATGAGTGGGACAAGAGGGTACTTGGCTCCTGAGTGGCTTTTGGAAAGAGGGGTCTCAGAAAAAACTGATATTTACAGTTATGGGATGGTTTTGTTGGAAATAGTTGGAGGGAGAAGGAATGTTATGAGGGTGGAGGATCCGAAGGACAGGACTAAGAAAAAGTGGGAGTTTTTTCCCAAGGTTGTTAACGAGAAAGTGAGGGAGGGGAAGTTCATGGAGATTGTGGATCGTAGGTTGATTGAAAGTGGGGGTATTGAGGAGAGTGAGGTAATTAGATTAGTGTATATTGCATTGTGGTGTATACAAGAGAAGCCAAGGTTGAGACCTAGCATGGCACAAGTGGTTGATATGCTTGAGAAGCGTGTGAGGGTGGATGAACCCCCTGGTTCCAGAATGATCCTTGTTGATATACTAGCTGTTGATGAAGATCCTGTAGATCATCGAAATCTTGCAAGGTTGTTGACCTCAGTTTCGAGCAATGTGGATTGTACATCTACTTACTCCCTGGGAACCACTATTTTATCTGGCAGATAGCTAGCTCTCACTAGTAACTTTTCTTGGGCATTAGAGATCCTAATGCACTTCAATCTATCTGCTCCAGGCTATTATTCCCTTCCTATTTTTTCTTCTTATTACTTAGTTACTTCAATTGTATAAATTGGGACATAGTTCTAATATATCTGTGAGGAAATTTAT

>PvRGL257

CCCACGCGTCCGCCAATTCCATGTGAGTCAAGAACTTCTTCCACTCTTTTATCCTACGCACTTCTCCCTGCTTATCCTGCGCCTCCTTTGCGAGATCCTCCCCTTGCACCGTCACTCTCCGCGCCTCTACACTCTTAATTCCGCTATTTTGTCATCTTATTTCGTTTCAGTGAGAAAGAATCATGAGTTGTTTCAGCTGTTGTGAGGAGGATGAACTCCAGAAGTCTGCTGAAAGTGGAGGACCCTATGTAGTAAAAAACCCAGCAGGAAATGATGGAAATTATCATGCTTCTGAAACTGCAAAGCAGGGCACTCAGCCGGTTAAAGCTCAGCCCATTGAAGTTCCAAATATACCAGCAGATGAACTAAAAGATGTTACAGATAACTTTGGTCAAGATGCTCTGATTGGAGAGGGGTCATATGGAAGAGTATATTATGGTGTTCTTAAAAGTGAGCAGGCTGCCGCAATCAAGAAGTTGGATGCCAGTAAACAGCCTGATGAGGAATTTTTAGCCCAGGTTTCAATGGTATCAAGGCTGAAGCATGAAAATTTTGTTCGGTTGCTTGGGTACTGCATTGATGGAAGCTCCCGTATTCTTGCTTATGAGTTTGCATCTAATGGATCTCTTCATGATATTTTACATGGCAGAAAAGGTGTTAAAGGTGCACAGCCTGGTCCAGTTTTGTCATGGGCACAAAGAGTAAAGATTGCTGTAGGGGCTGCAAGAGGACTTGAATACTTGCACGAGAAGGCTGATCCCCACATTATCCACCGGGACATCAAGTCAAGCAATGTGTTAATCTTTGATGATGATGTTGCTAAAATTGCAGATTTTGATTTATCAAATCAAGCTCCGGACATGGCTGCGCGTCTTCATTCTACTCGTGTCCTTGGAACCTTTGGTTATCATGCACCAGAATATGCAATGACTGGACAATTGAATGCAAAGAGCGATGTATACAGTTTTGGCGTTGTCCTTCTGGAACTTCTGACTGGAAGGAAACCTGTTGATCATACACTGCCACGTGGACAGCAAAGTCTGGTTACTTGGGCTACACCAAAACTCAGTGAGGATAAAGTCAGGCAGTGTGTTGATACAAGACTAGGAGGAGAATACCCACCCAAAGCTGTTGCTAAGATGGCTGCTGTTGCTGCACTTTGTGTGCAATATGAAGCTGATTTCAGACCAAACATGAGCATTGTAGTCAAAGCTCTTCAACCTTTGCTGGCTGCACGACCTGGACCTGCCGGTGAAACACCAAATTAGTTCTCTGTTTCTCTGTCTCTATATTTGTGAGTATATGCACAAGTTTGCATGCAAAATATAAAAGTGCTTCAGAAGATGGTGGTAAATTGCTCCAACATGACTTTATATTCAATTGTTTACACTGCATTCATTTTGTTCATAATCCATGAGGGTCTTTGCTTATATTCATTTTAATGTATTTGTTTTATAAGTT

>PvRGL258

CCCACGCGTCCGCAAGAACCTGAATTGGCTCTACCTGTCCAACTGCACCCTCGGAGGGAAACTTCCGGTGGGACTCGGCAACCTCACGGAGCTCACGGAATTGGAATTCTCCGACAATTTCATCACCGGCGAACTTCCCGCGGAGATTGTGAATCTTCGGAAACTCTGGCAGTTTGTGTTTTTCAACAATACCATCACGGGGAAGATTCCAACCGGGTTTAGAAACCTCAAGGGGCTTGAGTATTTGGACGGGTCCACGAATAGACTCGAAGGGGATCTCTCAGAATTGAAGTATTTGACCAATTTGGTTTCCTTGCAATTTTTTGAGAACAATTTATCGGGGGAAATTCCGAATGAAATAGGCGAATTCAAACGCCTCCGGTCTCTGTCTCTGTACAGGAACAAATTGACAGGTCCTATTCCTCAGAAGGTTGGTTCCTGGGCCGAGTTTGAATTCATCGACGTGTCGGAGAATTTATTGACGGGGACTATTCCGCCGGAGATGTGTAAAAAGGGGAATATGAACGCACTGCTCGTGCTTCAGAACAAGCTCACCGGCGAGATTCCGGCCACCTATGGAGAGTGTTGGAGTCTGAAGCGATTAAGAGTCAGCAATAACTCGCTCTCGGGTACCGTACCTCCCGCGATTTGGGGATTGCCCAACGCCGAAATTATCGATATTGAGCTGAATCAGTTTGAAGGTTGGGTTGCTTCGGATATCGGAAACGCCAAAAAATTAACTTCAATACTCGCCAGGCAGAATCGGTTGTCCGGTGAAATACCCAAAGAAATCTCAAAAGCAACGTCGTTGCTGAGTGTGGAACTGAGCGATGGATGCAAGATTTCAGGGATGAGTTGCAGAGGGAATCGGGGAGCTTAATAGATGTAGGTAGCCTTGAAGGGTGTAAGATCTATAGGTGACCAGGCTCGATAGCCACTTGACAGCTACCATGATGTGCACTGCAAATCCTCTCAACTCTTACCTTCTGGAACGCCTTTCTGAAGAGGATTCACTTCTCTCTGTTTGTGAAAACGGCTTTTAAAGAAGGGAAAGAGAAAAGCTTTCCACAGTTGTTGGAGATTGGGAAAGAAATTGTGAACAAGTGTGGAGGAATACCGTTGGCAGTGAAAACTTTAGCAAGTTCACTATTCTCTGTGGTTGATAAAACCAAGTGGGAGTCTATGAGGGACGACAAGATTTGGAATTTGCCACAAAAAGAAAGAGACATTTTGCCTGCTCTTGAAATAAGTTACAATCAACTTCCTTCACATTTGAAACCTTGTTTTGTTTGCTTCTCCCTTTTTTCTGAGGGTTCTGAATTCTTTAGTTTTTATGTTACTAAGTTGTGGGAGGCACTTGGTTTTCTTCCACCACCAAATGAAAATGAGACAATGGATGATGTTGCCATTCAATTTTTGCATGAGCTGTGGTCAAGATCTTTTCTCACAGATTTCACTGAATTGAGCCATGGTTACAGTTTTAAATTACATGATCTAGTTCATGATCTTGCTATGTATGCTGCCAAAGGTGAGTTTCAAACAATATACCCTCGCAGTTCAATAATATCTCCCAATGCTCGACATTTGGCATTCAGTGAGAATAACTTGCTAGACCAAGCTGTCATTCCCACAGGTCTGAGAACTATAATTTTTCCTGATGAAGCTTCCAATGAAGTTNTCATGAATACATTGGTGTCAAGGTGC

>PvRGL259

CCCACGCGTCCGCTAGTCCTTGATCGCGAGCGGCCCCTTTTTTTTTTTTTTTATGATAAACAACTACAAAATCTTTATTGAAAGTTGAACTAAAGATTTCACAATGTTCACAATTTTAAGTAGTGAGTGAGTTCTTGACTCATAAGTTGCTGCAATGAAATATGATGTAAAGGAATGTTGAATGGTGTGAGCCGAGTAAGAAAACATTGAGAGATGCATTTCATTGTTGGACGAGAGCAAGGATTTGGATTTAAGCATGCAAAGGCCACAATGGCAACAACAACTATGTCTTGTAAAACTGAAACGGTTGGGTGTGGAAGACGTTGGTCCAATATTTCACATACTGTAGAAGCTTGTTTGAAGTGATGAGAGTATATTCATTAGCCGATGTTTTCCCATCAAAGTTTCACAGTGCCACCACTCCGAAACTATACACATCACACTTTTCATTCACAACCGTATGTATACGCATTTAGCTCTGGAGCTATGTATCCAATGGTTCCAACAACTATAGTTTGATTGGATGAATCAAGGTTCAGAAATCGAGCTGTACCAAAGTCAGAAACAGTAGGTTCCCAATTGGAGTTAAGCAAAACATTGCTTGTTGATACGTCTCTATGCACTATTGGAGGGACACAATCATGATGAAGATATGAGAGAGCATGTGCAGTGCCTTTCACGGTGTTAACCCTCTTTATCCAATCCAATTCCATTGCTCCCATGTCATCAAACAAGATAGAAAGCATGCTTCCTTTTTCCATGTACTCATAGATCAAAAACACAATTCTTTTGTGAAAGTAGAATCCGTGGAGCTTGACGATATGTCGGTGTTTTATTTCTGACAATACTTTGACTTCGTTTTTGAAACTCCCATCAAATGTGGGGACCTCTGGCTTCAAAGCCATGGGAGTTTTTTTCACTGCAACAATCTTACCACTTGGTAGTTCAGCCCTATAAACACTCCCGTACGCACCTGTTCCGATACAATATTTGATGTCAAAGTCTTCAGTTGCTGTAATGATGTCTTCATAGGCTATGCTTCCG

>PvRGL260

CGAGATTCTTCCCTCTTCCTTCCTCTCTCTCTAGAACTTCGTCTTCTACTTCCTTCTTCTTCTCTGCGCGGTTGTTGCGAACCTACGAATAAGACCGAGAACCCTAACAAGGCGAGGAGAGGTGTTCGGTTGAAGCTCAGATCAACCATGGCAGCAGCAACTTGCACTTCCATGCGTTTCCTCCTCTCCGTTTCCGTAACCCTAACTCGTTGCTCCGCCTCGGGCGCCTCCAATTCCGAAGGCGACGCGCTCTACACGCTCAAACGGAGCCTCTCGGACCCGGACAACGTCCTCCAGAGTTGGGATCCCACGCTCGTGAGTCCTTGTACTTGGTTCCACGTCACCTGCAACCAGGACAACCGAGTCACTCGAGTGGATCTTGGTAACTCTAACCTATCTGGACATTTGGTACCTGAACTTGGGAAGCTCGAGCATCTGCAGTATCTTGAGTTGTACAAGAACAACATTCAAGGAACTATTCCTCCAGAACTTGGAAACCTGAAGAGTCTAGTTAGCTTGGACTTGTACAACAACAACATATCAGGCACCATTCCGTCTTCATTGGGGAAATTGAAGAATCTTGTATTTTTACGACTAAATGACAACCGACTCACTGGCCCTATCCCCAAGGAACTTGCTGCTGTTTCGAGCCTTAAAGTAGTGGATGTCTCCAACAATGATTTATGCGGTACAATTCCTACCTCTGGGCCATTCGAGCATATTCCATTGAATAACTTTGAGAATAACCCTCGTTTGGAAGGTCCAGAGTTGTTGGGTCTTGTAAGTTACGATACAAACTGCTCGTGAATAACGTGATGTGGTAGGATCTCAATATATATATTCTAAAATTTAAGTGTATTACCTCATAGTGTAATGGGCAGTGGTAAATGTGATCTCTGGTATATGTAAATTTACATACTGAAGAGCATTGGCATCTGTAACAATATTATGATCTGACAATGATGTTGAGACTTGAATATCTATTGATGTAATACTAG

>PvRGL261

GTTGTGTCTTTATAGGTAAGGACAGAGGCTGTGTGACCGAGGTACTAAACGGCTGTGGACTATATGCTGATATTGGAATAACAGTCCTCTTAGAGCGTAGCCTCATAAAAGTCGAAAAGAACAACAAACTCATAATGCATCCTTTGTTACGAGACATGGGAAGAGAGATTATTCGTGAAGGTTCAAGAAAAGAACCTGGGAAGTGCAGTAGATTGTGGTTTCAAGAGGATGTACGTGATGTATTGACAAATAGTACTGGGACAGATGCTGTTGAGGGATTGGCTCTGAAATTGAATTTAACCAACAGAGAATGCTTTAAAGCAGATACTTTTGAGGAAATGAGGAGTTTGAGACTGTTGCAACTTCATCATGTAGAACTCACGGGAGATTATGGCTATCTTTCTAAGCAATTGAGATGGATCTATTGGCAAGGGTTTCCTTCAACATACATACCTAACAACTTCTATCTGGGAGATGCAATTGCAATTAATTTGAAACACAGTAATCTTAGACAAGTATGGAAAGAACCCAAGGCTTTACACATGTTAAAGTTCCTCAATCTTAGTCATTCCAAGAACTTGACAGAAACCCCTGACTTTTCTGGACTACCAAATCTTGAAAAGCTCGTTCTCAAATATTGTCCAAGTTTGTGCTGTGTACACAAATCCATTGAGATCTCAGTAATATTGTGTTGATAAATTTGAAGGAGTGTACAAGCCTAAGCAATCTTCCGAGAGAGATATATAAGTTGAAATCTTTGAAAACTCTCATCCTATCTGGTTGTTCGAAGATTGACAAATTCGAAGAAGATATAGGGCAGATGGAATCCTTGACAACTCTAATTGCTGAAAATGCTGTTGTGAAACAAGTGCCCTTTTCAATAGTAAGCTCAAAAAGCATTGGATTTCTATTCCTACGTGGGAATGAAGGATTGTCGCATGATATTTTACGTCCTATTTATTTGGTCCTTGATGT

>PvRGL262

CGGCACGAGGGCAGCAGAACTTGTTGGTGGTTCTCTTCTTTCGGCCTTTCTTCAGGTTGCATTCGACAAGCTCGCTTCTCCTCAACTTCTGGACTTCTTTCGTGGAAGAAAACTTGATGAGAAGCTCCTTGGAAATTTGAACATCATGCTGCACTCCATCAACGCTCTAGCTGATGATGCTGAACTAAAGCAGTTCACAGATCCACACATCAAAGCATGGCTTTTTGCAGTCAAAGAGGCTGTCTTTGATGCAGAGGATCTTTTGGGTGAAATAGACTATGAACTCACCAGATCCCAAGTCGAAGCTCAATATGAACCTCAAACCTTTACTTACAAGGTATCAAATTTTTTCAACTCTACTTTCACTTCATTTAATAGGAAAATTGAATCAGGGATGAAAGAAGTCTTGGAAAAACTAGAATATCTTGCAAATCAAAAGGGTGCTCTTGGTTTGAAGGAGGGTACTTATTCTGGTGATGGATCAGGTAGTAAAGTGCCATCATCTTCTTTGGTGGTTGAAAATGTTGTTTATGGCAGAGATGTTGACAAAGATATAATCATTAATTGGCTCACATCTGAAACTGACAATTCTAACCAGCCATCAATACTTTCCATTGTGGGCATGGGTGGGTTGGGTAAGACCACACTCGCCCAACATGTGTACAATGACCCAAAGATTGAAGATGCAAAATTTGATATCAAAGCTTGGGTCTGTGTTTCTGATCATTTTCATGTTTTGACCGTCACAAGAACAATTCTTGAGGAAATCACAAATCAAAAAGATGATAGTGGGAACCTACAAATGGTTCACAAAAAGCTGAAAGAAAATTTGTCAAAAAAGAAATTTCTTCTTGTTTTGGATGATGTTTGGAACGAAAGACGAGAAGAATGGGAAGCTGTGCGAACTCCTCTTAGTTATGGGGCTCCAGGAAGTAGAATTCTTGTCACAACTCGTGGTGAGAAAGTTGCTTCTAGCATGAGGTCTGAAGTGCATCTTCTAAAGACATTACGAAAGGATGAATGCTGGAAAGTTTTTGAAAATCTTGCATTAAAAGATGATCTTGAATTGAATGAGGAGTTAATGAAGGTTGGTAGAAGGATAGTTGAGAAGTGCAAGGGATTGCCTCTAGCTCTGAAAACAATTGGATGCCTTTTACGCACAAAGTCATCCATTTCAGATTGGAAGAACATATTGGAGAGTGACATATGGGAGTTACCAAAAGAACACAATGAAATTATCCCTGCATTAATTTTGAGTTATCACTATCTTCCGTCTCATCTTAAAAGGTGCTTTGCTTATTGTGCCTTATTCCCCAAAGATTTTGGGTTTGTGAAGGAGGAGTTAATTTTGTTGTGGATGGCCCAAAATTTTCTACAGTGTCCACAACAAATTAGACATCCAGAAAAAGTTGGTGAAGAGTACTTCAATGATCTACTGTCAAGGTCTTTCTTTCAACAATCAGGTGTCCGGAGACGTTTCATCATGCATGACCTTCTGAATGATTTGGCAAAATATGTTTGTGCGGATTTTTGTTTCAGGTTGAAATTTGATAAAGGCCAATGTATACCGAAAACAACTCGTCATTTTTCATTTGAATTCCATGACATCAAAAGTTTTGATGGTTTTGGGAGTTTAAGTGATGCTAAAAGACTTCGTTCATTTCTTCAGTTCTCACAAGCTACGACTTTACAATGGAATTTCAAGATTTCCATACATGATTTGTTTTCCAAGATTAAGTTTATACGCATGTTATCTTTCCGTGGTTGTTCATTCCTTAAAGAGGTCCCGGATTCTGTAGGTGATCTTAAACATCTCCATTCATTAGATCTTTCATCGTGTAGTGCGATAAAAAAGCTACCTGACTCAATATGTTTGCTCTATAACTTACTAATACTGAAGTTGAACAAATGTTTCAATTTGAAGGAATTGCCCATAAATTTGCATAAACTTACCAAATTGCGTTGCTTGGATTTTGAAGGTACAAGAGTGTCANAGATGCCAATGCATTTTGGAGAATTGAAGAATC

>PvRGL263

CCCACGCGTCCGAAGACCGTTTATTGTATGTTATATGATGCTGGCGACGACGAGATTCGCTTAACTTCAGTTAAATTTGACTTGGATAGAGGAACTATCCCGAGAAGATTCGAGTACAAAGAGCTGGTGGATGCCACCAACGGGTTTTCAGATGAAAGAAGGCTCGGACAAGGAGCTTCGGGGCAGGTATACAAGGGTGTGTTGAGTTACCTAGGAAGAGTTGTGGCCATCAAGAGAATCTTTGCAGACTTCGAGAATTCAGAGAGAGTTTTCACCAACGAGGTTAGAATAATAAGCCGTTTAATCCACAAAAACTTGGTTCAGTTCATAGGGTGGTGTCACGAAGAAGGTGAATTTCTCTTGATTTTCGAATACATGCAAAACGGAAGCCTCGACACTCATTTGTTTGGAAACAAGAGGATGTTAGAGTGGCATGTGAGGTACAAGATCGCACTTGGCGTTGTAACTGCGCTTCATTATCTTCACGAGGACGCAGAGCAGTGTGTGCTACACAGGGACATAAAATCAGCGAATGTGTTATTGGACATGGAGTTTAACACCAAGGTGGGGGATTTTGGCATGGCCAAATTGGTGGATCCAAGGCTGAGAACTCAACGAACTGGGGTGGTGGGAACATACGGTTACTTAGCACCTGAATACGTGAACGGAGGAAGAGCTAGCAGGGAATCAGATATGTACAGTTTTGGAGTGGTGGCATTGGAGATAGCAAGTGGGAGGAGGACTTACCAAGATGGGGAGTTTCATGTTTGCTTGATGAATTGGGTGTGGCAGCTTTATGTGGAAGGGGAACTTCTGAGAGCTGCAGATGAGAAATTGAGGAATGAGTTTGATGAGAATGAGATGAGGAGTTTGCTTGTGGTGGGGTTGTGGTGCACAAACCCTTCTTGATCACTTGCAAGGTCAAATTCCTAAGGGCCGTTGTTTGCTTAGTTGGACACATCGCCTTCAAATTGCACGTCACACTGCTGAGGGTCTTGCTTACCTTCACTTCATGGCTGTGCCTCCTATCTACCACAGAGATGTCAAGTCCAGCAATATTCTCCTAGATATCAATATGTATGCCAAGGTTTCAGATTTTGGGTTGTCTAGGTTGGCTCAGACAGATATGAGTCATATCTCCACTTGTGCTCAGGGGACTCTTGGTTACCTTGATCCTGAGTATTATAGGAACTACCAATTGACTGATAAGAGTGATGTTTACAGTTTTGGAGTGGTGCTTCTTGAGCTTTTGACAGCTCAGAAGGCAATAGATTTTAACAGGGCACCAGATGATGTGAACTTGGCAGTTTATGTGCATAGAATGGTGGAGGAGGAACTGTTGGTGGATGTCATTGATCCAGTGTTGAAGAATGTAGCCACCACTATAGAGCTAGAGACTATGAAAGCAGTGGCATTTCTGGCATTGGGCTGCTTAGAGGAAAAGAGACAGAATCGCCCTTCAATGAAAGAGGTAGCAGAGGAGATTGAGTACATCATTAGTATTGCCTCAGCAAAGGTAGTGGAGTAGTTTATTTTGATGTTAATAATGTCGTCGAATTTTCTGAGTTTTTTTTGATTTGGTAACTTATGGTTGTGTTTGTTGTTGCTAGACTAAATTTTGAGTTCTAGACTTCAGGCTCTTGTTTTGCAGATGTATCATATCTATGTAAATGTATTATTAATTTATTACCCCTTTGCTCAAAAAACACATCAAACAAAAACCACAGTAAGGGCTCTGCGATGATGCCGATATTCCTC

>PvRGL264

AGAAATTGAAGGCAATGACAAACTGGGGGATATGAATAAAGTGCTTTCCCTAAGTTTCAATGAGTTGCCTTACTATCTTAAATCCTGTTTGTTGTACCTAAATATCTTCCCTGAGTTTCATGCAATTGAGCACATGAGACTAATTCGCTTGTGGATTGCTGAAGGTTTTGTGAATGGAGAAGATGGAAAGACAAGGGAGGAAGTTGCAGACAGTTACCTCAAGGAGCTCTTGGACAGAAGCCTGCTGCAAGTTGTAGCAAAAACCAGTGATGGCAGGATGAAGACTTGTCGTATGCATGATCTTCTAAGGGAGATTGTGAATTTGAAGGCAAAGGATCAAAACTTTGCAACAATAGCCAAAGATCAAGACATAATCTGGCCAGACAAAGTTCGACGCCTGTCAATCATAAATACATTGCATAATGTGCAAAAAATAGAGCTTCATTCCAACTCCGGTCTCTGCTACTGTTTGACTTATCAGATTCACTGGATAATTTTTCTATACGTGCATTATGTTCCACTGGTTACAAGTTAATCAGGGTCTTAGATTTGCAGGATACACCTTTGGAGGTTTTTCCTGCTGAAATTTTCCAGCCTTTACCTTCTCAAGTATCTGAGTTTGAAGAAGTACAAAGGTGAAAAGCATTCCAGGTACCTATTAAGATAGCTGCTAGCAGCTGGAGGACTCTAGATCTTAAACAAACCCTTGTCACTGTATTGCCAGTTGACAATTATGTAGCTGCAACAATTACGCCATCTCTTGAAGTATCGCTATGAGAAAAACACCTGTCATGCATTTCCATTCTTCCCATTCCTTCACGGCTATCAAGGTAAATGTCAACTTTTGGACTCAATGAAATCTTTGCAAATGCAGATGAGCTATTCCCATAAAAATCAACATCTATCCTCACTATATGATCAAGGCCGTCAATTGTCAACTCCTTGAGAAATGTCAAAAGTCCAAGGGAAGGCAACTGCACGCAATGTTTACAATTCTTCAAAGTTAAGGACACCACATTCAATAGAGATTTATCAGATAACCAACAGGGAAATTGTGTGCC

>PvRGL265

AACCTACTAACCTCTCTGAGACATGAAAACCTTGTTAGGGCTGAGAGGTTTCTGTTGCTCCAGAAGTAGAGGTGAATGTTTCCTTATCTATGACTTTGCCACCATGGGTAACCTGTCTCAATATCTTGACTTAGAAGATGGAAGTGGCAATGTGCTTGAGTGGTCCAAAAGAGTTTCTATCATCAACGGCATTGCAAAGGGCATTGGATATCTTCATAGCAATGAAGCAAGCAAACCTACAATAGTTCACCAGAATATTTCAGTGGAAAATGTTCTCCTAGACCATCAGTTTAATCCATTGATCATGGATGCTGGACTACCAAAGCTTCTTGCAGATGATGTTGTTTTCTCAGCTCTTAAAGTAAGTGCTGCAATGGGATACTTGGCTCCCGAATACATTACTACTGGACGATTTACTGAGAAGAGTGACATATACGCATTTGGAGTTATTATTCTTCAAGTTCTATCTGGCAAGACGACAATAGGTAGTTCAATACGGACAGCAGTTGAGTCTTTCAGATTTGATGATTCTGTTGACGCAAATCTTAGGGGAAGGTATTCTAAATCTGAAGCAGCCACCCTTTCAAAGCTAGCAATACAGTGTACTCATGAGTTTCCTGACCAAAGACCAACCGTGGTGGATGTGATTCAGGAGTTGACTGTGTCTTCTGCTCATTTTTAATCATTTACTACTACTGTCTATTGGTATTAGGAAACGTTTAAACACTTCCCCATAGTTGGGTTTCATACATGCTACTATTTTCATCAGCATATATATACTCTAAAACTAGCCATTGCCAAATAATTGTGGATCTAAATGGATCCCTTTCCTGTTATTACGAACTAGGATAGTTGAAATGTGTTCTTAATTC

>PvRGL266

GACCTTTCTGATATGAAATTCATAAATCAACTTTGTCTCAGTGGTTGCTCAAAGTTGGAAAATTTACCCGAGATTGAGGACACGTTGGAAGATCTAAAGAAGCTCAACTTAGACGGCACAGCCATACAGGCACTACCTTCATCCTTGTGCCGTTTGGTAGGACTTGAAGAACTGAGTTTGCGCCGTTGCTGCAATCTTGAGATTATTCCATCTTCCATTGGAACTCTCACCAGACTATGCAAGTTAGACCTTACCCACTGTAATTCACTTCAAACTTTTCCAAGCACCATTTTCAATTTGAAGTTGAAGAAGCTTGATTTGTGTGGTTGCTCAAGGCTGAGGACCTTCCCAGAGATCACAGAGCCGGCTCATACTTTTGCTCACATTAACTTAACATGTACGGCTGTTAAAGAACTGCCTTCCTCATTTGGCAAATTGGTTAATCTTCGATCCCTGGAGCTCCCCAAATGCACTGATCTCGAGTCACTTCCAAACTCCATTGTTAATCTGAAGCATCTCTCTGAACTTGATTGTTCGGGGTGTGCCAAATTAACAGAAATCCCAACACACATCGGTCGCTTGACGTCATTAGTGGAACTGTCACTGCGTGATAGCGGAATCGTGAACCTTCCTGAGAGCATTGGTCATCTTTCACGCTTGAAATTGCTTGATTTAAGGGACTGCAAAAAGCTTGAATGCATCCCACCGATTCCACCATTTCTACAACAACTCGTGGCATTGGATTGCCCATCCATTAGAAGAGTGATGCCAAATTCTCTTGTTCTAAACTTGTCAAATTCCAAAGAGGGTGTCTTCGGATTTTATCTCACCAATGCTCAACAACTGGATTCAGGTGGTCGTGCTAACATTGAGGAGGATGCAAGGCTAAGGATGACCGACGATAGATATAGGTCTGTGTTCTTCTGTTTTCCAGGGAGTACAGTTCCTGGTTGGTTCCATTTTCGTGGGAAGGGAGATTCAGTGACTATAAATGAAGATTTAAGTTTCTGCAGCAATGATAGGCTCACCGGTTTCGCTTTGTGTGTAGCTTTTGGAGTGTTAGATACGAATTATGTTAGCAGTAGATATGGTCTTTTTCGTTACAGCCTAAAACTTGAATCTGATGATGATGGCACA

>PvRGL267

AATTCATATGTAACAACTATTTTAGAATATAATAACATGTCCTTGTATTAGCAAATTTCACTACAAAATACAAAATAAATAATATAAACAAGTAAAATATCATGGACAACCAAATGTTTAGAATAAGAAGATGGATGCAAATAAGCAATGTTGTGTACCGTCGAATAGTATATACTTTACCAGTAAATAATTTCAACATTGAATATATCTAGCTAGAGTTGATTATTTGTTCCTTTGTCACTGTTTCATCTGGAAATAGAATAGGTTTTGGAGGGATTTCCAAGTCTTCAATATCCCCTTCAAGCATTTCCACTACTCTATTCATTGAGGGTCGATCATTTGGTTTTAGTTGTATACACCATAATGCAACTATGATCATCTTCTTTGCTATTTTATTTTCCTCTTCTGTCACATCTTCGATATCTACATCTTCCTCTTCCCTAATATGATCATAGATCCAAAGGGGAAAGTAAAGTTGACTTGAGTGGTCTGCATAAGGATTTAGATTCTTCCTCTTACTTGCCATCTCCATCAACAACATTCCGAAGCTATAAACATCAACCTTATGAGATATTCCTCCAATATTATTATAAAACAATTCTGGAGCCATATATCCAATGGTTCCTCTTGCTGCAGTCATTGTGACAATACTATTATCTATCGGATATAACTTTGCCAATCCAAAGTCAGAAACCTTAGGGATGAAGTTTTCATCTAGTAAGATATTATGGGGTTTGATGTCAAAATGCAAAATTTGCATCTCACACCCATGGTGGAGATAAGCAATCCCACGAGCCACTCCGATTGATATATTATATATCTTTTCATAGCTTAAATGTATATTTATCTCTTTTGAGAAAATAAATTTATCAAGAGATCCATTGGGCATGAATTTCATAGACAAGAGCACGTTTTGAGCCACTAACACAAAATCCAATTATACTGTACCACATTTTGATGATGTATTCTTCCAATGGTTGCAACTTCACATATAAAATCTTGTCCATTTGCTTTGGATTTGTC

>PvRGL268

GCAACTTCCAAATATAAACAATTCATTGCACATTTATATCTTTTTCTTTCTCATTTGTTACTCAAACATGGTTATTTTTATGATATTAACTTGTAGGTCTGGTTCAAACAATAAAAGGACAAAAGGAAGGTTTTGATGATGTCGGCAACTATAAATTAGGATTAATTATAGGACACTACGTTTTACCATCCTTCTGGGCAGGAAGATTTTTGTTTGGGATGACTTTATTTATTGCACTGTGTGATATACAAATGGAAAAGTCGACATTTGTCTATGTACGAAAGTATTGAAAATTACTTGCAACATGCCAATTTGATGCCTATTAGATATTCATATAAGGAAATCAAGAAAATGACTGAAGGTTTCAATGACAAGTTGGGTGAAGGAGGATATGGATCTGTGTTTAAGGGAAAGTTGTCTAGTGGACCTGTTGCGGCAATAAAAATGTTAGGTAAATCAACACGAGATGGACAAGATTTTATCAGTGAAGTTGCAACTATTGGAAGAATACATCATCAAAATATAGTCAAATTAGTTGGATTTTGTGCCGAAGGATCAAAACGTGCACTTGTGTACGAATTCATGTCAAATGGATCTCTTGATAAAATTATTTTTTCTAAAGATGGAAATATAGATTTAAGCTATGATGAAATATATAATATTTCAATTGGAGTAGCTCGGGGGATTGCTTATCTTCACCATGGGTGTGAGATGCAAATTTTGCATTTTGATATCAAGCCCCACAACATCCTACTAGATGGAAAACTCACCCCAAAGGTATCTGATTTTGGATTGGCAAAGTTATATCCAATAGATAACAACATTGCCACAATGACAGGAGCAAGAGGAACAATTAGGTATATGGCTCCAGAATTATTTTATAAAAATATTGGAAGAATATCTCACAAGGCTGATGTTTATAGCTTTGGAATGCTTCTGATGGAGATGGCAAACAAAGGAAAAAACCTAAATCCCCATGCAGAGCATTCAAGTCAACTATACTTTCCCTTTTGGATTCATAAGCATGTAGAAAAAGAAAAACATATAGAAATTGAAGATGCCAACGAGAAAGAAAAGGAAGAAGTAAAAAAAATGATTATAGTTGCACTTTGGTGTATACAGTTGAATCCAAATGATCGTCCCACAATGAATGAAGTAGTTGAAATGCTTGAAGGAGACATTGAAAACCTGAAAATACCTCCAAAGCCTACTTTATATCCAGATGAAACAATGTCAAGTGACCTAGGCTTCACTAGTTCTTCCAATTCTTCCATGTAAATTGCAATTAATTCTTTAATAGATTATATAAAAATGAATAATAGTT

>PvRGL269

AGGTCNACGGTATCNATAAGCTTGATATCGAATTCCGTTGCTGTCNGAAGCACATGATGGAGAAGGAAGAGGACAGCTTACCAACAATATTCGATTTTTCAACCATTGATGTTGCCACAAATCATTTTTCTAACAGAACCAAGTTAGGAGAAGGTGGCTTTGGAACAGTACACAAGGGCACATTGGTAGATGGACAAGAGATTGCAGTTAAGAGACTTTCAAAAACTTCTAGACAAGGAACTGAGGAGTTCAAAAATGAAGTGAAGTTGTTGGCAACACTTCAGCACCGTAATCTTGTAAGAACTTCTTGGTTGTTCTATTCAACAAGATGAGAAGTTGTTGATCTATGAATTCATGCCCAACAGAAGCTTGGATTACTTTATTTTTGATACAATGCAAAGCAAATTACTAGATTGGACCAAGCGCTTGGAAATCATCGAGGGAATTGCTCGAGGTCTTATGTATCTTCATCAAGATTCTAGACTCAGAATAATACACAGAGATATCTAAACAAGTAACATTCTTCTTGATAACGATATGATTCCAAAGATATCAGATTTTGGATTAGCAAGAATATTTGGAGGAGATCAAGCTGAAGAAAAAACAAACAGAGTGATGGGAACATATGGCTATATGCCTCCAGAATATGCAGTGCATGGTTCTTTCTCAACCAAATCTGATGTTTTCAGTTTTGGTGTAATTGTTATTGAGATAATTAGTGGGAGGAAGAATCGTGGATTTCGTGACCCTCACCATCATCTTCTCAACCTTCTAGGCCATGCGTGGAGATTATGGGATTGAAGAAGGGCCATTGGAGTTAATAGATGACATATTAGAAGATGGTGCAGCCTTATCTGTAGAAATATTACGGTGTATTCAAGTGGGTCTGTTGTGTGTACAACAGAATCCAGAAAATAGGCCTAACATGTCATCTGTTGTATTGATGTTAAATGGCGAGAAGTTACTACCAAAACCAACCCAACCTGGATTCTATATAGGTAAAGATAATAAATCTGNCACTGGATCTTCAAAGCAATATGAAAGTTTTTCAGTAAATGAAGTTTCAATGTCATCCTTAGGGGCTAGATAGAACATCATTTTCCAGTAATGATATAGATGTATAAATGAAATACTGTTCAAATGTGCAGAAACTTGTGTATATGTTGTATGTGTTCTTC

>PvRGL270

TGTGATGGCAGATTTATACACATCCTATTAGAGAGAAGTTGCACACGTTGTTTGTGACAGCATGGCTGGAGAAATCATGAAGAAATTTTGCTCAGGCTTCATGGCTTTGAATTACAATAATCAACATGTTCTTAGAAGCTTTGGTCTCCACATCCTAACAAACCAATTACTTTCTCCAGGCTTCTGATCCATGGTCCGTTTCTTGTTATTTGTTTCAGTCTCCTGGGAAGTTTCCACTCTTGCCACACTCCTCAAGGGCAATGGTGGACTAGAACGGGATGAAGAATATTCTACCATCCCATTCACCATTTCCAATACCTCACTCATCTTTGGGCGATTCTTTGGATTTTTTACCAAGCATCGGTTTGCTATCATAGAAAGTCTATGGGCTGATTTGAATATTTGTTTCTTATCAAGTCTTGGGTCTAATATTAGTTGAAATTTCTTCCCATCTGATAAGTATGGTCTTATCCATTCCAAGAGCTTCTGCTCACCCCTGGGGCGATTTCGATCTAAAGGGCGCCTACCGGTGATAAGTTCATAAAGGAAGACACCGTAGCTCCACACATCATTCTTTGATGTTAGACGTCCAGTTTGAATATATTCCGGAGATGCATATCCCATTGTTCCCACAACCGCGGTTGAGACATGAGTCAGTCCATCTGATGGTCCCAGCCTTGCCAACCCAAAGTCTGACAGCTTTGCATTCCATTGTTCATCCAAAAGGATATTTGAAGATTTGAAATCTCTGAAAATTATCTGAAAATCCATTTCTTCATGCAGGTATGTTAATCCACGAGCTGCATCTCGAGCTATTTTCAATCTCCTACTCCATGAAAGAGGAGCCTCTGATCGGTGGGATAAATGGTGTTCCACACTTCTGTTTGGCATGTATTCATAAATCAGAAGCCGCTGGATTCCTCTTTCATCATCTTCTGCACAATAACCCACTAGTTTCACAAGATTCGGATGCTCAACAACGCCCAGGACATTCACTTCTGTCACCCATTCCCTGTGCCCCTGCATTCCCCTTTTACTAAGCTGTTTAATTGCAACTTCACTTTTTCCAGAGGGGACCTCCACACTTTTAATCAATCCCAGGTAGACACACCCAAATCCGCCCTCTCCAATCTTAACAGAGCGACTGAAATTCCTTGTGGCTGATTTCAGTTCAGAAACAGTAAAAACTCTGAGGTTGCTGGGCCTCTGGGACAAACTGGGCATTGCATTCCTCCTGAGGGATTCTGTGCTGCCACTGTCTGAAGCTTCTAGAGAATTTAACTCGGAACCAGATCTTCTAATATCAGCACACGTGGAATTCTCTGACGGGCCCGATGTTGACCTAAAGCCTTTGGGCTCATCTTTCTTCTCTCCATAGGAGAATGTAAAACACTTCATGGCTGCTGACTGACTCTGGTTGATGCAAAAGGAAAGTTTTGATCTAACAGAAGTGAGATGTTGGCAGTCCCTTTTGGGTCAAGATTCTGAAGAGGACCAGCACCATTTCATTTGCC

>PvRGL271

AGAAAAGAAAGGGGTAATATTTCATTTACATGACCAGTGATTTTACATATGAATAGGAAAATCAATAAAAAAATATTTTATAATCATCTAAACCATCCTTTCAAAATTATCATATCCTTTCTGAAGTTAACAAAGGAAGGATATCTATACAGTAAATAGATTTATAATAAAAGTAAACTATTATCGTGGATAAAAACTAGTTGTAGGCATTTCATTGACAGAGAATGGCATGGAACTGTTGGCAGAAATGGTGGAACTTGATCCTTGCTGCATAACTTTTTTATCTATTCCGTGTAAGAAAAAGGCAGGTTCTTGAGGTGATGGCAATTCAAGTGAATGATTATTAAGATATGAAACTACTGTTGCCATGGTAGGTCTAATGTTTGGATTTTCTTGTACACATAATAATCCTAATTTGAATGCATCTAATAATCTATCAGTTTCATAATAATTTTCTTTCATATTTGGATCCAATATACTCATTGGTGTTTCATTCTTCCAATGTCTCCACACATAAGTCATGAGGCCTTCTTCCATATTGTGTGATTCATAAGAACGCGCATTCTTTTTACTTGTAATAATCTCAAGAACTATAATCCCAAAACTAAATACATCTGATTTTTCAGAGAATTGTCCAAGCATTGCATATTCTGGAGACATATAACCATATGTTCCAACAATTCGATCAGTTTCAGCACGATTTTGATCCACATCAAAAATTCTAGCCAGACCAAAATCTGATATTTTAGGATTCATATTTTCATCCAACAAAATATTACTAGGTTTAAGATCACGATGTATAACTTTAAGTCGAGAATATTCATGCAAATAAAGAATTCCTGAAGCAGTTCCTCCTATAATTTTATAACGTTCGTGCCAACTTAACTTTTGTTGTTGATTACCAAATAAGAAGTAATCCAGACTACGATTTGGCACATATTCATAAATAAGAATCTTTTCTTCCTCTTCTAAACAAAATCCCATGAATGCCACCAGATTTCTATGTTGTAACTTAGCTATCAACAAAATCTCGTTCTTAAATTNCACTGAACCTTGTTTGGAACCTATTGAAAGTCTTTTAACCGCTATATGTCGCCCATNCACAAGAACACCCTTATAAACCTCT

>PvRGL272

GAGGAATTCTTTATTTACATGAAGATTCTCAACTTAGAATTATACATCGGGATATAAAAGCTAGTAACGTTTTACTAGATCAAAATATGAATCCAAAGATTTCAGATTTTGGTATGGCAAAAATTTTCGAGGCAGATCAAACTCAAGTACAGACAGGAAGAATTGTTGGGACATATGGTTATATGTCTCCCGAATACGCAATGCGTGGACAATTTTCAGAGAAATCAGATGTGTTCAGCTTCGGTATCTTAGTTTTGGAGATTGTGAGTGGCAAGAAGAATGCTGGTTTGTATCAATCGAAACACGCTGATGACCTCTTAACTCTTGCTTGGAAGCATTGGACAGAGGAAACACCTATGGAGTTTCTGGATCCAACTCTGAGAGGTTCTTATTCAAGAAATGAAGTAAATAGATGCATCCATATTGGTCTATTATGTGTTCAGGAGAGTCCATCTGACAGACCATCAATGGCCACCATTGCACTTATGCTGAACAGTTATTCAGTTACCATGTCAATGCCACGACAACCAGCATCTTTCCTGCGTGGAAGAAGTCCTAACAGGCTAAACCCAGGGCTTGACTCTGATCAGTCTACCACCAACCAGTCTACCACTAGTTCAATTCCATGGTCTGTGAATGAAGTATCCATTACGGAATTATACCCTCGGTAAAGGATTGTTTTTAATGTTTTCTTCAATCAATCCAGTGAACCATCAAATCTTTCAAACTATAGTGTTACTTTCAAACGTACCAAACTGATATACAGAGTAAACCTAAATGGCTCANAGGGTTCTGTTTAAAATACTTCATGCTGGAT

>PvRGL273

GAACATATCCATTAACTTTTCCATTTTCCAATTTTGAATATGAAGAATACCCATCCTAAAAGCTCAAAACTTTCATACAATGAATTAAGAAGCTAGCTGCTACCAGAATTTAATACTCTACACTCACACAACCCGCAAAGGTGACAATTCTTTACATAGGCAAATAATGAAAGAACAAAGCATTTGTTTGTATAACAAAGTAATCTTCTGTTCTTATCTCTGTGGAAGGATGCAGAAGCATTCATCCAAAAAGTGGAGAACAAGTAAAGTTAAATCAGAATTTTTGGTTGCATTCATCTATACTTTTTCTGATTAACTTTTGTGAATGGCCATCAGAATTTTTGGTTGCATGGCTCTCAGGTGTTGGTGGTGTCCTTGTGAATGAGTTTGAGGAATTAAGTCCTTCCAATGCTGCTAGAACTTCCACCATAGGGGGTCTAAATTTGGGGTCAGTGTTGAGGCATTGCAGTGCAAGTGCAGCTGCAGCTTGTGCTCCTTTCTTAGAGTATTGACCCCCCAACCTTGTGTCCATTATTCTCAAAACTCTTCTGTTGTCAATCAAGAATGGCTTTGCCCAATCCACCAAGGTTTCCTCTGAAAATCCAGGTCTGTCATCTTCCACTGCACGCCTACCGGTAAGCAACTCTAACAAGACTACACCAAAACTATATACATCACTCCTTGAAGTCAGGTGACCTGTAGCAACATACTCTGGTGCAGCATAACCTTGAGTTCCTATAACTCGAGTTGAAACATGAGTATTATCTCCAGTTGGACCATCCCTTGCCAAACCAAAATCTGAAAGCTTTGCATTGAAGTCTGAATCAAGTAGGATATTGGAAGCCTTTAAATCACGAAAGATCACATTTTGGTCCAGGGAATGTAAGAATGTCAGTCCTCTTGCTACACTAATTGCAATGTTGACCCTTATAACCCAAGCCATTGGTTGAACACCTTTTCTGAATAAATGATTCTCCAAACTTCCTTTTTGCATGAACTCATAAACCAGAAGCCTGTTTTTACCTTCCAAACAATAACCAATAAGTTTCACCAAATTCTCATGCTGAAGCTGCCCAAGATAACTGACTTCTGCAAGCCATTCCTTGTGGCCTTGGAAGCTTTCTGGCTTAAGATTTTTAATGGCCACCACAATTCCAGTGCCTGGTCTTGTGGGACCATATGTGTTCTCATCAATCCATCCTTTGAAGACACGCCCAAATCCTCCCTCTCCAATTAAATTTTCTCGCCTGAAGTTCTTAGTAGCCTCCTTTAGATCATTCAAGCTAAAGGACTTGAGGCTACTAGAGATGGATGCGGTAATATTTGATTCTGAAGCTTTTGGAGCTTGATTTTGTACAGAAGAATTTGAATACTGCCTTGGCTTACTTACAGGCCTCTTACTTCCAGACAAATTGGAGGAAGATACATGAGCAATAGGTTTTCCTTTCGCACAAGTATTTCCCATGATGAAACGTATGTTGTTGTTGTTTAGACCAAAGCAGCAAAACTCATGAAGGAAAAGCTCA

>PvRGL274

AAAAGCTTTTGATGGATACTTCTTTAATTATGTTGAGTTTGGTAGCACTTAAACAATTTAAACCTTTTTTCATCAAAGGCCTAATATGAATAGAATCTATATCACAAGGGCACCAAATTTACAATTATATAGAGGCCAATCTGGCATAAACCAAAACTGACAAGATGTTGACAATTCTGAACAAACAATGAACAAAATTTTATGAATGAATATGATTAGGTACAGAGACAAATTTATAGCTGTCATCATCTTCAATGCCAAACATTGTATGCTTTTTCTTTTCCCTTTTAGGGAAATCTGCAGAATGGTGAGAAGGAAGAGAAGGATGATCAGAATTGGTTTCATCTCTAAAACCTTTGCGGATTTCCCACAGAACCTCTGCACAATTCTTCATAGGTGGCCGTGATTGTTTCAAAGGCGCAACGCATTGAAAAGCTAGCTTGAGAACCTGTTTTACTGCCTTGACGGAGGCTGGATTTCTTCTCAGCCTAGGGTCCATGGCAAATACAGCATCCCATTGTTTCAGCATCTTCATTACCCATCTGATTGTCACTCTTTCATCAAGGGGTCTCTTTGGTTCTATTGGATGTCTTCCTGTGACCATTTCAACAAGCAATACACCAAAGGAATAAACATCACTTTTTTCAGTGAGTTGATATGTTCTAAGATACTCAGGATCCATGTATCCAGCTGTTCCTTTAACTTGAGTTGAAATATGAGTTGCTGTAGGATCATCAGACAACCGTGCAAAACCAAAATCTGCCACTTTGGCCTTTAGGTTCTCAGTGATTAAGATGTTTGATGCTTTGATGTCTCTATGGATAATTGGATTATCTGTGTACATATGAAGGTAAGTTATTGCATGAGCTACATCAATTGCTATGTCTAGACGCTCCCCAATTTCTAATCCTTCTCCCCGAATCCCATCAAGGTGTTCTCGAAGGTTACCATTACCAACATATTCAACAACAATGATCTTCTCATTTCCATGTTCCAAGTATCCATACAATCTCACAAGATTCCTATGCTCAATTTGAGACAAGGTGTATATTTCATTCTTGAACTCATGTAAGTGATTGTGTATTACTTCCTTCTTGGCACGCTTTACGGCAACTATGGATCCATCATTTAGCTTTCCCTTGTAGACAGTTCCAAAGCCACCTTGTCCAATTTCATTATTTGGAGAGAATTTTGCAGTTGACTTGAAAATTTCTTCAAAGGAGAAGTTGCCAATTCCAAGTTGTCCACTTGCAGTGCTAGATGAAGCGTAGGAATAAGAAAACTTCCATTTTGATGAATTCTTGGTGTTACTCTCCGAAGATAAATCAGTTGATGCTCTGACTTTCGATGTATTTTTTCTGTCATCATTTCCAACAGCATAAGGGGCTGATTTTCTTTTGCCATGGAGAAACAATGAAACAATTTGTGCACATTTCTTCACAGCATTCTTGAAAGATTTCAGTGCAG

>PvRGL275

GGAGAAGGCACAAAGGAATTGCAAATGAAAACCTGTATATGCATAATACAGAAAACATGCTTGTATAATTTATTTTTATCTTTGAAAGTTTAGAACACAAAGGAACATAGATAGCTTGAAAAACCTGATCTCCTTCCCAGAGTTGTTCAAGATGGCAGTATGGCATTTCGAGGGTCACTAGATTTTGTGGGCAAAACTTTAGAGGCCAAAATCTTTGAGGGAAATAGTCCCAATATAGAATCTTTAGAGTGTCCGGCAGACCTACAAGAGATGATTCAAGAGACACTTTTTGAAACGTGTAGAGCCTAAGCATCCTAAGATTGTCCATCTTTTCGAAACTTTGTGCATGTACTACAACTTCTTTTACTTTGAAAGTATATAGTGATATACACTCTATTGCATCTGACCCCTTGTTCTTTCTTAAAACCTCACAAATTTCCTCAGCATTAAACAATCGGCTGCGTTTTCCAGGATGCTGAGGACACTCTTTACGAACAATTTCTTGACCCATTTCTTGTATTAGATCGTGCATCACAATTCTACCATCAAAGACAGATATTAGGCCCCTATCTTTGAGAATATCCATTCCAATCTTAGAAGAGAAACCACACTCATCAAGTCTTTCTGCTACCACAATCTCCTCGTGCATCCTCTGATAAAAGCAAGCTATGTCAAGAAATATATTCTTCTCCTCCTCCTCCAGCCCATTCATAACTTAATTTTAATACATTGAAAATACCAAGATGTTGGCCCTTTTCCAACTTTTGCAACTGACTCTCCCATGCTTCTCTTGTTCTGCCATAAAGCAATGACCCCAAAATCTGGAGAGCTAACGGAATCCCTTTTGCATATCTCAACACCTCTACTGACAGTTCCCTGTAAGCAAATTCTTCTGGAGATTTTTGTTTAAAAGCATGTAAACTAAAAAGTCTCAGAGAATCATAAAATATTCAACTCCTTAACTTCGTAGATATCATCAGCTCCAGCATTCCTAAGCACTTGTCTGTCTCTACTGGTCATAATGATTCTACTGCCCTGCCCAAAACTATCACTCCCTCCTATTAATCTTTTGAAGTAGAGCTGAATCGGTAACATCATCAAGAAATGAGGAGAACCTTTGTCCGTTTGAGCCTTTCTATTGTAATATGGTAAATAAGAGAATTTCTCTTCCTTTAGAAGCTCAGACTGATATTTTGTTCTGATACCGTCTATTCCATCTCTTTGTATTTTTTCTTGAACGTCTAAAACAAGGCTGCTGGAATCAAACTGCAATGCCAATATGTAATATATTTGATTACAAATTGTACTTTTTCCTATTCCTCCCATGCCCCAAATTCCTATGATCCGAACATTCGGTGACTCAGGATGCAACAAGGACTGAATTCCTTCAGTATGTTTTTCAATGCCGATGATTCCTTGATCATAACTGGTGGAATAAAGATTCAATTTTCCCAAAATATCTTCCACAATTTTTTCAACAAGTATGTGTTCTGTCCTGGTGACTTTTGAATCCCAGCCGATGAGCAAGTCCAGCAACTTCGGTTAGAGCTTCCTTCCATCCTTGCACTCTGTCCATGTCGTGCTTAAACCGCTGTTCATGCTTATCGAATGCTTCTTTGAATCTTTCTTCCTGCTTCCTAATAGTTGACGGATCCACCTTGTAGAAAACAGGTATCACATCCCTTCCATATCTCTTCTTGCAATCAAGTATCTTAGTGAGTTCTTTCAAGCACCAAGTGGAAGATGCATAGTTTTGGGAGAAAACGACGACATAAATCTTTGATTCTTCTATTGCAGAGAGCAGTGCAGGTGAAATTTCTTCGCCCCTGGGTAATTTGTAATCAATATATGCTTCAAAGTTCTTCCTTTGCAGTGCTGCATGAAGGTGGTCAATGAAGTTGTCACGTGTGTCTTCCCCTCTGAAGCTGAGAAACACATCATGCTTTGTTAGAGAAGCAGACATGGCCAATGATGAAGTGTTTCAATGTTCTCACTCATCACAGCCGCCAAATAACAACCCTACCAACGCCACCACCGTCATGGCTTCACTCTCCGTTTCGCGCCACAGCCACCTCTTATCGGGCTTCCATCCGGCCCGGCCCAAGCCCACCTCGGGGCCCACCAGGGTCCGAATGTCCCTCCGCGATGACGGTCCCGCCAATCGCCGTCGTCGGCGTCACCGGCGCCGTTGGCCAGGAGTTCCTCTCCGTCCTCTCCGACCGCGACTTGCCCTACCGTTCCATCAAAATTCTGGCCTCGCAGCGCTCCGCCGGCCGCCGCATCGTCTTCGAGGACAGGGACTACGTCGTGAAGGAGCTCACAGC

>PvRGL276

AGCCTGAAACTTAATTTGTTCTTTAGAATGCTTTTTATTTGTATCCAATACATGGTTTCTTGAAATATGCAGCACATATCTTAGATTACATTTGAGCATCTCTAGTAACTGTGACCACCTCTAACTCATATTCCATTTGACTCTGCACAAATGATATGAATCATATTCATACGTTCTTCATGAGTAGCATGCTTGAGTCCTCAAAAAATTTAAAGGAACATTTAGAAGTTCCTTATGAAACTACAATTTTAAGAGGTGATGGAGAAGCATGCACTGTTGAAACTCCCTTCAATGCATCAGGATCTCTTGTTGGGGCCTCAACAAGTCGAATTTCCCTGAGCAGTGCAACAGTGTCCTTCATTGTTGGACGGTCCTCAGCTCGGGTGCTGACACATAGAAATGACACAGCTAGAGTTTGTAGCATTTCATGCACAGTGGAATCAGTTCTCCCTCTCAGTTTTGGGTCAAGAATGTCACATGGGTCTCCTTTGCTTGCTAAGTGGTTCCTAACCCACTGAACCAAGTGTGCACCACCCCCAGGAAGTGTTGGGTCAAGTGGGTGCCTTCCTGTCAGAACCTCAAGTAGGACCACACCAAAACTGTACACATCACTTTTCTCAGTGATCTGCTGCATAGATGCGTGTTCTGGAGCCATGTACCCATAGGAGCCAGCAAGGTAGGGTCTTTGAAGTGGCTTGGAATTAGCACAATCAACATTTTCACTTGCAATTCTAGCTAGGCCAAAGTCAGCAAGGTAAGGCTGATAGCCAGGACCTAACAACACGTTCATGGCTTTAACATCTCCATGCAATATGGAAAGCTCGCAATCATGGTGCAAATATGCAAGTGCATGAGCCACTCCTACCATAACATCATATCTGGTCTCCCATTCTGCTTTTCCTTTTCCTAAACCATGAAGTAATGAGCTCAGACTTCCATTAGGGAGGTACTCATAGAACAGCAGCTTCATGTTCTTGCTGGACCCCCAACCCAGAAGCTT

>PvRGL277

CCCATATCCAAACTCAGAAGAGCGAGTAGTTGCAGGACTGGACTTAAATGGAGCAAGAGTGACGGGTCAAAACGGGTGCCCTTTGCCCGTCTCATCTCTCTTGGGCGTGAACCAAATACCCGAGTTAACATTCTTCCACGTCAACTCTAACAGCTTCGCCGGCTCGGTCCCCACGGATATCACCAAATTCCCCTTCTTCTTCGAGCTCGATCTCAGTAACAACAAAATCAAAGGCCAGTTTCCCATCGACGCGATTACGTCAAAAGTGCTAGTCTTCCTTGACCTCCGCTTCAATCAACTCACCGGCCCAATTCCCCCCCAACTCTTCCTGAAGGATCTCGACGTCATCTTCATCAACAACAACCAATTCACACAGTGTCTCCCCCAGAATTTCGGCTACACACCGGCCCGCTACCTCACTTTCGCCAACAACAACCTCACCGGCCCCATTCCAAAGAGCGTCGGTTACGCACCCAACCTTACCGAAGTTCTCTTCCTCGGGAACAAGTTTGACGGCTGCTTGCCCGTTGAAATTGGAAACCTAAAAAGAGCTGTCGTGTTTGATGTGAGTAAAAATTTACTCACCGGCCCAATTCCCCACTCCTTCGGGTGAATTCGAACATTTAAAACATCTTATTCTTGGAGTAAACAATTTTTCGGATCTATTCCCACAAATTTGGGAAATCTATCTTCCTTAATTACCTTGGATATTGCCTCAAATCCATTGACAGGAGTTGTGTCTCAAAGAAATTTTGCTAAATTGTCAAAATTAAAGGAACTGGATATATACTCATATTCATCGTTAATCTTTGATTTTGACTCCCATTGGATTCCACCCTTTCAACTTGAAGAATTAGTTATCGGGTTTTCAAATCCCAACCTTCCTGCGTGGTTATATACTCAACGATCTATTGAAAGGTTAACTATTTGGGAATCATCATTTGAGGCTCCAAACAAATTCTGGAATTTTGTATCAAGGATGACTAAAGTCCAATTACAAGGTAATTTGATAGACGGGAACATATCAAACGTGTTGTTGAACTCTACAATCATTGATCTCTCATCGAATGGTTTGAAAGGTTCCTTGCCTCAATTATCACCAAACGTTGTCTTTGTCAACTTATCAAATAATTCATTATCAGGAGACGTGTCTCCCCTATTATGTGATAATAAGATGTTGAATGGGAAAAACAGTCTGTTGTTCTTGGACATATCATTGAATAATCTATCTGGAGGGCTTACGAATTGTTGGAAGAATTGGAAATCTTTGGTTGCTATTCACTTGGGAAGCAATAATCTATCANGTAAGATACCCTCATCAATGGGCTTTCTATCTAACCTCACCTCACTCCATTTGCATGAGAATAAGCTAAATGGAGAGATTCCTCCCTCATTGCAAAATTGTCGCTCTCTACTGGTCTTTA

>PvRGL278

CCGTTGCTGTCGAGGGCTTGCACTTCTTCTTGCTATTATACTGCTAAGAACGTCAAGGAGGGATGACGATAAGCCCATCGATAACTATGATGAGGAACTCAATGGCAGGCCACACAGGTTATCTGAGGCACTTGTTTCCTCAAAGTTGGTGCTTTTCCAGAATTCAGATTGCAAGGATCTTACAGTTGCAGATTTGTTAAAATCCACAAACAATTTCAACCAGGCAAATATTATTGGTTGTGGTGGGTTTGGTCTTGTCTACAAGGCATATCTTCCAAATGGCACGAAAGCAGCTATCAAGAGACTTTCAGGTGACTGTGGTCAGATGGAACGTGAATTCCAAGCTGAAGTAGAGGCCCTCTCAAGAGCTCAACACAAGAACCTTGTTTCTCTGAAAGGATATTGTCGGCATGGCAATGACAGATTGCTAATATACTCGTACTTGGAGAATGGAAGCTTGGATTATTGGCTGCATGAGTGTGTGGATGAAAGCGCAGCTCTAAAATGGGATGCAAGACTCAAGATTGCACAAGGTGCTGCACGTGGATTAGCTTACTTGCATAAGGGTTGTGAACCATTCATAGTGCATCGGGACGTTAAATCTAGCAACATTCTTTTGGATGACAAGTTTGAAGCTCATTTGGCTGATTTTGGTCTCTCACGACTACTTCAACCATATGACACTCATGTCACCACTGACTTGGTAGGAACTTTAGGATATATTCCTCCAGAATATAGTCAGACACTGACAGCAACCTTTAGGGGTGATGTTTATAGTTTTGGTGTTGTTCTTCTTGAGCTTCTTACTGGTAGAAGACCTGTGGAAGTCATCAAGGGGAAAAATTGCAGAAATCTGGTTTTTTGGGTGTTTCAGATGAAATCTGAGAATAAGGAGCAGGATATTTTTGATCCAGCAATTTGGCATAAGGATCGTGAGAAACAGCTACTAGAGATGCTTGCCATTGCTTGTAAATGTCTAGACCAAGATCCTAGGCAGAGACCCGCTATTGAAGTAGTTGTTTCATGGCTTGATTGTGTAAGATTTGATGGTTCTCAACAGTGATCTTAGTTGCATTCGTTTCCTTGGGTTTTGGTCGGTGTATCATTGACTACACAGTTGTAAAGCAAGTTTGTTGTATGAAGGTGGAATGTTATTGATATAGTTACTGCCTTATTTCGTTTTTTAGTCCTTTAGTTCATTAAAACTGATAGGTGTAATATGTATAAATGATAATTTTAGCAATGAGATGGTCTTGC

>PvRGL279

TGGAATAGACAACTGGGAGAAAGACGAGATTTCTAAAAGAAATTTAAGGCAAGCATAATAGAACCGAATCTTTTCAAGAGTAGAAACAACCAACTCAATAGATATTTGGTCAGAGTCTGACTACTATACAGGACAAAAAGGCTATACTGTCGTGTAATAATCTAATATCAAGATCAGTATCAATGATGGACCCGTCTAAGAGCTGAGAGAAAGCCATGGGGTGATTTATCCTTGTGATCATTGACCTCTTTTGCTATGAGCTGAAGAATTTCTATTGTNTCAGAGAAATCTGGTCTGAGAGTCGGATCTTGTTGCCAGCACCGCTGAAGCAGTTCAGAAAGTTTTGGGTGTGTGGTTTTTGGAATTGTAGGCCTTAGGCCCTTCTGCACCACGCCGACTGCTGCTTGTAATGGGGTCAAGCAAGAGTATGGTAGTTCTCCAGTTAAAAGCTCCCAGAGAGCTATTCCAAAACTGAAAACATCTGCCTTCTGATCATATGGTTTGTGTTCAATGACCTCGGGAGCCATCCAACGGTATGTTCCAGTTTCAGCTGTCATCACACCAGACTGAGTTTGCACTCTAGCAACCCCAAAATCAGCAACCTTTACCACTTCATTTTCATCCATCAGAAGATTGGCAGTCTTGAGGTCCCTGTGAATTATATTATTTTGGTGCAAATAGTTCATTCCCTTGGAAACATCAATTGCTACTTTTAGCAAAGATGGAAGCTTAAATACACCTCTTTGTTTGTGCAGAAAGTCATATAAGCTTCCCCTAGACATAAACTCAGTAACAATACAAAGATTTGGGGGCCTGGTACATGCGCCGATGAACTGAACAACATTCTTGTGTCGAATCTTCCTCATGATATAAACTTCTTGTGCAAACTCCTTCAGCATAACTGTACTTATGCGCTCAGGCTTGAGAACTTTGATAGCAACATCTTGACTGCAATATGTACCTCTGTACAAGTCACCAAATGACCCAGAGCCAACTTTGTTTTCATATTTCAGCTGGTTGGTATCAAGTTCCCAGACATCCGCTCCATCAGTTGGTATTTGAATGCAATGAGGGGATGGTTCTATCCTTGTCTGGTAATGTTCATTGGCAGCATAATGTATGCCCTGAT

>PvRGL280

CTCCAAGCAATAAAACAAATAGAACTGGCCTAATTGTTGGAATTGTTGTTGGAGTTGGAGTTGTATGCTTCCTATCAGTTTTTGTGACTTTTTGTATCATTCGGAGAAGAAAACGTCAGCATGAGGATGAAGAGCTTTTAGGAATTGATACAAAGCCATACACTTTCAGTTATTCTGAGTTGAAGAATGCTACTAATGACTTTAATATCGAAAATAAGCTTGGAGAGGGAGGTTTTGGACCTGTTTATAAGGGGACACTTAATGATGGAAGGGTTATTGCTGTGAAACAACTGTCAGTAGCATCCCATCAAGGAAAGAGCCAGTTCATAACTGAGATTGCTACTATATCAGCTGTGCAACATCGTAATCTGGTGAAACTATATGGATGTTGTATTGAGGGAAACAAAAAGCTTCTGGTGTACGAGTATCTGGAGAATAAGAGTCTTGATCAAGGATTATTCAGTAATTCTTTAACCCTCAATTGGTCCACACGCTATGATATCTGCTTGGGTGTTGCCAGAGGTTTAGCTTATTTACATGAAGAGTCTCGTCTCCGTATTGTACACCGTGATGTGAAGGCTAGCAATATTTTGCTTGACTATGAGCTTGTCCCCAAAATATCAGATTTTGGGTTGGCCAAATTGTACGATGATAAAAAGACCCACATAAGCACTCGAGTGGCTGGAACAATTGGATATCTTGCACCGGAGTATGCCATGCGTGGACACCTTACAGAGAAAGCAGATATATTTTCATTTGGTGTTGTTGCTCTAGAGTTAGTCAGTGGGAGACCAAATTCTGATTCAAGCTTGGAAGGAGAGAAGGTGTATCTTCTTGAATGGGCTTGGCAGCTTTATGAAAACAACCTCTTAATGGATCTGGTGGACCCTAGAATATCAGAATTCAATGAGGAAGAAGTAAAACGTGTTGTGGGAATAGCACTTCTGTGCACTCAGACTTCACCATCATTAAGACCATCAATGTCCCGTGTGGTGGCAATGCTTTCAGGAGATATTGAAGTGAACAGTGTCACTACAAAGCCTGGATACCTTACTGACTGGAAATTTGATGATGTGACCAGCTTCATGACTGAGAATGCAATTGAAGGATTGAATACAAGTCACCAGTATTCATCAGGAAGTACCAGCATTGTGGGTGGTACAGACTTTTCACCATTAAGTGTTTCAAAACTGAACCTTAATGATGGTCTTAGTGAGGGTAGGTAAGAATTGGTGTACTCATTACTGTTCAGATCAAGTGTAGAACAATTATCATGTCACTGC

>PvRGL281

CTGAGATGGGTCTAAGAATTCAACAAAGAGGAGAGATTCTGAAGGGAGTGCCACTGGTTCAACCAGGAGATTATTTGTTCTGGTTTAACAAACCTGGTCTTATTCTTTACCTTATTAACTTTGTGCTCTTTCAGAATGCTTTTCAGCTTGCTTTCTTCTCCTGGTCTGTGCTTCAATTTGGGATTAAATCTTGTTTCCACGCACATACAGAGGATGTAGTGATCAGAATCACAATGGGGGTTCTTATTCAAATCCTGTGCAGCTACGTTACTCTTCCTCTCTATGCCCTAGTTACACAGATGGGTTCAACAATGAAGCCAACGATATTCAACGAAAGAGTTGCTGAGGCACTGCGGAACTGGCACCAGACCGCGAAGAAGCAGATAAGACAGAACCGTGTGGGACCCTTGTCGTTGTCGGTGACACCGATGTCGAGCAGACCCACAACCCCGAGCCACAACATGTCCCCAGTGCACCTCTTGCGCTACTACCGTTCTGAAATTGACAGCTTCCCCACCTCTCCACGACGTTCGAACGTCGACGGCGACCACACCCAGCCCTGGGACGTTGAATCCCCTTCTCCTTCCTACTCGCACCACGAAATGGAAATGGGTCACACCAATGACCCCACCACTAACACAACCCACCATGAGATTGTTCCTGCTGCAAACACCAGGGAATTCTCCTTTGATAAAAGACCTACCACAGCTCCGTAGTCACCCACCTACCATATTTATTTGAGAAATGTGCATGGATTAGATGCATTGCTTTTAAATGCCAGTTATAATAAGATATCTGGTCATATACCTTCCAATATCAATCGAATATGTAGATCATTGAAATTTTTGGATGTGTCTGGAAATCAACTTGCAGGAACAATTCCTGTTGATTTAGGGAATATGGTCTCCCTTGCATCGCTGAACCTCAGTAGGAATCAGTTAGCAAGGTCAAATTCCCACCAGCCTTGGCCAGATAAAGAATCTAAAGTTTCTTTCTTTGGCTGGTAATAAGTTAAATGGCTCAATTCCGACCAGCCTGGGGCAGTTGTACTCTTTGGAGAGTCTTGGACCTTTCTTCAAACTATCTTACTGGTGAGATTCCGAAGGCTATTGAGAACATGAGAAACCTGACTGATGTTTTGCTCAATAACAACAATCTTTCTGGTCACATTCCTGATGGTTTGGCATATGTCACTACACTCTCAGCATTCAATGTGTCTTTCAACAACTTATCTGGATCTTTGCCTTCAAATAGTGGCTTGTTTAAATGCAGCAGTGCTGTTGGGAATCCGTACCTAAGCCCCTGCCGCGGAGTTTCTCTGATCTGTGCCATCAGGGAAGTCAACTAGGGCCTATTGATGGCAACCCTTATAATTCAAACAGAACAACTACTGGCAAGAAATGGGAATGGCTTGAGTTCTATTGAAATAACATCTATAACTCTGCTCAGCCATTTTTTGGTTCTTATACCCTGATTGTTCTATTCTTTTACACACGGAAGTGGAAGCCAAGGTCCAGGGTTGTTGGCTCTACNAGAAAAGAAGTAACAGTGTTTACTGATATT

>PvRGL282

GGCCGCACGCATTTTTGATACTTGCCTTGGAAAGGGAGGCTATGGCACCGTGTACTATGGAAAACTTAAGGATGGACGAGAGGTTGCAATAAAATGTTTCCATGACGAGAGTGAGACAGAAAAGATCATTAAGCAATTCATGAAAGAAACTGAGATCTTGGTTTTGTTGCACCATACAAATCTGGTTCTACTTTATGGCCGCACTTCTCGCCATAGCAACAAACACATGCTAGTGTACGAGTACATCTCCAATGGCACTCTTTCTAAACATCTTCATGACTCTTCCTGTGGTAAACTACCATGGCATACTAGATTGAACATTGCCATCGAAACTGCAACTGCCTTGGTATATCTCCATGACTCAGGTATCATCCACCGAGATGTGAAAGGGAGTAACATTCTGTTGGATGAAAATTTTACTGTTAAAGTTGCAGATTTTGGATTCTCACGGTCTCTTCCAGATTATGTCACTCACGTTTCCACTATTCCAGTAGGAACTCGTGCTTACATAGATCCTGACTACTATGAATCGGGAAGAGTGAGTGACAAAAGTGATGTCTATAGTTTTGGGGTGGTCTTGTTTGAACTTATATCTTCCAACCCCCCAAGTTTAATGGAAGGCACAGATTACGTTACTCTTGCACAATTTGCAAAGAGGAAAATCTTGAACAAGGAATTAAACGCGGTAGTGGATCCAAGTTTTTGGTTTGGTGTTGACAGAAACATGATGGAAATGATAACAGCAGTGGCAGAGTTAGCATTTCAGTGTGTGCAGTGTCCCAAGGAACTCAGACCATCAATGAAACAGGTGCTAGAGACCCTTGAGGGCATTAGGAAAGGAACATGGGGATTCAACCAGATAACATAGTCTTTGTTCCTTCAGCCAACAAGCCTGATTCTTCCAAACCATTCATTTGATTTTGGTTGAAAAGATATGGAGTTTTGCCTTAGTATAGTAATTTGAGGTTAGCCATGCATCTTGCAAATGATGTGCATGTAAAGGAGTATTTAGGGTGTCTATGCTCTAGCTTGATTACTTTTTTTAGGCACATTGAAAGTGTATCCTCGTCTCACTGAATTCATGTTCAATACATTACTGTAGTTTCACC

>PvRGL283

AATTCCTGCAAACTTGAGCCAGCTCCCGCGGCTTAAGATGTTTAGTGTTTCCAATAATCTTTTGACAGGGCAGGTTCCATCCTTTAAAGCAGGTGTGGTTGATGCAGCAAGTTATGCAAACAATTCTGGTTTGTGTGGTGCTCCCTTGGATCCTTGCCAGGCCAAGAGTCAAGCGAGAAACACTGCTGTTATAGCTGGAGCAGCTGTTGGTGGTGTGACTGTTGCAGCATTAGGTTTGGGCATTGGAATGTTCTTCTACGTGCGCCGTATTTCTTATAGGAAAAGGGAAGAGGACCCTGAAGGAAACAAGTGGGCAAGAAGTCTAAAGGGAACCAAAACAATAAAGGTTTCTATGTTTGAGAAGGCAATTTCAAAAATGAACTTGAATGATCTCATGAAGGCAACTAATAACTTCAGTAAAAGCAATATTATTGGGTCTGGAAGATCAGGAACTGTTTACAAAGCCGTCCTTCCTGATGGCACATCACTCATGGTTAAGAGATTGCAGGAATCTCAACACTCAGAGAAAGAATTTATGTCTGAGATGGCTATACTAGGGACTGTCAAACATGGTAACCTGGTTCCTCTTTTAGGTTTTTGTCTGGCAAAACGGGAGAGGCTTTTGGTCTATAAAAATATGCCAAATGGTACCCTCCATGATCAACTGCATCCTGATGAAGGCGTGTGCACCATGGATTGGGCTCTAAGGCTCAAAATTGCAATTGGGGCAGCCAAAGGGTTAGCATGGCTCCATCATAGCTGCAATCCCCGTATTATCCATCGAAACATAAGCTCTAAGTGCATCTTGTTGGACACTGATTTTGAGCCCAGAATTTCTGATTTTGGTCTTGCTAGATTGATGAACCCAATTGATACACATTTGAGTACTTTTGTGAATGGGGAGTTTGGGGATTTGGGTTATGTTGCTCCTGAATATGCAAAAACTTTGGTGGCTACTCCTAAAGGGGATATTTATAGCTTCGGGATCGTGCTTCTTGAGTTGGTCACAGGTGAAAGAGCAACTCATGTGGCTAAAGCTCCAGAAACTTTTAAAGGAAATTTGGTTGAATGGATTTCACAGCAATCTAGCAATGCAAAACTCCATGATGTCATTGAGGAATCATTAGTTGGGAATGGAGTAGACCAGGAGCTTTTCCAATTTCTGAAGGTTGCATGCAATTGTGTTTCGGAAATGCCAAAGGAGAGGCCTACCATGTTTGAAGTATACCAGCTTCTAAGAGCCATTGGCATAAATTATAATTTCACAGCTGAGGATGAGATAATGCTGCCTGTAGACTCTGGTGACGCTGATAACTTAGAAGAACTTATTGTAGCTCGAGAGGGACATGATTGAAAAGGTTTGTATGTGAGATCTTATTAGGGTATGCAATCAATTTTAATATGTAAGTATCTCTTTCATTGTGGCCCTAGAATAAATGTTAGTCTTTTATATATAAAATATATAGGTTTTATGGTCT

>PvRGL284

CTAATTAATGTAGTACTATTTCAATTAATTTAGAACTGAGTTTTTTTTAATCAACAAGACAAAAACAAAAGTATTGAAGATAGGGAAAAAAGAGACATCCACATGTTACAAGCAGATGCAAAGAGGGTGTAGATGGACACTAGATTTCACCCATCTTTCTTGCATATCTGATACAATCATCCAATATCATTTTGGCATCATATTTATATACAAATCCTTTGTCACACAGCTTTGTTGAGGTCCACTTGATATCCTTACTCTGCCCATCTTTATATTCTTGTTTGACGGTGAATTCTGGATGATGAAGAGCGTAATGGTTAGTCATCTCTTGTAATGAAATGTAAGAACTTGCACACAAAAATCTTCCACTGATGGAAGTGCTTTCCATGCAGAAAATATGAGCTTCACATACGTCATCAATGAGTACAAGAGGAACTTTACCCAGCAATCCCTCTAGGAACTTGAGTGATTTGTAGGCCCTTTCCTGTTGCACGATCTGTGCGATACAAATTATGCTGCTACTGGGAGAAGATGTAAGGGTTTCACCTCCCACTAGTCCACAAGGGAGTGTTACCACTTCTAATCCTCCACCATTTTCATCATTTCCATAGCTCAACAGATGTTTCTCAGACAGTGTTTTTGAATAAGTATAGTCCTTGTACGCAGGATCTTCAAGGTAAACGTATTCCAAGGAATCATTGAGAGGGGTCCAGCAAGTTTCATCCATTACATCTTTGAAGTCACTCCCATCTTCTTTCAGTGGAGAGGCTGAAACAACAGACGCAGTGTAGATCAGACGTTTCACCGTCCCTGATCTCACACATGACATGAAAATGCTTTTTGCCCCTGCAACTGCTGCTTCTGAAGTATCCTTGTACTGAGATGAACCTGGTTCATGGGCGAGGGGAGTAGCAACATGAAACACAAACTCGCATCCTTGAATTGCAGGATCAAATTGAGTTGGGTTGTAAACATCAGCTTCAAACAGAACCAAATTGCCTTCTGCATGAGGAAGACTCTTTAAGAGCCCTACCTTGGACTCGTTCTTCAAGTCTCTGAGAGTTGCATGAACTGTGTAACCCCTGGCCAGTAGCTTGTAGATGAGCCAGGAACCTATGTAGCCATTGCCTCCTGTGACACACACCTTGCTCCCTTCGCTCATATTTTTTCCCTCTCAGAGAGACGACAACAAGCAGGTAGAATACAACTTGCAAC

>PvRGL285

GACTGCTTCAGCTCTACAACAAAGATAGAACTGCTGTATAAAATAAATGAAAAAATTATACTACAATTTTTGAGCAATCTCCAAATATCAGACAAAAAGATATTCCAACATTTGTAAGAAAATATCAATGAACTAGTGCTATATAAGGAGCCTTCCATGAGTAAATCTTTCTAATCATGGTTTTGGTGCCATATTTTTTTAAAGGAAAACTTGACAGAAAAAATAAGTATGAAGTCTTAGGGTACTGCTATAATACCTCTTGCTTATATTTGCACCATACACCTATCTACCTTCATTGTTCATTAGCTGGGAAAACACTTGGCTAGTACTAACATCTGAAGAAAAATTATCATAATCCCTCTCTGCTCTCGTGTTGGTAGAAGGATTTCCAGGAATAACTGTACTACTGGTCACACTCGCAGCTTCACTAGCACTGCTGTTGGCATGTGGTTCCCTTTGTTGTCCACTTTCTTGGAGCTGAAGTGCATACTCCAAATTCCATAAGACATCACCCATAGCTGGTCTGTCAACTCCATACTCAGACAAACATTTCTCTGCAGTTTCACAAAATTTGTTTAAGGAGTCTTGTTGGATCTGTCCAACAAGATGGGGGTCAACAATTTTGTCCAGCATGCCTTTCTTCAGCCATTCAAGTGCCCATTCTGCTAAATTCACCTGTTCTCTTGCTAATTGTGGATCAACAGCAGGTCTGCCACAAAGAACCTCAAAAAGCACGACACCAAATGAATAAACATCTGACTTATCCGTAAGCTGCTGTCTGCGGTAATACTCGGGATCAAGATAACCAAAACTACCTTTCACATTAGTACTCACATGTGTTTCATTGATGCATGGTCCTGACCCCTTGAAGACAAAGTCAGCAACTTTGGCCACATAGTCTTCATCAAGTAATATGTTGGTTGATTTAATGTCACGGTGGATGATTCCTTGAGCAAAGCCAGTGTGAAGATAGTGAAGACCTCTGGCCGCACCAATACATACTTCAAGACGCTGCTTCCAAGAGAGAGGTGGTTGTCGTGATGAGCCATACAAATGCTTCTTCAGCGGACCCTTTTCAACAAACTCATAGACCAGTATCATTTCTGAATTTTCTTCACAGAAACCAACTAGTGAAACAAGGTGGCGATGACGAATTTTGGACAAAACTGTTATCTCAGTTTGGAACTCGGGAAGGCCTTGTCTGGACCCTGGCATGCCTCTCTTGACAGCAACCTTCATATTGTCTCTGAACACTCCTTTGTAGACCATACCAAACCCACCAGACCCTATAATCAAATTTCTATTAAAGTTGTTTGTTGCTGATTGTATTTCAGCAAAAGGGATCTTCAAGCCAAGAAGGCCATGAGAACCAGGCTCAGAGCTTCTACTAAGGGAACTCCCTCCAAACATGCTTAAAGGTGTCCATCCAACACTTTCCAATGTTCTTTGTTTTGGTTTTTTGTTCCTGCATTTGGTACCAAGCAGAAAAGCAGCTACAACTAAAAATAAGACGACAATTCCTCCAACAACTGAACCCACCAACACCCACAAATTCTTCCTCCTATGCACAACATTAGTACTGACATCATTGACCATCTCCATTATCTCTGCACCATTCAATATGGCATTCCTCCTTATGGAACTGCTTAGCTCAGAAGGACCAACACTC

>PvRGL286

TTTTTTTTTAGATTTATAAACCTTGAAACACTCTTACAAATTCACCACAATTAAACAAAAGAAAACAACATATAAAGTATCACATTTAGCTTATTTATTCTTTCATGAGTCCTCGACTCAACACACTCTTTCTTTCAGCCTATATAACTAAGTTAACCACAAAACATTTTCACAAGAAATTCAGGCTTTCCCATGAGAAATACAAGGCTTCCCATGACCACTCCAAATACCATTCCACAGCCATATCCTATAGCCACTGCTTTCCAACCAAATCCAAATTTCTCTTCTCTCCACAATGTTTGTGAAGAATGTTGATCATGCTCCTCCCCACATTTCACTGACAATGGAACTCCACATAAGCCCAAGTTTCCCTCATAGGAATCATTCAGAAAAGAACCAAACTGTTTTCCTTGAGGTATTTCTCCCCCAAGATTATTATAGGAAAGATTCAGCACTTCAAGAAAGTTCATATTCACCAATTCTGTAGGAATCCTACCAGTCAGCATATTTGAAGACAGATCCAATGATTCCAATTTTATCAAATTTCCCATGGATTGGGGAATAGGACCACTGAGCCTATTGTGGGAGAGGTTCAGTCCAATGAGTGCTTCAAGCTCTCCAATTGCATTTGGAATCTCTCCTTCAAATTTGTTTCCTGATAAATCAATGATCACAAAGTTTGTTGGAATCTTTTTGAATTGCACACTCGTGCCTTTTGTTGTAATAGTCGTAGGTGAATAATATTCTGCAATAGCAGATGGCCATGCCACATAATGTTGTGAATTGCCAACAACCTCGTCTTGAACAATATTCTTCATGGATTCAAAACTCTGCATGTAGTCTTCTGGAATTGGGCCGCTGAAGTTGTTGGATGATATATCAAAAATATTTAAACAGGGAAATCCATGCTTGGTCTTGAAAATGGCAATGGGACCATGCAACTTATTAGCCCGCAAAACCAACACTTTCAAATACGGTAGAGTTCGAAGCCAGTGAGGAAATGTATCTTCTATTAGATTGTTGCCAACATTTAAAATCTTTAGGCGCATGCAGTTGGACAAAGATTCTGGCAAAGGACCTTCTAATCGGT

>PvRGL287

ATCCGTGGAGGGCTCATTGGTGGCATTTGGGTATAGGGATTTGCAAAATGCAACGAAGAATTTCTCAGAGAAACTGGGAGGAGGGGGTTTTGGCTCTGTCTTCAAAGGAACATTGCCTGATTCAAGTGTCATTGCAGTTAAGAAGCTTGAAAGCATTAGCCAAGGTGAGAAGCAGTTCAGAACAGAAGTGAGCACAATTGGGACAGTCCAGCATGTCAATCTTGTAAGGCTTCGCGGGTTCTGCTCTGAAGGTACTAAGAAACTTCTGGTTTATGATTACATGCCAAATGGTTCATTGGATTCCAAAATATTTCAAGAAGATAGATCCAAGGTGTTGGATTGGAAAGTGAGGTACCAAATTGCTCTTGGAACAGCGAGGGGGTTGACTTATCTTCACGAGAAGTGTAGAGACTGCATCATACACTGTGATGTGAAGCCAGAAAACATTCTCCTTGATGCTGATTTTGTTCCAAAGGTGGCAGACTTTGGCCTTGCAAAGCTGGTTGGAAGGGACTTCAGCAGGGTCCTCACTACCATGAGAGGGACAAGAGGTTATCTTGCCCCAGAGTGGATATCAGGTGTGGCTATCACTGCCAAAGCTGATGTCTACAGCTATGGAATGATGCTTTTTGAGTTGGTGTCAGGTAGGAGGAACTCTGAGGCATCGGAAGATGGCCAAGTGAGGTTCTTCCCTACTTTTGCTGCAAACATGGTCCACCAAGAAGGGAATGTGCTTAGCTTGTTGGACCCTAGGTTGGAGGGAAATGCTGACATTGAAGAGGTCAATAGAGTCATCANAATTGCTTCATGGTGTGTCCAGGATGATGAATCTCACAGGCCTTCCATGGGTC

>PvRGL288

TGATAAGAGGCCGTCGATGCCAACGACGTCGTTGGACTTCGGACTTTCCAGAATCGGACCAACGGGCATGGACAAGGCTCATGTCAGCACCGTGGTGAAGGGCAGCATGGGATACCTAGACCCAGAGTATTACAAACGACAGCGTTTGACGGAAAAGTCTGACGTGTACTCTTTCGGTGTGGTGCTGTTCGAGTTACTCTGCGCCCGTCCGCCTCTGATCCGAACGGCGGAGAAAAAACAGGTGTTACTGGCTGAATGGGCAAGACACTGTTACCAAAACGGTACCATTGCACAGATTGTTGACCCGGCATTGAAGGGTAGGATCGCAGCTGAGTGCTTGAAGAAGTTCTGCGAGATTGGGGTGAGTTGCTTGTTGGACGACGGAACGCAGAGGCCGTCGATGAACGACGTGGTTTGGATGCTGGAGTTTGCGTTGCAGCTACAAGAGAGTGCAGAACAGAGGGAAAATGCTAACGTTGTGGGTGGTGAAAGGGTGAATGAAAGAAGAGATGAAGGTATGGAGGATTTGTTTAGCAGTGGAACGAGTGTGGGCCAGGTTTCTGATTTTAACAAGAGTAGTGGAACGAGTGTGGTGAGTGTTAGCAGCAGTAGCGGAGAGTACAAAGAGAGTGATAGAATGATGTCTGGAACTGTTTTCTCTGAAATTGTTGACCCAAAGCCACGTTGAGGAGAATCAACCACTCCTCCTAAAGCTCACACCATTTATGTGTTTTAGTTACCTTCAACTGTCTCTGTACATTGCTTCTGTCTCTTCTCAATGAAGACAGGTCCAAAGTAAGCCAATTTCAAGCATTGCGTCCTTAATTATTGTAACAATTTTCATATTATCAGATTTAATGATAATAATATTGTTCTAAAAGTTCT

>PvRGL289

CGATTTATTAACTTCACTATACCTTGTTTTCACGACAACACAGCATATACAAGAGTGCAAACACCGTATAAATAAATAACTATTCACGTAACCAACTTCTACTGCACCAACAATGGGTGGTGTTAGGTTTATAACGGGGGTTGTGGTGTTGTTCATGGCACTCAGCTCCGGCGTGCGGTCGTGTCCTCCGTCGGACAGGGCCGCGCTGCTGGCATTCAAGGCGGCGCTACGCGAACCCTACCTGGGAATCTTCAACTCGTGGACCGGCGCCGACTGCTGCCACAACTGGTACGGCGTGAGCTGCGACCAGGAGACCCGCCGCGTCGCCGACATCAGCCTCCGCGGGGAGTCGGAGGAGCCGATCTTCGAGCGGGCGCACCGGACTGGCTACATGACCGGCTACATCTCGCCGGCGATCTGCAAGCTCGCGCGGCTTTCCAGCATCACCATCGCGGACTGGAAGGGAATCTCCGGGGAGATCCCTCGCTGCATCGCGGCCCTGCCGTTCCTCCGCATCGTCGACCTCATCGGAAACCGCATCTCCGGCAGCATCCCCGCGGACATCGGCAGGCTCCACCGCCTCACGGTGCTGAACATCGCCGACAACCGCATCTCCGGCACGATCCCGGCGTCGCTCGCGAACCTCACGAGTCTCATGCACTTGGATCTCCGTAACAACCTCCTCTCCGGGCCCATTCCGAGACACTTCGGTTCTCTCCGAATGCTGAGCCGGGCCTTGCTCAGTGGAAACCGCCTCAGCGGGCCCATCCCGGGCTCCGTTTCACTCATTTACCGTCTGGCGGATCTGGATCTGTCCCGGAACCAACTTTCCGGGCCCATTCCGGAGTCACTGGGTAAAATGGCGGTGCTCTCTACGCTGAATTTGGACCTGAACAAACTCTCCGGGCCCATTCCCGTGAGCCTGTTCAGTTCGGGTATCAGTGACCTAAACTTGAGCCGAAACGCGTTGGAGGGTAACATACCCGACGCTTTTGGTCCAAGGTCTTACTTCACTGCCCTTGATCTGTCCTACAATAATCTGAAAGGAACCATACCCAAATCACTATCCTCTGCTTCCTACATTGGACACTTAGATTTGAGTCACAACCATCTCTGTGGCAAGATTCCCCTTGGATCTCCCTTCGATCATCTAGAAGCTTCTTCCTTCGTCTTCAACGATTGTCTCTGCGGAAAGCCTCTCAAACCTTGCTAATCTTCCTCCAACAATCTTGTACAATTAATTAATTAATTAATTAATTAATAATTACCGTTTTTATTACTCATTACAAACTCACTCTCCCAAACTCAATATTTGTAATTTGCAATATAGGAATTTAGATTTGTGTGTACGTGCATTATTACTT

>PvRGL290

CAAAACTAACACCAACCACTGAATTCTCTCTTCTAATTCGAGGGGATGAACACACGGACCCAACCGATGAAGCTGTGCATATGGTTTCCTCCAAATTTTCCCACTCACTGCGTCTGAGAAGTTCAAGCTCCTCAGCCACTTCAATCATGGTAGGCCTCATGTCACTATGAAAAGAAAGGCATCTAAATGCAAGCTCAGACACTTTGTGCACAGAATAGAGTGTCCAAGCATCCCTATGTGGCTCAAGGAAGGGGTCAATAATTTCATCTAGACAAGCCCTCCTAATCCTTTCAACTGCAAGTGCAGCCAAATTGATTTCACTTTTAGGTCGCGAAAAATCAACTACTTTCATGGCAGTTATTATCTCTACCAAAACCACTCCAAAACTGTAGACATCACTCTTATCAGAAAGGTGGAAGTTCTGGTGGTATTGGGGATCAACATAGCCCGGGGTCCCCTGTGGAGCGGTTGAGATATGTGATGTTTCTGACATGCCAAGCCTAGAAAGCCCAAAATCTGCCACTTTTGATTGAAAACTATAGTCCAAGAGTATGTTGCTGGATTTTATGTCTCTGTGATATATTGGAGGGTTAATTGCAGAATGAAGGTATGCTATAGCATTAGCAGTTTCAGTAGCAATGGTGAGTCTTACTGTCCAAGGAAGTGCTCCGCCCCTCTCTCTTTGCAAATGTTGAGATAGTGTTCCGTTGGGCATAAACTCATACACAAGTATCTGCTCTCCCCCTTCTATGCAACAACCTAGGAGGCGCACTAAGTTTGGGTGACTCACTGAAGAAAGGAGCTTGATCTCGTTTATGACTTGGTCAACACTGTTGGTGTCTCTATACTTAATCTTTTTTATAGCAACGCACTCGTCATTGTGAAGGTGTCCAGCATAAACTGTGCCAAAAGCCCCAGTTCCCAGCCTATGTTTTTCATCGAAAAAACTTGTGGCCCTTTCTATTTCTTTGTAGGGATATAGAGGAACAGTAGAGTCGCC

>PvRGL291

CAAGAATTCAAAGCTCTAATTTTTCCCTACATGACAAATGGAAGCTTAGAGCAATGGCTGCATCCTAAGACACTAAGCGAAGAGGATCCGAGAAAATTGAGCCTTGATCAAAGATTAAATATCATGGTTGATGTTGCTTCTGCATTACATTATCTTCATCATGAATGTGAGCAACCAATAATTCATTGTGATTTAAAGCCAAGTAATGTCCTCCTCGACAATGACATGGTTGCTCATGTGAGTGATTTTGGCATATCAAAACTTCTCTCAGCCATCAATGACAACACTTCTAAACAAACAAGCACAATTGGAATAAAGGGTACTCTTGGCTATGTTCCTCCAGAGTATGGAGTGACTTCTGAAGTATCAACATACGGTGACATATACAGCTTTGGGATTCTTATGTTGGAAATGCTTACAGGAAAAAGTCCCACAGATGAAATGTTTGAAGATGGTCAAAATCTTCATAACTTTGTTCTAACTTCATTTTCTGATAATCTTTTGCAAATTTTGGATCCACTACTTATTCCAACAGATGAATCACCACCATTGAAAGAAAACAATTGGAATCTTGATCCAAATGTTGAGATGTGCTCAGTGTCCCTTTTTAGGATTGGACTTGCTTGTTCTATGAAATCACCAAAAGATAGAATGAATATTGCCCATGTCACTAGGGAGCTTAACAGAATCAGAGAGGTCTTTCTTGTTGGTGTGGATGATTCAAAATAATCAAATGCTTCAGACATTTGGGAAACAGGTGTTTGCAGTGCATGTGTTTGGCTGCAAAATTGTCTATTAGTTTTCCTCAGTTTTCTAGCTATCTATATAAATCAAATCCATCTTTTCATGTAACATATACTATGTACTGATGGATTTAGTATACCTTCTGGTTAGTGCAGTTCCATATTTTTATTTCAATGTCTGTGATTTTCT

>PvRGL292

GATATAAAGAACTATATATCTTGGCTCAATCAAATGACTTGCTTGGAAAACTATTTGATGAAGGAGAACTCTGTTCACTCAACCATAACTCTTGGTCTTGAACCGAGACTGGTGAATTACTATTAGGCCTTTGTAATGATTTTATAGGCTTGGTTTGGAACTTAGGGTACCCTTTTCCCTTTTCTCCATCGTTTTTTGTTGTGTTTTTTGTGGTACTTAGTGGTTTCTCACTTTCATTGGAGGAGGAAGAGCAAGAGGATAACTCAGTCGAGGTTGCACAATTGTTGTCTCCCAATGTTGTCCATTTACTCAACCTAAGTTGCTCCAACTCACAAGCCACTTCCGTCATCGAAGGCCTCATGTCCCTATGGAATGCTAGGCATCTAAATGCCAACTCAGCCACCTTGTGTATGGAGGAAAGGGTCCAAGCGTCACTTCTTGCTTTGGGTTCAAGGAATGGATCTATAATCTCATTCAAAAGCCCTTTTCCAATCCTATCTGCGGCAAGAGAAGCCAAGTTCACTTCATTATGCGGACGTGAAAAGTCTACCACTTTTAGCCCCGTTATGATCTCAACAAGAACAACACCAAAACTATAAACATCGCTCTTGTCAGAGAGGTGAAAATCTTGATGGTATTGTGGATCCACGTAGCCAGGAGTTCCCTGTGGGGCAGTTGAAATGTGCGATATTTCGGTCATTGCAAGTCTAGAAAGACCAAAGTCTGCCACTTTTGACCTGAAATTGTGGTCCAAAGTATGTTGCTGGATTTGATGTCTCTGTGGTAAATGGGGGGACTAATGGCTGAATGAAGATGCGCGATGGCTTGTGCTGTTTCAGTGGCAATCGTGAGACGAGCTGACCAAGGAAGACCTTGCCCTCTCTCCTTCTGCAAATGCTGACTCAGTGTTCCATTGGGCATGAACTCGTACACCAGGATTTGTTCTCCGTATTCAATGGAGCAACCCAACAAGCGAACTAAGTTGGTGTGGCTCACAGAGGAAAGGAGCTTGATCTCGTTCATTACCTGCTCAATACTGTCAGTGTCCCTGTGTTTTAGTCTTTTGATGGCAACCCATTCATCGGTGTACAGTTTCCCAGCATAGACCGTGCCGTACGCCCCAGTTCCCAACCGTTGTTTCTCTGAGAAACTATTCGTGGCTCTCTCAATGTCTTTGTAGGGATAGATAGGAACACCATTGTTGCCGGTGGCTTCGGTTAATCGTCTTTTCGTGCTTTTTGTGACTCTCAATTTGGAGCGACGGCGGCAGAAACAAAATAGTGAACCCGCAGTAACAATCAGTAAAACGCCAATAACAAACCCTCCGATTAAGACAATGAATCTCGTTGTTCCTCCACATCGGCCAGATAAGTACTTGGCTGGGTTGCATAACGACGCTTTCCGGCAACCTGCTCCGGCCAAAAACCCGTCGCCGACAAAGCCTTCGCGACAGTTACAGCGAAAACCAGGCTTTCCGTCAACCGGGGATTTAAGCGTGGTACAATTGGCGTCATCAGAACAGAGACATCGATCTCCCTG

>PvRGL293

AAAAGAAACTCATGGTTGAATCAATCTAAGCAAACAACAAGTCAATCCAAGGGATACAAGATAAATTATATTACATTTCTAGAATTTGAAAACCTCCTCCAACTATCTTGCATAAAGTAATAACTACTTAAGATGATAAATTCCATGTAGGAAAACCATGCTCGCCTCCAATAAGGATTAATGTAGAGAGTGGCAACAATTACCAGCAATACCGTTGTGTACGCCACAGCAAAACTTACACCAAAAGCATGCATGTCAACCGGACTGTCATTGTCTTCTTCATCATTTGGAGAAATGGTAGGTGATTGTGCAGAAGGATGACAACTTTTTAGCAATGGAGGTCCACAAAGAAGAGGATTTCCTTCGTAGCTGCTCTCATCAAAGGTAATAAATTGGCCTTTTCGTTCGGGTGTATCACCTGATAAGTTGTTATGTGCAACACTAAATACAGCAAGAGAGCTCAACTGGTTTAGTTGATGAGGAATTTGACCACTCAACATATTGAAAGAAAGATCTAAACTCTCCATTTGTACTAAATTGGAGAATGTCGTTGGAATTTGTCCAATCAAATCATTGTGAGACAAGTTCAATGCTCGAATTCTTGTTAAGTTTCCAAGCTCAGATGGAATACTTCCGTTTAGTTTATTTTTGGATAAATCAATTCCGGACATATAAGCAAGGATGCTCCCTGTATATGTGTAGGATGTTTTCTTCGTAGTGAAGTTTGCTTTCTCGTTTACATGTGGTATCAAAATTCCTGCATTGCTATTTTTTCCAATAAGAAAGCCATTAAATGCACCCCTTAATAAGTCAGGGTCTTCATTCTCAAAAGGCATTTTACCCAAGCAATTCGGTATTGGACCAAAAAAATTGTTATAAGAAAGGTCTAGTATACTTAAGTCTACCAATTGGCATAACTGCTCTGGTAAATGCCCTGTAAAGCGGTTACCTTTCAAAATGAGTATGTTCAACCTTGTATCAGCTAGGTCTTGCATCATATCTTGAATGCTACTGGTTATTTCATTGTAGCTAAGTTCTAGCATCATTAAGAAAGATCTTTTGCTGAACATTCTTTTGGATAAACCACTTAACCTGTTGTTGCTCAAATGGATGTAATTCACTGAAGAATTGACAAAA

>PvRGL294

CCGTTGCTGTCGCCTTCTCCAATGGCTTCCAACAAAATCCAACGCAGCTCTTCCTCACACGCTAAGAATTTTGACGTGTTTGTTAGCTTTAGAGGTGCAGATACTCGTAACGGTTTCACAAATCATCTTTTTGCAGCCCTTCAAAGAAAAGGAGTTGTCGCCTTTAGAGATGACCGAACAATCGAGAAAGGGGATTTCCTGGAGTCTGAGCTCTTGTACGCTATTGAAGGGTCAGAAGTTTTCATAATTGTCTTCTCAAAGGACTATGCATCATCCACATGGTGCATGAAAGAGCTTACAAAGATCGTTGATTGGGTGAAAGAAAAAGGTCGAAAATTGCTCCCTATTTTCTATGATGTCACTCCTTCTGAGGTCCGAAAGCAGAGTGGAGAGTTTGCAAAAGCTTTTGATGAACACGAAGAAAGGTTCAAAGATGACTTGGAAATGGTAAAAAAATGGAGGGAAGCTCTAAAAAGGAGTAGCGGTCATTGTGGGTGGGATGTCCAAAATAAGCAACAATATGAAGAAATTGAAAAGGTTGTTGAAAGGGTAATCTACATATTGGGTCGTAATCAAATTTGGAGTTTTGGAGATGATTTAGTTGATATGCATTCTCGTGTTAAACAATTGGAAGAACTTTTAGATTTGAGTGCAAATCATATTGTTCGCCTTGTTGGAATTTATGGAATGAGTGGAATAGGAAAGACGACACTTGCAAATACATTGTTTAAAGAAATCTCTCCTCAATACGATGCTTGGTGTTACATTGATGATTTAAGTAAAATTTATTTGGATCTTGGAGTAAACAAGTGCACAAAAACAACTTCTTTGTCAAGTTCTAAATCAAGGAAATATGGAGATACACCATGTTTCCCATGGAACTATGTTGATGAAAAATAGGCTACGTCACTTAAAGACGCTTATTGTTCTTGATAATGTTGATCAGGTTGAACAGTTAGAGAAATTGGGTTTGCATCCTGAATATCTAGGTCCAGGTAGTAGGATCATTATAATTTCTACCGATTGTCGTATCTTACAAAATTATGGAGTAAATGAAGTTTAC

>PvRGL295

GAAGCTTCAATTATGACTCTTCAATCATAACTTCACATTTCATTAAATCCATTCTATACAATAACTCTGAGAAACTAACTACCCCACTCTTCATTTTCAAAAACAAAAACCATTGCCTCAAGTTGCTAAAGCTAATTTTTTTCACTATCTTGGCATTCTAGGGAATCCATTTTGCTAATCATTCACTATCAACATGCAATCTAGTTTCCTTTGAACTCTTAAGAAGGGGAATTGCATCATAGTAGTGGCCAAAGTTCTTCTCACCATAAAATAATGGTTCTCCTAAGCTTTGCAATATTTGTAGAGCATCCTTCATGGAAGGCCTACTAGTAGGTAGTGTTGCAGTGCACATGACACCAAGTTTGAAAACACTGCACATTTCATCTAAGTAACTGCCTTCAATCACATCTTTGTCTAGCAATTCTTCAACATTGCCTCCAGTCAGAATGTGACGCCACGCCCACTCTGAAAGAGATGAGTGTTGGTCTCCATAACTTGCTTCTTTACCGGTTGTCAGTTCTAATAGGATCACCCCAAAGCTGAACACATCAACCTTTTCACTAACTCTGGTTGATTGCACATATTCTGGAGCAATATAGCCAAATGAGCCAATAACAGCTGACATGGTGCTAAGTTCCTCTGGCTTGATTAACAACTTGGCTAGTCCAAAATCAGCAACTTTTGCATTGAATTGAGAATCCAGAAGGATGTTGCTTGTTTTTACATCTCTATGAACAACAGGTGGTAAACAATCATGATGCATGTAGCTCAAACCCTGAGCAATCCCAATTGCTATTTTCAACCTCTTTGGCCAATCAAGGACCACTTTATTGACTGAACCTGGCTTAACATTCTTATGCAGCCACTTATCTAGGCTGAGATTTTCATGATACTCATATACAAGGAGCATAGAATCCTCATTAGAGATACAACACATCAACTTCACTATGTTGGTATGACGAATGTTGCTCAATATTCTAACTTCTGCACGGAAGGAGCTCTCAAGCTTCTTGTTCAACTTTCTGTTATTCCAAATCTTTTTCACAGCAACATATTCTGAACCAACATCAATGCGGTATACTGTACCATATCCACCACTACCAATAATGTTTTGTTCTGTCATTGATGATACAATGGTTGATTCTGTGAAATTCACCCTCTGAAAGGAAGTGAGCTTCCAAGAGTTATCTAATCCGTGCTTTCTCTTCCTTTGAAATCTAATAAACAAGAATGATGCCAGAAGGATCAGTAACAAGGCTACCACCGCCAAGCATATAATCGAACCAATAGACCAAGAAGAGCCCTTGCTTGTTCTTTGAAGGCCAGAATTGCACAAGGTGAGGTTCCGACAGCAACGGCGACAGCAACGG

>PvRGL296

ACCCCATAATGCATATTCCGGTGCCATGTACCCTATTGTCCCAGCAATTCGGGTGGTGATATGGGACTTCTCTTCTTCATCAAGCCTAGCCAACCCAAAGTCAGATATTTTAGGGTTTAAATTCCCATCCAGTAACACATTAGAAGCTTTAATGTCTCGATGAACAATCTTGAGTCTTGATTCTTCATGAAGAAATGCAAGACCTTTTGCCATGCCAATACAGATCTTAAGCCTTGTTGGCCAATCAAGTTTAAGTTGATCTTTTGAGCTGAATAAAGCATGAGCCAGGCTATTATTTTCCATGTACTCGTACACCAACATCAATTGATCCCCTTCAATGCAACATCCATGAAGTTTCACAAGATTAGGGTGTTGCAAACAAGATATTAGGCCTATCTCGTTTAAAAATTCACGATTTCCTTGCCGTGACTTTGATGAGAGTTGTTTGACTGCTACCAATGTACCATCAGATAATTGACCCTTGTAGACAGAACCAAAACCACCTTCTCCAATCTTGTTAGCTGGACTGAAATCCTCTGTGGCCTCTCTAATTTGTTCTAATGTAAATGTCCCCATTTGAGAATCTTGTCTTTCAGTACCTTTTATTCTCCTAATTATTCCTTTGAAGCAGTTCTTCCACCAAAAGATTCCAATAACAATGATAAAGACAAAGCACAGAGCTGTAATACCAAACCCAACTCCAACAATTATCGTATGCCTTGCACTTTTATGTTCTGAACAAGGTTTGGAATGAGAAACAATTGAGAGAGCTGATATGAGGGGACCATAAACTCCACCAACAGGAACCCTTGTAGTCCCCTTTCCAGCCCAATAGAATCGAATCTCCAGAAAATTGTCTGTTACACTGACATTATATGAGAACGTAAGTGGTTTTTCAGCACCATGAATTACATCTTCAATATTAAAATCCTTCCAAACTAATTTTTCCTGAACATAAATATCAAATAAGC

>PvRGL297

GGGGTGACCTTTCCGTTTCTTAAATATTCAGGAGGCGTATAAGCAAGATTGGTGCTATAA

CTCTTGCCCATCCCTGCTGTTCTTCATGAAACCGAAACAAGAGAGACGAGGATCACCTTC

CTGGTCGAACAGAACACGGTAAGCATTCAAGTCGTGGTACAGGGGGCGCCCCTCTGTACT

GCAATAATGCAAAGCCTGTGCGATGTACAGAGCCACCCGCAACCGCATGGCCCACTCAAT

AGTCTGAGTTTCCCAGTGGAAAAGATGCTTGGCGAGAGTGTCATTGGGCATGTACTCGGC

AACGAGGAGCCTCTCGTCGCCGTCGCAGCAGTAACCGATTAAATTGGCGAGCCTAGGGTG

GCGCAACTTGCCAACGCCGGAAGCCTCTTCGACGAACTGCTTGGGATCCGGCCACGCAGC

CTTGCTGAACTTCTTGACGGCAATCCAACGGCTCTGGTTGTGGAGGCGACCCTTGTAGAC

GAGGTTGGGCGCCTTCTCGCCACTCCCGGAGACGATATACTCTGAACTGAAATTGTTGGT

GGCCGCCTTGAGCTCCGGCAGCGAGAACTCGGCGAAGGTGAAATCGACCCCCTCTTTGTC

TGGGTCACCGGCAGAGTCTCCTCCAGCAGAAGCCCTGTGATGGGTTCCGTAGCCCTGGTC

TTTCTCCTTCTCCTTTTCCTTCTCCTTTTCCTTTTCCCTTTCCTTTTCCTTTTCAGGGTG

CGTCTCTCTCTCTCCCACTCTCAGCAGCATCGACGATTGGCAGCAACCCATCTTCACGCT

TTCTCTCTCTTTCTCTTCACCAAATATCACCGATCATCAGATCTCGTATCTCTCTGGTTC

TTCTTCTTCTTCTTCTTCTTCTTCTTCCTTTGTTTTGTATTCTATTCTATCCAACGCATC

CCTTC

>PvRGL298

CTTTGGACTGTGTTGCATTATTGCTTCTCTTCTAAACACTTCATCCACTCACCAATTCCT

TCCATGGAATTGGAATCTTCATCGCCCAAACTCCCACGGATGTACGATGTGCTCATCCAC

TTTAATGGAGAAGACATCCAAAGGAAATTTGTTTCTCATCTCGATTCTGCCCTCTCTGCT

GTTGCCTTCACCACTTTCCTCCACCACCCCAATGCAGTGAACCCAACGCACATCCAACAA

CCTATTCTCAATCTCTGTCGAGTAGCAATTGTTGTTTTCACCAAAACCTATTCTCAGTCT

GCTTGGTGTCTTCATCAACTCCAACAAATCCTTAAATGGCACCAAACTTACTGCCGACAT

GTTCTGCCTGTTTATTACGAAATTGAGCCATCTGATGTACGTCTTCAGAAGGGTGATTTT

GGAAAAGCCTTGCAAGCAACTGCACACCAAACGTTTTCAGGACGAGAACTGGAGCATGGC

ATGTCTAGGTGGAGCCACGCACTCACCAAAGCTGCAAATTTCTTTGGATGGAATGAGAGC

AATTACGGGAGTGATGCAGAACTAGTGGACAAAATTGTTAAGAGCGTTCTAATTTACCAG

TCTTGGTCTGCTACTAAATTTCCAGTTGGATTACATCCCGTGTGGAAGATGTGATTCTAA

CTATAAAAAACAAATCCACGCAAGTTGTACCATAGGGATATGTGAAATGGATGGAACTGG

TAAACCACCCGTTGCCAAGCCATCTACCGTCAAATTCATGGTTTAATCACAGAATAAAAG

CTCGTCAAAGATTTGGCACAGGTATCCAAACGAGAAGGGGATGTCATTACAACGACATCT

TCTTTCCAATGTCCTGAAAACAAAGGTCGAGAATAAAAGCGTTGAAATGGGAAGATTTTT

GATTTTGG

>PvRGL299

AAGATGATCAATCTCCGCTCACTGTCCATCACAGCAATTGAAGATTATGAGATCATTGAT

ATTCACAACATCTTCAGGCCTCCTCCCTATCTCCAGCAGTTATACTTGAGTGGACGTTTA

GACACTTTTCCCCACTGGATTAGTTCTCTTAAGAATTTGGTGCGAGTGTTTCTAAAATGG

AGTCGGCTAAGAGAGGATCCTCTGGTACATCTTCAAGATTTGCCAAATCTGAGACATCTC

GAGTTTCTTCAAGTTTATGTAGGTGAGACATTGAATTTCAAGGCAAAAGGGTTCCCAAGT

CTGAAGGTGTTAGGCCTTGATGATTTAGATGAACTGAAATGTATGACTGTGGAGGAGGGA

GCAATGCCTGGTCTTAAAAAGCTCATAATCCAGCGCTGTGGTTCACTGAAGCAGGTACCC

TTAGGCATTGAACATCTAACAAAACTAAAATCAATTGAGTTTTTTGACATGCCTGGAGAA

TTGATCACAAGACTTTCAAGTGGAGGTGAGGATTATTTGAGAGCACAACATGTCCCAGCA

CTTTATTCCTCATATTGGAGGGATGGAGGTTGGGATGTCTACTCATTAGAGACTTTAGGG

GAGCGAGAGACTGATTTCAGTCGCAGTACTGCAATGAGAAGTCTTGAAAATTGTACTCTC

TGGAAGGTTTAACTCTCATTTTTTTCTACTTTAACATGCATATGTGAGTGATGACAAGTT

TTTGTTTCACATCACCTTGCATATGCTAGCGAAAGCACTCTTTTGTTTCTGCACTCTTCA

TCTCACTGCAGATTCTTAAACTTTCAACCATTATTTGTTTCTTGAACATGTATTCTGAGG

TTAATGAATAAAAGTGTTCAACATTTTTGGTTTACGATT

>PvRGL300

CCCACGCGTCCGCAAATTCCATTTCTAGAATATCATGGAAATAGTTATGACTCTTACTTG

CTATTATCGTGGAAAGGTCAGGAGTATGAGTTTTGGAATCCAGAAAATCTTCTTAAGAGC

ATTGACCTCTCAAGTAATGATTTAATAGGTGAAGTACCCAAGGAGGTTGGATATTTACTT

GGATTGGTTTCTTTGAATTTATCAAGAAACAACTTCCATGGAAAGATTCCTTCTAGTATT

GGGAATTTAAGTTCGCTAGAATTCCTGGACTTATCAAGAAATAATTTCTCTGGCAATATT

CCTTTTACTCTTCCAAACATTGATAGCCTTGGTGTGTTAGACCTATCAAACAATAATCTC

AGTGGAAGAATCCCTCGAGGGAGACATTTTGAAGCCTTTGATGCCTCTTGTTTTGAAGGA

AATGTTGATCTTTGTGGAGAGCAACTCAACAAAAGTTGTCCTGGAGACGAAACAATGCAT

AAACCTCAAGAACCAGCAATTGATGGTGAAGAAGAAGACAATTCAATTTTCTGTGGAGCA

TTATACATGAGCTTGGGGTTAGGTTTCTTTGCCGGCTTTTGGGGCTTATTTGGGTCAATG

CTATTTTGGCAGCCATGGAGAATTTCTTATTTGAGATTCTTGAACAGACTGATAGACTAT

ATACTTTTAATGGCTGAACTGAACGTTGTCAAGTGTCATAGGTGGCTCAAAGGATAGTTA

ACAGAGTTTGCAGTTTATGGAAGATTAACTTCAGAATTCAATCTTGGATGAAAGCTACAT

GGATTACTGGAGGAGTAGGATAAGTAGTATTGAGGTTCACAGTGTAATGCAAAATCAGTG

TTTCAATAAGTTTATGTATTTCATTGTCTTCTATTGTTTTATCAATAATGGTGAGATT

>PvRGL301

CCCACGCGTCCGGCTAGTCCTTTTCATACACAAATGTTGTTCCTCTAGTGGGGTTCTGTA

TTGACTCAGAGGAGGGTTTGTTTTTGGTATACAAGTATGTGTCAGGGGGCAGCTTAGAGC

ATCACTTACATGGGAAGAAGAAAAGTGTGAAGGGTAGTTCACCACTTCCATGGGCTGTGA

GATATCAAGTTGCAATTGGGATTGCAGAAGCCGTGGCTTATCTGCATAATGGAACAGAAA

GATGTGTTGTTCATAGAGACATTAAGCCTTCAAACATTCTTCTCTCCTCTAAGAAGATTC

CCAAGTTATGTGATTTTGGATTAGCTACATGGACTTCTGCCCCGTCAGTTCCTTTTCTTT

GCAAAACTGTGAAAGGAACATTTGGTTATTTGGCTCCTGAGTATTTCCAACATGGGAAAG

TATCAGATAAGACTGATGTTTATGCTTTGGGAGTTGTTTTATTGGAACTCTTAACTGGCC

GCAAGCCAATTGAAGAAAAAAGACCCCCTGGAGAGGAAAACTTGGTAGTATGGGCTAAAC

CTTTGCTTAGAAAAGGGAAAGGAGCCATTGAAGAGTTGCTTGATCCTCAAGTCAAGTACA

ATTCGAGTTACATAGATCAAATGGCTCGGATGGTTGATGCAGCAGCTGCCTGTGTTACAA

ATGAAGAATCTAGGAGGCCTAGCATAGGTGAGATTGTTGCAATACTGAAAGGTGAAGTTG

AGCCTGTGTTCTCTAGAAGAAGGAAGTCTGGTTATCTTGGTAATGGATTTGTGATTGATT

ATTACCCTCAGTTACAACAAACAAATAATGAGATGAAAAGTCACTTGGCCTTGG

>PvRGL302

CCCACGCGTCCGCCCACCCCTCCGCACCAAACCTAAAACTGGGTTAAACTCAAAGCATGC

CAGTGCCGAGGCCAAAGATCTCAGCAGTCCAAGTAGTAAAATTACGAAAGATCTCAGTAG

CACAATTAGTAAGGTCTCTGAGGTTTCGGTGCCTCATACCCTTCAGAGTGGAGGTGAGAT

CTTGTATTCATCCAATCTGAAGAGCTTTAGTTTGACAGAACTTACAGCTGCCACAAGAAA

TTTCCGTAAGGACAGTGTGTTAGGAGAGAATGGTTTTGGATCAGTTTTTAAGGGCTGGAT

TGACGATCACTCACTTTCTGCAACCAAACCTGGCACGGGGGTTGTTGTTGCTGTGAAAAG

ACTTAGCCAGGATAGCTTCCAAGGTCACGGGGAGTGGTTGGCAAGGCATGGTATGACTCA

TGAAGCAGGTCTCGAGGAGGCTCATATTTCCAACCTCTTTCTTGGGGCCTGCGTTTGAAA

GTCGCTCTTGGTGCTGCCAAAGGGCTTGCATTTCTTCACAGTGCTGAAACAAAAGTGATA

TATCGAGATTTCAAGACCTCAAATGTCTTGCTGGATTCAATTCCCTTGCTGGTTTGCTTC

AGAATTATAATGCAAAGCTTGCTGATTTGGGGCTCTTAAAGGACGCACCTACACGTGACA

AAAGTCATGTCTCCACCAGAGTAATGGGAACCTACGGATATGCAGCTCCAGAATATCTAG

CCACAGGTCATGTGAGTGGTAAGAGTGATGTCTTCAGTTTTGGAGTTGTGCTGCTAGAAC

TGTTATCTGGCAGGAGGGCTGTGGACAAGAACAGACCATCAGGACAGCACAATTTTGGTG

GAATGGGCAAAGCCATACATGGGCAACAAGAAA

>PvRGL303

CCCACGCGTCCGGGCAACCATAGCTTGATACAAAATGCCTTCAAAGAGGCTTCCATGGAG

ATCCTTAGTACTTGGTTGTTTCAAGGCTAAAAACCAACAAACCTTGGAAGCTCCAAACAT

AGTTTCAAAGAAGTCGTCATCAAGTAGAATCTTTCTCTCTGATTTAAGCCTGAGCGATTA

TTCATCGCTCTCTATCATGAGTGATTTATCCAATTCTCTAGTGGGATCAAATCTTCGTAT

TTTCACCTACCAGGAGCTCAAAGAGATCACACACAGCTTTAGCAAATCCAACATTCTTGG

GGAAGGTGGGTTTGGGAAGGTGTATAAAGGGTTTATTGATGACAGTTTTAAGCGTGGGTT

GAAAGCTCAGGTTGTGGCAGTTAAGGCCTTAAACTTGGATGGAAAACAAGGCCACAGAGA

GTGGTTGGCTGAAGTAATCTTTCTGGGGCAGTTGAAGCATCGCCATCTGGTTAACTTGAT

TGGCTATTGCTGCGAAGATGAGTACAGGCTTCTTGTGTATGAATACATGGAGAGGGGCAA

TCTGGAGGAAAAGCTTTTCAAAGGTTATGTAGCAGCCTTGCCTTGGCTAACAAGAATCAA

AATAGCAATTGGGGCAGCTAAGGGACTTATGTTTCTCCATGAAGAAGAAAAACCAGTCAT

ATATAGGGATTTTAAGGCCTCAAACATCCTACTGGACGCTGATTACAATGCGAAGCTCTC

AGATTTTGGTTTAGCAATAGATGGACCAGAAAAAGATCAAACACACATTACAACTCGTGT

TATGGGCACGAAAGGTTATGCTGCCCCTGAGTACATCATGACAGGCCATTTGACAACCAT

GAGTGATGTATATAGCTTTGGAGTTGTACTCTTAGAACTCCTCACAGGAAAGAAATCTGT

GGACAAAAAACGACCCAGCAGAGAGCAGGATTTGGTGGGATG

>PvRGL304

GAATTCTCTATCTCCATGAAGATTCTCATCTTAATATTATACATTGTGATCTTAAACCTA

GTAACATTTTGTTAGACAACAAAATGAATGCAAAGATATCAGATTTTGGACTAGCAAGGA

TTATTTCCATTGATCAAATGCAAGGAAACACTAGTATAATTTCCGGAACATATGGCTATA

TGTCTCCTGAATATGCAATGCTTGACCAATTTTCTATGAAATCTGATGTTTTTAGTTTTG

GAGTTATTGTTCTTGAAATAGTCAGTGGAAAAAGAAACGTTGATGGTAATGGGAAAGATT

TTGATGATCTCTTAAGCCATGCTTGGAAGAAGTGGAAGGAAAATGAAGTATTTGAATTGT

TGGATCCCTCATTACAACACTCCTTCTCTGAAACAGAAATCATTAAGTGTGTACAAATTG

GTTTATTATGTGTGCAAGGAAACCCAGATCAGAGACCTATCATGGCAAAAATTGCTTTGT

ATTTTAGCTGTGAGCAAGTTGAGATGTTGCCTTCACCTCAAGAACCAGCATTTTTCAATC

GTGGAAAAATAGAAACCAAAGATGTATAACAAGGAATTAGAGTCTGATTCAAGTTTATTC

TCAACAACATTAGTTATTTGTTGCTCAAAATATTATAATTGTATTTGTAGTAATGTTTAG

GTGTGTATCTATATATACTTCG

>PvRGL305

GTGAAACAATTAAAGTTTGGGGGTTCTCAAGCAGATATTGATTCCTGCAGGGAAGTTCGG

GTTTTGAGTTGTGCACAACACAGAAATGTTGTGCCGTTGATAGGATTTTGTACAGAGAGC

AATTTGAGGATATTAGTTTATGAGTACATATGCAACGGATCCTTAGACCTCTACTTATAT

GTCCAAAGAGAGATCAAACAGTCTTGCACTTGATTAACAGGAGATGAGATTATGCCTTTC

GATTGGAACTCACGGTTAAAGATAGCAATTGGAACTGCACGAGGCTTGCGCTATCTTCAT

GAAGATTGTAGAGTTGGTTGTATAGTGCACAGAGACCTTCGACCCAAAAATATACTTTTA

ACCCATGACTTTGAACCTTTGGTGGCGGATTTTGGGCTTGCTAGATGGCACTCAGAATGC

AATATAAATACAGAAGATCGGGTTATAGGAACTTCAGGGTATCTTGCACCGGAGTATCTT

GAGACTGGAAACCTTACATATAAAGTAGATGTATATGCATTTGGAATTGTATTATTAGAG

TTAATAACAGGTAGAAGAATTGGCGAGTTGGAACAGTTCAATGGACTCTCGTATCTTTCT

GAATGGTTTCACCCTCTACGCATGTCAGAGCCCAGTCATATATTACAAAACGTTCGATCT

CTCAAACCATGCTTCAACTCTGATGAGTTATTGGCA

>PvRGL306

GTGAACTAGGATTCTAAAGATTCCTCCTCCAACAATGAACTTCAAACAATACTGCTGTTT

CCTCTTAGATGAAGGGCAAAGCAAAATTCAGATTGCTAAGAAGAAGAACAGTAGGGGTTA

TCCATGGGAAATGTATACCTTGAAGGAGTTGCTTAGAGCAACAAACAACTTTCATCAAGA

TAATAAGATTGGAGAGGGTGGATTTGGAAGTGTTTATTTTGGTCGAACAAGTAAAGGCGT

TCAGATTGCAGTGAAGCGATTGAAGACAATGACTGCTAAGGCAGAAATGGAGTTTGCAGT

GGAAGTGGAAGTATTAGGAAGAGTGAGGCATAAGAATTTGTTGGGATTGAGGGGATTCTA

TGCAGGAGGAGATGAAAGGTTAATTGTGTATGATTACATGCCTAATCATAGCTTGCTCAC

ACATTTGCATGGTCCACTTGCTAAGGAATGTCAGTTAGATTGGCCAAGAAGAATGAGCAT

AGCAATTGGAGCTGCTGAAGGTTTAGTGTATTTGCACCATGAGTCAACTCCTCACATCAT

ACATAGAGATATAAAGGCGAGTAATGTTCTTCTGGACTCTGAATTCCAATCCAAGGTTGC

TGATTTTGGTTTTGCAAAGCTGGTACCAGATGGTGTGACCCATCTTACCACANAGGGTAA

AGGCACCC

>PvRGL307

GGTTCGGAGGAGGAAGTGTAATAATCATTACAACAAGACCTGAAAACATACTGAGGATAT

ATAAAGCTGATTCTGTTATTCATGTAAAGTTAATGAACGCAAACGAGTCCCTTGAGCTTC

TTAGTTGGCACGCATTTAGAGAAGCAAAACCAAAACAAGAATACCATTTCCTTGCAGAAA

GAGTAGTTTCTTATTGTGGAGGAATACCTTTAGCTCTTGAAGTCATTGGAAGTTATTTAT

ATGAAAGGACCAAAGATGAATGGAATTCAGTACTGTTACTATTAGACAGTATTCCCCAGC

ACGATGTTGAATCGAAATTGAGAATAAGTTTCAACGGTTTAAGCAATCAAATGCAAAAAG

ATTTATTTCTTGATGTATGTTGTTCCTTTGTTGGTAAGAGCAGAACCTATGTCACGAAGA

TCCTAAATGGCTGTGGAATAGACGCTGATAGTGGAATAAGAGTTCTCATAAAGCATAGTC

TCATACAAATTAATAAGAACAACAAATTAATAATGCATCCGTTGCTACGAGAAATGGGAA

GAGAAATTATTCGTCATATTTCAACAAATCAACCCTGGAAGAACAGTCAACTGTGGGTGG

TTTGATAATGATGCAGAATATGCACTGTCAGAGAATACGCTCTNTTCCTCAAGGGCACAA

AAGTCATTCAGAG

>PvRGL308

GGCTGTTCAAAGAAATGAGATGGAGGTTAATGGAGACTGTTATGATGGTAGAAACAAGGA

AGTTGACCCAGTGCTTGGTGAAGGCACAGAATGGGAAATTCAATGGGAGGATCTTGATAT

TGGTGAGCGTATTGGTATTGGTTCGTATGGCGAAGTTTATCGTGCCGATTGCAATGGCAC

TGAAGTTGCTGTAAAGAAGTTTCTGGACCAAGATTTCTCTGGTGATGCACTAGCTCAGTT

CAAATCTGAAGTTGAGATCATGCTAAGGCTGCGACATCCAAATGTTGTGCTCTTCATGGG

AGCAATTACTCGGCCCCCGCAATTCTCTATCTTGACAGAATTTCTTCCAAGAGGCAGTTT

GTATAGGCTATTGCATCGTCCCAATCTTCGGCTTGATGAGAAGAAACGGTTGCGCATGGC

TCTAGACGTGGCTAAGGGAATGAATTACTTGCACACGAGCCATCCTCCCATTGTACACCG

AGATTTGAAATCTCCAAATCTTCTTGTTGACAGGCATTGGATTGTTAAGGTTTGTGATTT

TGGTCTGTCAAGAATGAAGCACCATACATTTTTGTCATCAAAGTCTTGTGCTGGAACGCC

TGAGTGGATGGCACCAGAAGTCTTGAGAAATGAGCCAGCCAATGAGAAGTGTGATGTGTA

TAGTTTTGGTGTGATCTTGTGGGAGTTGATCACTG

>PvRGL309

GCAATAACCTCGTCGGTCCAATCCCCCCCGCCATCGCTAAACTCACCCAACTCCACTATC

TCTATATCACCCACACCAATGTCTCCGGCGCAATACCCGATTTCTTGTCACAGATCAAAA

CCCTCGTCACCCTCGACTTCTCCTACAACGCCCTCTCCGGCACCCTACCTCCCTCCATCT

CTTCTCTCCCCAACCTCGTCGGAATCACATTCGACGGCAACCGAATCTCCGGCGCCATCC

CCGACTCCTACGGCTCATTTTCGAAGCTGTTTACGTCGATGACCATCTCCCGCAACCGCC

TCACCGGGAAGATTCCGCCGACGTTTGCGAATCTGAACCTGGCGTTCGTTGACTTGTCTC

GAAACATGCTGGAGGGTGACGCGTCGGTGTTGTTCGGGTCAGATAAGAACACGCAGAAGA

TACATCTGGCGAAGAACTCTCTTGCCTTTGATTTGGGGAAAGTGGGGTTGTCAAAGAACT

TGAACGGGTTGGATCTGAGGAACAACCGTATCTATGGGACGCTACCGCAGGGACTGACGC

AGCTAAAGTTTCTGCACAGTTTAAATGTGAGCTTCAACAATCTGTGCGGTGAGATTCCTC

AAGGTGGGAACTTGCAAAGATTTGACGTTTCTGCTTATGCCAACAACAAGTGCTTGTGTG

GTTCTCCTCTTCCTGCCTGCACTTAATCATTTCCAGATTCGGTAATTATGGATGCATCAT

GTNTGCCTTTCTATGAACANT

>PvRGL310

GGCTTCGAGCATCGGCGGAGATGCTGGGAAAAGGATGCTTGGGGACGGTTTACAGAGCGG

TGCTCGACGATGGGTGCACCGTGCAGTGAAGAGACTCAAAGACGCAAACCCCTGCGAGAG

AAACGAGTTTGAACAGTACATGGACGTTGTAGGGAAGCTGAAACACCCCAACATTGTTAA

ACTCCGAGCTTATTACTATGCCAAGGAAGAAAAGCTTCTTGTGTATGATTACCTCCCCAA

TGGAAGCTTGCATGCTCTTCTTCATGGTAACCGTGGACCTGGAAGGATTCCATTGGATTG

GACAACGAGAATAAGCTTGGTGTTAGGTGCGGCTAGAGGGTTGGCCAGGATTCATGCAGA

GTACAACGCGTCCAAGATACCTCACGGGAACGTCAAATCCTCCAACGTGCTTCTCGATAA

AACCGGAGTCGCTTTGATCTCCGACTTCGGGTTATCGCTGCTGTTGAACCCTGTTCACGC

GATTGCACGGTTGGGAGGGTACAGAGCGCCGGAACAGGTTGAAGTGAAGAAGCTGTCGCA

GGAGGCTGACGTGTACGCTTTCGGGGTGTTGGTGTTGGAGGTGTTGACAGGGAGGTCACC

GTCGACACCGTACCCTGGTTCCGCAGCTCGTCCGCGCGTGGAGGATGAGAGTGAGGTGGA

TCTTCCGAAGTGGGTTGGGTCTGTGGTGAAGGAGGAGTGGACCCGCTGAGTG

>PvRGL311

TAGTTTGGAGGGCAGCCAACTAAGGGGGCCAGAATTTCCACTTTTCAATTTCAGTTGCAT

TTCAGTAGCCACAAACAATTTCTCAGAAGAAAATAAGCTTGGGAAAGGGGGTTTTGGCCC

TGTCTACAAGGGAAAGCTTCCAGACGGAGAACAAATAGCTGTTAAGAGGCTTTCAAGACG

GTCTGGCCAAGGTTTAGAGGAGTTCAAGAATGAAATGATGCTGATAGCCAAACTACAGCA

CCGAAATCTGGTTAGACTAATGGGTTGTTCTATTCAAGGGGAAGAGAAGTTGCTGGTATA

CGAATACATGCCAAACAAAAGCTTGGATTGTTTTTTATTTGATCCATTCAAACAAACACA

ACTAGATTGGAGAAGACGGTTTGAAATCATTGAAGGCATTGCAAGAGGATTACTCTACTT

GCACCGAGATTCAAGGCTTAGAATAATTCATCGAGATTTAAAAGCGAGTAACGTTTTGTT

GGATGAAAGCATGAATCCAAAAATATCAGACTTTGGATTGGCTAGGATATTTGGTGGGAA

CCAGAATGAAGCCAATACAAATAGAGTAGTTGGTACATATGGTTATATGGCCCCTGAATA

TGCTATG

>PvRGL312

GGGCGTTCTGATGTGTTTTTCCAAAACTAAAATTTATTGAAATTTGCAGCTTCCACAGAT

GTATCCTCTGATAGTTCTAGGAGTTCATCAAAAGGGGTATTATCTCAATCCTCTCCATCA

TCTGGCACTTCAAGTAGTCACCTTGGAATTGGGAACTTCACCTTTGAGGAAATTTACAAG

GCAACAGCAAAGTTCTCTCCAGACAATAAGATTGGGGAAGGCGCGTTTGGTACAGTGTAT

AAGGGAAGGCTTTACGATGGATCCCTCGTGGCTGTGAAGCGTGCCAATAAGGATTTACAG

AACAAGAACGTAGCTGAGTTCAAGAATGAAATAAAAACCTTGTCAAATATTGAGCACAGG

AACCTTGTAAGGTGGCGTGGGTATTTGGAGCATGGAGATGAAAAGATTATTGTTCTTGAA

TATGTTAGTAATGGAACTCTTCGAGACCATTTAGATGGTATTCGGGGAAATGGACTAGAA

ATTGGTGAGCGTCTGGACATAGCCATTGATGTAGCTCATGGAATTACTTACCTTCACATG

TACACAGATCATCCAATTATTCATAGGGACATCAAAGCATCAAATATCTTAATCACTGAT

AAACTAAGGGCCAAAGTAGCAGACTNT

>PvRGL313

GCAAAAGAGAAACAAAGTGATCCTAGTGGCACTCCTTAGTTTAGGAGCTCTAGTACTAGT

GCTATGTGGGGTGGCACTTTCAATGTATATTTTTTGTCGTGGTAAAAGAAGAGGAGAAAG

TCATTCTAATTCTGAAGAAGCGCCAAGGAATGTACTCTTTTCCATATGGAGCTACGATGG

GAAAATGATGTTTGAAAATATCATTGAAGCTACAGAGAATTTTGATGACAAATATCTCAT

TGGAGCAGGAAGTCAAGGATATGTTTACAAGGTGGTGTTGCCTTCAGGTTTGGTTGTTGC

TGTGAAGAAACTTCATTCAGTGATAAATGAGGAAATGTCAGAATTTAGTTCAAAGGCTTT

TGCAAGTGAGATTAAAGCATTGACAGAAATCAAACATCGGAACATTATAAAGTTGCATGG

ATTTTGCTCACATTCACAGGTCTCATTTTTGGTCTATCAGCTCATGGAAGGGGGTAGCTT

GGATCAATTGCTGAACAAAGATACACAAGCAACGGCATTTGATTGGGAAAAGAGGGTGAA

TGTTGTTAAAGGTGTGGCCAATGCTTTATCTTATTTGCACCATGATTGCTCACCTCCAAT

TGTTCATCGGGACATATCAAGTAAAAATGTTCTTTTGGATCTTGAGTATGAAGCTCATGT

CTCTGACTTTGGGACTGCTAAGTTTTTGAAGCCTAGTTCAGATAGTTGGACTC

>PvRGL314

AAAGTTGGGTCCTACAGGGGACAAATCTCATGTATCTTCAAGAGTAATGGGTACTTATGG

ATATTGTGCTCCTGAGTATCAAAGAACTGGTCAACTAACAGTAAAATCTGATGTATACAG

CTATGGAGTCGTTTTGCTTGAATTGATCACTGGAAGGAGAGTCATTGATAACTCAATACC

>PvRGL315

GTGGAGATCATAAGCAAAATAAAGCACAGGAATCTTCTTGCTCTTCGGGGTTGTTGCATT

TCTAGTGATAGTTTGAAAGGTAAGAGGAGGTTTCTGGTTTATGATTTCATGTCTAATGGA

AGCCTGAGTTACCAATTGTCAGTTGCTGGGGCTAATAGGCTAACATGGCCACAAAGGAAG

AACATAATCCTTGATGTGGCTAAGGGGCTTGCTTATTTGCATTATGAGATCAAACCCCCA

ATTTATCACCGTGACATAAAGGCTACCAACATACTTTTGGATTCAAAAATGAAAGCCAAA

GTTTCAGATTTCGGGTTGGCCAAGCAAGGGAATGATGGCCAGTCTCATCTCACTACAAGG

GTTGCTGGCACATATGGTTATTTGGCACCAGAGTATGCTCTTTATGGGCAACTAACAGAG

AAGAGTGATGTGTACAGTTTTGGCATTGTGATTCTTGAGATGATGAGTGGAAGAAAAGTG

CTTGATACCATGAATTCATCGGTTGTCTTAATCACGGATTGNGCATGGACACTGGCAAAA

TCAGGGAACACACAGGACATTTTTGATCAGTCAATAAGAGAAGAGG

>PvRGL316

TGAAGTCAACGGCGTCTTCATCTGGCTACACCCACTGGATCCATGACGTGTTCTTGAATT

TCAGAGGAGACGACACGCGCGAGACTATCGTGTCACATCTCTACGCCGCACTCTCGAATG

CGGGGATCAACACTTTCATCGACGATAACATTGTGAAGGGAACGGTGTTGGGGGCAGAAC

TGGTGCGAACCATACAAGGCTCTCAGATATCCATCATCGTTTTCTCCGAAAACTATGCTT

CTTCTAATTGGTGTCTTAACGAACTGCTAGAAATCATGGAGTCTCGCATAGCTTACGGCC

AGAAGGCGGTGCCCTTGTTTTACCATGTTGATCCGTCGCATGTTCGTAATCAGGAGGGTG

CTTTTGGACAACAGTTGGAAGCACTTGCACAAAATTGTTTACCTAAAGGGTTGAACGATG

AGATGTTAATGATATGGAAAAATTCACTCTGGGAGGCTGCAAATCTTGCTGGTTGGCATG

CAGTGAATTTTAGGTATATCTAATCTATGTTGTCTACTTATTTTGGATCTCTTCCAACCA

TTTAATGGCATTTTGACAAATTTTAAGTAAACACTTAGAAAAAAAATTGGGTAAATTGAA

ACTGATCTTTTTAGAGAAAAATTATGTAAATTAAATTATGTTTAGCT

>PvRGL317

GCTTTCACACCCATTCTCTCTCTCAGCAACTTTTGCTTTCTCTAATGGCTTCTTCTTCTT

CTTCTTCTTCTTCATCTCTTGCAACAGACATCTACGACGTATTTGTCAGCTTCCGCGGTG

AAGACACGCGCAACAGCTTCACTGGCTTTCTCTTTCAGGCTCTCGGTACAAAAGGCATCA

ATGTGTTCAAAGATGTTGAAGATCTCAAAAAGGGTGAATCCATTGCACCAGAGCTGCTGC

AAGCCATTCAAGCCTCTCGACTTTTCATTGTTGTCTTCTCACAGAACTATGCTTCCTCCA

CTTGGTGCTTGCGTGAACTCGCAGAGATATGTAACTGCTTTGAAACTTCGCCAAGACGTG

TTATTATACCTATTTTTTATGATGTTCGTCCTTCAGTGGTGCGCAAACAGAGTGGATGTT

ATAAGAAAGCATTTGCAGAACACGAAAAGAGATTGAGAGAAGATGAAGTGAAGATGGAAG

AAGTTCAGAGATGGAGACAAGCTCTCACACAAGTCGCCAATATCTCTGGTTGGGATATCC

GAAATAAGTGAGTAATCCTCTCTCGTTTCAACATTCATCACAGTTTGAATNTATTTACTC

CTTTCTAAACTACAATTCTCAGATGTTAGGAANTNTCNGAAACAGTAGAAAACACA

>PvRGL318

GCTCAGACACTGTTCCTTGTTGACCAAACCCAACCAACCCATTTATCTCTCACCCTTTCT

TCTCATCTCTCAGAAAAATCTTCTCTTTTTGGTTTCAATTTTTGTTCTTGTTTTCACCAC

TTTCCAAGGTGACCTTTTAAGTGGTTTAATTCAAAGATATTTATTCCATTTCATATTATT

GGCCGTGTTCGAATACTTCATCGCTAGTCATGACTTGCTTCCCATTCTCATTTAGTAGGA

AAGTGCCTTCTGCACCAAAACATGATCCAGATATTGATGAAGAATTCTCAGGCATTCAAA

ATGTAAGAATCTTTACCTTCAAAGAACTAAAAGTTGCGTCTGACAATTTCAGTCAGTCCA

ATAAAATTGGGCAGGGTGGCTTTGGTTCTGTTTATAAGGGATTACTAAAAGATGGGAAAC

TAGCTGCTATAAAAGTTCTTTCAGAAGAATCAACTCAAGGGGTGAAAGAATTTATGACAG

AGATTAATGTCATCTCAGAAATAGAGCATGAAAATTTGGTTAAGCTATATGGTTGTTGTG

TGGAAGGGAAAAACCGAATATTAGTCTACAATTTTCTTGAGAACAATAGCCTTGCGCAAA

CCATTTTAGGTTCAGGTCACAGTAACAATATTTTTGATTGGAAAACAAGATCTANGATTT

GCATTGNGATTGCACGCGGGCTCTCCTATCTACATG

>PvRGL319

GGAAGGTTTCATTCATTCTAGTTTCTGAGAACCTTAAAAAATGGGTTCAGAATCCTTAAC

CGTTTGCGTTACAGGGGCTTCTGGTTTCATCGGATCATGGCTTGTCATGACACTCATCCA

ACGTGGCTATACTGTTCGAGCCACTGTTCTTGACCCAGATGATACGAAGGAGGTGAAGCA

TTTGCTGGAGATAGGAGGTGCAAAGAGCAAGCTTTCACTGTGGAAGGCTAACCTTGAAGA

AGAGGGAAGCTTTGATGAAGCCATTAAAGGGTGCATTGGAGTTTTCCACTTGGCCACCCC

CATTAACTTTGAATCCAAAGACCCTGAGAATGAAGTGATAAAGCCTGCAATAAGGGGAGT

AATAGATATCATGAAAGCATGCTTGAAGGCAAAAAGTGTGAGAAGGCTAGTATTCACATC

CTCAGCCATAACCACCCAAATTTCTCATCACCAAAAGCCTCTGTATGATGAGACCTGTTG

GACTGATGTTGAGTTATGCAGAGCAGCCAAGATGACTGGTTGGATGTATTTCGTTTCTAA

AACTCTGGCAGAGCAAGAAGCATGGAAATTTGCCAAAGAAAAAGGAATGGACTTCGTCAC

TATCCTTCCAACTCTCGTTGTTGGCCCTTNTCTGCTCCCATCAATGCCATCTAGCTTAAT

CACTGCACTTTCCCCTATCACAGGANAAGAGCAGCATTATTCGATCAT

>PvRGL320

CGGAAATGCGTTGAAGATTGGATTGGGTGTTGGAGTGCCTCTGGTGGTTCTTCTTGCAGC

GGGTGTTGTAGGGTGGGTTTATTGGTTGAAGAGAAAGCAGAGAGAAAGTGAGACTCAGAT

TTTGGGAACTCTCAAGAGTTTACCTGGAACTCCCAGAGAGTTCAGATTTCAAGAATTGAA

GAAAGCCACTAATAATTTCGATGAGAAGCATAAGCTCGGACAAGGTGGATACGGCGTCGT

TTTTAGAGGAACACTTCCGAAGGAGAAGTTGGAAGTAGCCGTTAAAATGTTCTCCAGAGA

TAAAATGAAAAGCACGGATGATTTCTTGGCTGAACTCACCATCATCAATCGCCTTCGTCA

CAAAAACCTTGTTCGCTTACTCGGGTGGTGTCACAGAAACGGGGTGTTGCTGTTAGTGTA

CGATTACATGCCCAATGGAAGTTTGGACAACCACATTTTCTGTGAGGAAGGGAGAAGCAC

TAGCCCACTAAGTTGGCCTTTAAGGTACAAGATCATAGCAGGGGTGGCTTCTGCATTGAA

CTACCTGCACAACGAGTATGATCAGAAAGTGGTCCACAGAGACCTTAAGGNCAGCAACAT

CATGCTGGACTCAGACTTCAATGCTCGTTTAGGTGACTTCCGTCTGGCTCGTGCATTGGA

AAATGATAAAACATCTTATGCAAAGTTGGAAGGANTGCATGGCACCTGGGGTACATTGCA

CCTGANTGCTTCCCCC

>PvRGL321

TGACTATGGAGATTCTGGATTACCTTTTGTTCCTAAAATGTTGGGGAATTTAATCCACCT

GAGGTATTTAAGCTTTAGGGGAACACAGATAGAAAGTCTTCCAAAATCCATTGGTAAGCT

CCTGAACTTGGAGACCTTGGATATACGACAAACAAAAGTGTGTAAGATTCCAAAGGAGAT

CACCAAGCTTCAAAAGTTACGATATCTTCTGACACCTAACTGTGCATCTTCTTCAATTGA

ATGGAAGGATATTGGAGGCATGACAATGCTACAAAAGATACCACAAGTGCGTGTGGAGTT

TGATGAAGTGGCGATTAGAGAGGTGGGAAAGCTAAAGCAGTTAAGGGATCTGAGGGTGCT

TTGTTTCACCGGAGACCACAACAATGTTCTTTGCTCCTCAATACAGAATATGGAACACTT

GCATGCACTACGCATTGAAACATCTGGTCATCACCGCAGTGAAGTAGTCGACTTGAACAT

TACGTTACGAAATTCTAAACTGAAGAAGCTTTTCCTTGATATGAAGTTAGAAAGGTTACC

AAATTGGATTCCTCAACTCCATAATCTTGAGAGATTGACCTTACGCCACTCTGACTTAAC

CAATGATCCGTTGAAATCACTAAAAGATATGCCTTGTTTGACGGTTCTCTCTTTAACCCA

CGCTTATGAAGGTCAAACTTTGCATTTTCAACGTGGAGGGTTTCGGAAACTAAGGAAGCT

AATTCTCTCA

>PvRGL322

ATCATTTATAAATTCTTCTGTGAAATTTATCCATTTAAGCAATAATCATTTAGCAGGATT

GTCCAAAAAAATGTTCACTGAAAACTCTCCTATAGTGATGTTGGACCTTAGTTACAACAA

AATATCTGGTGGCATTCAGGATATGATTCAAGATCTCAATTATTCAAAGTTGAATTTTCT

CCTTTTAAAAGGTAATCACATTACTGGGGATATACCTAAGCAATTATGTCAATTGATAGA

GTTAACCATGCTAGATCTTTCAGACAATAAACTTTTAGGAGAAATTCCTCATTGCTTGGG

GACAATGCCTTTTGATAATAACAACCTTGATCCATTCTTAAAAGCATCCAAGGGATATTT

TGTTGAAGAACATAGTCTACGACCATCACAATCAGAGCATAAGAAAGAGAAAGCAAGTTT

CACTTCAAAGAAAAGAATTGATACTTACACGGGAAGCATCCTTATTTATATGTGTGGAGT

TGACTTATCCAATAATAAATTGAAGGGGAATATTCCTTACGAGCTTGGAAACTTGACAAA

AATCCAAACATTGAACTTGTCTCGTAATGATTTGATTGGACAAATTCCAGATTCATTCTC

GAAGTTGATGCAAATAGAGAGTTTAGATCTTTCTTTTAATAAGTTGAGTGGTGAAATTCC

TTCTAAACTCAATATTCTAATCACACTTGAAGTATTAAGTCTAGCACACAACAACTTATC

AGGTCCAATACCTGAATGGACAAATC

>PvRGL323

GCCTGATGGAAGAGAGATTGCTATTAAGAGATTATATTTCAACAACAGGCATAGAGCAGC

GGATTTCTACAATGAAGTCAACATAATTAGTAGTGTGGAACACAAAAATCTAGTCAGACT

CTTAGGATGCAGTTGTTCAGGACCAGAAAGCCTGCTTATATACGAGTTTCTAGCTAACAG

AAGTCTTGATCGCTTCATATTTGATAAAAACAAGGGTAGAGAACTAAATTGGACCAAGAG

ATATGACATTATCGTGGGGACAGCTGAAGGATTGGTTTACCTGCATGAGAACTCCAACAT

CAGGATAATCCACAGAGATATAAAAGCCAGCAACATCTTATTAGATGCAAAGCTTCGTGC

TAAAATTGCTGATTTTGGTTTGGCCAGGTCCTTTCAAGATGATAAGAGCCACATTACTAC

AGCTATTGCTGGAACTTTGGGTTATATGGCTCCAGAGTACCTAGCACATGGTCAGTTAAC

AGAGAAAGCAGACGTATATAGCTTTGGGGTGTTACTGTTAGAAATAATTACTGGGAGACA

GAATAACAGGAGCAAAGCATCAGAATATTCAGACAGCCTAATAATAATGACATGGAAGCA

TTTTCAATCAGGGACTGCGGAACAAATAATTGATCCATATCTATTGTTGGATGACAACCA

TAGAAGCAAAGTTAAGAATGATATTTTAAGAGTGGTGCAGATAGGACTTCTATGCACCCA

AGAAATCTCTTCCTTGCGACCATCTATGTCAAAGGCACTAAAGATGCTAACAAAGAAGGA

GGAGCATGTGGAAGCACCCTCCAATCCACCCTTTATAGATGAAAGTACCATGGAACTCCA

TGACCAAAATGA

>PvRGL324

ATATAAAGAGTTTCAAAATGAGATCAATTTTTTCTCATTCTGTCACATGAATCTAGTCTC

ACTGCTAGGGTACTGCCAAGAGGGAAATGAAATGATACTGGTGTATGAATACATGGCTGA

GGGTCCTCTGTCTGACCACTTATACAAGAAAAAAAATCAGCCGTTGCCTTGGATTCAAAG

AATAAAGATTTGTATTGGTGCAGCTAGAGGACTACATTACCTCCACACTAGCACAAGGCA

CCCAGTGATTCACCGTGATGTGAAGTCAGCCAACATATTATTGGATCAGAATTGGGTAGC

CAAGATTGCAGATTTTGGATTGTGTAGAACAGTTCCCTCACTTTACCATTCACATGTTAG

CACTGAGGTTAAAGGTACTTTTGGATATTTAGACCCTGAGTACTATAAGAGAAGAAAACT

GACTCAAAAATCTGATGTGTATTCATTTGGAGTGGTTTTGTTTGAGGTGTTGTGTGGAAG

AGCAGCTGTGAATACTGTGGCAGTGGAAGAGGAAAGTGAGAAAGTTGGGTTGGCCACATG

GGCCATGCACTGTTACAAGTGTGGAAGCATTGATGAATTAGTTGATCCTCATTTAGCAGG

GAATGTTAGGCCAGAATGCCTGAGGGCGTTTGTGGAGATAGGCATCCAGTGCTTGGCTGG

TAGAAGTACGGATAGGCCAACTATGGGAGAGGTCCTCAATAGTTTAGAGAGGATCCTTTT

CCTTGCACGATAGTTTAGAAATACAACTCAGTTTCTAAAATAGCTTATCACTAGTGTCTT

CCAAATATTTTATTACTCAATGTGATTAAATTATATGATCAATATTTCT

>PvRGL325

GGCGTCGTTAACATGGTCAACAAGAATGAAAATTGCTGCTGGAGCAGCAAAGGGTCTAGC

CTTTCTACATGAAGCAAAAAAACCAGTCATCTATAGAGATTTCAAAGCTTCAAACATATT

GTTAGACTCTGTAAGTAATGAATTGATCACTTTCTCTGTCATACATACTCTAGTGTTGCG

TCTGAACAATTGAACCCCTTAATTGTTTATTAACTCACTCTGGCCATTCATTCTTTCAGG

ATTATGATGCAAAGCTTTCTGATTTTGGCTTGGCAAAAGATGGTCCCGAAGGAGATGACA

CACACGTTTCAACTAGAGTGATGGGCACACAAGGGTACGCGGCCCCAGAATACATCATGA

CAGGTAAAAATAATTTCACTAAAAGTAAAACACAATGAGCATGGTTCACTTGAACAGCAA

TCAATGAATTAATTGGTCACTCAATTATTCTTACACGTACAATGTTTTTTTCTTCCATAT

AGGACACTTGACAGCAATGAGTGATGTATACAGTTTTGGAGTTGTTCTCTTGGAGATTCT

AACAGGAAGAAGATCAGTTGACAAAGGGCGCCCGCCAAGAGAACAAAATCTGGTGGAATG

GGCCAGGCCTGTTTTGAATGATCCCCGGAAACTTAGCCGAATAATGGATCCAAGACTCGA

GGGACAGTATTCTGAGGAAGGGGCAAGAAAAGCAGCTGCATTGGCTTACCAATGTCTCAG

CCACAGGCCAAGAAATAGACCCTTGATGAGCACTGTGGTTAGTGTTCTTGAGCCTCTTAA

GGACTTTGATGATGTTTCCATTGGACCCTTTGTTTACACTGTTCCCACTGATCACAATGA

AATGGCAAAAGAGGTTTGAACACC

>PvRGL326

AAATGTAGGGATGTCTATCAGATGGGACATTAATAGCAGTAAAGCTACTCTCTTCTAAAT

CAAGACAAGGGAATCGTGAGTTCTTAAATGAGATAGGAATGATTTCTGCTTTGCAACATC

CTTATCTTGTTAAACTCTATGGATGTTGCGTAGAAGGAGATCAACTATTGTTGATATATG

AATATATGGAAAATAATAGCCTTGCTCGAGCTTTATTTGGTAAATCTACTTATTATATCA

TGAACTTTTGATTATTATCACTTATATTAACTTATGAATATGACTATATATGTTACTTTT

TTCCGTTTTATATCATTTTCTCAAATGAAGGTCCTGAAGAATTCCAAATAAAATTGGATT

GGCCAACAAGGTACAAGATTTGTGTTGGTATTGCTGGAGGTTTGGCATACCTTCATGAAG

AATCACGATTGAAGGTTGTTCACAGGGACATCAAGGCCACTAATGTGTTGCTTGATAAAG

ATCTCAACCCAAAAATATCTGACTTTGGTTTGGCAAAGCTTGATGATCAGGAAAACACTC

ATATTAGCACCCAAATAGCTGGAACATAGTAAGTTCCTATCTCTTTTGTGTAATTCAATA

GAGAAATATTTTTCATGTATATGATTGTACTCTTAAGAAACAAAAAATTAAAAGGATTCA

TAGATGATTCACTTAACCAAATTCATTTATTTTTTAAATGCAATAATCTGGTTTGGATAA

GTGGATCAAAGAAAAATTTTGTCTCTAATGCAATCCAACATTTAAAGTAATATCTTCTAA

GTAGTGNGATGAGAGTAGTTTTTTTTTTTGTTATATGGAAAAAAATACAAAATTATTAT

>PvRGL327

GCATGCAAACGGAAATGGGTTGACCTTATGGAGAGACTGAACATAGCAATAGATATGTGT

GTGCATTGGACTACNTGCACAATGATAGTGAGATCCCAGTTGTGCACTGTGATNTGATGC

CTAGCAACATTCTCTTGGACACAGACATGACGGCAAAGGTTGGAGACTTTGGATTGGCTA

GGTTGCTTATTCAAAGATCAACCAATCAAGTTTCCATTAGCTCCAGTCATGTTCTTAGGG

GTTCTATCGGCTACATACCTCCAGAATATGGATGGGTAGAGAAAGCATGTGCAGCTGGAG

ATGTATATAGTTTTGGTATAGTGTTGCTAGAGTTATTCTCTGGCAAAAGTCCAACTGATG

AATGTTTCACTGGAGGGCTAAACATAAGAAGATGGGCGCATTCAGCATTCGAAGAGAAGA

CAATGGAAGCCATTGACCCTAAGTTGGTGTCCCTCATTTTGCATGATGACCCTTCAGAGG

GACAAAATAACGTACAAGTGTATTGTGTGGATGGAATTCTAGGAGTAGCCATAGCTTGTA

CTGCAGACAACTTAGATGAACGCATTGGCATTAGAGATGCTGTTCGCCAACTCAAAGCTT

CTGAAGACTCTTTGAATCACTCTTATCACACTTCTACTGCACACATCGTTTCGCTAAAGT

AATGCTGAAATGTTATGATCTGGGAAATGCATAACGTAATAATACTTTTGTTGACCATGA

AATGATCAAGTTTTTAGTTTGTCTAAGTCAAAGAATATGATAGAAATTGAGGCGCTAATA

ATAGATTATAAGTGTTTGTGTTTGTGGTTAATTACACTTGTAAAACTTATGTATCTCTTC

TAATTATTTATGGCATTTTTTTTAGT

>PvRGL328

TCGTTGGTGGCTGAAGAGGTTATTTATGGCAGAGATGAGGACAAAGAAAACATCTTTAAT

TGGCTCACATCTGACAATGAGAACTATCATAACCAACTGTCAATACTTTCTATTGTGGGT

ATGGGCGGGATGGGTAAGACCACACTTGCTCAACATTTGTATAACGACCCAATGATAAAG

GGTGAATTTGCTATCAAAGCCTGGGTCTATGTTTCTGATGAATTCGATGTTTTCAAGTTA

ATCAGAGCAATTCTTGAGACAATCCATAAGTCAACCGATGATAGCAGAAACCTAAAAATG

GTTCAGGGACAATTGAAAGAATCGTTGATGGGAAAGAAGTTTCTACTCGTTCTGGATGAT

GTCTAGAATGAAGACTGAAGTCAATGGAAATCATTGCAAACTCCTATTAAATATGGGACT

AAGGGAAGTAAAATTCTTGTCACAGCATGCAGTAACAAAGTTGCTTCTAAGTCTGTAAAT

TCATCAACCAACTTCATATTTTTCCATCAATTTTCTTCATCCATTTTACCCACACAATGC

ATCCATAAAACTACAACGTATAAAAAATTACAAAGAGTTACGTAGATTCAACATAGGACT

ACATTATTTCCAGTTTGAAAACAAATAATTTTTTATTTTATTTATTAGAGTATGAATAGG

CTGGTTTTTTCCTTTTAAAAATTAGAGCAACAAGAAATTATTTATATAAATGATGAATAA

AAAATATTTTTTAATTT

>PvRGL329

GATGAATGCAAGGTAAAGTGTTGGGAAAATTGTTCGTGCACGGCTTATGCAAACTTGGAT

ATAAGAGGAGCAGGAAGTGGGTGTTCTATTTGGTTCGGTGATCTTATTGATTTGAAGGTT

GTTTCACAAAGTGGGCAATATCTGTATATTCGAATGGCAGATTCAAGGACAGATCCTAAA

GATGCCCACAAGAAGAAGGCATTGTTGCTGATAGGTACCACCGTGGCTCCTACTGTTCTT

GTCATTCTATTGGCAATCTTATACTATTACAGGAGAAAAAGAAAGTATGAAGAAGAAAAC

GTGTCAGTAGTAAAGAATGATAAAGTTGTTGGACAAGAAGATAGTATGGAACTACCGTTG

TTTGATTTTGCTACATTAGTTAATGCTACCAACAACTTCTCAACTGACAACAAACTTGGG

CAAGGTGGGTTTGGGCCTGTATACAAGGGTGTATTAGCAGATGGACAAGAAATTGCTGTT

AAAAGGCTATCAAGGAGTTCAGGACAAGGATTAACAGAATTTAAGAATGAAGTTATATTG

TGTGCCAAACTGCAACACCGAAATCTGGTTAAAGTTCTTGGTTGCTGCATTGAAGCAGAG

GAGAAAATGCTTCTCTATGAATATATGCCCAACAGAAGTTTAGATTCATTTCTTTTTGAT

TCAACTAAAAGTGAGGTTTTGGACTGGTCTAAGCGCTTTCATATCTTATGTGCAACTGCT

CGTGGACTTCTTTATCTTCATCAGGATTCTAGATTAAGAATAATACATAGAGATTTAAAA

GCAAGTAATATTTTATTGGACAAACACTTGAATCCTAAATTTCAGATTTTGGGTTTAGCA

AGATGTG

>PvRGL330

CAAGAGAATCTATATTGAAACATCTCTAATCAGATATCTAACAGTGAAACCTACTGTTGCGTTACGATTAAGCAAGCTTGTTTCATGAGCTTGTCCTTTACAAGTCTATAAAGTGTCTCATTCCTCTGCAAGGAGGGTGAGAACCACCGTTCTGAAGACAAACCACTAATAAAACAGTAAAAGAGCAAATGTTCTGCAGAATTTAGAAGATATAATACTGTAAATCACCTCCCTTCAGATTTCACCAGCTGTGAGAATACCCTACTCATGGAAACTCCAGACAGATCATCCAGACTTGAAGCTTCAAATTGTGTAGCGGAAACACTTTCTTCATGGTCGAAATTGTTGACCTGGGGTGACAGTTCTCCGATCATATTAGTACTGTTTTCTTCAGGGTCACCTCGAACAATAGCCTCTTGAAGTTGGAGAGCATACTCCAAATTCCACAAGACATCCCCCATGGAAGGCCTGTCAACACCAAAGTCAGCCAAACATTTTTCAGCAGTTTCTCCAAACTTCCTAAGAGAATCTGGTCTGATTTTTCCATTAAGTGTTGGATCTATTATTTGCTCCAATTGCCCTCTTTTCTGCCATTTCATAGTCCATTCTGCCAAGTTCACCATTTCTCTGGGAAGAGTTGGATCTATAACAGGTCTAGCACAAAGAACCTCAAACAGAACTACTCCAAACGAATACACATCTGACTTTTCTGTAAGTTGTTGCCTCCTGAAATACTCAAGATCAAGGTACCCAAAACTCCCTTTGACAGCGGTGCTCACATGTGTCTGATCAATTTCAGGCCCTGTCTTCGATAGCCCAAAATCAGCAACTTTGGCCATGAGGCTGTCATCAAGCAGGATATTTGCAGACTTCACATC

>PvRGL331

ATGGAAAGAGAGAGTTCAAATTGCCTTAGATGCAGCAAGTGGATTGGATTATCTGCATAATGGATGTAAGCCACCTATTATTCATAGAGATTTGAAAGGTTCCAACATCCTAGTAGATGAAAACATGCATGCCAAGATTTCAGACTTTGGATTGTCTAGAACTTTTGCAAATGATAGTGACACTCATGTCTTAACTAATTATCCAGGTGGCACACCAGGTTACATTGATCCTGAGTATCACTCCTCTGGGACTCTTAATAAAAGAAGTGATGTGTACAGTTTTGGGGTAATTTTGTTAGAGCTAATTACAGGTCAAGCAGCAATAAGAGGAACTCCTGAGAAACCTAGTCACATACATTCTTGGGTTAAACCCAAACTTGAAGCTGGAGATATCCAAGCAATAGTTGATCCAAGATTAGAAGGAAATTACAATGTTGCTTCAGCTTGGAAATTTTTGGATATAGCCATGTCTTGCCTTCCTGATGTTGCCATTCAAAGGCCTGACATAAGCCACATAACCTCAGAACTCAAGGATTGTTTGTCTTTGGAACTATCCCTTCAAAGGACTGTCACCAACAACAACATACATTCATTCCTCATGGATTCTATGCTCATTGATTTCAATGAATCTGATATAGGTCCATATCCTAGGTAGAATTTCTCCCACCTAAATTACCTCAACATAAATTGATTTATTTCTATAAACAATGTTTTTCTTTGTAATATTATTGTAACTAGTTTTTGGTAAGGATATACATTAAATTTTATTTTTAATTTGTAATTTTCGCTTTCAAATTATATATATAGTAAACCAGTTGTTATT

>PvRGL332

CCGTTGCTGTCGTCAAGACACACACTCAACTAAAATTGCTTTATAATGTTCGTATGGTTTCGGTATGTATATACAGATCCAATAAAGAAAACACGGTTGGATTGGCAAATGCGCTACCAAACAATTAGAGGTATTGCAAGAGGCATTCTCTATCTCCACGAGGATTCTCAATTGCGAATTATTCATCGAGATCTTAAAGCAAGCAACATTCTCTTGGATGAAAACATGTATCCTAAGATATCAGATTTTGGGATGGCAAGATTGGTTCAAGTAGATCAGACTCAAGCAAATACACACAGAATTGTTGGAACCTAGTAAGTGAGAGAATATAATATAATTGTTCTAGAAGCTTAATTTGAATCAACTGTCAAGTAAAATATTGTACACTGAAGTTTCATAAATTTTATGAGTAACTAAACGTGCATGTGGTTGGCACGCAGTGGATATATGGCACCTGAGTATGCTATATATGGTCAGTTTTCAACAAAATCAGATGTTTTTAGTTATGGTGTACTCGTTCTTGAGATTGTAAGTGGCAGAAAAAGCATTGGTTCTCGTCACGGGGAGAGAGTGGAGGATCTATTGAGCATTGTAAGTCTTTCTTTTCTTGTCACTGTATCTTCCCTCTCCTTTGTCAGAATAATAGGATTAGAAAATTATTTGTTCCTTCACATAATAACTGTATGAATGAAATCTGACGAACATGCATTTGAGCAATTCATACAGACATGGCGAAACTGGAAGGATGGAACAATTGCAAATATTGTAGACCCCACATTAATCAAGGGTTCCCAAAATGAAATAATGAGATGCATCCACATTGNGTTACTATGTGTTCAGAAAACATGGATGCCAGACCAACCATGACTAGTGTTATGCTCATGCTTAACAGCTATTCTCTCACTCTCCCCGT

>PvRGL333

GCTAGCTTCACTCACGAAGTTGAGGTTATAGCGAGTGTTCGCCATGTGAATCTTGTTACCTTAAGAGGTTATTGTACTGCCACGACCAACTTAGAGGGGCACCAGAGGATTATTGTGACTGATTTGATGGAAAATGGGAGTCTTTATGATCATTTATTTGGTTCTTCTAAGAAAAATCTGACTTGGCCTATTCGTCAGAAGATTGCTCTCGGAACTGCTAGAGGGTTGGCTTATTTGCATTATGGGGCTCAACCTTCCATCATTCACAGGGATATTAAAGCTAGTAACATACTTCTGGACCATAAGTTTGAAGCCAAGGTGGCGGATTTCGGGTTGGCCAAGTTCAACCCTGAGGGAATGACGCATATGAGCACTAGAGTGGCTGGGACTATGGGGTATGTTGCTCCTGAGTATGCCTTGTATGGACAGTTGACAGAGAGAAGTGATGTGTTCAGTTATGGTGTTGTCTTCTTGAGCTTTTGAGTGGGAAAAAGGCTCTTCAAACCAACGATGATGGGCAACCCTCTGCACTCACTGACTTAGCTTGGTCGTTGGTTAGAAACGGTAGTGCTTTGGATGTTGTTGAAGATGGCATTCCGGAGCCTGGTCCACCAGAAGTTCTCGAGAAGTACGTGTTGATTGCTGTCTTGTGTTCTCATCCACAGCTATATGCTANGCCAACAATGGATCAGGTTGTTAAAATGCTGGAGACAGATGAATCGGTTCCCTCCTTAATGGAAAGGCCAATTCCTTTTATTGCTGGGAGGATTGACATTGAGAAATCTGCGCTGAAGTACTCGGGTCAGCTCTGCAGTCCAACCGGTTATCTAGCATATA

>PvRGL334

AACTGAACTCGAGACTGTTGGCAGCATTAAGCACCGGAATCTGGTCAGTCTCCAAGGCTACTCTTTGTCCCCTTATGGCCACCTTCTGTTTTATGACTACATGGACAATGGCAGTCTGTGGGATCTTCTTCATGGACCTACTAAGAAGAAAAAGCTTGACTGGGATCTGCGTCTAAAAGTAGCACTTGGAGCAGCACAAGGGCTTGCCTATCTGCACCATGATTGCAGTCCTCGAATAATCCACAGGGATGTGAAATCATCAAACATTTTATTGGACTCAGACTTTGAGCCCCATCTTACTGATTTTGGCATTGCCAAAAGCCTCTGCCCCTCAAAGTCCCATACTTCTACTTACATAATGGGCACAATTGGCTACATAGACCCTGAGTATGCTAGAACTTCCCGTCTCACCGATAAGTCTGATGTGTACAGTTATGGCATCGTTTTACTCGAGTTGCTAACTGGAAGGAAAGCTGTTGACAATGAATCCAACCTCCACCATCTGATTTTGGCCAAGGCAGCAACCAATGCTGTAATGGAAACAGTTGATCCAGACATTACTGCCACATGCAAGGACCTTGGAGCTGTAAAAAAGGTTTACCAGCTCGCTCTATTATGCACAAAGAGGCAGCCCGCTGACAGGCCGACAATGCATGAAGTGACACGTGTACTTGGGAGCCTTGTGCCATCAAACACCCCTCCAAAACAACCAGCAGCACTACCTCCTGCTTTCAACCCATCTGCCAAAGTGCCATGCTACATGGATGAGTATGCAAAACCTCAGACTCCACACTTGGTGAACTG

>PvRGL335

CCGTTGCTGTCGCTTGATCATTCTTAATGACCTGCACAGTACTAATGTCAATCTCTCCGAAAGAAGGATCAAGGGGTGGAGGTAGGTTGTTATTAGTACTGTGCAGGTCATTAAGAATGAAGATCTTGAAGAGCTGCGAGAACTAGGTTCTGGTACCTTTGGGACCGTGTATCATGGAAAATGGAGAGGAACAGATGTTGCTATTAAAAGAATAAAGAAGAGCTGCTTCACGGGTCGGTCATCTGAACAAGAGAGATTGACTGTAGAGTTTTGGCGGGAAGCTGACATTCTTTCCAAGCTTCATCATCCAAATGTGGTTGCATTTTATGGTGTGGTACAGCACGGACCAGGTGGTACAATGGCCACTGTGGCAGAATATATGGTTGATGGTTCTCTTAGGCATGTATTACTTCGCAAGGATAGGTATCTTGATCGTCGCAAGAGGCTGATAATTGCAATGGATGCAGCTTTTGGAATGGAATATTTGCACTCAAAAAACATTGTGCATTTTGACTTAAAATGTGACAATTTGCTTGTAAACTTGAAAGATCCTTTACGTCCAATATGCAAGGTTGGTGATTTTGGCTTATCAAAAATTAAGCGAAATACTTTGGTTACTGGTGGTGTGCGCGGGACATTACCTTGGATGGCACCAGAGCTTCTGAATGGCAGCAGCAATAAGGTCTCAGAAAAGGTTGATGTGTTCTCCTTTGGCATAGTATTGTGGGAAATTCTTACTGGTGAGGAGCCATATGCCAATATGCACTATGGTGCAATTATAGGTGGGATTGTAAATAACACATTAAGGCCAACCATCCCAAGTAACTGTGATCCTGAATGGAGAACACTAATGGAACAATGTTGGGCTCCC

>PvRGL336

TGCCATATGAAGAATGGAATATCGATTTCACAGAATTGACTGTGGAACTCGCGTTGGGATTGGGTTCTTTGGAGAAGTTTTCCGTGGCATATGGAATGGCACAGATGTCGCAATCAAAGTTTTCCTAGAGCAAGATTTAACTGCTGAAAACATGGAAGATTTCTGCAATGAGATATCCATTCTCAGCCGGCTCCGACATCCTAATGTTATCTTATTTCTTGGTGCATGCACAAAGCCCCCACGCTTGTCAATGGTAACTGAATACATGGAGATGGGCTCTTTGTTTTACTTGATTCATGTGAGTGGTCAAAAGAAGAAGCTTAGTTGGCGGAGAAGACTAAAGATGTTGCGGGACATTTGCCGGGGCCTAATGCACATACATCGTATGAAGATTATTCACCGTGATGTGAAAAGTGCAAATTGTCTTGTGGACAAGCATTGGACGGTGAAAATATGTGATTTTGGACTTTCAAGAATAATAGAAGATTCTCCCATGAGAGATTCCTTGTCAGCTGGAACCCCGGAATGGATGGCTCCTGAACTTGTTCGAAATGAACCATTCACTGAAAAATGTGATATATTCAGCCTGGGGGTCATAATGTGGGAGCTCTGCACCTTGAATAGGCCATGGGAAGGCGTGCCTCCGGAGAGGGTGGTTTATACCGTTGCTAACGAGGGTTCTCGATTGGATATTCCTGAAGGCCCGCTAGGCAGGCTGATTTCAGAGTGTTGGGCGGAACCACATGAACGGCCAAGTTGTGAGGAGATCCTCTCTCGCTTGGTGGACATCCAGTACTCTCTGTTTTGATGCAGTGTTTATCGTTTTGTACATAGTGAATTTCGGGTAGAGTTGTTACTTTAGTGTTTTCGGCTGATGTAAACTTCTATGAATTTTTGTTCTTTATCTAAAGCATAATGAAACATGCATCGGTGCGTTTCTAACT

>PvRGL337

ACATGAGGAATGGGAGCAAGTTGTGGTCCACAGAGACATTAAGGCTAGTAATGTGCTACTTGATTCCGAATTGAATGGTAGATTAGGGGACTTTGGTCTTTCAAGGTTGTATGAGCATGGAACTGACCCTCACACTACTCATGTGGTTGGAACACTTGGGTATCTAGCACCAGAGCACACTAGAACAGGTAAGGCCACTACAAACTCTGATGTGTTTGCTTTTGGTGCATTTATGCTAGAGGTTGTTTGTGGAAGAAGGCCAATAGAGCAAGTGGGGGGGTCTGGGAGTGAAATTCTGGTTGATTGGGTGTATAATTGTTGGAAAAGAGGTGAGATTGTTGAGGTAAGGGATCCAAACTTGGGCACAAATTATAAGGCAGAGGAGGTGGAGTTGGTGTTGAAACTAGCTTTGTTGTGCTCACATTCAGATCCTTTGGCTAGGCCAAGCATGCGCCAAGTTGTGCAGTACTTGGAGAGAGATGTGCCTCTGCCAGACCTGTCTTTGCTTAGCTTATCTCCCACTGGCTTAACATTTGGTCTGCATGAAGACTTTCAGGATTGTCCAATGTCTTATCCTTCATCCATGGATAGGCCAATCTCCCATACTTCTTCAATTGCTGAATCACTTCTCTCTGGGGGGCGTTGATTTTACAAAAAACACACATCAAATGTGCATGCCTCATTCCTCCATTGTATTTCATTTCATTGCATTCTTAATATTTGTTATTTCATTTTGTATTATATTCTAAAGAGAGATGAGTTATCATACATC

>PvRGL338

GATGAATCTAAAAGGTCACAACTAGACTGGAAAAAGCGATTTGATATCGTATGTGGGATTGCTAGAGGAGTCTTATACCTTCACCAAGATTCACGATTGAGAATCATTCATAGAGACCTAAAAGCCAGCAATGTCTTGCTGGACTCTGCATTGAACCCCAAAATTGCAGATTTTGGTATGGCTAGAATATTTGGTGGAGACCAAATTGAAGCAAACACAAATCGTGTTGTTGGAACCTAGTAAGTAATCAACAAGCACAACATTAATAATCTTCATTGCATTCTCTGTTACTCAGACAGTACATTAGTAAATATGGTGATTTCATTTTTGTTTTCCTTTTGTAGTGGCTATATGTCACCAGAATATGCCATGGAAGGACAATTTTCTATAAAGTCTGATGTATACAGCTTCGGAGTTTTACTTCTAGAGATTATTACAGGCAGAAAGAATAGTGGTCAACACGAAGACATTACAGCCACAAATTTAGTTGGACATGTTAGTATTTTTATGAGAACTTCTGCTGCAATTTAAATATATCCCTTCAATGATCCAATTGACCTGATTTTCAATTAATGTAGATATGGGATCTGTGGAGAGAAGGGAAAACCATGGAGATTGTTGACCAATCACTAGGAGAGTCGCGCTGTGACCTTGAAGTCCAAAGATGCATACAAATTGGTCTTTTATGCGTGCAAGATTATGCCGCTGACAGACCATCTATGTCAGCAGTTGTTTTCATGCTGGGTAATGACTCAACTCTTCCTGCTCCAAAACAACCAGCGTTTATTTTCAAGAAAACTAATTATGAGAGTTCAAATCCATCAACCAGTGAAGGAATTTATTCAGTANATGATGCAAGTATAACTATGATTGAAGCTCGCT

>PvRGL339

TCATTATATTGGATATTGCACATAACACAATCTCTGGTCATATACCCAATTGTCTTCCTAATATCAAAACCCTACTTTTCAACAATGTTTCTAAAAACAAACTTTCTTTTTCCTTTCNTACATCTCGTAATGCTTACATCTATGAAAGTGACAATCTTGAATTGGTTACTAAAGGTCGAGTATCAAAATATGACCGAAACCTACACTTTATGACCCTAATTGATATGTCAAGTAACAATTTGTCTGGAACAATACCTCCACAAATGTTTAGTCTTATGGGATTGTCTTCCTTGAACTTATCCAACAATAATTTATCAGGAAAAATACTAAATGAGATAGGAAATATGAAAAACTTAGAGTCTCTTGATTTTTCAAAAAACCAACTTGGGGGTGAAATTCCTCAGAGCTTATCCAGTTTGTCCTTTTTGGCTTACTTGAATATTTCATTCAACAATTTGAGAGGCAAAATACCATCAGGCACACAACTTCAAGGATTCACTGCTTTTAGTTATATGGGCAACAGTGATCTTTGTGGACCCCCACTTACCAAAATGTGCTTTCAGGATGATAAACATAAAGACACAAAGCTTGTTGGGGAAGATGGAAATCAATATGAATTTTGGCCATGGTTTTACATCGGAATAGAATATGGATTTGTAACGGGCTTTTTGGGAGTTTGTTGTGCCGTTTTCTTCAACACAAAACTAAGACATGCTTACTTCAAGTTTCTTAATGACTTGAGAGATCGACTTCGTGTCTTGGTGGTTGTCAACATGAATCCCTTCCGTTAAGCTCAAACAAGTTGCATACAAATACTGGTAAGTGAAAACCTTTTGTT

>PvRGL340

GAATCAAAAGGCAACGGACAAGAATTTATCAATGAAGTGGCAAGTATTAGTAGAACTTCACATGTTAATATTGTTAGACTTTTGGGATTTTGTTTTCATGGTTCTAAGCGTGCTTTAATATATGTGTTTATGCCAAATAGATCTCTTGACAAGTTTATATATGAACAGAAAAATTCATTACAAGTTGCTCATCAATTGGATTGCAAGATATTATATGATATAGCAATTGGCATTGCTCGTGGATTAGAGTATTTACATAGAGGTTGCAATACCAAAATCTTGCATTTTGACATAAAACCTCACAATATATTACTAGATGAGAATTTTAGTCCGAAAATTTCAGATTTTGGACTAGCAAAAATATGTTCAAGAAAAGAAAGTGCGATATCCATTACTTTTGCAAGGGGAACTGTAGGTTATATTGCTCCAGAAGTTTTTTCCAGGAACTTTGGTGTTGTGTCACATAAATCAGATGTTTACAGCTATGGAATGATGATCTTAGAAATGGTTGGAAGAAGAAAGAATATTGAAGTTGAAGTCGATTGTTCAAGTGAATTATATTTTCCTCATTGGATTTATGATCGTCTTGAGTCAAATAATGAACTTGATCTAGAGATTGTGAAAAATGAAATTGATGAGAAAACGATGAGAAAAATGGCTGTGATAGGTTTATGGTGCATACAAACCAACCTCTCAACTCGACCAACCATAAGTAGTGTGGTGGAAATGTTAGAAAACAGAGTTGAGTTATTGCAACTTCTGCCTAAACCGTTTTTATCTTCTCCTTCACCATCTCCGGTTCATTTATCTTCGCCGATTCATTTGTTAGACCAGACACTATAATTTATGAGATTAAGATGTGGATC

>PvRGL341

GTGGAAGACCCAATGGTAACATTAGAGGAGCTTCCTAATTTGAAGTTTCTAAACGGGTGGGATATGTTTGTTGGAAAGAAAATGGCATGCACACGAAATGGTTTTCGTCAGCTAAAGGTTATTATACTTCGTGGCTTGCCTAATCTAGATGAGTGGACAATAGAGAATGAAGCCATGCCTAGTCTTTACCGATTGAGCATATCTGATTGCAACAATTTAAAGACGATTCCTGATGGGCTAAAATATGTTACTAGTCTTCGGGAGCTAGAAATCAGGTGGATGCCTAAATCATTCAAGACCAGGCTTGGAACTTCTGGAGAGGATTACCACAAAGTCCAGCATGTTCCATCTATTGTATTTTTGAACTAAGGATTGAATCAATAACTATCCTCCTTTACCCTCGTGCTTCCAGTCTCCAGAATCTGAGGTTTAAACTTGGAACTTGCTATGTTCGTGCCTGGTTTTTTTTGCATTGTCATCGATGGATTCAATTATTTTTATTTAGTATGCTTTTTTATATTTGCGAGATTGTTTGTAGAATAGATTTTCCGAATCCTATTTAGTGAAAAAGCTTGATTGTTTTATTATCACAAACTTGTAAATGTAAACATTGTTTTCTATGTTTTCAGAATGTATTACATACTTCATTTCAT

>PvRGL342

GAAATGTGAAGTGAAGTTGGAATACTTGGAGCCGGAGTATTTTATGACTGCAAGGTTATCAGACAAATCTGATGTGTACTCTTTTGGGGTGGTAATGTTAGAGATTTTGTGTAGGAAGAAAGCATGTTTTTTAACCCCAAGTGGAGATTGTGAGTACCTTGTTAAGTGGGCATTTGATGATGAAAGGAAGGGGGTTTTTGAGAAGATTGTTGATCCATCTCTTATTGGAAAAATAGCACCTGCTTGTTGGGAAGTGTTCATTGAGATTGTTCAGAGATGTTTGGCTTCAGTTGAAGAGAGGCCAACAATGGGTGAAGTGACGATGGTTCTTGAAAATGCATTTCTGTTGCAGAGAGAGCAGATGTGTGAAGGAGTGTAATGAGCCTAGAATCTGTGAGGGGGTCAATTCAAACTAATCTTTGAGTCTATCACTCGGTGTTACACTAAGCGACTCCCTCACCTTGAGGCGGGTTTCAATATGCATGTACATTTTATTTACGAGTTTATTTGATTATAAAGATAATCTTTCCTGC

>PvRGL343

TCATTCACCGAGATTTAAAAGCCAGCAACATATTGCTGGATACCAATATGAATCCCAAAATATCAGATTTTGGAATGGCAAAAATATTTGGTGAAAATGAACTTCAAGCAAATACAAACAGAATTGTTGGAACATATGGTTACATGGCCCCTGAATATTCTATGGAAGGACTTTTCTCAATAAAATCGGACGTGTTTAGTTTTGGGGTACTTTTGTTGGAGATCATAAGTGGCAAGAAGAATACTGGCTTTTATCAAACCGGTTCCTTTAATCTTCTTGGATATGCTTGGGATCTTTGGACAAGCAATTCAGGAATGGACCTAATGGATACTGCACTAGACGATAGTGATACTAGTAGTAATTGTATGCATACTGTGCCAAGATATGTGAACATGGGACTTCTTTGCGTTCAAGAAAGTCCAGAAGATAGGCCAACCATGTCTGATGTTGTAGCAATGATTGGGAATGACACTGTACCCCTTCCTTCTCCTAAACCACCAGCATTTCTAAAGTTGAGAGGTGATGACAGTTCAACACTGCCCTGCAGAATGACAGAAAGTTTATCAGTGAATGGTATGACAGATACAATTGTGCAAGCAAGATGAGTTCTATATTTAAAGCTTTTCTATTTAAAGTTTTCTCAATTAATGTTTCTATTCCTCTTCTATATTTACCACGGAATCAGATTTAGAAAAACTGAGTTTGATTCCAAAAGTTAAATGATGTTTAAAATACTTTTTTGGAATCTTAGCTTGTATTTTTAAAGATTAAATATGATGCAAGGTTGCAACATATAAGAGATATGTTGGAACCAAATCTAGCTGGTTTTATATT

>PvRGL344

GTTATTAGTATTTCTTGGGTGACCATCAAAAGGAGAAGAACGGAGGATGGTGTTGGTTTCGATAGAGAAGTTATGCCTAGAAGGTTTGGTTATGGCGAAATAGTTTCAGCCACCAACGGGTTTGCAGATGATAGAAGGCTGGGAGAAGGAGGGTCTGGAGAAGTTTACAAAGGGTTTCTGAATGACGGAGGGCGCGTGGTTGCTGTGAAAAGGATATTTTCTGATGTGGAAGATTCTGAGAGAATATTTAGAAACGAGGTGAAGATTATAAGTCGTCTTATACATAGAAACCTTGTGCAATTCATGGGGTGGTGCCAGGAACAAGGGGAGCTGTTACTGGTTTTTGAGTACATGAGTAATGGAAGCCTTGACAATCATCTTTTTGGCAACAGAAGAAGGTTGACATGGGATGTTAGATACAAGATAGCATTAGGTGTGGCTAGAGCACTTCGTTATCTTCATGAAGACGCAGAACAGTGTGTTGTTCATAGGGATATAAAGTCAGGTAATGTTTTGTTAGACACAGATTTCAATACTAAGATTAGTGACTTTGGGATAGCATAGTTGGTGGATCCAAGATTGAGGACTCAGAAGACAAGGGTGGTGGGGACATATGGGTACCTAGCTCCAGAATATATAAACGAAGGAAGGGTTAGCAAAGAATCAGAACAGGTTATAAATGTTCTAAAACAAGAAGTGCCATTGCCAATGTTTTCTGCAAAACTCTCTGATAATGGGCAACCTGATTCGCTTGAATCGTCATCCACAAAAGCATAAATCCTCCCATTTCAAATCTTTTGTTATTTAAAGAATCACTCCACCTAAAGAGGATGTACCAATAAGAGGTAGTTCACATGAATTTCTTCTTAC

>PvRGL345

AGGATATTTGTGAAATAAAGAAGGGAATTGAGTTTTTGGGATTCATGGGAATGGGGGCAATTGCAGAGAGTGTGGTTTCAGTGTTGTTGTTAATGTTTGGGTTAACAATGGGAATGGGTTTGAGTTCGAGACCGGAATGGGTCGCGTTAAGGGAGCTTCGATCGTCGTTGGGGATTAGGGCAAAGTATTGGCCCATAAAAGCAGAACCGTGCAGGAACTGGACCGGAGTTCAGTGCCGGAAAGGTCGAGTTGTGGGTTTAAACTTGTCCGGTTTGAGGAGAACCCGGTTGGGTAGCGTTAACCCAAGTTTTGAAGTGGGTTCTCTTGTGAATTTGACACTGTTAGAATCGTTTAATGCTTCTGGGTTTGTGCTTAATGGGTCTATTCCTGAGTGGTTGGGTGGGAGACTGAGGGTGTTGGATGTTCTTGACCTTAGGTTTTGTTCTATAACGGGTTGGATTCCTTATTCAATTGGGAAGTTGAGTGTGTTGAAGGTTTTGGTGCTTTCAGGGAATTTTCTCACTGGTAGAATGCCTTCAACGCTTGGGAACCTCACAAGGTTGTCTGTTCTTGATCTCTCTGCTAACTCTCTTTCAGGCTATGTGCCTGCTTCTGTGTCTATACTTGGGAATCTCACAAGGCTTGATCTTTCTGGCAATTATTTTTCTGGGTCTGTTCCACCTGAACTGGGTGCACTTTCGAGTCTTCAAAAGTTGAACCTTTCTGGGAATTCTTTTACTGGGTCGATTCCCTCTCAACTGGGTAACCTTTTTGAGTTGGTTGAGGTTGATCTTAGTATGAATTTTCTGTCGGGTTCGTTTCCAGGTGATTTGTTTTTCTCTCGGCTCTTGGCCCTCGAGGTTCTCATTCTCAGAGGGCCAAGAGCCGAGAGAAAAAAAAATCG

>PvRGL346

GGATATGGGAAAGTTTACAAGTGTAATCTTGATCACACACCAGTAGCTGTAAAGGTTCTGCATCAGGATGCAATCAACAAGAAAGAGGAGTTTCTAAAAGAGGTTGAGATTCTTAGTCAACTGCATCACCCTAATATGGTTTTGTTAGTTGGAGCCTGTCCTGAGAATGGTTGCCTAGTTTATGAATACCTGGAAAATGGAAGTCTAGAAGACTATCTTCTCAACAAAAATAGAAAACCACCACTTCCTTGGTTTTTTCGATTTCGCATAGTTTTTGAAATGGCTTGTGGACTTTCGTTCTTGCATAATTCGAAGCCAGAGCCGATTGTCCATCGAGATATAAAGCCTGGCAACATTTTGTTAGACAGAAATTATGTGAGCAAAATTTCTGATGTTGGGCTAGCTAAACTCCTTGCAGAAGTCGTGCCTGACAATATTACAGACTACCGAGAATCAGTCCTTGCCGGTACTTTGCATTACATGGACCCAGAATATCAGAGAACTGGCACTGTTCGACCAAAATCAGATGTATATGCATTTGGAGTTATAACTCTCCAATTAATAACTGGTCGCCATGCACGTGGGCTCATTTTGATTGTTGAAGATGCAATTAGAAATGGCTCCTTCCGTGATTTTTTAGATTCATCAGCTGGAGATTGGCCATTGGATGAGACAGTGGAATTGGCTCANATTGCTCTTAAATGCACAGCACTTAGATGCAGAGATAGACCAGAACTTGATACTGAAGTTCTTCCACTGCTCGAAAGATTTTCTAATGCGGCAAATGCTAGTGCAAGGATAGGAAGAAATAGTGTAAGTGCACCAAGCCAGTATTATTGTCCAATCCTTCANNGAATCATGGATGATCCATATATTGCTG

>PvRGL347

CTTCTTGATGATGAGATGGTTGCTCATGTTAGCGATTTTGGCTTAGCAAGACTTCTCTCGACAATCAACAACTCTCACAACCTTAGCACAAGTGGAATAAAAGGGACTATAGGTTATTCTCCCCCAGAGTATGGAGCCAGCTTTCAGGTGTCAACAAAAGGTGACATCTACAGTTTTGGTATTCTAATTCTGGAGATGTTAACTGGGAAACGACCGACAGAAGAAATGTTTAAAGATGGACACAGCCTCCATAACTATGCTAAAAATTCATTTCCAAATAACTTGTTGGATATTGTTGATGCAACTCTTGTTCCAATGGAAAATGAATCTCCTACAATGACTCTAACAGAACAGCACAACATCTCTGAGATTGTAGATCATTTTCATCCTAATACCAATAAGTGTTTGTTTTCATTATTTAAGATTGGTCTAGCTTGCTCAGTGGAGTCACCAGGAGAAAGGATGCTTATGATGGAAGTCACCACGCAGCTAAATATGATTAGAAATGCTTTTTATGCTCGACGGATCAGAGGTAGATATAGCTGAAAATAAGGTGGTACCTGAACCGTAATTAGGGGTGGCAATCCGTCCACCCTTCTTCATTTAAGTCAGGTTAATTTTTGAACTCATCAACCCACATTGGTTCGACCTACCTAGTATGTCAATCCTCCAAATTCCAGGCGGGACGAGACATAACGAACTAGTTTGTCAATTTGTCCTTTTATATTTATAATATTTTTGAAGAAAGATGTCATGCTTTTG

>PvRGL348

ACGGCCATAATCTTTTGTCATCGATTCTTTCTTCGACAATCTCACGCAAAGAACGACAGAAGGACCATTGCAACAGTTTGCATGTTCCTTGCTGGGAAGGTTGAAGAGACTCCCCGGCCACTAAAGGATGTTATTCTCGTTTCTTATGAGATCATTCATAAGAAGGATCCTGCTGCTGCACAGAGGATAAAGCAGAAGGAAGTGTATGAGCAGCAAAAAGAATTAATTTTACTTGGAGAGAGGGTTGTGCTTGCGACTTTAGCTTTTGATCTGAATGTTCAACATCCATATAAACCTCTTGTGGAGGCCATAAAGAAGTTCAATGTTGCAAAGAATGCCCTTGCTCAAGTTGCGTGGAACTTTGTTAATGATGGGCTGAGGACATCGCTCTGCCTGCAATTTAAGCCACATCATATTGCGGCAGGTGCCATTTTCCTGGCTGCTAAGTTCTTGAAAGTGAAGCTTCCATCAGATGGTGAGAAGGTTTGGTGGCAAGAGTTTGATGTCACCCCACGTCAATTAGAGGAAGTTAGCAATCAAATGCTGGAACTCTATGAGCAGAACAGACTTCCACCAGCACAGGGAAGTGAAGTAGAAGGAAGTGCTGGAGGAACAAGATCTACTACAAAGGCGCCTGCTGTGAATGAGGAGCAAGCGTCAAAGCAAATATCATCTCAAGCCCCCCAACACTCATCTGTAGAAAGAACTGTGGTGCCGCAAAGAGGAACTGAGAACCAAAGCAACGATGGAAGTGCAGAAATGGGCAGTGACATTACTGATCACAAGATGGACTTGGATAATAGTGAGTCTCGGAATTCAGAGCAGTTGACT

>PvRGL349

GAGATATCAGGGGTTATTCCAGACTCCCTTGCCAACCTCCTCAATTTAAACCTGATTGATTTGTCAAGAAACAAACTAATGGGGAGAATACCTACCAGTTTTGGGAATTTGCAAAGTCTGCTTTACATGGACTTATCAAGCAACCAACTCAATGAAAGCAATCCTATGGAAATCCTCAATCTCCCAACTTTGAGCAATGTTTTGAATTTGTCAATGAACCCTTTTGAGTGGACCAATACCTCAAATTGGGAGATTGAGTGGTGTTGCCTCCATTATTGACTTTTCTAGCAACCAATTGGATGGTGACATCCCCAGTTCATTCAGCAATTGTCTCAGCTTAGAAAATTTATTTTTGACCAGGAATCAGCTTTCAGGTTCCATTCCAAAGGCTCTTGGAGAAGTGAGAGGCTTGGAAACTTTGAACCTATCCTCCAACCAACTCTCTGGAACCATTCCTGTTGAACTTCAAAACTTACAGGTGCTTAGGCTCTTAAACTTCTCGTATAATGATTTAGAAGGAGACATTCCAAGTGGTGGAGTTTTCCAAAATCTCTCTGCTGTACATTTAGAAGGCAATGGAAATCTCTGCTTGCAAGCCCCGTGTGTGAATCGTGGCGAAGGAAGAAGAAATATAAGACATTACATTATCGTAGCCGTTTCAGTAGCATTGGCACTGTGTCTCACAATTGGCTCAATACTATATATAAAGATTAGAAAGGTGAAGGTATCATCATCATCATCATCATCTGAACAGTTAGCCTCTTGCACTAATGATCTCTTATGATGAGCTTCATTTAGCAACTGAGGAAATCAACCAGGAAACTTACTAGGTGTTGGGAGCTTTGGCTCAGTTTACAAAGGCAATCTATGTTATGGAACTACTGTTGC

>PvRGL350

GGAAGGTACATATGTGGCAAGCATGTTGAGCAGAAGCAAACAGATGCTGAATCCAGCAATCACACGGTTACCATTCCAAAAGCCTCAACTCTTGGGCCTGATGTATTTGAAATGGATAAGCCTGTTATTTTTACATATGAAGAGATTTTCTCCACATCTGATGGTTTCTCTGATTCAAATCTACTTGGGCATGGAACATATGGTTCTGTTTATTATAGCCTCCTTCGTGATCAGGAAGTTGCTATTAAAAGAATGACTGCTACTAAAACAAAAGAATTTTTGTCAGAGATGAAAGTTTTGTGCAAGGTTCATCATGCTAATCTGGTAGAATTGATTGGCTATGCAGCTAGTCACGAGGAGCTTTTCCTAGTTTATGAATATGCTCAGAAGGGTTCACTTAGAAGCCATTTGCATGATCCTAATAGTAAGGGTCATTCCCCACTTTCTTGGATCATGAGGGTGCAGATTACGCTTGATGCTGCTAGGGGCCTCGAATACATACACGAGCACACAAAAACTCATTATGTACACCGTGATATCAAAACCAGTAACATATTACTTGATGCTTCCTTTAGAGCAAAGATTTCAGATTTCGGGTTAGCAAAACTTGTTGGGAAAACAAATGAGGGAGAAATATCAACTACCAAAGTTGTTGGTACATATGGATATCTTGCCCCAGAATATTTGAGTGAGGGTCTTGCGACAGCTAAAAGTGATGTCTATGCATTTGGGGTTGTCCTTTTCGAGATTATAT

>PvRGL351
[truncated: 12,262 more chars]
